# Speciation‐Controlled C–F Bond Functionalization Enabled by Zwitterionic Pnictinidenes

**DOI:** 10.1002/anie.6579940

**Published:** 2026-07-21

**Authors:** Irene Sánchez‐Sordo, Sergio Fernandez, Selwin Fernando, Diego M. Andrada, Oriol Planas

**Affiliations:** ^1^ Department of Chemistry Imperial College London Molecular Sciences Research Hub London UK; ^2^ Department of Chemistry Queen Mary University of London London UK; ^3^ General and Inorganic Chemistry Department University of Saarland Saarbrücken Germany

**Keywords:** antimony, bismuth, catalysis, defluorofunctionalization, redox catalysis, redox neutral

## Abstract

Low‐valent pnictogen systems have recently emerged as redox catalysts for C–F activation. However, catalytic turnover has remained largely restricted to hydrodefluorination pathways reliant on metallomimetic reductive elimination. Herein, we report a defluorinative functionalization platform mediated by zwitterionic pnictinidenes that enables the formation of diverse C–X bonds (X = C, O, S, N, P, Se) from polyfluoro(hetero)arenes under mild conditions. Combined experimental and DFT studies reveal that, although a Sb(I)/Sb(III) redox cycle could be accessed, catalysis instead proceeds through Sb(III)–F species that promote C–F substitution via a redox‐neutral manifold. These findings establish catalyst speciation as a decisive mechanistic branch point, showing how strategies to modulate ligand scrambling events may enable alternative pnictogen‐based redox or redox‐neutral manifolds.

## Graphical abstract

Zwitterionic pnictinidenes turn C–F activation into C–X bond construction, enabling broad defluorinative functionalization of polyfluoro(hetero)arenes under mild conditions. Rather than following a metallomimetic Sb(I)/Sb(III) redox turnover, catalysis is redirected by ligand scrambling into a redox‐neutral manifold, revealing catalyst speciation as a powerful switch in pnictogen chemistry.

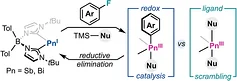

## 1 Introduction

Fluorocarbons are central substrates to modern materials and pharmaceuticals, enabling applications ranging from polymers and liquid crystals to agrochemicals and refrigerants [1, 2, 3, 4, 5, 6, 7, 8, 9, 10]. Yet the exceptional strength of the C–F bond that underpins their value also renders fluorinated motifs environmentally persistent [11, 12], making their selective activation and functionalization both a synthetic opportunity and a sustainability imperative [13, 14, 15, 16, 17, 18, 19]. While both metal‐catalyzed and metal‐free approaches for C–F bond functionalization have been developed [20], transition metals have long dominated the field. These systems typically rely on oxidative addition to generate nucleophilic metal‐fluoride intermediates, which subsequently enable C–E bond formation (E = B, C, Si, N, P, O, S, Se) through fluorophile‐assisted turnover [21, 22, 23, 24, 25]. Indeed, transition‐metal‐fluoride complexes have been widely used as catalysts, as the incorporation of a fluoride ligand into the metal coordination sphere enhances its activity and alters reaction selectivity compared to other TM–halide analogs [26, 27].

**SCHEME 1:**
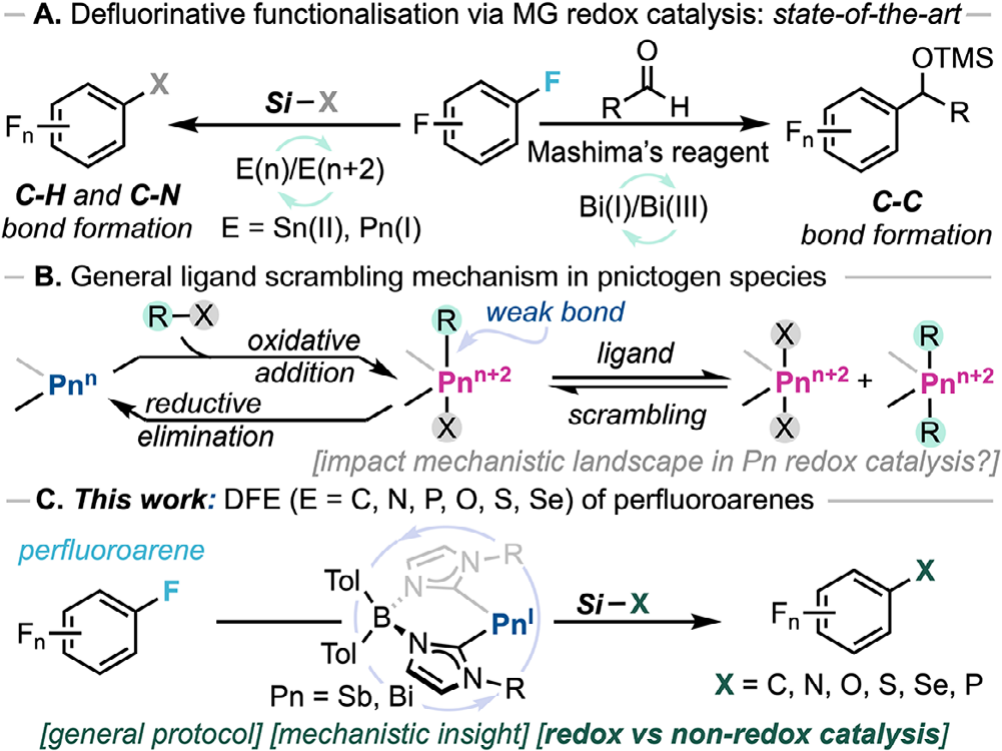
(A) Examples of low‐valent main‐group catalyzed defluorofunctionalization (DFF) processes. (B) Ligand scrambling processes of organopnictogens. (C) This work: Pn‐catalyzed defluorofunctionalization of polyfluoro(hetero)arenes for C–X (X = C, N, O, S, Se and P) bond formation.

Over the past decade, low‐valent main‐group species have emerged as viable alternatives to transition metals, challenging conventional assumptions about redox catalysis [28, 29, 30, 31]. Stoichiometric C–F bond activation of fluoro(hetero)arenes has been demonstrated across multiple elements of the *s* and *p* blocks [32, 33, 34, 35, 36, 37, 38, 39]. However, redox manifolds enabling catalytic C–F bond activation have only been achieved within the pnictogen (Pn) family and selected tetrel systems [40, 41, 42, 43, 44, 45], almost exclusively in the context of hydrodefluorination (Scheme 1A, left). These transformations typically invoke a metallomimetic C(*sp* ^2^)–H reductive elimination step following oxidative addition and transmetallation. Beyond hydrodefluorination, catalytic C–X (X = C, heteroatom) bond formation from main‐group‐mediated C–F activation remains strikingly limited. Only two examples of defluoroamination have been reported, both invoking phosphine‐based reductive elimination pathways [40, 41], while a recent Bi(I)‐catalyzed reductive arylation proceeds via migratory insertion followed by Si–O reductive elimination (Scheme 1A, right) [46]. Despite these advances, the scope of bonds accessible remains limited to three: C–H, C–C, and C–N bonds. Moreover, while low‐valent pnictogen systems have recently been interpreted through a metallomimetic redox lens [47, 48, 49], the possibility that catalyst speciation modifies the catalytic manifold remains largely unexplored. In transition‐metal catalysis, speciation is a key factor governing turnover, selectivity, and even the operative mechanism [50, 51]. Given that ligand scrambling is an intrinsic feature of heavier organopnictogen species due to their weak Pn–C bonds [52, 53, 54, 55], catalyst speciation is unlikely to be static under catalytic conditions following oxidative addition (Scheme 1B) [41, 44]. This raises a fundamental question: does low‐valent pnictogen redox catalysis always operate through metallomimetic turnover, or can speciation divert the system into alternative, redox‐neutral regimes? Elucidating how such dynamic processes redirect formally accessible redox pathways and harnessing this to enable new bond‐forming pathways define a central frontier in *p*‐block redox chemistry.

Recently, we reported a family of zwitterionic heavier pnictinidene species that demonstrated the ability to promote catalytic hydrodefluorination of polyfluorinated (hetero)arenes via a Pn(I)/Pn(III) (Pn = Sb, Bi) redox cycle [43]. A key feature of this process is a transition‐metal‐like reductive elimination step from an Ar–Pn(III)–hydride intermediate, forging new C(sp*^2^*)–H bonds. Encouraged by these results and recognizing the limited precedent for main‐group redox manifolds beyond hydrodefluorination, we set out to examine whether alternative C–X bonds could be constructed through the same mechanism (Scheme 1B). Herein, we disclose a methodology for C–X bond formation (X = C, N, P, O, S, Se) catalyzed by bis(NHC)borate‐stabilized pnictinidenes (Scheme 1B). Beyond establishing broad substrate scope, mechanistic investigations uncover a divergent catalytic landscape in which a redox pathway ultimately diverges into a redox‐neutral manifold via ligand redistribution.

## 2 Results and Discussion

On the onset of our investigation, C–O bond formation was explored. Catalytic defluoroalkoxylation was tested using pentafluoropyridine and 1.1 equiv. of trimethyl(*p*‐tolyloxy)silane in the presence of 5 mol% of zwitterionic stibinidene **1a** (Table 1, Entry 1). Gratifyingly, the reaction furnished mono‐alkoxylated product **2a** in quantitative yield, together with trace amounts of dialkoxylated product **2a’**. These results encouraged us to further explore the reaction conditions and limitations of this methodology.

**TABLE 1:** Optimization of reaction conditions for the Sb‐catalyzed defluoroalkoxylation of pentafluoropyridine.^a^

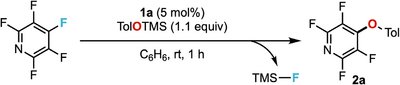		
Entry	Deviation from standard conditions	2a, yield (%)
1	None	> 95
2	2 mol%	> 95
3	0.5 mol%	> 95
4	CH_3_CN as solvent	76
5	Et_2_O as solvent	75
6	**1b** instead of **1a**	> 95
7	**3** as catalyst instead of **1a**	> 95
8	**4** as catalyst instead of **1a**	n.d.
9	**5** as catalyst instead of **1a**	< 5
10	**TASF** as catalyst instead of **1a**	< 5
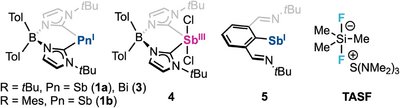		

^a^ 0.2 mmol of pentafluoropyridine. Yields determined by ^19^F NMR using *α,α,α* ‐trifluorotoluene (0.50 equiv.) as internal standard.

Using pentafluoropyridine and trimethyl(*p*‐tolyloxy)silane as model substrates, stibinidene **1a** catalyzed the formation of **2a** in quantitative yield within 1 h at a loading of 2 mol% (Entry 2). Remarkably, the reaction proceeded in quantitative yield even at a reduced loading of 0.5 mol% (Entry 3), and **2a** was isolated on multigram scale (1.23 g, 96%) using only 14 mg (0.5 mol%) of catalyst **1a**. Optimization studies also demonstrated broad solvent compatibility (see Table S1 for full details), with the reaction performing well in polar and apolar solvents such as CH_3_CN and Et_2_O (entries 4 and 5). Notably, mesityl stibinidene **1b** and bismuthinidene complex **3** provided similar results (Entries 6 and 7), while Sb(III) dichloride species **4** did not afford reactivity (Entry 8). Interestingly, *N,C,N*‐pincer‐supported Sb(I) species **5** delivered only negligible yields under identical conditions (Entry 9), highlighting the superior reactivity of zwitterionic species **1** and **3** and underscoring their potential as viable alternatives to conventional *N,C,N*‐pincer‐Pn(I) platforms. To probe the potential role of fluoride anions in catalyzing the defluoroalkoxylation step in our protocol [56], soluble fluoride (**TASF**, Entry 10) was employed in catalytic amounts. However, < 5% conversion was observed, ruling out free fluoride as the active catalyst and suggesting the involvement of a pnictogen‐based species. Indeed, when the reaction was performed in absence of **1a** no product was detected (Table S2). Using pentafluoropyridine as a model substrate, a range of trimethyl(aryloxy)silanes was tested with 2 mol% of catalyst **1a**, which was identified as the optimal loading, as reduction to 0.5 mol% required impractical amounts of catalyst (Table 2). The protocol bodes well with both electron‐donating (**2b**, 75%) and electron‐withdrawing substituents (**2c** and **2d**, 96% and 56%, respectively). Furthermore, sterically hindered aryloxysilanes bearing *ortho*‐Me (**2e**), OSiMe_3_ (**2f**), and OMe (**2g**) groups were also well tolerated, delivering the corresponding defluoroalkoxylation products in excellent yields. Alkyloxysilanes were also compatible with this transformation, affording the product in moderate yield (**2h**, 59%). When an excess of aryloxysilane was employed, disubstitution occurred, leading to the corresponding defluorodialkoxylation product (**2a’**). In addition, other electron‐poor perfluoroaryl compounds were found to undergo defluoroaryloxylation (**2i**–**k**), although in some cases only low yields were obtained (**2l–m**). By contrast, perfluoroarenes bearing electron‐donating groups resulted unreactive, similarly to what we previously observed in HDF reactions with catalytic amounts of pnictinidenes **1** and **3** [42]. Importantly, the reactivity in the presence of spin and radical traps was also evaluated, and the formation of the corresponding defluoroalkoxylation products proceeded unaffected, in excellent yields (Table S4).

**TABLE 2:** Scope of pnictinidene‐catalyzed defluorofunctionalization of fluorinated arenes and hexafluoropropene.^a^

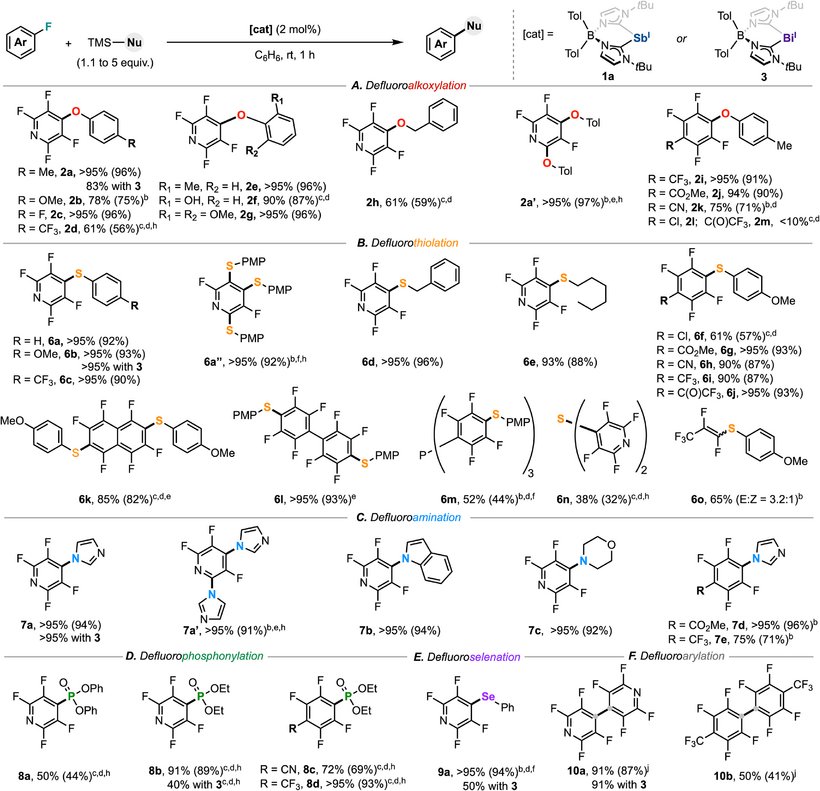

^a^ Reactions performed at 0.2 mmol of starting material. Yields calculated by quantitative ^19^F NMR using *α,α,α*‐trifluorotoluene as internal standard unless otherwise stated. Isolated yield in brackets.

^b^Reaction time: 2 h.

^c^ Reaction time: 16 h.

^d^Reaction temperature: 60°C.

^e^Reaction with 2.0 equiv. of TMS‐Nu.

^f^Reaction with 3.0 equiv. of TMS‐Nu.

^g^Reaction with 5.0 equiv. of TMS‐Nu.

^h^Reaction performed with 5 mol% of [cat].

^i^Reaction performed in CH_3_CN in presence of 1,4‐bis(trimethylsilyl)‐1,4‐dihydropyrazine (0.5 equiv.). Blank reactions were performed for all substrate classes and showed poor to no activity in each case (see Table S3).

Given the ability of Pn(III)–F species generated after oxidative addition to activate alkoxy‐ and hydrosilanes, zwitterionic pnictinidene systems were also tested in other C–X bond‐forming reactions. Defluorothiolation was briefly optimized (Table S2) using trimethyl(*p*‐tolylthio)silane as coupling partner, showing comparable efficiency to the defluoroalkoxylation process and proceeding under similar reaction conditions (Table 2B). In this case, while *N,C,N*‐pincer‐supported Sb(I) species **5** exhibited similar reactivity to the zwitterionic species **1** in the formation of the monothiolation product **6a** (see Entry 7, Table S2), our catalyst demonstrated superior activity, being able to promote up to three consecutive defluorothiolation steps within the same arene scaffold to deliver pyridine **6a″** in 92% isolated yield. The reaction also tolerated thiols bearing both electron‐donating (**6b**, 93%) and electron‐withdrawing (**6c**, 90%) substituents, as well as thioalkyl groups, including benzyl (**6d**, 96%) and hexyl (**6e**, 88%) chains. Moreover, other perfluoroarenes proved compatible with the protocol (**6f**, 57%), particularly those incorporating strong electron‐withdrawing substituents in the *para*‐position, such as ester (**6g**, 93%), cyano (**6h**, 87%), trifluoromethyl (**6i**, 87%) and trifluoroacetyl (**6j**, 93%). Consistent with the alkoxylation methodology, partially fluorinated and electron‐rich perfluoroarenes were unreactive towards defluorothiolation, representing the main limitation of this protocol. Nevertheless, perfluorinated *π*‐extended arenes (**6k**, 82%), biphenyl (**6l**, 93%) and phosphine (**6m**, 44%) derivatives underwent thiolation successfully. Interestingly, when bis(trimethylsilyl)sulfide was used, bis(tetrafluoropyridin‐4‐yl)sulfide **6n** was obtained, albeit in moderate yields. In addition, a gas considered a polyfluoroalkyl substance (PFAS) such as hexafluoropropene is also reactive under catalytic conditions, providing a mixture of stereoisomers **6o** (*E/Z* ratio of 3.2:1, 65%) from thiolation of the terminal C(sp^2^)–F bond. In this case, reactivity in presence of spin and radical traps also proceeded unaffected, discarding the intermediacy of open‐shell intermediates (see Table S5), which were also discarded in alkoxylation reactions. It is important to note that Crimmin and co‐workers recently reported a similar methodology for C–F bond thiolation and hydrogenation using group 13 (Al, Ga) fluorides in presence of silanes and silyl thioethers [57], yet this method required harsher conditions, longer reaction times and an external promoter (NaBArF).

The efficiency and mild conditions of the alkoxylation and thiolation protocols prompted us to investigate other C–heteroatom bond‐forming reactions. Notably, 1‐(trimethylsilyl)‐1*H*‐imidazole proved to be an excellent coupling partner to enable the formation of C–N bonds with pentafluoropyridine, yielding **7a** in 94% when stibinidene **1a** was used as catalyst. Moreover, reaction outcomes could be finely controlled by adjusting the stoichiometry of 1‐(trimethylsilyl)‐1*H*‐imidazole, affording the 2,4‐substituted product **7a’** in 91% yield. Other trimethylsilyl‐substituted *N*‐nucleophiles were also well tolerated in this transformation, including indole (**7b**, 94%) and morpholine (**7c**, 92%). Furthermore, perfluoroarenes bearing electron‐withdrawing groups in the *para* position proved compatible with the protocol, affording the corresponding products in excellent yields (**7d**, 96%; **7e**, 71%). The scope was also expanded to C–P bond formation (Table 1D), which was achieved with a range of trimethylsilyl phosphite derivatives (**8a–d**), affording the corresponding products in good to excellent yields. Likewise, C–Se bond formation (Table 1E) was successfully demonstrated, delivering **9a** in 94% isolated yield. In C–P and C–Se bond forming reactions, **3** resulted less effective, possibly due to an overall greater instability of the putative Bi(III) intermediates, leading to catalyst decomposition as we observed in previous studies [43]. Overall, the formation of C–X bonds (X = O, S, N, P, Se) highlights the versatility of the protocol and its potential as a general platform for C–F bond activation in perfluoroarenes, resulting in an unprecedented main‐group‐catalyzed C–heteroatom bond‐forming reaction.

The catalytic formation of C–C bonds with alkyl‐, aryl‐ and alkynylsilanes with perfluoroarenes proved more challenging. Attempts to promote reactivity employing harsher conditions, higher catalyst loading, or extended reaction times were unsuccessful, and no desired products were obtained (see unsuccessful substrates in Section 3.9.3). However, small amounts of dimeric product **10a** were observed when the reaction was attempted in CH_3_CN, consistent with stoichiometric reactivity previously reported by Slattery and co‐workers [41]. This process is speculated to proceed via ligand scrambling, followed by reductive elimination from Sb(III) diaryl species [44], and we hypothesised that the accompanying formation of Sb(III) difluoride species could inhibit catalytic turnover (see Supporting Information, Section 4.4.1). Encouragingly, the use of Mashima's pyridazine reagent as an external reductant enabled the formation of biaryl products **10a** and **10b** in good yields [58], providing a proof‐of‐concept example of reductive coupling to catalytically forge C–C bonds, extending this methodology beyond C–heteroatom bond formation.

**FIGURE 1:**
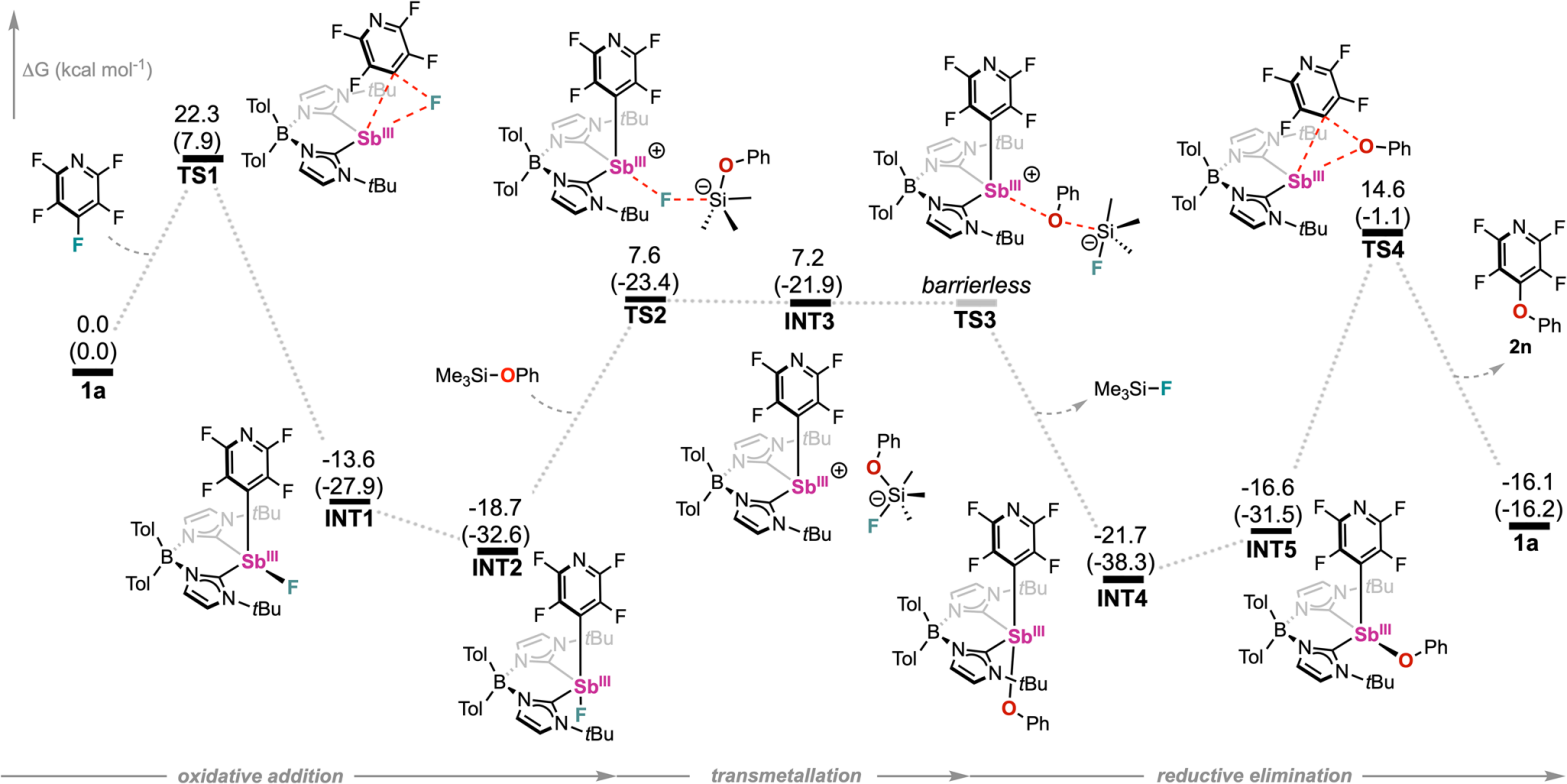
Gibbs energy (ΔG) for the defluoroalkoxylation of pentafluoropyridine with PhOSiMe_3_ proceeding via Sb(I)/Sb(III) redox catalysis with stibinidene species 1a. Enthalpy (ΔH) in parentheses. Calculations were performed at the CPCM(benzene)‐PBE0‐D3BJ/def2‐TZVPP//CPCM(benzene)‐PBE0‐D3BJ/def2‐SVP level of theory.

**FIGURE 2:**
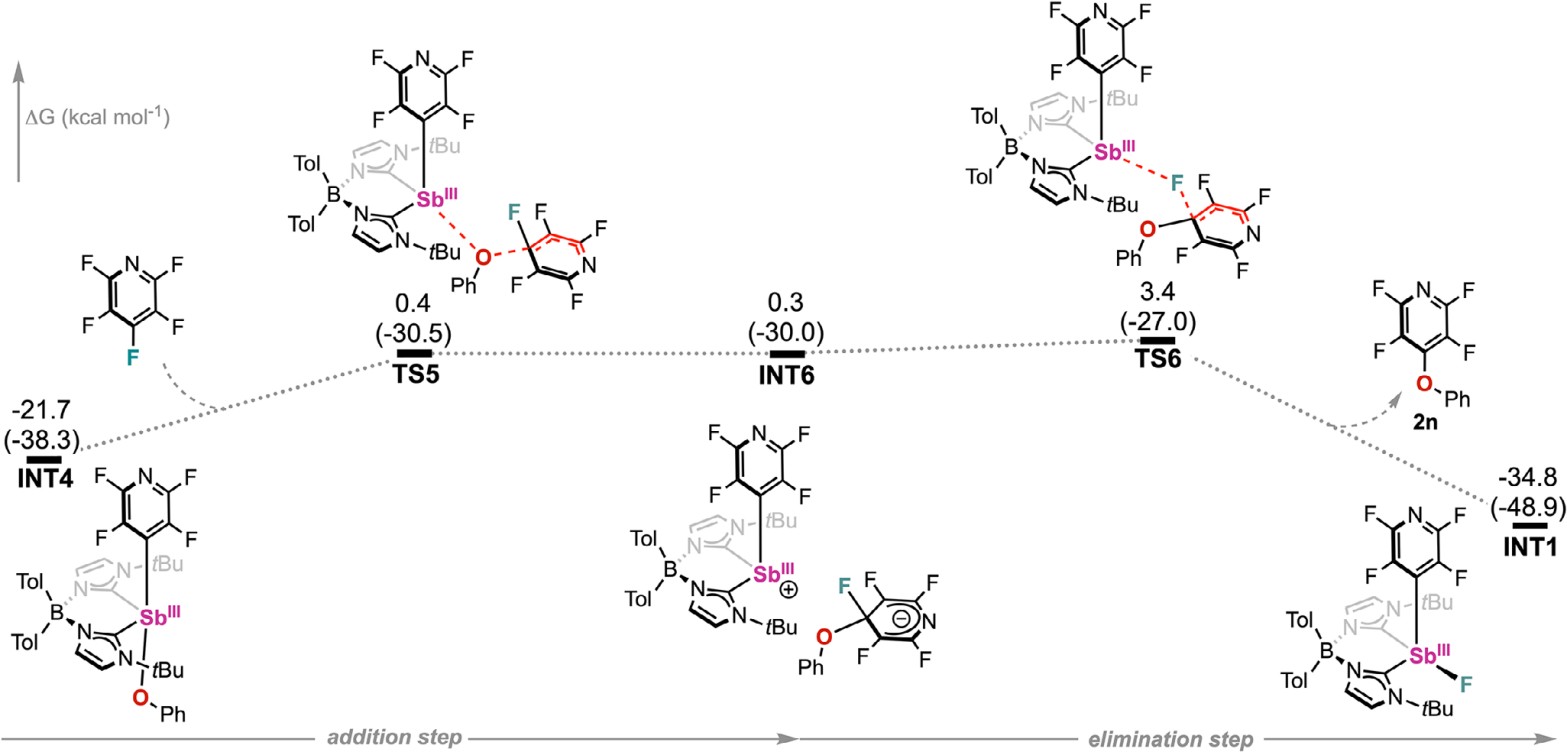
Gibbs energy (Δ*G*) for an S_N_Ar‐type mechanism of the Sb(III)‐fluoride‐catalyzed defluoroalkoxylation of pentafluoropyridine with PhOSiMe_3_. Enthalpy (Δ*H*) in parentheses. All calculations were performed at the CPCM(benzene)‐PBE0‐D3BJ/def2‐TZVPP//CPCM(benzene)‐PBE0‐D3BJ/def2‐SVP level of theory.

To gain deeper insight into the underlying mechanism of pnictinidene‐catalyzed defluorofunctionalization, DFT calculations were carried out on several plausible pathways, focusing on Sb species **1a** (see Figures 1 and 2; for Bi species **3** see Supporting Information). To do so, the transformation of pentafluoropyridine with trimethyl(phenyloxy)silane was selected as a representative case study. Based on previous studies [40, 41, 42, 43], a catalytic pathway involving Sb redox cycling was first evaluated [43, 59, 60, 61, 62, 63, 64]. In this case, the reaction begins with the oxidative addition of Sb(I) species **1a** to the C4–F bond of the pyridine ring, resulting in Sb(III) intermediate **INT1** (Δ*G* = –13.6 kcal mol^−1^). This step is thermodynamically favourable, with a connecting transition state (**TS1**) located at +22.3 kcal mol^−1^ relative to the reactants. After oxidative addition, **INT1** features the fluorine atom in the equatorial position, and undergoes isomerization to the more thermodynamically favoured species **INT2** (ΔG = –18.7 kcal mol^−1^), in which the fluorine occupies an axial site. Subsequent ligand metathesis with PhOSiMe_3_ proceeds in a stepwise manner. Transmetallation begins with silane‐mediated fluoride abstraction, forming the fluorosilicate anion [PhOSiMe_3_F]^−^ and the corresponding cationic Sb(III) intermediate (**INT3**), as a contacted ion‐pair. This step is endergonic by +25.9 kcal mol^−1^ and it shows a Gibbs activation energy of 26.3 kcal mol^−1^ (**TS2**, Δ*G* = 7.6 kcal mol^–^^1^). **INT3** (Δ*G* = +7.2 kcal mol^−1^), which is a relatively high‐energy intermediate, rapidly evolves to the Sb(III)–OPh phenyloxy species (**INT4,** Δ*G* = –21.7 kcal mol^−1^) in a barrierless process (**TS3**). All attempts to locate a transition state were unsuccessful. Additionally, other plausible mechanistic scenarios for the conversion of **INT2** to **INT4** were systematically explored computationally (e.g., four‐membered ring concerted transmetallation) [42], but all attempts converged back to the silicate‐derived pathway providing no viable alternative to the fluoride‐abstraction‐initiated manifold described above. To undergo metallomimetic concerted reductive elimination, **INT4** must isomerize first, exchanging the phenyloxy ligand from a thermodynamically favoured axial position to an equatorial site, resulting in **INT5** (Δ*G* = –16.6 kcal mol^−1^). Finally, C–O reductive elimination from **INT5** yields (phenyloxy)tetrafluoropyridine **2n** and regenerates the low‐valent Sb catalyst **1a**, with a barrier (**TS4**) of +36.3 kcal mol^−1^. Overall, the reaction is exergonic by ‐16.1 kcal mol^−1^, yet the isomerization/reductive elimination step from **INT4** proved thermodynamically disfavoured by 5.6 kcal mol^−1^. Moreover, the calculated barrier of +36.3 kcal mol^−1^ including both isomerization and C–O reductive elimination (**TS2**) is not consistent with the optimized reaction conditions (see Table 1), suggesting that a hypothetical Sb(I)/Sb(III) redox cycle proceeding through a metallomimetic reductive elimination is unlikely to be the operative mechanism in this transformation. Based on these results, which indicate a redox mechanism in which Sb revolves between oxidation states +1 and +3 is unlikely, a mechanism involving a redox‐neutral step after C–F bond activation was evaluated for transformations leading to C–X (X = N, O, S, Se and P) bond formation, starting from Sb(III)‐OPh species **INT4** as model substrate (Figure 2) for C–O bond formation. Thus, following the pathway depicted in Figure 1, after oxidative addition and transmetallation, **INT4** can undergo S_N_Ar‐type reactivity with pentafluoropyridine for C–O bond formation, with a transition state (**TS5**) located with a relative Gibbs free energy of Δ*G* = +0.4 kcal mol^–^^1^. Interestingly, this corresponds to a low Gibbs activation barrier (ΔΔ*G* = +22.1Tab kcal mol^−1^) and leads to the corresponding Meisenheimer‐like intermediate **INT6** (Δ*G* = +0.3 kcal mol^−1^). Subsequent C–F bond cleavage proceeds to Sb–F bond formation (**TS6**, Δ*G* = +3.4 kcal mol^−1^), leading to **INT1** and the product **2n** in an overall thermodynamically favoured manner (ΔΔ*G* = −18.7 kcal mol^−1^) when compared to the potential energy surface in Figure 1. The overall barrier of this process is 25.1 kcal mol^–^^1^, in better agreement with reaction conditions depicted in Table 1.

**SCHEME 2:**
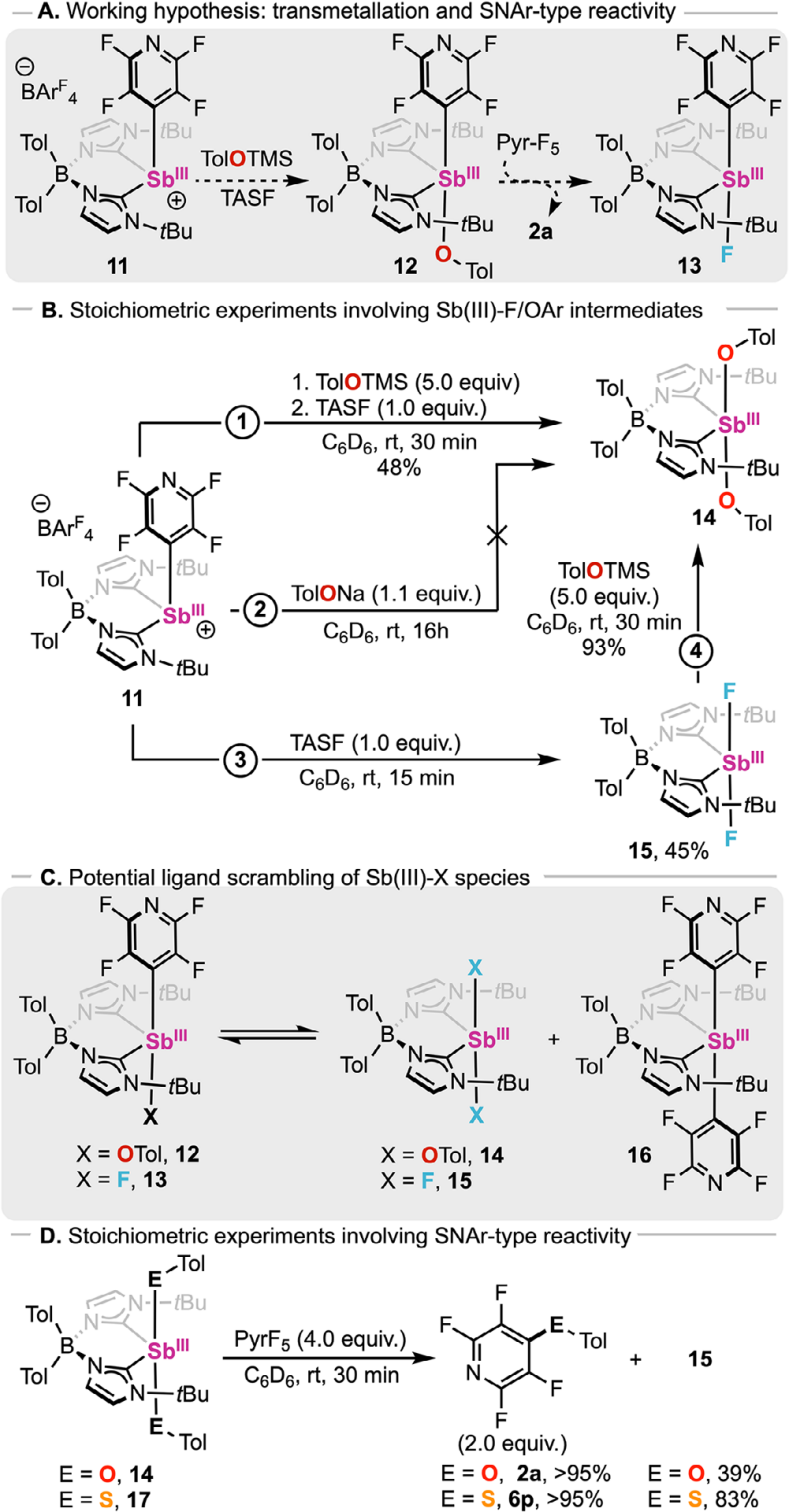
(A) Working hypothesis and potential isolable Sb(III)‐X species. (B) Stoichiometric studies on transmetallation and reductive elimination of C─O bonds from zwitterionic Sb(III) complexes. (C) Proposed ligand scrambling of Sb‐F species. (D) S_N_Ar of Sb(XAr)_2_ species to pentafluoropyridine.

To support the computational results presented above, stoichiometric studies were carried out aiming at isolating a putative Sb–OTol intermediate **12** (Scheme 2A), analogous to the computed species **INT4**. Access to such an intermediate would allow direct evaluation of its S_N_Ar‐like reactivity toward pentafluoropyridine, where formation of the defluoroalkoxylation product **2a** accompanied by the corresponding Sb–F species **13** would be anticipated. In an initial attempt, treatment of tetrafluoropyridinyl–Sb(III) complex **11** with excess trimethyl(p‐tolyloxy)silane in the absence of a fluoride source resulted in no reaction, consistent with our previous observations (see Figures S2, S3) and indicating fluoride anions are needed to promote transmetallation of the alkoxide from Si to Sb. In fact, the addition of 1.0 equiv of **TASF** in the presence of 5.0 equiv. of trimethyl(p‐tolyloxy)silane at room temperature enabled reactivity (Scheme 2B, Pathway 1), but neither product **2a** nor the targeted F_4_Pyr–Sb(III)–OTol species **12** was detected. Instead, NMR spectroscopy revealed formation of a new Sb(III) dialkoxide complex **14** (48%) together with two major fluoride‐containing byproducts, likely originating from decomposition of the Sb–PyrF_4_ moiety (see Supporting Information, Section 4.3.1). The absence of stibinidene **1a** in the reaction mixture further supports a redox‐neutral pathway following C–F bond activation and C–O bond formation, as proposed in Figure 2.

To further probe this hypothesis, a stoichiometric reaction between Sb(III) species **11** and sodium 4‐methylphenoxide was attempted (Scheme 2B, Pathway 2), but no reactivity was observed even after prolonged reaction times. In contrast, treatment of Sb(III) species **11** with 1.0 equivalent of TASF in C_6_D_6_ generated a clear solution and a cream‐coloured precipitate within 30 min. NMR analysis of the solution confirmed the formation of Sb(III) difluoride species **15** in 45% yield (Scheme 2B, Pathway 3), supporting the ligand scrambling process depicted in Scheme 2C. Based on mass balance and reaction stoichiometry, the cream‐coloured precipitate is assigned to the bis(tetrafluoropyridyl)‐Sb(III) intermediate **16**. However, attempts to characterise **16** were unsuccessful due to its poor solubility at low temperature and pronounced instability at room temperature: attempts to redissolve the precipitate in polar solvents such as THF‐d_8_ or CD_3_CN led to rapid decomposition, affording multiple species, among which perfluoro‐4,4′‐bipyridine **10a** was identified (see Figures S18–S21). Although **16** could not be unambiguously identified, it is proposed as a transient intermediate, consistent with the observed reactivity, DFT calculations and related pnictogen‐mediated C(sp^2^)–C(sp^2^) reductive elimination chemistry reported by Slattery [41], Cornella [44], and McNally [65, 66]. Furthermore, exposure of difluoride **15** to 5.0 equiv of trimethyl(*p*‐tolyloxy)silane afforded dialkoxide **14** in 93% yield (Scheme 2B, Pathway 4), demonstrating that ligand exchange from Sb–F to Sb–OTol is chemically feasible. While formation and subsequent scrambling of a short‐lived F_4_Pyr–Sb(III)–OTol intermediate **12** cannot be definitively excluded, combined experimental and computational evidence points to an initial formation of F_4_Pyr–Sb(III)–F species **13**, ligand scrambling and ligand metathesis leading to the formation of Sb(III) dialkoxide **14**. Attention was then focused on establishing whether dialkoxide **14** could undergo an S_N_Ar‐type reaction with pentafluoropyridine to generate **2a** in a stoichiometric manner. Indeed, treatment of **14** with excess pentafluoropyridine (Scheme 2D) produced Sb(III) difluoride **15** and product **2a** in 39% and > 95% yield, respectively, together with free borate ligand as byproduct. Analogously, the corresponding disulfide **17** afforded **15** and **6p** in 83% and 95% yield [42, 47], respectively.

Finally, the catalytic relevance of species **14** and **15** was evaluated in the model defluoroalkoxylation of pentafluoropyridine with trimethyl(*p*‐tolyloxy)silane (Table 3, Entries 1 and 2). In both cases, when these complexes were used with a catalyst loading of 5 mol% in benzene, product **2a** was obtained in almost quantitative yield, with only trace amounts of dialkoxylated product **2a’**. In contrast, simple Sb(III) salts such as SbF_3_ or Sb(OTol)_3_ were inactive under the same conditions (Table 3, Entries 3 and 4). These results highlight the key role of the supporting ligand framework and suggest enhanced nucleophilic character of the Sb–OR bond in tetracoordinate Sb(III) species such as **14**, in line with related observations reported by Crimmin for group 13 fluoride complexes [57].

**TABLE 3:** Sb‐catalyzed defluoroalkoxylation of pentafluoropyridine with Sb‐X nucleophiles.^a^

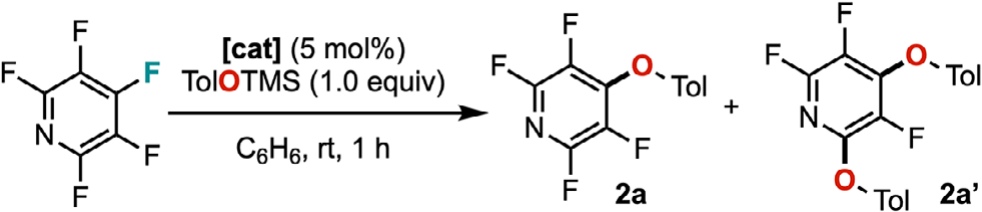			
Entry	[cat]	2a, yield (%)	2a’, yield (%)
1	**14**	> 95	< 5
2	**15**	> 95	< 5
3	SbF_3_	< 5	< 5
4	Sb(OTol)_3_	< 5	< 5

^a^ 0.2 mmol of pentafluoropyridine. Yields determined by ^19^F NMR using *α*,*α*,*α* ‐trifluorotoluene (0.50 equiv.) as internal standard.

Notably, when **2a** was subjected to the catalytic conditions to generate **2a’**, quantitative conversion was achieved only with **14** or **15** as catalysts, whereas stibinidene **1a** resulted completely inactive (see Supporting Information). These results further corroborate that defluorinative functionalization does not proceed via an antimony‐mediated C–X bond reductive elimination, but rather through Sb(III)‐mediated pathways leveraging the ability of fluoride to activate the corresponding organosilyl coupling partner.

**FIGURE 3:**
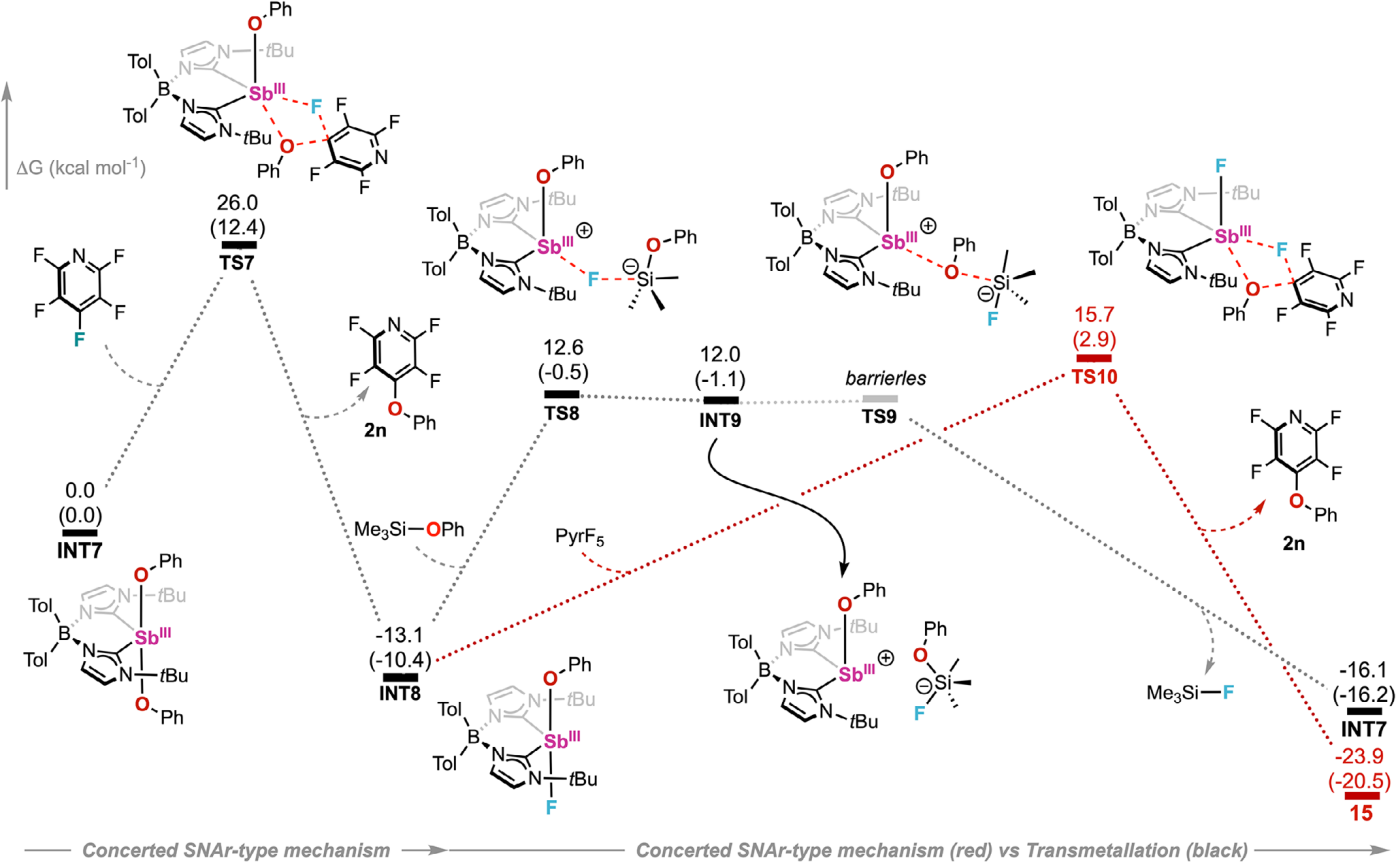
Gibbs energy (Δ*G*) for an S_N_Ar‐type mechanism. Enthalpy (Δ*H*) in parentheses. All calculations were performed at the CPCM(benzene)‐PBE0‐D3BJ/def2‐TZVPP//CPCM(benzene)‐PBE0‐D3BJ/def2‐SVP level of theory.

Considering the mechanistic results displayed in Scheme 2 as well as catalytic experiments in Table 3, the formation of **2n** by reaction of **INT7**, analogous to species **14**, and pentafluoropyridine was also studied computationally to gain a deeper understanding of the C–O bond forming event operative in this transformation (Figure 3). In this case, **INT7** undergoes S_N_Ar‐type reactivity with perfluoropyridine for C–O bond formation, with a transition state (**TS7**) located with a relative Gibbs free energy of *Δ*G = +26.0 kcal mol^−1^. Notably, **TS7** is a concerted transition state in which both C–O bond formation and C–F bond cleavage occur synchronously, which contrasts with the high energy Meisenheimer intermediate **INT6** obtained from **INT4** (Figure 2) through a stepwise S_N_Ar‐type mechanism. Eventually, **INT7** evolves to **INT8**, which was not detected experimentally, and it is hypothesised to undergo subsequent ligand exchange with PhOSiMe_3_ or S_N_Ar with another molecule of pentafluoropyridine. Both possibilities were therefore investigated computationally. As shown in Figure 3, **INT8** can undergo ligand metathesis with an additional molecule of PhOSiMe_3_ through the formation of a silicate intermediate.

This process begins with a silane‐mediated fluoride abstraction, forming the fluorosilicate anion [PhOSiMe_3_F]^−^ and the corresponding cationic Sb(III) intermediate (**INT9**), as a contacted ion‐pair. This step is endergonic by +25.1 kcal mol^−1^ and proceeds through **TS8**, with an overall Gibbs activation energy for the fluoride abstraction step of 25.7 kcal mol^−1^ (**TS8**, Δ*G* = +12.6 kcal mol–^1^). Following fluoride abstraction, **INT9** (Δ*G* = +12.0 kcal mol^−1^) rapidly evolves to the Sb(III)–OPh phenoxy species (**INT7,** Δ*G* = –16.1 kcal mol^−1^) in a barrierless process (**TS9**), similarly to the pathway described in Figure 1. This transmetallation step closes the catalytic cycle and supports Sb(III)–(OR)_2_ species as the active catalyst of this transformation.

The second possibility involves **INT8** engaging in a second S_N_Ar‐type reaction with pentafluoropyridine through a higher‐energy transition state (**TS10**, Δ*G* = +15.7 kcal mol–^1^), with an overall activation barrier of 28.8 kcal mol^−1^. Although this pathway is experimentally feasible, as shown in Scheme 2D, it is less likely to operate under catalytic conditions as the energy difference between both competing pathways (ΔΔ*G* ^‡^_TS10–TS8_ = 3.1 kcal mol^−1^) favours ligand metathesis over the second S_N_Ar process. Overall, these results suggest that **INT8** preferentially undergoes fluoride‐assisted ligand exchange with PhOSiMe_3_ to regenerate the active LSb(III)–(OPh)_2_ species **INT7**, rather than reacting with a second molecule of pentafluoropyridine. This preference for ligand metathesis is also consistent with catalytic ^19^F NMR experiments, in which no signals attributable to Sb–F species were observed (Figures S36 and S37; for comparison, Sb–F signals for **15** appear at *δ*_F_ = −110 ppm). Together, these data support a speciation regime dominated by LSb(OR)_2_ species under catalytic conditions, rather than LSbF_2_ or heteroleptic LSbF(OR) resting states.

**SCHEME 3:**
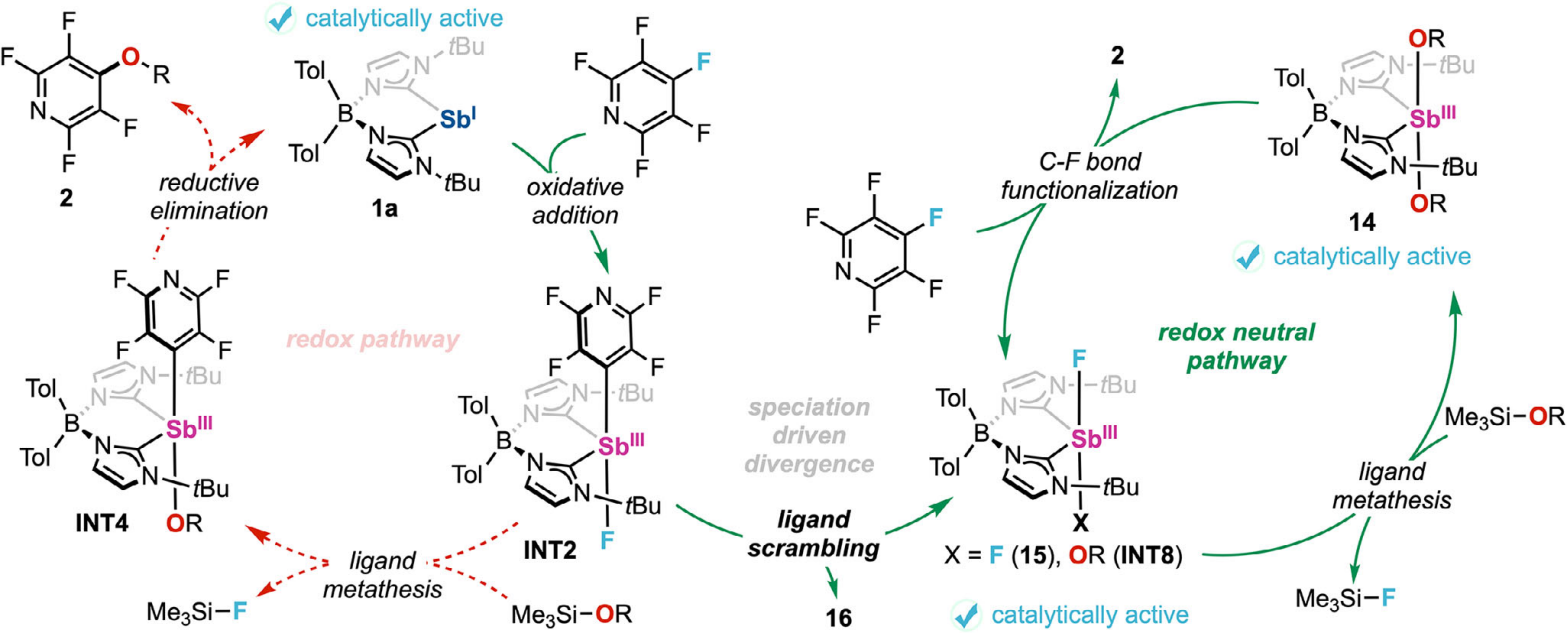
Plausible mechanism for the catalytic defluoroalxokylation of pentafluoropyridine: Sb(I)/Sb(III) redox manifold (non‐operating in C–O bond formation, dashed red arrows) versus Sb(III)–F nucleophilic catalysis (green arrows).

Taken all together, these results support the mechanistic proposal outlined in Scheme 3, in which catalyst speciation plays a decisive role. The reaction is initiated by thermodynamically favourable oxidative addition of stibinidene **1a** into the C–F bond at the 4‐position of pentafluoropyridine, affording the Sb(III) intermediate **INT2**. However, rather than progressing along a classical Sb(I)/Sb(III) turnover manifold through ligand metathesis (**INT4**) and reductive elimination, **INT2** undergoes intermolecular ligand scrambling to generate the Sb(III) difluoride **15** and the bis(4‐tetrafluoropyridyl) complex **16** (see Scheme 2C). DFT calculations indicate that this speciation event diverts the system toward a redox‐neutral manifold with a lower overall Gibbs activation barrier than the formal redox pathway. In addition, although reductive elimination from **16** could, in principle, regenerate stibinidene **1a** and close a redox cycle, this step is strongly disfavoured in benzene (Δ*G* ^‡^ = +36.6 kcal mol^−1^; Figure S37), consistent with stoichiometric observations (Scheme 2B). In contrast, the barrier decreases in polar media such as acetonitrile (Δ*G* ^‡^ = +22.6 kcal mol^−1^, Figure S38), rationalizing the solvent‐dependent homodimerization behaviour. These findings indicate that the operative catalytic regime is dictated not simply by redox accessibility, but by the thermodynamic landscape governing catalyst speciation. The Sb(III) difluoride **15** formed through scrambling is not an off‐cycle byproduct but a turnover‐relevant species. It undergoes ligand metathesis with trimethyl(p‐tolyloxy)silane to furnish the dialkoxide **14**, which mediates C–F functionalization through an S_N_Ar‐type pathway, regenerating **15** and closing a redox‐neutral catalytic cycle. Stibinidene **1a** therefore functions as a precatalyst that enables initial C–F activation but reorganizes under catalytic conditions into the active Sb(III)–F manifold.

## 3 Conclusion

In summary, we have developed a catalytic defluorinative functionalization platform based on heavier group 15 elements (Sb and Bi), enabling the formation of diverse C–heteroatom bonds (C–O, C–S, C–N, C–P, C–Se) from perfluoro(hetero)arenes under mild conditions. Combined DFT and stoichiometric studies demonstrate that catalysis does not proceed through a classical Sb(I)/Sb(III) redox turnover manifold. Instead, oxidative addition of **1a** is followed by ligand scrambling, generating the Sb(III) difluoride **15**. This speciation event constitutes a mechanistic branch point in which a formally redox‐active pnictinidene platform reorganizes into a redox‐neutral catalytic manifold that is thermodynamically and kinetically preferred under the operative conditions. The resulting Sb(III)–F complexes promote C–F functionalization through nucleophilic substitution pathways rather than metallomimetic reductive elimination, establishing ligand scrambling as a productive transformation that unlocks a distinct catalytic regime. More broadly, these findings identify catalyst speciation as a governing parameter in main‐group redox chemistry. A formally accessible Sb(I)/Sb(III) cycle is superseded by a lower‐energy neutral pathway selected through ligand exchange processes intrinsic to heavier pnictogens. Notably, such a speciation‐driven, redox‐neutral pathway may also be operative in previously reported pnictogen‐catalyzed C–Heteroatom bond‐forming reactions that have been interpreted through metallomimetic redox cycles,[67] but where the role of ligand redistribution has not been explicitly considered. In this context, control over ligand dynamics emerges as a design principle for next‐generation *p*‐block catalysts, where the operative catalytic manifold is dictated not solely by oxidation state but by the thermodynamic landscape of accessible species. Strategies to modulate or redirect such speciation processes may enable alternative pnictogen‐based redox or redox‐neutral cycles, thereby expanding the reactivity landscape of main‐group catalysis and strengthening its role as a sustainable alternative to transition‐metal systems in bond activation and functionalization.

## Author Contributions

**Irene Sánchez‐Sordo**: formal analysis, writing – review and editing, investigation, methodology, data curation. **Sergio Fernandez**: writing – review and editing, formal analysis, investigation, conceptualization, methodology, data curation, visualization. **Selwin Fernando**: investigation. **Diego M. Andrada**: supervision, validation, writing – review and editing, funding acquisition, conceptualization, formal analysis. **Oriol Planas**: supervision, writing – original draft, project administration, validation, funding acquisition, conceptualization, visualization.

## Conflicts of Interest

The authors declare no conflicts of interest.

## Supporting information




**Supporting File**: anie73688‐sup‐0001‐SuppMat.pdf.

## Data Availability

The data that support the findings of this study are available in the Supporting Information of this article.
