# Supplementary material for: Speciation‐Controlled C–F Bond Functionalization Enabled by Zwitterionic Pnictinidenes
Source: Angew Chem Int Ed Engl. 2026 Jul 21;65(39):e6579940. doi: 10.1002/anie.6579940 (PMC13592294; doi:10.1002/anie.6579940)
Supplement: Supplementary file 1 [file ANIE-65-e6579940-s001.pdf]

Supporting Information

Speciation-Controlled C–F Bond Functionalization Enabled by Zwitterionic Pnictinidenes

Irene Sánchez-Sordo,[a] Sergio Fernandez,[a*] Selwin Fernando,[a,b] Diego M. Andrada,[c,*] Oriol Planas[a*]

[a]Department of Chemistry, Molecular Sciences Research Hub, Imperial College London, 82 Wood Lane, Shepherds Bush, London, W12 0BZ, UK.

[b]School of Physical and Chemical Sciences, Department of Chemistry, Queen Mary University of London, Mile End Road, London, E1 4NS, UK.

[c]Department of Chemistry, Faculty of Natural Sciences and Technology, Saarland University, Saarbrücken, Germany

*Corresponding authors:

Dr Sergio Fernandez, s.fernandezgonzalez@imperial.ac.uk

Dr Diego M. Andrada, diego.andrada@uni-saarland.de

Dr Oriol Planas, o.planas@imperial.ac.uk

Table of Contents

1 General methods 4

2 Starting materials. Preparation of Silyl nucleophiles 5

3 Catalytic defluorinative functionalization reaction 6

3.1 General methods 6

3.2 Optimization of Sb-catalyzed defluoroalkoxylation of pentafluoropyridine 7

3.3 Results and spectroscopic data for defluoroalkoxylation products 8

3.4 Optimization of the Sb-catalyzed defluorothiolation of pentafluoropyridine 15

3.5 Results and spectroscopic data for defluorothiolation products 15

3.6 Results and spectroscopic data for defluoroamination products 24

3.7 Results and spectroscopic data for defluorophosphonylation products 27

3.8 Results and spectroscopic data for defluoroselenation products 29

3.9 Results and spectroscopic data for defluoroarylation products 29

4 Blank reactions 31

5 Mechanistic studies 32

5.1 Radical trap tests for defluoroalkoxylation reaction 32

5.2 Radical trap tests for defluorothiolation reaction 33

5.3 Attempts of reductive elimination of C-X bonds from **11** 34

5.4 Attempts on the isolation of species **16** 46

5.5 Stoichiometric experiments and synthesis of Sb(III) relevant species 52

5.6 Catalytic experiments 65

6 Computational details 71

6.1 Plausible mechanisms for defluorinative alkoxylation of pentafluoropyridine 71

6.2 Sb-catalyzed homodimerization of pentafluoropyridine 73

6.3 Sb-catalyzed defluorosilylation of pentafluoropyridine 76

6.4 Mechanism A 78

6.5 Mechanism B (Redox-neutral S_NAr without ligand scrambling) 102

6.6 Mechanism C (Redox-neutral S_NAr after ligand scrambling) 111

6.7 Mechanism D (OA to Si-O) 124

6.8 Dimerization mechanism in C_6H_6 128

6.9 Dimerization mechanism in CH_3CN 139

6.10 Defluorosilylation mechanism 160

6.11 Plausible mechanisms for defluorinative alkoxylation of pentafluoropyridine employing bismuthinidene 3 167

6.12 Bi-catalyzed homodimerization of pentafluoropyridine 172

6.13 Mechanism A (Bismuth) 175

6.14 Mechanism B (Redox-neutral S_NAr without ligand scrambling) (Bismuth) 195

6.15 Mechanism C (Redox-neutral S_NAr after ligand scrambling) 204
6.16 Dimerization mechanism in C_6H_6 (Bismuth) 220
6.17 Dimerization mechanism in CH_3CN (Bismuth) 228
7 NMR Characterization data 245
7.1 NMR characterization of defluoroalkoxylation products...................................... 245
7.2 NMR characterization of defluorothiolation products... 271
7.3 NMR characterization of defluoroamination products... 304
7.4 NMR characterization of defluorophosphonylation products 310
7.5 NMR characterization of defluoroselenation products.. 326
7.6 NMR characterization of defluoroarylation products... 329
7.7 NMR characterization of Sb(III) relevant species 14 and 15............................... 331
8 References.. 346

1 General methods

Unless otherwise stated, all manipulations were performed using standard Schlenk techniques under dry nitrogen in oven-dried glassware or in N_2-filled MBraun Unilab SP glovebox. Celite was dried at 150 °C under vacuum for 3 days and stored in the glovebox prior to use. Anhydrous solvents were collected from a MBraun SPS-800 solvent purification system under N_2 and stored over activated 4Å MS. Deuterated solvents were stored over activated 4Å MS under N_2 prior to use. Fluorinated (hetero)arenes were distilled and stored over activated 4Å molecular sieves prior to use. All the other reagents utilized in this work were obtained from Sigma-Aldrich and used without further purification. Zwitterionic stibinidine complexes **1a, 1b**, perfluoropyridylstibane complex **11** and bismuthinidene complex **3**,[1] *N,C,N*-stibinidine complex **5,**[2] and trialkylsilyl nucleophiles (see Section 2) were prepared according to reported procedures. For catalytic defluorinative functionalization (DFF) processes, α,α,α-trifluorotoluene was distilled under vacuum and stored over activated 4Å MS prior to be used as internal standard.

NMR spectra were recorded using 400 MHz Bruker Avance III. ^{1}H NMR spectra (400 MHz) were referenced to the residual protons of the corresponding deuterated solvent:[6] $CDCl_3$ (δ = 7.26 ppm), C_6D_6 (δ = 7.16 ppm) or CD_3CN (δ = 1.94 ppm). ^{13}C{^{1}H} NMR spectra (101 MHz) were referenced internally to the D-coupled ^{13}C resonances of the corresponding deuterated solvent:[6] $CDCl_3$ (δ = 77.2 ppm), C_6D_6 (δ = 128.1 ppm) or CD_3CN (δ = 118.3 ppm). Chemical shifts (*δ*) are given in ppm, relative to deuterated solvent residual peak, and coupling constants (*J*) provided in Hz. NMR data are represented according to the following structure: chemical shift, multiplicity (s = singlet, d = doublet, dd = doublet of doublet, t = triplet, q = quartet, quint = quintuplet, hept = heptuplet, m = multiplet, bs = broad singlet), coupling constants (Hz), integration and assigned atom (labelled according to the depicted structure).

HRMS data were only acquired for compounds that had not been previously reported in the bibliography. Atmospheric Solid Probe Analysis coupled with Capillary Heated Electrospray Ionization (ASAP-HESI) was used to acquire mass spectra at the University College London (UCL) Chemistry Mass Spectrometry Facility. The generated gas phase ions were measured using the Exactive Plus mass spectrometer. HESI parameters were the auxiliary gas to 5 and the sheath and sweep gases to 0 and the auxiliary gas heater to 400 ºC and typical operating temperatures were between 100 °C and 450 °C but higher temperatures are needed for some compounds and lower more appropriate for others. The discharge voltage was set to between 3.5 and 4.0 kV to give a stable background ion signal would depend on the structure of analyte.

2 Starting materials. Preparation of Silyl nucleophiles

Silyl nucleophiles **S1-S6** employed in this work are depicted in Figure S1. Compounds **S2f**, **S3a**, **S3c** and **S5** were purchased from commercial suppliers and used without further purification. Compounds **S1a-h**,[3] **S1a'**,[4] **S1a''**,[5] **2a-e**,[6] **S3b**,[7] **S4a-b**,[8] and **S6**[9] were prepared and purified according to reported general procedures.

The following notation will be used throughout the Supporting Information to avoid confusion with the nomenclature employed in the main manuscript.

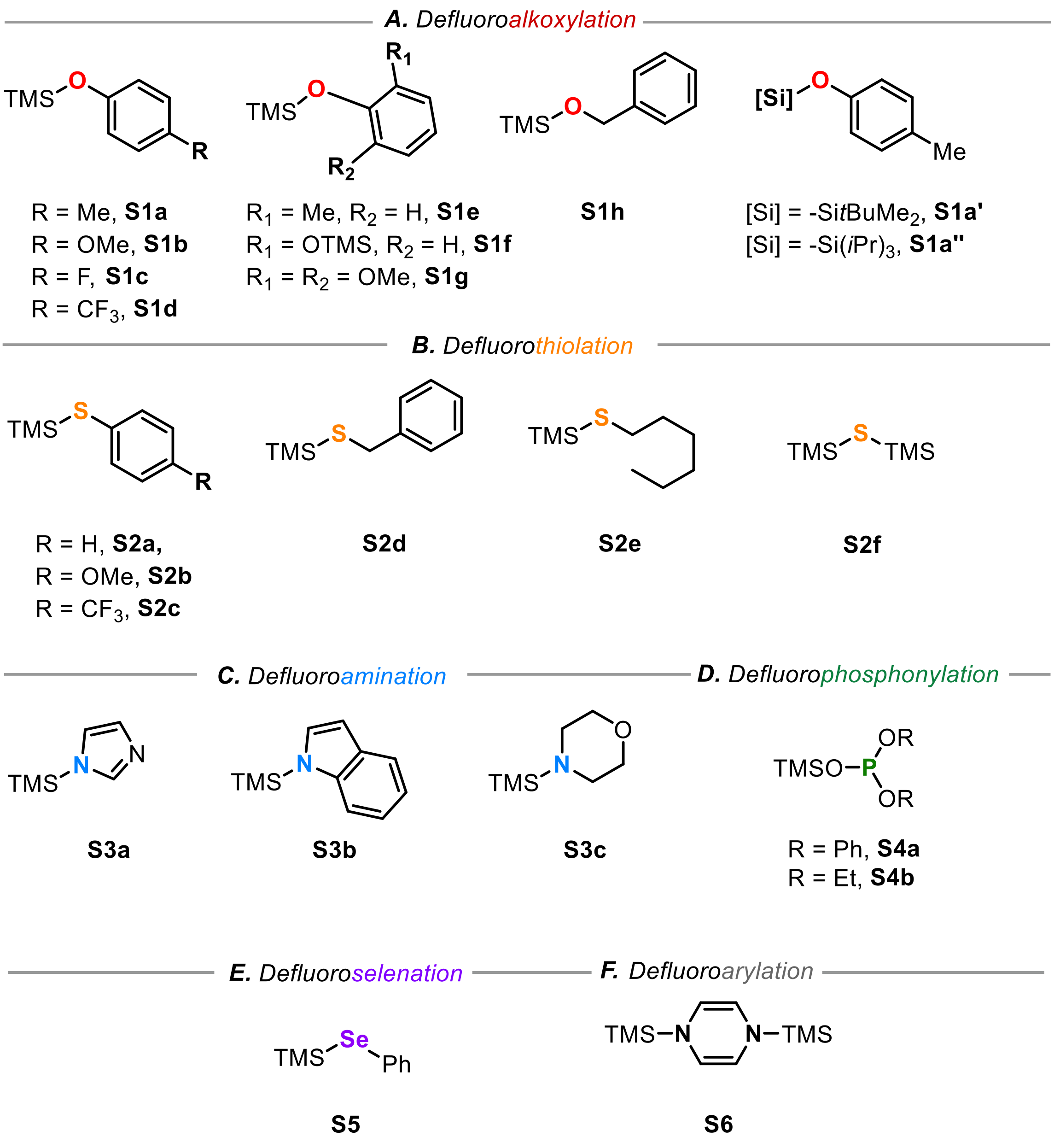

Figure S1. Trialkylsilyl nucleophiles **S1-S6** employed in this work.

3 Catalytic defluorinative functionalization reaction

3.1 General methods

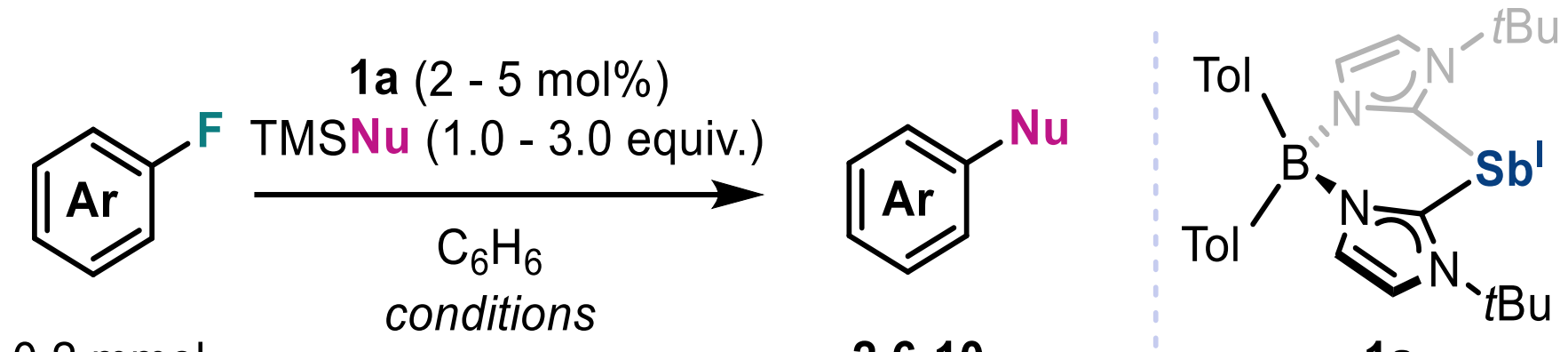

0.2 mmol **2,6-10** **1a**

To a solution of the corresponding perfluoro(hetero)arene (0.20 mmol) and the corresponding trimethylsilyl nucleophile **S1-5** (1.0 to 3.0 equiv.) in benzene (0.25 mL) was added a solution of the Sb(I) complex **1a** (0.004 mmol, 2 mol%, unless otherwise stated) in benzene (0.25 mL) as catalyst. The mixture was then stirred at rt or 60 ºC for 1, 2 or 16h. After this, α,α,α -trifluorotoluene (0.10 mmol) was added to the mixture as internal standard and the reaction was monitored by ^{19}F NMR. The functionalized products were isolated via flash column chromatography using SiO_2 as stationary phase and a suitable solvent mixture as eluent or via direct filtration through a SiO_2 plug.

For defluoroarylation reactions, 0.50 equivalents of Mashima's reagent **S6** were used as reductant and CH_3CN was used as solvent.

3.2 Optimization of Sb-catalyzed defluoroalkoxylation of pentafluoropyridine

Table S1. Table showing the optimization of the catalytic defluoroalkoxylation of pentafluoropyridine.

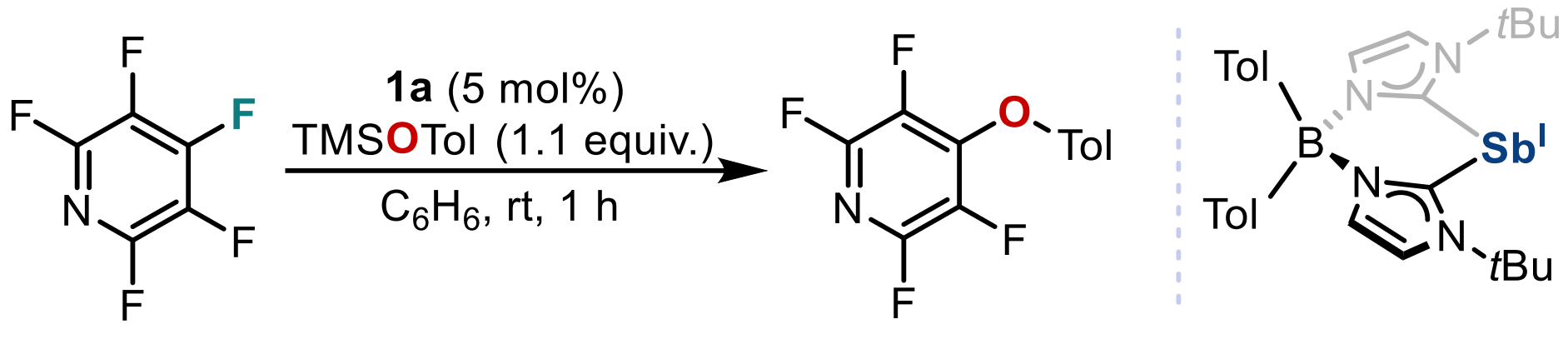

Entry	Deviation from standard conditions	Yield of 2a (%)[a]
1	none	>95
2	No **1a**	0
3	0.5 mol% **1a**	>95
4	1 mol% **1a**	>95
5	2 mol% **1a**	>95
6	**1b** instead of **1a**	>95
7	**Bi(I)** (**3**) instead of **1a**	>95
8	**Sb(III)** (**4**) instead of **1a**	0
9	***N,C,N*-Sb(I)**[2] (**5**) instead of **1a**	<5
10	**TASF** instead of **1a**	0
11	**THF** as solvent	86
12	**Et_2O** as solvent	75
13	**CH_3CN** as solvent	76
14	**Hexane** as solvent	81
15	**TBSOTol S1a'** instead of TMSOTol	10
16	**TIPSOTol S1a''** instead of TMSOTol	>95
17	**SbF_3** instead of **1a**	0
18	**$Sb(OTol)_3$** instead of **1a**	0

[a]Calculated by ^{19}F NMR using α,α,α-trifluorotoluene (0.50 equiv.) as IS.

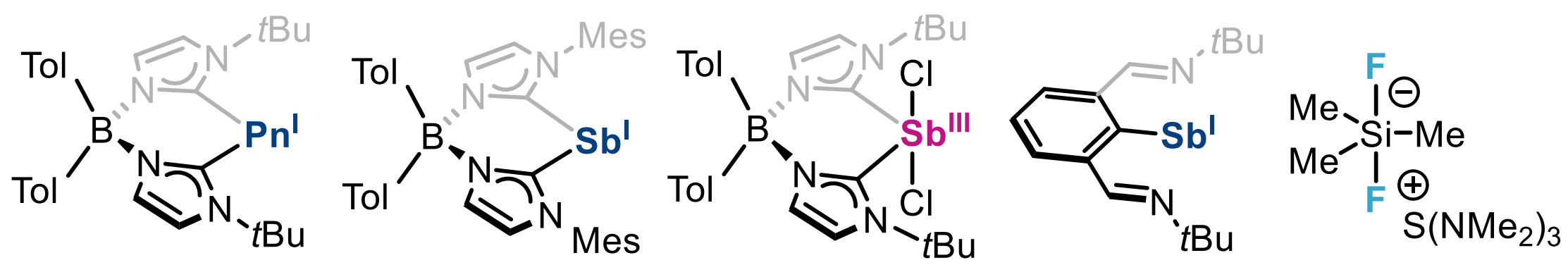

Pn = Sb (**1a**), Bi (**3**) **1b** **4** **5** **TASF**

3.3 Results and spectroscopic data for defluoroalkoxylation products

3.3.1 2,3,5,6-tetrafluoro-4-(*p*-tolyloxy)pyridine (**2a**)

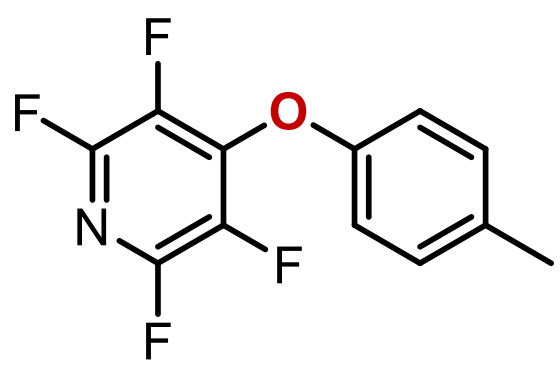

Using the general method with 1.0 equiv. of silyl ether **S1a** and pentafluoropyridine resulted in the formation of **2a** at >95% NMR yield after 1 h at rt. The same result was obtained at multigram scale using 0.5 mol% of stibinidene complex **1a**. The product was isolated as a white solid (1.23 g, 96% yield) using flash column chromatography (Hexane:Ethyl acetate, 10:1).

Spectroscopic data are in accordance with previously reported values.[10]

^{1}H NMR (400 MHz, $CDCl_3$) δ = 7.20 (d, $J_{H\text{-}H}$ = 8.6 Hz, 2H), 6.98 (d, $J_{H\text{-}H}$ = 8.6 Hz, 2H), 2.38 (s, 3H).

^{13}C{^{1}H} NMR (101 MHz, $CDCl_3$) δ = 153.8 (C), 145.6 – 142.8 (m, 2 x C), 145.1 – 144.6 (m, C), 137.9 – 134.5 (m, 2 x C), 134.9 (C), 130.4 (2 x CH), 116.6 (2 x CH), 20.7 (CH_3).

^{19}F{^{1}H} NMR (377 MHz, $CDCl_3$) δ = -84.48 – -94.55 (m, 2F), -150.99 – -157.89 (m, 2F).

3.3.2 2,3,5,6-tetrafluoro-4-(4-methoxyphenoxy)pyridine (**2b**)

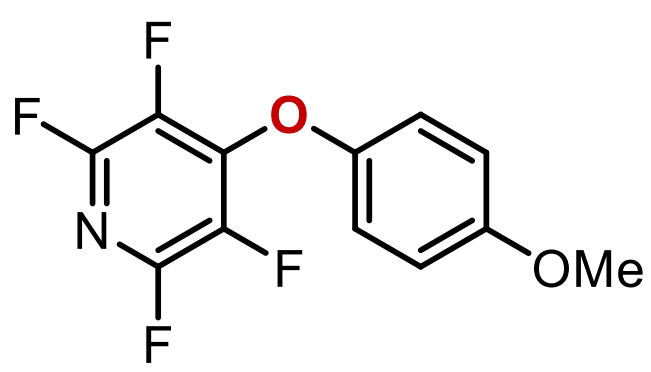

Using the general method with 1.0 equiv. of silyl ether **S1b** and pentafluoropyridine resulted in the formation of **2b** at 78% NMR yield after 2 h at rt. The product was isolated as a colourless oil (38 mg, 75% yield) using flash column chromatography (Hexane:Ethyl acetate, 10:1).

Spectroscopic data are in accordance with previously reported values.[11]

^{1}H NMR (400 MHz, $CDCl_3$) δ = 7.05 (d, $J_{H\text{-}H}$ = 9.1 Hz, 2H), 6.91 (d, $J_{H\text{-}H}$ = 9.2 Hz, 2H), 3.84 (s, 3H).

^{13}C{^{1}H} NMR (101 MHz, $CDCl_3$) δ = 156.9 (C), 149.7 (C), 146.3 – 141.8 (m, 3 x C), 138.9 – 133.3 (m, 2 x C), 118.2 (2 x CH), 114.9 (2 x CH), 55.7 (CH_3).

^{19}F{^{1}H} NMR (377 MHz, $CDCl_3$) δ = -82.02 – -100.87 (m, 2F), -139.74 – -173.57 (m, 2F).

3.3.3 2,3,5,6-tetrafluoro-4-(4-fluorophenoxy)pyridine (**2c**)

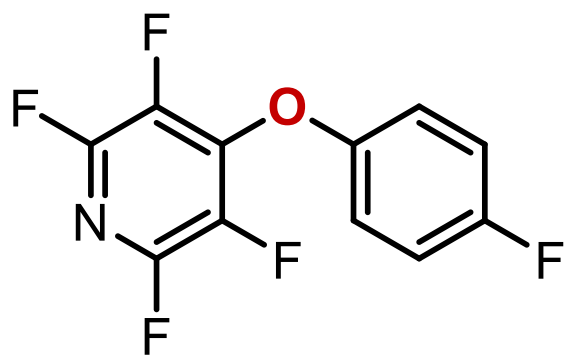

Using the general method with 1.0 equiv. of silyl ether **S1c** and pentafluoropyridine resulted in the formation of **2c** at >95% NMR yield after 1 h at rt. The product was isolated as a white solid (50 mg, 96% yield) using flash column chromatography (Hexane to Hexane:Ethyl acetate, 20:1).

Spectroscopic data are in accordance with previously reported values.[12]

^{1}H NMR (400 MHz, CDCl$_3$) δ = δ 7.26 – 6.28 (m, 4H).

^{13}C{^{1}H} NMR (101 MHz, CDCl$_3$) δ = 159.7 (d, $J_{C\text{-}F}$ = 244.6 Hz, C), 151.7 (C), 146.5 – 142.2 (m, 3 x C), 139.6 – 133.7 (m, 2 x C), 118.5 (d, $J_{C\text{-}F}$ = 8.5 Hz, 2 x CH), 116.7 (d, $J_{C\text{-}F}$ = 24.0 Hz, 2 x CH).

^{19}F{^{1}H} NMR (377 MHz, CDCl$_3$) δ = -88.23 – -88.59 (m, 2F), -117.19 (s, 1F), -154.43 – -154.70 (m, 2F).

3.3.4 2,3,5,6-tetrafluoro-4-(4-(trifluoromethyl)phenoxy)pyridine (**2d**)

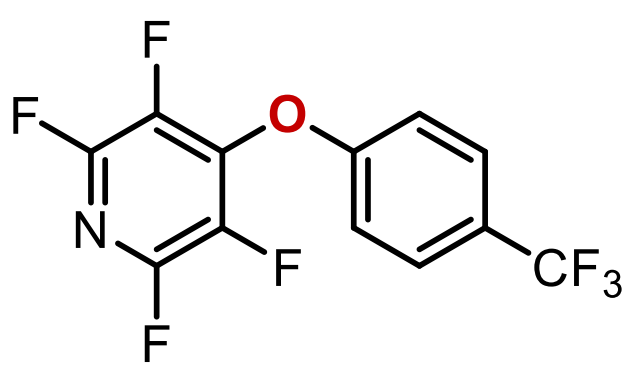

Using the general method with 1.0 equiv. of silyl ether **S1d**, pentafluoropyridine and 5 mol% of stibinidene complex **1a** resulted in the formation of **2d** at 61% NMR yield after 16 h at 60 ºC. The product was isolated as a colourless oil (35 mg, 56% yield) using flash column chromatography (Hexane:Dichloromethane, 10:1).

Spectroscopic data are in accordance with previously reported values.[13]

^{1}H NMR (400 MHz, CDCl$_3$) δ = 7.67 (d, $J_{H\text{-}H}$ = 8.3 Hz, 2H), 7.15 (d, $J_{H\text{-}H}$ = 8.3 Hz, 2H).

^{13}C{^{1}H} NMR (101 MHz, CDCl$_3$) δ = 158.0 (C), 127.8 (q, $J_{C\text{-}F}$ = 3.9 Hz, 2 x CH), 123.6 (q, $J_{C\text{-}F}$ = 233.0 Hz, C), 116.8 (2 x CH). C atoms directly attached to fluorine atoms could not be observed due to low intensity and broadening due to coupling pattern with the F nuclei.

^{19}F{^{1}H} NMR (377 MHz, CDCl$_3$) δ = -62.15 (s, 3F), -87.27 – -87.81 (m, 2F), -153.28 – -153.76 (m, 2F).

3.3.5 2,3,5,6-tetrafluoro-4-(*o*-tolyloxy)pyridine (**2e**)

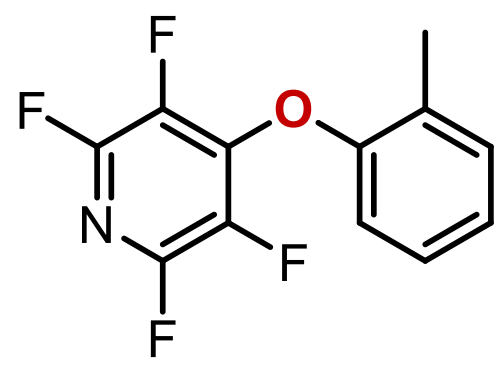

Using the general method with 1.0 equiv. of silyl ether **S1e** and pentafluoropyridine resulted in the formation of **2e** at >95% NMR yield after 1 h at rt. The product was isolated as a colourless oil (49 mg, 96% yield) using flash column chromatography (Hexane:Ethyl acetate, 10:1).

Spectroscopic data are in accordance with previously reported values.[13]

^{1}H NMR (400 MHz, $CDCl_3$) δ = 7.33 – 7.29 (m, 1H), 7.25 – 6.98 (m, 2H), 6.84 (dd, $J_{H\text{-}H}$ = 8.0, 1.5 Hz, 1H), 2.40 (s, 3H).

^{13}C{^{1}H} NMR (101 MHz, $CDCl_3$) δ = 154.3 (C), 146.7 – 141.8 (m, 3 x C), 137.5 – 134.25 (m, 2 x C), 131.8 (CH), 128.0 (C), 127.2 (CH), 125.3 (CH), 115.8 (CH), 15.8 (CH_3).

^{19}F{^{1}H} NMR (377 MHz, $CDCl_3$) δ = -83.97 – -91.90 (m, 2F), -153.08 – -159.33 (m, 2F).

3.3.6 2-((perfluoropyridin-4-yl)oxy)phenol (**2f**)

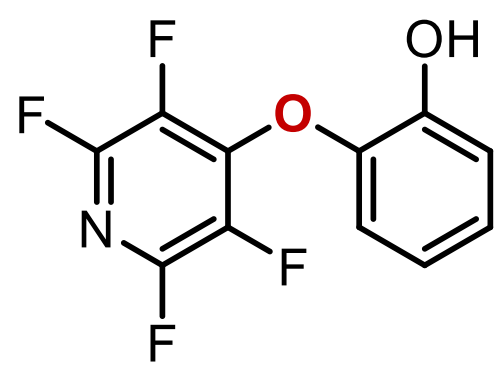

Using the general method with 1.0 equiv. of silyl ether **S1f** and pentafluoropyridine resulted in the formation of **2f-OTMS** at 90% NMR yield after 16 h at 60 ºC. However, the TMS group was lost during the purification step, and the product **2f** was isolated as a colourless oil (45 mg, 87% yield) using flash column chromatography (Hexane to Hexane:Ethyl acetate, 10:1).

^{1}H NMR (400 MHz, $CDCl_3$) δ = 7.33 – 7.20 (m, 2H), 7.17 – 6.99 (m, 2H).

^{13}C{^{1}H} NMR (101 MHz, $CDCl_3$) δ = 146.5 – 142.9 (m, 2 x C), 145.5 (2 x C), 144.3 – 144.2 (m, C), 137.2 – 134.3 (m, 2 x C), 126.7 (2 x CH), 119.0 (2 x CH).

^{19}F{^{1}H} NMR (377 MHz, $CDCl_3$) δ = -85.29 – -90.11 (m, 2F), -153.26 – -158.95 (m, 2F).

HRMS (ESI-pos, m/z): calc'd for $C_{11}H_4O_2NF_4$ $[M-H]^-$ 258.0184; found 258.0183.

3.3.7 4-(2,6-dimethoxyphenoxy)-2,3,5,6-tetrafluoropyridine (**2g**)

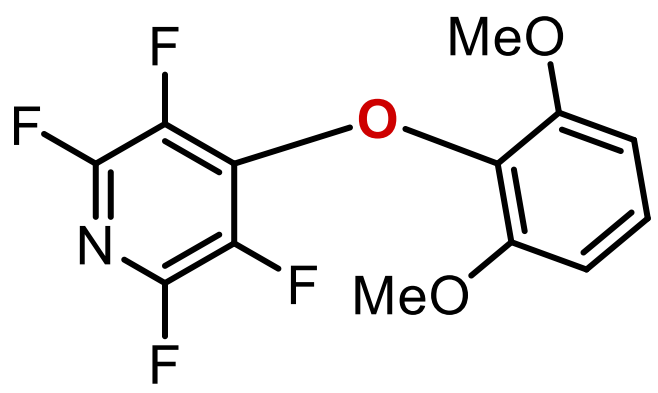

Using the general method with 1.0 equiv. of silyl ether **S1g** and pentafluoropyridine resulted in the formation of **2g** at >95% NMR yield after 1 h at rt. The product was isolated as a white solid (58 mg, 96% yield) using flash column chromatography (Hexane:Ethyl acetate, 10:1).

^{1}H NMR (400 MHz, $CDCl_3$) δ = 7.15 (t, J_{H-H} = 8.5 Hz, 1H), 6.62 (d, J_{H-H} = 8.5 Hz, 2H), 3.82 (s, 6H).

$^{13}C\{^1H\}$ NMR (101 MHz, $CDCl_3$) δ = 152.0 (3 x C), 147.0 - 146.8 (m, C), 146.0 – 142.0 (m, 2 x C), 136.7 – 133.3 (m, 2 x C), 126.5 (CH), 105.0 (2 x CH), 56.4 (2 x CH_3).

$^{19}F\{^1H\}$ NMR (377 MHz, $CDCl_3$) δ = -84.47 – -97.01 (m, 2F), -139.76 – -182.98 (m, 2F).

HRMS (ESI-pos, m/z): calc'd for $C_{13}H_{10}O_3NF_4$ $[M+H]^+$ 304.05913; found 304.05484.

3.3.8 4-(benzyloxy)-2,3,5,6-tetrafluoropyridine (**2h**)

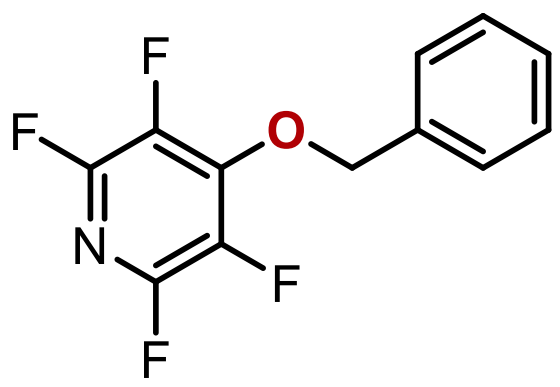

Using the general method with 1.0 equiv. of silyl ether **S1h** and pentafluoropyridine resulted in the formation of **2h** at 61% NMR yield after 16 h at 60 °C. The product was isolated as a colourless oil (30 mg, 59% yield) using flash column chromatography (Hexane to Hexane:Ethyl acetate, 20:1).

^{1}H NMR (400 MHz, $CDCl_3$) δ = 7.46 – 7.37 (m, 5H), 5.51 (s, 2H).

$^{13}C\{^1H\}$ NMR (101 MHz, $CDCl_3$) δ = 134.7 (C), 129.5 (CH), 129.1 (2 x CH), 128.3 (2 x CH), 75.9 (t, J_{C-F} = 4.5 Hz, CH_2). C atoms directly attached to fluorine atoms could not be observed due to low intensity and broadening due to coupling pattern with the F nuclei.

$^{19}F\{^1H\}$ NMR (377 MHz, $CDCl_3$) δ = -90.24 – -90.62 (m, 2F), -157.45 – -157.83 (m, 2F).

HRMS (ESI-pos, m/z): calc'd for $C_{18}H_8ONF_4$ $[M-H]^-$ 258.0537; found 258.0539.

3.3.9 2,3,5-trifluoro-4,6-bis(*p*-tolyloxy)pyridine (**2a'**)

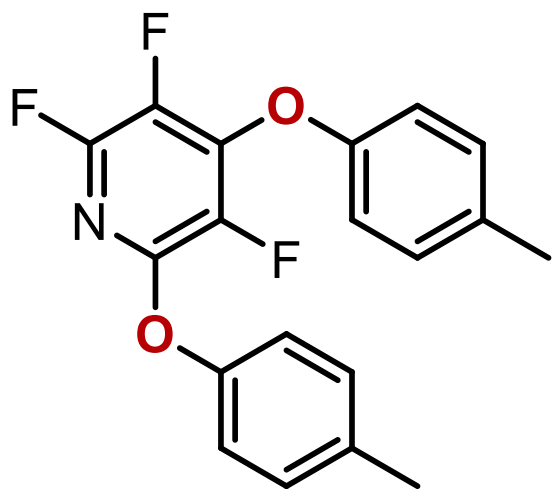

Using the general method with 3.0 equiv. of silyl ether **S1a**, pentafluoropyridine and 5 mol% of stibinidene complex **1a** resulted in the formation of **2a'** at >95% NMR yield after 2 h at rt. The product was isolated as a white solid (67 mg, 97% yield) using flash column chromatography (Hexane:Ethyl acetate, 10:1).

^{1}H NMR (400 MHz, CDCl$_3$) δ = 7.22 (d, J_{H-H} = 8.2 Hz, 2H), 7.18 (d, J_{H-H} = 8.2 Hz, 2H), 7.06 (d, J_{H-H} = 8.5 Hz, 2H), 6.99 (d, J_{H-H} = 8.6 Hz, 2H), 2.38 (s, 3H), 2.36 (s, 3H).

^{13}C{^{1}H} NMR (101 MHz, CDCl$_3$) δ = 154.3 (C), 150.6 (C), 146.2 – 143.1 (m, 3 x C), 138.2 (dd, J_{C-F} = 260.4, 8.1 Hz, C), 135.3 (C), 134.4 (C), 134.3 (dd, J_{C-F} = 259.2, 30.3 Hz, C), 130.5 (2 x CH), 130.3 (2 x CH), 120.8 (2 x CH), 116.5 (2 x CH), 21.0 (CH_3), 20.8 (CH_3).

^{19}F{^{1}H} NMR (377 MHz, CDCl$_3$) δ = -89.49 (dd, J_{F-F} = 26.7, 22.4 Hz, 1F), -153.39 (d, J_{F-F} = 26.3 Hz, 1F), -159.65 (d, J_{F-F} = 22.4 Hz, 1F).

HRMS (ESI-pos, m/z): calc'd for $C_{19}H_{15}O_2NF_3$ $[M+H]^+$ 346.1049; found 346.1050.

3.3.10 1,2,4,5-tetrafluoro-3-(*p*-tolyloxy)-6-(trifluoromethyl)benzene (**2i**)

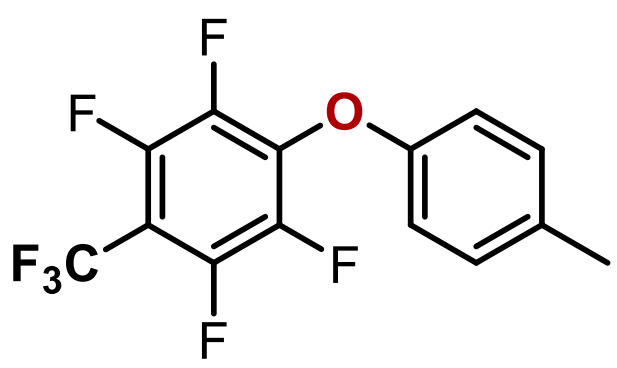

Using the general method with 1.0 equiv. of silyl ether **S1a** and octafluorotoluene resulted in the formation of **2i** at 90% NMR yield after 1 h at rt. The product was isolated as a white solid (59 mg, 87% yield) using flash column chromatography (Hexane).

Spectroscopic data are in accordance with previously reported values.[10]

^{1}H NMR (400 MHz, CDCl$_3$) δ = 7.15 (d, J_{H-H} = 8.2 Hz, 2H), 6.90 (d, J_{H-H} = 8.6 Hz, 2H), 2.34 (s, 3H).

^{13}C{^{1}H} NMR (101 MHz, CDCl$_3$) δ = 154.7 (C), 134.4 (2 x CH), 130.5 (2 x CH), 116.0 (C), 20.8 (CH_3). C atoms directly attached to fluorine atoms could not be observed due to low intensity and broadening due to coupling pattern with the F nuclei.

^{19}F{^{1}H} NMR (377 MHz, CDCl$_3$) δ = -55.83 (t, J_{F-F} = 21.8 Hz, 3F), -140.37 – -140.74 (m, 2F), -151.77 – -152.60 (m, 2F).

3.3.11 Methyl 2,3,5,6-tetrafluoro-4-(*p*-tolyloxy)benzoate (**2j**)

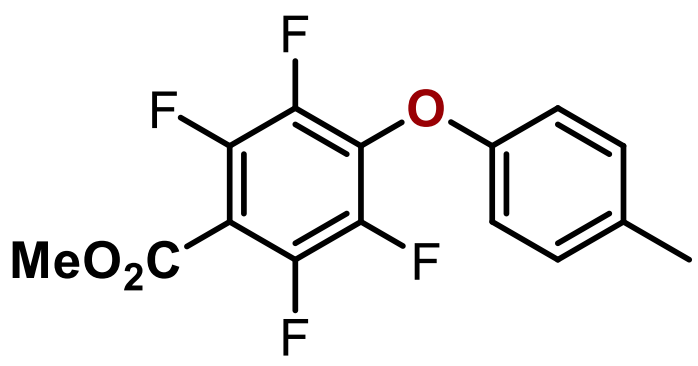

Using the general method with 1.0 equiv. silyl ether **S1a** and methyl pentafluorobenzoate resulted in the formation of **2j** at 94% NMR yield after 1 h at rt. The product was isolated as a colourless oil (57 mg, 91% yield) using flash column chromatography (Hexane:Ethyl acetate, 10:1).

^{1}H NMR (400 MHz, $CDCl_3$) δ = 7.16 (d, J_{H-H} = 8.2 Hz, 2H), 6.91 (d, J_{H-H} = 8.6 Hz, 2H), 4.02 (s, 3H), 2.35 (s, 3H).

^{13}C{^{1}H} NMR (101 MHz, $CDCl_3$) δ = 160.3 (C), 155.0 (C), 147.3 – 140.2 (m, 6 x C), 134.1 (C), 130.5 (2 x CH), 115.9 (2 x CH), 108.5 – 108.1 (m, C) 53.5 (CH_3), 20.9 (CH_3).

^{19}F{^{1}H} NMR (377 MHz, $CDCl_3$) δ = -128.38 – -145.36 (m, 2F), -149.69 – -159.75 (m, 2F).

HRMS (ESI-pos, m/z): calc'd for $C_{15}H_{11}O_3F_4$ $[M+H]^+$ 304.0638; found 315.0637.

3.3.12 2,3,5,6-tetrafluoro-4-(*p*-tolyloxy)benzonitrile (**2k**)

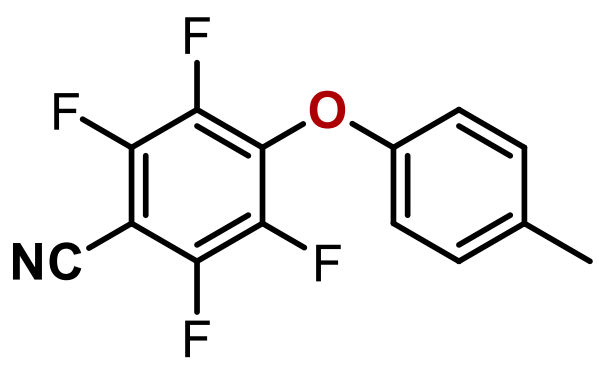

Using the general method with 1.0 equiv. of silyl ether **S1a** and pentafluorobenzonitrile resulted in the formation of **2k** at 75% NMR yield after 2 h at 60 ºC. The product was isolated as a colourless oil (40 mg, 71% yield) using flash column chromatography (Hexane:Ethyl acetate, 10:1).

Spectroscopic data are in accordance with previously reported ones.[10]

^{1}H NMR (400 MHz, $CDCl_3$) δ = 7.16 (d, J_{H-H} = 8.1 Hz, 1H), 6.90 (d, J_{H-H} = 8.8 Hz, 2H), 2.34 (s, 2H).

^{13}C{^{1}H} NMR (101 MHz, $CDCl_3$) δ = 154.4 (C), 134.7 (C), 130.5 (2 x CH), 128.3 (C), 116.0 (2 x CH), 20.7 (CH_3). C atoms directly attached to fluorine atoms could not be observed due to low intensity and broadening due to coupling pattern with the F nuclei.

^{19}F{^{1}H} NMR (377 MHz, $CDCl_3$) δ = -132.11 – -132.36 (m, 2F), -150.46 – -151.02 (m, 2F).

3.3.13 Unsuccessful substrates for defluorinative alkoxylation

The following substrates were obtained in less than 10% yield after 16 h at 60 ºC employing 5 mol% of stibinidine **1a**:

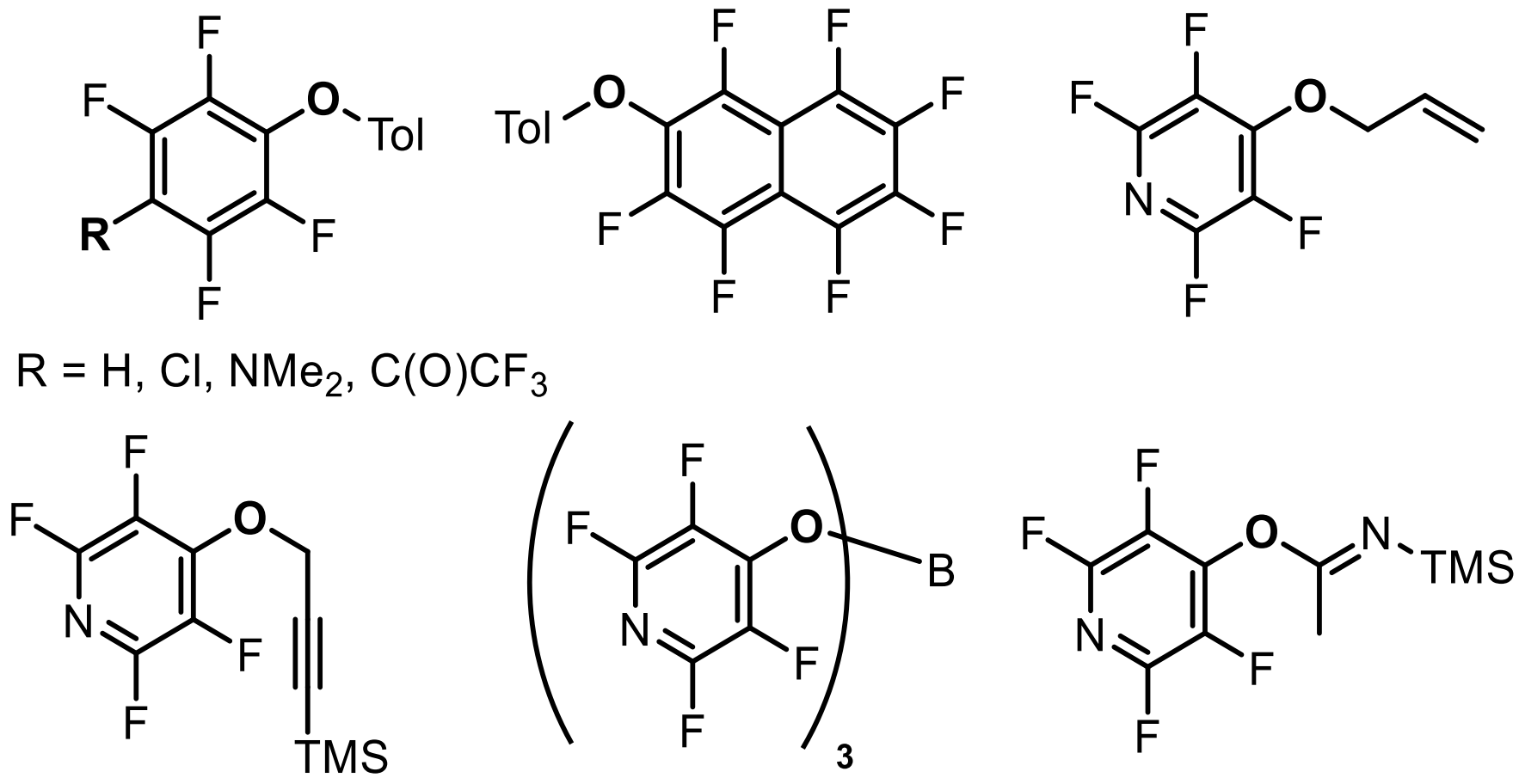

3.4 Optimization of the Sb-catalyzed defluorothiolation of pentafluoropyridine

Table S2. Table showing the optimization of the catalytic defluorothiolation of pentafluoropyridine.

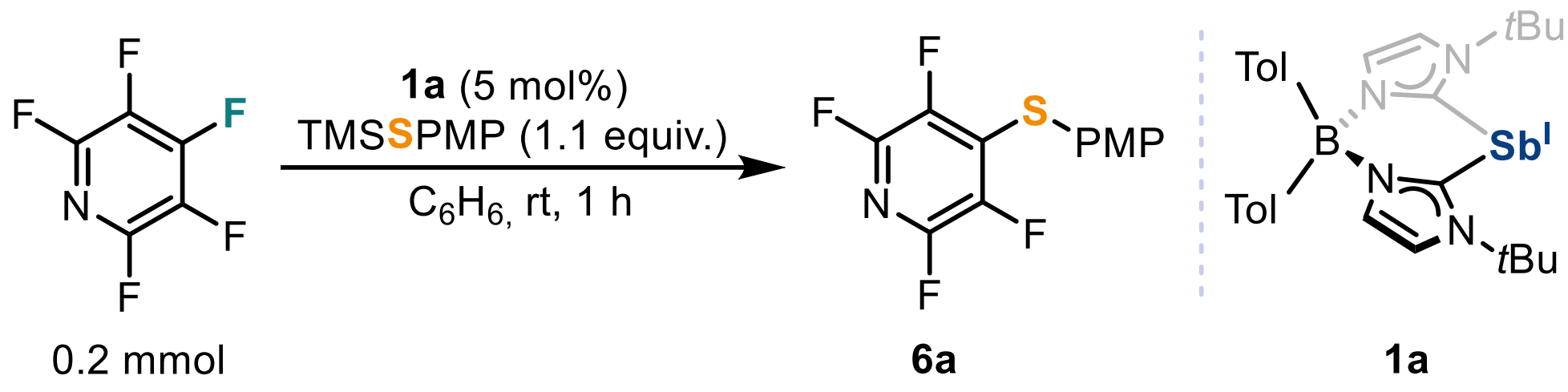

Entry	Deviation from standard conditions	Yield of 6a (%)[a]
1	None	>95
2	No **1a**	0
3	0.5 mol% **1a**	>95
4	1 mol% **1a**	>95
5	2 mol% **1a**	>95
6	**Sb(III)** (**4**) instead of **1a**	0
7	***N,C,N*-Sb(I)**[2] (**5**) instead of **1a**	>95%
8	**THF** as solvent	>95
9	**Et_2O** as solvent	>95
10	**CH_3CN** as solvent	>95
11	**Hexane** as solvent	>95

[a]Calculated by ^{19}F NMR using α,α,α-trifluorotoluene (0.50 equiv.) as IS.

3.5 Results and spectroscopic data for defluorothiolation products

3.5.1 2,3,5,6-tetrafluoro-4-(phenylthio)pyridine (**6a**)

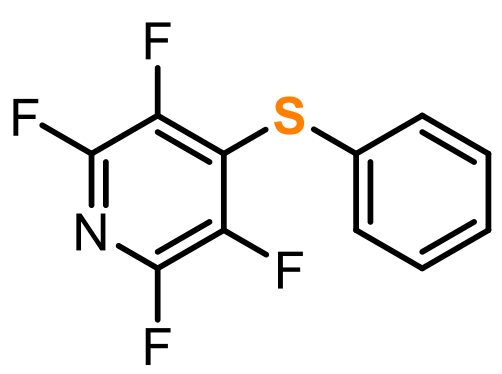

Using the general method with 1.0 equiv. of silyl thioether **S2a** and pentafluoropyridine resulted in the formation of **6a** at >95% NMR yield after 1 h at rt. The product was isolated as a colourless oil (48 mg, 92% yield) using flash column chromatography (Hexane:Ethyl acetate, 10:1).

Spectroscopic data are in accordance with previously reported values.[14]

^{1}H NMR (400 MHz, $CDCl_3$) δ = 7.60 – 7.47 (m, 2H), 7.45 – 7.34 (m, 3H).

^{13}C{^{1}H} NMR (101 MHz, $CDCl_3$) δ = 145.4 – 139.0 (m, 5 x C), 132.9 (2 x CH), 129.7 (2 x CH), 129.5 (CH).

^{19}F{^{1}H} NMR (377 MHz, $CDCl_3$) δ = -90.30 – -90.62 (m, 2F), -136.28 – -136.65 (m, 2F).

3.5.2 2,3,5,6-tetrafluoro-4-((4-methoxyphenyl)thio)pyridine (**6b**)

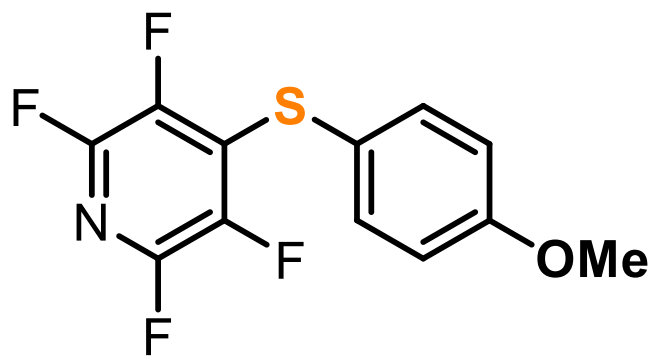

Using the general method with 1.0 equiv. of silyl thioether **S2b** and pentafluoropyridine resulted in the formation of **6b** at >95% NMR yield after 1 h at rt. The product was isolated as a colourless oil (54 mg, 93% yield) using flash column chromatography (Hexane:Ethyl acetate, 10:1).

Spectroscopic data are in accordance with previously reported values.[11]

^{1}H NMR (400 MHz, CDCl$_3$) δ = 7.52 (d, $J_{H\text{-}H}$ = 9.0 Hz, 2H), 6.89 (d, $J_{H\text{-}H}$ = 9.0 Hz, 2H), 3.83 (s, 3H).

^{13}C{^{1}H} NMR (101 MHz, CDCl$_3$) δ = 161.1 (C), 136.0 (C), 118.7 (2 x CH), 115.2 (2 x CH), 55.5 (CH$_3$). C atoms directly attached to fluorine atoms could not be observed due to low intensity and broadening due to coupling pattern with the F nuclei.

^{19}F{^{1}H} NMR (377 MHz, CDCl$_3$) δ = -83.90 – -105.20 (m, 2F), -137.70 – -138.19 (m, 2F).

3.5.3 2,3,5,6-tetrafluoro-4-((4-(trifluoromethyl)phenyl)thio)pyridine (**6c**)

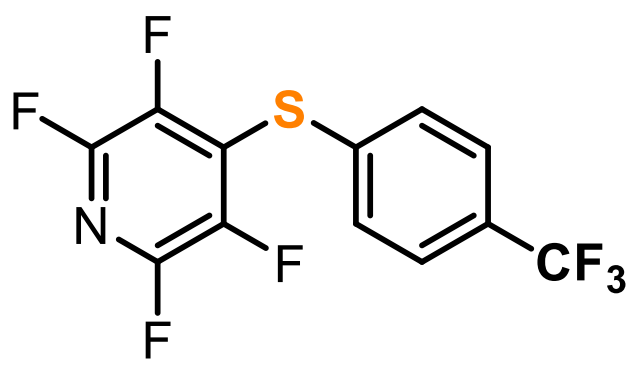

Using the general method with 1.0 equiv. of silyl thioether **S2c** and pentafluoropyridine resulted in the formation of **5c** at >95% NMR yield after 1 h at rt. The product was isolated as a colourless oil (59 mg, 90% yield) using flash column chromatography (Hexane:Dichloromethane, 10:1, R_f = 0.23).

^{1}H NMR (400 MHz, CDCl$_3$) δ = 7.62 (d, $J_{H\text{-}H}$ = 8.4 Hz, 2H), 7.57 (d, $J_{H\text{-}H}$ = 8.6 Hz, 2H).

^{13}C{^{1}H} NMR (101 MHz, CDCl$_3$) δ = 145.8 – 139.7 (m, 5 x C), 134.6 (C), 132.0 (2 x CH), 131.4 (q, $J_{C\text{-}F}$ = 32.8 Hz, C), 126.7 (q, $J_{C\text{-}F}$ = 3.7 Hz, 2 x CH), 123.7 (q, $J_{C\text{-}F}$ = 272.9 Hz, C).

^{19}F{^{1}H} NMR (377 MHz, CDCl$_3$) δ = -62.97 (s, 3F), -89.26 – -89.48 (m, 2F), -135.04 – -135.26 (m, 2F).

HRMS (EI-pos, m/z): calc'd for $C_{12}H_4F_7NS$ $[M\text{-}H]^-$ 326.9900; found 326.9901.

3.5.4 2,5-difluoro-3,4,6-tris((4-methoxyphenyl)thio)pyridine (**6a”**)

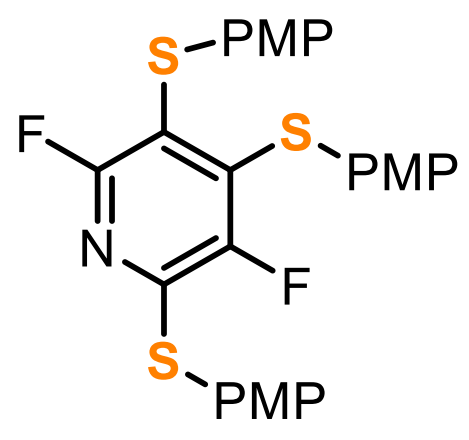

Using the general method with 3.0 equiv. of silyl thioether **S2b**, pentafluoropyridine and 5 mol% of stibinidene complex **1a** resulted in the formation of **6a”** at >95% NMR yield after 2 h at rt. The product was isolated as a white solid (98 mg, 92% yield) using flash column chromatography (Hexane to Hexane:Ethyl acetate, 5:2).

^{1}H NMR (400 MHz, $CDCl_3$) δ = 7.42 (d, J_{H-H} = 8.9 Hz, 2H), 7.40 (d, J_{H-H} = 8.6 Hz, 2H), 7.34 (d, J_{H-H} = 8.8 Hz, 2H), 6.92 (d, J_{H-H} = 8.8 Hz, 2H), 6.85 (d, J_{H-H} = 8.8 Hz, 2H), 6.81 (d, J_{H-H} = 8.8 Hz, 2H), 3.82 (s, 3H), 3.81 (s, 3H), 3.77 (s, 3H).

^{13}C{^{1}H} NMR (101 MHz, $CDCl_3$) δ = 160.9 (C), 160.3 (C), 159.5 (C), 158.2 (dd, J_{C-F}= 237.9, 4.6 Hz, C), 151.5 (dd, J_{C-F} = 254.6, 4.6 Hz, C), 146.8 (dd, J_{C-F} = 23.4, 15.3 Hz, C), 142.9 (dd, J_{C-F} = 13.4, 2.6 Hz, C), 137.4 (2 x CH), 134.9 (2 x CH), 133.1 (2 x CH), 124.8 (C), 121.9 (d, J_{C-F} = 4.1 Hz, C), 117.3 (d, J_{C-F} = 2.4 Hz, C), 114.9 (2 x CH), 114.8 (2 x CH), 114.8 (2 x CH), 55.5 (2 x CH_3), 55.4 (CH_3).

^{19}F{^{1}H} NMR (377 MHz, $CDCl_3$) δ = -64.12 (d, J_{F-F} = 27.3 Hz, 1F), -118.96 (d, J_{F-F} = 27.7 Hz, 1F).

HRMS (ESI-pos, m/z): calc’d for $C_{26}H_{21}F_2NO_3S_3$ $[M-H]^+$ 530.0724; found 530.0729.

3.5.5 4-(benzylthio)-2,3,5,6-tetrafluoropyridine (**6d**)

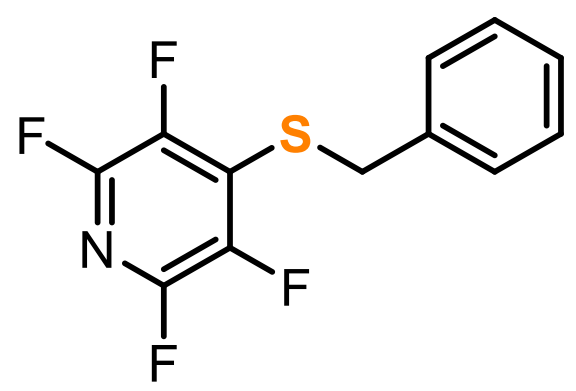

Using the general method with 1.0 equiv. silyl thioether **S2d** and pentafluoropyridine resulted in the formation of **6d** at >95% NMR yield after 1 h at rt. The product was isolated as a colourless oil (52 mg, 96% yield) using flash column chromatography (Hexane to Hexane:Ethyl acetate, 20:1).

Spectroscopic data are in accordance with previously reported values.[15]

^{1}H NMR (400 MHz, $CDCl_3$) δ = 7.34 – 7.27 (m, 5H), 4.37 (s, 2H).

^{13}C{^{1}H} NMR (101 MHz, $CDCl_3$) δ = 144.9 – 139.1 (m, 5 x C), 135.3 (C), 128.9 (2 x CH), 128.9 (2 x CH), 128.3 (CH), 37.5 (t, J_{C-F} = 4.9 Hz, CH_2).

^{19}F{^{1}H} NMR (377 MHz, $CDCl_3$) δ = -91.08 – -91.34 (m, 2F), -137.01 – -137.33 (m, 2F).

3.5.6 2,3,5,6-tetrafluoro-4-(hexylthio)pyridine (**6e**)

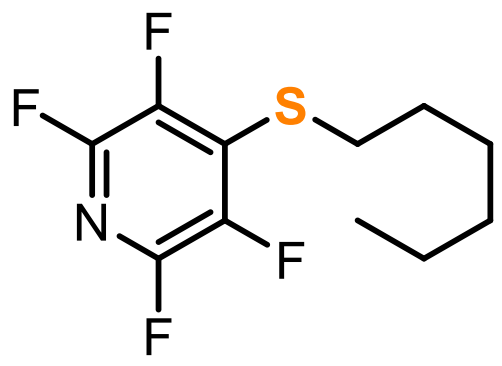

Using the general method with 1.0 equiv. of silyl thioether **S2e** and pentafluoropyridine resulted in the formation of **6e** at 93% NMR yield after 1 h at rt. The product was isolated as a colourless oil (49 mg, 88% yield) using flash column chromatography (Hexane).

Spectroscopic data are in accordance with previously reported values.[16]

^{1}H NMR (400 MHz, $CDCl_3$) δ = 3.17 (t, J_{H-H} = 7.4 Hz, 2H), 1.73 – 1.59 (m, 2H), 1.50 – 1.38 (m, 2H), 1.36 – 1.24 (m, 4H), 0.88 (d, J_{H-H} = 7.0 Hz, 3H).

^{13}C{^{1}H} NMR (101 MHz, $CDCl_3$) δ = 145.0– 131.9 (m, 5 x C), 33.4 (t, J_{C-F} = 4.9 Hz, CH_2), 31.4 (CH_2), 30.0 (CH_2), 28.3 (CH_2), 22.6 (CH_2), 14.1 (CH_3).

^{19}F{^{1}H} NMR (377 MHz, $CDCl_3$) δ = -91.60 – -91.82 (m, 2F), -138.60 – -138.84 (m, 2F).

3.5.7 (4-chloro-2,3,5,6-tetrafluorophenyl)(4-methoxyphenyl)sulfane (**6f**)

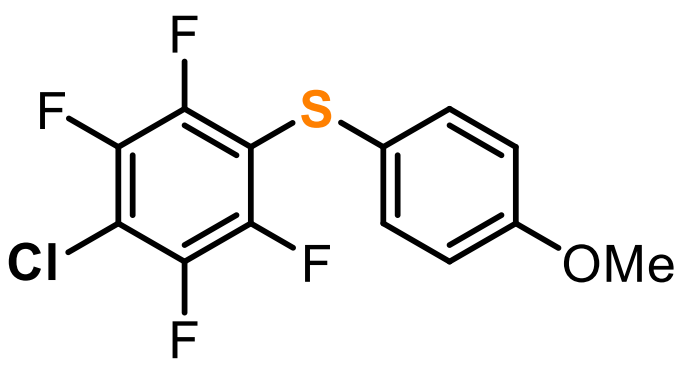

Using the general method with 1.0 equiv. of silyl thioether **S2b** and chloropentafluorobenzene resulted in the formation of **6f** at 61% NMR yield after 16 h at rt. The product was isolated as a colourless oil (36 mg 57% yield) using flash column chromatography (Hexane to Hexane: Ethyl acetate, 10:1).

^{1}H NMR (400 MHz, $CDCl_3$) δ = 7.45 (d, J_{H-H} = 8.8 Hz, 2H), 6.84 (d, J_{H-H} = 8.8 Hz, 2H), 3.79 (s, 3H).

^{13}C{^{1}H} NMR (101 MHz, $CDCl_3$) δ = 160.4 (C), 135.0 (2 x CH), 122.6 (C), 115.1 (2 x CH), 55.5 (CH_3). C atoms directly attached to fluorine atoms could not be observed due to low intensity and broadening due to coupling pattern with the F nuclei.

^{19}F{^{1}H} NMR (377 MHz, $CDCl_3$) δ = -132.47 – -132.73 (m, 2F), -136.58 – -150.97 (m, 2F).

HRMS (ESI-pos, m/z): calc'd for $C_{13}H_7OClF_4S$ $[M]^+$ 321.9837; found 321.9830.

3.5.8 Methyl 2,3,5,6-tetrafluoro-4-((4-methoxyphenyl)thio)benzoate (**6g**)

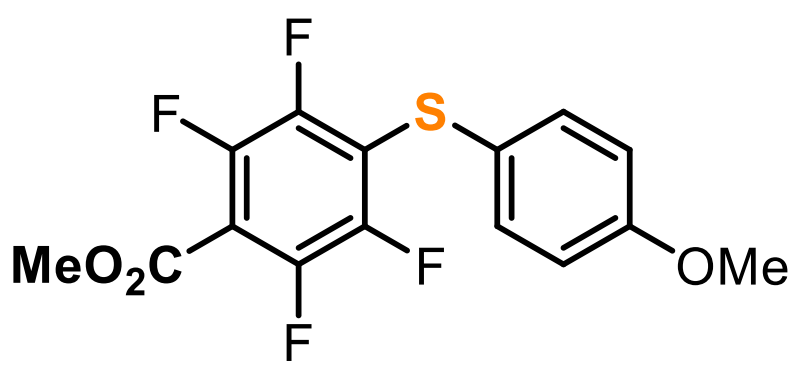

Using the general method with 1.0 equiv. of silyl thioether **S2b** and methyl pentafluorobenzoate resulted in the formation of **6g** at >95% NMR yield after 1 h at rt. The product was isolated as a colourless oil (64 mg, 93% yield) using flash column chromatography (Hexane:Ethyl acetate, 10:1).

^{1}H NMR (400 MHz, $CDCl_3$) δ = 7.46 (d, $J_{H\text{-}H}$ = 8.7 Hz, 2H), 6.84 (d, $J_{H\text{-}H}$ = 8.7 Hz, 2H), 3.96 (s, 3H), 3.80 (s, 3H).

^{13}C{^{1}H} NMR (101 MHz, $CDCl_3$) δ = 160.6 (C), 160.1 (C), 148.3 – 145.1 (m, 2 x C), 146.5 – 143.2 (m, 2 x C), 135.2 (2 x CH), 121.6 (C), 120.3 (t, $J_{C\text{-}F}$ = 19.4 Hz, C), 115.1 (2 x CH), 112.1 (t, $J_{C\text{-}F}$ = 16.1 Hz, C), 55.5 (CH_3), 53.4 (CH_3).

^{19}F{^{1}H} NMR (377 MHz, $CDCl_3$) δ = -130.45 – -136.34 (m, 2F), -137.63 – -141.76 (m, 2F).

HRMS (ESI-pos, m/z): calc'd for $C_{15}H_{11}O_3F_4S$ $[M+H]^+$ 347.0359; found 347.0354.

3.5.9 2,3,5,6-tetrafluoro-4-((4-methoxyphenyl)thio)benzonitrile (**6h**)

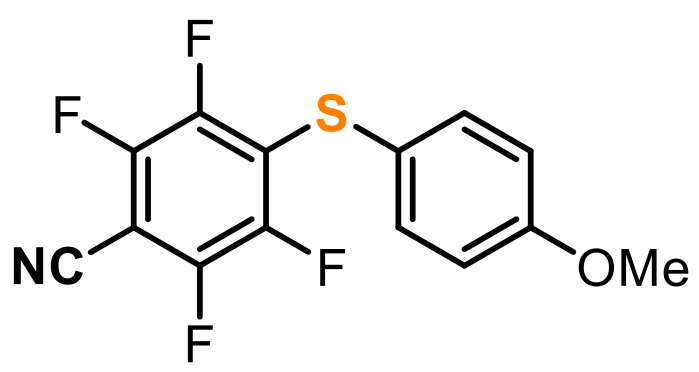

Using the general method with 1.0 equiv. of silyl thioether **S2b** and pentafluorobenzonitrile resulted in the formation of **6h** at 90% NMR yield after 1 h at rt. The product was isolated as a colourless oil (55 mg, 87% yield) using flash column chromatography (Hexane:Ethyl acetate, 10:1).

^{1}H NMR (400 MHz, $CDCl_3$) δ = 7.51 (d, $J_{H\text{-}H}$ = 8.8 Hz, 1H), 6.89 (d, $J_{H\text{-}H}$ = 8.8 Hz, 1H), 3.84 (s, 2H).

^{13}C{^{1}H} NMR (101 MHz, $CDCl_3$) δ = 161.1 (C), 147.5 – 146.6 (m, 2 x C), 148.6 – 144.6 (m, 2 x C), 135.8 (2 x CH), 135.6 (C), 125.5 – 124.9 (m, C), 120.0 (C), 115.3 (2 x CH), 107.9 – 107.3 (m, C), 55.6 (CH_3).

^{19}F{^{1}H} NMR (377 MHz, $CDCl_3$) δ = -130.59 – -131.74 (m, 2F), -131.79 – -132.79 (m, 2F).

HRMS (ESI-pos, m/z): calc'd for $C_{14}H_8ONF_4S$ $[M+H]^+$ 314.0253; found 314.0254.

3.5.10 (4-methoxyphenyl)(2,3,5,6-tetrafluoro-4-(trifluoromethyl)phenyl)sulfane (**6i**)

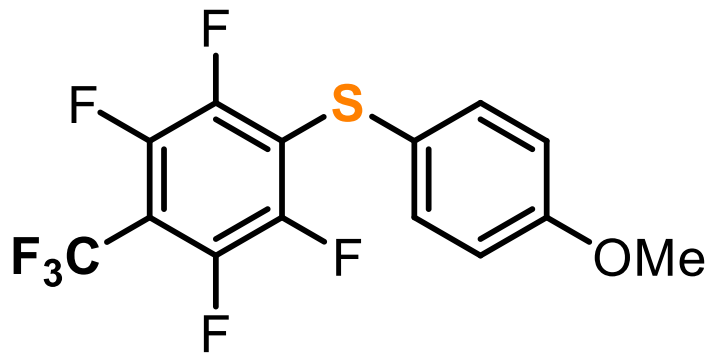

Using the general method with 1.0 equiv. of silyl thioether **S2b** and octafluorotoluene resulted in the formation of **6i** at 90% NMR yield after 1 h at rt. The product was isolated as a colourless oil (62 mg, 87% yield) using flash column chromatography (Hexane).

^{1}H NMR (400 MHz, CDCl$_3$) δ = 7.49 (d, $J_{H\text{-}H}$ = 8.9 Hz, 2H), 6.86 (d, $J_{H\text{-}H}$ = 8.9 Hz, 2H), 3.81 (s, 3H).

^{13}C{^{1}H} NMR (101 MHz, CDCl$_3$) δ = 160.9 (C), 149.7 – 141.1 (m, 4 x C), 135.6 (2 x CH), 122.2 (C), 120.9 (C), 120.8 (q, $J_{C\text{-}F}$ = 253.3 Hz, C), 115.2 (2 x CH), 55.5 (CH$_3$). One C atom could not be observed due to low intensity and a strong coupling with the F nuclei.

^{19}F{^{1}H} NMR (377 MHz, CDCl$_3$) δ = -56.24 (d, $J_{F\text{-}F}$ = 21.8 Hz, 3F), -130.27 – -136.59 (m, 2F), -138.47 – -144.09 (m, 2F).

HRMS (ESI-pos, m/z): calc'd for $C_{14}H_7OF_7S$ [M]$^+$ 356.0100; found 356.0093.

3.5.11 2,2,2-trifluoro-1-(2,3,5,6-tetrafluoro-4-((4-methoxyphenyl)thio)phenyl)ethan-1-one (**6j**)

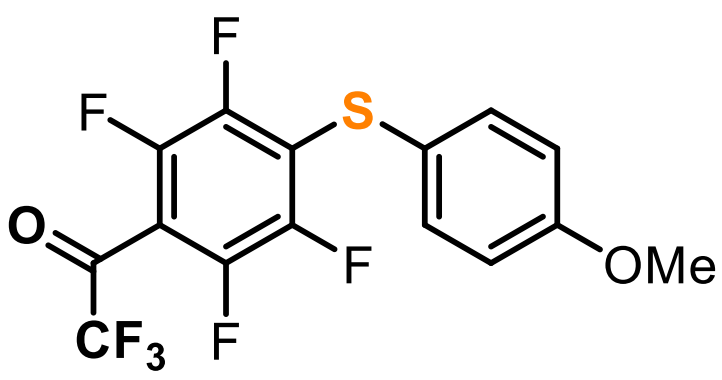

Using the general method with 1.0 equiv. of silyl thioether **S2b** and perfluoroacetophenone resulted in the formation of **6j** at >95% NMR yield after 2 h at 60ºC. The product was isolated as a colourless oil (73 mg, 93% yield) using flash column chromatography (Hexane:Ethyl acetate, 10:1 to 3:1).

^{1}H NMR (400 MHz, CDCl$_3$) δ = 7.51 (d, $J_{H\text{-}H}$ = 8.8 Hz, 2H), 6.88 (d, $J_{H\text{-}H}$ = 8.9 Hz, 2H), 3.82 (s, 3H).

^{13}C{^{1}H} NMR (101 MHz, CDCl$_3$) δ = 161.0 (C), 148.1 – 144. 8 (m, 2 x C), 146.5 – 143.1 (m, 2 x C), 135.8 (2 x CH), 124.4 (t, J = 19.2 Hz, C), 120.4 (C), 115.3 (2 x CH), 114.9 (q, J = 290.2 Hz, C), 111.8 (t, J = 16.8 Hz, C), 55.5 (CH$_3$). Carbonyl C atom could not be observed due to low intensity and a strong coupling with the F nuclei.

^{19}F{^{1}H} NMR (377 MHz, CDCl$_3$) δ = -76.97 (t, $J_{F\text{-}F}$ = 10.3 Hz, 3F), -132.05 – -132.25 (m, 2F), -137.98 – -138.22 (m, 2F).

HRMS (ESI-pos, m/z): calc'd for $C_{15}H_7O_2F_7S$ [M]$^+$ 384.0050; found 384.0040.

3.5.12 (Perfluoronaphthalene-2,6-diyl)bis((4-methoxyphenyl)sulfane) (**6k**)

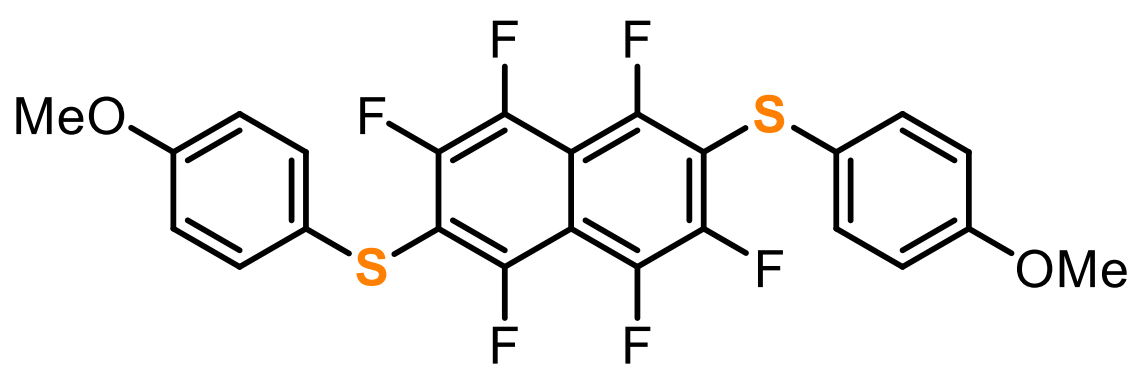

Using the general method with 2.0 equiv. of thiosilyl ether **S2b** and perfluoronaphthalene resulted in the formation of **6k** at 85% NMR yield after 16 h at 60 ºC. The product was isolated as a white solid (84 mg, 82% yield) using flash column chromatography (Hexane to Hexane:Ethyl acetate, 20:1).

^{1}H NMR (400 MHz, CDCl$_3$) δ = 7.46 (d, $J_{H\text{-}H}$ = 8.8 Hz, 4H), 6.82 (d, $J_{H\text{-}H}$ = 8.8 Hz, 4H), 3.78 (s, 6H).

^{13}C{^{1}H} NMR (101 MHz, CDCl$_3$) δ = 160.3 (2 x C), 134.8 (4 x CH), 122.7 (2 x C), 115.1 (4 x CH), 55.5 (2 x CH$_3$). C atoms directly attached to fluorine atoms could not be observed due to low intensity and a strong coupling with the F nuclei.

^{19}F{^{1}H} NMR (377 MHz, CDCl$_3$) δ = -109.30 – -114.09 (m, 2F), -129.31 – -134.68 (m, 2F), -143.13 – -149.14 (m, 2F).

HRMS (ESI-pos, m/z): calc'd for $C_{24}H_{14}O_2F_6S_2$ [M]$^+$ 512.0322; found 512.0339.

3.5.13 (Perfluoro-[1,1'-biphenyl]-4,4'-diyl)bis((4-methoxyphenyl)sulfane) (**6l**)

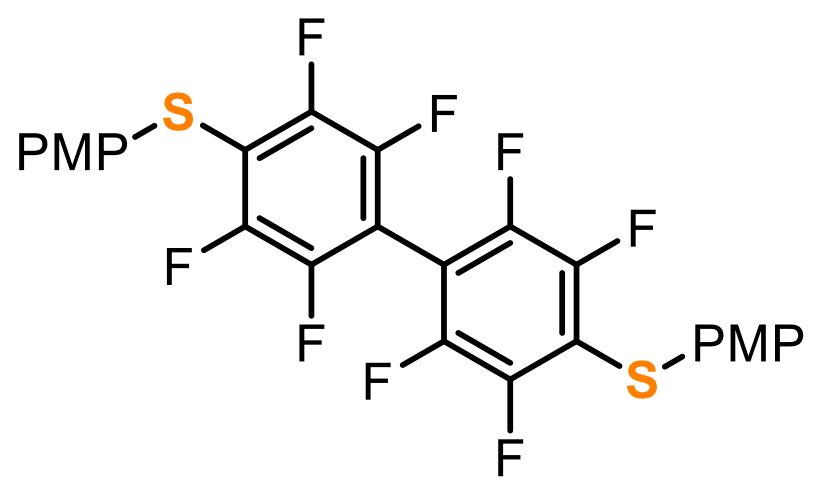

Using the general method with 2.0 equiv. of silyl thioether **S2b** and perfluorobiphenyl resulted in the formation of **6l** at >95% NMR yield after 1 h at rt. The product was isolated as a white solid (107 mg, 93% yield) using flash column chromatography (Hexane:Ethyl acetate, 10:1).

^{1}H NMR (400 MHz, CDCl$_3$) δ = 7.52 (d, $J_{H\text{-}H}$ = 8.8 Hz, 4H), 6.87 (d, $J_{H\text{-}H}$ = 8.8 Hz, 1H), 3.81 (s, 6H).

^{13}C{^{1}H} NMR (101 MHz, CDCl$_3$) δ 160.5 (2 x C), 148.4 – 145.3 (m, 4 x C), 145.3 – 142.2 (m, 4 x C), 135.4 (4 x CH), 122.0 (2 x C), 119.0 – 118.6 (m, 4 x C), 115.0 (4 x CH), 55.5 (2 x CH$_3$).

^{19}F{^{1}H} NMR (377 MHz, CDCl$_3$) δ = -132.48 – -133.75 (m, 4F), -136.94 – -138.23 (m, 4F).

HRMS (ESI-pos, m/z): calc'd for $C_{26}H_{14}O_2F_8S_2$ [M]$^+$ 574.0302; found 574.0292.

3.5.14 Tris(2,3,5,6-tetrafluoro-4-((4-methoxyphenyl)thio)phenyl)phosphane (**6m**)

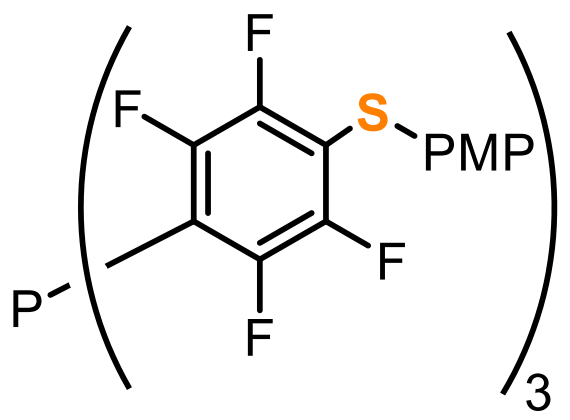

Using the general method with 3.0 equiv. of silyl thioether **S2b** and tris(pentafluorophenyl)phosphine resulted in the formation of **6m** at 52% NMR yield after 2 h at 60 ºC. The product was isolated as a white solid (77 mg, 44% yield) using flash column chromatography (Hexane to Hexane:Ethyl acetate, 10:1).

^{1}H NMR (400 MHz, CDCl$_3$) δ = 7.47 (d, $J_{H\text{-}H}$ = 8.8 Hz, 6H), 6.85 (d, $J_{H\text{-}H}$ = 8.8 Hz, 6H), 3.80 (s, 9H).

^{13}C{^{1}H} NMR (101 MHz, CDCl$_3$) δ = 160.6 (3 x C), 148.8-145.8 (m, 6 x C), 147.6-145.0 (m, 6 x C), 135.2 (6 x CH), 121.7 (3 x C), 115.1 (6 x CH), 55.5 (3 x CH_3). Three C atom could not be observed due to low intensity and broadening due to coupling pattern with the F and P nuclei.

^{19}F{^{1}H} NMR (377 MHz, CDCl$_3$) δ = -129.71 – -130.40 (m, 6F), -132.65 – -133.44 (m, 6F).

^{31}P NMR (162 MHz, CDCl$_3$) δ = -72.55 (hept, $J_{P\text{-}F}$ = 35.2 Hz).

HRMS (ESI-pos, m/z): calc'd for $C_{39}H_{22}F_{12}O_3PS_3$ $[M+H]^+$ 893.0277; found 893.0265.

3.5.15 Bis(perfluoropyridin-4-yl)sulfane (**6n**)

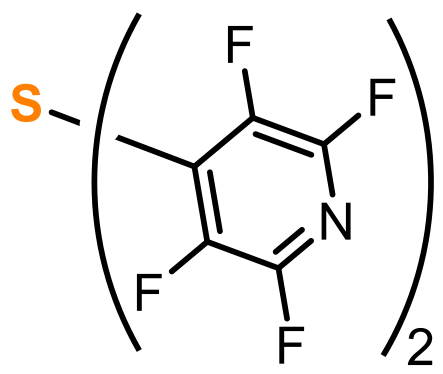

Using the general method with 0.5 equiv. of bistrimethylsilyl sulfide **S2f** and pentafluoropyridine resulted in the formation of **5n** at 38% NMR yield after 16 h at 60 ºC. The product was isolated as an off-white solid (11 mg, 32% yield) after filtration through a silica plug using DCM as eluent.

Spectroscopic ^{19}F NMR data are in accordance with previously reported values.[17]

^{19}F{^{1}H} NMR – (377 MHz, CDCl$_3$) δ = -87.12 – -91.05 (m, 4F), -131.38 – -135.27 (m, 4F).
^{13}C{^{1}H} NMR (101 MHz, CDCl$_3$) δ = C atoms could not be observed due to low intensity and broadening due to coupling pattern with the F nuclei.

3.5.16 *Z/E*-(4-methoxyphenyl)(perfluoroprop-1-en-1-yl)sulfane (**6o**)

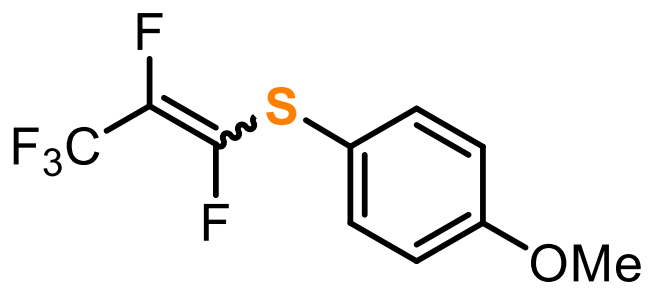

Using the general method with 1.0 equiv. of silyl thioether **S2b** and an excess of hexafluoropropene resulted in the formation of **6o** at >75% NMR yield after 2 h at rt. The product was isolated as a colourless oil (35 mg, 65% yield) using flash column chromatography (Hexane) as a mixture of isomers in a *E:Z* ratio 3.2:1.

^{1}H NMR (400 MHz, $CDCl_3$) δ = 7.75 – 7.38 (m, *Z* + *E* isomer), 7.07 – 6.83 (m, *Z* + *E* isomer), 3.83 (s, 3H, *E* isomer), 3.82 (s, 3H, *Z* isomer)

^{13}C{^{1}H} NMR (101 MHz, $CDCl_3$) δ = 161.4 (*E* isomer, C), 161.3 (*Z* isomer, C), 136.2 (*E* isomer, 2 x CH), 135.5 (*Z* isomer, 2 x CH), 116.8 (2 x C), 115.4 (4 x CH), 55.6 (2 x CH_3). C atoms directly attached to fluorine atoms could not be observed due to low intensity and broadening due to coupling pattern with the F nuclei.

^{19}F{^{1}H} NMR (377 MHz, $CDCl_3$) δ = -64.26 (t, $J_{F\text{-}F}$ = 10.4 Hz, 3F, *Z* isomer), -67.49 (dd, $J_{F\text{-}F}$ = 21.1, 11.7 Hz, 3F, *E* isomer), -99.79 (qd, $J_{F\text{-}F}$ = 10.1, 4.0 Hz, 1F, *Z* isomer), -120.56 (dq, $J_{F\text{-}F}$ = 145.5, 21.4 Hz, 1F, *E* isomer), -138.07 (qd, $J_{F\text{-}F}$ = 10.9, 4.1 Hz, 1F, *E* isomer), -157.64 (dq, $J_{F\text{-}F}$ = 145.7, 11.9 Hz, 1F, *Z* isomer).

HRMS (ESI-pos, m/z): calc'd for $C_{10}H_7OF_5S$ $[M]^+$ 270.0132; found 270.0125.

3.5.17 Unsuccessful substrates for defluorothiolation

The following substrate was obtained in less than 10% yield using 5 mol% of stibinidene **1a** after 16 h at 60 ºC:

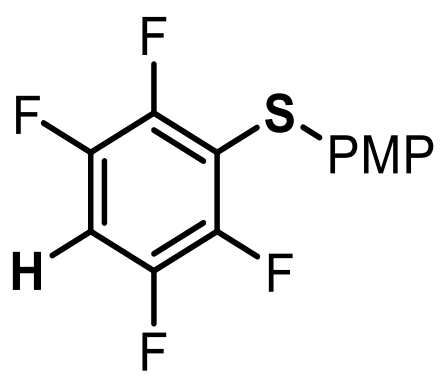

3.6 Results and spectroscopic data for defluoroamination products

3.6.1 2,3,5,6-tetrafluoro-4-(1*H*-imidazol-1-yl)pyridine (**7a**)

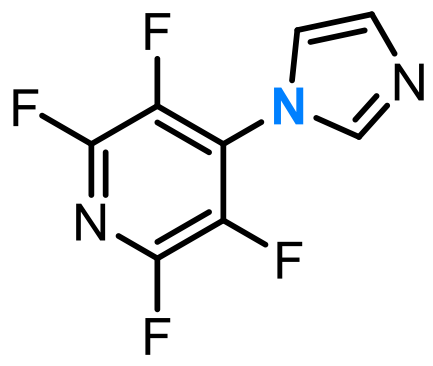

Using the general method with 1.0 equiv. of *N*-trimethylsilyl imidazole **S3a** and pentafluoropyridine resulted in the formation of **7a** at >95% NMR yield after 1 h at rt. The product was isolated as an off-white solid (41 mg, 94% yield) after filtration through a silica plug using DCM as eluent.

Spectroscopic data are in accordance with previously reported values.[18]

^{1}H NMR (400 MHz, CDCl$_3$) δ = 8.02 – 7.91 (bs, 1H), 7.45 – 7.36 (bs, 1H), 7.32 – 7.27 (bs, 1H).

^{13}C{^{1}H} NMR (101 MHz, CDCl$_3$) δ = 146.5 – 142.9 (m, 2 x C), 137.4 (t, $J_{C\text{-}F}$ = 5.6 Hz, CH), 137.3 –133.5 (m, 2 x C), 131.1 (CH), 129.0 – 127.2 (m, C), 119.3 (t, $J_{C\text{-}F}$ = 4.2 Hz, C).

^{19}F{^{1}H} NMR (377 MHz, CDCl$_3$) δ = -83.43 – -90.57 (m, 2F), -144.55 – -153.68 (m, 2F).

3.6.2 2,3,5-trifluoro-4,6-di(1*H*-imidazol-1-yl)pyridine (**7a'**)

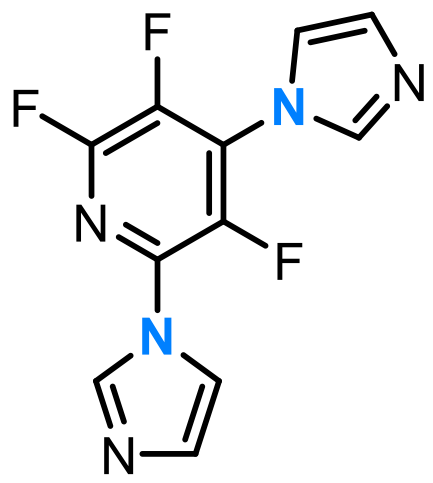

Using the general method with 3.0 equiv. of *N*-trimethylsilyl imidazole **S3a**, pentafluoropyridine and 5 mol% of stibinidene complex **1a** resulted in the formation of **7a'** at >95% NMR yield after 1 h at rt. The product was isolated as an off-white solid (48 mg, 91% yield) using flash column chromatography (Ethyl acetate:Methanol, 20:1, R_f = 0.31)

^{1}H NMR (400 MHz, CDCl$_3$) δ = 8.33 – 8.27 (m, 1H), 8.00 – 7.95 (m, 1H), 7.72 – 7.65 (m, 1H), 7.43 – 7.38 (m, 1H), 7.34 – 7.32 (m, 1H), 7.24 – 7.21 (m, 1H).

^{13}C{^{1}H} NMR (101 MHz, CDCl$_3$) δ =146.7 (ddd, $J_{C\text{-}F}$ = 242.6, 14.7, 2.5 Hz, C), 140.7 – 137.7 (m, 2 x C), 137.4 (t, $J_{C\text{-}F}$ = 4.9 Hz, CH), 136.2 (d, $J_{C\text{-}F}$ = 9.8 Hz, CH), 135.9 (dd, $J_{C\text{-}F}$ = 235.7, 31.8 Hz, C), 131.1 (CH), 131.0 (d, $J_{C\text{-}F}$ = 2.4 Hz, CH), 127.9 – 126.9 (m, C), 119.5 (t, $J_{C\text{-}F}$ = 3.4 Hz, CH), 117.5 (d, $J_{C\text{-}F}$ = 6.4 Hz, CH).

^{19}F{^{1}H} NMR (377 MHz, CDCl$_3$) δ = -83.26 (dd, $J_{F\text{-}F}$ = 28.6, 22.9 Hz, 1F), -139.25 (d, $J_{F\text{-}F}$ = 28.6 Hz, 1F), -147.49 (d, $J_{F\text{-}F}$ = 22.9 Hz, 1F).

HRMS (ESI-pos, m/z): calc'd for $C_{11}H_7N_5F_4$ $[M+H]^+$ 266.0648; found 266.0639.

3.6.3 1-(perfluoropyridin-4-yl)-1*H*-indole (**7b**)

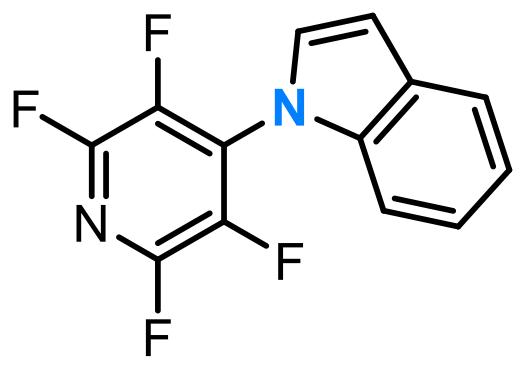

Using the general method with 1.0 equiv. of *N*-trimethylsilyl indole **S3b** and pentafluoropyridine resulted in the formation of **7b** at >95% NMR yield after 1 h at rt. The product was isolated as a colourless oil (50 mg, 94% yield) after filtration through a silica plug using DCM as eluent.

Spectroscopic data are in accordance with previously reported values.[19]

^{1}H NMR (400 MHz, $CDCl_3$) δ = 7.72 (d, $J_{H\text{-}H}$ = 7.9 Hz, 1H), 7.36 – 7.33 (m, 1H), 7.31 – 7.27 (m, 2H), 7.26 – 7.21 (m, 1H), 6.88 (dd, $J_{H\text{-}H}$ = 3.5, 1.0 Hz, 1H).

^{13}C{^{1}H} NMR (101 MHz, $CDCl_3$) δ = 147.0 – 142.3 (m, 2 x C), 139.6 – 135.6 (m, 2 x C), 135.5 (C), 130.7 – 129.8 (m, C), 129.2 (C), 127.5 (t, $J_{C\text{-}F}$ = 2.7 Hz, C), 124.0 (CH), 122.4 (CH), 121.6 (CH), 111.2 (t, $J_{C\text{-}F}$ = 3.4 Hz, CH), 107.8 (CH).

^{19}F{^{1}H} NMR (377 MHz, $CDCl_3$) δ = -87.60 – -88.41 (m, 2F), -145.95 – -146.43 (m, 2F).

3.6.4 4-(perfluoropyridin-4-yl)morpholine (**7c**)

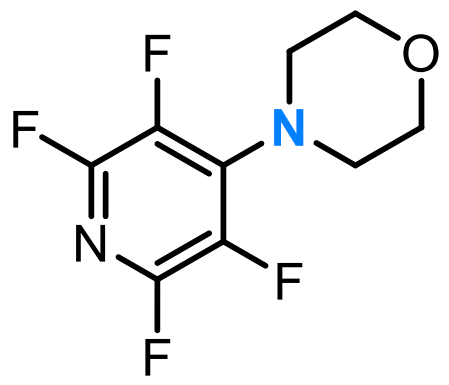

Using the general method with 1.0 equiv. of *N*-trimethylsilyl morpholine **S3c** and pentafluoropyridine resulted in the formation of **7c** at >95% NMR yield after 1 h at rt. The product was isolated as a colourless oil (43 mg, 92% yield) after filtration through a silica plug using DCM as eluent.

Spectroscopic data are in accordance with previously reported values.[20]

^{1}H NMR (400 MHz, $CDCl_3$) δ = 3.81 (dd, $J_{H\text{-}H}$ = 5.5, 3.9 Hz, 4H), 3.48 (ddt, $J_{H\text{-}H,H\text{-}F}$ = 4.6, 3.3, 1.7 Hz, 4H).

^{13}C{^{1}H} NMR (101 MHz, $CDCl_3$) δ = 148.0 – 142.8 (m, 2 x C), 141.2 – 138.5 (m, C), 137.1 – 131.3 (m, 2 x C), 67.1 (2 x CH_2), 50.5 (t, $J_{C\text{-}F}$ = 4.6 Hz, 2 x CH_2).

^{19}F{^{1}H} NMR (377 MHz, $CDCl_3$) δ = -88.93 – -98.41 (m, 2F), -151.33 – -158.82 (m, 2F).

3.6.5 Methyl 2,3,5,6-tetrafluoro-4-(1*H*-imidazol-1-yl)benzoate (**7d**)

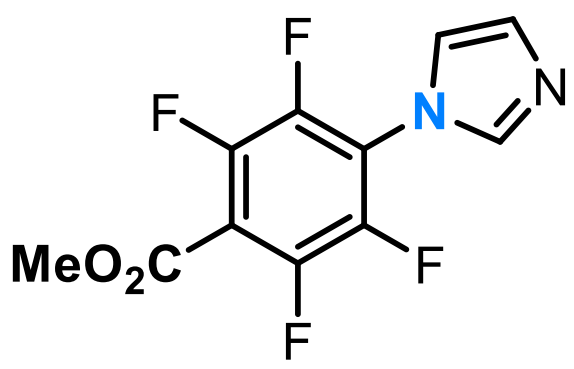

Using the general method with 1.0 equiv. of *N*-trimethylsilyl imidazole **S3a** and methyl pentafluorobenzoate resulted in the formation of **7d** at >95% NMR yield after 2 h at rt. The product was isolated as a white solid (53 mg, 96% yield) using flash column chromatography (Hexane:Ethyl acetate, 3:2).

^{1}H NMR (400 MHz, CDCl$_3$) δ = 7.82 (s, 1H), 7.32 – 7.24 (m, 2H), 4.00 (s, 3H).

^{13}C{^{1}H} NMR (101 MHz, CDCl$_3$) δ = 159.4 (C), 147.3 – 144.0 (m, 2 x C), 143.1 – 139.3 (m, 2 x C), 137.6 (CH), 130.6 (CH), 128.4 (C), 119.9 (CH), 111.8 – 111.5 (m, C), 53.7(CH$_3$).

^{19}F{^{1}H} NMR (377 MHz, CDCl$_3$) δ = -136.89 – -137.10 (m, 2F), -147.21 – -147.39 (m, 2F).

HRMS (ESI-pos, m/z): calc'd for $C_{11}H_7O_2N_2F_4$ $[M+H]^+$ 275.0438; found 275.0429.

3.6.6 1-(2,3,5,6-tetrafluoro-4-(trifluoromethyl)phenyl)-1*H*-imidazole (**7e**)

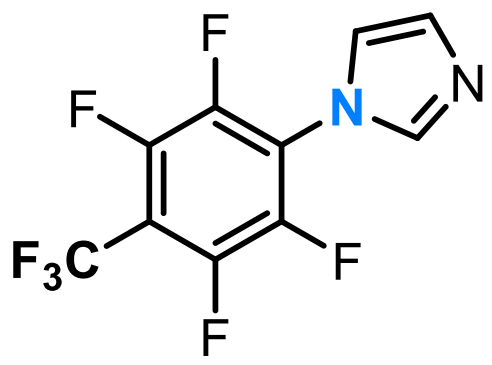

Using the general method with 1.0 equiv. of *N*-trimethylsilyl imidazole **S3a** and octafluorotoluene resulted in the formation of **7e** at 75% NMR yield after 2 h at rt. The product was isolated as a colourless oil (40 mg, 71% yield) using flash column chromatography (Hexane:Ethyl acetate, 3:2).

Spectroscopic data are in accordance with previously reported ones.[21]

^{1}H NMR (400 MHz, CDCl$_3$) δ = 7.85 (s, 1H), 7.31 (bs, 1H), 7.29 – 7.27 (m, 1H).

^{13}C{^{1}H} NMR (101 MHz, CDCl$_3$) δ = 146.6 – 143.4 (m, 2 x C), 142.7 – 139.5 (m, 2 x C), 137.4 (CH), 130.7 (CH), 119.6 (CH). Some C atoms directly attached to fluorine atoms could not be observed due to low intensity and broadening due to coupling pattern with the F nuclei.

^{19}F{^{1}H} NMR (377 MHz, CDCl$_3$) δ = -56.16 (t, $J_{F\text{-}F}$ = 21.7 Hz, 3F), -137.64 – -138.17 (m, 2F), -146.08 – -146.24 (m, 2F).

3.6.7 Unsuccessful substrates for defluoroamination

The following substrates were obtained in less than 10% yield using 5 mol% of stibinidene **1a** under 16 h at 60 ºC:

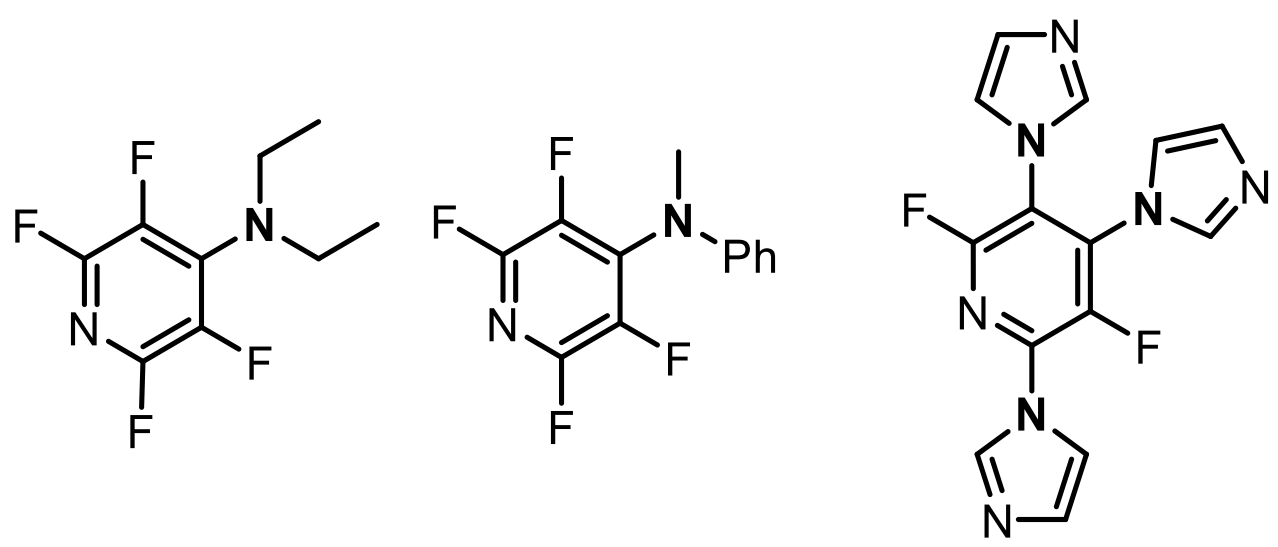

3.7 Results and spectroscopic data for defluorophosphonylation products

3.7.1 Diphenyl (perfluoropyridin-4-yl)phosphonate (**8a**)

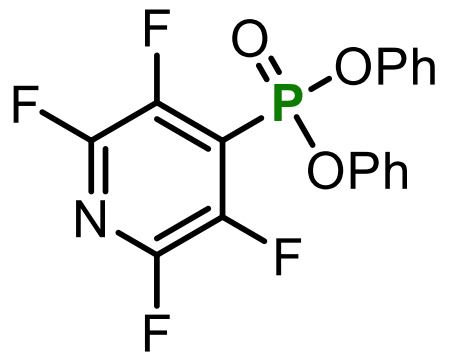

Using the general method with 1.0 equiv. of diphenyl(trimethylsilyl) phosphite **S4a**, pentafluoropyridine and 5 mol% of stibinidene complex **1a** resulted in the formation of **8a** at 50% NMR yield after 2 h at 60 ºC. The product was isolated as an off-white solid (34 mg, 44% yield) after filtration through a silica plug using DCM as eluent.

^{1}H NMR (400 MHz, CDCl$_3$) δ = 7.44 – 7.36 (m, 4H), 7.30 – 7.24 (m, 6H).

^{13}C{^{1}H} NMR (101 MHz, CDCl$_3$) δ = 149.5 (d, $J_{C\text{-}P}$ = 7.8 Hz, 2 x C), 130.3 (4 x C), 126.4 (2 x C), 120.4 (d, $J_{C\text{-}P}$ = 4.9 Hz, 4 x C). C atoms directly attached to fluorine atoms could not be observed due to low intensity and broadening due to coupling pattern with the F nuclei.

^{19}F{^{1}H} NMR (377 MHz, CDCl$_3$) δ = -81.79 – -94.08 (m, 2F), -125.11 – -142.20 (m, 2F).

^{31}P NMR (162 MHz, CDCl$_3$) δ = -7.08 (pent, $J_{P\text{-}F}$ = 7.8 Hz).

HRMS (ESI-pos, m/z): calc'd for $C_{17}H_{11}O_3NF_4P$ $[M+H]^+$ 384.0407; found 384.0397.

3.7.2 Diethyl (perfluoropyridin-4-yl)phosphonate (**8b**)

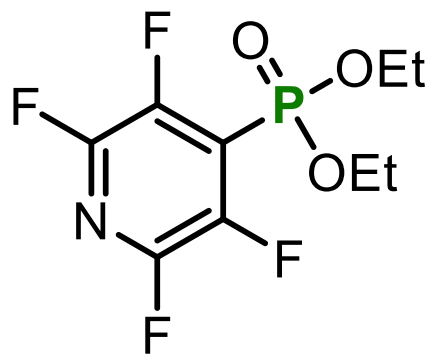

Using the general method with 1.0 equiv. of diethyl(trimethylsilyl) phosphite **S4b**, pentafluoropyridine and 5 mol% of stibinidene complex **1a** resulted in the formation of **8b** at 91% NMR yield after 16 h at 60 ºC. The product was isolated as a colourless oil (51 mg, 89% yield) after filtration through a silica plug using DCM as eluent.

^{1}H NMR (400 MHz, CDCl$_3$) δ = 4.48 – 4.13 (m, 4H), 1.40 (td, $J_{H\text{-}H,\,H\text{-}P}$ = 7.1, 0.9 Hz, 6H).

^{13}C{^{1}H} NMR (101 MHz, CDCl$_3$) δ = 64.3 (d, $J_{C\text{-}P}$ = 6.4 Hz, 2 x CH_2), 16.3 (d, $J_{C\text{-}P}$ = 6.4 Hz, 2 x CH_3). C atoms directly attached to fluorine atoms could not be observed due to low intensity and broadening due to coupling pattern with the F nuclei.

^{19}F{^{1}H} NMR (377 MHz, CDCl$_3$) δ = -86.58 – -94.08 (m, 2F), -127.93 – -138.24 (m, 2F).

^{31}P NMR (162 MHz, CDCl$_3$) δ = 2.20 – 0.89 (m).

HRMS (ESI-pos, m/z): calc'd for $C_9H_{11}O_3NF_4P$ $[M+H]^+$ 288.0407; found 288.0401.

3.7.3 Diethyl (4-cyano-2,3,5,6-tetrafluorophenyl)phosphonate (**8c**)

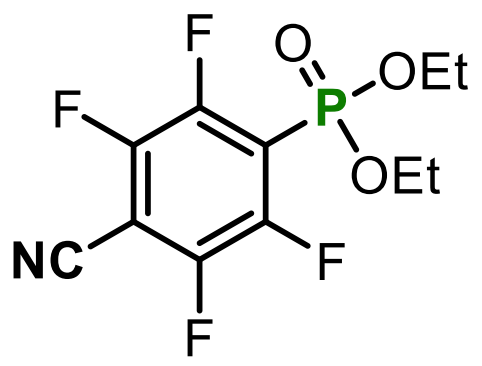

Using the general method with 1.0 equiv. of diethyl(trimethylsilyl) phosphite **S4b**, pentafluorobenzonitrile and 5 mol% of stibinidene complex **1a** resulted in the formation of **8c** at 72% NMR yield after 16 h at 60 ºC. The product was isolated as an orangish oil (43 mg, 69% yield) after filtration through a silica plug using DCM as eluent.

^{1}H NMR (400 MHz, CDCl$_3$) δ = 4.44 – 4.18 (m, 4H), 1.39 (td, $J_{H\text{-}H,\ H\text{-}P}$ = 7.0, 0.8 Hz, 6H).

^{13}C{^{1}H} NMR (101 MHz, CDCl$_3$) δ = 64.3 (d, $J_{C\text{-}P}$ = 6.4 Hz, 2 x CH$_2$), 16.3 (d, $J_{C\text{-}P}$ = 6.4 Hz, 2 x CH$_3$). Some C atoms directly attached to fluorine atoms could not be observed due to low intensity and broadening due to coupling pattern with the F nuclei.

^{19}F{^{1}H} NMR (377 MHz, CDCl$_3$) δ = -127.95 – -128.81 (m, 2F), -129.64 – -131.15 (m, 2F).

^{31}P NMR (162 MHz, CDCl$_3$) δ = 3.26 – 1.97 (m).

HRMS (ESI-pos, m/z): calc'd for $C_{11}H_{11}O_3NF_4P$ [M+H]$^+$ 312.0407; found 312.0396.

3.7.4 Diethyl (2,3,5,6-tetrafluoro-4-(trifluoromethyl)phenyl)phosphonate (**8d**)

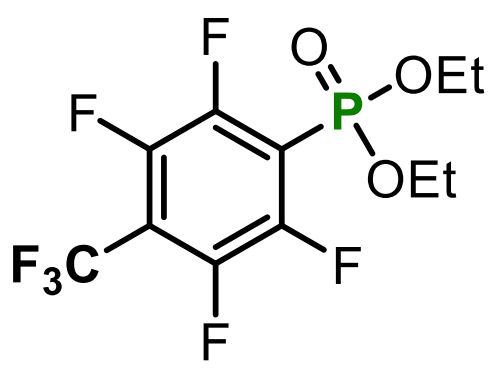

Using the general method with 1.0 equiv. of diethyl(trimethylsilyl) phosphite **S4b**, octafluorotoluene and 5 mol% of stibinidene complex **1a** resulted in the formation of **8d** at >95% NMR yield after 16 h at 60 ºC. The product was isolated as a colourless oil (66 mg, 93% yield) after filtration through a silica plug using DCM as eluent.

^{1}H NMR (400 MHz, CDCl$_3$) δ = 4.55 – 4.03 (m, 4H), 1.39 (td, $J_{H\text{-}P,\ H\text{-}H}$ = 7.1, 0.9 Hz, 6H).

^{13}C{^{1}H} NMR (101 MHz, CDCl$_3$) δ = δ 64.2 (d, $J_{C\text{-}P}$ = 6.4 Hz, 2 x CH$_2$), 16.4 (d, $J_{C\text{-}P}$ = 6.4 Hz, 2 x CH$_3$). C atoms directly attached to fluorine atoms could not be observed due to low intensity and broadening due to coupling pattern with the F nuclei.

^{19}F{^{1}H} NMR (377 MHz, CDCl$_3$) δ = -56.93 (t, $J_{F\text{-}F}$ = 21.7 Hz, 3F), -121.14 – -133.08 (m, 2F), -134.61 – -143.39 (m, 2F).

^{31}P NMR (162 MHz, CDCl$_3$) δ = 3.38 – 2.30 (m).

HRMS (ESI-pos, m/z): calc'd for $C_{11}H_{11}F_7O_3P$ [M+H]$^+$ 355.0329; found 355.0320.

3.8 Results and spectroscopic data for defluoroselenation products

3.8.1 2,3,5,6-tetrafluoro-4-(phenylselanyl)pyridine (**9a**)

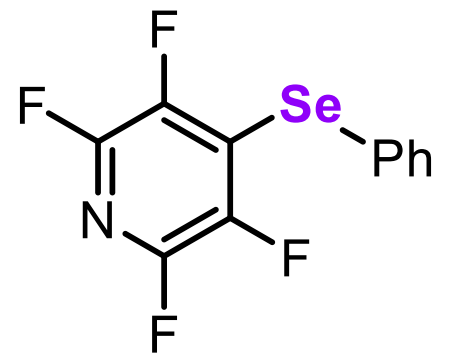

Using the general method with 1.0 equiv. of silyl selenoether **S5** and pentafluoropyridine resulted in the formation of **9a** at >95% NMR yield after 1 h at rt. The product was isolated as an off-white oil (108 mg, 94% yield) using flash column chromatography (Hexane:Ethyl acetate, 10:1).

Spectroscopic data are in accordance with previously reported values.[22]

^{1}H NMR (400 MHz, CDCl$_3$) δ = 7.67 (d, $J_{H\text{-}H}$ = 7.3 Hz, 2H), 7.47 – 7.38 (m, 1H), 7.37 – 7.32 (m, 2H).

^{13}C{^{1}H} NMR (101 MHz, CDCl$_3$) δ = 144.8 – 142.0 (m, 2 x C), 143.3 – 140.1 (m, 2 x C), 135.3 (2 x CH), 129.9 (2 x CH), 129.7 (C), 125.4 (CH). C atoms directly attached to fluorine atoms could not be observed due to low intensity and broadening due to coupling pattern with the F nuclei.

^{19}F{^{1}H} NMR (377 MHz, CDCl$_3$) δ = -90.70 – -90.93 (m, 2F), -131.36 – -131.64 (m, 2F).

3.9 Results and spectroscopic data for defluoroarylation products

3.9.1 Perfluoro-4,4'-bipyridine (**10a**)

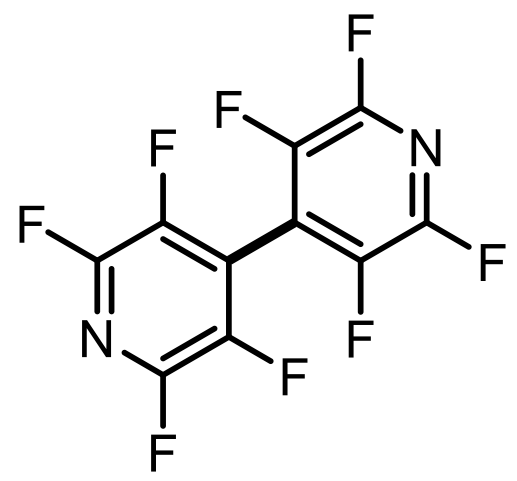

Using the general method with 0.50 equiv. of Mashima's reagent **S6** and 5 mol% of stibinidene complex **1a** resulted in the formation of **10a** at 91% NMR yield after 2 h at rt. The product was isolated as an off-white solid (52 mg, 87% yield) after filtration through a silica plug using DCM as eluent.

Spectroscopic data are in agreement with previously reported ones.[23]

^{19}F{^{1}H} NMR – (377 MHz, CDCl$_3$) δ = -85.84 – -88.04 (m, 4F), -137.17 – -138.52 (m, 4F).

3.9.2 2,2',3,3',5,5',6,6'-octafluoro-4,4'-bis(trifluoromethyl)-1,1'-biphenyl (**10b**)

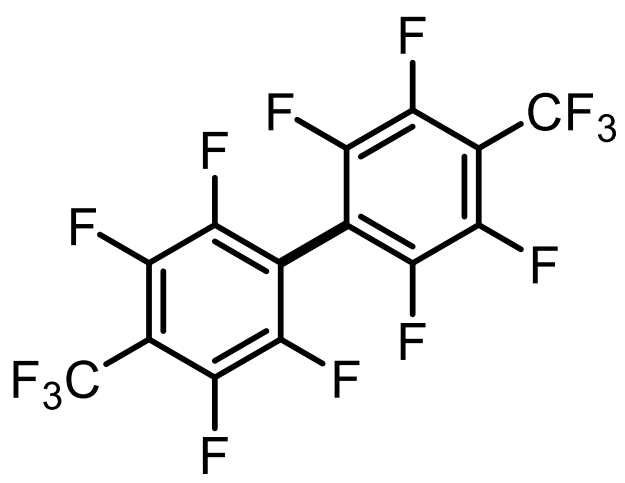

Using the general method with 0.50 equiv. of Mashima's reagent **S6** and 5 mol% of stibinidene complex **1a** resulted in the formation of **10b** at 50% NMR yield after 2 h at rt. The product was isolated as an off-white solid (36 mg, 41% yield) after filtration through a silica plug using DCM as eluent.

Spectroscopic data are in agreement with previously reported ones.[24]

$^{19}F\{^1H\}$ NMR – (377 MHz, $CDCl_3$) δ = -56.51 (t, $J_{F\text{-}F}$ = 21.7 Hz, 6F), -134.81 – -135.13 (m, 4F), -137.96 – -138.36 (m, 4F).

3.9.3 Unsuccessful substrates for defluoroalkylation or defluoroarylation

The following substrates were obtained in less than 10% yield using 5 mol% of stibinidene **1a** under 16 h at 60 ºC or more polar solvents (THF and CH_3CN):

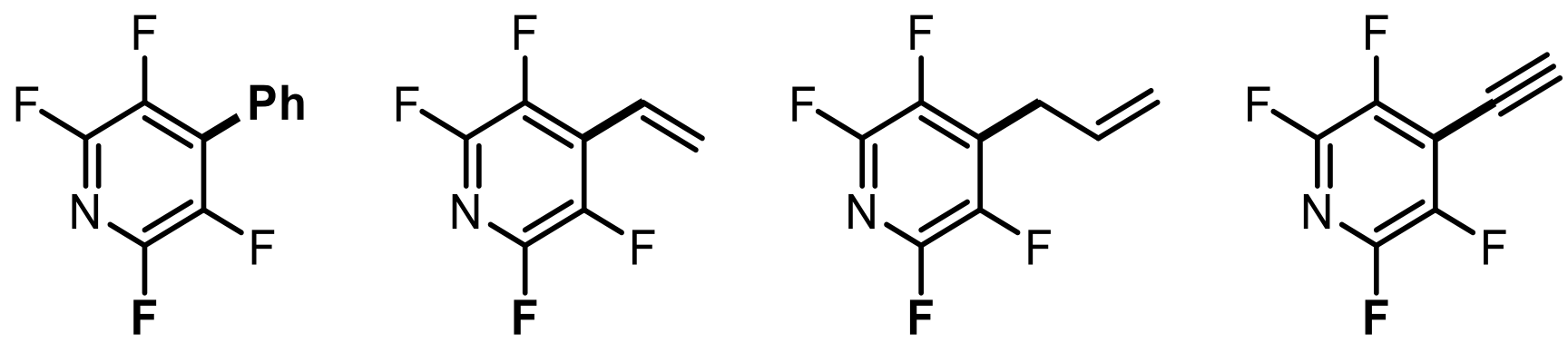

4 Blank reactions

Following the general procedure, the blank reactions between pentafluoropyridine and the representative nucleophiles for each C-X bond formation protocol (**S1a**, **S2b**, **S3a**, **S4b** and **S5** and the reducing agent **S6**) were tested, and the results are depicted below.

Table S3: Blank reactions for model C-X (X = O, S, N, P, Se and C) bond formation reactions in the present study.

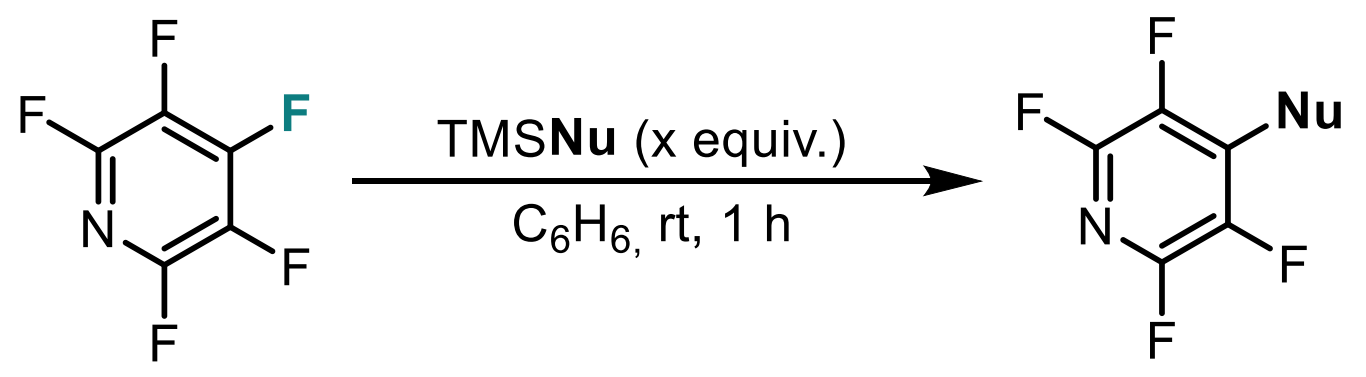

Entry	TMSNu	Yield (%)[a]
1	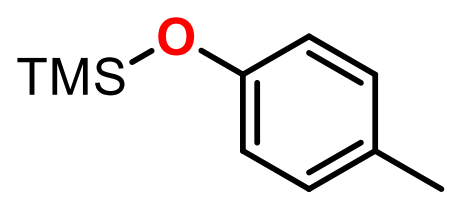 **S1a**	0
2	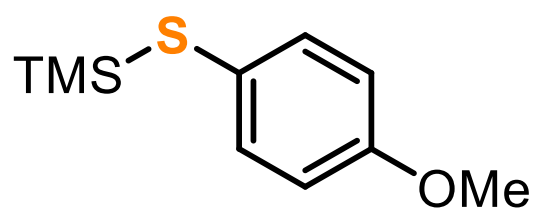 **S2b**	0
3-5	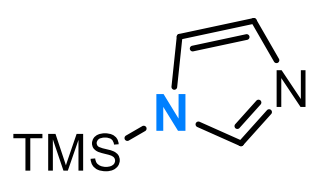 **S3a**	73[b], 30[c] or <10[d]
6	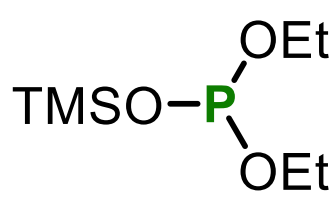 **S4b**	23%[e,f]
7	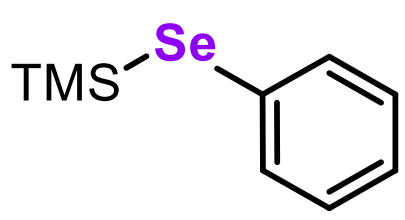 **S5**	<5%[e]
8	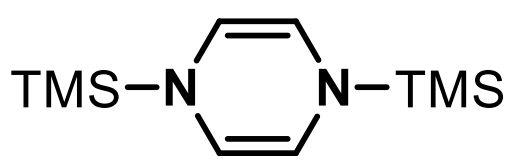 **S6**	0

[a]Calculated by ^{19}F NMR using α,α,α-trifluorotoluene (0.50 equiv.) as IS. [b]**S3a** containing ~15% imidazole. [c]**S3a** freshly distilled (<5% content of imidazole). [d]Reaction analysed after 15 min. [e]3.0 equivalents of TMS**Nu**. [f]Reaction performed at 60 °C for 16 h.

5 Mechanistic studies

5.1 Radical trap tests for defluoroalkoxylation reaction

Different radical trap inhibition experiments were conducted to explore the mechanism of this transformation. The defluoroalkoxylation of pentafluoropyridine with silyl ether **S1a** in the presence of 1.0 equiv. of the reagents listed below could proceed smoothly affording the expected products in the yields shown below, suggesting that SET processes do not take place in the present transformation.

Table S4. Table showing catalytic defluoroalkoxylation of pentafluoropyridine using **1** in the presence of different radical traps

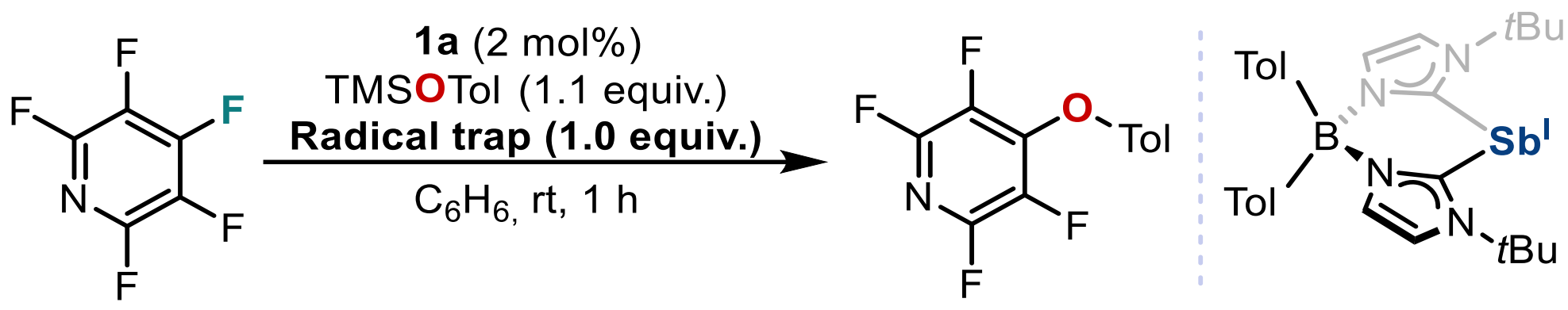

0.2 mmol | **2a** | **1a**

Entry	Reagent	Yield (%)[a]
1	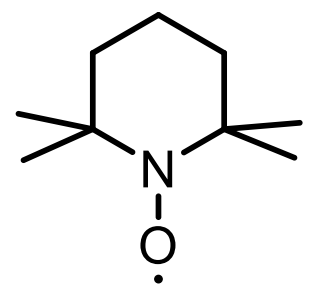	>95
2	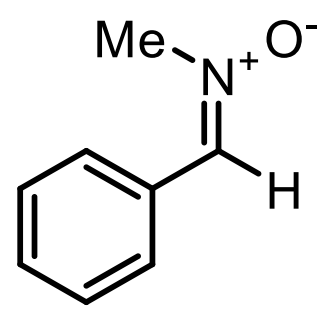	>95

[a]Estimated by ^{19}F NMR using α,α,α-trifluorotoluene (0.50 equiv.) as IS.

5.2 Radical trap tests for defluorothiolation reaction

Different radical trap inhibition experiments were conducted to explore the mechanism of this transformation. The defluorothiolation of pentafluoropyridine with silyl thioether **S2b** in the presence of 1.0 equiv. of the reagents listed below could proceed smoothly affording the expected products in the yields shown below, suggesting that SET processes do not take place in the present transformation.

Table S5. Table showing catalytic defluorothiolation of pentafluoropyridine using **1** in the presence of different radical traps

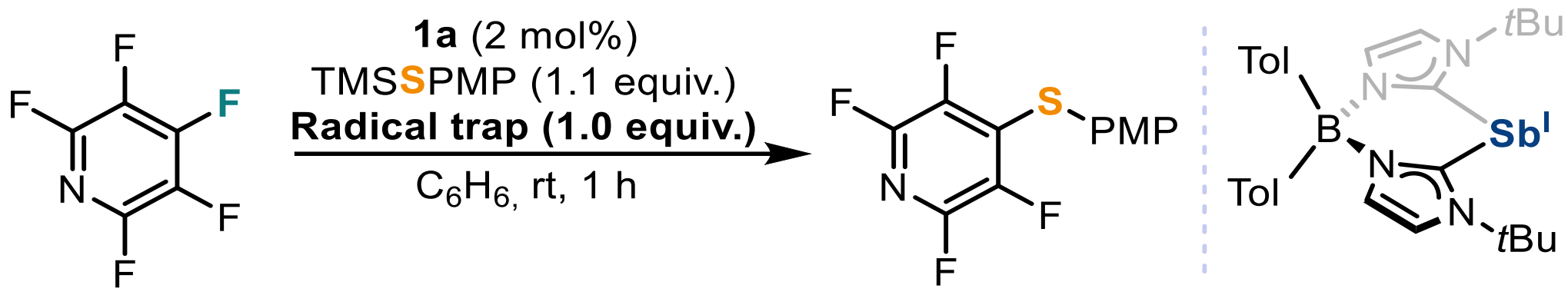

0.2 mmol **6b** **1a**

Entry	Reagent	Yield (%)[a]
1	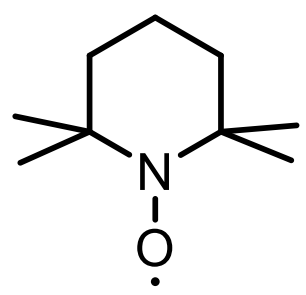	>95
2	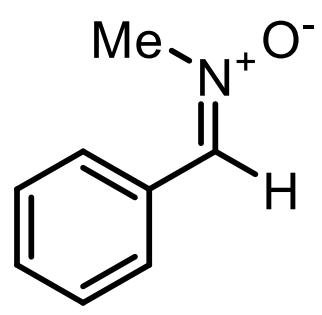	>95

[a]Estimated by ^{19}F NMR using α,α,α-trifluorotoluene (0.50 equiv.) as IS.

5.3 Attempts of reductive elimination of C-X bonds from 11

5.3.1 Attempt stoichiometric C-O bond formation from complex **11**

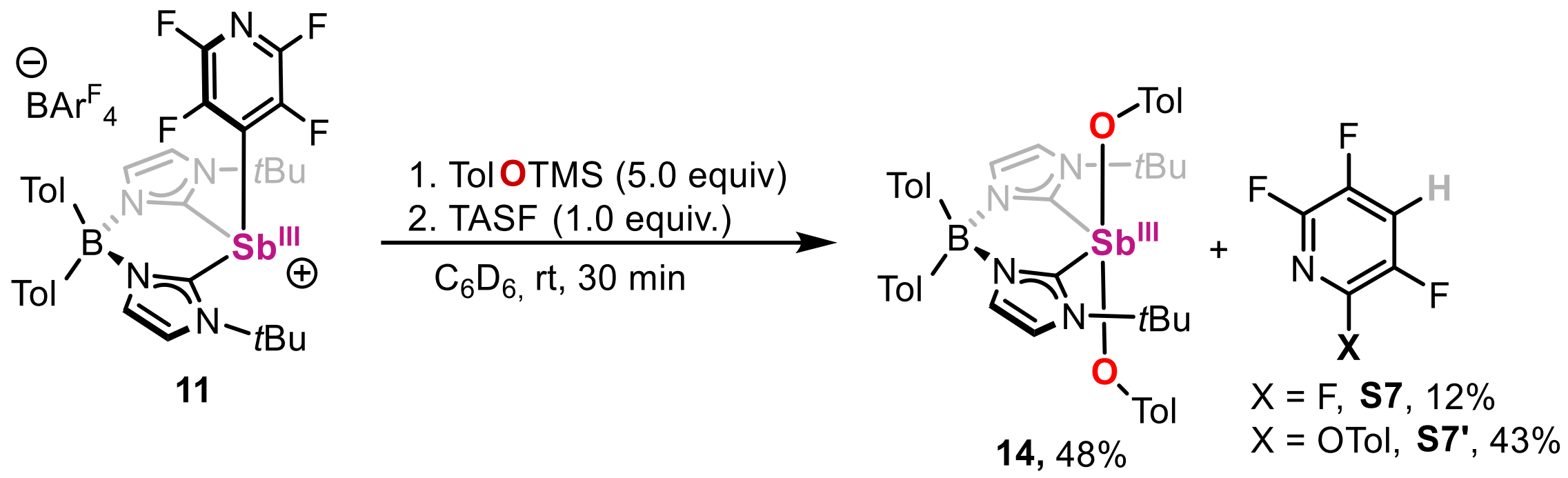

To a solution of Sb(III) complex **11** (39 mg, 1.0 equiv., 0.025 mmol) in 0.3 mL of C_6D_6 was added trimethyl(*p*-tolyloxy)silane **S1a** (22.5 mg, 5.0 equiv., 0.125 mmol) in 0.2 mL of C_6D_6. The resulting mixture was stirred for a further 15 min, transferred to a J-Young NMR tube and analysed by ^{1}H NMR (**Figure S2**) and ^{19}F NMR (**Figure S3**), which showed no reaction between **11** and **S1a**.

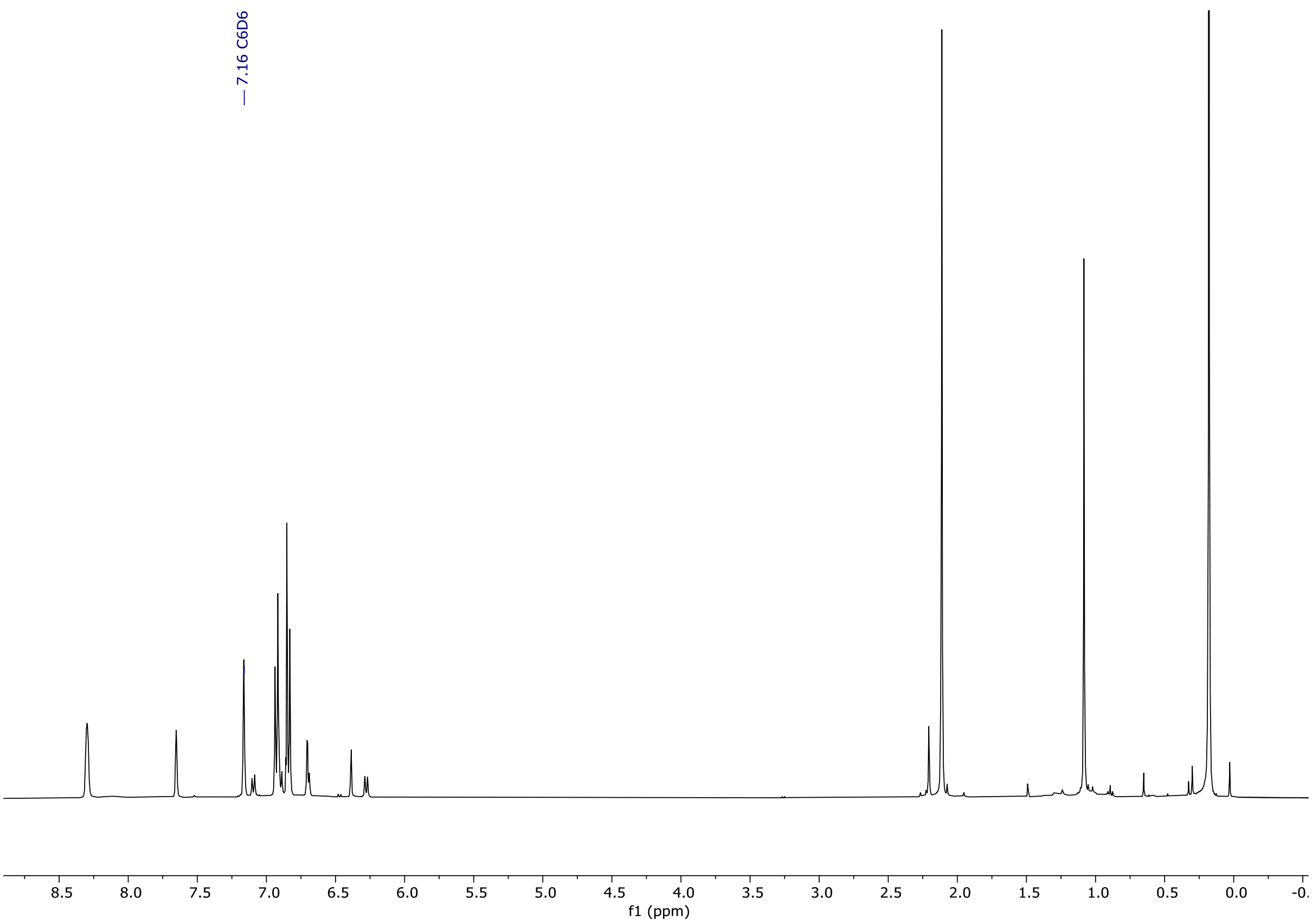

Figure S2. ^{1}H NMR spectrum of the reaction mixture of **11** with trimethyl(*p*-tolyloxy)silane **S1a** in C_6D_6.

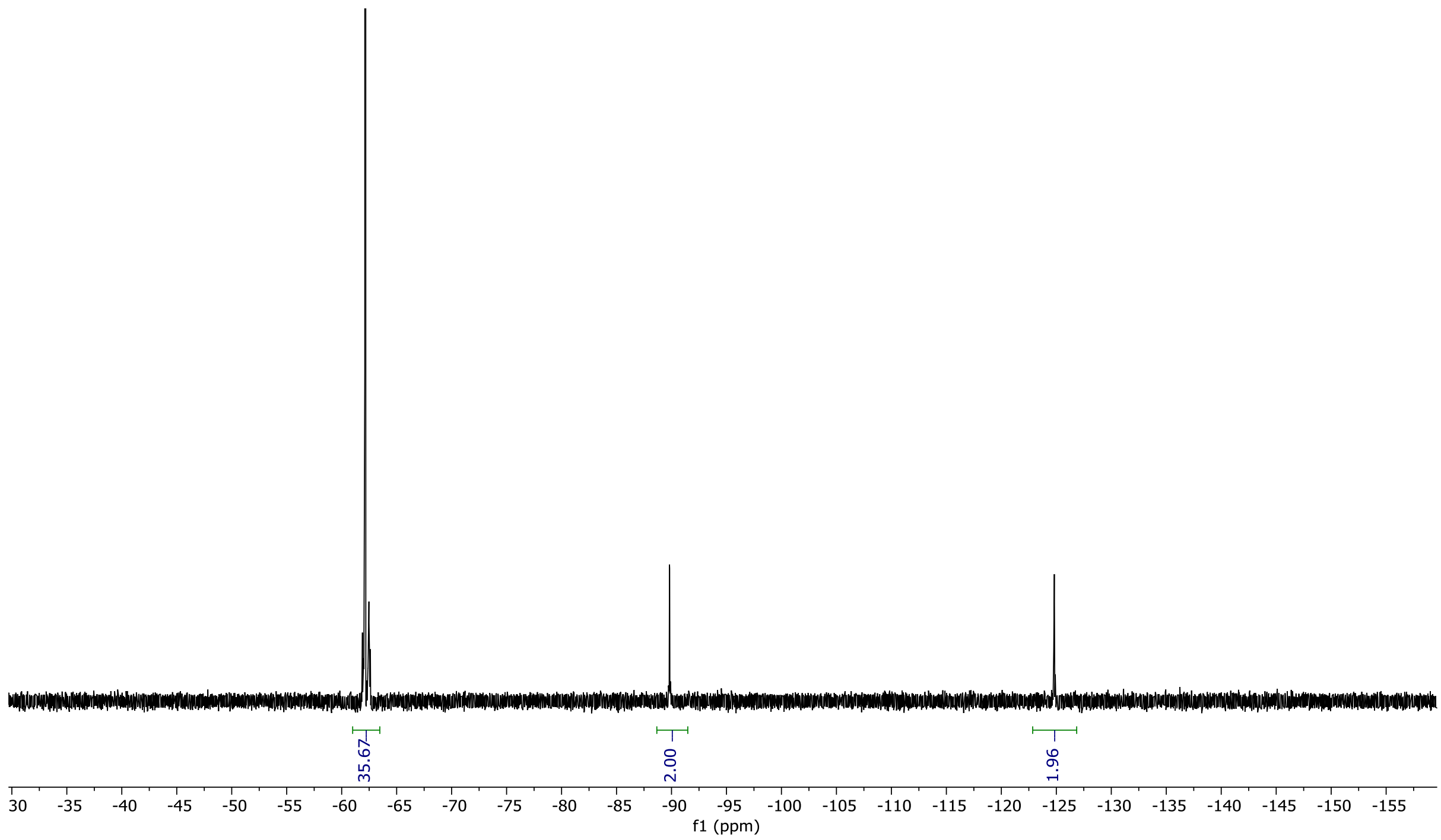

Figure S3. ^{19}F NMR spectrum of the reaction mixture of **11** with trimethyl(*p*-tolyloxy)silane **S1a** in C_6D_6.

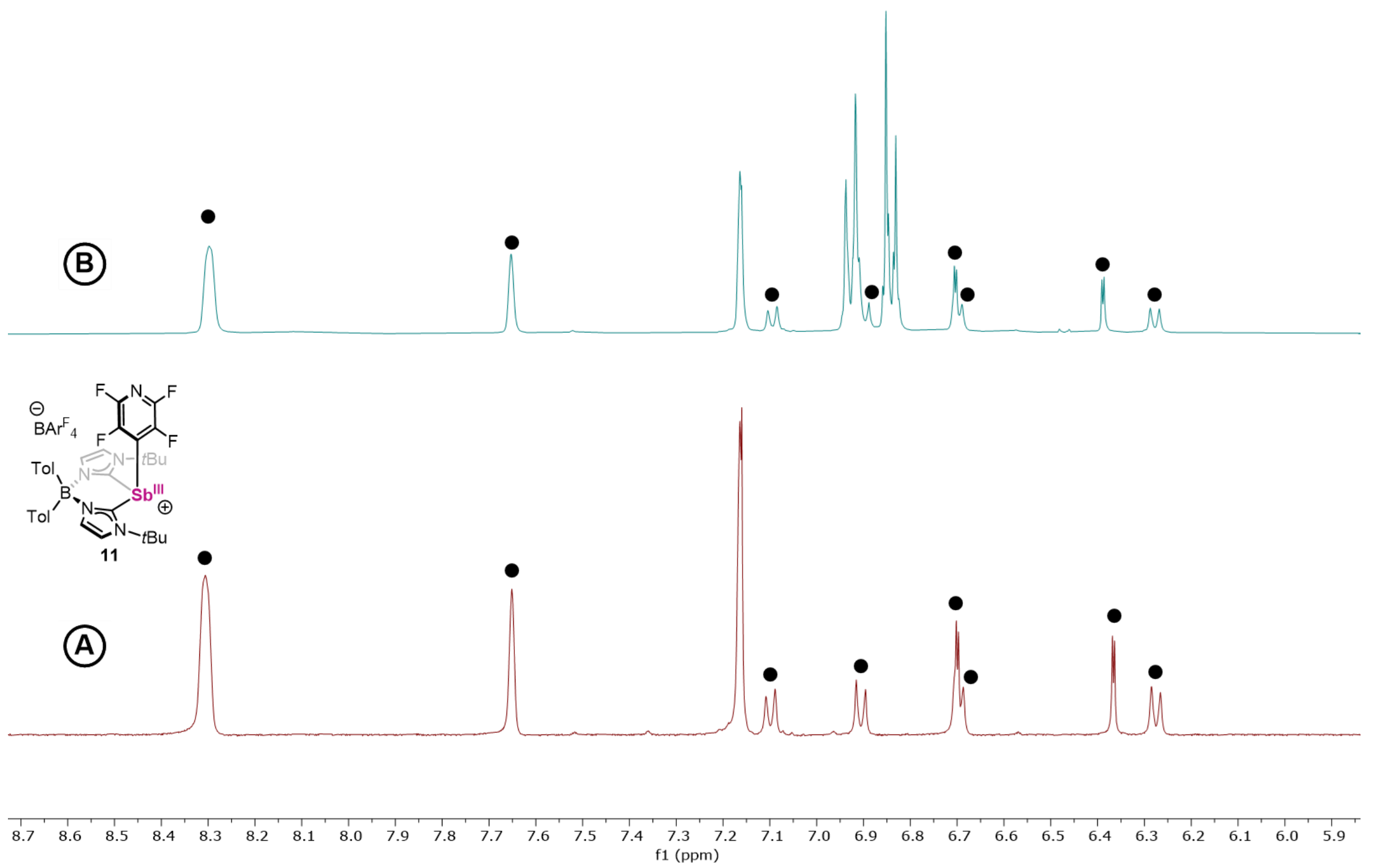

Figure S4. Stacked ^1H NMR spectra of (**A**) Sb(III) complex **11** (black dots); and (**B**) reaction mixture of **11** with trimethyl(*p*-tolyloxy)silane **S1a**. Both spectra were recorded in C_6D_6.

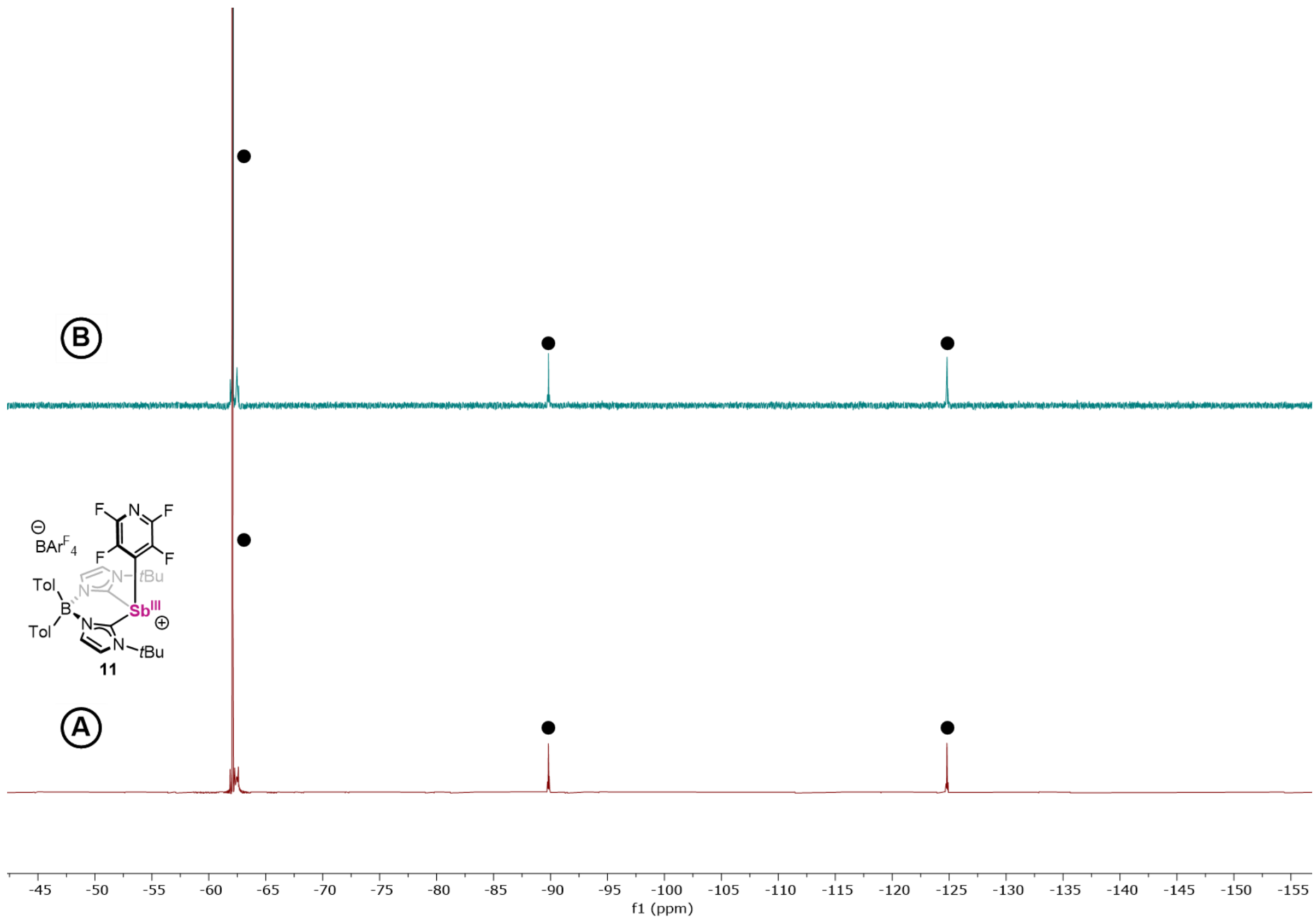

Figure S5. Stacked ^{1}H NMR spectra of (**A**) Sb(III) complex **11** (black dots); and (**B**) reaction mixture of **11** with trimethyl(*p*-tolyloxy)silane **S1a**. Both spectra were recorded in C_6D_6.

The sample was transferred back to the reaction vial inside the glovebox and α,α,α -trifluorotoluene (3.0 μL, 1.0 equiv., 0.025 mmol), 1,3,5-trimethoxybenzene (2.4 mg, 0.33 equiv., 0.0125 mmol) and tris(dimethylamino)sulfonium difluorotrimethylsilicate (6.9 mg, 1.0 equiv., 0.025 mmol) in 0.2 mL of C_6D_6 were added in one portion. The resulting mixture was stirred for further 30 min, transferred to a J-Young NMR tube and analysed by ^{1}H NMR (**Figure S6**) and ^{19}F NMR (**Figure S7**) again. In this case, the ^{1}H NMR spectra showed a major Sb-containing species, identified as Sb(III) dialkoxide complex **14**, as well as free borate ligand and TMSF. The ^{19}F NMR exhibited multiple signals, but none of them matching that of Sb(III) difluoride species **15**. The major species was initially misassigned as bipyridine **10a** and **10a'**, expecting a reductive elimination would take place prior a consecutive alkoxylation. However, an alternative synthesis of bipyridine **10a'** through our defluoroalkoxylation method (see Section 4.53) allowed enabled to reassign such species as alkoxylated product **S7'** in 43% yield. This result aligns perfectly with DFT studies shown in **Figure S39**, as a reductive elimination step to forge bipyridine **10a** is highly unlikely to proceed in C_6H_6 at room temperature. Noteworthy, pyridines **S7** and **S7'** could arise from decomposition and consecutive alkoxylation of the presumable Sb(III) diaryl species **16** shown in Scheme 2 (main

text). However, catalytic or stoichiometric defluoroalkoxylation processes of 2,3,5,6-tetrafluoropyridine **S7** using species **14** did not afford any product. Fortunately, species **S7'** could be synthesised by a different methodology to confirm our assignment (see *Section 4.5.2*). Therefore, species **S7'** might arise exclusively from decomposition of the presumable Sb(III) diaryl species **16** and silane **S1a** without involving species **14**. The ^{19}F NMR also displays peaks corresponding to TMSF in 60% yield and two other non-identified F-containing species in trace amounts.

On the following Figures (Figure S8-S12) different stacked NMR spectra are provided to illustrate the observed species during the reaction as explained above.

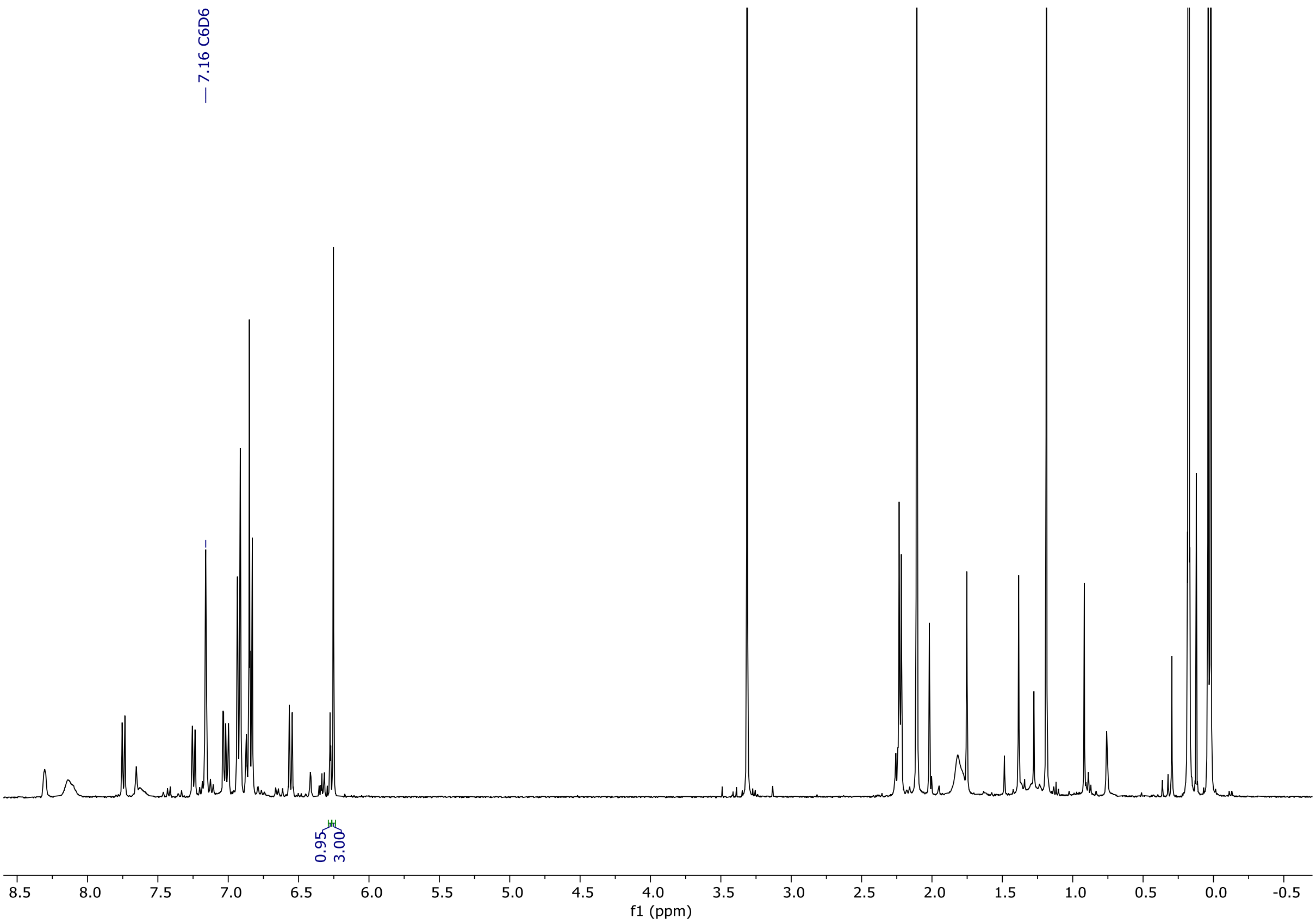

Figure S6. ^{1}H NMR spectrum of the reaction mixture of **11** with trimethyl(*p*-tolyloxy)silane **S1a**, TASF, 1,3,5-trimethoxybenzene and α,α,α-trifluorotoluene after 30 min in C_6D_6.

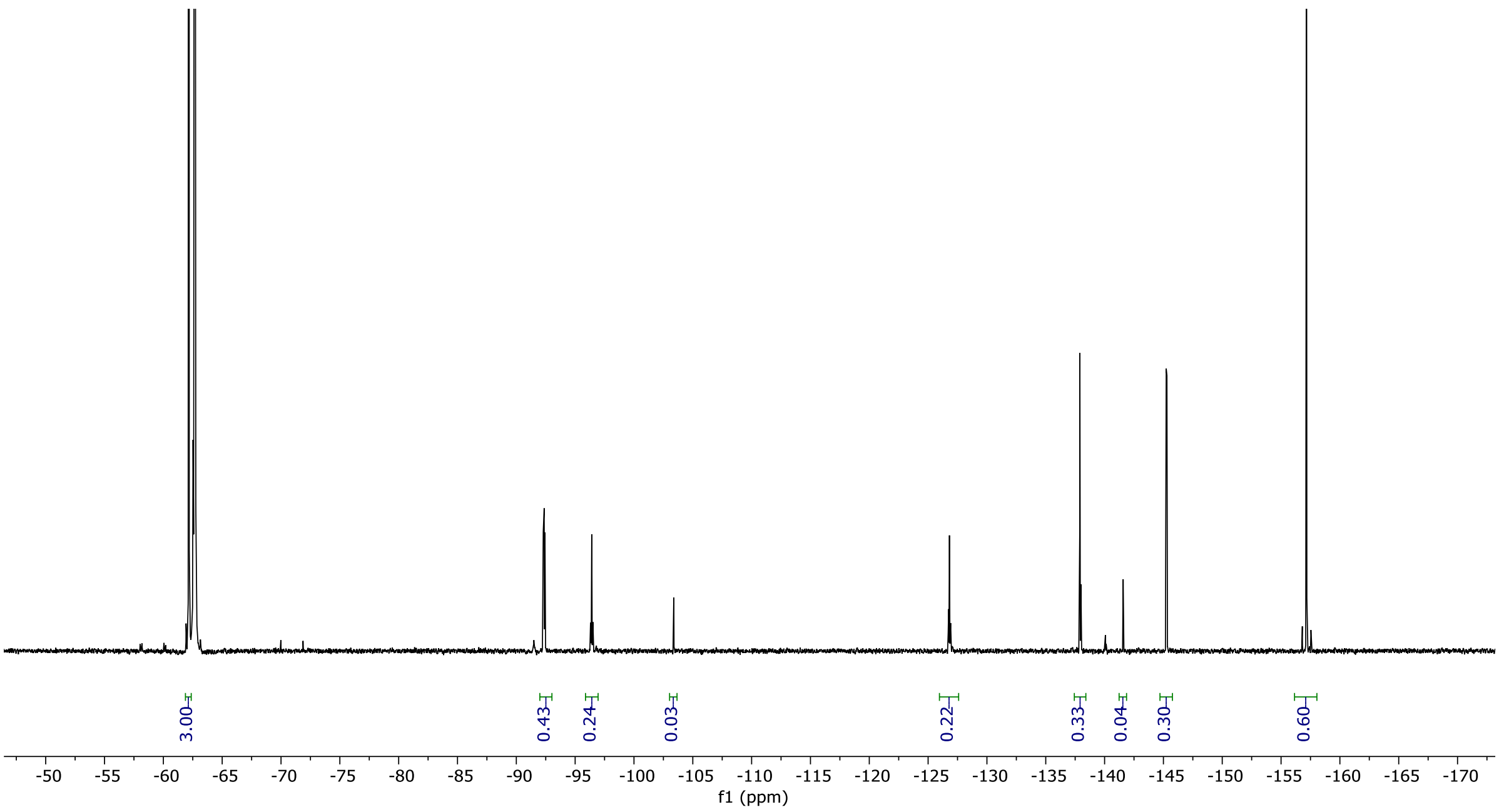

Figure S7. ^{19}F NMR spectrum of the reaction mixture of **11** with trimethyl(*p*-tolyloxy)silane **S1a**, TASF, 1,3,5-trimethoxybenzene and α,α,α-trifluorotoluene after 30 min in C_6D_6.

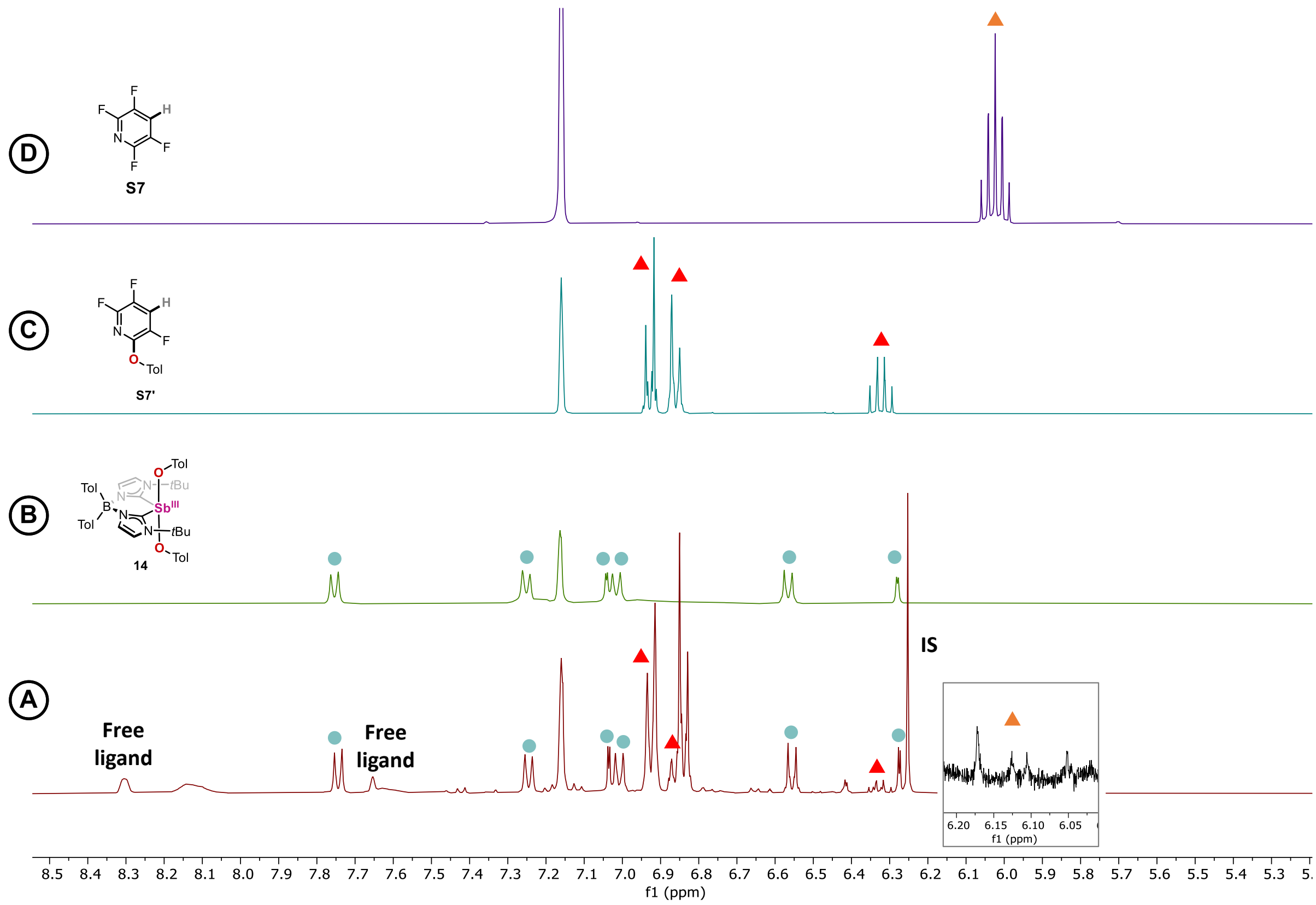

Figure S8. Stacked ^1H NMR spectra (aromatic region) of (**A**) reaction mixture of **11** with trimethyl(*p*-tolyloxy)silane **S1a**, TASF, and 1,3,5-trimethoxybenzene and α,α,α-trifluorotoluene as IS; (**B**) Sb(III) dialkoxide complex **14** (blue dots); (**C**) alkoxylated pyridine **S7'** (red triangles); and (**D**) 2,3,5,6-tetrafluoropyridine **S7** (orange triangles). All the spectra were recorded in C_6D_6.

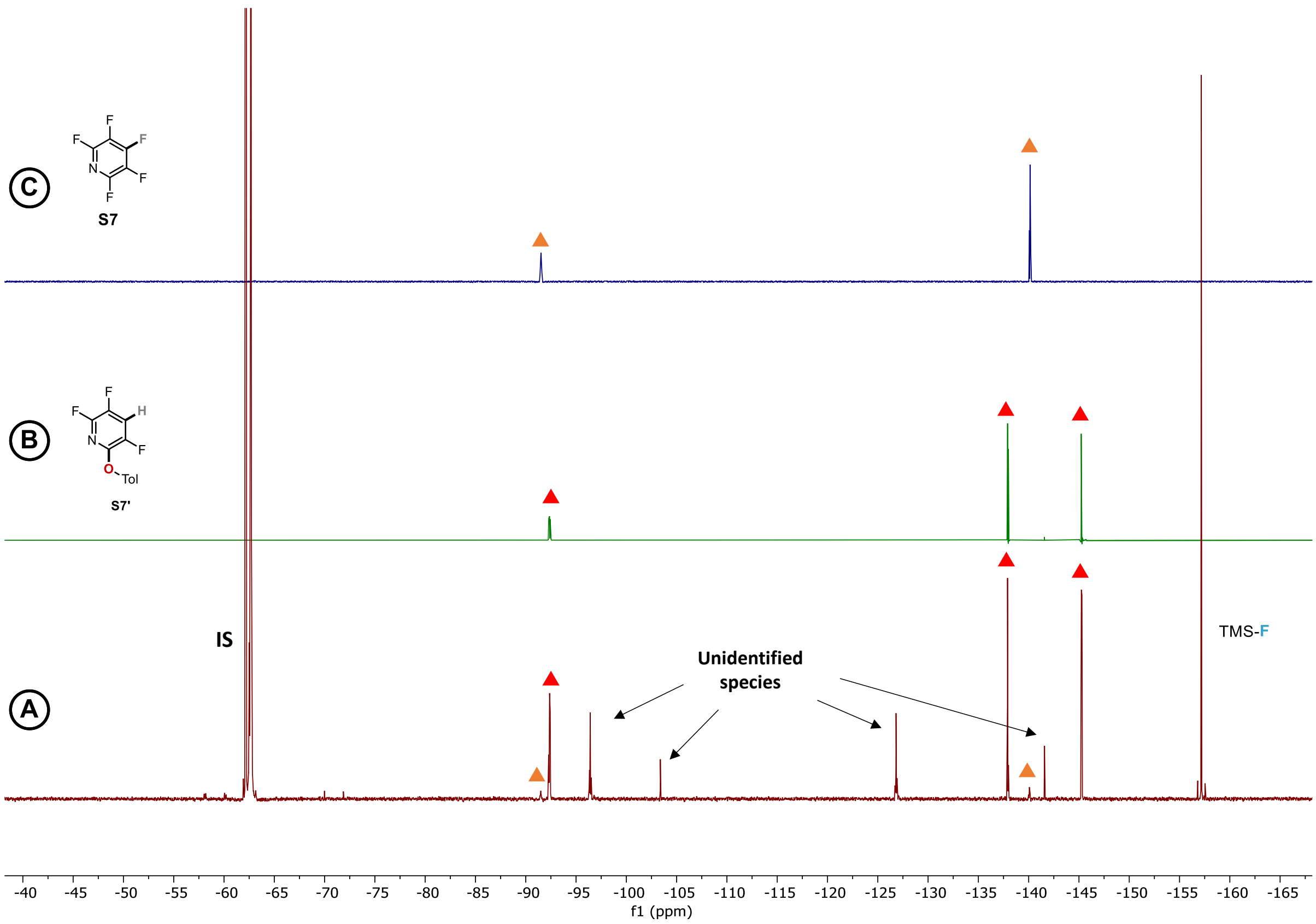

Figure S9. Stacked ^{19}F NMR spectra of (**A**) reaction mixture of **11** with trimethyl(*p*-tolyloxy)silane **S1a**, TASF, and 1,3,5-trimethoxybenzene and α,α,α-trifluorotoluene as IS; (**B**) alkoxylated pyridine **S7'** (red triangles); and (**C**) 2,3,5,6-tetrafluoropyridine **S7** (orange triangles). All the spectra were recorded in C_6D_6.

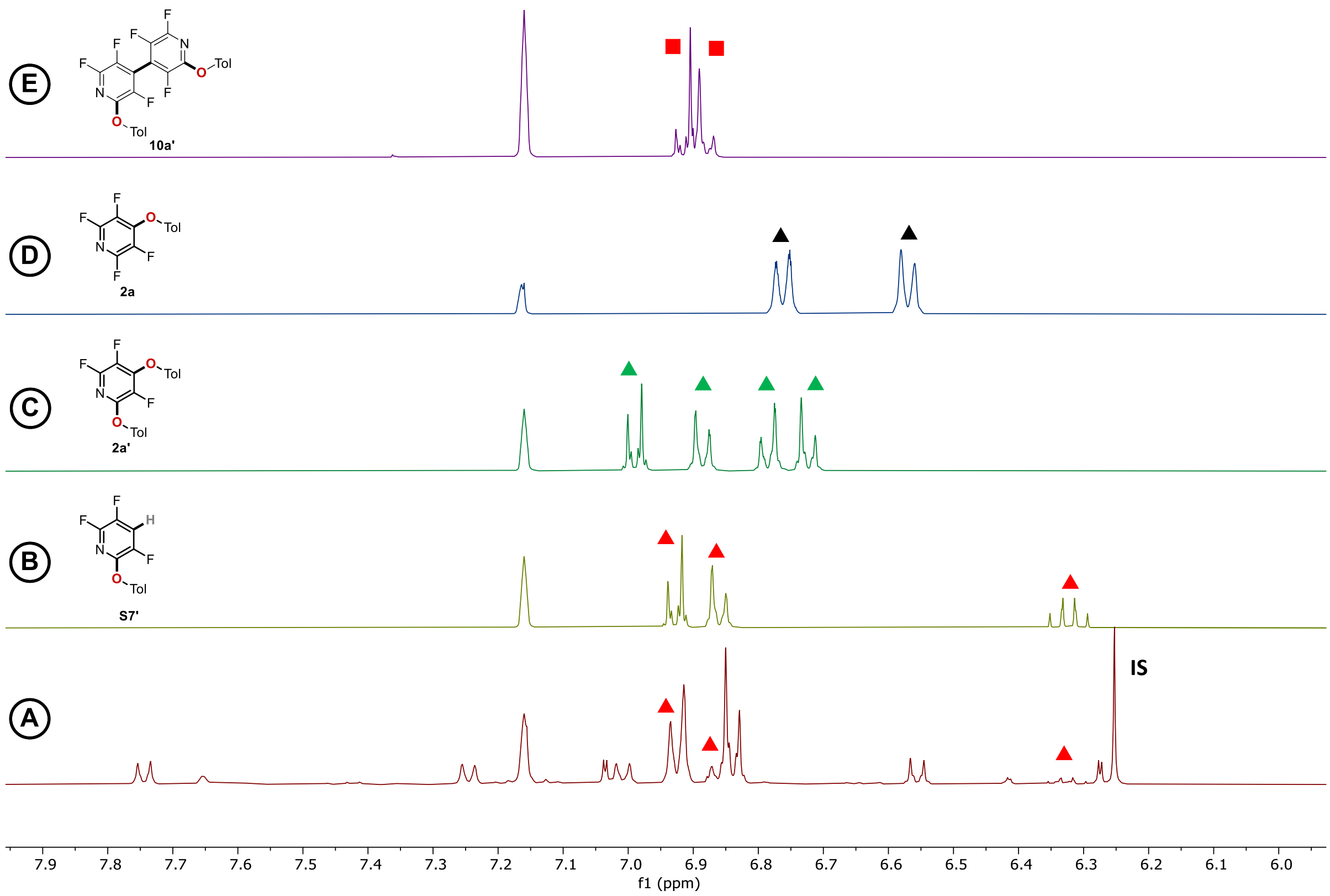

Figure S10. Stacked ^{1}H NMR spectra (aromatic region) to identify plausible major products: (**A**) reaction mixture of **11** with trimethyl(*p*-tolyloxy)silane **S1a**, TASF, and 1,3,5-trimethoxybenzene and α,α,α-trifluorotoluene as IS; (**B**) alkoxylated pyridine **S7'** (red triangles); and (**C**) product **2a'** (green triangles); (**D**) product **2a** (black triangles); and (**E**) dialkoxylated bipyridine **10a'** (red squares). All the spectra were recorded in C_6D_6

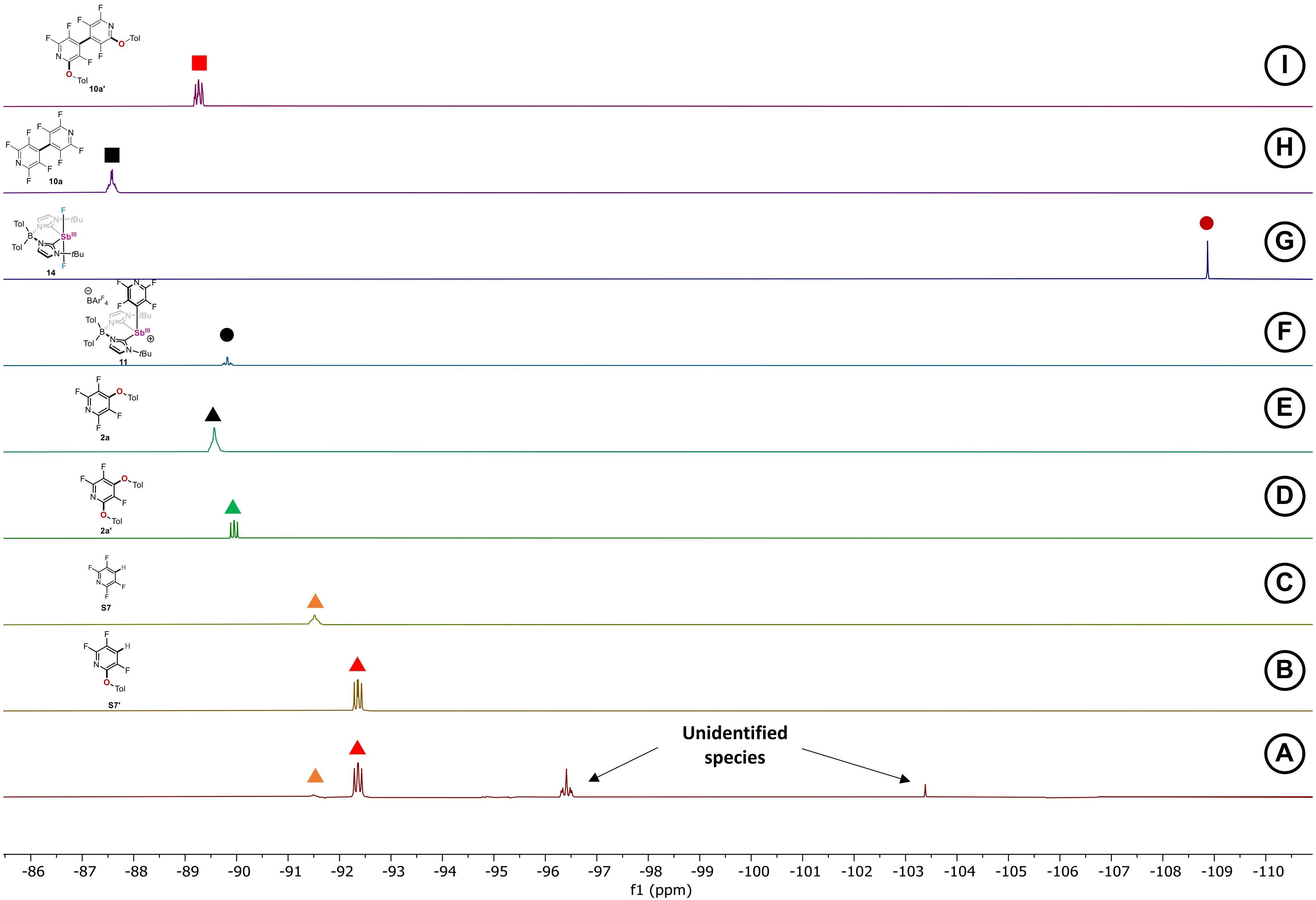

Figure S11. Stacked ^{19}F NMR spectra (-86 to -110 ppm) to identify plausible major products: (**A**) reaction mixture of **11** with trimethyl(p-tolyloxy)silane **S1a**, TASF, and 1,3,5-trimethoxybenzene and α,α,α-trifluorotoluene as IS; (**B**) alkoxylated pyridine **13a'** (red triangles); (**C**) 2,3,5,6-tetrafluoropyridine **13a** (orange triangles); (**D**) product **2a'** (green triangles); (**E**) product **2a** (black triangles); (**F**) Sb(III) complex **11** (black dot); (**G**) Sb(III) difluoride complex **14** (red dot); (**H**) product **10a** (black squares); and (**I**) dialkoxylated bipyridine **10a'** (red squares,). All the spectra were recorded in C_6D_6.

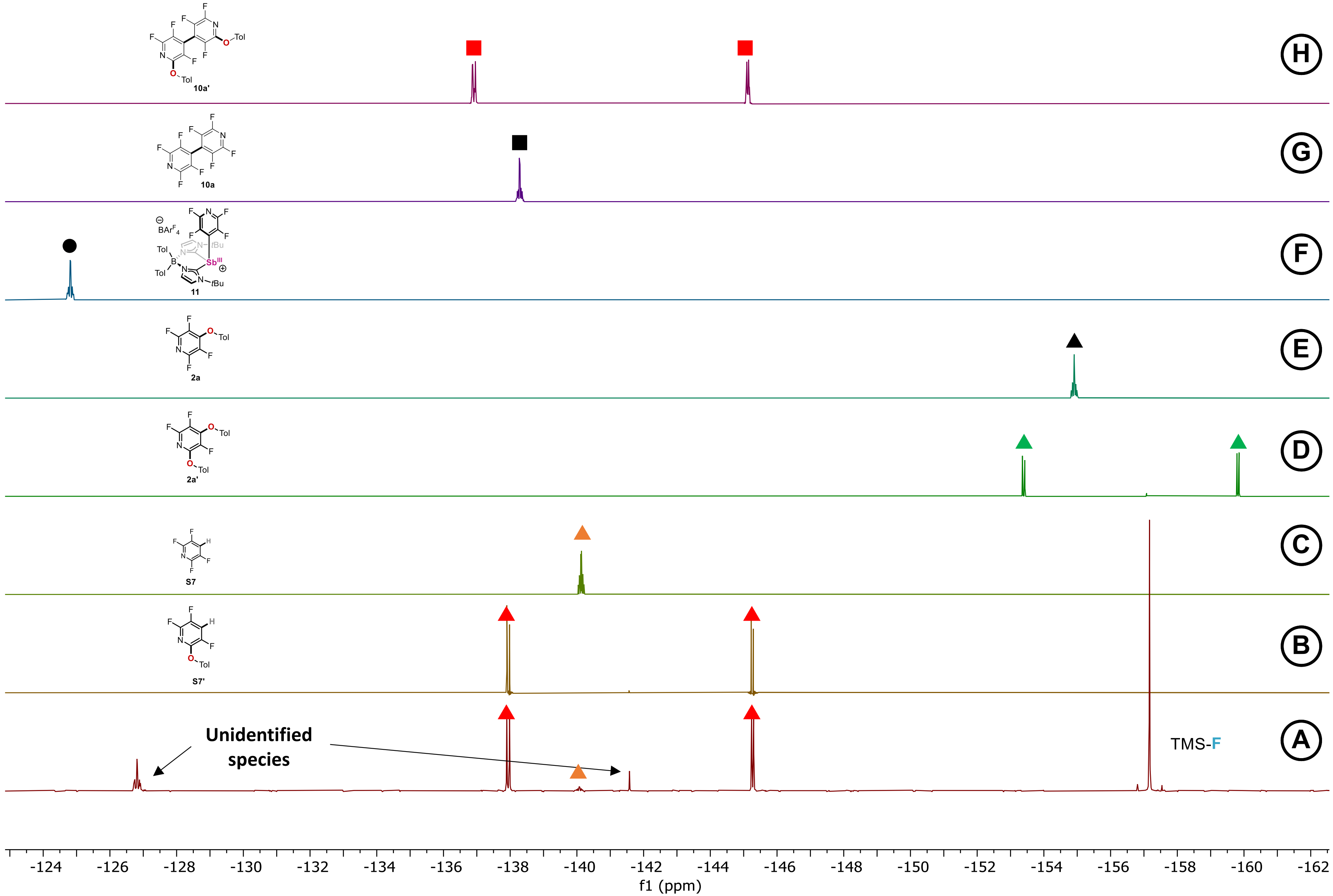

Figure S12. Stacked ^{19}F NMR spectra (-124 to -162 ppm) to identify plausible major products: (**A**) reaction mixture of **11** with trimethyl(p-tolyloxy)silane **S1a**, TASF, and 1,3,5-trimethoxybenzene and α,α,α-trifluorotoluene as IS; (**B**) alkoxylated pyridine **13a'** (red triangles); (**C**) 2,3,5,6-tetrafluoropyridine **13a** (orange triangles); (**D**) product **2a'** (green triangles); (**E**) product **2a** (black triangles,); (**F**) Sb(III) complex **11** (black dot); (**G**) product **10a** (black squares); and (**H**) dialkoxylated bipyridine **10a'** (red squares). All the spectra were recorded in C_6D_6.

5.3.2 Stoichiometric reaction of Sb(III) species **11** with TASF

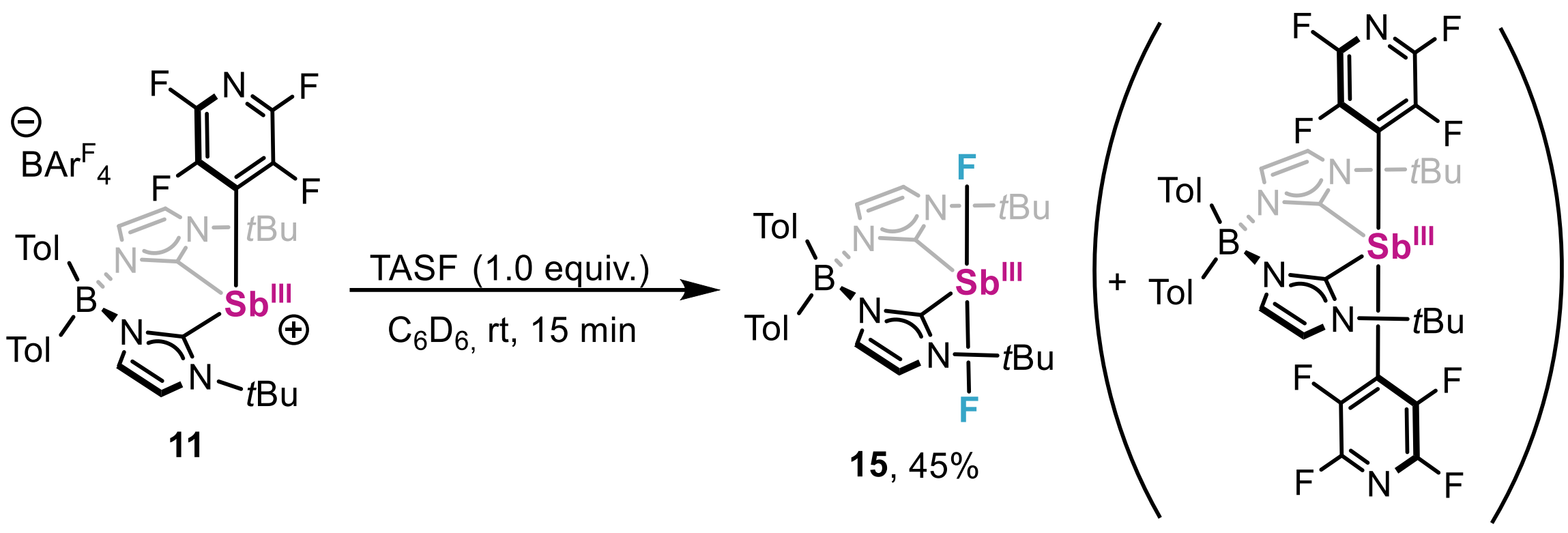

To a solution of Sb(III) complex **11** (39 mg, 1.0 equiv., 0.025 mmol) and 1,3,5-trimethoxybenzene (4.8 mg, 0.66 equiv., 0.0167 mmol) in 0.3 mL of C_6D_6 in a vial was added tris(dimethylamino)sulfonium difluorotrimethylsilicate (7.4 mg, 1.0 equiv., 0.025 mmol) in 0.2 mL of C_6D_6, observing the formation of a yellowish solution with concomitant precipitation of a cream-coloured solid. The resulting mixture was stirred for a further 15 min and transferred to an NMR J-Young tube to be analysed by ^1H NMR (**Figure S13**) and ^{19}F NMR (**Figure S**14), showing the formation of species **15** in 45% yield. We hypothesize that cream-coloured solid unsoluble precipitate corresponds to Sb(III) species **16**, indicating ligand scrambling.

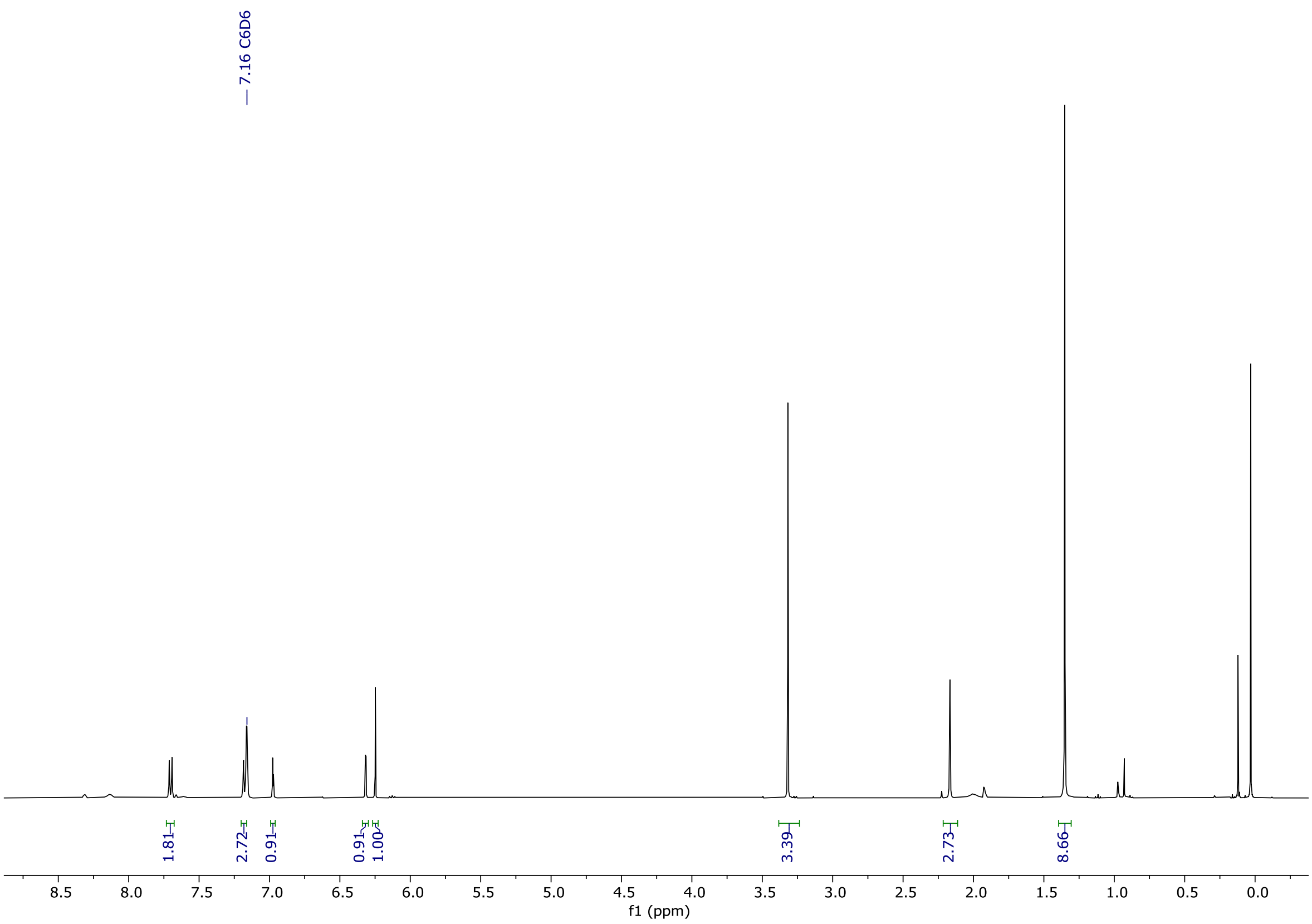

Figure S13. ^1H NMR spectrum of the reaction mixture of **11** with TASF, 1,3,5-trimethoxybenzene and α,α,α-trifluorotoluene after 15 min in C_6D_6.

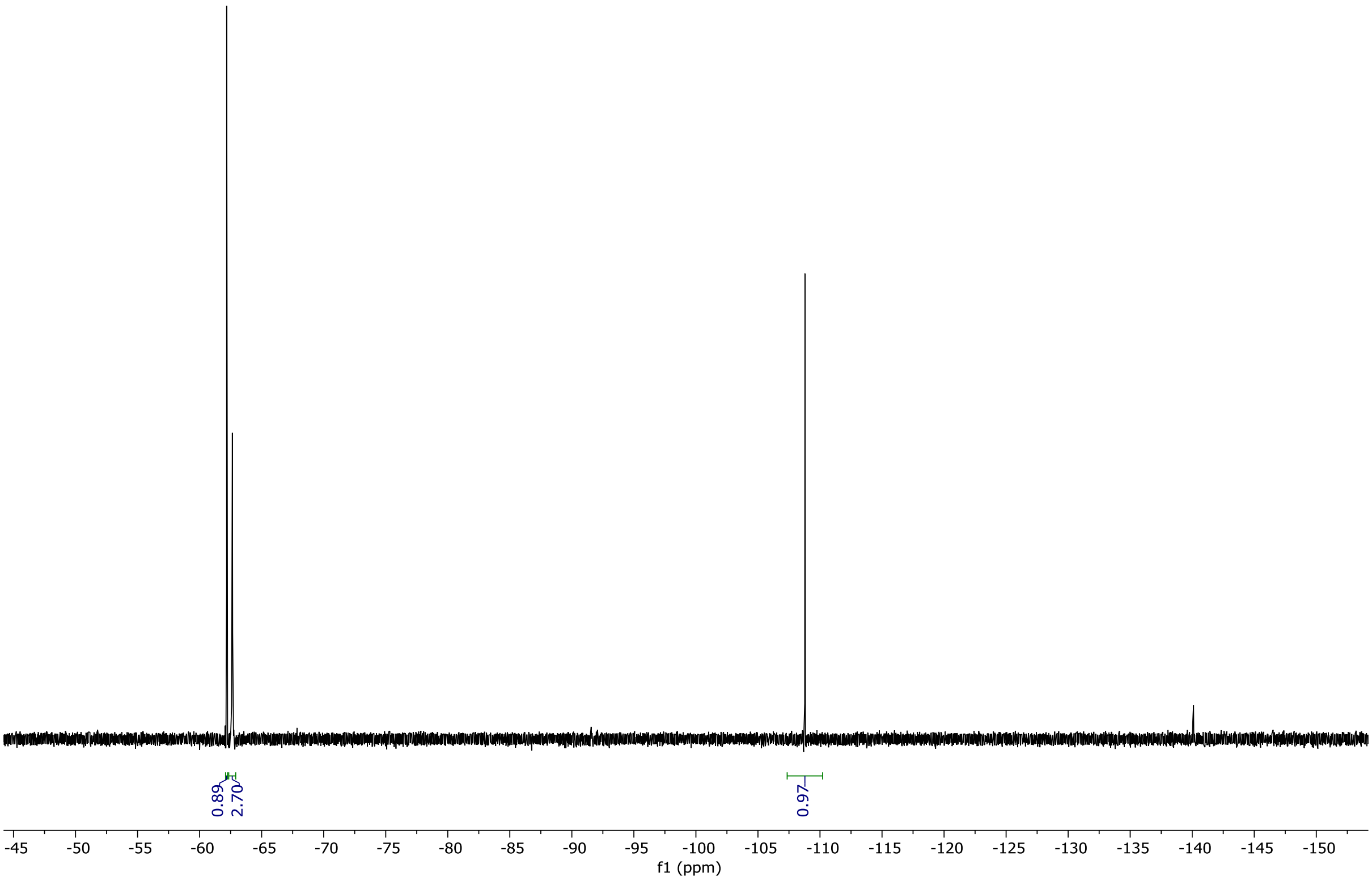

Figure S14. ^{19}F NMR spectrum of the reaction mixture of **11** with TASF, 1,3,5-trimethoxybenzene and α,α,α-trifluorotoluene after 15 min in C_6D_6.

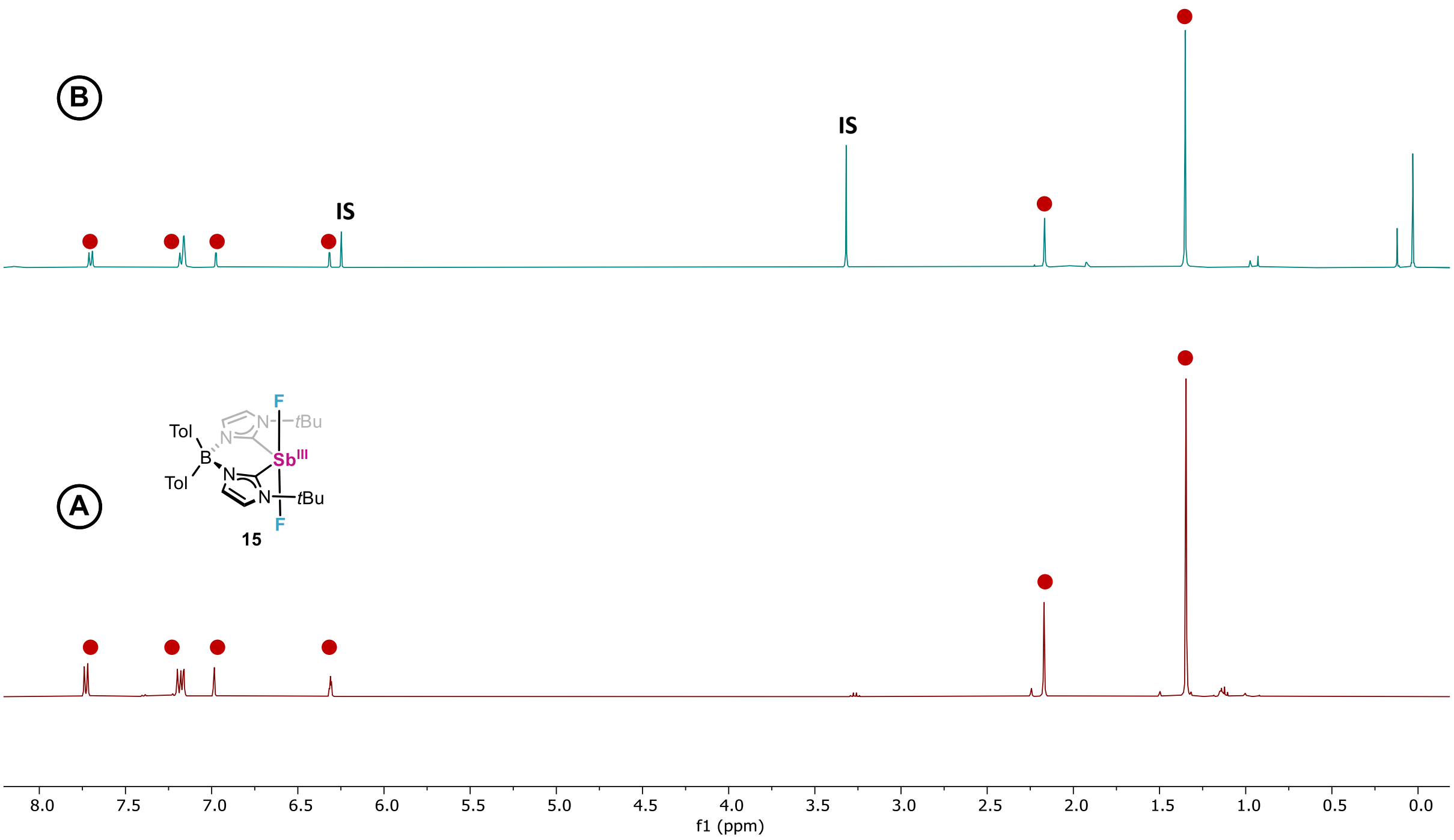

Figure S15. Stacked ^1H NMR spectra of (**A**) Sb(III) difluoride complex **15** (red dots); and (**B**) reaction mixture of **11** with TASF and 1,3,5-trimethoxybenzene as IS (blue spectrum). Both spectra were recorded in C_6D_6.

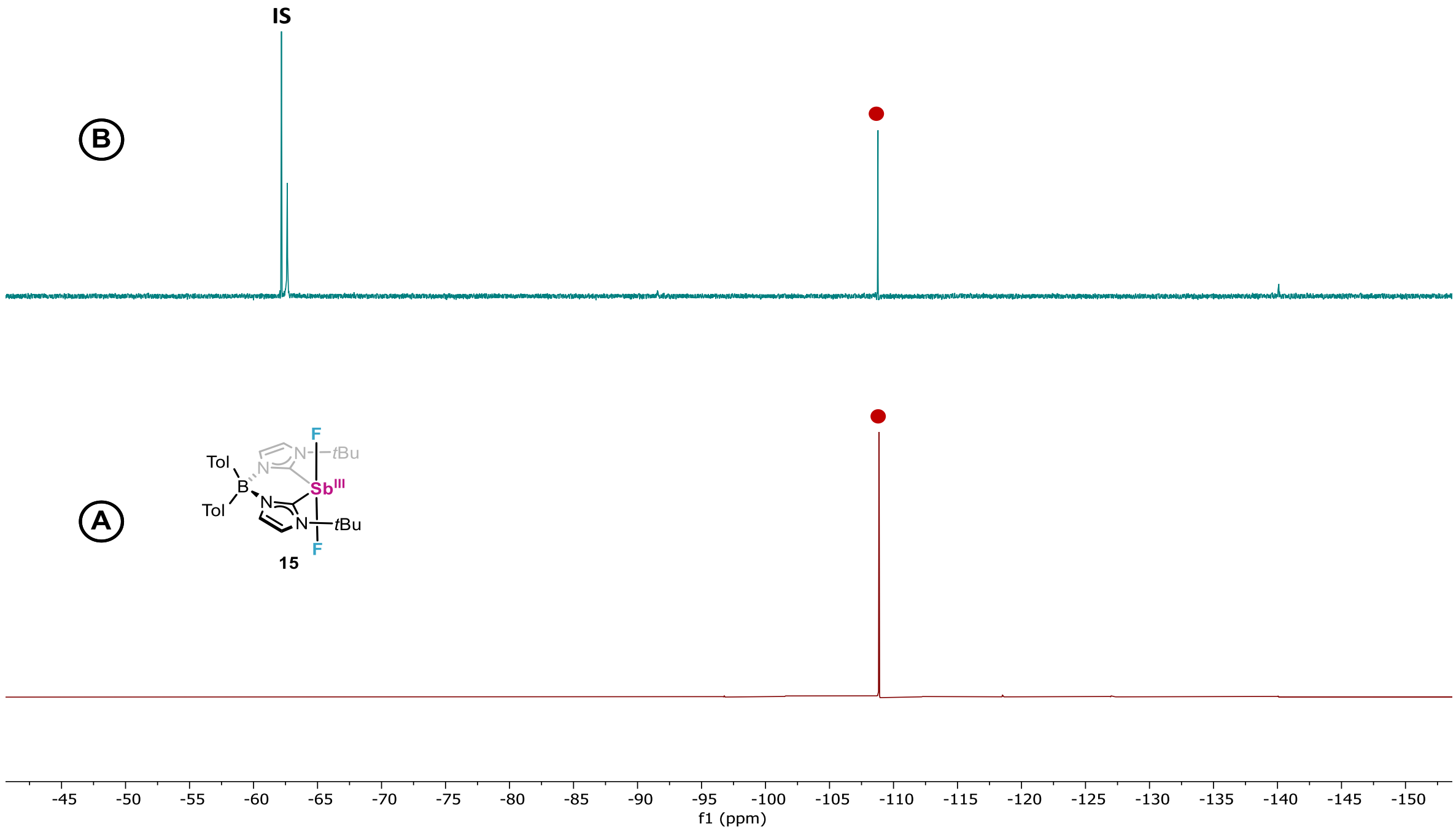

Figure S16. Stacking of the ^{1}H NMR spectra of (**A**) reaction mixture of **11**, TASF and α,α,α-trifluorotoluene as IS, and (**B**) Sb(III) difluoride complex **15** (red dots).

This reaction mixture proved to be unstable under the reaction conditions. In a separate vessel, employing the same experimental procedure but stirring for a longer period of time (30 min to 1 h until the NMR analysis was performed), a mixture of species **15**, free ligand and 2,3,5,6-tetrafluoropyridine **S7** could be observed due to protodemetalation by adventitious water. After 16 h, the amount of **S7** increased to 33%, further highlighting the instability of some of the Ar^{F}-Sb(III) species involved in the ligand scrambling process (**Figure S**17).

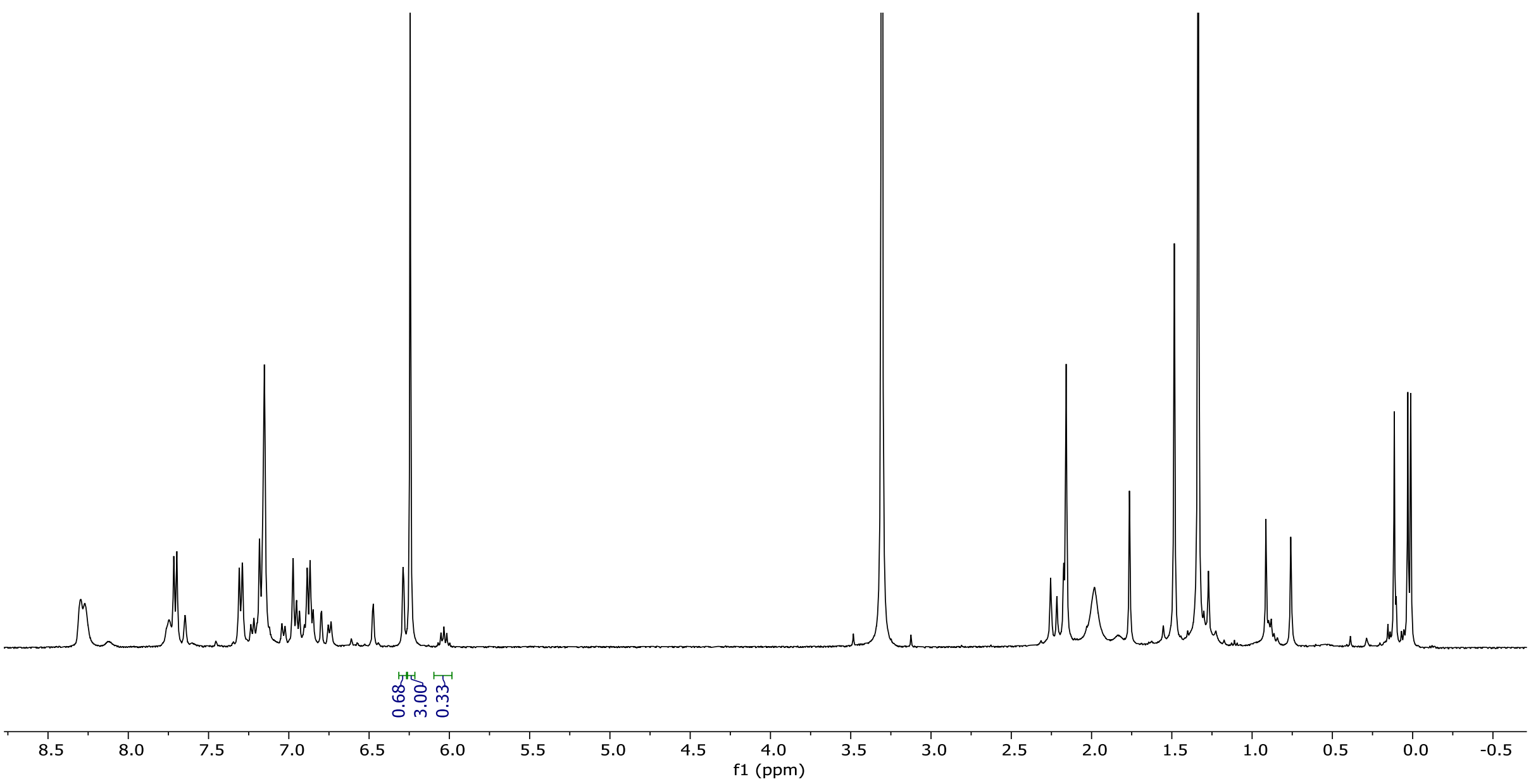

Figure S17. ^{1}H NMR spectrum of the reaction mixture of **11** with TASF, 1,3,5-trimethoxybenzene and α,α,α-trifluorotoluene after 16 h in C_6D_6.

5.4 Attempts on the isolation of species 16

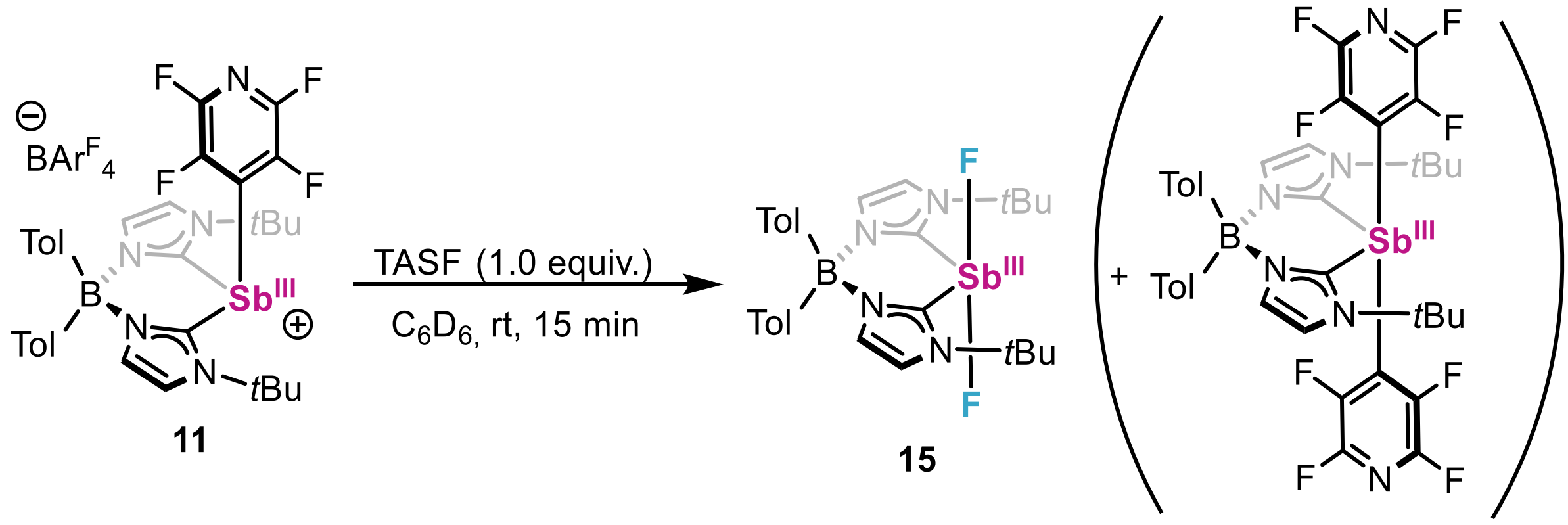

To a solution of Sb(III) complex **11** (39 mg, 1.0 equiv., 0.025 mmol) and 1,3,5-trimethoxybenzene (4.8 mg, 0.66 equiv., 0.0167 mmol) in 0.3 mL of C_6H_6 in a vial was added tris(dimethylamino)sulfonium difluorotrimethylsilicate (7.4 mg, 1.0 equiv., 0.025 mmol) in 0.2 mL of C_6H_6, observing the formation of a yellowish solution with concomitant precipitation of a cream-coloured solid. The supernatant was removed and the solid residue was washed with C_6H_6 (2 x 0.5 mL) and then dried under vacuum.

The solid sample was analyzed by HRMS in the solid state using ASAP as the ionization method. However, no molecular peak of species **16** was observed, and the only peak that could be identified corresponded to the protonated bis-NHC borate ligand. Dissolving the solid in cold acetonitrile or THF to perform ASAP-HESI ionization afforded similar results.

A similar sample was suspended in CD_3CN at -35 ºC (no full dissolution of the solid was observed) and then transferred to a J-Young NMR tube. The sample was then frozen in liquid nitrogen and transferred to an AV400 NMR apparatus calibrated at -40 ºC. No appreciable signals of species **16** or other fluorinated species could be observed at that temperature, probably indicating low insolubility of species **16**. Increasing the temperature to -20 ºC or 0 ºC exhibited no better results, observing only decomposition to free bis-NHC borate ligand and 2,3,5,6-tetrafluoropyridine **S7** as in the stoichiometric experiment depicted in section 5.3.1.

In a similar way, the reaction between species **11** and TASF was directly performed in CD_3CN at -35 ºC, observing immediate precipitation of an off-white precipitate from the initially colorless solution. The suspension was transferred to a J-Young NMR tube and analyzed as described in the previous paragraph. Once again, no signal attributed to species **16** could be observed at low temperatures, but adventitious 2,3,5,6-tetrafluoropyridine and protonated bisNHC-borate ligand were identified (Figure S18 and Figure S19). Also, although the ^1H NMR signals are not too clear due to the overlap with protonated bisNHC-borate ligand, a signal that might be attributed to species **15** was observed by ^{19}F NMR. Warming up to -20 ºC or 0 ºC afforded similar results. However, formation of bipyridine **10a** was observed after 5 min

when the sample was warmed to rt, although with concomitant full decomposition of the previously observed Sb(III)-F species **15** and the presumably formed Sb(I) complex **1a** to protonated bis-NHC borate ligand (Figure S20 and Figure S21).

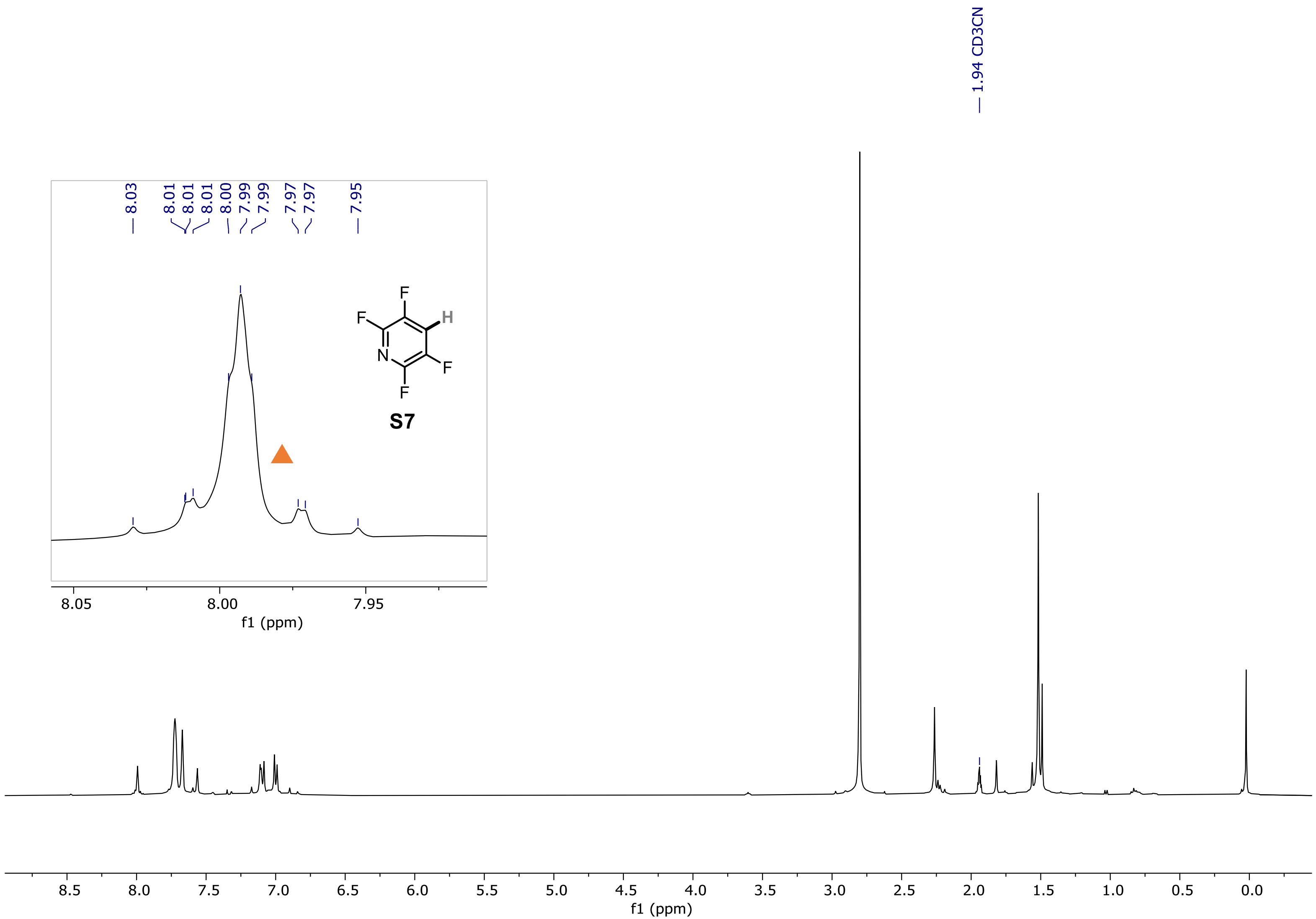

Figure S18: ^{1}H NMR spectrum of the reaction mixture of **11** with TASF at -40 ºC in CD_3CN. Major aromatic species corresponded to protonated bis-NHC borate ligand and species containing BAr^{F}_4 anion. Species **S7** was also identified in the mixture (orange triangle).

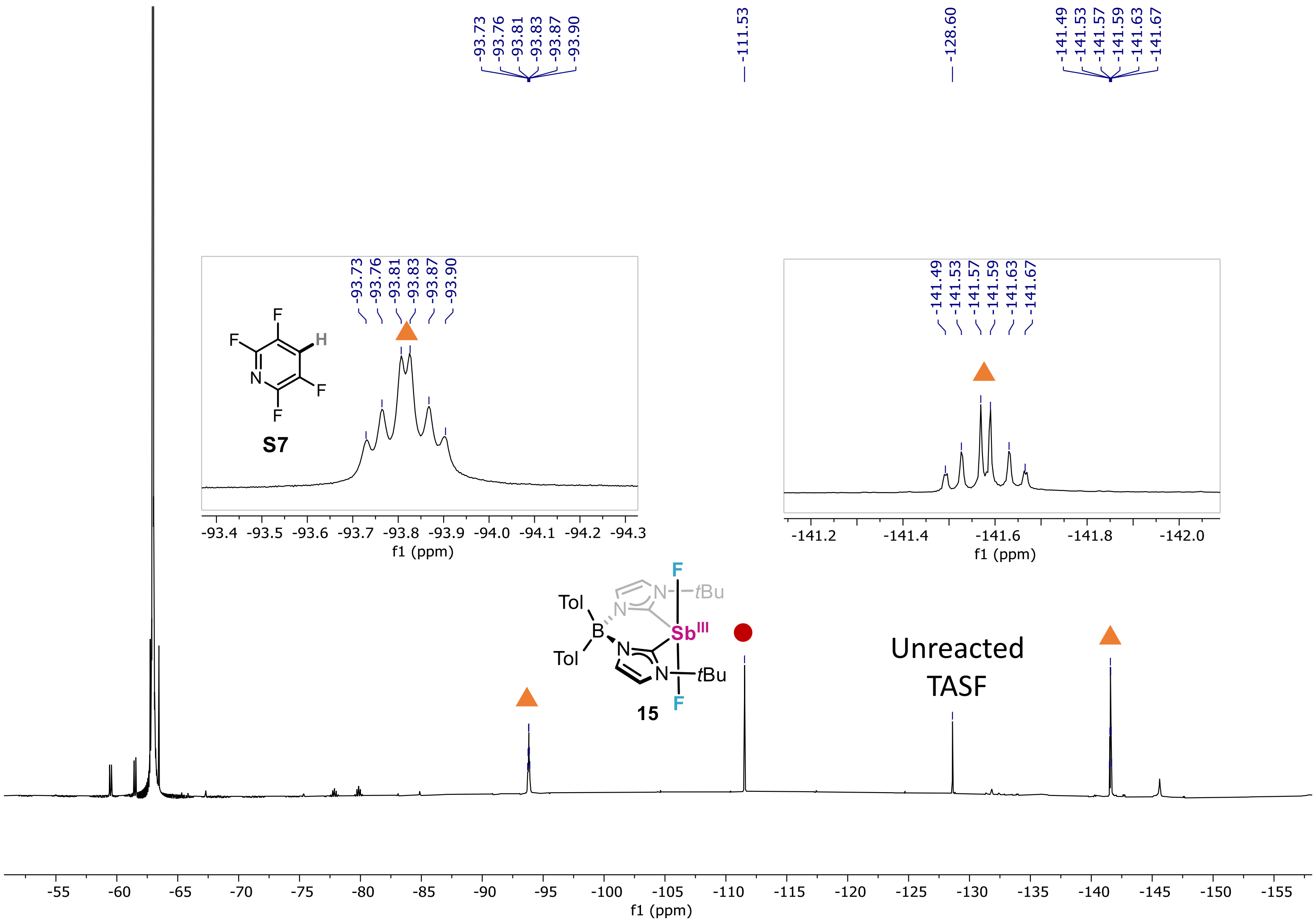

Figure S19: ^{19}F NMR spectrum of the reaction mixture of **11** with TASF at -40 ºC in CD_3CN. Species **S7** (orange triangles), unreacted TASF and potentially species **15** (red circle) were identified in the mixture. The major peak corresponds to species containing $BAr^F{}_4$ anion.

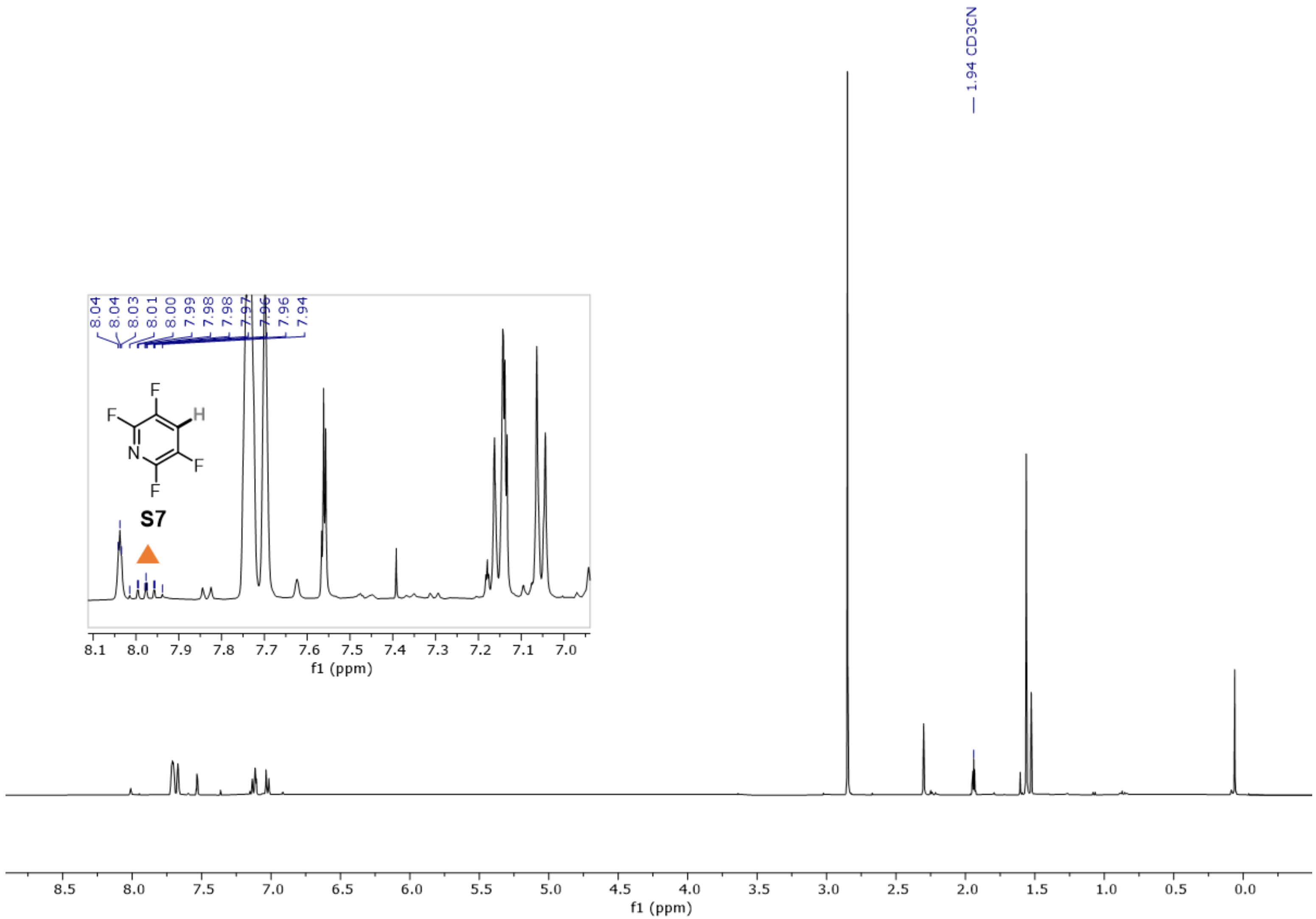

Figure S20: ^{1}H NMR spectrum of the reaction mixture of **11** with TASF in CD_3CN after warming to rt. Major aromatic species corresponded to protonated bis-NHC borate ligand and species containing $BAr^F{}_4$ anion. Species **S7** was also identified in the mixture (orange triangle).

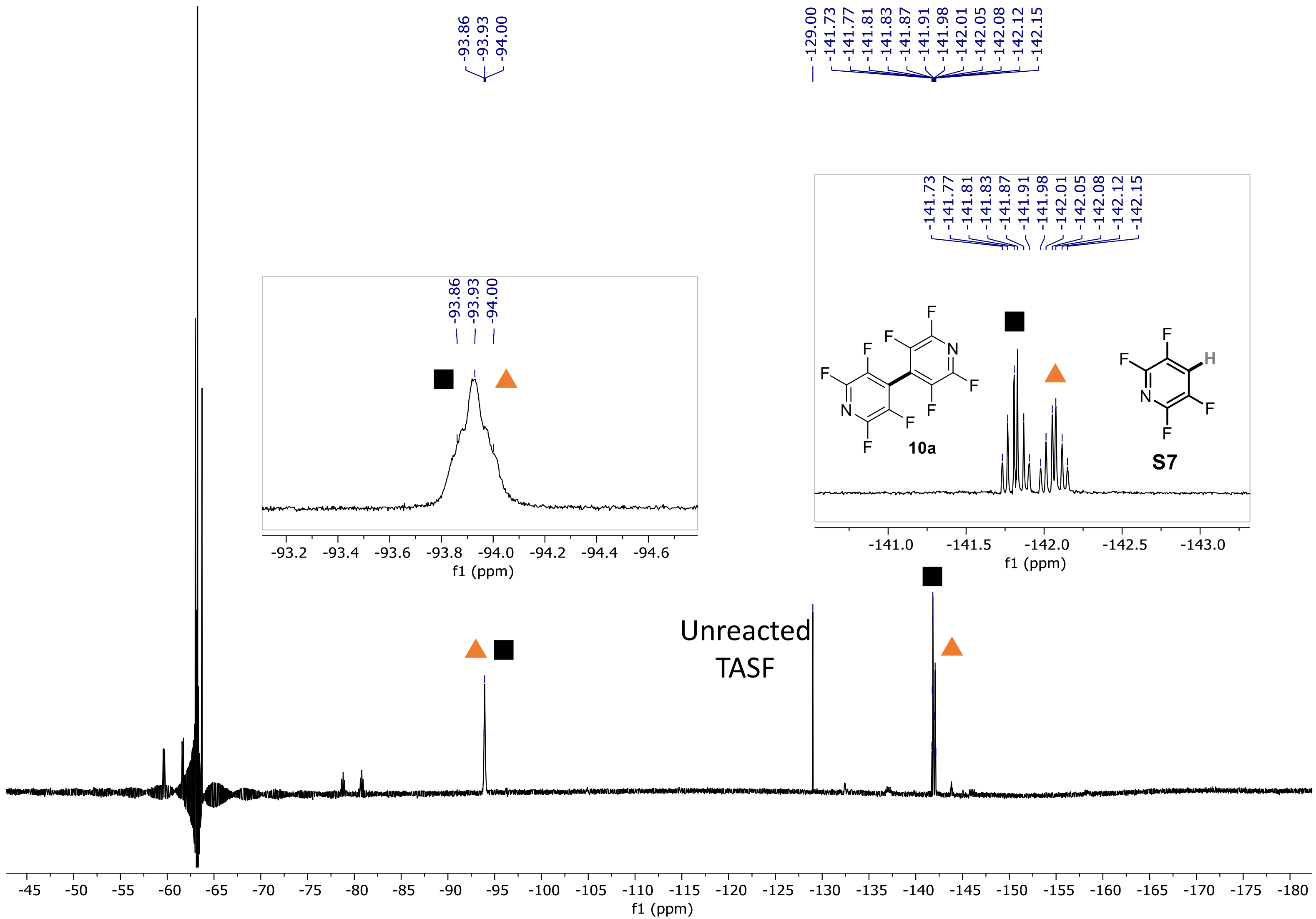

Figure S21: ^{19}F NMR spectrum of the reaction mixture of **11** with TASF in CD_3CN after warming to rt. Species **S7** (orange triangles), unreacted TASF and species **10a** (black squares) were identified in the mixture. The major peak corresponds to species containing BAr^F_4 anion.

5.5 Stoichiometric experiments and synthesis of Sb(III) relevant species

5.5.1 Reaction of **1a** with pentafluoropyridine in acetonitrile. Synthesis of Sb(III) difluoride **15**

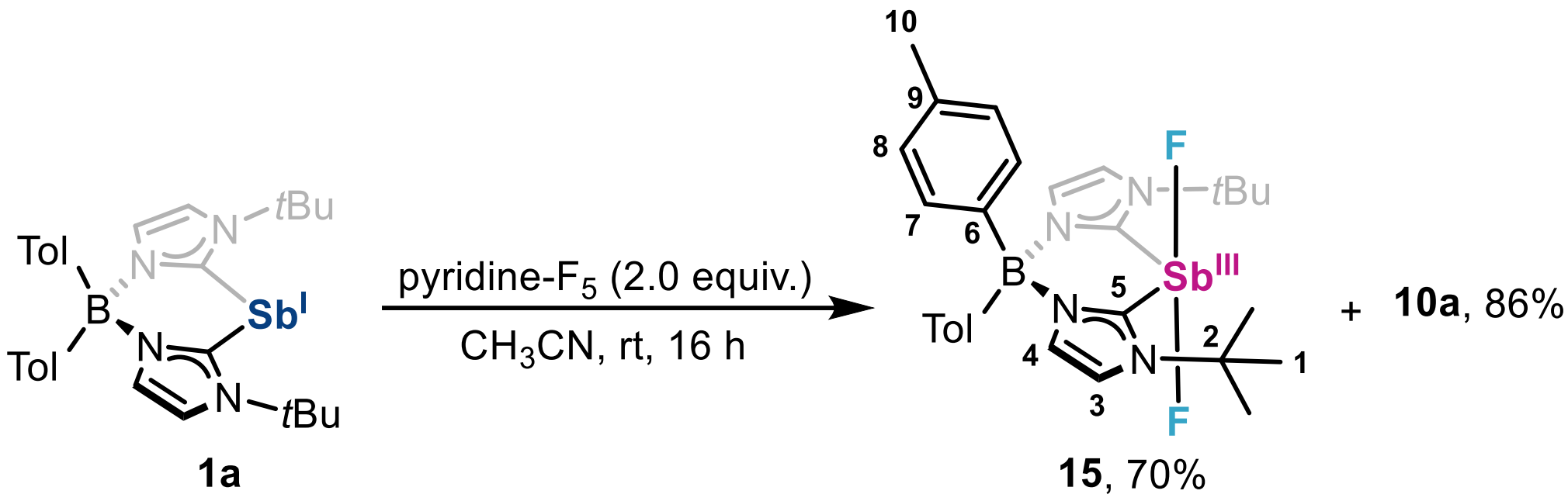

To a suspension of Sb(I) complex **1** (112 mg, 1.0 equiv., 0.200 mmol) in acetonitrile (6 mL), pentafluoropyridine (44 μL, 2.0 equiv., 0.400 mmol) and the mixture was stirred at room temperature, resulting in the formation of an off-white precipitate within minutes. The bright yellow-orangish slurry was stirred for another 16 h to afford a clear pale orange solution. After removal of the solvent under vacuum, the crude solid was washed with diethylether (10 mL) and then dried under vacuum to afford **15** as an off-white solid in 70% yield (84 mg, 0.140 mmol). Evaporation of the diethylether and short filtration of the supernatant through an SiO_2 plug with DCM afforded compound **10a** as a pale orange solid in 86% yield (52 mg, 0.17 mmol).

^{1}H NMR (400 MHz, C_6D_6) δ = 7.73 (d, $J_{H\text{-}H}$ = 8.1 Hz, 4H, H-7), 7.19 (dd, $J_{H\text{-}H}$ = 8.3, 0.7 Hz, 4H, H-8), 6.99 (d, $J_{H\text{-}H}$ = 1.8 Hz, 2H, H-4), 6.36 – 6.27 (m, 2H, H-3), 2.17 (s,6H, H-10), 1.34 (s, 18H, H-1).

^{13}C{^{1}H} NMR (101 MHz, C_6D_6) δ = 162.2 (t, $J_{C\text{-}F}$ = 16.1 Hz, 2 x C, C-5), 146.8 (2 x C, C-6), 135.8 (2 x C, C-9), 134.6 (4 x CH, C-8), 128.7 (4 x CH, C-7), 125.4 (2 x CH, C-4), 117.0 (2 x CH, C-3), 58.6 (2 x C, C-1), 31.7 (6 x CH_3, C-1), 21.3 (2 x CH3, C-10).

^{11}B NMR (128 MHz, C_6D_6) δ = 3.11 (bs)

^{19}F{^{1}H} NMR (377 MHz, C_6D_6) δ = -108.87 (s).

HRMS (ESI-pos, m/z): calc'd for $C_{28}H_{36}BF_2LiN_4Sb$ $[M+Li]^+$ 605.2199; found 605.2137.

5.5.2 Reaction of **15** with 1,4-bis(trimethylsilyl)-1,4-dihydropyrazine **S6** in acetonitrile

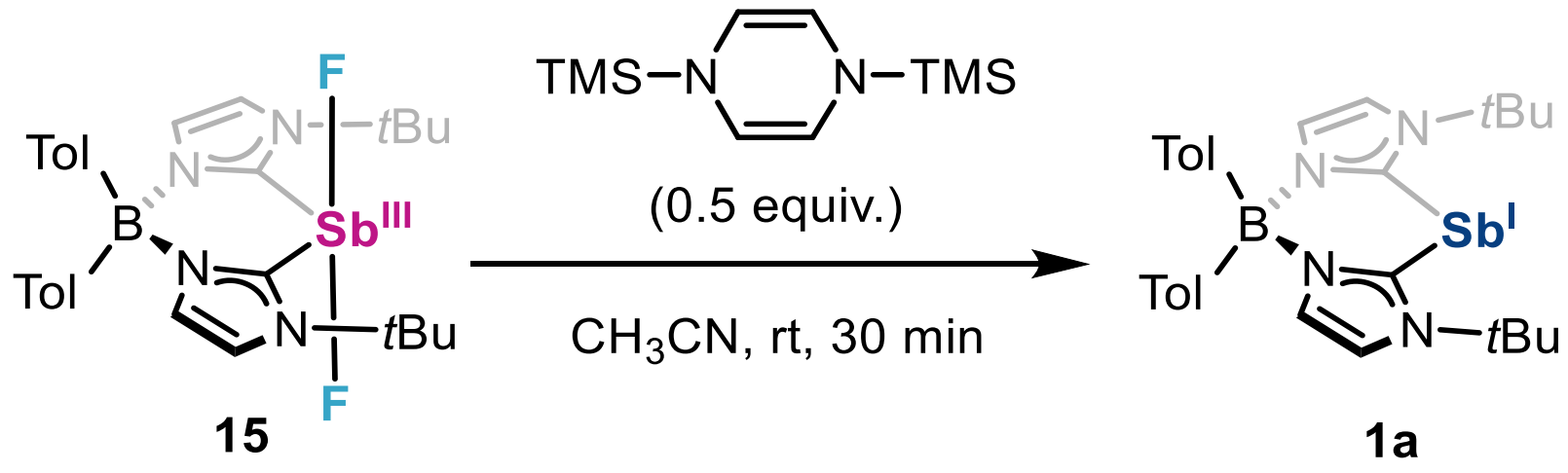

To a solution of Sb(III) difluoride complex **15** (12 mg, 1.0 equiv., 0.020 mmol) and 1,3,5-trimethoxybenzene (6.8 mg, 2.0 equiv., 0.040 mmol) in acetonitrile (0.5 mL), a solution of 1,4-bis(trimethylsilyl)-1,4-dihydropyrazine (5 mg, 1.1 equiv., 0.022 mmol) in acetonitrile (0.5 mL) was added at room temperature. After stirring for 30 min, C_6D_6 (0.3 mL) was added and the crude mixture was analysed by ^{1}H (**Figure S**22) and ^{19}F NMR (**Figure S**23). The observed NMR signals matched those reported for stibinidene complex **1a** indicating its formation in 93% yield. The ^{19}F NMR exhibited only a signal corresponding to TMSF, indicating quantitative reduction of Sb(III) difluoride species **15**.

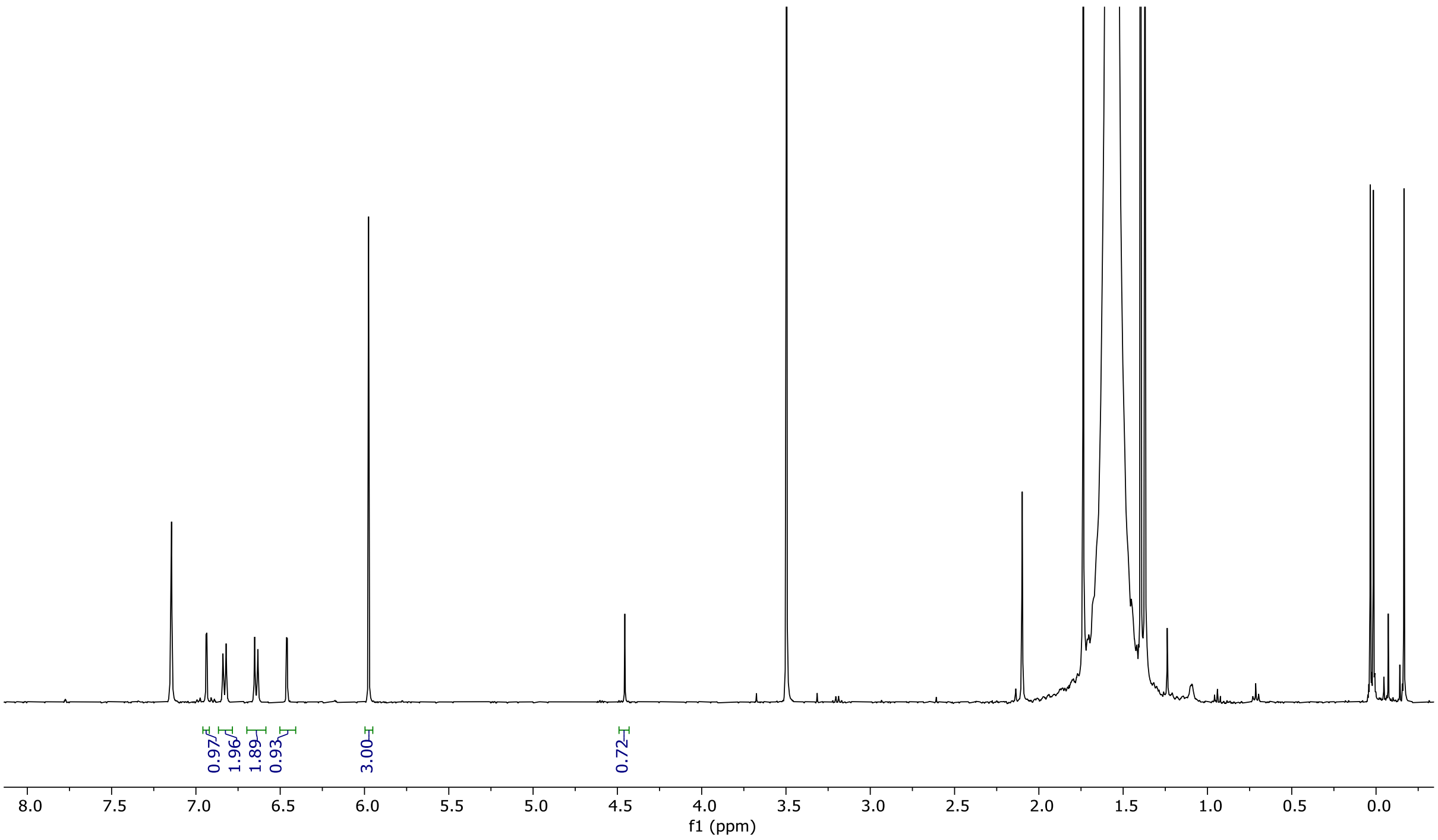

Figure S22. ^{1}H NMR spectrum of the reaction mixture of **15** with **S6** and 1,3,5-trimethoxybenzene as IS in acetonitrile. C_6D_6 was used as solvent to run the NMR sample.

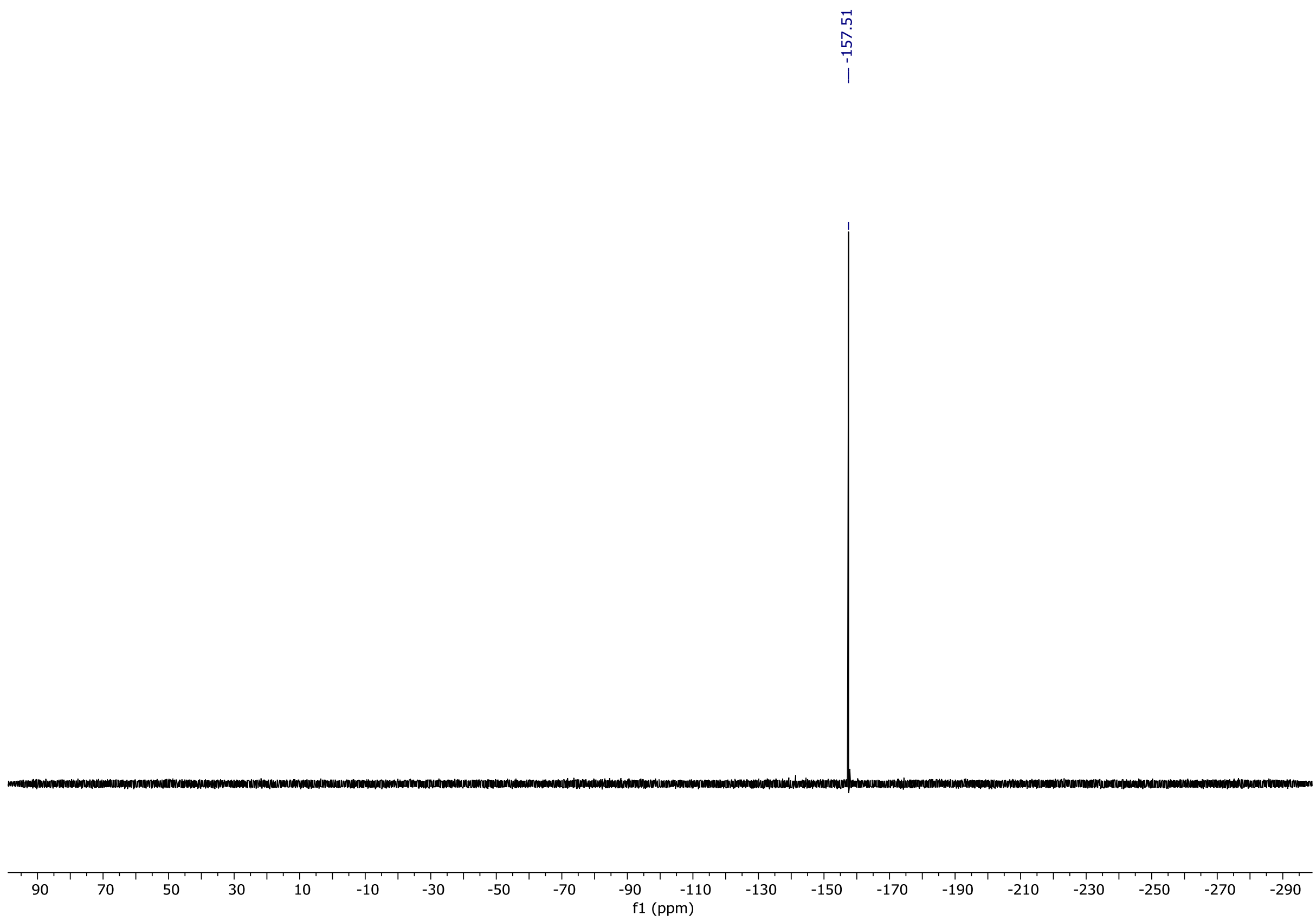

Figure S23. ^{19}F NMR spectrum of the reaction mixture of **15** with **S6** and 1,3,5-trimethoxybenzene as in acetonitrile. C_6D_6 was used as solvent to run the NMR sample.

5.5.3 Synthesis of Sb(III) dialkoxy complex **14** from Sb(III) dichloride complex **4**

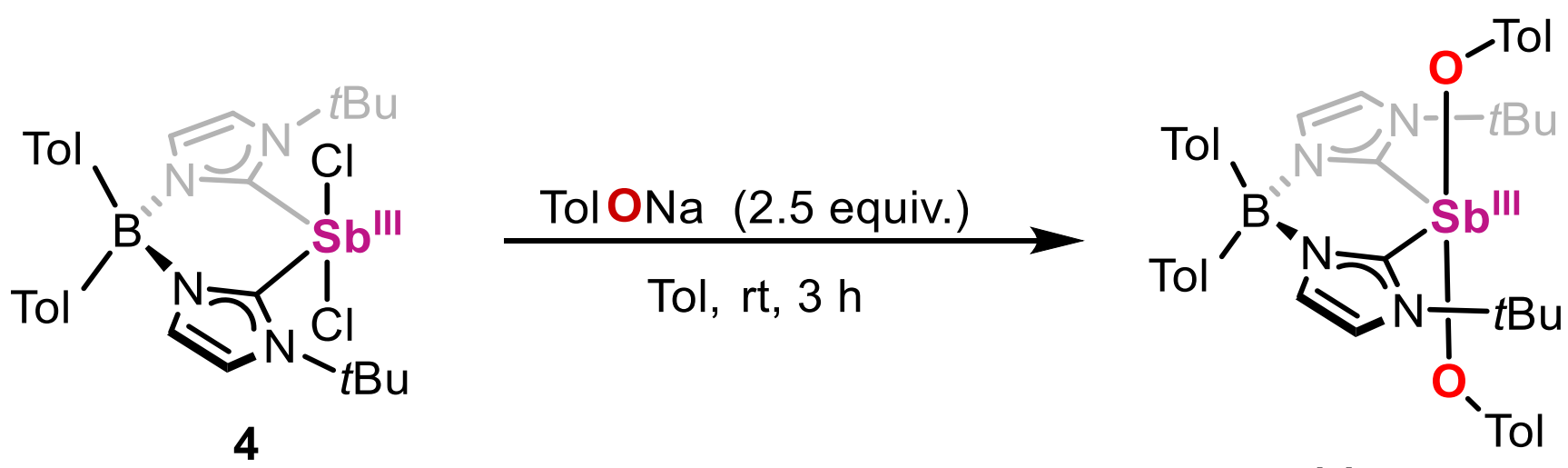

To a suspension of Sb(III) complex **4** (474 mg, 1.0 equiv., 0.750 mmol) in toluene (10 mL), sodium 4-methylphenolate (197 mg, 2.5 equiv., 1.500 mmol) was added in one portion and the mixture was stirred at room temperature, resulting in the formation of an off-white precipitate and a cream-coloured solution. The slurry was filtered through a small plug of celite to afford a clear cream-coloured solution. Removal of the solvent under vacuum afforded complex **14** as a cream-coloured solid in 74% yield (430 mg, 0.56 mmol).

It was found that complex **14** decomposed both in solid state and in C_6D_6 solution at room temperature over time generating Sb black and free bis-NHC borate ligand. A ^{13}C NMR spectrum recorded in a J-young NMR tube 1 h after the sample preparation exhibited small

signals corresponding to the free ligand, and after 16 h the sample showed an appreciable amount of free ligand in ^{1}H NMR.

^{1}H NMR (400 MHz, C_6D_6) δ = 7.75 (d, $J_{H\text{-}H}$ = 7.9 Hz, 4H, H-7), 7.25 (d, $J_{H\text{-}H}$ = 7.8 Hz, 4H, H-8), 7.04 (d, $J_{H\text{-}H}$ = 2.0 Hz, 2H, H-4), 7.02 (d, $J_{H\text{-}H}$ = 8.3 Hz, 4H, H-13), 6.57 (d, $J_{H\text{-}H}$ = 8.3 Hz, 4H, H-12), 6.28 (d, $J_{H\text{-}H}$ = 2.0 Hz, 2H, H-3), 2.24 (s, 6H, H-10), 2.22 (s, 6H, H-15), 1.19 (s, 18H, H-1).

^{13}C{^{1}H} NMR (101 MHz, C_6D_6) δ = 162.5 (2 x C, C-5), 159.2 (2 x C, C-14), 146.8 (2 x C, C-6), 135.8 (2 x C, C-9), 134.9 (4 x CH, C-8), 130.1 (4 x CH, C-13), 128.6 (4 x CH, C-7), 126.3 (2 x CH, C-4), 125.1 (2 x C, C-14), 120.1 (4 x CH, C-12), 117.3 (2 x CH, C-3), 59.0 (2 x C, C-2), 31.8 (6 x CH_3, C-1), 21.4 (2 x CH_3, C-10), 20.9 (2 x CH_3, C-15).

^{11}B NMR (128 MHz, C_6D_6) δ = -9.49 (bs).

HRMS (ESI-pos, m/z): Due to its instability, HRMS could not be obtained for this compound, and no recognisable patterns could be detected in the analysis.

5.5.4 Reaction of **15** with trimethyl(*p*-tolyloxy)silane **S1a** in C_6D_6

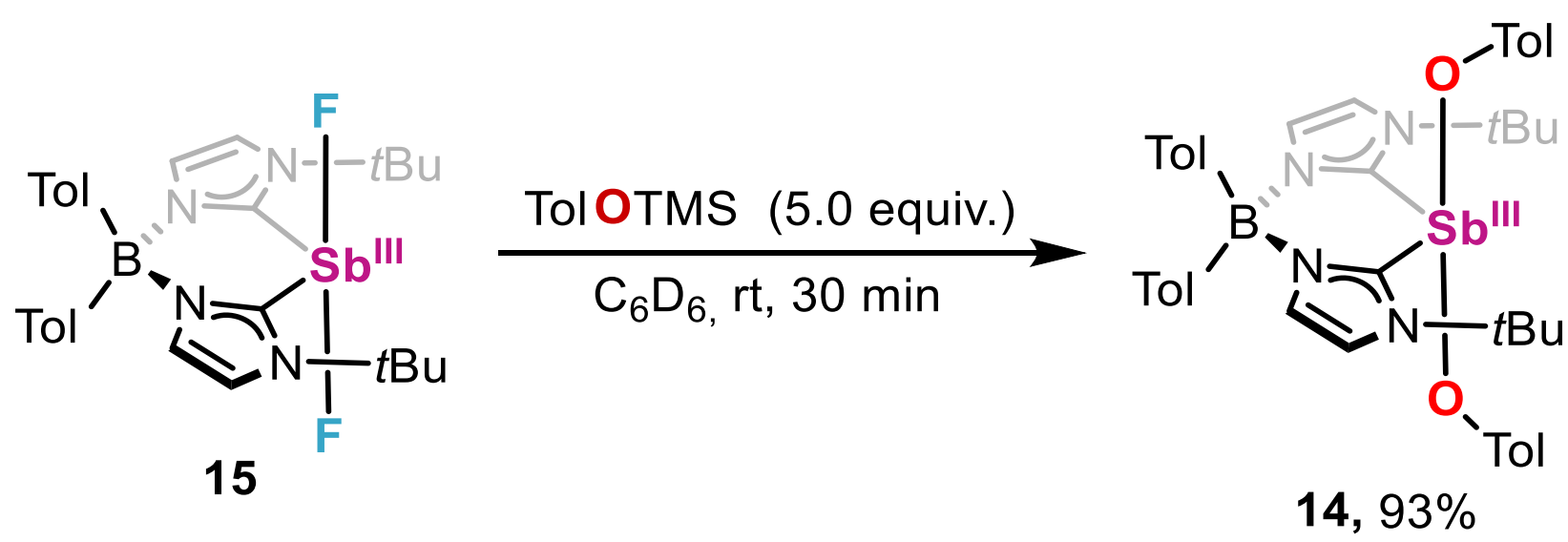

To a solution of Sb(III) difluoride complex **15** (12 mg, 1.0 equiv., 0.020 mmol), 1,3,5-trimethoxybenzene (10 mg, 1.0 equiv., 0.020 mmol) and α,α,α -trifluorotoluene (2.5 μL, 1.0 equiv., 0.020 mmol) in C_6D_6 (0.3 mL), a solution of trimethyl(*p*-tolyloxy)silane **S1a** (18 mg, 5.0 equiv., 0.100 mmol) in C_6D_6 (0.2 mL) was added at room temperature. After stirring for 30 min, the crude mixture was transferred to a J-Young NMR tube and analysed by ^{1}H (**Figure S24**) and ^{19}F NMR (**Figure S25**). The observed ^{1}H NMR signals matched those observed for complex **14**, obtained the synthetic methodology described in section 4.4.4, indicating its formation in 93% yield. Additionally, no signal of the starting Sb(III) difluoride complex **15** was observed in ^{19}F NMR, indicating full conversion, and TMSF was detected in 27% yield.

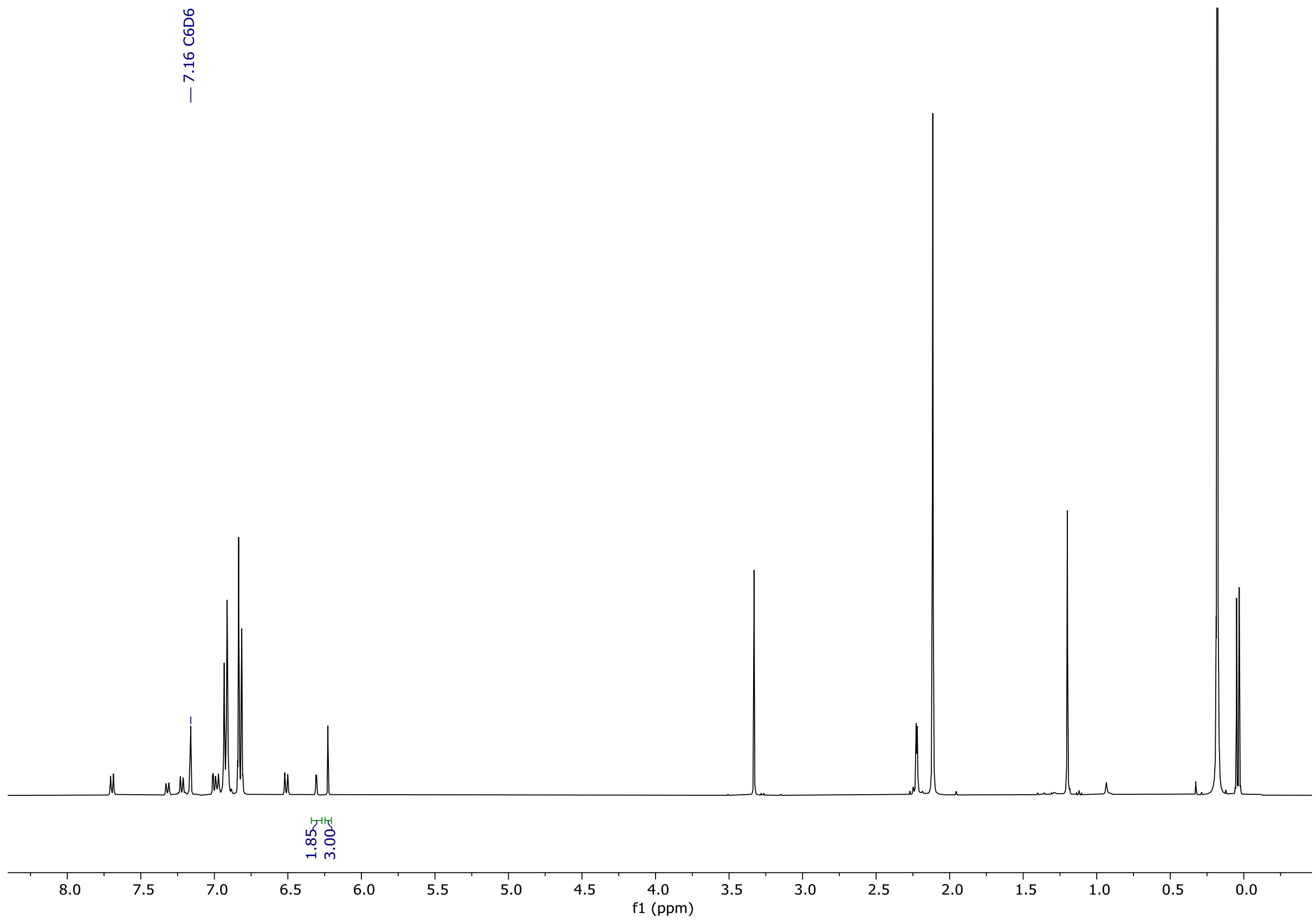

Figure S24. ^{1}H NMR spectrum of the reaction mixture of **15** with trimethyl(*p*-tolyloxy)silane **S1a**, 1,3,5-trimethoxybenzene and α,α,α-trifluorotoluene as IS after 30 min in C_6D_6.

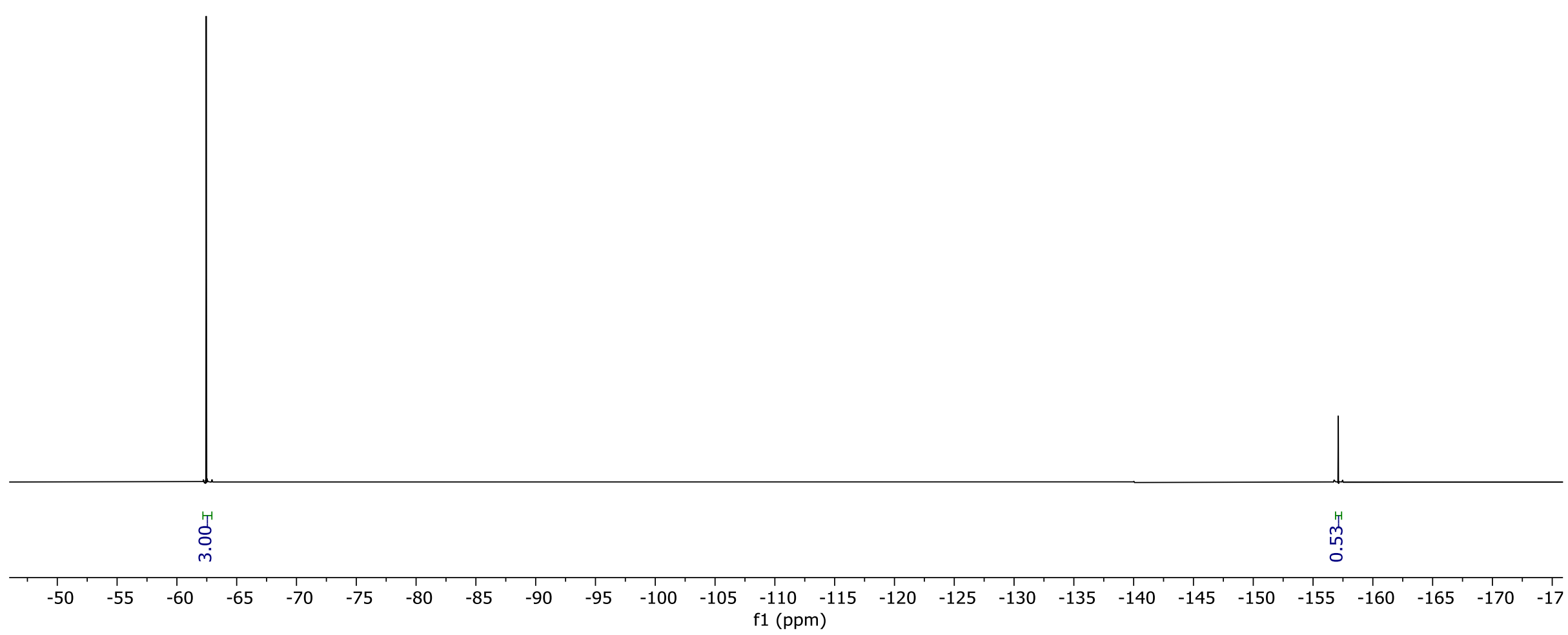

Figure S25. ^{19}F NMR spectrum of the reaction mixture of **15** with trimethyl(*p*-tolyloxy)silane **S1a**, 1,3,5-trimethoxybenzene and α,α,α-trifluorotoluene as IS after 30 min in C_6D_6.

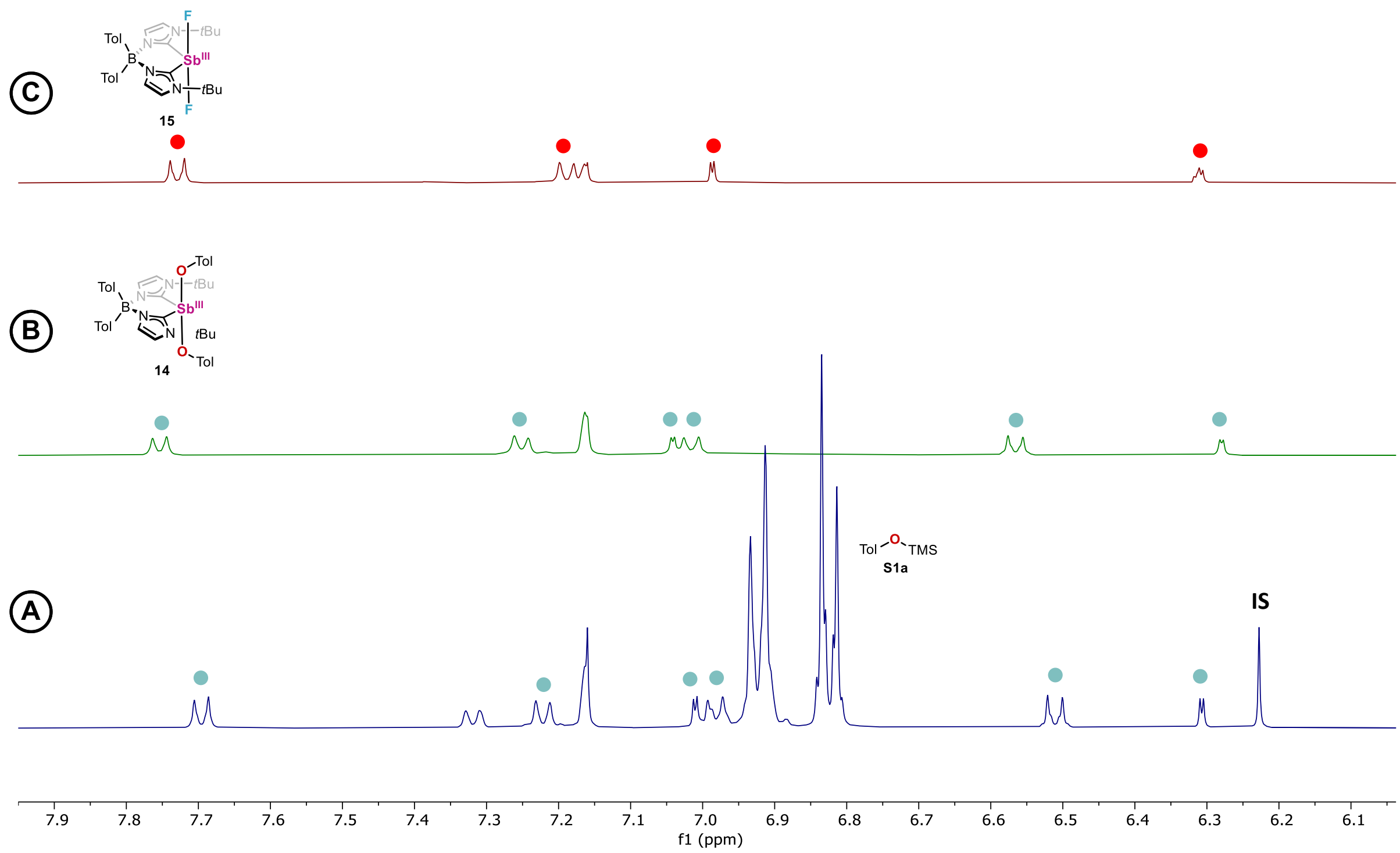

Figure S26. Stacked ^{1}H NMR spectra of (**A**) reaction mixture of **15**, trimethyl(*p*-tolyloxy)silane and α,α,α-trifluorotoluene and 1,3,5-trimethoxybenzene as IS; (**B**) pure **14** (blue dots); and (**C**) pure **15** (red dots).

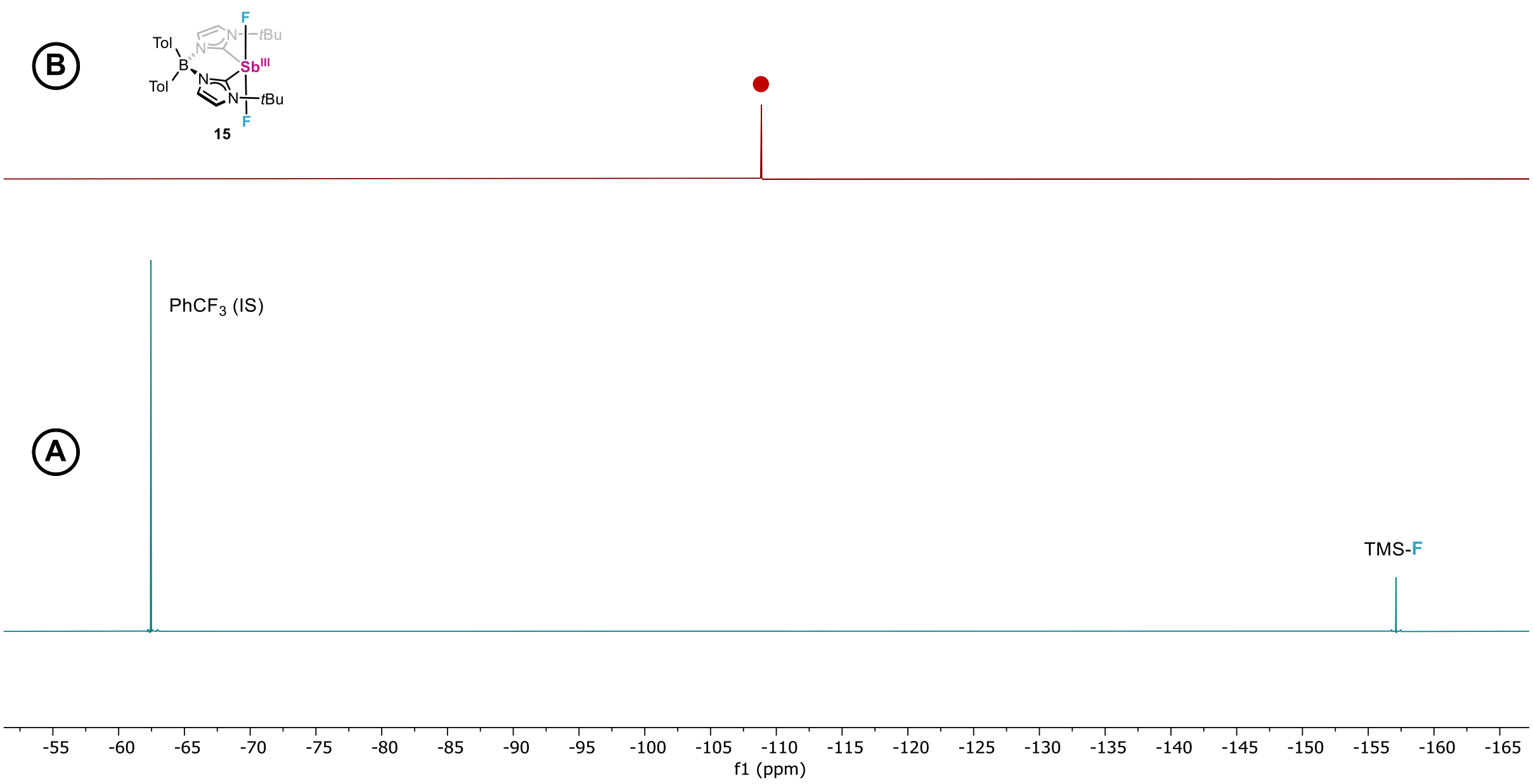

Figure S27. Stacked ^{19}F NMR spectra of (**A**) reaction mixture of **15**, trimethyl(*p*-tolyloxy)silaneand α,α,α-trifluorotoluene and 1,3,5-trimethoxybenzene as IS; and (**B**) pure **15** (red dots).

5.5.5 Synthesis of $Sb(OTol)_3$

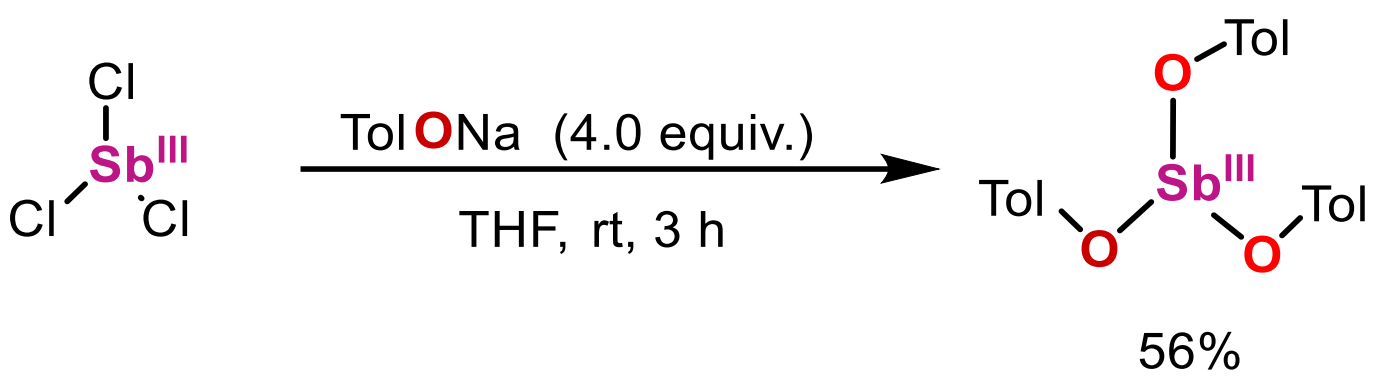

To a solution of $SbCl_3$ (179 mg, 1.0 equiv., 1.00 mmol) in THF (10 mL), sodium 4-methylphenolate (521 mg, 4.0 equiv., 4.00 mmol) was added in one portion and the mixture was stirred at room temperature, resulting in the formation of an off-white precipitate and a colorless solution. The slurry was filtered through a small plug of celite under nitrogen to afford a clear, colorless solution. Removal of the solvent under vacuum afforded $Sb(OTol)_3$ as an off-white solid in 56% yield (248 mg, 0.56 mmol). Note: extremely sensitive to air and moisture.

^{1}H NMR (400 MHz, C_6D_6) δ = 6.78 (bs, 12H, Ar-H), 2.06 (s, 9H, Ar-CH_3).

^{13}C{^{1}H} NMR (101 MHz, C_6D_6) δ = 155.6 (3 x C, C-O), 130.6 (6 x CH), 120.3 (6 x CH), 20.6 (3 x CH_3).

HRMS (ESI-pos, m/z): Due to its instability, HRMS could not be obtained for this compound, and no recognisable patterns could be detected in the analysis.

5.5.6 Reaction of **14** and **17** with pentafluoropyridine in C_6D_6

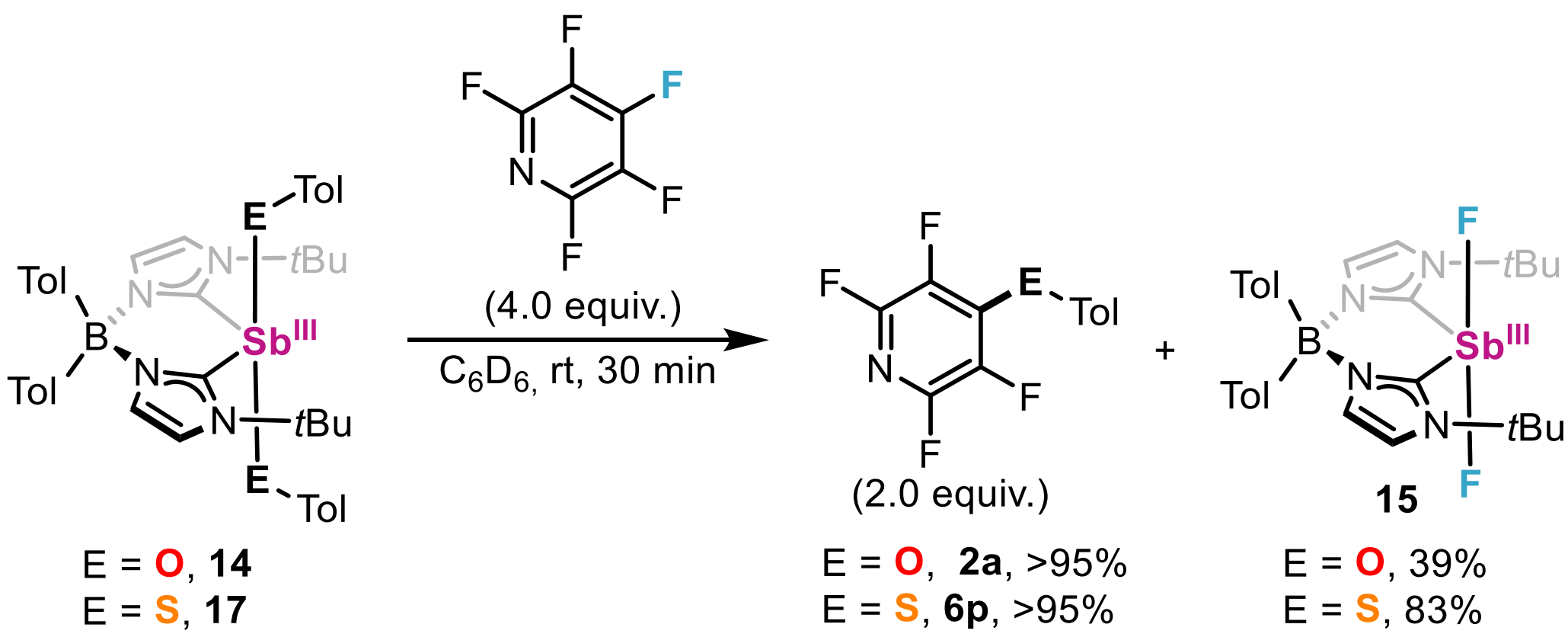

To a solution of Sb(III) dialkoxide complex **14** (15.5 mg, 1.0 equiv., 0.020 mmol) and α,α,α-trifluorotoluene (2.5 μL, 1.0 equiv., 0.020 mmol) in C_6D_6 (0.5 mL), pentafluoropyridine (8.8 µL, 4.0 equiv., 0.080 mmol) was added at rt in one portion and the mixture was stirred at that temperature for 30 min. The formation of a significant amount of black precipitate was observed during this time. The sample was then transferred to a J-Young NMR tube and analysed by ^{1}H (**Figure S28**) and ^{19}F NMR (**Figure S29**), indicating the formation of product **2a** in almost quantitative yield and Sb(III) difluoride complex **15** in 39% yield, respectively. ^{1}H

NMR spectrum exhibited significant decomposition of the Sb(III) difluoride complex **15** to Sb black (precipitate) and free ligand (60%).

In a similar manner, to a suspension of Sb(III) disulfide complex **17** (16 mg, 1.0 equiv., 0.020 mmol) and α,α,α-trifluorotoluene (2.5 μL, 1.0 equiv., 0.020 mmol) in C_6D_6 (0.5 mL), pentafluoropyridine (8.8 μL, 4.0 equiv., 0.080 mmol) was added at rt in one portion and the mixture was stirred at that temperature for 30 min. The formation of a clear, pale yellow solution and some black precipitate was observed during this time. The sample was then transferred to a J-Young NMR tube and analysed by ^{1}H (**Figure S32**) and ^{19}F NMR (**Figure S33**), indicating the formation of product **6p** in almost quantitative yield and Sb(III) difluoride complex **15** in 80% yield, respectively. ^{1}H NMR spectrum exhibited slight decomposition of the Sb(III) difluoride complex **15** to Sb black (precipitate) and free ligand (20%).

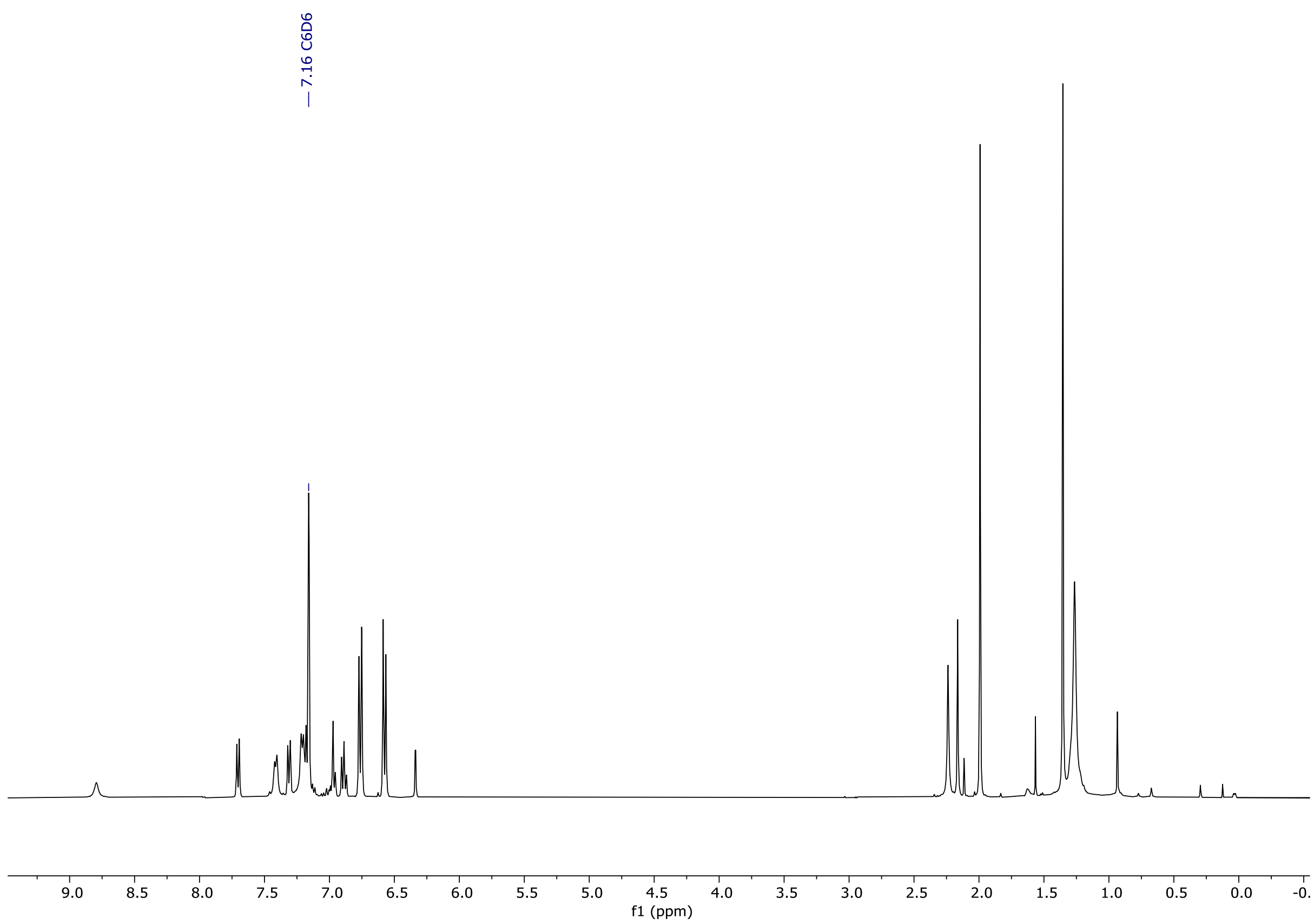

Figure S28. ^{1}H NMR spectrum of the reaction mixture of **14** with pentafluoropyridine and α,α,α-trifluorotoluene as IS after 30 min in C_6D_6.

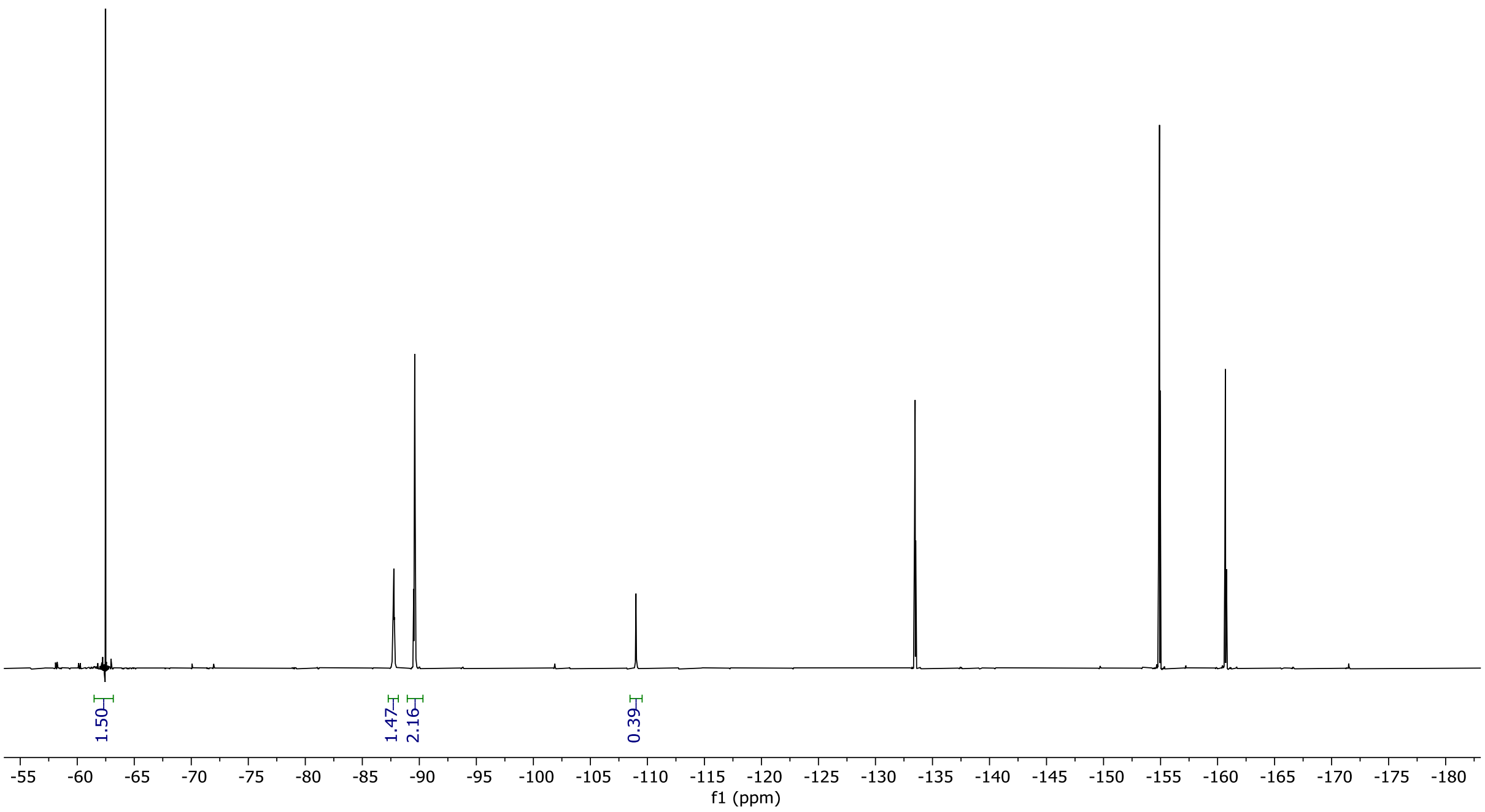

Figure S29. ^{19}F NMR spectrum of the reaction mixture of **14** with pentafluoropyridine and α,α,α-trifluorotoluene as IS after 30 min in C_6D_6.

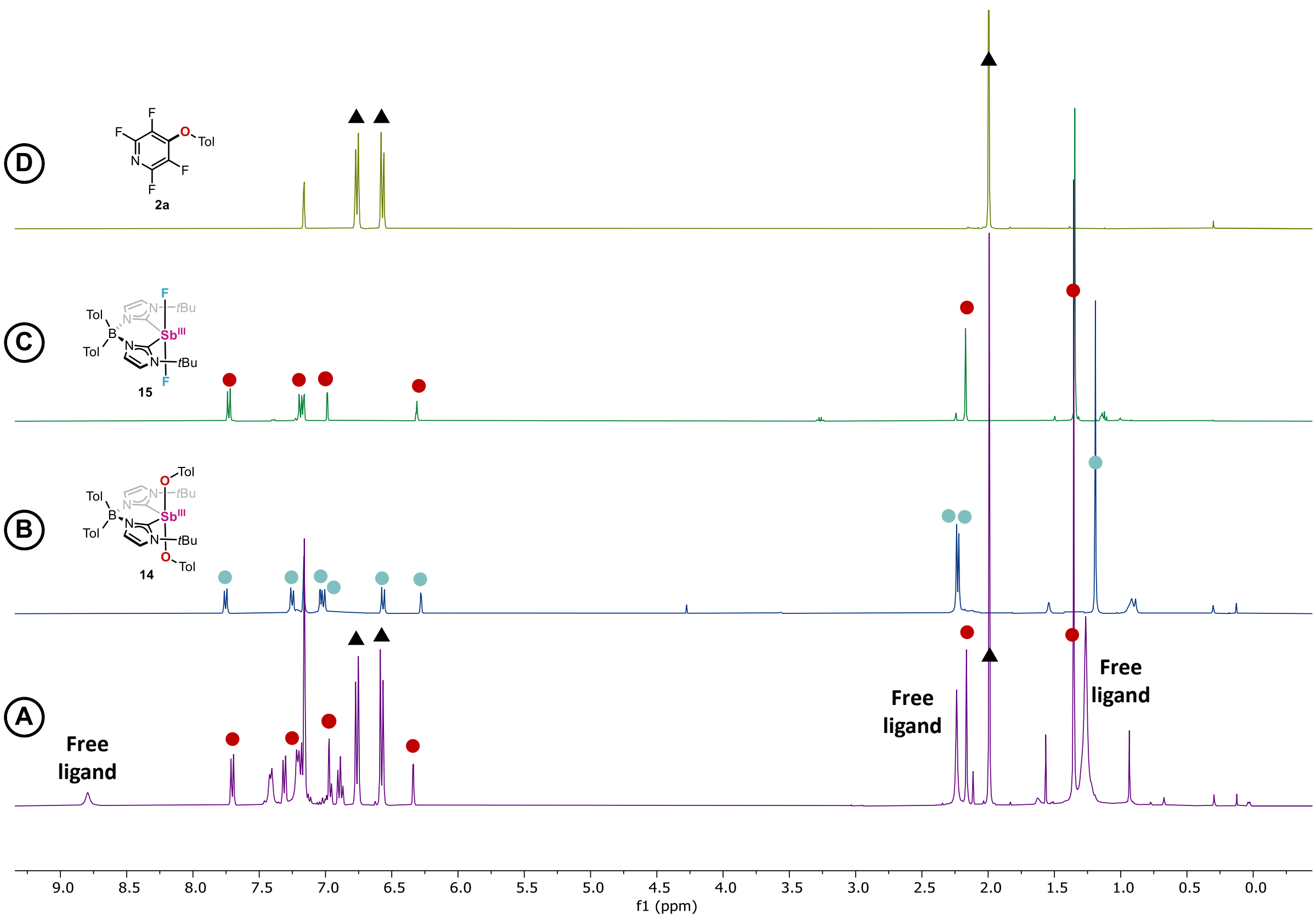

Figure S30. Stacked ^{1}H NMR spectra of: (**A**) reaction mixture of **14** with pentafluoropyridine and α,α,α-trifluorotoluene as IS after 30 min in C_6D_6; (**B**) pure **14** (blue dots); (**C**) pure **15** (red dots); and (**D**) pure **2a** (black triangles).

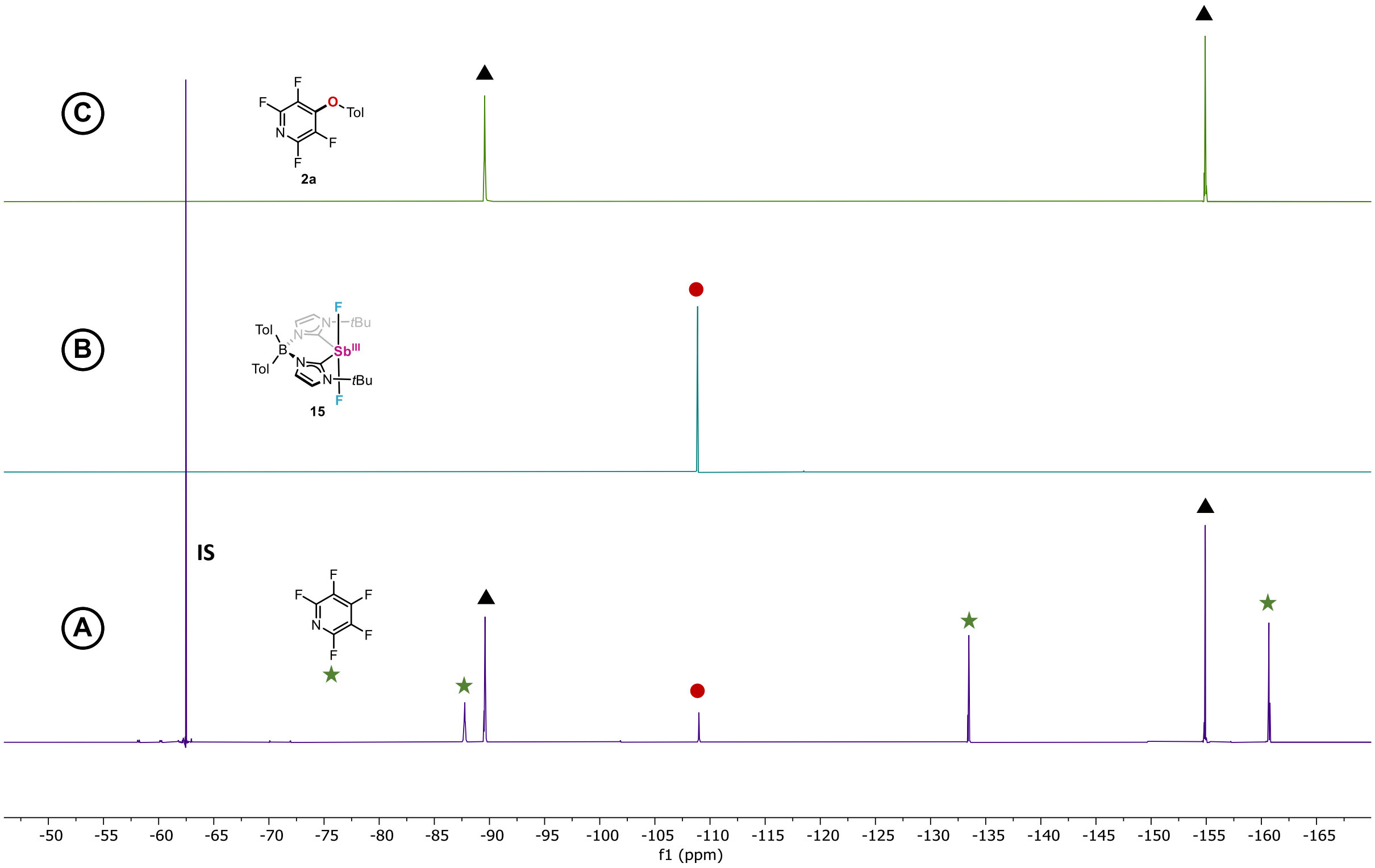

Figure S31. Stacked ^{19}F NMR spectra of: (**A**) reaction mixture of **14** with pentafluoropyridine and α,α,α-trifluorotoluene as IS after 30 min in C_6D_6; (**B**) pure **15** (red dots); (**C**) pure **2a** (black triangles). Excess pentafluoropyridine was marked as green stars.

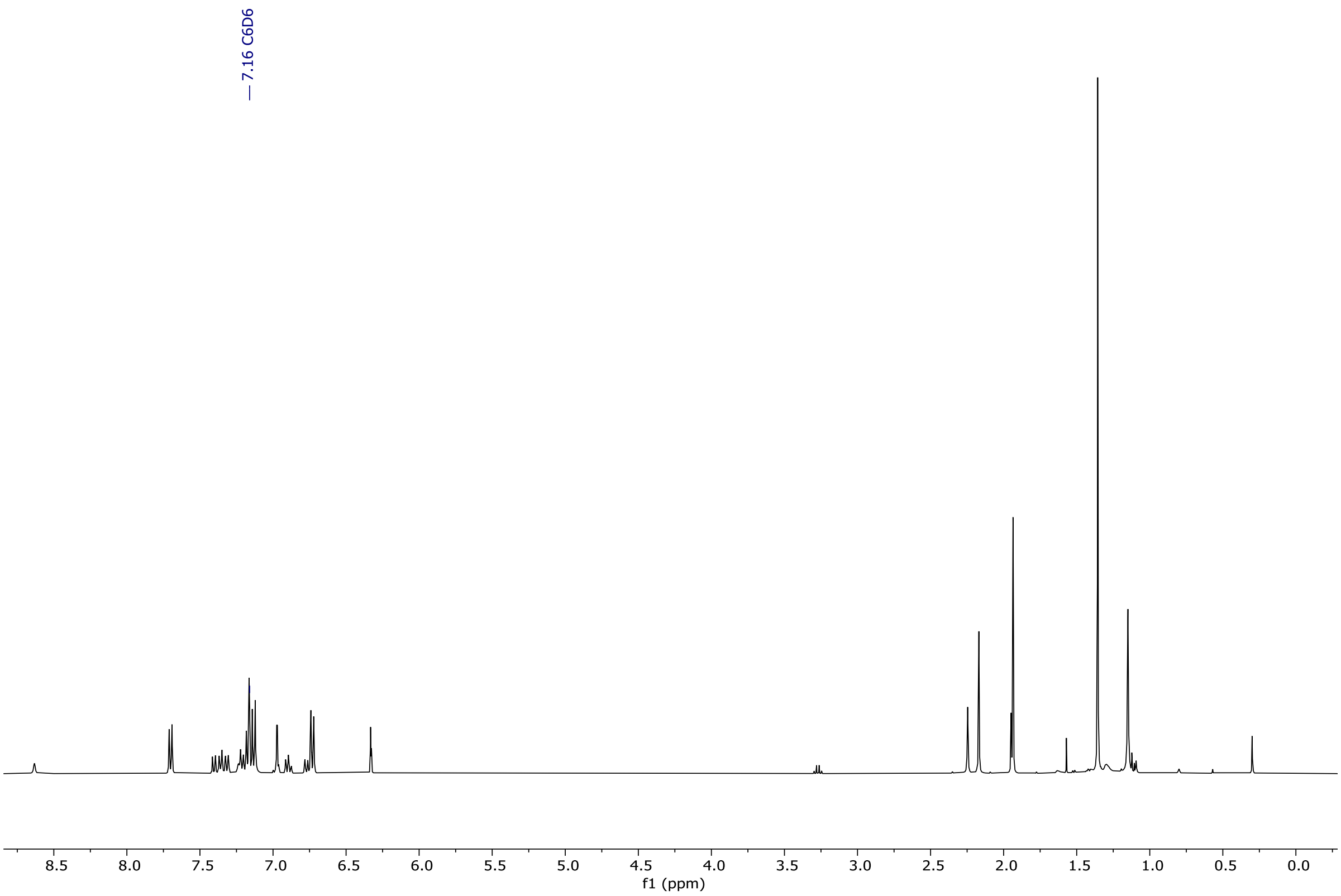

Figure S32. ^{1}H NMR spectrum of the reaction mixture of **17** with pentafluoropyridine and α,α,α-trifluorotoluene as IS after 30 min in C_6D_6.

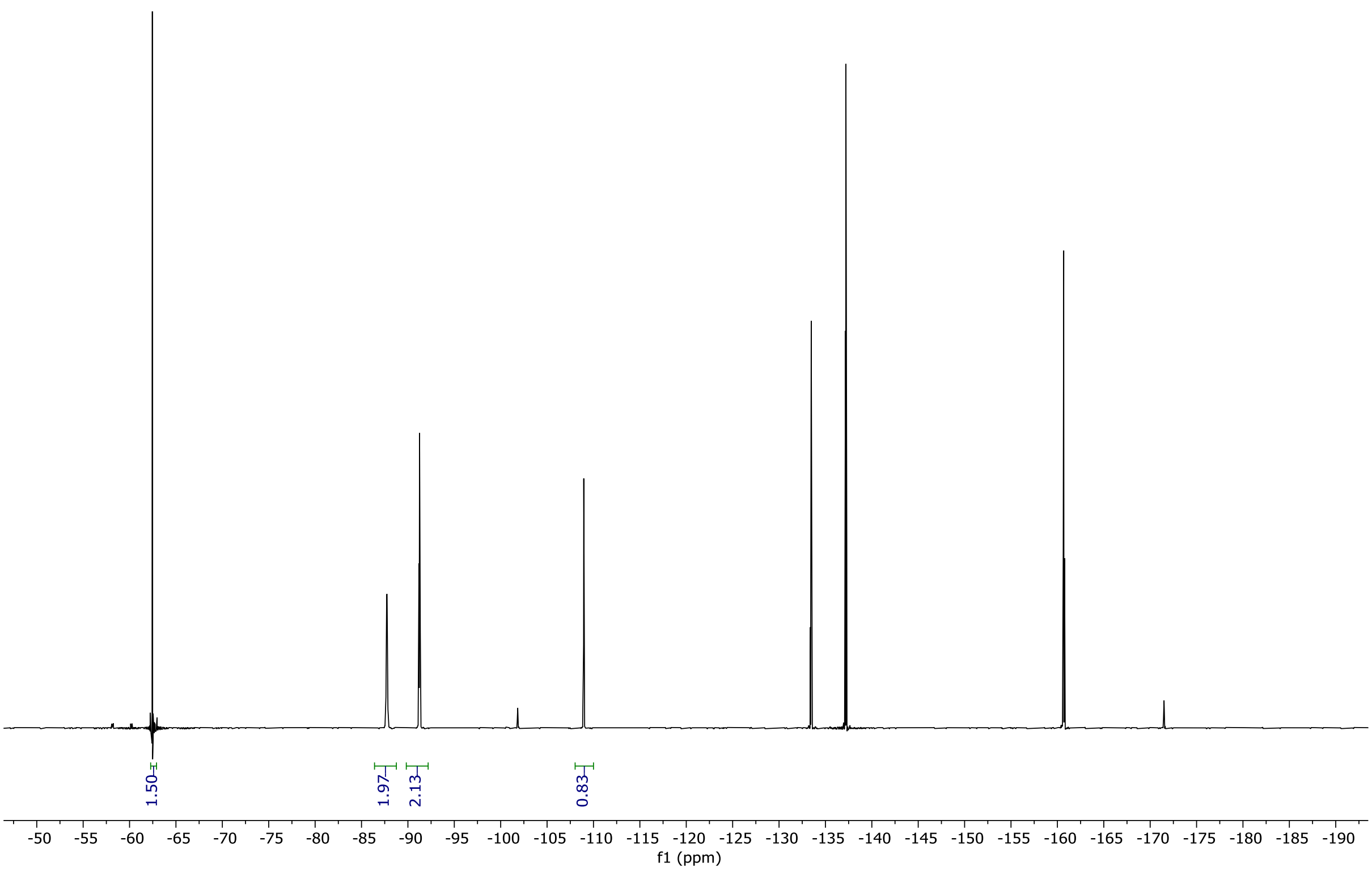

Figure S33. ^{19}F NMR spectrum of the reaction mixture of **17** with pentafluoropyridine and α,α,α-trifluorotoluene as IS after 30 min in C_6D_6.

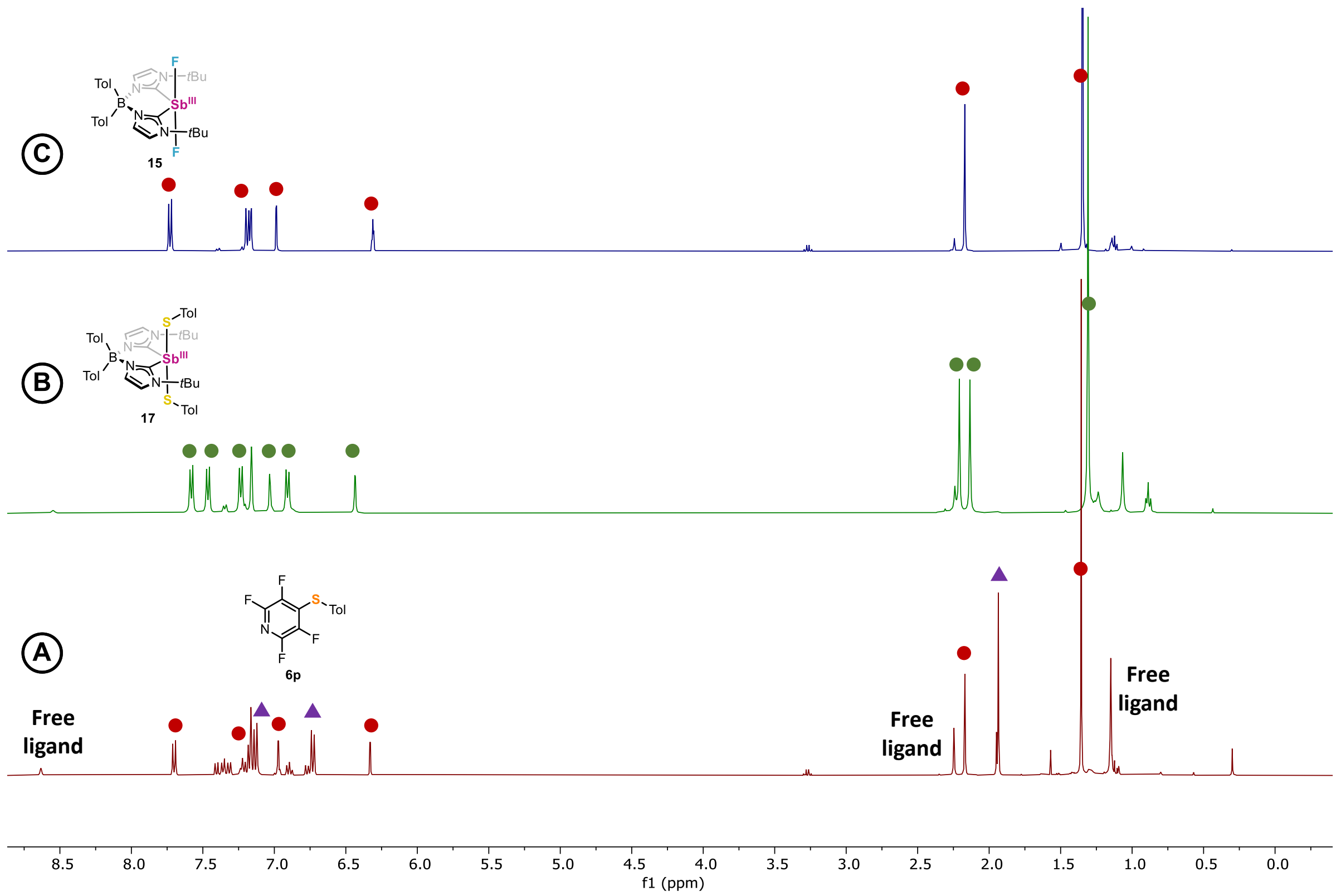

Figure S34. Stacked ^{1}H NMR spectra of: (**A**) reaction mixture of **17** with pentafluoropyridine and α,α,α-trifluorotoluene as IS after 30 min in C_6D_6; (**B**) pure **17** (green dots); and (**C**) pure **15** (red dots). Signals of **6p** were marked as purple triangles.

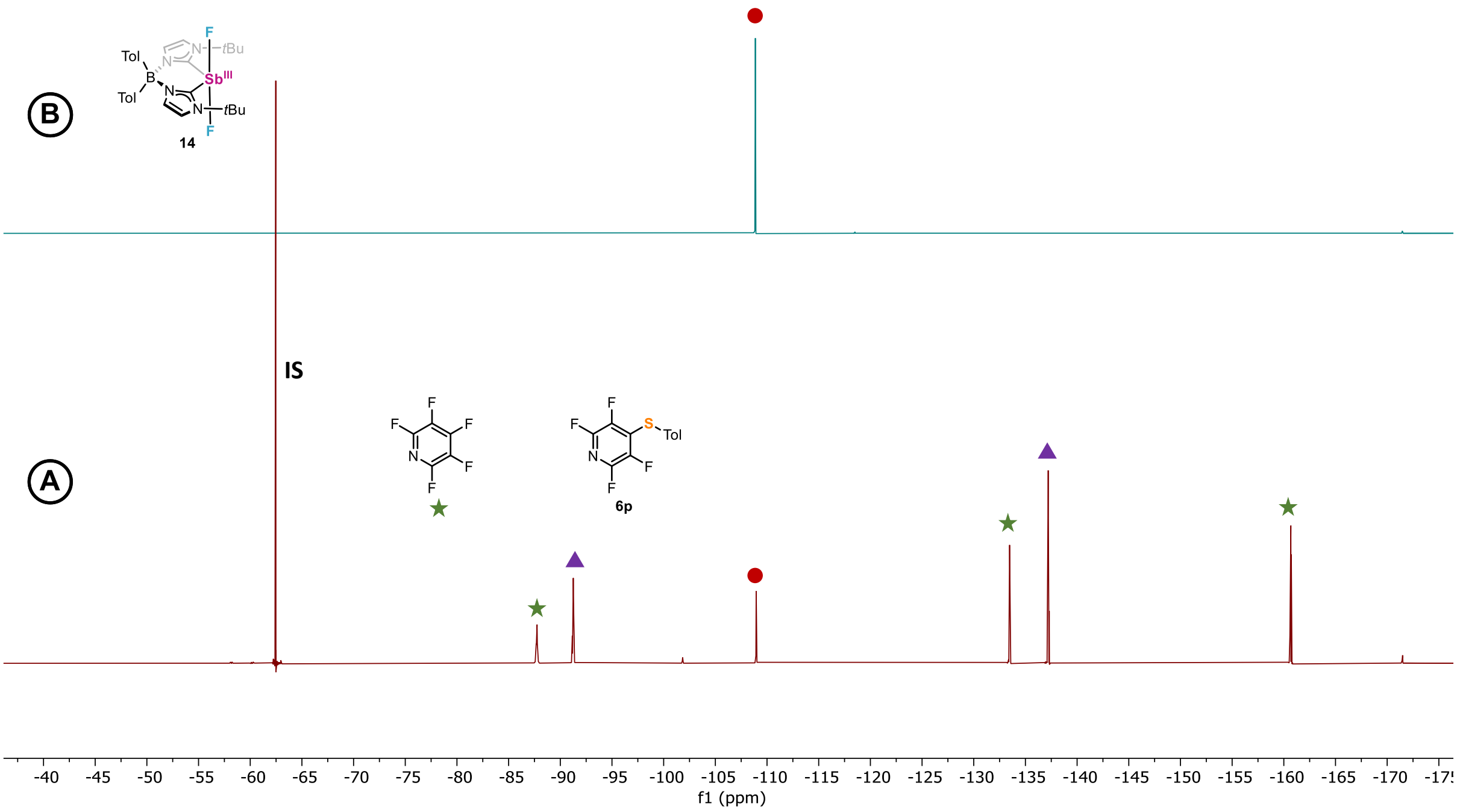

Figure S35. Stacked ^{1}H NMR spectra of: (**A**) reaction mixture of **17** with pentafluoropyridine and α,α,α-trifluorotoluene as IS after 30 min in C_6D_6; (**B**) pure **15** (red dots). Signals of **6p** were marked as purple triangles. Excess pentafluoropyridine was marked as green stars.

5.5.7 Synthesis of 2-(*p*-tolyloxy)-3,5,6-tetrafluoropyridine **S7’**

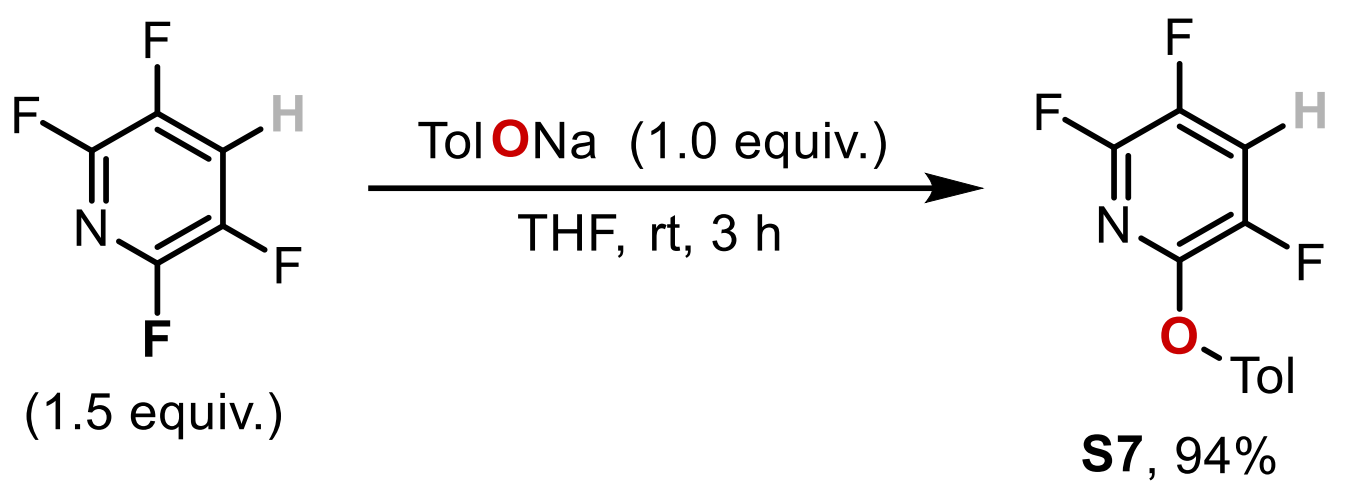

S7, 94%

To a solution of 2,3,5,6-tetrafluoropyridine **S7** (151 μL, 1.5 equiv., 1.50 mmol) in dry THF (1 mL), a solution of sodium 4-methylphenoxide (130 mg, 1.0 equiv., 1.00 mmol) in dry THF (2 mL) was added dropwise at rt. The mixture was then stirred at rt for 3 h. The solvent was then removed under vacuum to afford a clear viscous oil. The product was redissolved in DCM, observing precipitation of NaF, and purified through a short plug of SiO_2. Removal of the volatiles under vacuum afforded product **S7’** as a white crystalline solid in 94% yield (225 mg, 0.94 mmol).

^{1}H NMR (400 MHz, $CDCl_3$) δ = 7.50 (td, $J_{H\text{-}F}$ = 7.9, 7.0 Hz, 1H), 7.24 – 7.18 (m, 2H), 7.09 – 7.00 (m, 2H), 2.37 (s, 3H).

^{13}C{^{1}H} NMR (101 MHz, $CDCl_3$) δ = 150.9 (C), 145.4 – 142.3 (m, 3 x C-F), 139.4 (dd, $J_{C\text{-}F}$ = 256.8, 33.7 Hz, C-F), 135.2 (C), 130.3 (2 x CH), 120.7 (2 x CH), 117.5 (td, $J_{C\text{-}F}$ = 20.8, 3.4 Hz, CH), 21.0 (CH_3).

^{19}F{^{1}H} NMR (377 MHz, $CDCl_3$) δ = -91.56 (dd, $J_{F\text{-}F}$ = 30.3, 22.3 Hz, 1F), -137.79 (d, $J_{F\text{-}F}$ = 29.8 Hz, 1F), -145.00 (d, $J_{F\text{-}F}$ = 21.7 Hz, 1F).

HRMS (ESI-pos, m/z): calc’d for $C_{12}H_{19}F_3NO$ $[M+H]^+$ 240.06312; found 240.0627

Spectral data in C_6D_6:

^{1}H NMR (400 MHz, C_6D_6) δ = 6.96 – 6.90 (m, 2H), 6.89 – 6.82 (m, 2H), 6.32 (td, $J_{H\text{-}F}$ = 8.1, 7.0 Hz, 1H), 2.02 (s, 3H).

^{19}F{^{1}H} NMR (377 MHz, C_6D_6) δ = -92.36 (dd, $J_{F\text{-}F}$ = 30.3, 23.5 Hz), -137.94 (d, $J_{F\text{-}F}$ = 29.8 Hz), -145.26 (d, $J_{F\text{-}F}$ = 22.9 Hz).

5.6 Catalytic experiments

5.6.1 Catalytic defluoroalkoxylation of pentafluoropyridine

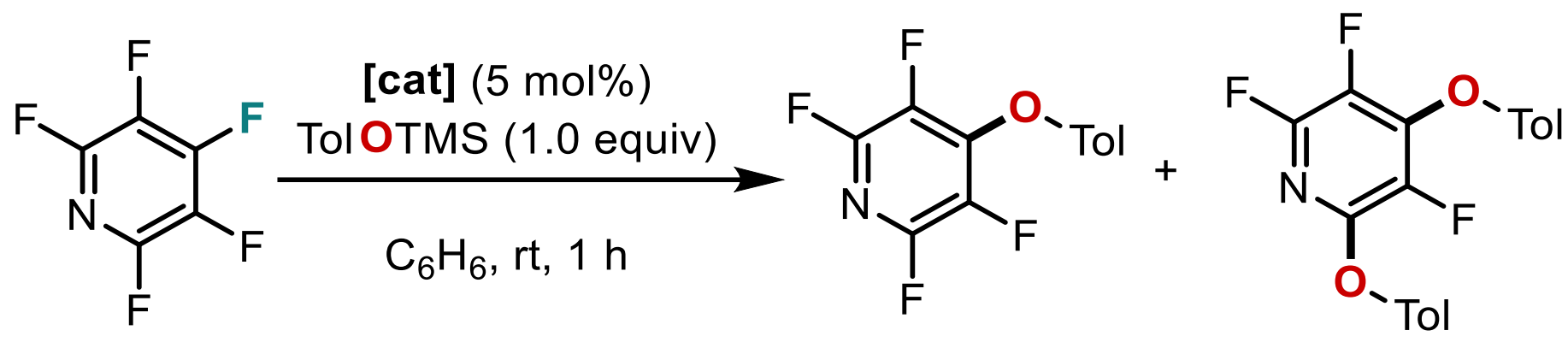

	2a	2a'
[cat] = **14**	>95%	<5%
[cat] = **15**	>95%	<5%

Following the general procedure, a solution of pentafluoropyridine (22 μL, 1.0 equiv., 0.20 mmol) and the corresponding trimethylsilyl nucleophile **S1a** (1.0 equiv.) in benzene (0.25 mL) was prepared. Then, a solution of Sb(III) difluoride complex **15** (6 mg, 5 mol%, 0.01 mmol) or Sb(III) dialkoxide complex **14** (8 mg, 5 mol%, 0.01 mmol) in benzene (0.25 mL) was added as catalyst. The mixture was then stirred at rt for 1. After this, α,α,α-trifluorotoluene (12.2 μL, 0.5 equiv., 0.10 mmol) was added to the mixture as internal standard and the reaction was monitored by ^{19}F NMR after adding $CDCl_3$ (0.2 mL). In both cases, almost quantitative yield of product **2a** was observed, along with <5% of by-product **2a’**.

5.6.2 Catalytic defluoroalkoxylation of product **2a**

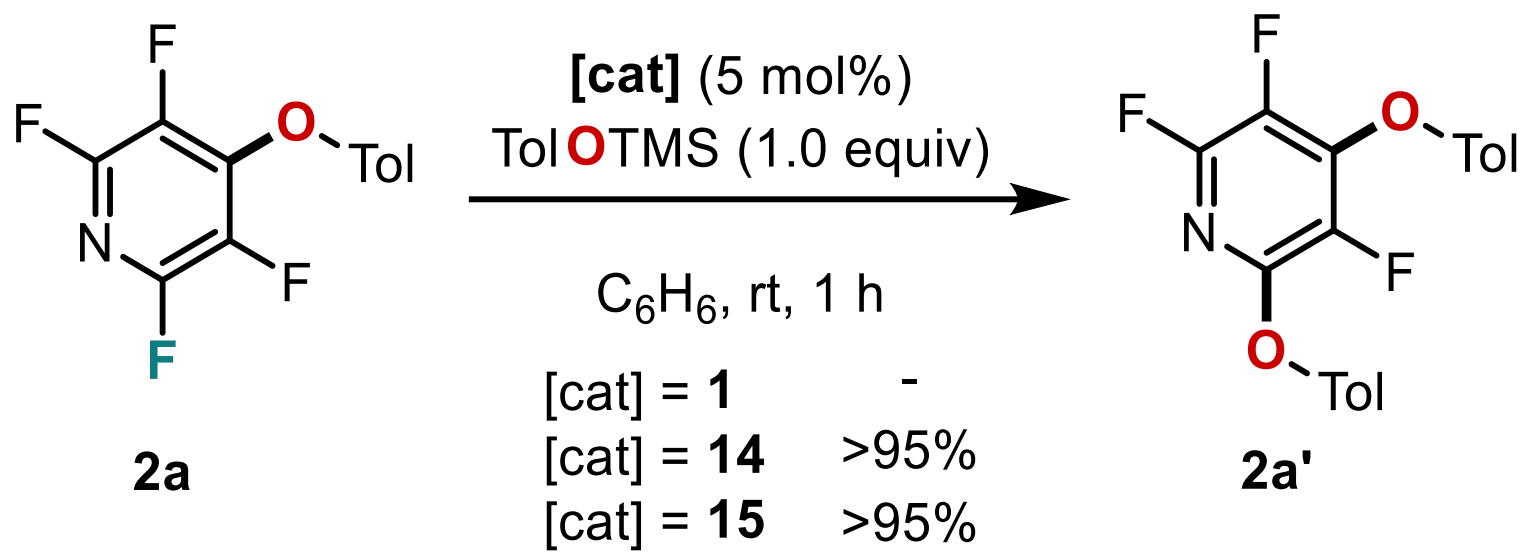

Following the general procedure, a solution of pyridine **2a** (51 mg, 1.0 equiv., 0.20 mmol) and the corresponding trimethylsilyl nucleophile **S1a** (36 mg, 1.0 equiv., 0.20 mmol) in benzene (0.25 mL) was prepared. Then, a solution of stibinidene complex **1** (5.6 mg, 5 mol%, 0.01 mmol), Sb(III) difluoride complex **15** (6 mg, 5 mol%, 0.01 mmol) or Sb(III) dialkoxide complex **14** (8 mg, 5 mol%, 0.01 mmol) in benzene (0.25 mL) was added as catalyst. The mixture was then stirred at rt for 1. After this, α,α,α -trifluorotoluene (12.2 μL, 0.5 equiv., 0.10 mmol) was added to the mixture as internal standard and the reaction was monitored by ^{19}F NMR after adding $CDCl_3$ (0.20 mL).

When stibinidene complex **1a** was used as catalyst no reaction was observed, detecting only the starting materials by ^{19}F NMR. However, the use of complexes **14** or **15** as catalysts afforded in both cases almost quantitative yield of product **2a’**.

5.6.3 NMR investigation of catalytic defluoroalkoxylation of pentafluoropyridine

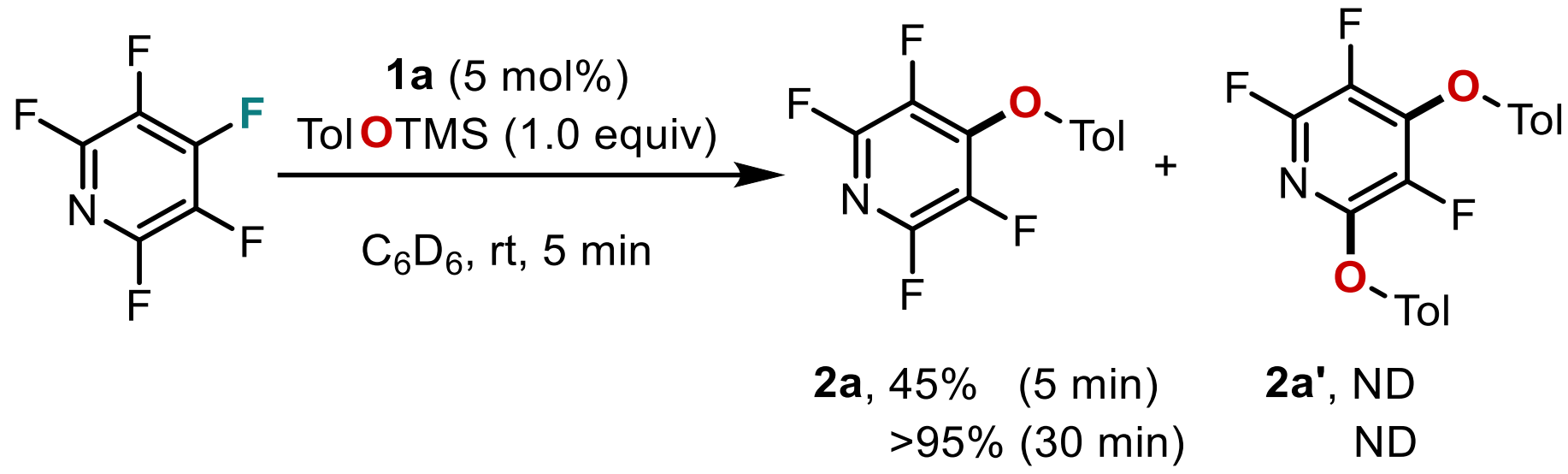

Following the general procedure, a solution of pentafluoropyridine (22 μL, 1.0 equiv., 0.20 mmol) and the corresponding trimethylsilyl nucleophile **S1a** (1.0 equiv.) in C_6D_6 (0.25 mL) was prepared and transferred to a J-Young NMR tube with septum. Then, a solution of Sb(III) difluoride complex **15** (6 mg, 5 mol%, 0.01 mmol) or stibinidene complex **1a** (5.6 mg, 5 mol%, 0.01 mmol) in C_6D_6 (0.25 mL) was added as catalyst. The mixture was shaken one and then immediately monitored by ^{19}F NMR. After 5 min (NMR residence time), a 1.2:1 mixture of pentafluoropyridine and product **2a** was observed (**Figure S36**), along with small traces of 2,3,5,6-tetrafluoropyridine **S7** probably due to decomposition of presumable species **16** (see stoichiometric experiment in section 5.3.1). After 30 min (**Figure S37**), the ratio changed to 1:>10 to the formation of product **2a**. In both cases, no visible signs of species **15** or other Sb-F species could be detected, strongly suggesting that dialkoxide species **14** is the resting state of the catalysis. ^{1}H NMR spectra didn't allow identification of species **14** due to low intensity of the expected signals and their overlap with the signal of both product **2a** and the silane employed as starting material.

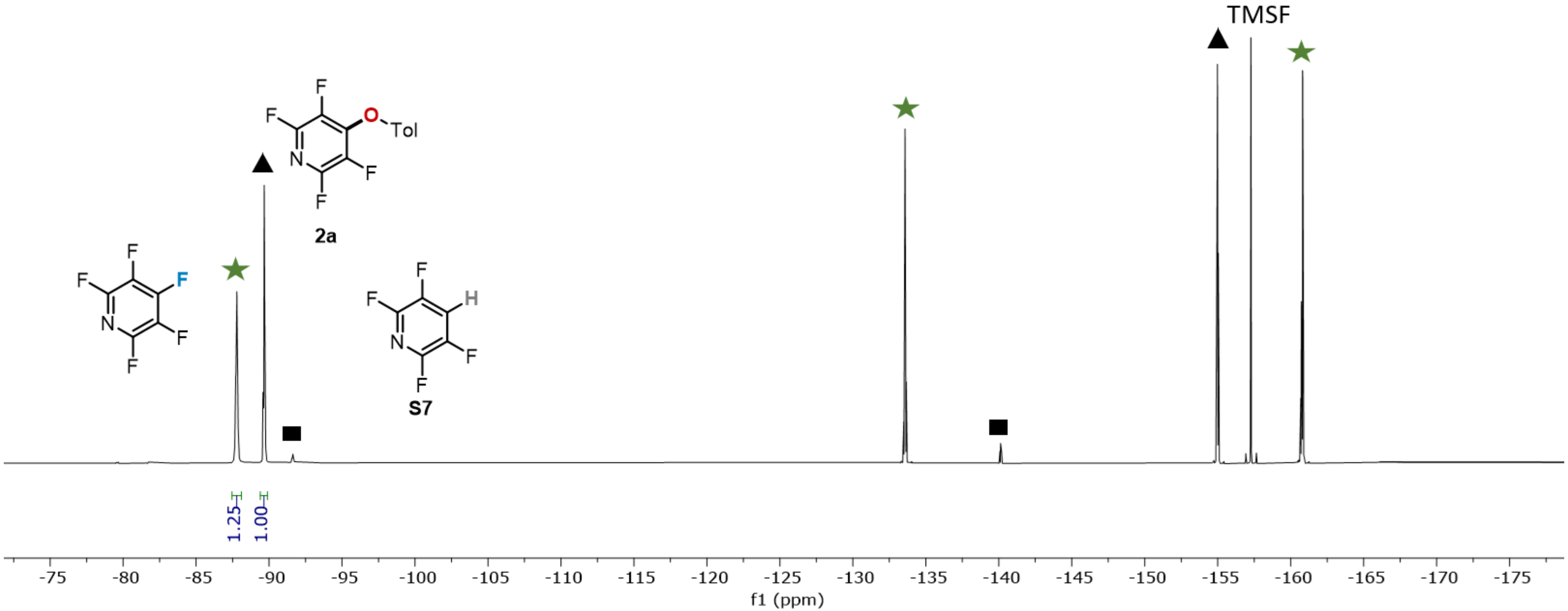

Figure S36: ^{19}F NMR of a regular catalytic reaction in C_6D_6 using stibinidene **1a** as catalyst after 5 min. Species **2a** (black triangles), pentafluoropyridine (green stars) and 2,3,5,6-tetrafluoropyridine **S7** (black squares) could be identified, along with TMSF.

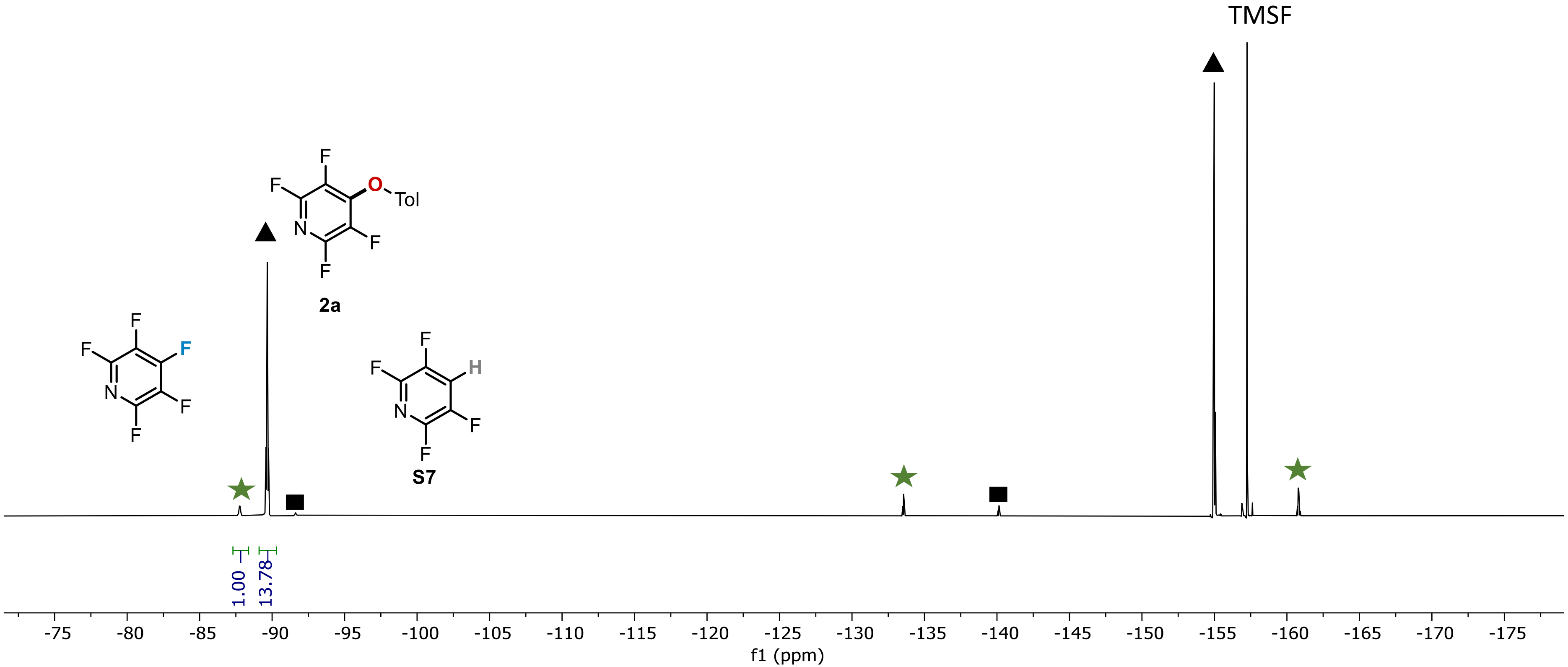

Figure S37: ^{19}F NMR of a regular catalytic reaction in C_6D_6 using stibinidene **1a** as catalyst after 30 min. Species **2a** (black triangles), pentafluoropyridine (green stars) and 2,3,5,6-tetrafluoropyridine **S7** (black squares) could be identified, along with TMSF.

5.6.4 Catalytic defluoroalkoxylation of octafluorobipyridine **10a**

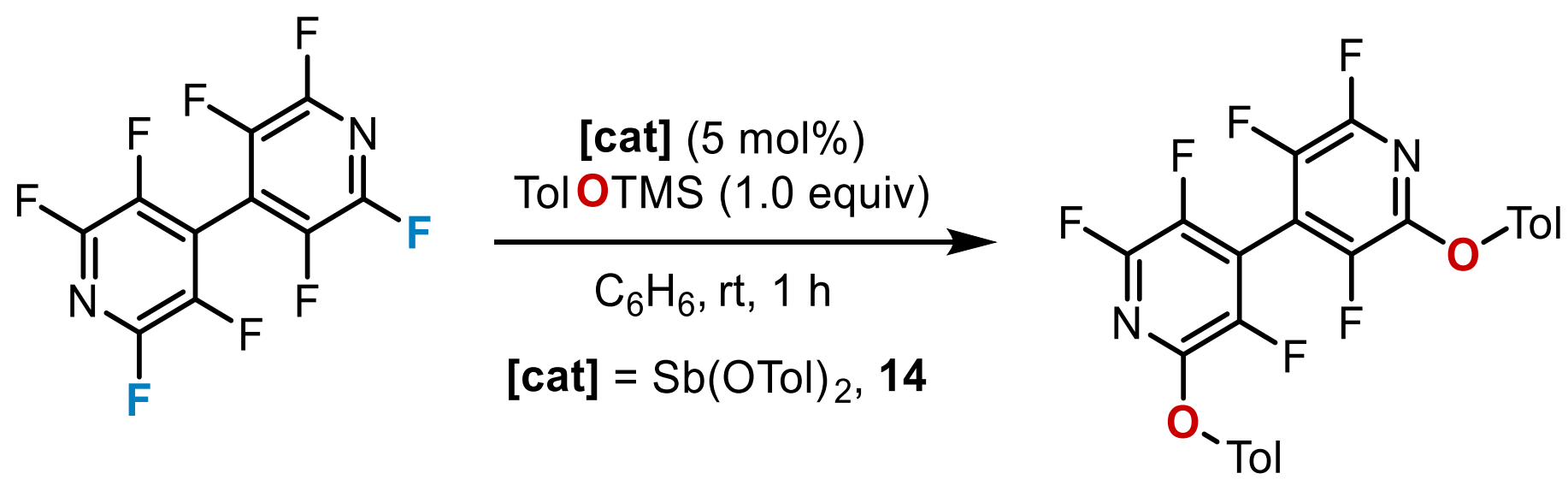

10a **10a'**, 84%

Following the general procedure, a solution of the bipyridine **10a** (60 mg, 1.0 equiv., 0.20 mmol) and the corresponding trimethylsilyl nucleophile **S1a** (180 mg, 5.0 equiv., 1.00 mmol) in benzene (0.25 mL) was prepared. Then, a solution of Sb(III) dialkoxide complex **14** (8 mg, 5 mol%, 0.01 mmol) in benzene (0.25 mL) was added as catalyst. The mixture was then stirred at rt for 1 h. After this, α,α,α-trifluorotoluene (0.10 mmol) was added to the mixture as internal standard and the reaction was monitored by ^{19}F NMR after adding $CDCl_3$ (0.20 mL), observing almost quantitative yield of product **10a'**. Flash column chromatography (Hex:DCM, 3:1, R_f = 0.23) afforded **10a'** as an off-white sticky solid in 84% yield (80 mg, 0.17 mmol).

^{1}H NMR (400 MHz, $CDCl_3$) δ = 7.39 – 7.27 (m, 4H), 7.24 – 7.11 (m, 4H), 2.47 (s, 6H).

^{13}C{^{1}H} NMR (101 MHz, $CDCl_3$) δ 150.4 (2 x C), 145.5 – 144.9 (m, 2 x C-F), 144.1 (dd, J_{C-F} = 239.4, 13.6 Hz, 2 x C-F), 141.0 (dd, J_{C-F} = 265.1, 6.4 Hz, 2 x C-F), 136.7 (dd, J_{C-F} = 261.6, 30.5 Hz, 2 x C-F), 135.7 (2 x C), 130.5 (4 x CH), 120.9 (4 x CH), 118.5 – 117.5 (m, 2 x C-F), 21.1 (2 x CH_3).

^{19}F{^{1}H} NMR (377 MHz, $CDCl_3$) δ = -89.25 (ddt, J_{F-F} = 32.0, 22.9, 4.0 Hz), -136.03 – -137.71 (m), -144.98 (dquint, J_{F-F} = 21.7, 4.6 Hz).

HRMS (ESI-pos, m/z): calc'd for $C_{24}H_{15}F_6N_2O_2$ $[M+H]^+$ 477.10327; found 477.1022.

6 Computational details

Geometry optimizations were performed using the ORCA 6.0.0 software.[25] All geometry optimizations were computed using the functional PBE0[26,27] functional with Grimme dispersion corrections D3[28] and the Becke-Jonson damping function[29] in combination with the def2-SVP basis set.[30] Analytical Hessians were computed to determine the nature of stationary points (one and zero imaginary frequencies for transition states and minima, respectively)[31] and to calculate unscaled zero-point energies (ZPEs) as well as thermal corrections and entropy effects using the standard statistical-mechanics relationships for an ideal gas.

The exploration of the potential energy surface (PES) was performed at the PBE0D3(BJ)/def2-SVP level of theory, using CPCM with benzene or acetonitrile as implicit solvation depending on the studied mechanism. All optimized structure energies were later refined at the CPCM(benzene)-PBE0-D3(BJ)/def2-TZVPP or CPCM(acetonitrile)-PBE0-D3(BJ)/def2-TZVPP level of theory. The nature of the stationary points was characterized with the normal mode analysis (one and zero imaginary frequencies for transition states and local minima, respectively). All transition states were confirmed either by IRC or by optimizing the geometries following the negative eigenvalue of the Hessian.

6.1 Plausible mechanisms for defluorinative alkoxylation of pentafluoropyridine

A redox mechanism for the formation of product **2n** is presented and discussed in the main text (Figure 1). Similarly, the lowest energy pathways invoking redox-neutral, SNAr-type mechanisms for the formation of **2n**, either with or without a ligand scrambling step, and transmetallation steps are presented and discussed in the main text (Figure 2 and Figure 3). In addition to these three plausible mechanisms, an alternative route involving an initial oxidative addition of the Si-O of $PhOSiMe_3$ was identified (Figure S38). In this case, Si–O bond oxidative addition into species **1a** would generate Sb(III) species **INT10** (ΔG = 34.1 kcal mol^{-1}). This highly endergonic process would occur through **TS11** ($\Delta\Delta G$ = 39.9 kcal mol^{-1}), making this reaction pathway highly unlikely. These results are in agreement with the lack of reactivity between stibinidene **1a** and the trimethylsilylethers employed in the study. Thus, no further calculations were performed.

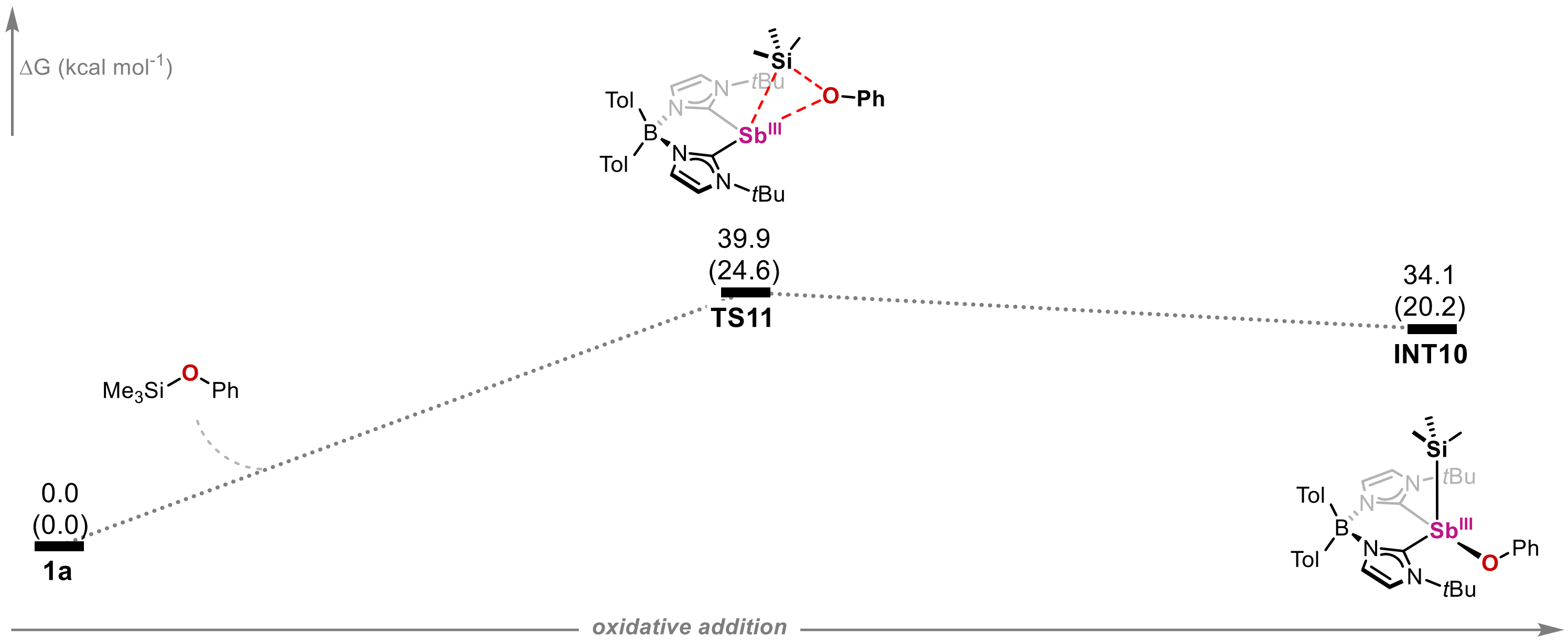

Figure S38. Gibbs energy (ΔG) for an alternative initial oxidative addition of the Si-O bond of $PhSiOMe_3$ by stibinidene **1a**. Enthalpy (ΔH) in parentheses. All calculations were performed at the CPCM(benzene)-PBE0-D3(BJ)/def2-TZVPP//CPCM(benzene)-PBE0-D3(BJ)/def2-SVP level of theory.

6.2 Sb-catalyzed homodimerization of pentafluoropyridine

Based on our experimental results (see Sections 4.4.1 and 4.4.2), a redox Sb-catalyzed pathway involving a ligand scrambling and the Sb(I)/Sb(III) redox couple was evaluated both in benzene and acetonitrile.

As previously stated, the reaction begins with oxidative addition of Sb(I) species **1a** to the C4–F bond of the pyridine ring to afford the more thermodynamically favoured species **INT2** (ΔG = -18.7 kcal mol^{-1}), in which the fluorine occupies an axial site after isomerization. Subsequent ligand scrambling with another molecule of **INT2** would generate Sb(III) difluoride species **15** and Sb(III) bis(4-tetrafluoropyridyl) species **16** (ΔG = -12.9 kcal mol^{-1}). This process was found to be endergonic by +5.8 kcal mol^{-1}. At this stage, reduction of Sb(III) difluoride species **15** by Mashima's reagent **S6** would regenerate one molecule of stibinidene **1a** while forming pyridazine and two molecules of TMSF in a highly exergonic process ($\Delta\Delta$G = -48.3 kcal mol^{-1}). On the other hand, isomerization of species **16** to **INT11** (ΔG = -49.9 kcal mol^{-1}) followed by a reductive elimination step would generate another molecule of stibinidene **1a** and form dimerization product **10a**. Although this process would be thermodynamically favoured by -17.5 kcal mol^{-1}, the energetic barrier to overcome **TS12** (ΔG = -24.6 kcal mol^{-1}) from species **16** would make this process highly unfeasible in benzene ($\Delta\Delta$G = +36.6 kcal mol^{-1}).

When the same reaction profile was computed in acetonitrile, although comparable values were found for the initial oxidative addition process, significant differences in energy were observed for the ligand scrambling and reductive elimination stages. For instance, the ligand scrambling step from **INT2** to form species **15** and **16** becomes more thermodynamically disfavoured ($\Delta\Delta$G = +13.5 kcal mol^{-1} vs +5.8 kcal mol^{-1} in benzene). In contrast, the energic barrier for reductive elimination of product **10a** from species **16** decreased dramatically ($\Delta\Delta$G = +22.6 kcal mol^{-1} in acetonitrile vs +36.6 kcal mol^{-1} in benzene). Additionally, this last step is more exergonic ($\Delta\Delta$G = -28.9 kcal mol^{-1} acetonitrile vs -24.6 kcal mol^{-1} in benzene). These results would account for the experimental results observed, as the ligand scrambling steps were observed in both solvents, but reductive elimination to form species **10a** only took place efficiently in acetonitrile.

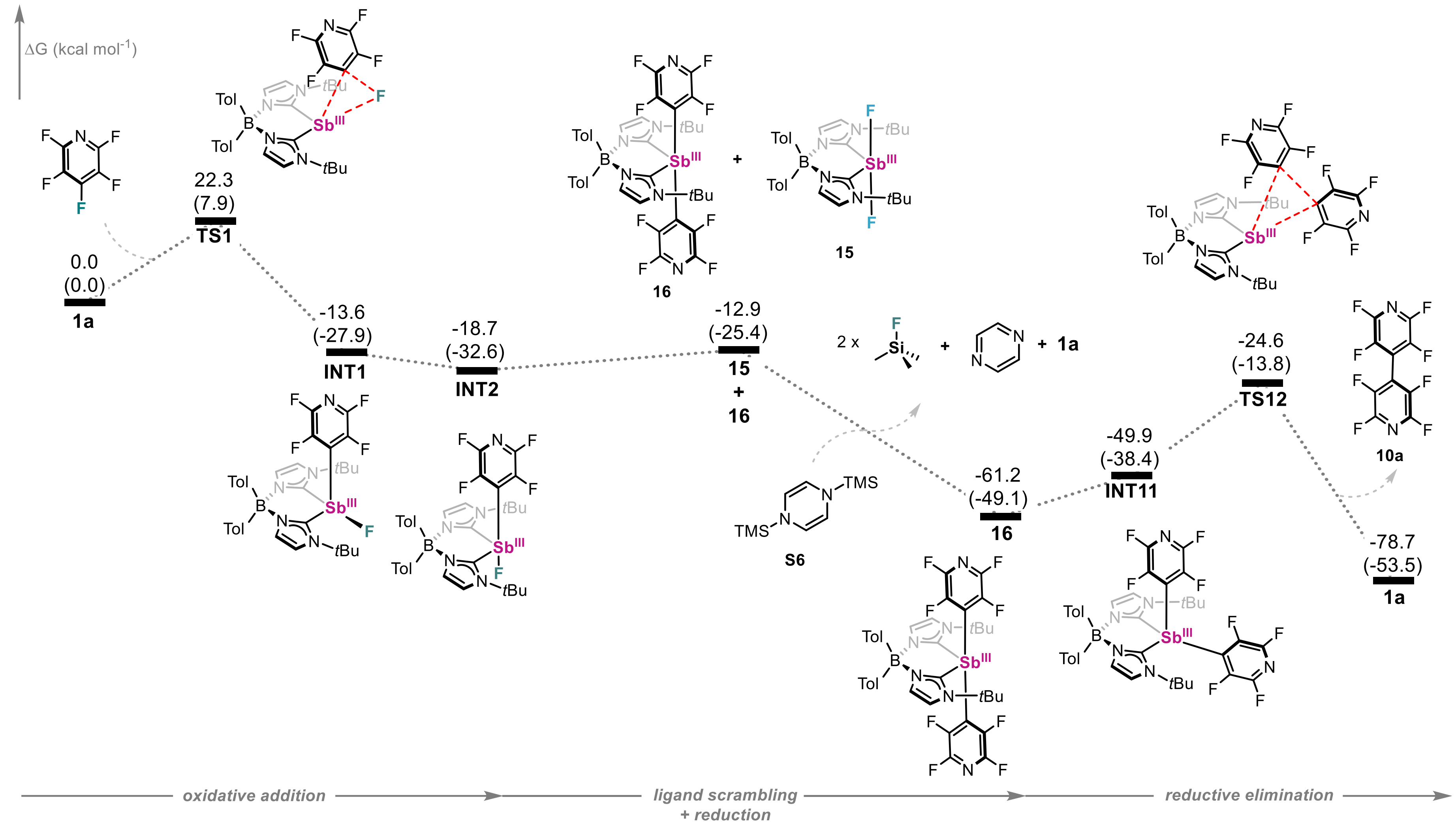

Figure S39. Gibbs energy (ΔG) for the stibinidene-catalyzed homodimerization of pentafluoropyridine proceeding *via* redox catalysis using species **S6** as reductant in benzene. Enthalpy (ΔH) in parentheses. Calculations were performed at the CPCM(benzene)-PBE0-D3(BJ)/def2-TZVPP//CPCM(benzene)-PBE0-D3(BJ)/def2-SVP level of theory.

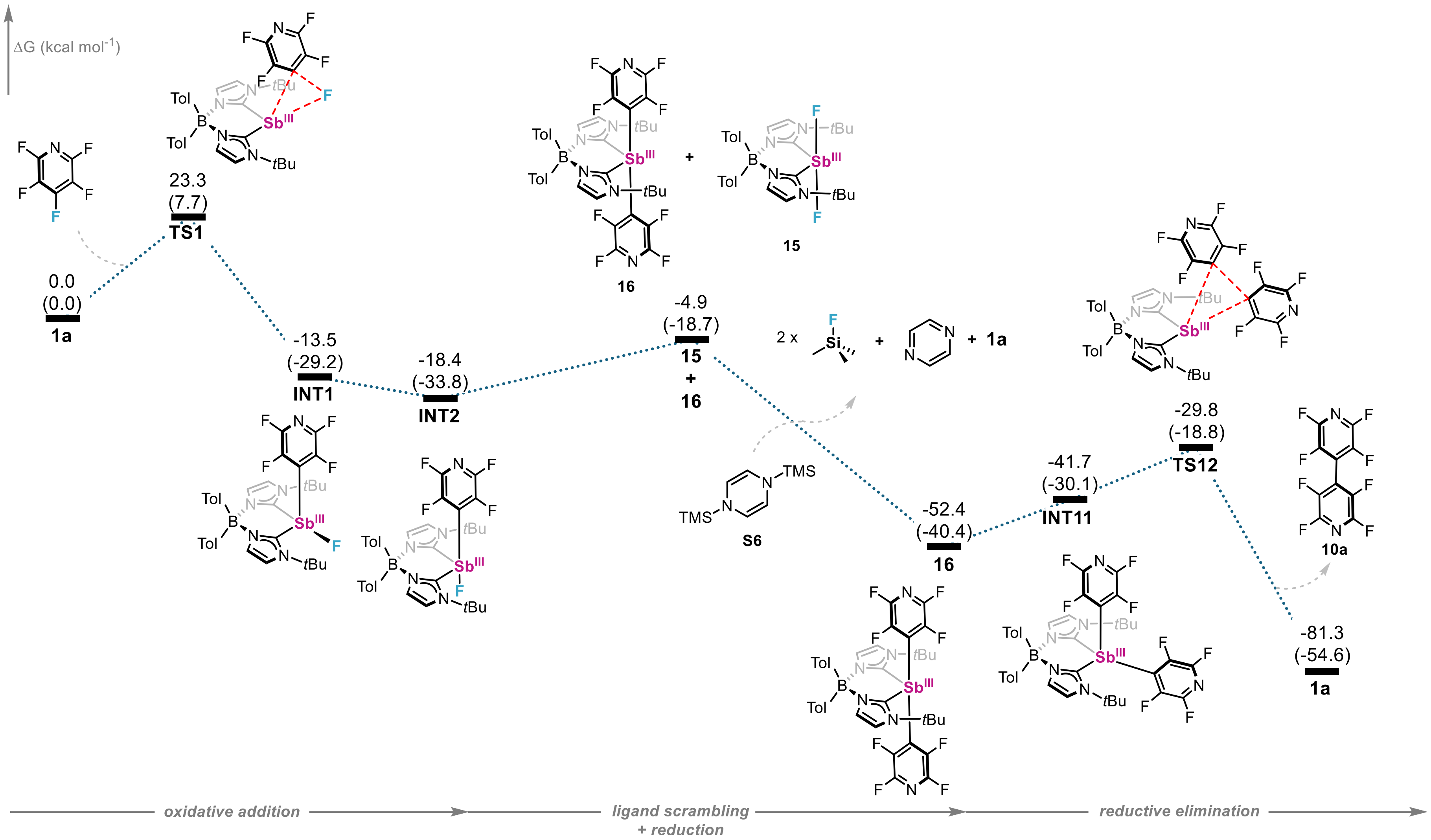

Figure S40. Gibbs energy (⊿G) for the stibinidene-catalyzed homodimerization of pentafluoropyridine proceeding *via* redox catalysis using species **S6** as reductant in acetonitrile. Enthalpy (⊿H) in parentheses. Calculations were performed at the CPCM(acetonitrile)-PBE0-D3(BJ)/def2-TZVPP//CPCM(acetonitrile)-PBE0-D3(BJ)/def2-SVP level of theory.

6.3 Sb-catalyzed defluorosilylation of pentafluoropyridine

Taking into account the work developed by Cornella and co-workers,[32] a potentially competing redox pathway involving a defluorinative silylation reaction was computed in acetonitrile.

As previously stated, the reaction begins with oxidative addition of Sb(I) species **1a** to the C4–F bond of the pyridine ring to afford the more thermodynamically favoured species **INT2** ($\Delta G = -18.4$ kcal mol^{-1}). At this stage, reaction of **INT2** with Mashima's reagent **S6** would regenerate $PyrF_4$-Sb(III)-$SiMe_3$ species **INT12** ($\Delta G = -16.5$ kcal mol^{-1}) while forming pyridazine and one molecule of TMSF in a slightly endergonic process ($\Delta\Delta G = +1.9$ kcal mol^{-1}). Then, isomerization of **INT12** to **INT13** ($\Delta G = -5.7$ kcal mol^{-1}) followed by a reductive elimination step would generate another molecule of stibinidene **1a** and silylated product **S8** through **TS13** ($\Delta G = +4.5$ kcal mol^{-1}). This process is highly feasible in acetonitrile from an energetic point of view, since it would be highly exergonic ($\Delta\Delta G = -38.9$ kcal mol^{-1}) with a feasible barrier of +21.0 kcal mol^{-1}, compatible with the experimental conditions employed. However, no traces of product **S8** were experimentally detected during the catalytic homodimerization reaction of pentafluoropyridine.

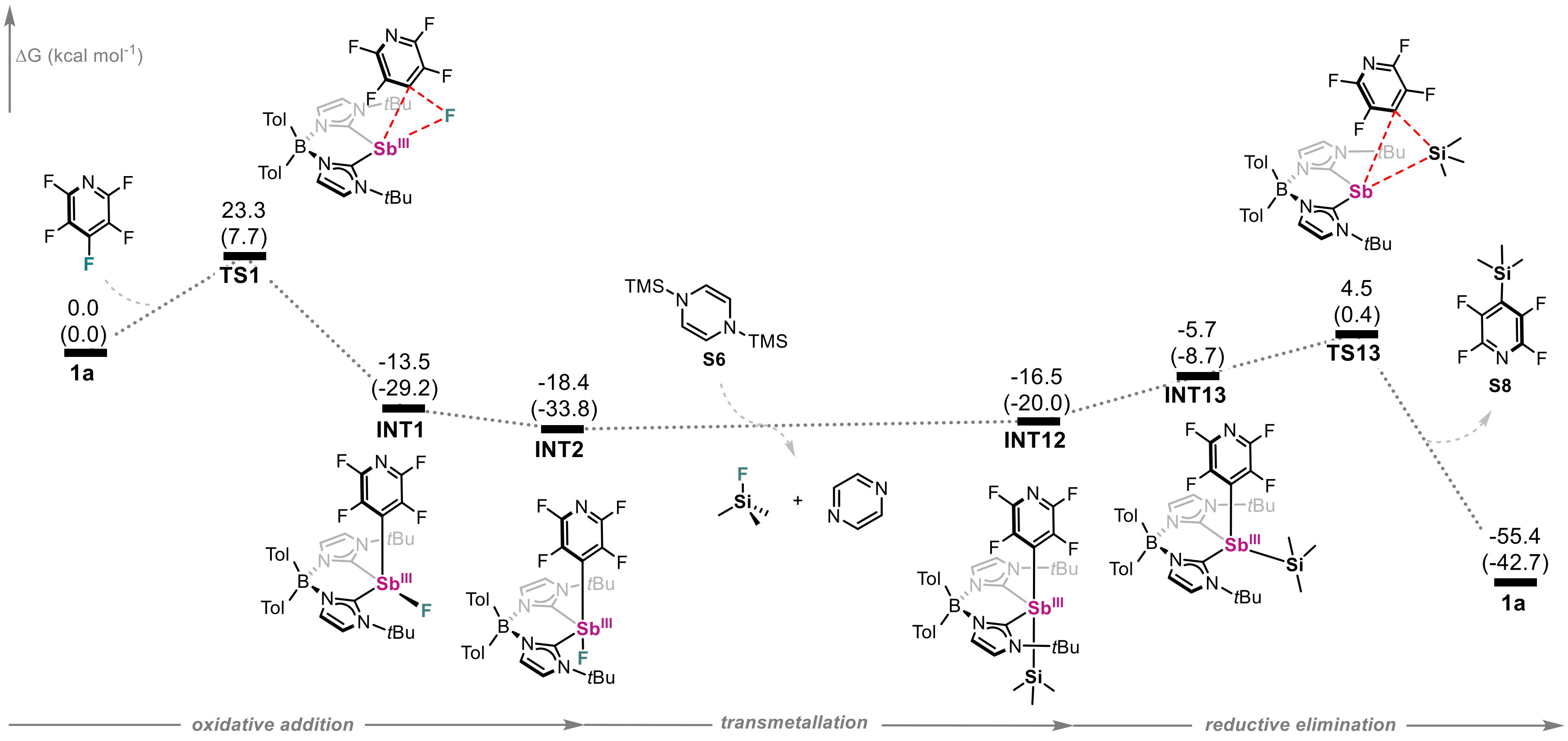

Figure S41. Gibbs energy (ΔG) for a potential stibinidene-catalyzed defluorosilylation of pentafluoropyridine proceeding via redox catalysis using species **S6** as silyl moiety source in acetonitrile. Enthalpy (ΔH) in parentheses. Calculations were performed at the CPCM(acetonitrile)-PBE0-D3(BJ)/def2-TZVPP//CPCM(acetonitrile)-PBE0-D3(BJ)/def2-SVP level of theory.

6.4 Mechanism A – xyz coordinates (in Å) and Energies (in Hartree)

6.4.1 **Stibinidene 1a**

Energy(PBE0-D3(BJ)/def2-TZVPP) = -1571.86482602289

6	1.076894000	-2.419080000	-1.034963000
7	0.472764000	-1.273555000	-0.578076000
6	-0.842939000	-1.507575000	-0.417164000
6	0.122158000	-3.376629000	-1.152699000
1	0.209164000	-4.408320000	-1.470780000
1	2.143971000	-2.467070000	-1.227150000
7	-1.074053000	-2.804279000	-0.765864000
6	-2.388171000	-3.498089000	-0.796805000
51	-2.233358000	0.000146000	0.261570000
7	0.472995000	1.273687000	-0.577592000
6	-0.842699000	1.507815000	-0.416812000
6	0.122741000	3.376962000	-1.151668000
7	-1.073597000	2.804651000	-0.765178000
1	0.209926000	4.408732000	-1.469443000
1	2.144434000	2.467120000	-1.226193000
6	1.077328000	2.419237000	-1.034129000
6	-2.387621000	3.498647000	-0.796095000
5	1.252561000	-0.000081000	-0.079794000
6	-3.068139000	3.349403000	0.563567000
1	-3.340434000	2.307947000	0.788297000
1	-2.414905000	3.716122000	1.368800000
1	-3.999837000	3.933024000	0.572603000
6	-2.172129000	4.987139000	-1.059446000
1	-1.538045000	5.450328000	-0.289027000
1	-1.731532000	5.174133000	-2.049225000
1	-3.151018000	5.485250000	-1.035256000
6	-3.234166000	2.903618000	-1.921061000
1	-2.738682000	3.052829000	-2.892043000
1	-3.389478000	1.824711000	-1.769490000
1	-4.216554000	3.397159000	-1.949414000
6	-3.234779000	-2.902655000	-1.921515000
1	-3.389928000	-1.823767000	-1.769648000
1	-2.739431000	-3.051681000	-2.892594000
1	-4.217238000	-3.396055000	-1.949877000
6	-3.068505000	-3.349083000	0.562968000
1	-2.415205000	-3.716037000	1.368040000
1	-3.340688000	-2.307655000	0.787961000
1	-4.000253000	-3.932625000	0.571986000
6	-2.172907000	-4.986551000	-1.060523000
1	-1.732510000	-5.173381000	-2.050420000
1	-1.538737000	-5.449983000	-0.290320000
1	-3.151851000	-5.484552000	-1.036269000

6	1.286942000	-0.000399000	1.539042000
6	1.345693000	-1.192186000	2.274405000
6	1.345506000	1.191041000	2.274952000
6	1.445173000	-1.197271000	3.664714000
6	1.444983000	1.195490000	3.665277000
6	1.491399000	-0.001051000	4.389982000
1	1.307006000	-2.151707000	1.748350000
1	1.306651000	2.150809000	1.749348000
1	1.485566000	-2.150497000	4.201419000
1	1.485212000	2.148468000	4.202432000
6	2.728045000	-0.000003000	-0.750578000
6	2.884986000	0.000635000	-2.149123000
6	3.902602000	-0.000447000	0.008797000
6	4.139056000	0.000778000	-2.750809000
6	5.166346000	-0.000300000	-0.587739000
6	5.309635000	0.000310000	-1.977183000
1	1.995278000	0.001037000	-2.788157000
1	3.830072000	-0.000938000	1.100055000
1	4.217839000	0.001267000	-3.842674000
1	6.061429000	-0.000675000	0.041958000
6	1.556983000	-0.001427000	5.890378000
1	2.077843000	-0.892395000	6.271451000
1	2.075791000	0.890502000	6.271982000
1	0.545581000	-0.002749000	6.331630000
6	6.662059000	0.000528000	-2.629624000
1	6.795331000	-0.883815000	-3.273523000
1	6.795855000	0.886054000	-3.271795000

6.4.2 **PyrF5**

Energy(PBE0-D3(BJ)/def2-TZVPP) = -744.036204165279

6	-1.259345000	0.000000000	-1.733144000
6	-1.259346000	1.203913000	-1.030271000
6	-1.259345000	1.130988000	0.360545000
7	-1.259345000	0.000000000	1.017030000
6	-1.259345000	-1.130988000	0.360545000
6	-1.259345000	-1.203914000	-1.030271000
9	-1.259346000	2.250266000	1.054501000
9	-1.259345000	2.355993000	-1.671188000
9	-1.259345000	0.000002000	-3.045196000
9	-1.259345000	-2.355995000	-1.671188000
9	-1.259345000	-2.250265000	1.054502000

6.4.3 **TMSOPh**

Energy(PBE0-D3(BJ)/def2-TZVPP) = -715.754882850414

14	-1.131833000	3.413305000	-0.613902000
6	0.713010000	3.504199000	-0.865469000
1	1.204846000	3.908710000	0.033327000
1	1.130915000	2.505307000	-1.064887000
1	0.961522000	4.157906000	-1.715792000
6	-1.598070000	2.252549000	0.777609000
1	-2.691915000	2.148973000	0.854186000
1	-1.168350000	1.250859000	0.620456000
1	-1.225623000	2.638013000	1.740356000
6	-1.886318000	5.104406000	-0.342067000
1	-1.627775000	5.789773000	-1.164356000
1	-2.984092000	5.040660000	-0.279059000
1	-1.518038000	5.544001000	0.598902000
8	-1.723414000	2.777426000	-2.078698000
6	-3.020899000	2.488073000	-2.332823000
6	-3.522476000	1.208751000	-2.064974000
6	-3.863814000	3.455225000	-2.893473000
6	-4.851572000	0.905498000	-2.353200000
6	-5.191609000	3.143014000	-3.178516000
6	-5.693511000	1.869397000	-2.909045000
1	-2.853411000	0.459674000	-1.635790000
1	-3.458785000	4.446824000	-3.107355000
1	-5.232257000	-0.097116000	-2.141802000
1	-5.840467000	3.904991000	-3.618102000
1	-6.734706000	1.628107000	-3.134496000

6.4.4 **TS1**

Energy(PBE0-D3(BJ)/def2-TZVPP) = -2315.88935619146

6	2.072945000	-0.197987000	2.422904000
7	1.346337000	-0.361289000	1.272965000
6	0.277328000	-1.120755000	1.549953000
6	1.444021000	-0.880092000	3.416294000
1	1.716090000	-0.990804000	4.458604000
1	2.991143000	0.380159000	2.445944000
7	0.331074000	-1.466821000	2.862173000
6	-0.589672000	-2.385567000	3.596467000
51	-1.252993000	-1.627621000	0.094559000
7	1.341741000	-0.563364000	-1.287750000
6	0.220537000	-1.275405000	-1.467311000
6	1.409269000	-1.378333000	-3.335793000
7	0.251711000	-1.797210000	-2.723284000
1	1.672377000	-1.645731000	-4.351718000
1	3.028900000	-0.088730000	-2.531734000
6	2.082105000	-0.610666000	-2.438135000
6	-0.697653000	-2.778710000	-3.327529000
5	1.752186000	0.362142000	-0.077151000
6	-2.127702000	-2.274028000	-3.178669000
1	-2.475356000	-2.277444000	-2.136676000
1	-2.235325000	-1.253718000	-3.569738000
1	-2.804184000	-2.938243000	-3.734555000
6	-0.392684000	-2.921616000	-4.816722000
1	-0.471231000	-1.957399000	-5.340131000
1	0.600628000	-3.354670000	-5.001298000
1	-1.134211000	-3.604020000	-5.253489000
6	-0.498244000	-4.126340000	-2.633879000
1	0.539265000	-4.472083000	-2.752921000
1	-0.723863000	-4.060960000	-1.559083000
1	-1.170065000	-4.874920000	-3.078001000
6	-0.500491000	-3.773870000	2.962981000
1	-0.843804000	-3.769795000	1.918452000
1	0.535368000	-4.143598000	2.988324000
1	-1.136263000	-4.474065000	3.523443000
6	-2.008457000	-1.827058000	3.559001000
1	-2.045454000	-0.831794000	4.021529000
1	-2.415838000	-1.727283000	2.544772000
1	-2.676268000	-2.502511000	4.112330000
6	-0.149088000	-2.480376000	5.054864000
1	0.860528000	-2.903729000	5.157699000
1	-0.186469000	-1.502790000	5.556942000
1	-0.845288000	-3.150112000	5.577585000
6	1.011154000	1.784083000	-0.209838000
6	1.098724000	2.725969000	0.825878000
6	0.367250000	2.198643000	-1.383048000
6	0.583942000	4.012048000	0.694000000
6	-0.154713000	3.483085000	-1.519066000
6	-0.058545000	4.414982000	-0.481854000
1	1.590385000	2.455114000	1.765351000

1	0.266908000	1.506994000	-2.225128000
1	0.663184000	4.715723000	1.527584000
1	-0.672047000	3.761710000	-2.441821000
6	3.371783000	0.474162000	-0.098065000
6	4.165728000	-0.675000000	0.071665000
6	4.050124000	1.675633000	-0.324920000
6	5.554245000	-0.625248000	0.023881000
6	5.445714000	1.733779000	-0.374871000
6	6.224215000	0.587223000	-0.201080000
1	3.679944000	-1.641169000	0.247198000
1	3.474891000	2.594410000	-0.467804000
1	6.135950000	-1.541959000	0.163165000
1	5.939663000	2.693782000	-0.553025000
6	-0.704321000	5.762218000	-0.593965000
1	-0.261672000	6.486823000	0.104799000
1	-0.628533000	6.167344000	-1.614142000
1	-1.777079000	5.671017000	-0.354904000
6	7.723581000	0.637664000	-0.256442000
1	8.086888000	1.667113000	-0.383771000
1	8.171589000	0.229653000	0.663599000
1	8.111727000	0.036370000	-1.094632000
6	-2.694082000	0.353906000	0.359986000
6	-2.270452000	1.396267000	1.239451000
6	-3.198428000	0.879266000	-0.867383000
6	-2.487430000	2.719974000	0.931847000
6	-3.317830000	2.232784000	-1.075301000
7	-3.017374000	3.159094000	-0.190413000
9	-1.682200000	1.064188000	2.403704000
9	-3.551173000	0.009007000	-1.833349000
9	-2.162216000	3.639275000	1.838869000
9	-3.785461000	2.655007000	-2.250639000
9	-3.596804000	-0.586691000	0.989422000

6.4.5 **INT1**

Energy(PBE0-D3(BJ)/def2-TZVPP) = -2315.94811226537

6	1.703400000	0.347374000	2.620281000
7	1.101669000	-0.044308000	1.449789000
6	0.043966000	-0.829770000	1.722809000
6	1.014287000	-0.217549000	3.641260000
1	1.177473000	-0.156032000	4.712114000
1	2.578388000	0.989249000	2.635452000
7	-0.005288000	-0.955143000	3.078560000
6	-0.907894000	-1.740732000	3.963471000
51	-1.393976000	-1.643638000	0.030260000
7	1.377518000	-0.593981000	-1.052237000
6	0.415780000	-1.517541000	-1.220578000
6	1.853744000	-1.706578000	-2.894822000
7	0.719471000	-2.239176000	-2.332671000
1	2.299357000	-2.094103000	-3.802876000
1	3.131677000	-0.047221000	-2.172689000
6	2.261474000	-0.690351000	-2.094194000
6	0.040038000	-3.417539000	-2.960865000
5	1.616562000	0.504461000	0.072486000
6	-0.881104000	-2.900513000	-4.064442000
1	-1.654983000	-2.262025000	-3.617742000
1	-0.309135000	-2.332446000	-4.813777000
1	-1.361352000	-3.751334000	-4.570393000
6	1.118282000	-4.336261000	-3.544789000
1	1.639599000	-3.893621000	-4.404514000
1	1.860862000	-4.617347000	-2.782859000
1	0.630640000	-5.252710000	-3.904034000
6	-0.744731000	-4.216219000	-1.926163000
1	-0.132164000	-4.445710000	-1.040687000
1	-1.660934000	-3.684872000	-1.644489000
1	-1.047015000	-5.169282000	-2.382153000
6	-1.949172000	-2.519829000	3.179287000
1	-2.622729000	-1.857243000	2.620811000
1	-1.489392000	-3.236916000	2.486194000
1	-2.560437000	-3.087273000	3.895206000
6	-1.614976000	-0.765741000	4.908101000
1	-0.902920000	-0.223557000	5.546450000
1	-2.198785000	-0.031835000	4.335488000
1	-2.298862000	-1.321578000	5.565680000
6	-0.046443000	-2.734367000	4.748737000
1	0.486509000	-3.408178000	4.061273000
1	0.694867000	-2.229220000	5.383714000
1	-0.689602000	-3.342273000	5.401185000
6	0.882442000	1.890723000	-0.304774000
6	0.245722000	2.682287000	0.660512000
6	0.933248000	2.411026000	-1.605429000
6	-0.329809000	3.910363000	0.342930000
6	0.357856000	3.636734000	-1.932366000
6	-0.296241000	4.407209000	-0.964748000
1	0.177715000	2.326948000	1.693082000

1	1.418631000	1.837958000	-2.400532000
1	-0.836050000	4.488480000	1.122013000
1	0.399831000	3.997097000	-2.964396000
6	3.229916000	0.680796000	0.209996000
6	4.037125000	-0.424697000	0.535702000
6	3.884143000	1.904052000	0.039041000
6	5.415367000	-0.315081000	0.676196000
6	5.270406000	2.022985000	0.178944000
6	6.062231000	0.918061000	0.498251000
1	3.569165000	-1.404110000	0.683827000
1	3.298079000	2.792941000	-0.210174000
1	6.007971000	-1.199897000	0.929445000
1	5.745535000	2.998440000	0.036965000
6	-1.001023000	5.680477000	-1.325172000
1	-0.953564000	6.416350000	-0.508813000
1	-0.577894000	6.136617000	-2.231924000
1	-2.067760000	5.479478000	-1.522743000
6	7.551662000	1.032885000	0.647956000
1	8.076392000	0.394265000	-0.080979000
1	7.892304000	2.067080000	0.497743000
1	7.878827000	0.709582000	1.649264000
6	-2.050557000	0.430954000	-0.194447000
6	-2.748262000	1.037810000	0.837417000
6	-2.025426000	1.116966000	-1.402428000
6	-3.370399000	2.265957000	0.637984000
6	-2.685850000	2.340419000	-1.501813000
7	-3.341135000	2.888464000	-0.510027000
9	-2.845480000	0.448695000	2.035480000
9	-1.380776000	0.656045000	-2.462134000
9	-4.025552000	2.837164000	1.637605000
9	-2.668663000	2.991691000	-2.653938000
9	-2.460804000	-1.763344000	-1.775089000

6.4.6 **INT2**

Energy(PBE0-D3(BJ)/def2-TZVPP) = -2315.95571780954

6	-1.895863000	-0.310690000	-2.416062000
7	-1.213258000	-0.350248000	-1.225216000
6	-0.314684000	-1.343440000	-1.283633000
6	-1.414517000	-1.304246000	-3.203484000
1	-1.699773000	-1.601913000	-4.206821000
1	-2.676385000	0.418908000	-2.605408000
7	-0.436427000	-1.953669000	-2.484283000
6	0.199232000	-3.187609000	-3.032120000
51	0.972463000	-1.855894000	0.401353000
7	-1.286559000	0.082539000	1.326254000
6	-0.351844000	-0.775553000	1.747275000
6	-1.502027000	-0.115752000	3.515449000
7	-0.470813000	-0.918082000	3.087490000
1	-1.804848000	-0.041437000	4.552658000
1	-2.831637000	1.202395000	2.323280000
6	-2.008602000	0.501051000	2.415210000
6	0.317552000	-1.827156000	3.972435000
5	-1.517370000	0.726316000	-0.103947000
6	1.810700000	-1.597605000	3.739678000
1	2.143604000	-1.939310000	2.748543000
1	2.065803000	-0.534502000	3.843369000
1	2.382071000	-2.176401000	4.478704000
6	0.012606000	-1.495705000	5.430986000
1	0.266189000	-0.453744000	5.676715000
1	-1.039501000	-1.683807000	5.687885000
1	0.625652000	-2.151426000	6.064225000
6	-0.099113000	-3.268503000	3.679701000
1	-1.143653000	-3.426805000	3.988822000
1	-0.038913000	-3.514401000	2.609202000
1	0.537711000	-3.959313000	4.251393000
6	-0.910362000	-4.230110000	-3.188349000
1	-1.373947000	-4.424822000	-2.209887000
1	-1.688994000	-3.900807000	-3.891056000
1	-0.483090000	-5.168863000	-3.568881000
6	1.258101000	-3.748042000	-2.097864000
1	2.086307000	-3.040885000	-1.947965000
1	0.810242000	-4.044431000	-1.138573000
1	1.681407000	-4.649259000	-2.563202000
6	0.839916000	-2.831058000	-4.373242000
1	0.099220000	-2.490411000	-5.110463000
1	1.592565000	-2.040502000	-4.240160000
1	1.335999000	-3.720787000	-4.786299000
6	-0.559333000	2.012175000	-0.286503000
6	0.081171000	2.306607000	-1.497707000
6	-0.367653000	2.926011000	0.758773000
6	0.893907000	3.427397000	-1.647542000
6	0.444551000	4.049220000	0.618332000
6	1.104502000	4.315077000	-0.586045000
1	-0.030510000	1.629430000	-2.349859000

1	-0.844035000	2.750269000	1.727388000
1	1.397314000	3.606080000	-2.602564000
1	0.587000000	4.724189000	1.467406000
6	-3.096964000	1.083457000	-0.206915000
6	-4.063674000	0.073999000	-0.045579000
6	-3.570826000	2.373460000	-0.462081000
6	-5.424960000	0.341066000	-0.131682000
6	-4.938508000	2.649255000	-0.550300000
6	-5.890592000	1.640692000	-0.386247000
1	-3.738719000	-0.953191000	0.153208000
1	-2.854051000	3.188697000	-0.595963000
1	-6.146867000	-0.471150000	-0.000144000
1	-5.271644000	3.671983000	-0.751608000
6	2.056253000	5.465667000	-0.721301000
1	1.976305000	5.944993000	-1.708582000
1	1.883368000	6.228651000	0.051256000
1	3.096113000	5.112838000	-0.614369000
6	-7.362039000	1.925199000	-0.474651000
1	-7.870683000	1.692113000	0.474853000
1	-7.554205000	2.981439000	-0.710371000
1	-7.841592000	1.310072000	-1.252854000
6	2.078360000	0.150326000	-0.028403000
6	2.367556000	1.160323000	0.873528000
6	2.743800000	0.250347000	-1.239119000
6	3.247195000	2.185135000	0.530276000
6	3.613764000	1.302733000	-1.500245000
7	3.859182000	2.244946000	-0.625331000
9	2.555902000	-0.676976000	-2.197188000
9	1.809139000	1.194507000	2.089445000
9	3.492042000	3.154651000	1.401317000
9	4.226324000	1.379230000	-2.674482000
9	-0.504197000	-3.382486000	0.560033000

6.4.7 **TS2**

Energy(PBE0-D3(BJ)/def2-TZVPP) = -3031.69713752746

6	-1.540663000	-0.058234000	-1.913037000
7	-0.585966000	-0.391091000	-0.987152000
6	-0.109304000	-1.597165000	-1.299071000
6	-1.639522000	-1.085543000	-2.799424000
1	-2.280203000	-1.194138000	-3.665856000
1	-2.080940000	0.881948000	-1.867405000
7	-0.742148000	-2.048717000	-2.403715000
6	-0.567857000	-3.375649000	-3.071673000
51	1.320314000	-2.664710000	-0.079599000
7	0.346424000	-0.134788000	1.416471000
6	1.012100000	-1.285782000	1.579479000
6	0.779207000	-0.349642000	3.566566000
7	1.271809000	-1.446415000	2.899023000
1	0.873866000	-0.227018000	4.639712000
1	-0.302800000	1.412314000	2.761175000
6	0.201958000	0.459302000	2.642395000
6	1.978192000	-2.525641000	3.657012000
5	-0.080995000	0.641054000	0.103750000
6	3.298211000	-1.944081000	4.164861000
1	3.931169000	-1.622506000	3.327446000
1	3.128097000	-1.082920000	4.827272000
1	3.838481000	-2.711211000	4.737932000
6	1.085283000	-2.939926000	4.828666000
1	0.946501000	-2.132520000	5.560622000
1	0.097866000	-3.261805000	4.471614000
1	1.555061000	-3.783651000	5.352726000
6	2.237125000	-3.750324000	2.795273000
1	1.314331000	-4.176692000	2.372678000
1	2.963790000	-3.550131000	1.996681000
1	2.685533000	-4.524433000	3.433243000
6	-1.158558000	-4.452747000	-2.168520000
1	-0.693137000	-4.478529000	-1.173395000
1	-2.234310000	-4.280830000	-2.024765000
1	-1.021930000	-5.440299000	-2.632010000
6	0.912478000	-3.625258000	-3.354808000
1	1.354879000	-2.798946000	-3.925520000
1	1.502300000	-3.780152000	-2.438294000
1	1.009425000	-4.552970000	-3.935466000
6	-1.310519000	-3.361363000	-4.404810000
1	-2.395367000	-3.244860000	-4.271972000
1	-0.938819000	-2.570755000	-5.073353000
1	-1.144357000	-4.328497000	-4.898103000
6	1.199027000	1.444022000	-0.468824000
6	1.190152000	1.974791000	-1.766394000
6	2.352629000	1.676593000	0.292957000
6	2.279664000	2.668954000	-2.287246000
6	3.446499000	2.370545000	-0.219370000
6	3.437114000	2.870185000	-1.526694000
1	0.314811000	1.826536000	-2.405376000

1	2.416590000	1.293310000	1.315865000
1	2.240759000	3.049190000	-3.312124000
1	4.335199000	2.514552000	0.402686000
6	-1.341949000	1.577349000	0.488745000
6	-2.523766000	0.992819000	0.980625000
6	-1.323504000	2.970482000	0.379526000
6	-3.624429000	1.760650000	1.341821000
6	-2.427858000	3.748093000	0.739963000
6	-3.597282000	3.159482000	1.226992000
1	-2.579685000	-0.095882000	1.088600000
1	-0.423231000	3.466473000	0.005464000
1	-4.525144000	1.270097000	1.723804000
1	-2.377674000	4.836786000	0.642170000
6	4.647930000	3.539486000	-2.104653000
1	4.398259000	4.125967000	-3.000189000
1	5.129778000	4.206556000	-1.374188000
1	5.396799000	2.784268000	-2.397884000
6	-4.790408000	3.984005000	1.614480000
1	-5.091127000	3.785810000	2.655546000
1	-4.585219000	5.059459000	1.518104000
1	-5.660312000	3.747466000	0.980496000
6	2.976526000	-1.372515000	-0.887420000
6	3.023938000	-0.731918000	-2.118816000
6	4.148000000	-1.305620000	-0.148121000
6	4.187803000	-0.075418000	-2.519272000
6	5.261587000	-0.623987000	-0.625852000
7	5.273099000	-0.030960000	-1.791160000
9	1.985146000	-0.715956000	-2.950398000
9	4.226027000	-1.901756000	1.049095000
9	4.213391000	0.530257000	-3.694068000
9	6.359142000	-0.567912000	0.109569000
14	-1.755740000	-4.358193000	1.800009000
9	-1.008542000	-2.994428000	0.894788000
6	-2.539100000	-2.957264000	2.882242000
1	-3.015596000	-2.184959000	2.256723000
1	-3.296833000	-3.360473000	3.574465000
1	-1.780126000	-2.441363000	3.496731000
6	-1.518763000	-5.577081000	3.266271000
1	-0.696191000	-5.227901000	3.913802000
1	-2.426777000	-5.596083000	3.890509000
1	-1.274599000	-6.607900000	2.971095000
6	-3.195037000	-4.878010000	0.693851000
1	-4.010253000	-5.274673000	1.320702000
1	-3.585804000	-3.995427000	0.162753000
1	-2.940177000	-5.639207000	-0.056110000
8	-0.263663000	-5.141017000	0.967698000
6	0.101290000	-6.382325000	0.701126000
6	1.471599000	-6.653079000	0.491847000
6	-0.802034000	-7.457960000	0.566090000
6	1.913455000	-7.930060000	0.162785000
6	-0.346927000	-8.734057000	0.239344000
6	1.009278000	-8.986537000	0.033111000
1	2.181741000	-5.828951000	0.591764000

1	-1.867381000	-7.286614000	0.721039000
1	2.982265000	-8.103216000	0.006966000
1	-1.072694000	-9.546876000	0.143757000
1	1.356765000	-9.989797000	-0.223464000

6.4.8 **INT3**

Energy(PBE0-D3(BJ)/def2-TZVPP) = -3031.69815824536

6	-1.497640000	-0.069542000	-1.865253000
7	-0.552290000	-0.399726000	-0.930343000
6	-0.074976000	-1.609447000	-1.232065000
6	-1.585662000	-1.097708000	-2.752037000
1	-2.217465000	-1.207325000	-3.624796000
1	-2.039353000	0.870162000	-1.826126000
7	-0.693010000	-2.059948000	-2.347099000
6	-0.507137000	-3.379853000	-3.029178000
51	1.371545000	-2.650259000	-0.006049000
7	0.425399000	-0.079189000	1.446603000
6	1.070482000	-1.238470000	1.624340000
6	0.907890000	-0.244732000	3.589841000
7	1.357887000	-1.370354000	2.940032000
1	1.028102000	-0.099000000	4.657590000
1	-0.158084000	1.517793000	2.763010000
6	0.324453000	0.551554000	2.658883000
6	2.000784000	-2.473501000	3.717768000
5	-0.038347000	0.661824000	0.130468000
6	3.341340000	-1.955989000	4.240712000
1	4.005643000	-1.680015000	3.411325000
1	3.207092000	-1.078118000	4.889235000
1	3.829024000	-2.742873000	4.833769000
6	1.064749000	-2.816223000	4.879933000
1	1.006247000	-2.006223000	5.619693000
1	0.051459000	-3.026376000	4.511698000
1	1.440905000	-3.710473000	5.395483000
6	2.215140000	-3.719232000	2.872269000
1	1.283771000	-4.109985000	2.434732000
1	2.966274000	-3.559010000	2.086697000
1	2.617511000	-4.505815000	3.525527000
6	-1.151873000	-4.457834000	-2.168313000
1	-0.725826000	-4.500960000	-1.155576000
1	-2.230937000	-4.274243000	-2.072646000
1	-1.006916000	-5.442538000	-2.635300000
6	0.979262000	-3.652640000	-3.251192000
1	1.462691000	-2.827711000	-3.789183000
1	1.524137000	-3.840269000	-2.313210000
1	1.081881000	-4.572694000	-3.842824000
6	-1.188587000	-3.332485000	-4.394304000
1	-2.277477000	-3.210780000	-4.308708000
1	-0.780991000	-2.531982000	-5.029332000
1	-1.007506000	-4.291651000	-4.897720000
6	1.219114000	1.469757000	-0.485082000
6	1.179763000	1.981512000	-1.789658000
6	2.386239000	1.724179000	0.249130000
6	2.251406000	2.679616000	-2.341861000
6	3.461699000	2.423144000	-0.294042000
6	3.420952000	2.905056000	-1.607390000
1	0.294127000	1.815549000	-2.409623000

1	2.476281000	1.355078000	1.275119000
1	2.187534000	3.044476000	-3.371088000
1	4.360827000	2.584551000	0.308322000
6	-1.308211000	1.582620000	0.521847000
6	-2.460921000	0.984250000	1.063765000
6	-1.329591000	2.970868000	0.363897000
6	-3.573122000	1.735104000	1.424851000
6	-2.445997000	3.731220000	0.724248000
6	-3.587038000	3.129219000	1.259862000
1	-2.484249000	-0.101150000	1.211692000
1	-0.452251000	3.476585000	-0.049655000
1	-4.450662000	1.234706000	1.846016000
1	-2.427939000	4.816705000	0.587456000
6	4.611986000	3.581555000	-2.217378000
1	4.337522000	4.153688000	-3.114932000
1	5.099531000	4.263444000	-1.504660000
1	5.364419000	2.832346000	-2.516444000
6	-4.793190000	3.934694000	1.646982000
1	-5.060613000	3.769921000	2.702870000
1	-4.622356000	5.010824000	1.502852000
1	-5.671966000	3.648202000	1.046784000
6	3.007748000	-1.362253000	-0.838148000
6	3.053416000	-0.730793000	-2.074093000
6	4.174476000	-1.282304000	-0.092727000
6	4.216000000	-0.072549000	-2.475577000
6	5.285107000	-0.596232000	-0.571159000
7	5.296876000	-0.014334000	-1.742058000
9	2.016873000	-0.726834000	-2.907835000
9	4.247144000	-1.871285000	1.108065000
9	4.244951000	0.518902000	-3.657182000
9	6.378756000	-0.525720000	0.168030000
14	-1.808190000	-4.387614000	1.808655000
9	-0.935081000	-2.994558000	1.202931000
6	-2.944470000	-3.122583000	2.748347000
1	-3.381199000	-2.372906000	2.065883000
1	-3.777742000	-3.634884000	3.260044000
1	-2.382821000	-2.565809000	3.520089000
6	-1.381646000	-5.433909000	3.341874000
1	-0.675520000	-4.873097000	3.974123000
1	-2.287186000	-5.591482000	3.949602000
1	-0.921484000	-6.407854000	3.125449000
6	-3.105839000	-5.047029000	0.600520000
1	-3.943668000	-5.468376000	1.180794000
1	-3.514032000	-4.195994000	0.031008000
1	-2.759681000	-5.807391000	-0.111845000
8	-0.290519000	-5.176784000	0.900428000
6	0.023004000	-6.418328000	0.600164000
6	1.300988000	-6.679984000	0.050136000
6	-0.840560000	-7.525582000	0.770494000
6	1.688242000	-7.964133000	-0.314122000
6	-0.439294000	-8.809266000	0.403429000
6	0.821503000	-9.046816000	-0.143157000
1	1.982963000	-5.835855000	-0.080196000

1	-1.833002000	-7.375107000	1.195519000
1	2.684685000	-8.123316000	-0.736981000
1	-1.133721000	-9.641798000	0.550365000
1	1.125473000	-10.056534000	-0.428102000

6.4.9 **INT4**

Energy(PBE0-D3(BJ)/def2-TZVPP) = -2522.78207029698

6	-1.668958000	-0.868057000	-2.207096000
7	-0.948976000	-0.691833000	-1.055148000
6	0.124706000	-1.497886000	-1.095800000
6	-1.028882000	-1.799255000	-2.958091000
1	-1.283411000	-2.209338000	-3.929160000
1	-2.585391000	-0.320666000	-2.401022000
7	0.083870000	-2.194327000	-2.256033000
6	0.991887000	-3.232532000	-2.833084000
51	1.529789000	-1.652108000	0.578021000
7	-1.043752000	-0.112141000	1.456670000
6	0.040510000	-0.760357000	1.903093000
6	-1.173532000	-0.186419000	3.658342000
7	-0.022175000	-0.819257000	3.254867000
1	-1.464274000	-0.091696000	4.697072000
1	-2.740017000	0.789626000	2.426007000
6	-1.805399000	0.249725000	2.537240000
6	0.933151000	-1.480205000	4.198450000
5	-1.391775000	0.404941000	-0.000991000
6	2.363681000	-1.070765000	3.853764000
1	2.704864000	-1.493245000	2.897165000
1	2.463471000	0.021729000	3.812764000
1	3.041645000	-1.463112000	4.624287000
6	0.638263000	-1.002572000	5.619098000
1	0.714630000	0.091326000	5.707791000
1	-0.350710000	-1.327784000	5.971630000
1	1.384684000	-1.449745000	6.289385000
6	0.736829000	-2.992208000	4.114690000
1	-0.286751000	-3.259693000	4.418547000
1	0.892351000	-3.373545000	3.094419000
1	1.440476000	-3.489274000	4.798479000
6	0.152183000	-4.479823000	-3.114575000
1	-0.309894000	-4.853811000	-2.190030000
1	-0.638791000	-4.289944000	-3.853312000
1	0.803918000	-5.266386000	-3.520841000
6	2.115699000	-3.600099000	-1.878730000
1	2.794017000	-2.753534000	-1.702739000
1	1.732175000	-3.992044000	-0.925365000
1	2.712374000	-4.393377000	-2.349773000
6	1.581511000	-2.659152000	-4.122697000
1	0.801280000	-2.432016000	-4.863336000
1	2.146138000	-1.739883000	-3.914370000
1	2.264358000	-3.396184000	-4.568942000
6	-0.620443000	1.792248000	-0.289930000
6	-0.035142000	2.083873000	-1.528846000
6	-0.575755000	2.804648000	0.677937000
6	0.589641000	3.303364000	-1.778458000
6	0.050115000	4.025658000	0.438636000
6	0.657119000	4.295227000	-0.793155000
1	-0.043150000	1.330863000	-2.322885000

1	-1.023642000	2.634788000	1.661176000
1	1.055055000	3.483528000	-2.752277000
1	0.084568000	4.781470000	1.228700000
6	-3.004710000	0.566378000	-0.110376000
6	-3.877518000	-0.478158000	0.245905000
6	-3.599467000	1.729586000	-0.611812000
6	-5.256660000	-0.367988000	0.107305000
6	-4.984250000	1.847294000	-0.756837000
6	-5.839880000	0.802103000	-0.401424000
1	-3.471537000	-1.409786000	0.649808000
1	-2.964068000	2.570809000	-0.901533000
1	-5.897471000	-1.206165000	0.398231000
1	-5.408140000	2.774159000	-1.155083000
6	1.409569000	5.569712000	-1.032801000
1	1.264895000	5.938831000	-2.059141000
1	1.103653000	6.359346000	-0.331666000
1	2.491906000	5.404247000	-0.896400000
6	-7.329118000	0.917780000	-0.549138000
1	-7.833846000	0.834292000	0.426986000
1	-7.615792000	1.880040000	-0.996263000
1	-7.730374000	0.113147000	-1.185761000
6	2.285881000	0.467040000	0.049744000
6	2.397083000	1.559438000	0.894195000
6	2.951904000	0.599958000	-1.157704000
6	3.111467000	2.688164000	0.496530000
6	3.651026000	1.758751000	-1.473684000
7	3.728146000	2.776582000	-0.654403000
9	2.927713000	-0.400817000	-2.056464000
9	1.819425000	1.576631000	2.099747000
9	3.182540000	3.732708000	1.309002000
9	4.265507000	1.861381000	-2.643692000
8	0.521656000	-3.649087000	1.018845000
6	-0.522849000	-4.303443000	0.567052000
6	-1.757341000	-3.676154000	0.286347000
6	-0.442055000	-5.696672000	0.329180000
6	-2.830657000	-4.388178000	-0.240791000
6	-1.524136000	-6.403768000	-0.186147000
6	-2.726518000	-5.758328000	-0.488940000
1	-1.851147000	-2.610164000	0.490993000
1	0.500835000	-6.201262000	0.556193000
1	-3.764745000	-3.860599000	-0.457257000
1	-1.425196000	-7.479058000	-0.362385000
1	-3.569517000	-6.316360000	-0.902396000

6.4.10 **INT5**

Energy(PBE0-D3(BJ)/def2-TZVPP) = -2522.79235810919

6	-1.495834000	-0.810906000	-2.172400000
7	-0.723537000	-0.543911000	-1.073146000
6	0.294922000	-1.424511000	-1.048794000
6	-0.965242000	-1.884979000	-2.809106000
1	-1.302109000	-2.400152000	-3.700393000
1	-2.387859000	-0.238107000	-2.403228000
7	0.132922000	-2.283578000	-2.088047000
6	0.911265000	-3.499535000	-2.488710000
51	1.940562000	-1.279704000	0.402196000
7	-0.752002000	0.266461000	1.369553000
6	0.241368000	-0.534240000	1.776762000
6	-0.932314000	0.095948000	3.560144000
7	0.127542000	-0.668593000	3.124356000
1	-1.231557000	0.165671000	4.599002000
1	-2.339852000	1.331317000	2.367625000
6	-1.478238000	0.677255000	2.459570000
6	0.903668000	-1.605638000	3.987548000
5	-1.164658000	0.645221000	-0.098473000
6	2.370972000	-1.188136000	4.041395000
1	2.863274000	-1.264775000	3.062563000
1	2.470850000	-0.153552000	4.396706000
1	2.909795000	-1.850962000	4.733605000
6	0.336475000	-1.575064000	5.404580000
1	0.437267000	-0.582057000	5.866754000
1	-0.719666000	-1.880506000	5.432594000
1	0.906844000	-2.285479000	6.018281000
6	0.746332000	-3.019168000	3.425409000
1	-0.316409000	-3.296477000	3.365569000
1	1.189406000	-3.111045000	2.423667000
1	1.259033000	-3.737014000	4.081875000
6	-0.074779000	-4.542775000	-3.024416000
1	-0.865386000	-4.760076000	-2.290395000
1	-0.540502000	-4.243684000	-3.973175000
1	0.479852000	-5.471048000	-3.218361000
6	1.622737000	-4.111726000	-1.288131000
1	2.478575000	-3.504190000	-0.973321000
1	0.929166000	-4.269802000	-0.447847000
1	2.021323000	-5.092104000	-1.583687000
6	1.899275000	-3.086847000	-3.577044000
1	1.364784000	-2.682085000	-4.449688000
1	2.591422000	-2.332213000	-3.179252000
1	2.475917000	-3.964732000	-3.904577000
6	-0.527072000	2.048488000	-0.565738000
6	-0.482719000	2.411607000	-1.919376000
6	-0.082511000	3.007053000	0.353930000
6	0.005479000	3.647569000	-2.335479000
6	0.406181000	4.246541000	-0.053900000
6	0.473051000	4.586757000	-1.409301000
1	-0.818168000	1.704459000	-2.682887000

1	-0.093836000	2.777958000	1.423920000
1	0.044719000	3.882643000	-3.403169000
1	0.768533000	4.957633000	0.694680000
6	-2.792879000	0.677298000	-0.116713000
6	-3.528174000	-0.462521000	0.257253000
6	-3.530911000	1.810268000	-0.470293000
6	-4.917616000	-0.470779000	0.271135000
6	-4.929199000	1.810663000	-0.456972000
6	-5.648944000	0.672514000	-0.087738000
1	-2.993979000	-1.374464000	0.546075000
1	-3.002710000	2.721107000	-0.765510000
1	-5.453004000	-1.378958000	0.565801000
1	-5.471543000	2.717797000	-0.740450000
6	1.097348000	5.874885000	-1.854638000
1	0.683594000	6.217592000	-2.814156000
1	0.960426000	6.672232000	-1.109759000
1	2.183332000	5.738211000	-1.993440000
6	-7.150008000	0.662091000	-0.066154000
1	-7.532676000	0.486546000	0.952436000
1	-7.563891000	1.616091000	-0.421984000
1	-7.553418000	-0.142301000	-0.701733000
6	2.411527000	0.815960000	-0.002084000
6	2.900875000	1.614986000	1.018724000
6	2.484749000	1.347275000	-1.284225000
6	3.445114000	2.862910000	0.731985000
6	3.048108000	2.609047000	-1.465441000
7	3.520176000	3.334049000	-0.483406000
9	2.024237000	0.698539000	-2.341717000
9	2.875107000	1.193875000	2.285281000
9	3.907497000	3.614312000	1.719536000
9	3.123755000	3.111647000	-2.686834000
8	3.399032000	-1.504507000	-1.312797000
6	4.617767000	-1.866852000	-0.967016000
6	5.332586000	-1.223173000	0.071924000
6	5.265161000	-2.941302000	-1.622550000
6	6.611225000	-1.640797000	0.436601000
6	6.545243000	-3.343103000	-1.257068000
6	7.230898000	-2.703577000	-0.220141000
1	4.880572000	-0.364158000	0.574044000
1	4.735203000	-3.448987000	-2.432142000
1	7.133694000	-1.117273000	1.242682000
1	7.017152000	-4.174541000	-1.789100000
1	8.235495000	-3.024833000	0.064229000

6.4.11 **TMSF**

Energy(PBE0-D3(BJ)/def2-TZVPP) = -508.926216960041

9	-1.462446000	2.458082000	-2.058446000
14	-1.124342000	3.334899000	-0.704330000
6	0.717226000	3.632185000	-0.700130000
1	1.011530000	4.217845000	0.185673000
1	1.270478000	2.680263000	-0.676935000
1	1.030597000	4.190904000	-1.595848000
6	-1.669519000	2.303104000	0.751058000
1	-2.749429000	2.092621000	0.703319000
1	-1.132629000	1.341947000	0.776688000
1	-1.467552000	2.830894000	1.696991000
6	-2.090649000	4.925043000	-0.835562000
1	-1.794975000	5.495091000	-1.730235000
1	-3.172245000	4.726937000	-0.895329000
1	-1.909498000	5.559872000	0.046716000

6.4.12 **TS4**

Energy(PBE0-D3(BJ)/def2-SVP) = -2522.73226253801

6	1.680619000	0.382710000	2.458439000
7	1.307080000	-0.143925000	1.251826000
6	0.372822000	-1.084632000	1.469865000
6	0.979952000	-0.256732000	3.429396000
1	1.001324000	-0.122899000	4.504537000
1	2.435111000	1.158559000	2.537994000
7	0.183595000	-1.190181000	2.810225000
6	-0.754730000	-2.040998000	3.604094000
51	-0.724338000	-2.024772000	-0.160437000
7	1.928808000	-0.672285000	-1.183116000
6	0.974450000	-1.563428000	-1.477427000
6	2.554675000	-1.702022000	-3.027121000
7	1.351380000	-2.217953000	-2.609170000
1	3.070289000	-2.045947000	-3.915276000
1	3.777860000	-0.088819000	-2.110973000
6	2.908102000	-0.737576000	-2.135332000
6	0.660618000	-3.363965000	-3.271212000
5	1.883384000	0.465745000	-0.099529000
6	-0.800563000	-3.003586000	-3.513097000
1	-1.378317000	-2.908448000	-2.585324000
1	-0.888756000	-2.058954000	-4.068252000
1	-1.278735000	-3.800133000	-4.100229000
6	1.320363000	-3.640057000	-4.619861000
1	1.283368000	-2.760546000	-5.279425000
1	2.363691000	-3.970039000	-4.514619000
1	0.767012000	-4.452168000	-5.110488000
6	0.801400000	-4.597784000	-2.379576000
1	1.862637000	-4.831276000	-2.207742000
1	0.313287000	-4.451728000	-1.404897000
1	0.328432000	-5.461840000	-2.868032000
6	-1.177458000	-3.281105000	2.828882000
1	-1.873877000	-3.040667000	2.015599000
1	-0.311984000	-3.834065000	2.435211000
1	-1.719702000	-3.944404000	3.517067000
6	-1.975127000	-1.190385000	3.954168000
1	-1.684176000	-0.311030000	4.547641000
1	-2.475037000	-0.851119000	3.037663000
1	-2.683538000	-1.788279000	4.545991000
6	-0.027881000	-2.501454000	4.870277000
1	0.901489000	-3.035815000	4.622274000
1	0.210945000	-1.672838000	5.550496000
1	-0.685689000	-3.189093000	5.419225000
6	0.940705000	1.657996000	-0.636887000
6	0.712557000	2.793101000	0.155026000
6	0.375240000	1.666738000	-1.918709000
6	-0.039057000	3.870260000	-0.302965000
6	-0.385672000	2.738359000	-2.381367000
6	-0.612631000	3.860136000	-1.579625000
1	1.132220000	2.843036000	1.164237000

1	0.524155000	0.815323000	-2.589351000
1	-0.206483000	4.731270000	0.350218000
1	-0.827492000	2.696729000	-3.381285000
6	3.418533000	0.909485000	0.182597000
6	4.327032000	-0.002473000	0.750222000
6	3.930643000	2.160015000	-0.177876000
6	5.664216000	0.318909000	0.954813000
6	5.273348000	2.490630000	0.024257000
6	6.164707000	1.579754000	0.596428000
1	3.976168000	-0.999152000	1.039363000
1	3.265387000	2.898484000	-0.633314000
1	6.338903000	-0.419703000	1.399007000
1	5.635997000	3.479570000	-0.272142000
6	-1.507658000	4.969529000	-2.041871000
1	-1.336515000	5.894727000	-1.472959000
1	-1.369517000	5.185435000	-3.111983000
1	-2.560452000	4.673478000	-1.899482000
6	7.604417000	1.931962000	0.834714000
1	8.277635000	1.147856000	0.454474000
1	7.872758000	2.880187000	0.347861000
1	7.813166000	2.038389000	1.912127000
6	-2.322185000	-0.331477000	-0.237594000
6	-2.191504000	0.955083000	0.376873000
6	-2.960376000	-0.222463000	-1.509163000
6	-2.819508000	2.059183000	-0.144728000
6	-3.492847000	0.962802000	-1.960103000
7	-3.470265000	2.096972000	-1.294219000
9	-1.572330000	1.041044000	1.569351000
9	-3.067907000	-1.344222000	-2.257239000
9	-2.802065000	3.191407000	0.559994000
9	-4.080946000	0.979534000	-3.156586000
8	-3.025993000	-1.334165000	0.803718000
6	-4.340288000	-1.604522000	0.646231000
6	-5.295213000	-0.590206000	0.487198000
6	-4.752627000	-2.943730000	0.660151000
6	-6.640756000	-0.924122000	0.343845000
6	-6.102030000	-3.263605000	0.536568000
6	-7.053842000	-2.256112000	0.371642000
1	-4.984010000	0.455420000	0.489384000
1	-3.996847000	-3.725103000	0.770024000
1	-7.376498000	-0.125720000	0.217233000
1	-6.411793000	-4.311796000	0.559279000
1	-8.111505000	-2.507809000	0.265750000

6.4.13 **PyrF4OPh (2n)**

Energy(PBE0-D3(BJ)/def2-TZVPP) = -950.8909678204

6	-0.457157000	0.220025000	-1.725571000
6	-0.164682000	1.580463000	-1.868788000
6	0.544487000	2.211793000	-0.851473000
7	0.938156000	1.599647000	0.235464000
6	0.668088000	0.328588000	0.385488000
6	-0.017331000	-0.424092000	-0.562437000
9	0.828677000	3.493693000	-0.975304000
9	-0.553509000	2.236557000	-2.945329000
9	-0.249505000	-1.711581000	-0.376696000
9	1.082366000	-0.265608000	1.488008000
8	-1.084195000	-0.411342000	-2.726657000
6	-2.192956000	-1.195401000	-2.478102000
6	-2.450739000	-2.210851000	-3.394529000
6	-3.045697000	-0.954292000	-1.404141000
6	-3.581675000	-3.004956000	-3.224087000
6	-4.170446000	-1.762111000	-1.243620000
6	-4.442846000	-2.787487000	-2.147875000
1	-1.760892000	-2.364551000	-4.226245000
1	-2.841314000	-0.142222000	-0.702889000
1	-3.787787000	-3.804677000	-3.939281000
1	-4.842129000	-1.579114000	-0.401682000
1	-5.326519000	-3.415211000	-2.015525000

6.5 Mechanism B (Redox-neutral S_NAr without ligand scrambling) – xyz coordinates (in Å) and Energies (in Hartree)

6.5.1 **TS5**

Energy(PBE0-D3(BJ)/def2-TZVPP) = -3266.81820067654

6	-7.908107000	-2.779627000	4.287773000
7	-7.262999000	-2.457340000	5.449353000
6	-6.016562000	-2.950540000	5.398846000
6	-7.042608000	-3.487337000	3.517013000
1	-7.178919000	-3.912191000	2.528834000
1	-8.934073000	-2.480871000	4.100515000
7	-5.866865000	-3.601429000	4.217298000
6	-4.690172000	-4.280700000	3.585628000
51	-4.657901000	-2.812886000	7.109421000
7	-7.506522000	-1.896819000	7.951632000
6	-6.308976000	-2.262626000	8.428150000
6	-7.656498000	-1.948183000	10.148951000
7	-6.381485000	-2.302567000	9.781073000
1	-7.972851000	-1.897143000	11.185083000
1	-9.387080000	-1.404951000	8.869474000
6	-8.352708000	-1.700895000	9.009430000
6	-5.337785000	-2.530395000	10.828995000
5	-7.915445000	-1.456607000	6.484100000
6	-4.032414000	-3.006201000	10.216475000
1	-4.151963000	-3.948030000	9.665280000
1	-3.572570000	-2.232577000	9.581276000
1	-3.320599000	-3.200762000	11.030527000
6	-5.109183000	-1.203949000	11.557036000
1	-4.747998000	-0.431308000	10.866693000
1	-6.029921000	-0.841824000	12.036509000
1	-4.356043000	-1.352786000	12.343944000
6	-5.857531000	-3.585581000	11.806251000
1	-6.774613000	-3.259381000	12.315475000
1	-6.042672000	-4.541105000	11.301188000
1	-5.094881000	-3.757207000	12.578726000
6	-5.148664000	-5.639748000	3.053607000
1	-5.527917000	-6.274755000	3.863618000
1	-5.915093000	-5.548600000	2.272199000
1	-4.283880000	-6.149152000	2.606426000
6	-3.566043000	-4.500466000	4.583874000
1	-3.104996000	-3.557196000	4.904593000
1	-3.892151000	-5.089600000	5.449804000
1	-2.776212000	-5.077696000	4.083764000
6	-4.214656000	-3.393236000	2.433730000
1	-4.995046000	-3.276363000	1.667544000
1	-3.926963000	-2.397987000	2.795794000
1	-3.339632000	-3.857236000	1.956325000
6	-7.369711000	0.036699000	6.203726000
6	-6.775359000	0.414916000	4.992656000
6	-7.542938000	1.047768000	7.159249000

6	-6.349082000	1.720621000	4.758509000
6	-7.118713000	2.354870000	6.934661000
6	-6.502030000	2.715961000	5.730808000
1	-6.621161000	-0.328963000	4.204626000
1	-8.009517000	0.810309000	8.119771000
1	-5.874509000	1.972154000	3.805043000
1	-7.255771000	3.110691000	7.713351000
6	-9.521374000	-1.579178000	6.353055000
6	-10.156657000	-2.803949000	6.621448000
6	-10.339892000	-0.517630000	5.955533000
6	-11.532345000	-2.959170000	6.503389000
6	-11.724357000	-0.666782000	5.834330000
6	-12.347013000	-1.887678000	6.106496000
1	-9.557954000	-3.662923000	6.933603000
1	-9.889265000	0.453510000	5.732884000
1	-11.985447000	-3.931095000	6.722641000
1	-12.333944000	0.186063000	5.520928000
6	-5.973359000	4.100932000	5.507793000
1	-6.141927000	4.436397000	4.473570000
1	-6.438580000	4.826623000	6.189939000
1	-4.884408000	4.127761000	5.684176000
6	-13.833495000	-2.057476000	5.985572000
1	-14.283744000	-2.319476000	6.956742000
1	-14.316664000	-1.137969000	5.626338000
1	-14.086492000	-2.871047000	5.287113000
6	-4.195064000	-0.655078000	6.700546000
6	-4.391376000	0.408448000	7.569586000
6	-3.472515000	-0.350979000	5.556117000
6	-3.896673000	1.672506000	7.250874000
6	-3.014774000	0.940541000	5.320491000
7	-3.221816000	1.922254000	6.159471000
9	-3.209119000	-1.300827000	4.651189000
9	-5.050473000	0.266102000	8.717156000
9	-4.109247000	2.674869000	8.085440000
9	-2.344722000	1.204300000	4.212074000
8	-5.778453000	-5.216767000	7.528504000
6	-7.107288000	-5.488476000	7.514945000
6	-7.858689000	-5.384706000	8.690087000
6	-7.721216000	-5.932158000	6.338947000
6	-9.207167000	-5.734748000	8.690124000
6	-9.069535000	-6.282008000	6.344404000
6	-9.814238000	-6.194213000	7.521072000
1	-7.364647000	-5.044661000	9.600579000
1	-7.120087000	-6.017216000	5.433046000
1	-9.785110000	-5.656756000	9.614086000
1	-9.539455000	-6.635013000	5.423377000
1	-10.868943000	-6.478457000	7.525553000
6	-4.883757000	-6.508313000	7.907068000
6	-5.156608000	-6.971185000	9.240585000
6	-5.786250000	-8.169140000	9.473446000
7	-6.078171000	-9.064986000	8.553871000
6	-5.717209000	-8.743096000	7.328959000
6	-5.084419000	-7.578720000	6.967484000

9	-6.109932000	-8.469529000	10.730775000
9	-4.902450000	-6.118561000	10.256067000
9	-3.678965000	-5.841313000	7.773790000
9	-4.765086000	-7.342288000	5.677669000
9	-5.968142000	-9.637928000	6.374245000

6.5.2 **INT6**

Energy(PBE0-D3(BJ)/def2-TZVPP) = -3266.8183930243

6	-7.915232000	-2.793290000	4.298656000
7	-7.267523000	-2.462749000	5.456134000
6	-6.018219000	-2.948791000	5.402551000
6	-7.048626000	-3.499415000	3.527384000
1	-7.186456000	-3.928813000	2.541431000
1	-8.943545000	-2.500986000	4.114036000
7	-5.869444000	-3.603600000	4.222928000
6	-4.688278000	-4.270596000	3.585941000
51	-4.655058000	-2.801197000	7.109333000
7	-7.506703000	-1.896334000	7.955845000
6	-6.307741000	-2.260867000	8.430238000
6	-7.652428000	-1.949690000	10.153393000
7	-6.377798000	-2.302426000	9.783333000
1	-7.967124000	-1.900050000	11.190090000
1	-9.385957000	-1.407056000	8.877369000
6	-8.350983000	-1.701952000	9.015192000
6	-5.330632000	-2.526268000	10.829314000
5	-7.917193000	-1.457591000	6.488042000
6	-4.031568000	-3.018367000	10.215926000
1	-4.159983000	-3.961623000	9.669038000
1	-3.565192000	-2.251772000	9.576668000
1	-3.319552000	-3.214819000	11.029358000
6	-5.089016000	-1.194182000	11.542693000
1	-4.719672000	-0.432372000	10.844865000
1	-6.006631000	-0.818061000	12.017454000
1	-4.337954000	-1.342375000	12.331723000
6	-5.854807000	-3.565667000	11.821092000
1	-6.757317000	-3.218470000	12.342341000
1	-6.066021000	-4.520518000	11.325250000
1	-5.083899000	-3.746134000	12.583233000
6	-5.140393000	-5.624135000	3.034703000
1	-5.533832000	-6.265283000	3.832837000
1	-5.893329000	-5.524740000	2.241247000
1	-4.269216000	-6.130654000	2.596831000
6	-3.566164000	-4.498441000	4.584797000
1	-3.107631000	-3.557555000	4.916178000
1	-3.891214000	-5.097776000	5.444218000
1	-2.773215000	-5.067032000	4.079735000
6	-4.213952000	-3.366357000	2.446635000
1	-4.994024000	-3.242346000	1.681209000
1	-3.930144000	-2.375005000	2.822115000
1	-3.336609000	-3.821097000	1.964595000
6	-7.367553000	0.033103000	6.201928000
6	-6.767044000	0.404374000	4.991751000
6	-7.545404000	1.049444000	7.151007000
6	-6.339383000	1.708741000	4.752377000
6	-7.119749000	2.355114000	6.921270000
6	-6.497177000	2.709472000	5.718335000
1	-6.609629000	-0.343766000	4.208373000

1	-8.017152000	0.817153000	8.110280000
1	-5.860550000	1.955019000	3.799684000
1	-7.260732000	3.115354000	7.694920000
6	-9.523365000	-1.575494000	6.357921000
6	-10.162948000	-2.796777000	6.631669000
6	-10.338012000	-0.513835000	5.952631000
6	-11.538802000	-2.948789000	6.511313000
6	-11.722597000	-0.659630000	5.829257000
6	-12.349453000	-1.877202000	6.106636000
1	-9.567337000	-3.655650000	6.949808000
1	-9.884214000	0.454678000	5.725158000
1	-11.995245000	-3.918190000	6.734630000
1	-12.328941000	0.193222000	5.509686000
6	-5.968100000	4.093444000	5.490205000
1	-6.130062000	4.422316000	4.452829000
1	-6.438493000	4.822921000	6.164719000
1	-4.880370000	4.122581000	5.673667000
6	-13.836092000	-2.043416000	5.983002000
1	-14.289561000	-2.297638000	6.954734000
1	-14.315606000	-1.124902000	5.616441000
1	-14.089652000	-2.860866000	5.289314000
6	-4.189316000	-0.649292000	6.706325000
6	-4.399837000	0.415398000	7.570933000
6	-3.452203000	-0.347063000	5.570509000
6	-3.903876000	1.679764000	7.255003000
6	-2.995928000	0.945463000	5.337274000
7	-3.216606000	1.928405000	6.171224000
9	-3.173028000	-1.299166000	4.673415000
9	-5.072438000	0.274477000	8.710036000
9	-4.128224000	2.683095000	8.084871000
9	-2.312528000	1.208212000	4.237196000
8	-5.809448000	-5.254014000	7.532928000
6	-7.145612000	-5.521815000	7.518950000
6	-7.893293000	-5.407925000	8.693278000
6	-7.758510000	-5.952304000	6.339817000
6	-9.246507000	-5.739782000	8.689682000
6	-9.111742000	-6.283554000	6.342253000
6	-9.856609000	-6.188518000	7.518120000
1	-7.394410000	-5.076383000	9.604244000
1	-7.155273000	-6.043030000	5.435866000
1	-9.825556000	-5.655568000	9.612307000
1	-9.585115000	-6.627238000	5.419561000
1	-10.915087000	-6.458387000	7.519904000
6	-4.939216000	-6.485725000	7.895041000
6	-5.185455000	-6.971547000	9.232358000
6	-5.709630000	-8.215694000	9.475013000
7	-5.942918000	-9.137073000	8.562874000
6	-5.630902000	-8.784416000	7.332159000
6	-5.102613000	-7.574216000	6.959313000
9	-5.992445000	-8.536832000	10.738267000
9	-4.995729000	-6.095842000	10.245641000
9	-3.715596000	-5.816730000	7.768721000
9	-4.834866000	-7.313596000	5.659746000

9	-5.832483000	-9.695441000	6.379464000

6.5.3 **TS6**

Energy(PBE0-D3(BJ)/def2-TZVPP) = -3266.81820067654

6	-7.792892000	-3.119019000	4.403854000
7	-7.147539000	-2.620014000	5.498778000
6	-5.849057000	-2.948957000	5.416289000
6	-6.872155000	-3.765797000	3.639209000
1	-6.993873000	-4.285020000	2.696028000
1	-8.857241000	-2.973727000	4.250056000
7	-5.662245000	-3.661118000	4.275267000
6	-4.397451000	-4.103504000	3.603638000
51	-4.491821000	-2.640933000	7.106075000
7	-7.396888000	-1.907108000	7.947297000
6	-6.193217000	-2.262166000	8.415872000
6	-7.521392000	-1.934525000	10.146596000
7	-6.252665000	-2.300554000	9.767260000
1	-7.829296000	-1.882486000	11.185246000
1	-9.266792000	-1.405859000	8.881384000
6	-8.229321000	-1.695215000	9.012240000
6	-5.209048000	-2.579825000	10.801123000
5	-7.827117000	-1.564729000	6.460494000
6	-3.926374000	-3.099558000	10.176019000
1	-4.094876000	-4.004749000	9.574752000
1	-3.412911000	-2.326324000	9.582092000
1	-3.235769000	-3.368604000	10.987233000
6	-4.932261000	-1.273013000	11.545305000
1	-4.582819000	-0.495287000	10.852883000
1	-5.830629000	-0.901179000	12.058775000
1	-4.156243000	-1.444979000	12.304777000
6	-5.751961000	-3.646810000	11.752242000
1	-6.670121000	-3.323261000	12.261167000
1	-5.946334000	-4.583624000	11.214539000
1	-4.998245000	-3.847240000	12.526411000
6	-4.658654000	-5.386643000	2.812928000
1	-5.027144000	-6.191459000	3.459787000
1	-5.361010000	-5.233898000	1.982177000
1	-3.706669000	-5.707207000	2.368039000
6	-3.305328000	-4.378244000	4.627271000
1	-2.893263000	-3.454170000	5.055664000
1	-3.658136000	-5.041950000	5.428746000
1	-2.468314000	-4.874536000	4.117445000
6	-3.991006000	-2.982998000	2.644487000
1	-4.760112000	-2.833303000	1.872116000
1	-3.841331000	-2.037350000	3.180041000
1	-3.049422000	-3.250438000	2.143201000
6	-7.296889000	-0.087961000	6.074184000
6	-6.634953000	0.207314000	4.876297000
6	-7.559511000	0.993328000	6.928424000
6	-6.220542000	1.501540000	4.563136000
6	-7.149347000	2.287769000	6.625464000
6	-6.457528000	2.566439000	5.438793000
1	-6.420269000	-0.591056000	4.158537000

1	-8.089985000	0.820984000	7.869421000
1	-5.693773000	1.688275000	3.622395000
1	-7.358644000	3.101156000	7.326178000
6	-9.433049000	-1.699430000	6.327643000
6	-10.107708000	-2.854963000	6.760443000
6	-10.216275000	-0.702308000	5.734382000
6	-11.482438000	-3.002228000	6.613017000
6	-11.597391000	-0.844573000	5.581918000
6	-12.257674000	-1.995546000	6.019519000
1	-9.543344000	-3.666950000	7.224405000
1	-9.737719000	0.213786000	5.378006000
1	-11.963128000	-3.919896000	6.964508000
1	-12.174196000	-0.041910000	5.112568000
6	-5.947746000	3.944759000	5.142772000
1	-5.934720000	4.148285000	4.062007000
1	-6.556407000	4.716045000	5.636357000
1	-4.912035000	4.056276000	5.507849000
6	-13.742278000	-2.157300000	5.869182000
1	-14.234417000	-2.239748000	6.851847000
1	-14.189237000	-1.304954000	5.338392000
1	-13.988867000	-3.074584000	5.311238000
6	-4.126567000	-0.472313000	6.800285000
6	-4.477078000	0.565286000	7.653034000
6	-3.260817000	-0.129688000	5.770795000
6	-4.004599000	1.854017000	7.403316000
6	-2.839676000	1.184016000	5.602354000
7	-3.207685000	2.145781000	6.409028000
9	-2.817862000	-1.065609000	4.924455000
9	-5.257017000	0.378616000	8.712772000
9	-4.372470000	2.837677000	8.204342000
9	-2.035270000	1.488927000	4.600113000
8	-6.527773000	-5.214592000	7.843398000
6	-7.840856000	-5.572956000	7.989842000
6	-8.467803000	-5.322590000	9.209695000
6	-8.559364000	-6.129741000	6.929114000
6	-9.818839000	-5.623526000	9.369466000
6	-9.905988000	-6.442801000	7.102697000
6	-10.540147000	-6.191735000	8.319623000
1	-7.883539000	-4.892506000	10.023050000
1	-8.061823000	-6.296084000	5.974006000
1	-10.308172000	-5.419221000	10.324776000
1	-10.467145000	-6.877688000	6.272186000
1	-11.597161000	-6.435619000	8.448599000
6	-5.586878000	-6.295291000	7.777872000
6	-5.523364000	-7.136588000	8.951931000
6	-5.412822000	-8.500882000	8.864472000
7	-5.418176000	-9.201146000	7.745846000
6	-5.559200000	-8.489240000	6.647610000
6	-5.677022000	-7.124430000	6.600822000
9	-5.311896000	-9.194626000	9.999462000
9	-5.525594000	-6.518940000	10.154462000
9	-4.375379000	-5.409203000	7.701167000
9	-5.870375000	-6.482617000	5.422285000

9	-5.609123000	-9.161863000	5.495247000

6.6 Mechanism C (Redox-neutral S_NAr after ligand scrambling) – xyz coordinates (in Å) and Energies (in Hartree)

6.6.1 **INT7**

Energy(PBE0-D3(BJ)/def2-TZVPP) = -2185.19324927852

6	-1.570224000	-0.814223000	-2.167366000
7	-0.878776000	-0.672768000	-0.991854000
6	0.176332000	-1.501517000	-1.017702000
6	-0.925337000	-1.738913000	-2.922178000
1	-1.158077000	-2.123861000	-3.909080000
1	-2.472316000	-0.248101000	-2.374351000
7	0.164662000	-2.161637000	-2.197861000
6	1.105588000	-3.162058000	-2.778545000
51	1.587671000	-1.624920000	0.656666000
7	-1.005426000	-0.083740000	1.516394000
6	0.027367000	-0.798705000	1.984815000
6	-1.247406000	-0.227876000	3.702984000
7	-0.106081000	-0.899575000	3.333581000
1	-1.586319000	-0.156913000	4.728955000
1	-2.701330000	0.868375000	2.434363000
6	-1.800980000	0.278284000	2.570427000
6	0.700978000	-1.701621000	4.307230000
5	-1.307339000	0.442783000	0.052430000
6	2.191448000	-1.564487000	4.016202000
1	2.488539000	-2.048829000	3.076146000
1	2.505314000	-0.511541000	3.987967000
1	2.752439000	-2.069197000	4.814853000
6	0.447253000	-1.167566000	5.716980000
1	0.665052000	-0.091594000	5.790871000
1	-0.580833000	-1.352612000	6.057934000
1	1.116318000	-1.698513000	6.407518000
6	0.253970000	-3.159116000	4.210123000
1	-0.826139000	-3.244187000	4.402750000
1	0.471997000	-3.564433000	3.211574000
1	0.787554000	-3.756636000	4.963947000
6	0.314456000	-4.438820000	-3.062909000
1	-0.128229000	-4.833337000	-2.137257000
1	-0.489982000	-4.268210000	-3.792274000
1	0.988805000	-5.200376000	-3.480236000
6	2.251888000	-3.473396000	-1.831231000
1	2.875624000	-2.585255000	-1.646729000
1	1.900594000	-3.900960000	-0.880759000
1	2.898322000	-4.221010000	-2.311224000
6	1.677214000	-2.561176000	-4.064273000
1	0.895570000	-2.358169000	-4.809869000
1	2.205455000	-1.620800000	-3.847011000
1	2.392086000	-3.267573000	-4.509805000
6	-0.513642000	1.819752000	-0.218430000
6	-0.101697000	2.197659000	-1.503496000
6	-0.286200000	2.745831000	0.805975000

6	0.519950000	3.418814000	-1.747011000
6	0.330091000	3.974201000	0.569700000
6	0.756469000	4.331150000	-0.711027000
1	-0.250485000	1.512910000	-2.343896000
1	-0.585454000	2.507321000	1.831175000
1	0.840309000	3.668988000	-2.763403000
1	0.503364000	4.660764000	1.403943000
6	-2.918783000	0.624351000	-0.078184000
6	-3.800081000	-0.438781000	0.192216000
6	-3.502675000	1.827053000	-0.489572000
6	-5.177616000	-0.309377000	0.056751000
6	-4.886431000	1.965125000	-0.630165000
6	-5.750517000	0.901358000	-0.361225000
1	-3.399945000	-1.402165000	0.521635000
1	-2.858721000	2.682714000	-0.710081000
1	-5.825892000	-1.163163000	0.277746000
1	-5.302676000	2.922888000	-0.957180000
6	1.493392000	5.614205000	-0.959481000
1	1.307618000	5.999989000	-1.973017000
1	1.209688000	6.391944000	-0.235062000
1	2.581392000	5.457568000	-0.860986000
6	-7.238422000	1.038493000	-0.505896000
1	-7.747843000	0.895494000	0.460965000
1	-7.515654000	2.030511000	-0.889576000
1	-7.644036000	0.280961000	-1.195368000
8	2.031911000	0.298200000	-0.166830000

6.6.2 **TS7**

Energy(PBE0-D3(BJ)/def2-TZVPP) = -2929.21160445051

6	-6.397052000	-4.846638000	3.092556000
7	-5.833029000	-4.391841000	4.251730000
6	-4.522610000	-4.678168000	4.219458000
6	-5.411479000	-5.406543000	2.338367000
1	-5.461473000	-5.866147000	1.358324000
1	-7.456553000	-4.728100000	2.888370000
7	-4.243022000	-5.292463000	3.047429000
6	-2.909633000	-5.568990000	2.428406000
51	-3.199557000	-4.313123000	5.901551000
7	-6.169715000	-3.592574000	6.670003000
6	-4.932456000	-3.796580000	7.136547000
6	-6.256859000	-3.453410000	8.869655000
7	-4.965944000	-3.717527000	8.487914000
1	-6.558655000	-3.341303000	9.903894000
1	-8.063270000	-3.188938000	7.599146000
6	-7.002410000	-3.377155000	7.730931000
6	-3.809521000	-3.870801000	9.422963000
5	-6.595016000	-3.342511000	5.169787000
6	-2.712453000	-2.887076000	9.018175000
1	-2.217790000	-3.159543000	8.074188000
1	-3.107028000	-1.866137000	8.915375000
1	-1.924925000	-2.885321000	9.784546000
6	-4.266622000	-3.531315000	10.838427000
1	-4.634092000	-2.497419000	10.914759000
1	-5.043490000	-4.222087000	11.196036000
1	-3.402811000	-3.632808000	11.508944000
6	-3.324789000	-5.317349000	9.388591000
1	-4.108327000	-6.002352000	9.738133000
1	-3.024563000	-5.655308000	8.387285000
1	-2.454078000	-5.423413000	10.051346000
6	-3.029750000	-6.769511000	1.490777000
1	-3.385142000	-7.656721000	2.030957000
1	-3.692199000	-6.571861000	0.637066000
1	-2.034330000	-6.985949000	1.079753000
6	-1.857955000	-5.876068000	3.486089000
1	-1.507744000	-4.964731000	3.995808000
1	-2.205538000	-6.611315000	4.223903000
1	-0.971827000	-6.289520000	2.985374000
6	-2.521924000	-4.307356000	1.653579000
1	-3.255832000	-4.088041000	0.863911000
1	-2.466410000	-3.442290000	2.331896000
1	-1.538054000	-4.448038000	1.182859000
6	-6.132801000	-1.848729000	4.753307000
6	-5.542596000	-1.543258000	3.521591000
6	-6.386799000	-0.767477000	5.609741000
6	-5.205493000	-0.237403000	3.168862000
6	-6.057081000	0.541373000	5.264111000
6	-5.448460000	0.831916000	4.036805000
1	-5.325384000	-2.343363000	2.807362000

1	-6.858506000	-0.944269000	6.581662000
1	-4.735738000	-0.044462000	2.199629000
1	-6.270024000	1.355894000	5.963405000
6	-8.186775000	-3.566394000	5.012213000
6	-8.808306000	-4.738146000	5.473941000
6	-9.009244000	-2.629722000	4.372438000
6	-10.172259000	-4.960609000	5.310511000
6	-10.377147000	-2.847988000	4.202207000
6	-10.986549000	-4.016046000	4.671481000
1	-8.210923000	-5.503860000	5.972719000
1	-8.569339000	-1.704129000	3.991191000
1	-10.612127000	-5.890304000	5.683607000
1	-10.984850000	-2.094173000	3.692328000
6	-5.031876000	2.230861000	3.687996000
1	-5.021654000	2.390550000	2.599905000
1	-5.700295000	2.977413000	4.141799000
1	-4.012710000	2.437222000	4.057576000
6	-12.462783000	-4.242304000	4.520950000
1	-13.002689000	-3.939695000	5.434187000
1	-12.877732000	-3.657750000	3.687129000
1	-12.691661000	-5.304425000	4.347929000
8	-2.929565000	-2.435626000	5.122838000
6	-2.586180000	-0.332331000	6.156276000
6	-0.734678000	-1.874267000	5.930894000
6	-0.392333000	0.278997000	6.984897000
1	-2.137831000	1.549538000	7.095764000
1	1.156605000	-1.202047000	6.710164000
6	-2.090335000	-1.572999000	5.730151000
6	-1.738562000	0.584318000	6.772530000
6	0.102859000	-0.953417000	6.559113000
1	-3.641554000	-0.111040000	5.984245000
1	-0.340029000	-2.833163000	5.582516000
1	0.267608000	0.999297000	7.473518000
8	-5.004228000	-6.865485000	6.538878000
6	-6.306765000	-7.242925000	6.738046000
6	-6.898521000	-6.949757000	7.965350000
6	-7.040180000	-7.872655000	5.730083000
6	-8.230700000	-7.291097000	8.189030000
6	-8.366006000	-8.228235000	5.970110000
6	-8.964809000	-7.941108000	7.196876000
1	-6.300358000	-6.459824000	8.734279000
1	-6.573301000	-8.063544000	4.763927000
1	-8.694897000	-7.055603000	9.149675000
1	-8.939764000	-8.722415000	5.182608000
1	-10.005937000	-8.218046000	7.377482000
6	-4.061966000	-7.936680000	6.424158000
6	-3.991783000	-8.830940000	7.555093000
6	-3.808561000	-10.181861000	7.394902000
7	-3.762561000	-10.818553000	6.240258000
6	-3.935434000	-10.059140000	5.180012000
6	-4.126348000	-8.701303000	5.204895000
9	-3.689524000	-10.931179000	8.491718000
9	-4.064870000	-8.286199000	8.786538000

9	-2.845557000	-7.014177000	6.395372000
9	-4.356736000	-8.014010000	4.060096000
9	-3.949169000	-10.671287000	3.993569000

6.6.3 **INT8**

Energy(PBE0-D3(BJ)/def2-TZVPP) = -1978.355298907551

6	-1.490155000	-0.693919000	-2.257023000
7	-0.807930000	-0.551451000	-1.074138000
6	0.229144000	-1.399488000	-1.080939000
6	-0.862429000	-1.648279000	-2.989676000
1	-1.101368000	-2.046396000	-3.968766000
1	-2.374722000	-0.107980000	-2.484071000
7	0.211766000	-2.084596000	-2.246584000
6	1.041678000	-3.247076000	-2.674760000
51	1.607088000	-1.571719000	0.600074000
7	-0.969174000	-0.010390000	1.445950000
6	0.021629000	-0.782648000	1.907991000
6	-1.247144000	-0.203137000	3.627166000
7	-0.136142000	-0.919359000	3.250216000
1	-1.597946000	-0.145815000	4.650104000
1	-2.635701000	0.988081000	2.369947000
6	-1.761004000	0.359162000	2.501018000
6	0.657331000	-1.780003000	4.181884000
5	-1.252997000	0.542246000	-0.015337000
6	2.139835000	-1.429924000	4.078025000
1	2.561349000	-1.646498000	3.086502000
1	2.312741000	-0.368191000	4.304528000
1	2.702202000	-2.035460000	4.802458000
6	0.208970000	-1.512895000	5.616832000
1	0.345457000	-0.459122000	5.901905000
1	-0.838261000	-1.801845000	5.784548000
1	0.828981000	-2.125575000	6.285327000
6	0.391178000	-3.243249000	3.831754000
1	-0.674027000	-3.477844000	3.980664000
1	0.643063000	-3.473853000	2.786502000
1	0.981867000	-3.890587000	4.496529000
6	0.278183000	-4.516150000	-2.293660000
1	0.130981000	-4.534680000	-1.203866000
1	-0.699654000	-4.550955000	-2.796923000
1	0.854579000	-5.403287000	-2.595098000
6	2.402245000	-3.235796000	-1.989420000
1	2.893909000	-2.253704000	-2.065403000
1	2.314352000	-3.550683000	-0.940975000
1	3.049328000	-3.973455000	-2.483858000
6	1.257261000	-3.158613000	-4.185899000
1	0.325511000	-3.278572000	-4.755239000
1	1.722573000	-2.200963000	-4.463475000
1	1.929641000	-3.971511000	-4.492758000
6	-0.477964000	1.935124000	-0.253222000
6	-0.077906000	2.344794000	-1.532284000
6	-0.244977000	2.838555000	0.790461000
6	0.539357000	3.572978000	-1.752559000
6	0.366588000	4.073417000	0.577721000
6	0.782944000	4.460893000	-0.697683000
1	-0.231373000	1.679183000	-2.387151000

1	-0.532622000	2.575302000	1.812729000
1	0.851102000	3.847210000	-2.765460000
1	0.545789000	4.740291000	1.426613000
6	-2.866785000	0.689674000	-0.144949000
6	-3.705087000	-0.421524000	0.060280000
6	-3.493377000	1.898356000	-0.462081000
6	-5.088037000	-0.329025000	-0.045039000
6	-4.883585000	1.999235000	-0.570061000
6	-5.706750000	0.890051000	-0.363536000
1	-3.258928000	-1.390479000	0.310115000
1	-2.879798000	2.787791000	-0.630915000
1	-5.706173000	-1.216922000	0.121605000
1	-5.337580000	2.962856000	-0.820893000
6	1.519253000	5.749259000	-0.919280000
1	1.362347000	6.137755000	-1.936540000
1	1.209410000	6.522887000	-0.201001000
1	2.604673000	5.598677000	-0.787427000
6	-7.201034000	0.987515000	-0.472452000
1	-7.687077000	0.750561000	0.487969000
1	-7.517635000	1.996952000	-0.770775000
1	-7.595162000	0.274234000	-1.213937000
8	2.042923000	0.389069000	-0.154845000
6	2.711416000	1.566360000	1.824810000
6	3.572624000	2.174302000	-0.344851000
6	4.264103000	3.415454000	1.620701000
1	3.402558000	2.746636000	3.485217000
1	4.937017000	3.833186000	-0.391347000
6	2.749583000	1.333757000	0.435175000
6	3.459407000	2.588775000	2.404103000
6	4.310989000	3.195826000	0.240128000
1	2.055652000	0.952886000	2.446004000
1	3.599748000	2.003197000	-1.423391000
1	4.847174000	4.219066000	2.076224000
9	0.660721000	-3.442246000	0.740893000

6.6.4 **TS8**

Energy(PBE0-D3(BJ)/def2-TZVPP) = -2694.0975727423

6	-2.041498000	-1.307254000	-2.215486000
7	-1.508719000	-1.063887000	-0.975535000
6	-0.383614000	-1.778961000	-0.855222000
6	-1.221716000	-2.177560000	-2.859700000
1	-1.299122000	-2.609133000	-3.851592000
1	-2.959216000	-0.831229000	-2.545884000
7	-0.179897000	-2.457515000	-2.005707000
6	0.940371000	-3.354119000	-2.417116000
51	0.718069000	-1.904713000	1.008212000
7	-1.785047000	-0.072155000	1.421940000
6	-0.710767000	-0.593900000	2.018580000
6	-2.019557000	0.232112000	3.596138000
7	-0.836306000	-0.425017000	3.356082000
1	-2.360511000	0.499179000	4.588915000
1	-3.551317000	0.923053000	2.145966000
6	-2.606416000	0.446885000	2.387781000
6	0.087883000	-0.923800000	4.417920000
5	-2.090741000	0.134989000	-0.117393000
6	1.511082000	-0.467554000	4.111693000
1	1.927834000	-0.949925000	3.216782000
1	1.563372000	0.619921000	3.961597000
1	2.166801000	-0.742906000	4.949561000
6	-0.332091000	-0.332526000	5.760862000
1	-0.316136000	0.767060000	5.742664000
1	-1.328751000	-0.676304000	6.072642000
1	0.384973000	-0.667959000	6.521977000
6	-0.008773000	-2.447967000	4.490411000
1	-1.044239000	-2.765446000	4.676097000
1	0.336733000	-2.937965000	3.568723000
1	0.622534000	-2.814469000	5.312395000
6	0.400079000	-4.779800000	-2.469035000
1	0.033219000	-5.070710000	-1.476158000
1	-0.422228000	-4.873193000	-3.193696000
1	1.201323000	-5.472485000	-2.763366000
6	2.100617000	-3.270654000	-1.436871000
1	2.429092000	-2.231415000	-1.279778000
1	1.859837000	-3.753143000	-0.479387000
1	2.947743000	-3.832188000	-1.853585000
6	1.431700000	-2.885263000	-3.787816000
1	0.663861000	-2.982589000	-4.567336000
1	1.759969000	-1.836082000	-3.743831000
1	2.288329000	-3.504223000	-4.089189000
6	-1.371851000	1.509209000	-0.582563000
6	-0.423230000	1.577633000	-1.610208000
6	-1.652253000	2.712148000	0.086255000
6	0.228301000	2.766786000	-1.935446000
6	-1.006787000	3.904507000	-0.232723000
6	-0.040452000	3.952316000	-1.245163000
1	-0.155758000	0.676037000	-2.167378000

1	-2.390791000	2.723892000	0.893336000
1	0.975402000	2.769977000	-2.734821000
1	-1.250539000	4.816755000	0.320397000
6	-3.698014000	0.089711000	-0.292632000
6	-4.400514000	-1.053012000	0.132311000
6	-4.448661000	1.110942000	-0.881314000
6	-5.777764000	-1.163644000	-0.017155000
6	-5.834405000	1.005679000	-1.034720000
6	-6.524395000	-0.130259000	-0.604715000
1	-3.846674000	-1.879435000	0.590818000
1	-3.941892000	2.013346000	-1.235482000
1	-6.288914000	-2.069006000	0.324970000
1	-6.390449000	1.824313000	-1.501810000
6	0.709334000	5.217251000	-1.545623000
1	1.041598000	5.251281000	-2.593464000
1	0.096354000	6.108713000	-1.346647000
1	1.611304000	5.292902000	-0.914623000
6	-8.013242000	-0.249816000	-0.755867000
1	-8.511776000	-0.274303000	0.227031000
1	-8.429770000	0.594766000	-1.322759000
1	-8.288859000	-1.180546000	-1.276396000
8	1.763087000	-0.282240000	0.181977000
6	1.765211000	1.820535000	1.297143000
6	3.702427000	0.377396000	1.411516000
6	3.716180000	2.511194000	2.558957000
1	1.915890000	3.690974000	2.346137000
1	5.365288000	1.114924000	2.564194000
6	2.396924000	0.613919000	0.948439000
6	2.423334000	2.756423000	2.090487000
6	4.350338000	1.317343000	2.210923000
1	0.756135000	2.007579000	0.925841000
1	4.195490000	-0.558022000	1.133589000
1	4.228104000	3.246905000	3.183653000
14	-1.538734000	-4.892544000	1.706479000
9	-1.301325000	-3.096036000	1.415833000
6	-3.052979000	-4.296455000	2.745878000
1	-3.652545000	-3.535612000	2.221064000
1	-3.707121000	-5.152379000	2.982130000
1	-2.740971000	-3.855291000	3.708081000
6	-1.072755000	-6.324800000	2.888376000
1	-0.512306000	-5.921234000	3.750556000
1	-1.983546000	-6.797276000	3.287557000
1	-0.441702000	-7.103267000	2.435807000
6	-2.311177000	-5.507526000	0.099465000
1	-3.304666000	-5.939935000	0.297471000
1	-2.449311000	-4.667184000	-0.600207000
1	-1.700576000	-6.271116000	-0.404501000
8	0.245725000	-4.744008000	1.166371000

6.6.5 **INT9**

Energy(PBE0-D3(BJ)/def2-TZVPP) = -2694.09850577736

6	-2.187832000	-1.332699000	-2.131330000
7	-1.612657000	-1.092724000	-0.910543000
6	-0.474958000	-1.794312000	-0.837995000
6	-1.381604000	-2.187306000	-2.813545000
1	-1.484637000	-2.599407000	-3.811092000
1	-3.125481000	-0.868936000	-2.420807000
7	-0.312032000	-2.472469000	-1.997355000
6	0.887458000	-3.194804000	-2.517298000
51	0.743223000	-1.877604000	0.959883000
7	-1.792901000	-0.103196000	1.488383000
6	-0.692831000	-0.627121000	2.035448000
6	-1.937580000	0.179502000	3.672721000
7	-0.765046000	-0.472463000	3.378573000
1	-2.238415000	0.435005000	4.681430000
1	-3.528124000	0.882977000	2.291257000
6	-2.574274000	0.405127000	2.491038000
6	0.193181000	-0.997089000	4.398000000
5	-2.153141000	0.115195000	-0.035735000
6	1.608797000	-0.545898000	4.049217000
1	1.994736000	-1.022706000	3.137247000
1	1.663871000	0.543230000	3.911645000
1	2.289932000	-0.836518000	4.861177000
6	-0.172525000	-0.424340000	5.764492000
1	-0.147163000	0.675348000	5.765232000
1	-1.160053000	-0.765228000	6.106960000
1	0.568284000	-0.779579000	6.493170000
6	0.077488000	-2.520894000	4.443024000
1	-0.957010000	-2.822395000	4.661547000
1	0.374769000	-3.005994000	3.501707000
1	0.730028000	-2.913409000	5.236012000
6	0.423402000	-4.442719000	-3.265762000
1	-0.137472000	-5.111386000	-2.598168000
1	-0.200668000	-4.205077000	-4.138226000
1	1.307021000	-4.982427000	-3.633491000
6	1.814438000	-3.613536000	-1.387909000
1	2.349939000	-2.749710000	-0.963294000
1	1.287038000	-4.171735000	-0.600274000
1	2.588779000	-4.275608000	-1.798705000
6	1.616088000	-2.222925000	-3.447073000
1	0.985854000	-1.932141000	-4.300416000
1	1.901239000	-1.315584000	-2.893236000
1	2.528391000	-2.696353000	-3.837963000
6	-1.428995000	1.478151000	-0.526146000
6	-0.530135000	1.539264000	-1.597859000
6	-1.662567000	2.681344000	0.159544000
6	0.120326000	2.721364000	-1.949304000
6	-1.018499000	3.867213000	-0.185930000
6	-0.100639000	3.907294000	-1.242719000
1	-0.304074000	0.637899000	-2.173543000

1	-2.362596000	2.698945000	1.000348000
1	0.827812000	2.718576000	-2.783947000
1	-1.224745000	4.780234000	0.380939000
6	-3.764836000	0.105114000	-0.160903000
6	-4.487395000	-1.005387000	0.312433000
6	-4.502651000	1.131623000	-0.757453000
6	-5.870499000	-1.081739000	0.199243000
6	-5.893997000	1.060909000	-0.874114000
6	-6.603466000	-0.044435000	-0.398210000
1	-3.949264000	-1.835814000	0.780435000
1	-3.981013000	2.011105000	-1.145854000
1	-6.395740000	-1.963898000	0.578666000
1	-6.439366000	1.883043000	-1.347566000
6	0.650117000	5.163705000	-1.574624000
1	0.908173000	5.210534000	-2.642737000
1	0.070020000	6.062205000	-1.317843000
1	1.595939000	5.211283000	-1.008428000
6	-8.097531000	-0.130370000	-0.516622000
1	-8.572697000	-0.195384000	0.475581000
1	-8.511979000	0.746884000	-1.033138000
1	-8.401990000	-1.029882000	-1.075238000
8	1.720111000	-0.261429000	0.078550000
6	1.798759000	1.818584000	1.232470000
6	3.720178000	0.348469000	1.238010000
6	3.810124000	2.458966000	2.423201000
1	2.018882000	3.665982000	2.310308000
1	5.439364000	1.042502000	2.334185000
6	2.401436000	0.613487000	0.835537000
6	2.502104000	2.730386000	2.015038000
6	4.413684000	1.264520000	2.027133000
1	0.776987000	2.021686000	0.907054000
1	4.188542000	-0.587268000	0.921112000
1	4.357400000	3.175752000	3.039840000
14	-1.532557000	-4.928577000	1.515409000
9	-1.329484000	-3.190027000	1.458416000
6	-3.448209000	-4.628110000	1.531890000
1	-3.791610000	-4.122563000	0.613102000
1	-3.993662000	-5.585346000	1.602728000
1	-3.763667000	-4.005392000	2.387063000
6	-1.445484000	-5.804536000	3.193586000
1	-1.761789000	-5.073197000	3.956599000
1	-2.182658000	-6.623323000	3.225408000
1	-0.462608000	-6.199370000	3.483873000
6	-1.474085000	-5.810820000	-0.161099000
1	-2.250228000	-6.591238000	-0.213369000
1	-1.741005000	-5.053513000	-0.918708000
1	-0.505623000	-6.249250000	-0.438435000
8	0.403787000	-4.712797000	1.503638000

6.6.6 **TS10**

Energy(PBE0-D3(BJ)/def2-TZVPP) = -2722.372469265682

6	-6.233373000	-2.170037000	2.512198000
7	-5.935842000	-2.524083000	3.800646000
6	-5.375351000	-3.742847000	3.782847000
6	-5.859798000	-3.192760000	1.700537000
1	-5.933152000	-3.291089000	0.622801000
1	-6.692853000	-1.218411000	2.267154000
7	-5.339277000	-4.176886000	2.501672000
6	-4.934659000	-5.493547000	1.922682000
51	-4.454979000	-4.676325000	5.523022000
7	-5.754720000	-1.908538000	6.318621000
6	-5.082555000	-2.977123000	6.759241000
6	-5.262085000	-1.552527000	8.439844000
7	-4.772108000	-2.777930000	8.065168000
1	-5.143466000	-1.144980000	9.436270000
1	-6.378157000	-0.063238000	7.226999000
6	-5.872222000	-1.015625000	7.348428000
6	-4.166183000	-3.775310000	8.999568000
5	-6.603977000	-1.727822000	4.998817000
6	-2.757842000	-4.142319000	8.540113000
1	-2.728272000	-4.606937000	7.544869000
1	-2.116810000	-3.251366000	8.513720000
1	-2.327516000	-4.865445000	9.246967000
6	-4.063652000	-3.159996000	10.392151000
1	-3.414174000	-2.272277000	10.399620000
1	-5.049312000	-2.894425000	10.800338000
1	-3.617093000	-3.906952000	11.062235000
6	-5.096659000	-4.987959000	9.061461000
1	-6.077890000	-4.692597000	9.462652000
1	-5.277624000	-5.451306000	8.081154000
1	-4.664418000	-5.749534000	9.725909000
6	-6.134581000	-6.029554000	1.136521000
1	-7.012404000	-6.133928000	1.791597000
1	-6.403900000	-5.383728000	0.289513000
1	-5.882801000	-7.019658000	0.731134000
6	-4.592761000	-6.499199000	3.012349000
1	-3.646640000	-6.246886000	3.509951000
1	-5.413653000	-6.610626000	3.739012000
1	-4.446759000	-7.480544000	2.540262000
6	-3.727401000	-5.268983000	1.014698000
1	-3.963893000	-4.581420000	0.189517000
1	-2.895565000	-4.856585000	1.600069000
1	-3.417708000	-6.228756000	0.576551000
6	-6.636023000	-0.147864000	4.647818000
6	-5.564021000	0.498698000	4.008767000
6	-7.697401000	0.668776000	5.062944000
6	-5.547580000	1.876822000	3.808558000
6	-7.686550000	2.050404000	4.867794000
6	-6.610621000	2.682675000	4.236600000
1	-4.715716000	-0.085671000	3.642223000

1	-8.558160000	0.213220000	5.561125000
1	-4.690027000	2.337491000	3.308701000
1	-8.533551000	2.651020000	5.213097000
6	-8.066319000	-2.389250000	5.240003000
6	-8.387584000	-3.185255000	6.344727000
6	-9.081767000	-2.207347000	4.285825000
6	-9.646183000	-3.771237000	6.491440000
6	-10.337637000	-2.789639000	4.423066000
6	-10.645537000	-3.588843000	5.532799000
1	-7.642462000	-3.372689000	7.122681000
1	-8.891863000	-1.580661000	3.409166000
1	-9.853713000	-4.387049000	7.371756000
1	-11.099687000	-2.617874000	3.656441000
6	-6.603630000	4.164312000	3.996810000
1	-6.895307000	4.396544000	2.958728000
1	-7.309249000	4.683763000	4.660944000
1	-5.601809000	4.592043000	4.152624000
6	-11.993927000	-4.234404000	5.669837000
1	-12.150652000	-4.627871000	6.684276000
1	-12.804860000	-3.523813000	5.447258000
1	-12.103059000	-5.076703000	4.966339000
8	-2.400323000	-2.802204000	5.323136000
6	-2.786185000	-0.555981000	6.030309000
6	-0.848661000	-1.758173000	6.843685000
6	-1.299137000	0.490303000	7.621485000
1	-3.066238000	1.432325000	6.799856000
1	0.395989000	-0.698796000	8.243054000
6	-1.998633000	-1.701511000	6.055153000
6	-2.438972000	0.537808000	6.820739000
6	-0.503788000	-0.656857000	7.624543000
1	-3.666446000	-0.527095000	5.392362000
1	-0.241247000	-2.663600000	6.844606000
1	-1.024352000	1.348924000	8.238474000
6	-1.660615000	-3.041615000	4.128987000
6	-1.814875000	-2.071480000	3.091065000
6	-0.767840000	-1.752297000	2.258974000
7	0.456220000	-2.219938000	2.377384000
6	0.658767000	-3.054799000	3.374456000
6	-0.301162000	-3.458233000	4.272837000
9	-0.988460000	-0.887062000	1.270939000
9	-3.041040000	-1.545346000	2.904969000
9	-2.544120000	-4.342422000	3.760924000
9	-0.008888000	-4.308704000	5.274094000
9	1.896804000	-3.520836000	3.525170000
9	-6.163876000	-5.626220000	5.932343000
6	-6.233373000	-2.170037000	2.512198000
7	-5.935842000	-2.524083000	3.800646000
6	-5.375351000	-3.742847000	3.782847000

6.7 Mechanism D (OA to Si-O) – xyz coordinations (in Å) and Energies (in Hartree)

6.7.1 **TS11**

Energy(PBE0-D3(BJ)/def2-TZVPP) = -2287.58230043031

6	-1.852641000	-1.119203000	-2.000255000
7	-1.112982000	-0.770558000	-0.899745000
6	0.018096000	-1.502909000	-0.885882000
6	-1.177496000	-2.086286000	-2.666591000
1	-1.436704000	-2.607424000	-3.579531000
1	-2.805859000	-0.651288000	-2.221934000
7	-0.023082000	-2.340762000	-1.960793000
6	0.991306000	-3.311899000	-2.476654000
51	1.493002000	-1.355249000	0.726754000
7	-1.165336000	0.207401000	1.482369000
6	-0.054968000	-0.371878000	1.958667000
6	-1.227954000	0.414919000	3.677877000
7	-0.082632000	-0.259439000	3.315134000
1	-1.485698000	0.639836000	4.705470000
1	-2.845968000	1.209216000	2.383983000
6	-1.899068000	0.699881000	2.532217000
6	0.881648000	-0.850613000	4.286247000
5	-1.575741000	0.458029000	-0.018210000
6	2.295055000	-0.367772000	3.971118000
1	2.645385000	-0.685388000	2.978786000
1	2.353519000	0.728849000	4.018149000
1	2.995526000	-0.787182000	4.706973000
6	0.528015000	-0.389110000	5.698077000
1	0.556948000	0.706702000	5.788463000
1	-0.458738000	-0.752711000	6.018763000
1	1.273535000	-0.802783000	6.390696000
6	0.772982000	-2.373547000	4.215853000
1	-0.252958000	-2.695175000	4.449512000
1	1.037424000	-2.749518000	3.216798000
1	1.457156000	-2.828680000	4.946522000
6	0.271391000	-4.402532000	-3.273891000
1	-0.511448000	-4.890208000	-2.673824000
1	-0.171911000	-4.028517000	-4.206820000
1	1.012199000	-5.163455000	-3.554134000
6	1.734190000	-3.991800000	-1.334761000
1	2.408105000	-3.306756000	-0.809191000
1	1.035676000	-4.444657000	-0.616042000
1	2.362727000	-4.788135000	-1.756941000
6	1.946484000	-2.538155000	-3.383466000
1	1.398043000	-2.086578000	-4.224076000
1	2.455818000	-1.753421000	-2.807398000
1	2.709477000	-3.218067000	-3.789835000
6	-0.884814000	1.811721000	-0.563498000
6	-0.226569000	1.891624000	-1.796141000
6	-0.977018000	3.003022000	0.173395000

6	0.323260000	3.085521000	-2.262462000
6	-0.430060000	4.198689000	-0.284048000
6	0.241484000	4.261538000	-1.511424000
1	-0.127652000	0.996938000	-2.418194000
1	-1.484867000	3.001418000	1.142670000
1	0.838484000	3.100811000	-3.227565000
1	-0.518250000	5.103048000	0.325849000
6	-3.199472000	0.505018000	-0.067370000
6	-3.953639000	-0.571485000	0.434508000
6	-3.919185000	1.570215000	-0.617190000
6	-5.343151000	-0.580265000	0.393952000
6	-5.316379000	1.568919000	-0.662469000
6	-6.054872000	0.496678000	-0.156813000
1	-3.433801000	-1.432184000	0.869173000
1	-3.376149000	2.427367000	-1.025066000
1	-5.893117000	-1.437571000	0.794947000
1	-5.843443000	2.421190000	-1.102238000
6	0.882261000	5.533226000	-1.986261000
1	0.848483000	5.618034000	-3.082661000
1	0.392288000	6.418581000	-1.555273000
1	1.944623000	5.570391000	-1.690554000
6	-7.555837000	0.489573000	-0.188875000
1	-7.975453000	0.523791000	0.829981000
1	-7.950691000	1.352705000	-0.743163000
1	-7.942090000	-0.426765000	-0.662607000
8	3.513130000	-1.244184000	-0.939961000
6	4.632917000	-1.911338000	-1.195184000
6	5.114494000	-2.884836000	-0.295993000
6	5.354790000	-1.703632000	-2.387277000
6	6.267676000	-3.611774000	-0.576760000
6	6.510680000	-2.432429000	-2.658981000
6	6.977910000	-3.391502000	-1.758976000
1	4.561294000	-3.053399000	0.631953000
1	4.986490000	-0.961310000	-3.098733000
1	6.618840000	-4.360685000	0.138963000
1	7.052926000	-2.250340000	-3.591376000
1	7.884119000	-3.961614000	-1.976201000
14	3.286618000	0.552891000	-0.059971000
6	4.919715000	0.358429000	0.884027000
1	4.809764000	-0.342243000	1.728242000
1	5.729111000	-0.028001000	0.247857000
1	5.224613000	1.331764000	1.298973000
6	3.431917000	1.348056000	-1.770963000
1	4.371061000	1.041449000	-2.254657000
1	2.601633000	1.019391000	-2.413984000
1	3.395439000	2.446449000	-1.708482000
6	2.468087000	1.982081000	0.933158000
1	1.494398000	2.254073000	0.501437000
1	2.308628000	1.728562000	1.992901000
1	3.122763000	2.872153000	0.890551000

6.7.2 **INT10**

Energy(PBE0-D3(BJ)/def2-TZVPP) = -2287.59058030253

6	-1.717268000	-1.145771000	-1.967457000
7	-0.885220000	-0.735929000	-0.958950000
6	0.214319000	-1.514302000	-0.957002000
6	-1.138555000	-2.207635000	-2.577047000
1	-1.490215000	-2.802214000	-3.410512000
1	-2.662816000	-0.654296000	-2.168940000
7	0.048773000	-2.456914000	-1.929986000
6	0.912980000	-3.598380000	-2.378777000
51	1.816626000	-1.324607000	0.543684000
7	-0.834217000	0.331535000	1.376346000
6	0.127233000	-0.475508000	1.849904000
6	-0.942659000	0.445618000	3.577289000
7	0.058712000	-0.427214000	3.207010000
1	-1.198642000	0.659854000	4.607902000
1	-2.335654000	1.588721000	2.284982000
6	-1.498424000	0.912849000	2.429397000
6	0.837236000	-1.274114000	4.152928000
5	-1.276249000	0.549687000	-0.118505000
6	2.320823000	-0.921177000	4.070879000
1	2.734197000	-1.110681000	3.070216000
1	2.488453000	0.134573000	4.326638000
1	2.886305000	-1.543262000	4.779371000
6	0.354923000	-1.022091000	5.579007000
1	0.516664000	0.020628000	5.889570000
1	-0.708217000	-1.274250000	5.703905000
1	0.933462000	-1.664344000	6.257088000
6	0.595345000	-2.741228000	3.796974000
1	-0.478972000	-2.974749000	3.839625000
1	0.965548000	-2.973891000	2.788465000
1	1.123148000	-3.388111000	4.512729000
6	0.007259000	-4.724847000	-2.889081000
1	-0.739503000	-5.013341000	-2.134185000
1	-0.508824000	-4.474078000	-3.825740000
1	0.638316000	-5.599367000	-3.097712000
6	1.729913000	-4.165772000	-1.227101000
1	2.509221000	-3.471701000	-0.902883000
1	1.089343000	-4.435057000	-0.374220000
1	2.233317000	-5.076751000	-1.578996000
6	1.809045000	-3.089948000	-3.506211000
1	1.200486000	-2.746646000	-4.356828000
1	2.427559000	-2.261372000	-3.136461000
1	2.464918000	-3.901244000	-3.854408000
6	-0.633003000	1.859935000	-0.809086000
6	-0.315041000	1.889632000	-2.173880000
6	-0.537245000	3.080462000	-0.124173000
6	0.084570000	3.061436000	-2.816126000
6	-0.122351000	4.252339000	-0.752660000
6	0.200419000	4.266007000	-2.115467000
1	-0.376963000	0.971906000	-2.765378000

1	-0.803591000	3.128670000	0.936054000
1	0.325162000	3.036699000	-3.883236000
1	-0.053049000	5.179736000	-0.175755000
6	-2.904515000	0.632399000	-0.097282000
6	-3.654308000	-0.394883000	0.504288000
6	-3.628741000	1.683390000	-0.667460000
6	-5.043660000	-0.373737000	0.532741000
6	-5.026402000	1.713595000	-0.640979000
6	-5.760275000	0.687964000	-0.041418000
1	-3.130266000	-1.240754000	0.962499000
1	-3.090518000	2.504036000	-1.149888000
1	-5.590254000	-1.194157000	1.008617000
1	-5.557233000	2.554016000	-1.098680000
6	0.684512000	5.518077000	-2.786856000
1	0.501707000	5.490669000	-3.870868000
1	0.194534000	6.413067000	-2.375140000
1	1.770543000	5.646704000	-2.641167000
6	-7.260856000	0.713038000	-0.000671000
1	-7.629876000	0.786191000	1.035463000
1	-7.664444000	1.567517000	-0.562047000
1	-7.688340000	-0.208160000	-0.427523000
8	3.178044000	-1.344459000	-1.334516000
6	4.406538000	-1.794655000	-1.398960000
6	4.975217000	-2.660027000	-0.430570000
6	5.234158000	-1.430902000	-2.491104000
6	6.283029000	-3.123341000	-0.552172000
6	6.537741000	-1.900933000	-2.601875000
6	7.080616000	-2.753481000	-1.635836000
1	4.372809000	-2.956888000	0.432358000
1	4.816106000	-0.762297000	-3.248494000
1	6.685701000	-3.787905000	0.218416000
1	7.144217000	-1.595591000	-3.460050000
1	8.105877000	-3.119314000	-1.725984000
14	2.939181000	1.036326000	0.549173000
6	4.684082000	0.595407000	1.123868000
1	4.690094000	0.070889000	2.091999000
1	5.208117000	-0.033171000	0.389371000
1	5.253064000	1.532963000	1.245880000
6	3.054286000	1.884669000	-1.115043000
1	3.298224000	1.136661000	-1.882371000
1	2.117796000	2.387399000	-1.386366000
1	3.862212000	2.634107000	-1.073142000
6	2.192801000	2.167034000	1.858708000
1	1.149329000	2.419290000	1.633433000
1	2.235001000	1.707582000	2.856458000
1	2.772472000	3.105102000	1.889615000

6.8 Dimerization mechanism in C_6H_6 – xyz coordinates (in Å) and Energies (in Hartree)

6.8.1 **Sb(III)-F species 15**

Energy(PBE0-D3(BJ)/def2-TZVPP) = -1771.51808842061

6	-1.864389000	-0.343790000	-2.407585000
7	-1.159092000	-0.341170000	-1.230231000
6	-0.243694000	-1.318546000	-1.281869000
6	-1.376438000	-1.344565000	-3.184009000
1	-1.677031000	-1.667278000	-4.173944000
1	-2.664171000	0.363899000	-2.599751000
7	-0.365192000	-1.950463000	-2.472656000
6	0.275675000	-3.211433000	-2.944784000
51	1.104498000	-1.731074000	0.391302000
7	-1.258679000	0.075315000	1.317651000
6	-0.381715000	-0.844469000	1.738255000
6	-1.559505000	-0.178994000	3.491113000
7	-0.552942000	-1.016987000	3.071032000
1	-1.895321000	-0.122200000	4.519266000
1	-2.777514000	1.242964000	2.299855000
6	-1.994471000	0.497555000	2.395144000
6	0.171282000	-1.978208000	3.954344000
5	-1.449973000	0.741619000	-0.109206000
6	1.676271000	-1.759924000	3.808877000
1	2.045948000	-2.040569000	2.812370000
1	1.941528000	-0.708600000	3.993742000
1	2.207588000	-2.390774000	4.535239000
6	-0.213381000	-1.714283000	5.407597000
1	0.039554000	-0.690706000	5.721802000
1	-1.282021000	-1.895958000	5.590763000
1	0.351202000	-2.411436000	6.041624000
6	-0.238938000	-3.398387000	3.567320000
1	-1.314424000	-3.542296000	3.752521000
1	-0.050289000	-3.607855000	2.503979000
1	0.316484000	-4.120936000	4.183282000
6	-0.669480000	-4.360111000	-2.588745000
1	-0.806811000	-4.385784000	-1.497754000
1	-1.646021000	-4.233692000	-3.079960000
1	-0.236661000	-5.315588000	-2.920593000
6	1.623743000	-3.431951000	-2.271366000
1	2.270586000	-2.543706000	-2.337085000
1	1.487700000	-3.740752000	-1.226558000
1	2.137116000	-4.258729000	-2.781980000
6	0.490357000	-3.112758000	-4.455135000
1	-0.453599000	-3.071248000	-5.015522000
1	1.095532000	-2.230679000	-4.712761000
1	1.026599000	-4.010258000	-4.792795000
6	-0.486514000	2.022337000	-0.284627000
6	-0.107737000	2.475728000	-1.555491000
6	-0.068111000	2.797370000	0.803223000

6	0.649479000	3.631661000	-1.731860000
6	0.689079000	3.955939000	0.636276000
6	1.067699000	4.395973000	-0.636269000
1	-0.405303000	1.910239000	-2.444032000
1	-0.329290000	2.491126000	1.820766000
1	0.925589000	3.947858000	-2.742939000
1	0.998214000	4.530681000	1.515049000
6	-3.025006000	1.130614000	-0.225697000
6	-4.017630000	0.143175000	-0.085574000
6	-3.468449000	2.436289000	-0.456049000
6	-5.372026000	0.443712000	-0.171461000
6	-4.828840000	2.746529000	-0.543575000
6	-5.805774000	1.758498000	-0.403065000
1	-3.719210000	-0.894973000	0.097066000
1	-2.731930000	3.236630000	-0.569769000
1	-6.113445000	-0.353495000	-0.057193000
1	-5.136285000	3.781038000	-0.724837000
6	1.916688000	5.620735000	-0.821872000
1	1.624656000	6.182528000	-1.722172000
1	1.845277000	6.296542000	0.043035000
1	2.980185000	5.351683000	-0.940326000
6	-7.269655000	2.080131000	-0.491321000
1	-7.788414000	1.839293000	0.450773000
1	-7.435183000	3.145554000	-0.705083000
1	-7.760483000	1.493809000	-1.284650000
9	-0.090948000	-3.473986000	0.451731000
9	1.703474000	0.192121000	0.122816000

6.8.2 **Sb(III) diaryl species 16**

Energy(PBE0-D3(BJ)/def2-TZVPP) = -2860.38111940882

6	1.821981000	0.323120000	2.693542000
7	1.199345000	-0.053387000	1.532466000
6	0.144496000	-0.819128000	1.844521000
6	1.139025000	-0.227213000	3.729238000
1	1.321462000	-0.168166000	4.796646000
1	2.708725000	0.948395000	2.691667000
7	0.093158000	-0.941027000	3.191471000
6	-0.821038000	-1.714991000	4.081715000
51	-1.162328000	-1.690930000	0.329260000
7	1.489834000	-0.678506000	-0.947921000
6	0.411485000	-1.443995000	-1.164909000
6	1.829573000	-1.774457000	-2.830157000
7	0.599955000	-2.122084000	-2.321097000
1	2.220032000	-2.184349000	-3.755210000
1	3.329453000	-0.357035000	-2.013891000
6	2.377265000	-0.875534000	-1.972917000
6	-0.271120000	-3.091752000	-3.046471000
5	1.722603000	0.461181000	0.128306000
6	-0.397004000	-2.607468000	-4.493622000
1	-0.817677000	-1.591543000	-4.520880000
1	0.566239000	-2.604360000	-5.021305000
1	-1.072003000	-3.280460000	-5.040576000
6	0.393544000	-4.466225000	-2.973281000
1	1.387588000	-4.458688000	-3.443024000
1	0.504902000	-4.786166000	-1.926964000
1	-0.226991000	-5.205767000	-3.499425000
6	-1.667168000	-3.156442000	-2.447409000
1	-1.682848000	-3.638113000	-1.460621000
1	-2.133628000	-2.161268000	-2.402807000
1	-2.290168000	-3.780345000	-3.103084000
6	-2.002400000	-2.301578000	3.326714000
1	-2.603725000	-1.521904000	2.839507000
1	-1.693199000	-3.066116000	2.600899000
1	-2.651401000	-2.807658000	4.054824000
6	-1.356449000	-0.756864000	5.148052000
1	-0.562007000	-0.363694000	5.796857000
1	-1.876366000	0.088698000	4.675063000
1	-2.071157000	-1.292993000	5.788062000
6	-0.004308000	-2.849383000	4.702874000
1	0.379942000	-3.518036000	3.918165000
1	0.847195000	-2.466700000	5.283435000
1	-0.642191000	-3.436441000	5.379030000
6	0.926452000	1.787662000	-0.328176000
6	0.340629000	2.664323000	0.593588000
6	0.858941000	2.159834000	-1.677764000
6	-0.312377000	3.826466000	0.190146000
6	0.208019000	3.319820000	-2.090471000
6	-0.405101000	4.170601000	-1.163572000
1	0.363755000	2.423517000	1.661059000

1	1.308214000	1.518130000	-2.441390000
1	-0.780921000	4.471654000	0.939600000
1	0.156097000	3.562902000	-3.155886000
6	3.320805000	0.724380000	0.256804000
6	4.212222000	-0.306631000	0.610150000
6	3.883636000	1.986045000	0.033922000
6	5.580220000	-0.087101000	0.728402000
6	5.257386000	2.213399000	0.152609000
6	6.132557000	1.182433000	0.500807000
1	3.826605000	-1.311482000	0.796966000
1	3.232021000	2.819776000	-0.240911000
1	6.238380000	-0.917188000	1.003758000
1	5.656439000	3.215819000	-0.029870000
6	-1.187998000	5.371009000	-1.604181000
1	-1.107904000	6.195518000	-0.880361000
1	-0.854288000	5.735222000	-2.586655000
1	-2.258196000	5.116489000	-1.690447000
6	7.610276000	1.412785000	0.629564000
1	8.175501000	0.775711000	-0.069680000
1	7.874146000	2.459581000	0.422782000
1	7.965483000	1.166887000	1.643296000
6	-1.942946000	0.480266000	-0.119746000
6	-2.583521000	1.178617000	0.889208000
6	-2.138530000	0.992359000	-1.389508000
6	-3.337918000	2.312932000	0.613133000
6	-2.893713000	2.146530000	-1.580906000
7	-3.486976000	2.780622000	-0.601059000
9	-2.477270000	0.777927000	2.171582000
9	-1.599183000	0.400210000	-2.466607000
9	-3.931245000	2.970871000	1.601181000
9	-3.027045000	2.648305000	-2.801986000
6	0.106055000	-3.772197000	0.799195000
6	-0.615150000	-4.953138000	0.798007000
6	1.462102000	-3.938399000	1.010834000
6	0.006510000	-6.186532000	0.983603000
6	2.016822000	-5.205101000	1.188503000
7	1.297571000	-6.299726000	1.171556000
9	2.291597000	-2.884534000	1.056077000
9	3.322426000	-5.334444000	1.384671000
9	-0.711985000	-7.301173000	0.974400000
9	-1.948392000	-4.941231000	0.610706000

6.8.3 TMS$_2$Pyrdz S6

Energy(PBE0-D3(BJ)/def2-TZVPP) = -1082.28073378437

6	0.721497000	2.066682000	-2.439679000
6	0.535090000	3.179729000	-1.716043000
7	0.624232000	3.219030000	-0.307964000
6	0.945994000	1.961632000	0.256677000
6	1.132402000	0.848559000	-0.466917000
7	1.041088000	0.808764000	-1.874882000
1	0.628032000	2.107272000	-3.526018000
1	0.296702000	4.117113000	-2.219269000
1	1.041817000	1.921543000	1.342817000
1	1.373062000	-0.088338000	0.036148000
14	0.763677000	4.709799000	0.628093000
14	0.898610000	-0.681933000	-2.810578000
6	-0.046520000	6.081151000	-0.360121000
1	0.507550000	6.309664000	-1.283913000
1	-1.082732000	5.823933000	-0.629790000
1	-0.071082000	7.000456000	0.246685000
6	-0.122868000	4.455580000	2.262808000
1	-1.178020000	4.185008000	2.101526000
1	0.345907000	3.663235000	2.867220000
1	-0.090884000	5.384125000	2.855310000
6	2.569010000	5.122913000	0.956355000
1	2.669796000	6.034243000	1.567995000
1	3.064388000	4.297671000	1.492755000
1	3.109922000	5.282680000	0.010020000
6	1.705028000	-2.054915000	-1.821540000
1	1.150222000	-2.281355000	-0.897673000
1	1.727121000	-2.974602000	-2.427866000
1	2.741948000	-1.800474000	-1.551937000
6	-0.907527000	-1.090807000	-3.139693000
1	-1.010041000	-2.001894000	-3.751414000
1	-1.449287000	-1.249359000	-2.193635000
1	-1.400758000	-0.264437000	-3.676341000
6	1.786543000	-0.430460000	-4.445000000
1	2.842376000	-0.162795000	-4.283297000
1	1.752217000	-1.358999000	-5.037386000
1	1.320237000	0.363107000	-5.049732000

6.8.4 **Pyrz**

Energy(PBE0-D3(BJ)/def2-TZVPP) = -264.115873541511

6	0.831521000	2.012829000	-2.420428000
6	0.831521000	3.201697000	-1.687811000
7	0.831521000	3.204811000	-0.357478000
6	0.831520000	2.015144000	0.237857000
6	0.831521000	0.826276000	-0.494760000
7	0.831521000	0.823163000	-1.825093000
1	0.831521000	2.033595000	-3.515790000
1	0.831522000	4.170790000	-2.198766000
1	0.831520000	1.994378000	1.333219000
1	0.831522000	-0.142817000	0.016194000

6.8.5 **INT11**

Energy(PBE0-D3(BJ)/def2-TZVPP) = -2860.36390684287

6	1.638270000	0.339706000	2.493779000
7	1.009917000	-0.003898000	1.320295000
6	-0.015682000	-0.837428000	1.581135000
6	1.002775000	-0.306594000	3.501496000
1	1.203375000	-0.306649000	4.567996000
1	2.495407000	1.005062000	2.520038000
7	-0.010784000	-1.043906000	2.926338000
6	-0.842762000	-1.941190000	3.770012000
51	-1.541886000	-1.481135000	-0.167882000
7	1.241836000	-0.474282000	-1.202223000
6	0.349883000	-1.476156000	-1.286155000
6	1.862690000	-1.776457000	-2.873273000
7	0.750791000	-2.317829000	-2.274841000
1	2.376913000	-2.255128000	-3.699901000
1	2.990115000	0.054164000	-2.342792000
6	2.163381000	-0.634749000	-2.205208000
6	0.351789000	-3.721630000	-2.602960000
5	1.498196000	0.604764000	-0.046121000
6	0.057801000	-3.824357000	-4.099316000
1	-0.749783000	-3.145365000	-4.396531000
1	0.942528000	-3.588752000	-4.707533000
1	-0.242510000	-4.854734000	-4.335478000
6	1.538873000	-4.616130000	-2.227107000
1	2.438211000	-4.374793000	-2.810539000
1	1.777881000	-4.510051000	-1.158295000
1	1.280530000	-5.666668000	-2.422913000
6	-0.857608000	-4.182249000	-1.806330000
1	-0.667689000	-4.159479000	-0.723286000
1	-1.758309000	-3.601359000	-2.042646000
1	-1.063586000	-5.227137000	-2.076334000
6	-1.864217000	-2.705651000	2.945722000
1	-2.592373000	-2.037266000	2.470075000
1	-1.383868000	-3.322108000	2.173083000
1	-2.415801000	-3.377368000	3.618440000
6	-1.563921000	-1.087291000	4.815134000
1	-0.855318000	-0.569918000	5.477828000
1	-2.196375000	-0.333835000	4.326377000
1	-2.200880000	-1.729061000	5.440752000
6	0.092509000	-2.949760000	4.444029000
1	0.635152000	-3.536336000	3.687436000
1	0.829326000	-2.458663000	5.095106000
1	-0.495947000	-3.641356000	5.064157000
6	0.800828000	2.025246000	-0.337238000
6	0.256169000	2.807877000	0.690134000
6	0.832461000	2.601494000	-1.614312000
6	-0.247165000	4.085037000	0.454284000
6	0.326590000	3.875844000	-1.859876000

6	-0.230933000	4.641633000	-0.829665000
1	0.209996000	2.410046000	1.708087000
1	1.247440000	2.035294000	-2.453311000
1	-0.677161000	4.659008000	1.280670000
1	0.351871000	4.282266000	-2.875281000
6	3.119706000	0.747678000	0.069655000
6	3.908633000	-0.376884000	0.375225000
6	3.797169000	1.958068000	-0.100702000
6	5.290383000	-0.297647000	0.497082000
6	5.187743000	2.046467000	0.019634000
6	5.960687000	0.923178000	0.319563000
1	3.422507000	-1.347574000	0.523089000
1	3.227349000	2.861725000	-0.333239000
1	5.867623000	-1.196655000	0.735380000
1	5.680955000	3.012960000	-0.121943000
6	-0.844251000	5.983248000	-1.099334000
1	-0.758992000	6.650511000	-0.229142000
1	-0.377068000	6.476343000	-1.964351000
1	-1.918229000	5.872463000	-1.324959000
6	7.453602000	1.006591000	0.453740000
1	7.957169000	0.331619000	-0.256885000
1	7.817626000	2.026907000	0.267804000
1	7.780810000	0.709134000	1.463212000
6	-2.143668000	0.631716000	-0.143927000
6	-2.769271000	1.086973000	1.008651000
6	-2.167697000	1.503467000	-1.226278000
6	-3.365518000	2.343717000	1.038441000
6	-2.817199000	2.731683000	-1.105198000
7	-3.393824000	3.132982000	-0.002404000
9	-2.829036000	0.320555000	2.101720000
9	-1.595178000	1.207074000	-2.380375000
9	-3.943905000	2.763545000	2.152137000
9	-2.872846000	3.535578000	-2.153961000
6	-2.722907000	-1.418312000	-2.272265000
6	-4.066373000	-1.722697000	-2.116388000
6	-2.335755000	-1.209631000	-3.586168000
6	-4.919960000	-1.828654000	-3.212649000
6	-3.246991000	-1.354146000	-4.630285000
7	-4.507405000	-1.654389000	-4.442492000
9	-1.079426000	-0.880823000	-3.910934000
9	-2.834955000	-1.196408000	-5.882258000
9	-6.199844000	-2.121942000	-3.028974000
9	-4.579443000	-1.932227000	-0.892783000

6.8.6 **TS12**

Energy(PBE0-D3(BJ)/def2-TZVPP) = -2860.32336804505

6	1.852256000	0.269635000	2.676804000
7	1.370577000	-0.149385000	1.467528000
6	0.247744000	-0.855039000	1.667307000
6	1.016483000	-0.199984000	3.638825000
1	1.055137000	-0.075766000	4.715268000
1	2.751510000	0.871566000	2.759412000
7	0.027474000	-0.915113000	3.008361000
6	-1.069658000	-1.535540000	3.814318000
51	-0.855139000	-1.837344000	0.027369000
7	1.853986000	-0.677534000	-1.012009000
6	0.863293000	-1.536853000	-1.289985000
6	2.321539000	-1.618605000	-2.949387000
7	1.144576000	-2.141141000	-2.471649000
1	2.758794000	-1.926792000	-3.892792000
1	3.660494000	-0.106808000	-2.030806000
6	2.762431000	-0.716061000	-2.035899000
6	0.387029000	-3.136099000	-3.290710000
5	1.987277000	0.415042000	0.130670000
6	-0.102469000	-2.425781000	-4.554006000
1	-0.783121000	-1.604197000	-4.293898000
1	0.733608000	-2.020106000	-5.141563000
1	-0.644487000	-3.143764000	-5.186084000
6	1.339823000	-4.284513000	-3.629529000
1	2.187695000	-3.958131000	-4.247380000
1	1.731842000	-4.748502000	-2.712181000
1	0.791956000	-5.048995000	-4.197888000
6	-0.806045000	-3.702498000	-2.543819000
1	-0.514625000	-4.215396000	-1.616157000
1	-1.568252000	-2.935858000	-2.330842000
1	-1.292330000	-4.443663000	-3.193026000
6	-1.895831000	-2.513496000	2.997303000
1	-2.477605000	-2.023308000	2.207009000
1	-1.279003000	-3.318592000	2.573219000
1	-2.629270000	-2.979224000	3.669995000
6	-1.957846000	-0.403284000	4.331719000
1	-1.388717000	0.299153000	4.958055000
1	-2.404980000	0.148270000	3.493869000
1	-2.769116000	-0.824738000	4.942645000
6	-0.428061000	-2.304782000	4.972023000
1	0.279672000	-3.058782000	4.596518000
1	0.098736000	-1.648505000	5.677756000
1	-1.217584000	-2.822291000	5.534309000
6	1.186109000	1.750658000	-0.286840000
6	0.300117000	2.405505000	0.579265000
6	1.399996000	2.352177000	-1.535294000
6	-0.347792000	3.583797000	0.216456000
6	0.757836000	3.530752000	-1.903661000
6	-0.138518000	4.165709000	-1.037190000
1	0.092338000	1.990066000	1.569689000

1	2.081100000	1.886568000	-2.253759000
1	-1.050913000	4.050144000	0.912726000
1	0.936927000	3.958137000	-2.894401000
6	3.570847000	0.643150000	0.390691000
6	4.398190000	-0.445295000	0.722504000
6	4.180902000	1.899687000	0.329609000
6	5.756433000	-0.287886000	0.972362000
6	5.546027000	2.065907000	0.579670000
6	6.359583000	0.977620000	0.903586000
1	3.964641000	-1.449455000	0.786566000
1	3.574726000	2.774859000	0.080096000
1	6.366973000	-1.159998000	1.226590000
1	5.987569000	3.065285000	0.521496000
6	-0.911812000	5.377400000	-1.460783000
1	-1.013706000	6.102658000	-0.639468000
1	-0.441318000	5.882230000	-2.316716000
1	-1.929742000	5.079055000	-1.762549000
6	7.828150000	1.142893000	1.167075000
1	8.429466000	0.586591000	0.429815000
1	8.129278000	2.198819000	1.119970000
1	8.101531000	0.753937000	2.160846000
6	-2.070450000	-0.178542000	-0.787146000
6	-2.837535000	0.559443000	0.161398000
6	-1.602024000	0.686410000	-1.821441000
6	-3.345069000	1.803963000	-0.132614000
6	-2.198290000	1.906422000	-2.038683000
7	-3.071353000	2.474667000	-1.229198000
9	-3.189230000	-0.045371000	1.318895000
9	-0.719064000	0.220104000	-2.719936000
9	-4.141572000	2.393853000	0.756657000
9	-1.872849000	2.586450000	-3.134450000
6	-3.365688000	-1.471463000	-1.615990000
6	-4.255632000	-2.168390000	-0.814196000
6	-3.841242000	-1.190430000	-2.887538000
6	-5.521336000	-2.516649000	-1.273832000
6	-5.125933000	-1.571755000	-3.270182000
7	-5.945742000	-2.214532000	-2.475978000
9	-3.078136000	-0.569589000	-3.798561000
9	-5.555061000	-1.295790000	-4.494812000
9	-6.346136000	-3.197777000	-0.487523000
9	-3.911229000	-2.562263000	0.428603000

6.8.7 **BiPy-F8 10a**

Energy(PBE0-D3(BJ)/def2-TZVPP) = -1288.52075517266

6	-1.259342000	-0.000003000	-1.771564000
6	-1.259345000	1.193727000	-1.053705000
6	-1.259350000	1.131284000	0.338981000
7	-1.259350000	0.000001000	0.992906000
6	-1.259346000	-1.131283000	0.338984000
6	-1.259342000	-1.193731000	-1.053701000
9	-1.259353000	2.250594000	1.032420000
9	-1.259344000	2.355165000	-1.679768000
9	-1.259336000	-2.355170000	-1.679762000
9	-1.259347000	-2.250592000	1.032426000
6	-1.259342000	-0.000007000	-3.249301000
6	-0.065674000	-0.000005000	-3.967193000
6	-2.453012000	-0.000002000	-3.967191000
6	-0.128179000	-0.000008000	-5.359921000
6	-2.390507000	0.000000000	-5.359919000
7	-1.259343000	-0.000001000	-6.014084000
9	1.095969000	0.000003000	-3.341530000
9	-3.614652000	0.000009000	-3.341524000
9	-3.510119000	0.000014000	-6.052948000
9	0.991432000	0.000008000	-6.052950000

6.9 Dimerization mechanism in CH_3CN – xyz coordinates (in Å) and Energies (in Hartree)

6.9.1 **Stibinidene 1a**

Energy(PBE0-D3(BJ)/def2-TZVPP) = -1571.87539064459

6	1.076682	-2.423035	-1.023125
7	0.468673	-1.274980	-0.574911
6	-0.847728	-1.511642	-0.421646
6	0.122864	-3.381882	-1.142101
1	0.211555	-4.414854	-1.457226
1	2.144446	-2.473926	-1.210736
7	-1.075065	-2.808955	-0.766071
6	-2.387882	-3.511005	-0.799064
51	-2.249728	0.000184	0.235484
7	0.468774	1.275475	-0.574534
6	-0.847623	1.512142	-0.421162
6	0.123067	3.382707	-1.140627
7	-1.074892	2.809635	-0.764919
1	0.211812	4.415842	-1.455202
1	2.144635	2.474774	-1.209478
6	1.076858	2.423786	-1.022025
6	-2.387699	3.511707	-0.797542
5	1.246578	0.000132	-0.078722
6	-3.078486	3.349667	0.554836
1	-3.360416	2.307380	0.765413
1	-2.429852	3.706146	1.368513
1	-4.006441	3.938877	0.561441
6	-2.162350	5.001543	-1.042172
1	-1.530500	5.451283	-0.262107
1	-1.714458	5.197111	-2.026861
1	-3.139757	5.502339	-1.017280
6	-3.227496	2.934238	-1.936055
1	-2.721566	3.089686	-2.900636
1	-3.396438	1.855944	-1.796725
1	-4.204027	3.438639	-1.967198
6	-3.227748	-2.932775	-1.937134
1	-3.396736	-1.854588	-1.797032
1	-2.721855	-3.087513	-2.901849
1	-4.204256	-3.437200	-1.968594
6	-3.078617	-3.349987	0.553470
1	-2.429891	-3.706940	1.366863
1	-3.360694	-2.307896	0.764788
1	-4.006487	-3.939333	0.559719
6	-2.162504	-5.000653	-1.044783
1	-1.714734	-5.195508	-2.029668
1	-1.530523	-5.450904	-0.265120
1	-3.139883	-5.501516	-1.020114
6	1.286154	-0.000126	1.541818
6	1.346088	-1.192303	2.278264
6	1.346120	1.191759	2.278713

6	1.448779	-1.197566	3.669408
6	1.448818	1.196482	3.669869
6	1.497188	-0.000676	4.395369
1	1.305812	-2.152328	1.752784
1	1.305882	2.151990	1.753610
1	1.490903	-2.150868	4.206038
1	1.490969	2.149576	4.206866
6	2.722843	-0.000004	-0.749947
6	2.877249	-0.001304	-2.149055
6	3.900087	0.000937	0.006003
6	4.130155	-0.001509	-2.753836
6	5.163592	0.000774	-0.592795
6	5.303603	-0.000427	-1.983181
1	1.986859	-0.002188	-2.787148
1	3.832077	0.001881	1.097800
1	4.206276	-0.002518	-3.845875
1	6.060416	0.001584	0.034650
6	1.569314	-0.000999	5.895507
1	2.090718	-0.892736	6.273780
1	2.088483	0.891825	6.274268
1	0.559604	-0.002428	6.340628
6	6.654180	-0.000573	-2.639124
1	6.784436	-0.885350	-3.282992
1	6.784210	0.883654	-3.283800
1	7.462743	-0.000109	-1.894496

6.9.2 **PyrF5**

Energy(PBE0-D3(BJ)/def2-TZVPP) = -744.038368108901

6	-1.259346	0.000000	-1.733269
6	-1.259345	1.203370	-1.030432
6	-1.259346	1.130516	0.359250
7	-1.259347	0.000000	1.017151
6	-1.259346	-1.130516	0.359250
6	-1.259344	-1.203370	-1.030432
9	-1.259346	2.250137	1.055549
9	-1.259342	2.356135	-1.670782
9	-1.259347	0.000001	-3.045188
9	-1.259345	-2.356135	-1.670782
9	-1.259343	-2.250137	1.055551

6.9.3 **TS1**

Energy(PBE0-D3(BJ)/def2-TZVPP) = -2315.90177420252

6	1.830609	0.792445	2.499703
7	1.316293	0.128003	1.414019
6	0.349932	-0.705525	1.840277
6	1.174092	0.361549	3.604224
1	1.291497	0.651654	4.642403
1	2.640952	1.508492	2.408475
7	0.265154	-0.587069	3.193282
6	-0.656771	-1.221284	4.183767
51	-0.829979	-1.952908	0.513304
7	1.581378	-0.691211	-1.021825
6	0.527599	-1.516295	-1.121675
6	1.779775	-1.766641	-2.939057
7	0.635365	-2.185504	-2.301926
1	2.091625	-2.144258	-3.905509
1	3.267783	-0.255570	-2.286830
6	2.364042	-0.837940	-2.138313
6	-0.263583	-3.252609	-2.829415
5	1.821983	0.507674	-0.035987
6	-1.711940	-2.797881	-2.698715
1	-2.041539	-2.716741	-1.655028
1	-1.872085	-1.826785	-3.189589
1	-2.369131	-3.541324	-3.170542
6	0.037134	-3.481651	-4.308330
1	-0.081639	-2.557286	-4.892585
1	1.045736	-3.886560	-4.471628
1	-0.680001	-4.220954	-4.690151
6	-0.001053	-4.540231	-2.049471
1	1.048216	-4.850068	-2.163584
1	-0.212592	-4.413341	-0.977128
1	-0.647738	-5.342344	-2.432982
6	-1.304295	-2.479913	3.628597
1	-2.038862	-2.256655	2.843195
1	-0.557418	-3.197499	3.260540
1	-1.863181	-2.961679	4.442936
6	-1.727752	-0.190989	4.543196
1	-1.276781	0.709805	4.985120
1	-2.294112	0.099875	3.648588
1	-2.423357	-0.623135	5.277164
6	0.159672	-1.613794	5.417259
1	0.984210	-2.288194	5.141936
1	0.573377	-0.744909	5.946259
1	-0.499371	-2.141242	6.120755
6	1.039750	1.809829	-0.603779
6	1.047102	3.023189	0.104252
6	0.429409	1.835256	-1.865886
6	0.496258	4.190045	-0.420052
6	-0.125792	2.999349	-2.397860
6	-0.100081	4.202154	-1.686701
1	1.512761	3.071395	1.092822

1	0.385750	0.924401	-2.470449
1	0.528553	5.114231	0.165600
1	-0.595288	2.970397	-3.385997
6	3.430843	0.721729	0.060868
6	4.234024	-0.282142	0.632019
6	4.096496	1.842463	-0.445871
6	5.618531	-0.169578	0.699184
6	5.488309	1.963650	-0.383380
6	6.275618	0.962510	0.191378
1	3.758354	-1.181835	1.037557
1	3.516414	2.647271	-0.906242
1	6.207617	-0.972847	1.153141
1	5.972600	2.856507	-0.791038
6	-0.746988	5.440419	-2.232518
1	-0.301956	6.352552	-1.808644
1	-0.668539	5.489889	-3.328820
1	-1.821334	5.449827	-1.981713
6	7.770008	1.083591	0.270185
1	8.113787	1.088541	1.317307
1	8.265535	0.232043	-0.223019
1	8.124154	2.008104	-0.207313
6	-2.872892	-0.261481	0.373846
6	-2.548376	1.119507	0.480927
6	-3.640660	-0.566012	-0.784017
6	-2.863538	1.987698	-0.539553
6	-3.818298	0.379083	-1.766581
7	-3.468388	1.645742	-1.659813
9	-1.887897	1.542775	1.565051
9	-4.064839	-1.825029	-0.950891
9	-2.581343	3.276980	-0.383373
9	-4.416379	0.015207	-2.899592
9	-3.314868	-0.849280	1.548295

6.9.4 **INT1**

Energy(PBE0-D3(BJ)/def2-TZVPP) = -2315.96183763704

6	1.680541	0.365341	2.609863
7	1.082949	-0.025753	1.436515
6	0.034625	-0.824385	1.709161
6	0.998570	-0.213527	3.628447
1	1.163682	-0.158860	4.699845
1	2.548405	1.016690	2.631737
7	-0.012952	-0.958593	3.062828
6	-0.899575	-1.769626	3.945334
51	-1.394304	-1.632919	0.035126
7	1.359121	-0.572857	-1.065913
6	0.405721	-1.507644	-1.222076
6	1.839102	-1.702142	-2.899193
7	0.711914	-2.238067	-2.326531
1	2.286076	-2.096790	-3.804245
1	3.104188	-0.023411	-2.203651
6	2.239975	-0.672847	-2.111069
6	0.047225	-3.438190	-2.930605
5	1.600119	0.522790	0.060110
6	-0.866360	-2.954236	-4.055259
1	-1.646914	-2.309143	-3.630322
1	-0.290597	-2.403233	-4.814299
1	-1.337452	-3.820829	-4.542540
6	1.137078	-4.363310	-3.480064
1	1.666858	-3.934856	-4.341551
1	1.869437	-4.621529	-2.700512
1	0.657731	-5.290085	-3.823808
6	-0.744560	-4.212435	-1.883871
1	-0.139259	-4.420139	-0.988203
1	-1.663355	-3.675970	-1.620704
1	-1.041715	-5.175854	-2.320967
6	-1.939315	-2.548151	3.159084
1	-2.625883	-1.885875	2.615238
1	-1.477402	-3.254157	2.455701
1	-2.537689	-3.129489	3.874574
6	-1.606685	-0.816667	4.911579
1	-0.893508	-0.276873	5.550587
1	-2.206404	-0.081526	4.356727
1	-2.276208	-1.392470	5.566586
6	-0.017566	-2.764076	4.705304
1	0.515416	-3.420206	4.000857
1	0.722181	-2.257317	5.340897
1	-0.648548	-3.388969	5.353488
6	0.873989	1.915651	-0.315340
6	0.232517	2.704887	0.649220
6	0.937402	2.443570	-1.612951
6	-0.333978	3.938603	0.334440
6	0.370403	3.674453	-1.937707
6	-0.287110	4.443265	-0.970280
1	0.153563	2.343309	1.678894

1	1.429876	1.874087	-2.406590
1	-0.842222	4.515059	1.113711
1	0.425454	4.042466	-2.966787
6	3.214856	0.692411	0.199519
6	4.014326	-0.419758	0.523172
6	3.878557	1.911459	0.034252
6	5.393323	-0.319827	0.667679
6	5.265771	2.021663	0.178131
6	6.049663	0.909949	0.495906
1	3.540055	-1.396909	0.666403
1	3.300661	2.806136	-0.214128
1	5.979289	-1.209593	0.919198
1	5.748221	2.994372	0.040649
6	-0.976860	5.726009	-1.328143
1	-0.934460	6.453908	-0.504330
1	-0.538848	6.186348	-2.225795
1	-2.042985	5.537638	-1.541967
6	7.539485	1.014110	0.649586
1	8.060754	0.378152	-0.084191
1	7.886460	2.047723	0.509953
1	7.861962	0.677205	1.647893
6	-2.050025	0.447870	-0.194130
6	-2.745966	1.052670	0.840344
6	-2.027592	1.140490	-1.398356
6	-3.369799	2.280656	0.644768
6	-2.687677	2.362989	-1.494052
7	-3.344048	2.908555	-0.501042
9	-2.840925	0.464147	2.036446
9	-1.385433	0.685449	-2.464598
9	-4.026160	2.849254	1.645991
9	-2.673489	3.018647	-2.646012
9	-2.490677	-1.731945	-1.783901

6.9.5 **INT2**

Energy(PBE0-D3(BJ)/def2-TZVPP) = -2315.96919703204

6	-1.891557	-0.300478	-2.422356
7	-1.220392	-0.352385	-1.224932
6	-0.325336	-1.349619	-1.284165
6	-1.407390	-1.289861	-3.213938
1	-1.684357	-1.577899	-4.222990
1	-2.665707	0.434494	-2.617503
7	-0.439397	-1.949348	-2.491036
6	0.201704	-3.178072	-3.049619
51	0.949597	-1.876133	0.404736
7	-1.289542	0.084087	1.328633
6	-0.359166	-0.779784	1.749725
6	-1.488118	-0.096181	3.522343
7	-0.468153	-0.910590	3.091902
1	-1.782769	-0.011717	4.561699
1	-2.815057	1.226901	2.335398
6	-1.998504	0.517162	2.421395
6	0.324208	-1.815310	3.981255
5	-1.522334	0.722401	-0.103006
6	1.816183	-1.590975	3.739398
1	2.144151	-1.930512	2.745648
1	2.075448	-0.529583	3.848986
1	2.387323	-2.175576	4.473852
6	0.025596	-1.474740	5.438403
1	0.281135	-0.431323	5.675130
1	-1.025720	-1.659903	5.700179
1	0.642534	-2.127539	6.070878
6	-0.096523	-3.257389	3.700152
1	-1.141082	-3.409867	4.012399
1	-0.033626	-3.510052	2.632015
1	0.540216	-3.943606	4.277047
6	-0.909570	-4.210215	-3.254238
1	-1.401801	-4.423855	-2.293692
1	-1.666447	-3.863261	-3.971798
1	-0.474428	-5.142452	-3.641417
6	1.235379	-3.761787	-2.101922
1	2.071590	-3.069513	-1.929066
1	0.768121	-4.061359	-1.153127
1	1.653541	-4.664121	-2.569400
6	0.871964	-2.801430	-4.370270
1	0.147343	-2.438039	-5.112412
1	1.631396	-2.023173	-4.207216
1	1.364922	-3.689794	-4.789782
6	-0.562164	2.008489	-0.291130
6	0.096958	2.286760	-1.496644
6	-0.384785	2.936613	0.744806
6	0.911012	3.406886	-1.650512
6	0.428448	4.059251	0.600735
6	1.104760	4.310045	-0.598467
1	-0.001603	1.598021	-2.341117

1	-0.876635	2.774619	1.708263
1	1.426571	3.573266	-2.601459
1	0.555891	4.747550	1.441853
6	-3.102791	1.079948	-0.202883
6	-4.066708	0.067951	-0.039367
6	-3.582585	2.368574	-0.454598
6	-5.429636	0.330579	-0.119678
6	-4.951811	2.641399	-0.537017
6	-5.900851	1.629571	-0.370431
1	-3.738855	-0.958836	0.157174
1	-2.870244	3.187607	-0.590175
1	-6.148660	-0.483966	0.013720
1	-5.289119	3.663480	-0.735455
6	2.051875	5.464672	-0.736964
1	1.992181	5.921747	-1.736163
1	1.856422	6.242421	0.015379
1	3.092056	5.123496	-0.598542
6	-7.373471	1.909898	-0.452070
1	-7.876839	1.671267	0.498872
1	-7.569204	2.966317	-0.684076
1	-7.853819	1.294030	-1.229200
6	2.076716	0.124136	-0.018435
6	2.355477	1.141954	0.878534
6	2.754247	0.217729	-1.223023
6	3.239617	2.163093	0.538354
6	3.627731	1.267414	-1.480790
7	3.866230	2.216078	-0.610206
9	2.576467	-0.711417	-2.178585
9	1.783843	1.190344	2.086955
9	3.477756	3.139185	1.406480
9	4.255299	1.336774	-2.649101
9	-0.557293	-3.392100	0.569566

6.9.6 **Sb(III)-F species 15**

Energy(PBE0-D3(BJ)/def2-TZVPP) = -1771.53494355964

6	-1.869754	-0.339297	-2.414460
7	-1.180547	-0.360368	-1.226659
6	-0.276695	-1.349977	-1.279880
6	-1.383552	-1.336050	-3.197321
1	-1.675469	-1.646207	-4.194854
1	-2.657241	0.380661	-2.612113
7	-0.390036	-1.962974	-2.480255
6	0.251730	-3.214127	-2.981180
51	1.041744	-1.819506	0.397538
7	-1.275834	0.056508	1.324227
6	-0.385281	-0.850320	1.746322
6	-1.541673	-0.169657	3.506683
7	-0.534598	-1.003591	3.083717
1	-1.862455	-0.100410	4.539467
1	-2.789877	1.225801	2.317348
6	-1.999717	0.487217	2.407360
6	0.225318	-1.933049	3.974658
5	-1.469769	0.717392	-0.102916
6	1.722206	-1.685979	3.796326
1	2.082210	-1.987303	2.802002
1	1.965561	-0.624661	3.951627
1	2.277329	-2.285938	4.530803
6	-0.139904	-1.648958	5.428304
1	0.099503	-0.614979	5.717519
1	-1.201802	-1.846369	5.632397
1	0.450085	-2.324072	6.063031
6	-0.162316	-3.368600	3.624580
1	-1.228985	-3.532644	3.841390
1	0.002341	-3.595332	2.561645
1	0.426584	-4.064885	4.239147
6	-0.756535	-4.346140	-2.777704
1	-0.990289	-4.433267	-1.706572
1	-1.683475	-4.157902	-3.339327
1	-0.325254	-5.294994	-3.128599
6	1.528929	-3.529856	-2.217492
1	2.228020	-2.679632	-2.209944
1	1.299064	-3.861452	-1.196037
1	2.031688	-4.367945	-2.719877
6	0.594138	-3.024772	-4.458600
1	-0.297081	-2.893900	-5.087049
1	1.257602	-2.158166	-4.597657
1	1.117041	-3.922716	-4.815711
6	-0.499741	1.997786	-0.273111
6	-0.036742	2.405133	-1.532689
6	-0.159854	2.820172	0.809358
6	0.726037	3.559424	-1.702971
6	0.604784	3.976377	0.648933
6	1.067807	4.369121	-0.612011
1	-0.271318	1.804010	-2.416775

1	-0.494056	2.557056	1.817930
1	1.067244	3.838695	-2.705125
1	0.848815	4.588284	1.523244
6	-3.042017	1.115326	-0.220409
6	-4.037670	0.130998	-0.078346
6	-3.482518	2.420742	-0.458520
6	-5.391556	0.434535	-0.168205
6	-4.842200	2.735060	-0.550594
6	-5.822266	1.749744	-0.406914
1	-3.742766	-0.907319	0.109148
1	-2.745319	3.220309	-0.575366
1	-6.135332	-0.360183	-0.051800
1	-5.147097	3.769367	-0.737951
6	1.920109	5.593029	-0.788424
1	1.675321	6.124364	-1.720652
1	1.798919	6.293023	0.051181
1	2.989587	5.326943	-0.841781
6	-7.285077	2.074482	-0.499751
1	-7.806284	1.834654	0.441207
1	-7.447219	3.139997	-0.715637
1	-7.774274	1.486472	-1.292819
9	-0.258688	-3.488897	0.479084
9	1.770435	0.070246	0.083547

6.9.7 **Sb(III) diaryl species 16**

Energy(PBE0-D3(BJ)/def2-TZVPP) = -2860.378769483

6	1.829143	0.349080	2.712976
7	1.212266	-0.046363	1.541773
6	0.149822	-0.824968	1.858961
6	1.139081	-0.200049	3.760413
1	1.314782	-0.128495	4.836255
1	2.715612	0.987502	2.710885
7	0.094000	-0.931977	3.220514
6	-0.830592	-1.710960	4.120656
51	-1.165772	-1.724198	0.339227
7	1.504543	-0.677125	-0.952656
6	0.415433	-1.452824	-1.169702
6	1.839272	-1.775001	-2.858887
7	0.605034	-2.131476	-2.341081
1	2.227087	-2.182972	-3.795249
1	3.348909	-0.343393	-2.040021
6	2.392908	-0.869819	-1.992817
6	-0.280502	-3.105359	-3.074369
5	1.734870	0.466143	0.129952
6	-0.409848	-2.610261	-4.527598
1	-0.831367	-1.585875	-4.547986
1	0.559442	-2.609033	-5.060562
1	-1.094562	-3.284521	-5.076764
6	0.386677	-4.489432	-3.009915
1	1.382729	-4.480714	-3.493666
1	0.505436	-4.816547	-1.957989
1	-0.246527	-5.228594	-3.538014
6	-1.681052	-3.164417	-2.462241
1	-1.693192	-3.655780	-1.470966
1	-2.145372	-2.159878	-2.409976
1	-2.316177	-3.786525	-3.121354
6	-2.008309	-2.312349	3.353666
1	-2.612688	-1.533959	2.850247
1	-1.685103	-3.088077	2.633340
1	-2.666849	-2.818898	4.084804
6	-1.379344	-0.740035	5.182105
1	-0.583181	-0.333123	5.833785
1	-1.908906	0.102630	4.696260
1	-2.096726	-1.281900	5.827805
6	-0.004143	-2.840607	4.760667
1	0.393038	-3.518517	3.978885
1	0.843911	-2.441190	5.349969
1	-0.649774	-3.429111	5.441075
6	0.931279	1.797436	-0.329190
6	0.325254	2.673249	0.596143
6	0.875699	2.179080	-1.687760
6	-0.334058	3.842257	0.188238
6	0.218649	3.345604	-2.105510
6	-0.413456	4.195804	-1.174701
1	0.336714	2.423747	1.670043

1	1.341423	1.538131	-2.453986
1	-0.819349	4.485680	0.940637
1	0.176408	3.596169	-3.178210
6	3.340516	0.733730	0.257593
6	4.236928	-0.305709	0.613526
6	3.912087	2.002793	0.033458
6	5.615471	-0.088468	0.732863
6	5.295550	2.229487	0.152743
6	6.175898	1.188704	0.503746
1	3.845817	-1.317046	0.800976
1	3.257542	2.844270	-0.243989
1	6.277476	-0.925636	1.010225
1	5.701562	3.237991	-0.030850
6	-1.201028	5.400030	-1.621612
1	-1.140097	6.227701	-0.886945
1	-0.856694	5.774754	-2.605806
1	-2.276684	5.137992	-1.726482
6	7.661110	1.416182	0.632913
1	8.229501	0.772570	-0.071133
1	7.931931	2.470093	0.427810
1	8.020319	1.161583	1.652246
6	-1.977204	0.484836	-0.118864
6	-2.623602	1.187175	0.894995
6	-2.163200	1.005827	-1.396130
6	-3.382760	2.332307	0.616888
6	-2.920162	2.171709	-1.591432
7	-3.526652	2.811210	-0.606714
9	-2.519608	0.783009	2.194577
9	-1.605906	0.414149	-2.486120
9	-3.990545	3.000648	1.615636
9	-3.041204	2.693544	-2.827577
6	0.105249	-3.825075	0.805041
6	-0.619096	-5.013385	0.791111
6	1.469691	-3.987148	1.017291
6	0.009916	-6.256884	0.967522
6	2.033231	-5.263810	1.185276
7	1.312784	-6.371590	1.157856
9	2.306051	-2.916153	1.072656
9	3.357966	-5.396995	1.384478
9	-0.712456	-7.391984	0.948871
9	-1.969434	-5.000253	0.598924

6.9.8 **TMS_2Pyrdz S6**

Energy(PBE0-D3(BJ)/def2-TZVPP) = -1082.28489965447

6	0.725449	2.067825	-2.440829
6	0.534173	3.180621	-1.716169
7	0.615488	3.218339	-0.306901
6	0.939449	1.960846	0.257920
6	1.130721	0.848044	-0.466733
7	1.046245	0.809680	-1.875747
1	0.634894	2.109198	-3.527706
1	0.298013	4.118726	-2.219793
1	1.033054	1.920116	1.344562
1	1.369904	-0.089413	0.036674
14	0.759241	4.713209	0.630193
14	0.902698	-0.685162	-2.812648
6	-0.048670	6.082539	-0.360069
1	0.501683	6.301803	-1.288380
1	-1.088378	5.830206	-0.621308
1	-0.062332	7.003468	0.244864
6	-0.124153	4.457275	2.265089
1	-1.183194	4.199736	2.106639
1	0.340828	3.657296	2.862481
1	-0.078969	5.384176	2.859551
6	2.567439	5.116333	0.952400
1	2.669465	6.028382	1.562934
1	3.058753	4.289801	1.490904
1	3.106550	5.274744	0.004597
6	1.709317	-2.054724	-1.821624
1	1.158575	-2.273049	-0.893319
1	1.722297	-2.975932	-2.426140
1	2.749185	-1.803199	-1.560230
6	-0.905242	-1.088231	-3.136132
1	-1.006416	-2.000411	-3.746642
1	-1.445187	-1.246810	-2.188841
1	-1.396227	-0.261949	-3.675308
6	1.787424	-0.430029	-4.446978
1	2.846313	-0.172528	-4.287310
1	1.743016	-1.357215	-5.041041
1	1.323345	0.369810	-5.045242

6.9.9 **Pyrz**

Energy(PBE0-D3(BJ)/def2-TZVPP) = -264.119608728201

6	0.831521	2.013725	-2.421683
6	0.831521	3.202418	-1.689182
7	0.831521	3.204305	-0.357792
6	0.831521	2.014248	0.239112
6	0.831521	0.825555	-0.493389
7	0.831521	0.823668	-1.824779
1	0.831522	2.035651	-3.516865
1	0.831520	4.170837	-2.201087
1	0.831521	1.992323	1.334294
1	0.831520	-0.142864	0.018515

6.9.10 **TMSF**

Energy(PBE0-D3(BJ)/def2-TZVPP) = -508.928550436732

9	-1.462792	2.458209	-2.056962
14	-1.122861	3.338966	-0.697733
6	0.717809	3.631406	-0.701454
1	1.010535	4.216055	0.185680
1	1.269807	2.678576	-0.678532
1	1.028283	4.192871	-1.596659
6	-1.670320	2.302015	0.751043
1	-2.750168	2.091045	0.700518
1	-1.128906	1.343229	0.777460
1	-1.470536	2.832506	1.696058
6	-2.090823	4.925512	-0.836648
1	-1.792838	5.494842	-1.731170
1	-3.172359	4.725582	-0.894528
1	-1.908285	5.558873	0.046557

6.9.11 **INT11**

Energy(PBE0-D3(BJ)/def2-TZVPP) = -2860.36217030537

6	1.638814	0.356357	2.519036
7	1.016526	0.004838	1.331854
6	-0.011557	-0.848352	1.592114
6	0.999382	-0.301736	3.533657
1	1.197123	-0.303937	4.608751
1	2.495866	1.033745	2.550797
7	-0.011179	-1.053905	2.949952
6	-0.846607	-1.972561	3.794477
51	-1.537663	-1.500239	-0.150778
7	1.258777	-0.460747	-1.206139
6	0.361030	-1.477495	-1.289965
6	1.878070	-1.765583	-2.903222
7	0.762821	-2.317035	-2.297687
1	2.397330	-2.247601	-3.735354
1	3.013004	0.078748	-2.362787
6	2.181610	-0.616266	-2.224051
6	0.366737	-3.740044	-2.619131
5	1.512724	0.616711	-0.040143
6	0.085459	-3.855323	-4.126411
1	-0.727109	-3.175775	-4.439131
1	0.983655	-3.625524	-4.731664
1	-0.213611	-4.896299	-4.354283
6	1.562310	-4.629974	-2.222335
1	2.469555	-4.389690	-2.809194
1	1.793589	-4.511183	-1.145047
1	1.303608	-5.690088	-2.410553
6	-0.856506	-4.191642	-1.821796
1	-0.671252	-4.161998	-0.729645
1	-1.760180	-3.605444	-2.072548
1	-1.065328	-5.245973	-2.085278
6	-1.863464	-2.743562	2.953974
1	-2.600366	-2.071885	2.476550
1	-1.370760	-3.358332	2.175950
1	-2.417731	-3.427371	3.625100
6	-1.580726	-1.121401	4.846871
1	-0.871409	-0.599491	5.518236
1	-2.222807	-0.365681	4.355859
1	-2.218383	-1.777000	5.471107
6	0.103237	-2.980421	4.470289
1	0.656830	-3.562621	3.706793
1	0.835508	-2.478819	5.131725
1	-0.486919	-3.684348	5.088811
6	0.815141	2.045570	-0.334051
6	0.247726	2.830168	0.693452
6	0.866009	2.631423	-1.618090
6	-0.259466	4.115493	0.451585
6	0.356696	3.913845	-1.870439

6	-0.224781	4.680673	-0.839700
1	0.185198	2.425153	1.716369
1	1.302439	2.065040	-2.456831
1	-0.708003	4.690214	1.278581
1	0.398829	4.328313	-2.891113
6	3.141877	0.760151	0.080813
6	3.932119	-0.374202	0.393240
6	3.830479	1.975639	-0.095581
6	5.324342	-0.299219	0.516704
6	5.230615	2.060527	0.025664
6	6.005443	0.926591	0.333366
1	3.439003	-1.349870	0.543955
1	3.260824	2.887292	-0.336105
1	5.902662	-1.206102	0.759978
1	5.733231	3.030813	-0.121348
6	-0.843342	6.026407	-1.117826
1	-0.789259	6.694540	-0.235387
1	-0.357753	6.533238	-1.975361
1	-1.918588	5.908603	-1.374668
6	7.505681	1.004347	0.467712
1	8.010274	0.314576	-0.241075
1	7.879661	2.028476	0.274237
1	7.835048	0.706456	1.485568
6	-2.152895	0.640897	-0.146537
6	-2.796296	1.101489	1.005921
6	-2.169604	1.514319	-1.239858
6	-3.402393	2.366772	1.025569
6	-2.828753	2.750786	-1.130455
7	-3.424055	3.161369	-0.026979
9	-2.863651	0.330146	2.114333
9	-1.574941	1.211412	-2.401331
9	-4.000321	2.799350	2.145357
9	-2.874084	3.565949	-2.196098
6	-2.776975	-1.427920	-2.290870
6	-4.127605	-1.721332	-2.109374
6	-2.407276	-1.233021	-3.619792
6	-5.008084	-1.832445	-3.198074
6	-3.341048	-1.384158	-4.658394
7	-4.613403	-1.673311	-4.449083
9	-1.136526	-0.908889	-3.971109
9	-2.945590	-1.241717	-5.938716
9	-6.308496	-2.113580	-2.992262
9	-4.628955	-1.918406	-0.858163

6.9.12 **TS12**

Energy(PBE0-D3(BJ)/def2-TZVPP) = -2860.34297084334

6	1.828033	0.293214	2.668725
7	1.347142	-0.125976	1.458647
6	0.235779	-0.849794	1.662304
6	1.005020	-0.195227	3.632748
1	1.049263	-0.078036	4.710387
1	2.719021	0.906706	2.755060
7	0.024627	-0.921253	3.003348
6	-1.050757	-1.574253	3.815398
51	-0.854692	-1.848477	0.031990
7	1.835194	-0.660661	-1.018643
6	0.856415	-1.537773	-1.284868
6	2.309288	-1.618084	-2.947986
7	1.141111	-2.149684	-2.460436
1	2.750386	-1.933653	-3.887802
1	3.631695	-0.079462	-2.054416
6	2.740569	-0.698841	-2.045933
6	0.397083	-3.170047	-3.262657
5	1.966044	0.431817	0.121893
6	-0.093920	-2.485469	-4.539251
1	-0.777773	-1.662085	-4.291779
1	0.741564	-2.089342	-5.134074
1	-0.633605	-3.218269	-5.155997
6	1.363430	-4.313344	-3.576501
1	2.211473	-3.985739	-4.193337
1	1.751111	-4.757218	-2.647540
1	0.824894	-5.090883	-4.136014
6	-0.792871	-3.734642	-2.509776
1	-0.498032	-4.227614	-1.572429
1	-1.564122	-2.971936	-2.315890
1	-1.265691	-4.493831	-3.147968
6	-1.876518	-2.548315	2.993840
1	-2.471923	-2.051341	2.217485
1	-1.256158	-3.341320	2.552444
1	-2.595573	-3.029915	3.670911
6	-1.945060	-0.464260	4.368584
1	-1.375514	0.231146	5.002184
1	-2.409296	0.099378	3.547956
1	-2.741334	-0.911099	4.981144
6	-0.378286	-2.355740	4.946366
1	0.327851	-3.095785	4.541407
1	0.155848	-1.704046	5.650601
1	-1.152926	-2.889763	5.513965
6	1.173698	1.774010	-0.302005
6	0.287399	2.435116	0.558433
6	1.401948	2.376652	-1.548516
6	-0.346157	3.622313	0.193632
6	0.774267	3.562337	-1.919390
6	-0.121786	4.205356	-1.056746
1	0.070544	2.019059	1.546727

1	2.087716	1.907660	-2.260501
1	-1.044518	4.097382	0.889325
1	0.970187	3.993439	-2.905908
6	3.550559	0.659324	0.384239
6	4.372853	-0.434139	0.713414
6	4.166900	1.913098	0.329335
6	5.731667	-0.283390	0.966175
6	5.532799	2.073569	0.582396
6	6.341232	0.980033	0.903511
1	3.934867	-1.436543	0.773028
1	3.566952	2.793428	0.082261
1	6.337754	-1.159320	1.218116
1	5.979208	3.071291	0.529135
6	-0.869420	5.432329	-1.485577
1	-1.104159	6.085450	-0.632138
1	-0.304946	6.014933	-2.228411
1	-1.827317	5.144003	-1.950690
6	7.810121	1.137786	1.170165
1	8.409529	0.580862	0.431751
1	8.115440	2.192815	1.128138
1	8.079530	0.741590	2.162183
6	-2.062447	-0.174956	-0.776717
6	-2.838135	0.556890	0.171119
6	-1.601518	0.698011	-1.809025
6	-3.366242	1.790200	-0.128044
6	-2.214618	1.907695	-2.028915
7	-3.102973	2.465483	-1.226509
9	-3.186312	-0.045521	1.329010
9	-0.714548	0.248005	-2.710600
9	-4.179797	2.371841	0.755463
9	-1.897332	2.594348	-3.127324
6	-3.379835	-1.448863	-1.625018
6	-4.283168	-2.138586	-0.832496
6	-3.845244	-1.157427	-2.897239
6	-5.549721	-2.468823	-1.301766
6	-5.131499	-1.519311	-3.289487
7	-5.966193	-2.155714	-2.504931
9	-3.072685	-0.537896	-3.803353
9	-5.553811	-1.227875	-4.515709
9	-6.391229	-3.142637	-0.523976
9	-3.957186	-2.537471	0.411837

6.9.13 **BiPy-F8 10a**

Energy(PBE0-D3(BJ)/def2-TZVPP) = -1288.52449963549

6	-1.259332	0.000000	-1.771509
6	-1.259315	1.193362	-1.053195
6	-1.259331	1.130529	0.338641
7	-1.259356	-0.000002	0.994371
6	-1.259372	-1.130532	0.338640
6	-1.259364	-1.193363	-1.053196
9	-1.259322	2.250441	1.034159
9	-1.259294	2.355907	-1.676732
9	-1.259383	-2.355908	-1.676735
9	-1.259397	-2.250446	1.034155
6	-1.259336	0.000001	-3.249379
6	-0.066021	0.000032	-3.967710
6	-2.452656	-0.000019	-3.967701
6	-0.128946	0.000024	-5.359588
6	-2.389742	-0.000027	-5.359579
7	-1.259346	-0.000009	-6.015521
9	1.096731	0.000055	-3.344550
9	-3.615404	-0.000031	-3.344533
9	-3.509954	-0.000043	-6.054683
9	0.991262	0.000031	-6.054700

6.10 Defluorosilylation mechanism – xyz coordinates (in Å) and Energies (in Hartree)

6.10.1 **INT12**

Energy(PBE0-D3(BJ)/def2-TZVPP) = -2625.18321197526

6	-1.917650	-0.327035	-2.413178
7	-1.312459	-0.484334	-1.190884
6	-0.377296	-1.441726	-1.297622
6	-1.343470	-1.198978	-3.278692
1	-1.534948	-1.366063	-4.333257
1	-2.702420	0.404840	-2.575860
7	-0.387062	-1.896698	-2.576712
6	0.499077	-2.870546	-3.283732
51	0.617027	-2.267975	0.461078
7	-1.269995	0.037117	1.364115
6	-0.379381	-0.871875	1.774412
6	-1.214229	0.101725	3.574586
7	-0.325985	-0.850041	3.127206
1	-1.370836	0.321902	4.623895
1	-2.568602	1.409312	2.404163
6	-1.802773	0.643308	2.476070
6	0.622613	-1.606557	4.003124
5	-1.577197	0.610500	-0.081316
6	2.050359	-1.195028	3.648094
1	2.291286	-1.401503	2.594661
1	2.192144	-0.119937	3.816740
1	2.754637	-1.755432	4.279153
6	0.350912	-1.252182	5.461661
1	0.523893	-0.185419	5.663064
1	-0.672241	-1.518218	5.764917
1	1.047804	-1.827292	6.086445
6	0.423391	-3.109042	3.830124
1	-0.591781	-3.412441	4.116297
1	0.612255	-3.451818	2.801872
1	1.138224	-3.639383	4.474441
6	-0.366709	-3.804860	-4.130256
1	-1.090457	-4.349816	-3.508546
1	-0.912003	-3.272478	-4.921172
1	0.288524	-4.539250	-4.618776
6	1.301724	-3.704679	-2.298863
1	2.027448	-3.096967	-1.742202
1	0.646577	-4.258452	-1.607926
1	1.875968	-4.450215	-2.866026
6	1.440765	-2.064537	-4.180639
1	0.877249	-1.475920	-4.919206
1	2.060937	-1.386411	-3.579844
1	2.101808	-2.753642	-4.726157
6	-0.608656	1.877688	-0.389530
6	0.177824	1.969266	-1.546946
6	-0.530995	2.966220	0.492337

6	1.003087	3.062870	-1.801954
6	0.295055	4.061573	0.250408
6	1.088360	4.128527	-0.900666
1	0.169992	1.156885	-2.278492
1	-1.120029	2.962682	1.413567
1	1.608751	3.079975	-2.713457
1	0.335442	4.879338	0.976720
6	-3.161283	0.967220	-0.125496
6	-4.112208	-0.040989	0.114570
6	-3.663059	2.240048	-0.415829
6	-5.479855	0.207495	0.075912
6	-5.036677	2.500272	-0.455513
6	-5.971362	1.491294	-0.208843
1	-3.771070	-1.056734	0.335345
1	-2.965450	3.057422	-0.619223
1	-6.185039	-0.607170	0.269043
1	-5.389151	3.510626	-0.685435
6	2.037956	5.266408	-1.131853
1	2.125933	5.511158	-2.200982
1	1.726423	6.171824	-0.590694
1	3.046998	4.998863	-0.774976
6	-7.448165	1.759252	-0.243446
1	-7.910319	1.565405	0.738167
1	-7.661076	2.801935	-0.518560
1	-7.955658	1.103239	-0.968725
6	2.235914	0.064815	0.225926
6	2.315124	1.218518	0.980623
6	3.125595	0.056017	-0.825413
6	3.199778	2.246773	0.668802
6	3.975639	1.121610	-1.101414
7	4.015801	2.202067	-0.358430
9	3.207480	-1.016897	-1.661443
9	1.533874	1.411244	2.068602
9	3.250831	3.347110	1.425424
9	4.799492	1.080219	-2.152951
14	-1.596911	-3.808483	0.710483
6	-1.034904	-5.574711	1.088375
1	-0.462842	-5.623218	2.028451
1	-1.906406	-6.244243	1.183864
1	-0.393738	-5.962327	0.280231
6	-2.614774	-3.852674	-0.881322
1	-2.042165	-4.294655	-1.709158
1	-3.512506	-4.472939	-0.718655
1	-2.944005	-2.848472	-1.190015
6	-2.760563	-3.234084	2.090524
1	-2.980494	-2.157868	2.028782
1	-3.714146	-3.781696	2.000512
1	-2.349559	-3.435990	3.089302

6.10.2 **INT13**

Energy(PBE0-D3(BJ)/def2-TZVPP) = -2625.16473568672

6	-1.699223	-0.104723	-2.719785
7	-1.141731	-0.241545	-1.465457
6	-0.616999	-1.471268	-1.323732
6	-1.514643	-1.281521	-3.375664
1	-1.818862	-1.575793	-4.374886
1	-2.217170	0.794677	-3.042010
7	-0.858483	-2.117892	-2.494919
6	-0.506470	-3.533110	-2.760084
51	1.409820	-1.727859	0.278745
7	-1.010347	0.131788	1.098628
6	-0.119663	-0.780046	1.547969
6	-1.394601	-0.169665	3.260175
7	-0.374279	-0.998394	2.865433
1	-1.781988	-0.143638	4.271298
1	-2.577826	1.255775	2.059181
6	-1.783017	0.525395	2.163251
6	0.288116	-1.945419	3.811210
5	-1.344977	0.804577	-0.330679
6	1.536476	-1.263197	4.359463
1	2.201130	-0.960744	3.539936
1	1.262975	-0.363946	4.930789
1	2.083187	-1.948060	5.023174
6	-0.670493	-2.262991	4.959588
1	-0.875000	-1.388565	5.591843
1	-1.621411	-2.674148	4.589685
1	-0.193424	-3.019797	5.596896
6	0.606082	-3.258133	3.102771
1	-0.281905	-3.652715	2.587485
1	1.433475	-3.169939	2.386517
1	0.929707	-3.994797	3.850814
6	-1.388271	-4.075797	-3.882752
1	-2.456235	-3.959274	-3.644718
1	-1.182781	-3.584923	-4.845122
1	-1.179241	-5.147467	-4.008478
6	-0.762557	-4.343599	-1.490546
1	-0.122058	-4.004317	-0.664375
1	-1.813690	-4.254876	-1.177748
1	-0.543072	-5.403988	-1.683637
6	0.962408	-3.627129	-3.170741
1	1.157184	-3.012702	-4.061962
1	1.620628	-3.280432	-2.361863
1	1.218596	-4.671111	-3.404422
6	-0.486205	2.159281	-0.538533
6	-0.029199	2.572416	-1.798094
6	-0.257455	3.047426	0.523764
6	0.628749	3.787090	-1.985282
6	0.405820	4.260205	0.347668
6	0.871235	4.652132	-0.912876
1	-0.170938	1.923360	-2.666282

1	-0.592314	2.783903	1.531222
1	0.982496	4.060755	-2.984236
1	0.577176	4.913408	1.209033
6	-2.950267	1.120897	-0.324053
6	-3.865435	0.053244	-0.273008
6	-3.495985	2.406316	-0.389136
6	-5.240360	0.258003	-0.275295
6	-4.878604	2.622475	-0.396249
6	-5.777063	1.554595	-0.337122
1	-3.484244	-0.973222	-0.230520
1	-2.826665	3.270524	-0.434262
1	-5.918189	-0.600713	-0.231775
1	-5.266276	3.644745	-0.449453
6	1.655362	5.918034	-1.095447
1	1.548870	6.318326	-2.114343
1	1.345929	6.694574	-0.380279
1	2.729465	5.729355	-0.927214
6	-7.262691	1.772136	-0.342686
1	-7.729723	1.359518	0.566254
1	-7.512485	2.841284	-0.397116
1	-7.736791	1.267615	-1.200077
6	2.079290	0.150159	-0.632772
6	2.406067	1.266685	0.128068
6	2.452958	0.198287	-1.966888
6	3.130000	2.305703	-0.446662
6	3.145788	1.302910	-2.456011
7	3.489629	2.316077	-1.705282
9	2.190376	-0.810357	-2.796572
9	2.084297	1.351475	1.414336
9	3.509328	3.328375	0.307656
9	3.496456	1.338632	-3.734147
14	3.831472	-1.512239	1.515355
6	4.179525	-3.119907	2.462331
1	3.796463	-3.992864	1.909088
1	5.268333	-3.248226	2.585618
1	3.721378	-3.124098	3.462675
6	4.584702	-0.075552	2.500255
1	4.123786	0.050519	3.491320
1	5.658036	-0.283673	2.650053
1	4.494827	0.878676	1.961003
6	4.845820	-1.616874	-0.093377
1	4.929239	-0.627235	-0.569455
1	5.864987	-1.974034	0.131329
1	4.403947	-2.309561	-0.829787

6.10.3 **TS13**

Energy(PBE0-D3(BJ)/def2-TZVPP) = -2625.14952078588

6	-2.582756000	-0.937919000	-2.258386000
7	-1.702933000	-0.639489000	-1.245676000
6	-0.739672000	-1.578800000	-1.209859000
6	-2.156251000	-2.086052000	-2.853773000
1	-2.588871000	-2.646120000	-3.675652000
1	-3.450469000	-0.323028000	-2.477353000
7	-1.011251000	-2.472540000	-2.188043000
6	-0.167674000	-3.651236000	-2.516551000
51	1.207100000	-0.911205000	-0.158143000
7	-1.324468000	0.209503000	1.111413000
6	-0.289324000	-0.589605000	1.436985000
6	-1.577172000	-0.344304000	3.229114000
7	-0.462368000	-0.978715000	2.728479000
1	-1.919271000	-0.465589000	4.249758000
1	-2.998328000	1.012025000	2.205728000
6	-2.109972000	0.389580000	2.218654000
6	0.243104000	-2.071484000	3.468353000
5	-1.726306000	0.669332000	-0.356861000
6	1.622722000	-1.596540000	3.897049000
1	2.222652000	-1.301023000	3.031650000
1	1.543880000	-0.728464000	4.565381000
1	2.145475000	-2.406288000	4.425544000
6	-0.554585000	-2.438101000	4.718434000
1	-0.600862000	-1.609760000	5.439653000
1	-1.575646000	-2.765343000	4.474160000
1	-0.039172000	-3.274515000	5.210217000
6	0.304506000	-3.298657000	2.561951000
1	-0.708502000	-3.598092000	2.254487000
1	0.902119000	-3.111315000	1.660905000
1	0.766971000	-4.132469000	3.108886000
6	-0.916588000	-4.560093000	-3.485197000
1	-1.875996000	-4.895305000	-3.063929000
1	-1.101473000	-4.068652000	-4.451280000
1	-0.299367000	-5.448655000	-3.676683000
6	0.129694000	-4.418848000	-1.230596000
1	0.712724000	-3.811181000	-0.524226000
1	-0.803343000	-4.728137000	-0.736478000
1	0.716575000	-5.317652000	-1.468128000
6	1.122611000	-3.156262000	-3.170250000
1	0.899642000	-2.565556000	-4.071161000
1	1.710405000	-2.531043000	-2.480685000
1	1.747749000	-4.013587000	-3.459376000
6	-0.679125000	1.768646000	-0.905883000
6	-0.319803000	1.858232000	-2.258938000
6	-0.201384000	2.781592000	-0.061589000
6	0.480843000	2.892110000	-2.740647000
6	0.585414000	3.828921000	-0.539625000
6	0.945278000	3.904660000	-1.889751000
1	-0.663107000	1.088470000	-2.958119000

1	-0.446537000	2.752112000	1.005082000
1	0.752446000	2.918013000	-3.800853000
1	0.939635000	4.598708000	0.152015000
6	-3.248396000	1.237046000	-0.326849000
6	-4.329055000	0.397304000	0.001689000
6	-3.567613000	2.567067000	-0.625872000
6	-5.642068000	0.857680000	0.035802000
6	-4.882500000	3.038649000	-0.595089000
6	-5.945771000	2.194933000	-0.259922000
1	-4.138566000	-0.654847000	0.238237000
1	-2.766160000	3.260244000	-0.896224000
1	-6.451936000	0.168193000	0.294280000
1	-5.086812000	4.085563000	-0.840634000
6	1.774708000	5.040782000	-2.414740000
1	1.142489000	5.799180000	-2.907124000
1	2.325483000	5.541480000	-1.605945000
1	2.504197000	4.692914000	-3.160994000
6	-7.357644000	2.701303000	-0.195760000
1	-7.637873000	2.955860000	0.840488000
1	-7.487581000	3.608891000	-0.802763000
1	-8.073744000	1.942504000	-0.545204000
6	2.814211000	0.821634000	0.644373000
6	2.591818000	1.613724000	1.772987000
6	3.387888000	1.543208000	-0.409558000
6	2.993542000	2.940781000	1.825590000
6	3.771956000	2.866082000	-0.264816000
7	3.594472000	3.557163000	0.836707000
9	3.522270000	0.989541000	-1.621491000
9	1.986188000	1.109197000	2.855973000
9	2.763863000	3.647102000	2.929731000
9	4.334041000	3.497723000	-1.292763000
14	3.818297000	-1.296356000	0.955289000
6	3.714252000	-3.123856000	1.480489000
1	3.236141000	-3.719151000	0.687594000
1	4.751101000	-3.480301000	1.615605000
1	3.174080000	-3.296895000	2.419242000
6	4.879535000	-0.476733000	2.296197000
1	4.305252000	-0.253397000	3.207875000
1	5.695929000	-1.166818000	2.567041000
1	5.315708000	0.463655000	1.927529000
6	4.813244000	-1.359843000	-0.646853000
1	5.296274000	-0.405762000	-0.892575000
1	5.598218000	-2.116696000	-0.482761000
1	4.211882000	-1.679773000	-1.510982000

6.10.4 **PyrF4TMS S8**

Energy(PBE0-D3(BJ)/def2-TZVPP) = -1053.34244555772

6	-1.260972	0.054854	-1.766330
6	-0.889715	1.129246	-0.963580
6	-0.971580	1.027059	0.425224
7	-1.388114	-0.050480	1.036997
6	-1.745130	-1.081359	0.314322
6	-1.699084	-1.073722	-1.076675
9	-0.614723	2.059295	1.172029
9	-0.451326	2.271098	-1.482641
9	-2.081614	-2.167040	-1.734888
9	-2.162590	-2.164027	0.949403
14	-1.211789	0.026506	-3.690282
6	-0.581628	1.657775	-4.355524
1	0.440746	1.869993	-4.009491
1	-0.568283	1.599051	-5.456115
1	-1.230955	2.496032	-4.062850
6	-2.970376	-0.284495	-4.259805
1	-3.000358	-0.333030	-5.360295
1	-3.360572	-1.232116	-3.859791
1	-3.633852	0.532210	-3.934805
6	-0.059803	-1.371237	-4.172350
1	0.959764	-1.180294	-3.802474
1	-0.405097	-2.333728	-3.766921
1	-0.016894	-1.451589	-5.270584

6.11 Plausible mechanisms for defluorinative alkoxylation of pentafluoropyridine employing bismuthinidene 3

Analogous mechanisms for the formation of product **2n** under Bi catalysis are shown below. As discussed, relatively similar values for energetic barriers were found in all the mechanisms evaluated, further supporting the similar behaviour between stibinidene **1a** and bismuthinidene **3** in the defluoroalkoxylation process.

First, a redox mechanism involving Bi(I)-Bi(III) redox catalysis is presented in Figure S42. In a similar manner to the mechanism depicted in the main text (Figure 1), the reaction begins with the oxidative addition of Bi(I) species **3** to the C4–F bond of the pyridine ring, resulting in Bi(III) intermediate **INT1-Bi** (ΔG = –12.7 kcal mol^{-1}). This step is thermodynamically favourable, with a connecting transition state (**TS1-Bi**) located at +22.3 kcal mol^{-1} relative to the reactants. After oxidative addition, **INT1-Bi** undergoes isomerization to the more thermodynamically favoured species **INT2-Bi** (ΔG = –15.3 kcal mol^{-1}). Subsequent ligand metathesis with $PhOSiMe_3$ proceeds similarly in a stepwise manner. Transmetallation begins with silane-mediated fluoride abstraction, forming the fluorosilicate anion $[PhOSiMe_3F]^-$ and the corresponding cationic Bi(III) intermediate (**INT3-Bi**), as a contacted ion-pair. This step is endergonic by +19.8 kcal mol^{-1} and it shows a Gibbs activation energy of 20.6 kcal mol^{-1} (**TS2-Bi**, ΔG = 5.3 kcal mol^{-1}). It is worth mentioning that this activation barrier is 5.7 kcal mol^{-1} lower than the calculated for Sb(III) species. **INT3-Bi** (ΔG = +4.5 kcal mol^{-1}) rapidly evolves to Bi(III)–OPh species (**INT4-Bi,** ΔG = –22.1 kcal mol^{-1}) in a barrierless process (**TS3-Bi**). All attempts to locate a transition state were unsuccessful. Additionally, other plausible mechanistic scenarios for the conversion of **INT2-Bi** to **INT4-Bi** were systematically explored computationally (e.g., four-membered ring concerted transmetallation), but all attempts converged back to the silicate-derived pathway providing no viable alternative to the fluoride-abstraction-initiated manifold described above. To undergo metallomimetic concerted reductive elimination, **INT4-Bi** isomerises to **INT5-Bi** (ΔG = –15.8 kcal mol^{-1}). Finally, C–O reductive elimination from **INT5-Bi** yields (phenyloxy)tetrafluoropyridine **2n** and regenerates the low-valent Bi catalyst **3**, with a barrier (**TS4-Bi**) of +35.4 kcal mol^{-1}, similar to that observed for Sb species (+36.0 kcal mol^{-1}), and discarding a potential Bi(I)/Bi(III) redox cycle.

Second, a similar redox-neutral pathway to that discussed in the main text (Figure 2) was also evaluated (Enthalpy in parentheses). Following the pathway depicted in **Figure S42**, after oxidative addition and transmetallation, **INT4-Bi** is able to undergo S_NAr-type reactivity with pentafluoropyridine for C–O bond formation, with a transition state (**TS5-Bi**) located with a relative Gibbs free energy of ΔG = -2.6 kcal mol^{-1} and a low Gibbs activation barrier ($\Delta\Delta G$ = +19.5 kcal mol^{-1}) and leads to the corresponding Meisenheimer-like intermediate **INT6-Bi** (ΔG

= -3.0 kcal mol^{-1}). Subsequent C–F bond cleavage proceeds to Bi–F bond formation (**TS6-Bi**, ΔG = +0.1 kcal mol^{-1}), leading to **INT2-Bi** and the product **2n** in an overall thermodynamically favoured manner ($\Delta\Delta$G = -15.2 kcal mol^{-1}) when compared to the potential energy surface in Figure S42. The overall barrier of this process is 22.3 kcal mol^{-1}.

Finally, a S_NAr-type mechanism analogous to the one reported in the main text (Figure 3) in disclosed in Figure S44. In this case, **INT7-Bi** undergoes S_NAr-type reactivity with perfluoropyridine for C–O bond formation, with a transition state (**TS7-Bi**) located with a relative Gibbs free energy of ΔG = +24.9 kcal mol^{-1}. Similarly to **TS7** for Sb species, **TS7-Bi** is a concerted transition state in which both C–O bond formation and C–F bond cleavage occur synchronously. Eventually, **INT7-Bi** evolves to **INT8-Bi**, which can undergo subsequent ligand exchange with $PhOSiMe_3$ or S_NAr with another molecule of pentafluoropyridine. Both possibilities were therefore investigated computationally. As shown in Figure S44, **INT8-Bi** can undergo ligand metathesis with an additional molecule of $PhOSiMe_3$ through the formation of a silicate intermediate. This process begins with a silane-mediated fluoride abstraction, forming the fluorosilicate anion $[PhOSiMe_3F]^-$ and the corresponding cationic Bi(III) intermediate (**INT9-Bi**), as a contacted ion-pair. The connecting transition state **TS8-Bi** could be located, with an overall Gibbs activation energy for the fluoride abstraction step of 19.9 kcal mol^{-1} (**TS8**, ΔG = +10.8 kcal mol^{-1}). Following fluoride abstraction, **INT9-Bi** (ΔG = +8.8 kcal mol^{-1}) would rapidly evolve to the Bi(III)–OPh phenoxy species (**INT7-Bi,** ΔG = –16.1 kcal mol^{-1}) in a barrierless process (**TS10-Bi**), similarly to the pathway described in Figure 3. This transmetallation step closes the catalytic cycle and supports Bi(III)–$(OR)_2$ species as the active catalyst of this transformation.

The second possibility involves **INT8-Bi** engaging in a second S_NAr-type reaction with pentafluoropyridine through a higher-energy transition state (**TS10-Bi**, ΔG = +18.3 kcal mol^{-1}), with an overall activation barrier of 27.4 kcal mol^{-1}. Once again, the energy difference between both competing pathways ($\Delta\Delta G^{\ddagger}_{TS10-TS8}$ = 7.5 kcal mol^{-1}) further favours ligand metathesis over the second S_NAr process. Overall, these results suggest that **INT8-Bi** preferentially undergoes fluoride-assisted ligand exchange with $PhOSiMe_3$ to regenerate the active Bi(III)–OPh species **INT7-Bi**, rather than reacting with a second molecule of pentafluoropyridine.

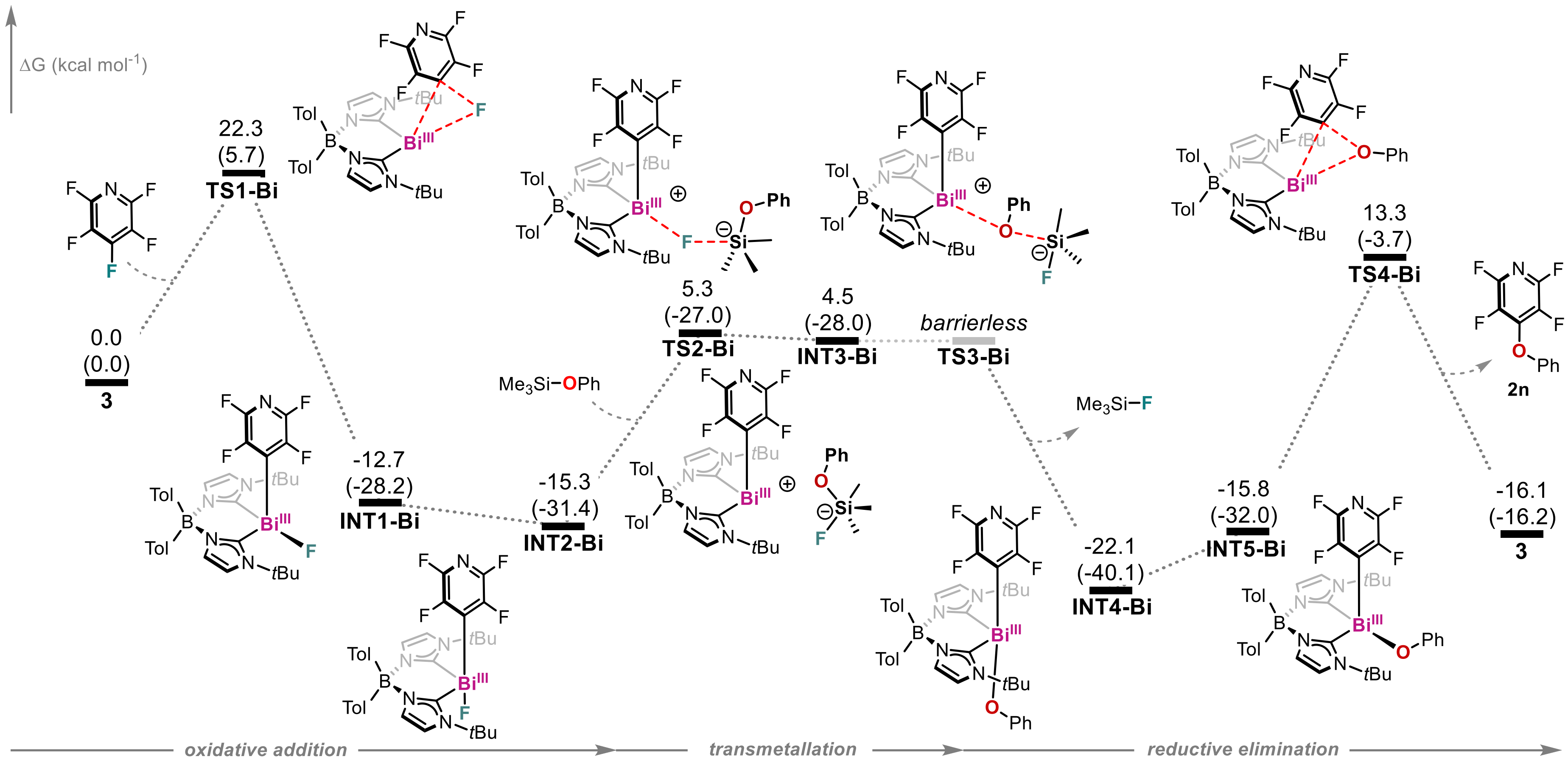

Figure S42. Gibbs energy (*Δ*G) for the defluoroalkoxylation of pentafluoropyridine with $PhOSiMe_3$ proceeding *via* Bi(I)/Bi(III) redox catalysis with bismuthinidene species **3**. Enthalpy (*Δ*H) in parentheses. Calculations were performed at the CPCM(benzene)-PBE0-D3BJ/def2-TZVPP//CPCM(benzene)-PBE0-D3BJ/def2-SVP level of theory.

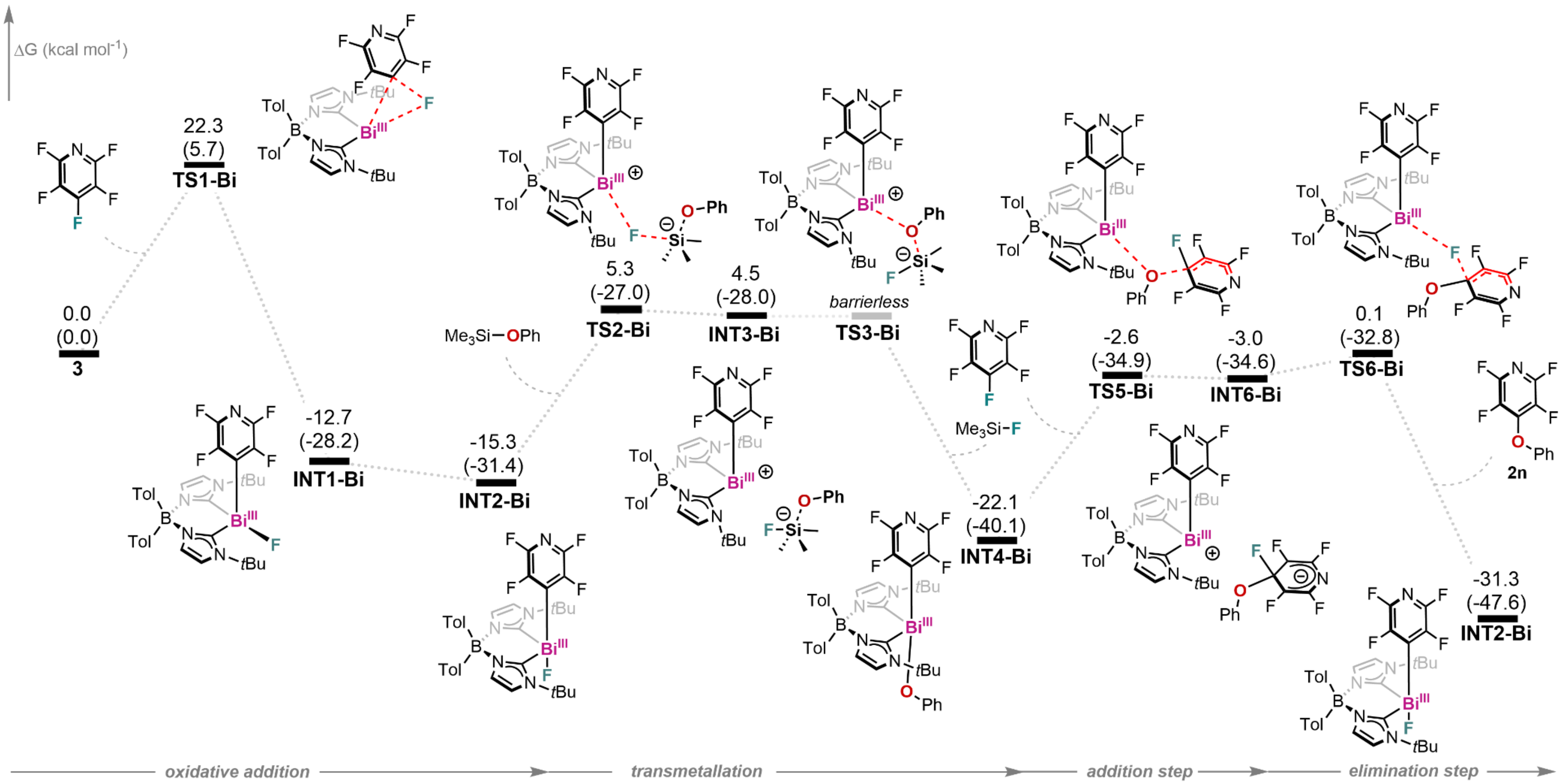

Figure S43. Gibbs energy (ΔG) for an S_NAr-type mechanism of the Bi(III)-fluoride-catalyzed defluoroalkoxylation of pentafluoropyridine with $PhOSiMe_3$. Enthalpy (ΔH) in parentheses. All calculations were performed at the CPCM(benzene)-PBE0-D3BJ/def2-TZVPP//CPCM(benzene)-PBE0-D3BJ/def2-SVP level of theory.

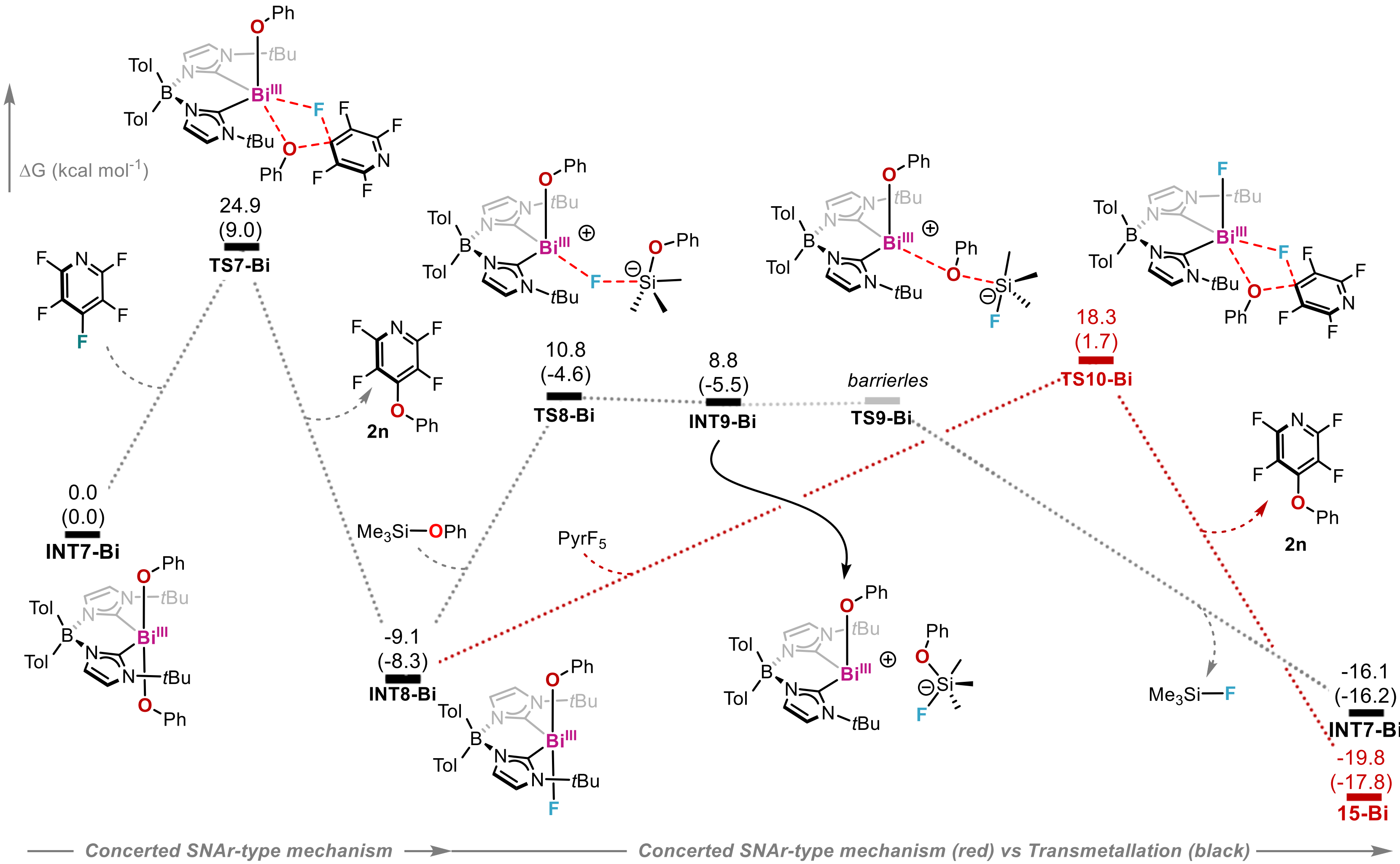

Figure S44. Gibbs energy (ΔG) for an S_NAr-type mechanism after ligand scrambling and transmetallation. Enthalpy (ΔH) in parentheses. All calculations were performed at the CPCM(benzene)-PBE0-D3BJ/def2-TZVPP//CPCM(benzene)-PBE0-D3BJ/def2-SVP level of theory.

6.12 Bi-catalyzed homodimerization of pentafluoropyridine

Based on our experimental results for Sb(I) (see Sections 4.4.1 and 4.4.2) and the comparable yield for the synthesis of **10a**, a similar redox Bi-catalyzed pathway involving a ligand scrambling and the Bi(I)/Bi(III) redox couple was evaluated both in benzene (Figure S45) and acetonitrile (Figure S46).

As previously stated, the reaction begins with oxidative addition of Bi(I) species **3** to the C4–F bond of the pyridine ring to afford the more thermodynamically favoured species **INT2-Bi** (ΔG = -15.3 kcal mol^{-1}), in which the fluorine occupies an axial site after isomerization. Subsequent ligand scrambling with another molecule of **INT2-Bi** would generate Bi(III) difluoride species **15-Bi** and Bi(III) bis(4-tetrafluoropyridyl) species **16-Bi** (ΔG = -13.7 kcal mol^{-1}). This process was found to be endergonic by +1.6 kcal mol^{-1}. At this stage, reduction of Bi(III) difluoride species **15-Bi** by Mashima's reagent **S6** would regenerate one molecule of bismuthinidene **3** while forming pyrazine and two molecules of TMSF in a highly exergonic process ($\Delta\Delta$G = -51.5 kcal mol^{-1}). On the other hand, isomerization of species **16-Bi** to **INT11-Bi** (ΔG = -54.3 kcal mol^{-1}) followed by a reductive elimination step would generate another molecule of bimuthinidene **3** and form dimerization product **10a**. Although this process would be thermodynamically favoured by -27.8 kcal mol^{-1}, the energetic barrier to overcome **TS12-Bi** (ΔG = -29.6 kcal mol^{-1}) from species **16-Bi** would make this process highly unfeasible in benzene ($\Delta\Delta$G = +35.6 kcal mol^{-1}).

When the same reaction profile was computed in acetonitrile, although comparable values were found for the initial oxidative addition process, differences in energy were observed for the ligand scrambling and reductive elimination stages. For instance, the ligand scrambling step from **INT2-Bi** to form species **15** and **16** becomes slightly more thermodynamically disfavoured ($\Delta\Delta$G = +3.1 kcal mol^{-1} vs +1.6 kcal mol^{-1} in benzene). In contrast, the energetic barrier for reductive elimination of product **10a** from species **16-Bi** decreased ($\Delta\Delta$G = +32.1 kcal mol^{-1} in acetonitrile vs +35.6 kcal mol^{-1} in benzene). Although this value is too high to be consistent with our experimental results, it follows the trend observed for Sb: lower activation barriers when a polar solvent (CH_3CN) is used. Additionally, this last step is slightly more exergonic ($\Delta\Delta$G = -28.6 kcal mol^{-1} acetonitrile vs -27.8 kcal mol^{-1} in benzene).

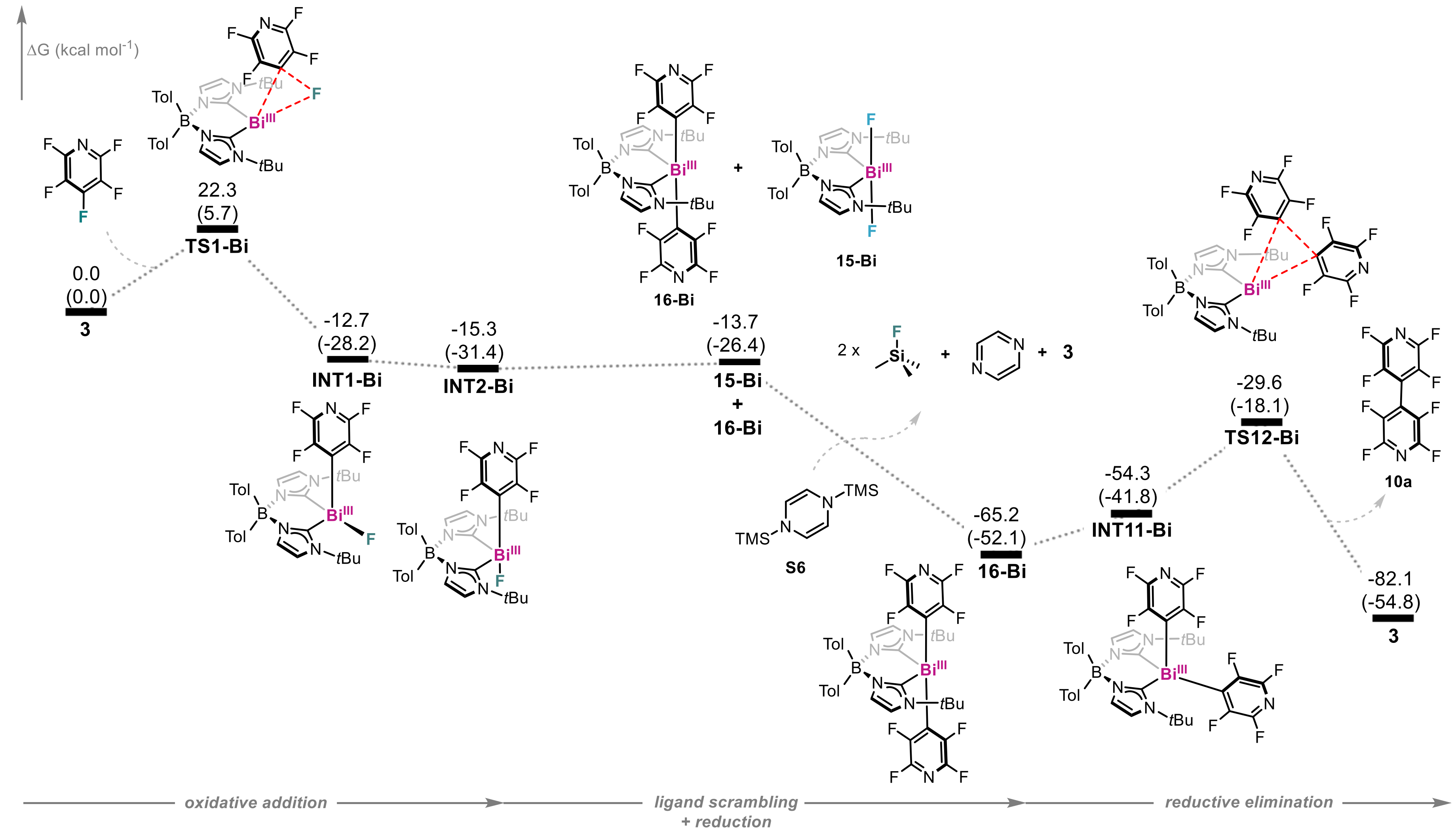

Figure S45. Gibbs energy (ΔG) and enthalpy (ΔH, parenthesis) for the bismuthinidene-catalyzed homodimerization of pentafluoropyridine proceeding *via* redox catalysis using species **S6** as reductant in benzene. Enthalpy energy (ΔH) in parentheses. Calculations were performed at the CPCM(benzene)-PBE0-D3(BJ)/def2-TZVPP//CPCM(benzene)-PBE0-D3(BJ)/def2-SVP level of theory.

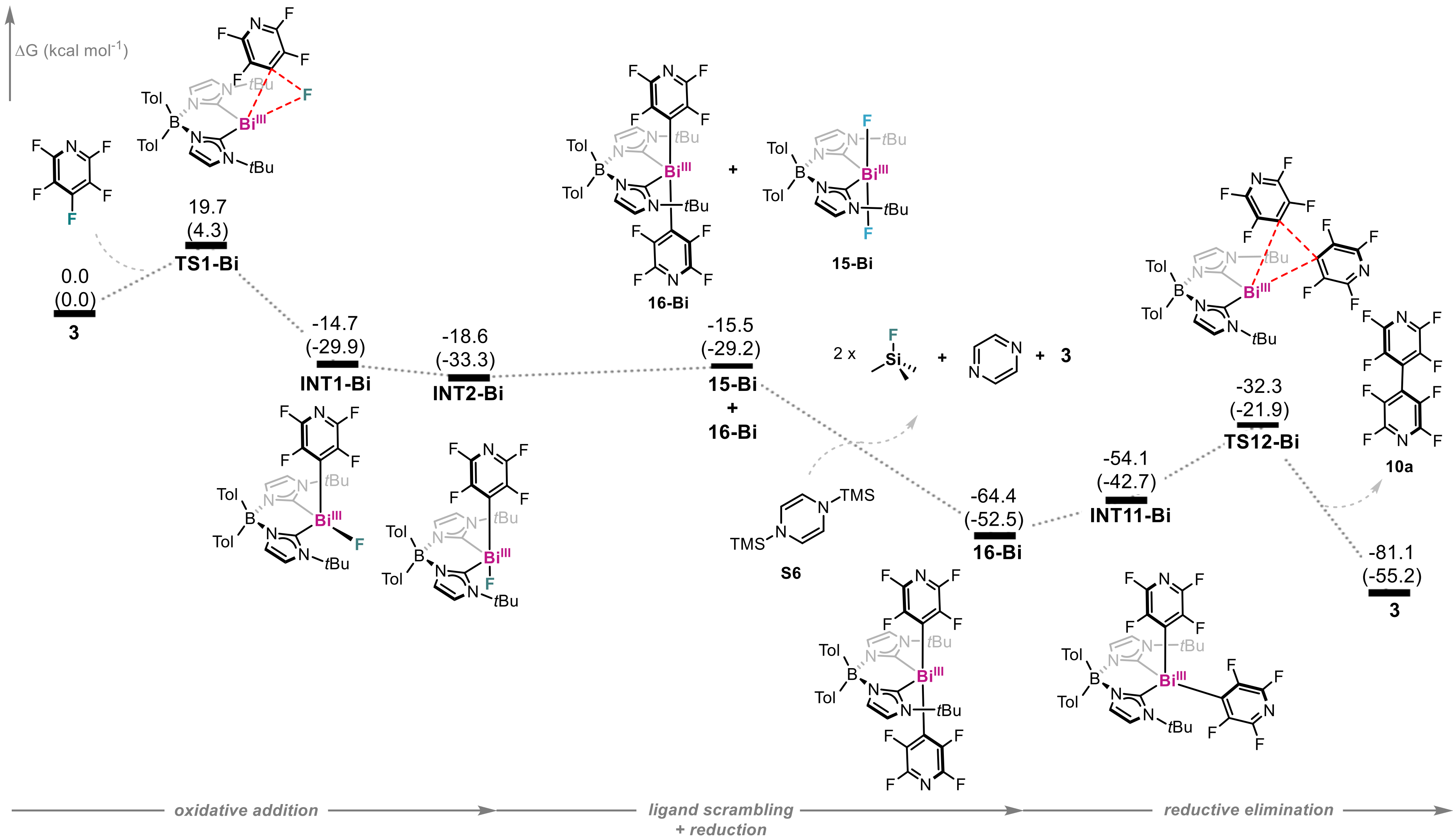

Figure S46. Gibbs energy (ΔG) and enthalpy (ΔH, parenthesis) for the bismuthinidene-catalyzed homodimerization of pentafluoropyridine proceeding *via* redox catalysis using species **S6** as reductant in acetonitrile. Enthalpy energy (ΔH) in parentheses. Calculations were performed at the CPCM(acetonitrile)-PBE0-D3(BJ)/def2-TZVPP//CPCM(acetonitrile)-PBE0-D3(BJ)/def2-SVP level of theory.

6.13 Mechanism A (Bismuth) – xyz coordinates (in Å) and Energies (in Hartree)

6.13.1 **Bismuthinidene 3**

Energy(PBE0-D3(BJ)/def2-TZVPP) = -1546.23836854948

6	1.075618000	-2.415056000	-1.054785000
7	0.445727000	-1.285342000	-0.588318000
6	-0.861180000	-1.555638000	-0.415795000
6	0.145325000	-3.396511000	-1.169571000
1	0.256504000	-4.424161000	-1.493604000
1	2.141738000	-2.437348000	-1.255646000
7	-1.060685000	-2.854185000	-0.769386000
6	-2.358577000	-3.576664000	-0.788346000
83	-2.348860000	0.000130000	0.310523000
7	0.445887000	1.285830000	-0.587681000
6	-0.861046000	1.556083000	-0.415246000
6	0.145628000	3.397294000	-1.167961000
7	-1.060462000	2.854807000	-0.768266000
1	0.256896000	4.425096000	-1.491487000
1	2.142085000	2.438228000	-1.253839000
6	1.075905000	2.415811000	-1.053339000
6	-2.358325000	3.577347000	-0.787175000
5	1.214000000	0.000067000	-0.091511000
6	-3.035830000	3.436001000	0.575028000
1	-3.347177000	2.404398000	0.792945000
1	-2.365231000	3.769289000	1.380348000
1	-3.945841000	4.052512000	0.597024000
6	-2.111797000	5.062868000	-1.041323000
1	-1.463448000	5.506453000	-0.271177000
1	-1.671431000	5.246231000	-2.031796000
1	-3.079025000	5.582879000	-1.009614000
6	-3.223526000	3.008264000	-1.911028000
1	-2.731532000	3.155679000	-2.884040000
1	-3.396435000	1.930649000	-1.767728000
1	-4.197191000	3.519343000	-1.929681000
6	-3.223702000	-3.007605000	-1.912262000
1	-3.396550000	-1.929970000	-1.769040000
1	-2.731687000	-3.155108000	-2.885251000
1	-4.197398000	-3.518627000	-1.930922000
6	-3.036128000	-3.435099000	0.573820000
1	-2.365575000	-3.768306000	1.379213000
1	-3.347416000	-2.403438000	0.791586000
1	-3.946175000	-4.051552000	0.595860000
6	-2.112141000	-5.062238000	-1.042298000
1	-1.671818000	-5.245782000	-2.032760000
1	-1.463787000	-5.505738000	-0.272108000
1	-3.079395000	-5.582194000	-1.010488000

6	1.252590000	-0.000342000	1.527194000
6	1.314173000	-1.191956000	2.262646000
6	1.313940000	1.190864000	2.263299000
6	1.416802000	-1.197204000	3.652727000
6	1.416559000	1.195361000	3.653395000
6	1.464022000	-0.001111000	4.378148000
1	1.275013000	-2.151548000	1.736806000
1	1.274586000	2.150737000	1.737988000
1	1.458942000	-2.150551000	4.189129000
1	1.458498000	2.148416000	4.190327000
6	2.693125000	0.000067000	-0.758939000
6	2.855204000	-0.000359000	-2.157094000
6	3.865497000	0.000360000	0.003591000
6	4.111029000	-0.000399000	-2.754883000
6	5.131330000	0.000339000	-0.588875000
6	5.279337000	-0.000025000	-1.977701000
1	1.967824000	-0.000665000	-2.799335000
1	3.790024000	0.000633000	1.094615000
1	4.193079000	-0.000731000	-3.846521000
1	6.024285000	0.000604000	0.043882000
6	1.532517000	-0.001554000	5.878443000
1	2.054201000	-0.892507000	6.258468000
1	2.051948000	0.890444000	6.259086000
1	0.521984000	-0.003008000	6.321728000
6	6.633817000	-0.000005000	-2.625917000
1	6.769334000	-0.884827000	-3.268714000
1	6.769426000	0.885025000	-3.268417000

6.13.2 **TS1-Bi**

Energy(PBE0-D3(BJ)/def2-TZVPP) = -2290.26567614582

6	2.152143000	-0.293279000	2.407523000
7	1.393076000	-0.439542000	1.274195000
6	0.297756000	-1.144260000	1.587698000
6	1.516293000	-0.932864000	3.424566000
1	1.805686000	-1.041802000	4.462558000
1	3.093108000	0.247197000	2.404477000
7	0.364392000	-1.472292000	2.901232000
6	-0.600352000	-2.315411000	3.665999000
83	-1.356059000	-1.622431000	0.086583000
7	1.386522000	-0.632323000	-1.308315000
6	0.253487000	-1.314522000	-1.522635000
6	1.489285000	-1.438886000	-3.360455000
7	0.306116000	-1.830445000	-2.777662000
1	1.772934000	-1.708725000	-4.370393000
1	3.115480000	-0.190079000	-2.510885000
6	2.155372000	-0.690686000	-2.441738000
6	-0.662894000	-2.773657000	-3.406346000
5	1.780609000	0.288573000	-0.084426000
6	-2.078609000	-2.220976000	-3.284317000
1	-2.463871000	-2.245520000	-2.256186000
1	-2.137768000	-1.185635000	-3.644959000
1	-2.761342000	-2.841511000	-3.881431000
6	-0.332631000	-2.919974000	-4.889820000
1	-0.373384000	-1.951213000	-5.409049000
1	0.652538000	-3.378295000	-5.055634000
1	-1.082846000	-3.579919000	-5.345843000
6	-0.526704000	-4.130978000	-2.716437000
1	0.497954000	-4.517419000	-2.820226000
1	-0.763616000	-4.063251000	-1.643977000
1	-1.220668000	-4.852093000	-3.171923000
6	-0.629203000	-3.712378000	3.046373000
1	-0.998332000	-3.700950000	2.010629000
1	0.376919000	-4.157312000	3.049095000
1	-1.299619000	-4.359189000	3.630009000
6	-1.974778000	-1.653111000	3.658642000
1	-1.925367000	-0.656436000	4.117774000
1	-2.402332000	-1.520246000	2.656328000
1	-2.677713000	-2.273494000	4.232734000
6	-0.129045000	-2.433673000	5.113208000
1	0.852022000	-2.924419000	5.189803000
1	-0.084962000	-1.453513000	5.609499000
1	-0.854582000	-3.051206000	5.659775000
6	1.007573000	1.695705000	-0.199347000
6	1.079611000	2.624646000	0.848924000
6	0.343179000	2.110942000	-1.360809000
6	0.536439000	3.901023000	0.738498000
6	-0.209915000	3.384626000	-1.474981000
6	-0.121038000	4.307010000	-0.428155000
1	1.581247000	2.350416000	1.782150000

1	0.251828000	1.428628000	-2.211604000
1	0.607367000	4.595497000	1.580438000
1	-0.738968000	3.664611000	-2.390736000
6	3.397725000	0.435146000	-0.109402000
6	4.219274000	-0.697233000	0.041291000
6	4.047849000	1.655281000	-0.319587000
6	5.606336000	-0.614396000	-0.009014000
6	5.441352000	1.746801000	-0.372352000
6	6.247213000	0.616146000	-0.218819000
1	3.757233000	-1.677051000	0.204658000
1	3.450906000	2.562557000	-0.445939000
1	6.209540000	-1.519077000	0.116802000
1	5.912053000	2.721004000	-0.535856000
6	-0.781592000	5.648404000	-0.525888000
1	-0.389877000	6.350213000	0.224369000
1	-0.651369000	6.094264000	-1.523630000
1	-1.864840000	5.534405000	-0.354562000
6	7.744384000	0.701775000	-0.289988000
1	8.088743000	1.745482000	-0.275473000
1	8.217191000	0.172071000	0.551573000
1	8.124376000	0.237072000	-1.214926000
6	-2.661229000	0.426335000	0.332395000
6	-2.255385000	1.479231000	1.202728000
6	-3.203513000	0.931116000	-0.883638000
6	-2.580905000	2.787276000	0.926604000
6	-3.435307000	2.273704000	-1.064142000
7	-3.178413000	3.205310000	-0.170939000
9	-1.597200000	1.175279000	2.339139000
9	-3.491082000	0.050997000	-1.869046000
9	-2.280189000	3.719661000	1.831002000
9	-3.955003000	2.680765000	-2.223882000
9	-3.610431000	-0.532457000	1.023259000

6.13.3 **INT1-Bi**

Energy(PBE0-D3(BJ)/def2-TZVPP) = -2290.32090254206

6	1.819136000	0.261458000	2.670018000
7	1.192487000	-0.138634000	1.513200000
6	0.103784000	-0.870483000	1.818127000
6	1.116416000	-0.243987000	3.713182000
1	1.289509000	-0.157262000	4.780895000
1	2.717500000	0.869967000	2.662535000
7	0.062858000	-0.952389000	3.175788000
6	-0.905644000	-1.637563000	4.067794000
83	-1.417263000	-1.748422000	0.065666000
7	1.462942000	-0.688018000	-1.018807000
6	0.476047000	-1.571061000	-1.249898000
6	1.941307000	-1.731805000	-2.904797000
7	0.776163000	-2.245981000	-2.389802000
1	2.390358000	-2.090584000	-3.822873000
1	3.255962000	-0.150209000	-2.084412000
6	2.365948000	-0.769235000	-2.048252000
6	0.033348000	-3.341510000	-3.086763000
5	1.682242000	0.405313000	0.120745000
6	-0.920352000	-2.701226000	-4.094673000
1	-1.675675000	-2.111515000	-3.555909000
1	-0.368452000	-2.062650000	-4.801007000
1	-1.430418000	-3.490026000	-4.667873000
6	1.048502000	-4.238504000	-3.799077000
1	1.552162000	-3.734171000	-4.635329000
1	1.809881000	-4.620717000	-3.102238000
1	0.509397000	-5.096926000	-4.222274000
6	-0.739138000	-4.193479000	-2.082842000
1	-0.113173000	-4.476001000	-1.222074000
1	-1.653537000	-3.670849000	-1.771063000
1	-1.058033000	-5.119047000	-2.582225000
6	-1.927621000	-2.437726000	3.277258000
1	-2.564143000	-1.789533000	2.660032000
1	-1.449453000	-3.204748000	2.651396000
1	-2.587378000	-2.954373000	3.988324000
6	-1.626418000	-0.571152000	4.896225000
1	-0.924710000	-0.003963000	5.524548000
1	-2.151615000	0.133207000	4.236555000
1	-2.362598000	-1.049312000	5.558536000
6	-0.127760000	-2.598051000	4.971504000
1	0.424170000	-3.334739000	4.368647000
1	0.588914000	-2.070754000	5.616507000
1	-0.829397000	-3.137816000	5.623478000
6	0.903873000	1.767752000	-0.258679000
6	0.246638000	2.545873000	0.703866000
6	0.937120000	2.283470000	-1.561829000
6	-0.360312000	3.757923000	0.382810000
6	0.331624000	3.493571000	-1.892558000
6	-0.337576000	4.252898000	-0.926076000
1	0.189905000	2.193131000	1.738115000

1	1.435751000	1.719182000	-2.355211000
1	-0.878674000	4.326595000	1.160983000
1	0.363566000	3.851509000	-2.925782000
6	3.291060000	0.628037000	0.243792000
6	4.133308000	-0.451333000	0.569243000
6	3.908848000	1.868146000	0.058794000
6	5.509200000	-0.301315000	0.695510000
6	5.292362000	2.027932000	0.184299000
6	6.118790000	0.948449000	0.502822000
1	3.695188000	-1.442667000	0.728520000
1	3.295171000	2.738207000	-0.190498000
1	6.129285000	-1.167061000	0.948917000
1	5.737807000	3.015715000	0.031614000
6	-1.063607000	5.514076000	-1.287216000
1	-0.996183000	6.264551000	-0.485535000
1	-0.670934000	5.956770000	-2.213958000
1	-2.134731000	5.303637000	-1.447210000
6	7.605781000	1.106894000	0.636012000
1	8.140896000	0.482579000	-0.097787000
1	7.914587000	2.150282000	0.480369000
1	7.953466000	0.795257000	1.634097000
6	-2.111889000	0.413617000	-0.223092000
6	-2.836380000	1.032610000	0.778934000
6	-2.070528000	1.064545000	-1.446071000
6	-3.466657000	2.249332000	0.538608000
6	-2.735547000	2.281251000	-1.588240000
7	-3.416700000	2.845865000	-0.623050000
9	-2.951582000	0.467976000	1.989816000
9	-1.405656000	0.574776000	-2.482560000
9	-4.150445000	2.837689000	1.509063000
9	-2.694371000	2.909748000	-2.752705000
9	-2.675715000	-1.916047000	-1.746838000

6.13.4 **INT2-Bi**

Energy(PBE0-D3(BJ)/def2-TZVPP) = -2290.32658004862

6	-1.952279000	-0.367045000	-2.386754000
7	-1.240912000	-0.395747000	-1.211162000
6	-0.321366000	-1.368298000	-1.296849000
6	-1.468940000	-1.346728000	-3.190676000
1	-1.773158000	-1.647056000	-4.187812000
1	-2.751336000	0.346810000	-2.556975000
7	-0.457920000	-1.973794000	-2.497467000
6	0.205016000	-3.184847000	-3.058710000
83	1.092332000	-1.892436000	0.419761000
7	-1.308603000	0.046699000	1.351299000
6	-0.364863000	-0.791967000	1.793935000
6	-1.567268000	-0.151435000	3.537560000
7	-0.512004000	-0.933928000	3.130881000
1	-1.894175000	-0.081797000	4.567905000
1	-2.890039000	1.145604000	2.316685000
6	-2.059661000	0.455581000	2.425498000
6	0.261436000	-1.845378000	4.023173000
5	-1.525665000	0.684924000	-0.085527000
6	1.759174000	-1.636144000	3.794840000
1	2.111731000	-2.063887000	2.845113000
1	2.018579000	-0.569036000	3.814440000
1	2.320015000	-2.154102000	4.585130000
6	-0.044188000	-1.498909000	5.478335000
1	0.219125000	-0.457076000	5.714597000
1	-1.099278000	-1.672426000	5.732491000
1	0.558050000	-2.155768000	6.120632000
6	-0.171135000	-3.283317000	3.733326000
1	-1.218275000	-3.428262000	4.040335000
1	-0.106787000	-3.537651000	2.662691000
1	0.454543000	-3.980109000	4.310670000
6	-0.864421000	-4.273938000	-3.165410000
1	-1.273534000	-4.483085000	-2.165733000
1	-1.686791000	-3.978903000	-3.832687000
1	-0.416018000	-5.195965000	-3.562426000
6	1.316722000	-3.691806000	-2.154615000
1	2.111892000	-2.941791000	-2.025699000
1	0.903198000	-4.023704000	-1.189794000
1	1.777753000	-4.565767000	-2.635544000
6	0.787755000	-2.814346000	-4.422083000
1	0.012856000	-2.506106000	-5.138034000
1	1.513749000	-1.994917000	-4.316040000
1	1.304576000	-3.687112000	-4.845748000
6	-0.552093000	1.958321000	-0.269518000
6	0.048920000	2.270367000	-1.496494000
6	-0.327227000	2.856663000	0.782328000
6	0.850201000	3.397643000	-1.658322000
6	0.474204000	3.986129000	0.630097000
6	1.088659000	4.274249000	-0.593221000
1	-0.089365000	1.604158000	-2.353460000

1	-0.772332000	2.665869000	1.763121000
1	1.319851000	3.592827000	-2.627290000
1	0.642030000	4.650552000	1.482852000
6	-3.099110000	1.073093000	-0.193285000
6	-4.089638000	0.086554000	-0.033352000
6	-3.543974000	2.372086000	-0.455572000
6	-5.444297000	0.383023000	-0.128201000
6	-4.904633000	2.677764000	-0.552985000
6	-5.879932000	1.691334000	-0.391029000
1	-3.789510000	-0.947029000	0.170896000
1	-2.808930000	3.170942000	-0.588620000
1	-6.184406000	-0.412833000	0.002276000
1	-5.213507000	3.706862000	-0.760479000
6	2.020547000	5.438573000	-0.748054000
1	1.886704000	5.938790000	-1.719104000
1	1.877001000	6.181992000	0.049139000
1	3.068322000	5.096451000	-0.700865000
6	-7.344130000	2.007687000	-0.490478000
1	-7.864126000	1.790789000	0.456717000
1	-7.511490000	3.066710000	-0.732629000
1	-7.832047000	1.399365000	-1.268842000
6	2.212691000	0.231534000	-0.063799000
6	2.502597000	1.237430000	0.838061000
6	2.834101000	0.354041000	-1.292622000
6	3.348630000	2.285066000	0.479883000
6	3.672730000	1.427989000	-1.570855000
7	3.923371000	2.367613000	-0.693862000
9	2.635395000	-0.570669000	-2.254130000
9	1.979954000	1.240126000	2.072430000
9	3.597524000	3.255355000	1.350218000
9	4.249138000	1.530878000	-2.762129000
9	-0.323855000	-3.620874000	0.569522000

6.13.5 **TS2-Bi**

Energy(PBE0-D3(BJ)/def2-TZVPP) = -3006.075083456849

6	-1.606011000	-0.027772000	-1.908212000
7	-0.645822000	-0.395340000	-0.997650000
6	-0.187964000	-1.600132000	-1.342253000
6	-1.732807000	-1.034429000	-2.813324000
1	-2.384961000	-1.114756000	-3.674478000
1	-2.128742000	0.920810000	-1.840839000
7	-0.843290000	-2.018315000	-2.446298000
6	-0.711419000	-3.342539000	-3.122808000
83	1.274563000	-2.783318000	-0.073514000
7	0.270021000	-0.158886000	1.440323000
6	0.954221000	-1.295189000	1.630898000
6	0.674539000	-0.333544000	3.602801000
7	1.196078000	-1.430426000	2.955751000
1	0.752807000	-0.192209000	4.675108000
1	-0.419644000	1.398907000	2.756323000
6	0.098715000	0.451186000	2.657376000
6	1.944590000	-2.472481000	3.718985000
5	-0.118642000	0.612204000	0.108393000
6	3.258368000	-1.845157000	4.187004000
1	3.862285000	-1.525203000	3.326940000
1	3.076863000	-0.974122000	4.833195000
1	3.834092000	-2.583286000	4.763784000
6	1.088241000	-2.899646000	4.912320000
1	0.914832000	-2.080293000	5.623258000
1	0.115188000	-3.283471000	4.576058000
1	1.607185000	-3.702368000	5.454382000
6	2.224689000	-3.700912000	2.868871000
1	1.305758000	-4.155364000	2.468896000
1	2.938369000	-3.490260000	2.060383000
1	2.701560000	-4.457765000	3.506617000
6	-1.342443000	-4.408144000	-2.231102000
1	-0.871154000	-4.470063000	-1.240179000
1	-2.408406000	-4.191123000	-2.073616000
1	-1.254184000	-5.393512000	-2.711011000
6	0.760308000	-3.639751000	-3.404468000
1	1.234883000	-2.816958000	-3.953969000
1	1.344287000	-3.833171000	-2.492040000
1	0.829666000	-4.558606000	-4.002842000
6	-1.447352000	-3.293998000	-4.459370000
1	-2.528807000	-3.146715000	-4.330029000
1	-1.049894000	-2.505977000	-5.115945000
1	-1.307124000	-4.259556000	-4.963689000
6	1.191672000	1.376094000	-0.449854000
6	1.221120000	1.889326000	-1.754277000
6	2.330931000	1.602367000	0.335087000
6	2.330384000	2.565420000	-2.256748000
6	3.444964000	2.277474000	-0.158927000
6	3.471229000	2.764590000	-1.470714000
1	0.359761000	1.743282000	-2.412653000

1	2.365136000	1.231794000	1.364104000
1	2.320342000	2.933860000	-3.286638000
1	4.320223000	2.419014000	0.482578000
6	-1.355907000	1.590142000	0.473819000
6	-2.559787000	1.045462000	0.958744000
6	-1.295890000	2.980798000	0.351457000
6	-3.641344000	1.848695000	1.300314000
6	-2.380798000	3.794063000	0.692302000
6	-3.572102000	3.244999000	1.172100000
1	-2.648805000	-0.040364000	1.076300000
1	-0.377588000	3.446248000	-0.017992000
1	-4.560407000	1.388859000	1.676890000
1	-2.297656000	4.879839000	0.584505000
6	4.699930000	3.420472000	-2.025936000
1	4.474039000	4.004893000	-2.929153000
1	5.171547000	4.087176000	-1.288451000
1	5.448659000	2.657853000	-2.299824000
6	-4.744835000	4.107980000	1.537839000
1	-5.065535000	3.926688000	2.576016000
1	-4.505613000	5.175976000	1.436913000
1	-5.613025000	3.893680000	0.893593000
6	3.044081000	-1.442372000	-0.919453000
6	3.094591000	-0.797883000	-2.145468000
6	4.206902000	-1.371782000	-0.171716000
6	4.256667000	-0.134252000	-2.538803000
6	5.321948000	-0.685150000	-0.640147000
7	5.338172000	-0.088187000	-1.803963000
9	2.056866000	-0.787629000	-2.982304000
9	4.281492000	-1.968803000	1.029265000
9	4.287815000	0.479294000	-3.710908000
9	6.417380000	-0.626379000	0.100730000
14	-1.752993000	-4.406681000	1.812240000
9	-1.134654000	-3.014884000	0.871649000
6	-2.500891000	-3.058728000	2.976922000
1	-3.074311000	-2.313227000	2.402120000
1	-3.163526000	-3.497343000	3.740879000
1	-1.710749000	-2.501625000	3.510476000
6	-1.423505000	-5.678780000	3.214195000
1	-0.592005000	-5.332246000	3.852364000
1	-2.312760000	-5.748598000	3.861435000
1	-1.162563000	-6.689986000	2.869204000
6	-3.216170000	-4.959543000	0.756033000
1	-3.978650000	-5.423900000	1.402266000
1	-3.674667000	-4.079485000	0.277867000
1	-2.961676000	-5.676306000	-0.036727000
8	-0.221281000	-5.098025000	0.917459000
6	0.206317000	-6.324811000	0.656755000
6	1.594261000	-6.568907000	0.581428000
6	-0.659397000	-7.407188000	0.397148000
6	2.089235000	-7.827795000	0.254149000
6	-0.153338000	-8.664771000	0.076825000
6	1.221360000	-8.890352000	-0.001411000
1	2.284817000	-5.748538000	0.789265000

1	-1.737070000	-7.253188000	0.452160000
1	3.171046000	-7.980433000	0.203371000
1	-0.851592000	-9.484029000	-0.116810000
1	1.610194000	-9.879028000	-0.254909000

6.13.6 **INT3-Bi**

Energy(PBE0-D3(BJ)/def2-TZVPP) = -3006.076755913142

6	-1.561030000	-0.048023000	-1.838504000
7	-0.625247000	-0.420343000	-0.905908000
6	-0.174949000	-1.634897000	-1.232339000
6	-1.674283000	-1.055225000	-2.744672000
1	-2.307199000	-1.131716000	-3.620335000
1	-2.077977000	0.904669000	-1.784967000
7	-0.803559000	-2.046088000	-2.355959000
6	-0.654122000	-3.358019000	-3.056084000
83	1.288623000	-2.794679000	0.064275000
7	0.335412000	-0.099401000	1.506844000
6	1.001778000	-1.240988000	1.720242000
6	0.803336000	-0.196978000	3.660496000
7	1.278303000	-1.330943000	3.041644000
1	0.915188000	-0.018125000	4.724124000
1	-0.281693000	1.524422000	2.778189000
6	0.214648000	0.562703000	2.702885000
6	1.978220000	-2.386904000	3.829161000
5	-0.089384000	0.624547000	0.165261000
6	3.342366000	-1.833432000	4.243894000
1	3.951305000	-1.597822000	3.360940000
1	3.233716000	-0.922062000	4.849657000
1	3.875343000	-2.581948000	4.847834000
6	1.123947000	-2.693308000	5.062424000
1	1.102092000	-1.854512000	5.771333000
1	0.090840000	-2.931736000	4.773827000
1	1.547865000	-3.559673000	5.588445000
6	2.157190000	-3.664683000	3.022427000
1	1.205519000	-4.067149000	2.644311000
1	2.878484000	-3.533312000	2.203707000
1	2.587428000	-4.429883000	3.682956000
6	-1.375851000	-4.424337000	-2.240936000
1	-0.979855000	-4.511607000	-1.218636000
1	-2.447772000	-4.191052000	-2.169949000
1	-1.266203000	-5.403718000	-2.728812000
6	0.825181000	-3.693503000	-3.235244000
1	1.368147000	-2.868548000	-3.712668000
1	1.327259000	-3.950838000	-2.290665000
1	0.910245000	-4.590357000	-3.863991000
6	-1.282079000	-3.253451000	-4.444278000
1	-2.367890000	-3.091489000	-4.396026000
1	-0.821299000	-2.452502000	-5.041186000
1	-1.118375000	-4.205899000	-4.966037000
6	1.202653000	1.388867000	-0.437140000
6	1.197651000	1.896405000	-1.744056000
6	2.365048000	1.615982000	0.313762000
6	2.293741000	2.568732000	-2.280089000
6	3.465127000	2.288684000	-0.213098000
6	3.455515000	2.771198000	-1.526757000
1	0.318780000	1.749186000	-2.378395000

1	2.429274000	1.249023000	1.342405000
1	2.255191000	2.932468000	-3.310994000
1	4.357716000	2.431722000	0.403604000
6	-1.337760000	1.589575000	0.526577000
6	-2.515806000	1.031656000	1.057611000
6	-1.316781000	2.975845000	0.353085000
6	-3.611083000	1.818433000	1.393305000
6	-2.415974000	3.772479000	0.687827000
6	-3.582090000	3.210336000	1.212463000
1	-2.572930000	-0.051073000	1.216839000
1	-0.419011000	3.451121000	-0.052711000
1	-4.509229000	1.348770000	1.806472000
1	-2.364310000	4.855402000	0.539393000
6	4.668353000	3.427407000	-2.115760000
1	4.422387000	3.986935000	-3.029370000
1	5.141740000	4.117843000	-1.401514000
1	5.422959000	2.668146000	-2.381950000
6	-4.770778000	4.054693000	1.569992000
1	-5.076305000	3.891874000	2.615699000
1	-4.558321000	5.125150000	1.439534000
1	-5.640294000	3.803910000	0.940779000
6	3.043277000	-1.478971000	-0.845508000
6	3.087314000	-0.825316000	-2.066960000
6	4.212549000	-1.424673000	-0.107085000
6	4.252790000	-0.170422000	-2.465366000
6	5.327749000	-0.738813000	-0.576322000
7	5.338426000	-0.134105000	-1.736199000
9	2.044268000	-0.800084000	-2.896546000
9	4.289494000	-2.040131000	1.084815000
9	4.283111000	0.442699000	-3.637490000
9	6.428094000	-0.690587000	0.157366000
14	-1.889630000	-4.409959000	1.833352000
9	-1.087308000	-2.987721000	1.263441000
6	-3.144265000	-3.265304000	2.763467000
1	-3.659067000	-2.577841000	2.070009000
1	-3.919544000	-3.851359000	3.286738000
1	-2.636607000	-2.641596000	3.520374000
6	-1.286154000	-5.353678000	3.365537000
1	-0.818800000	-4.639988000	4.061623000
1	-2.164562000	-5.763536000	3.891445000
1	-0.572462000	-6.168228000	3.183369000
6	-3.118811000	-5.203834000	0.636625000
1	-3.923565000	-5.674944000	1.225325000
1	-3.591141000	-4.398402000	0.050478000
1	-2.711441000	-5.948509000	-0.059289000
8	-0.315005000	-5.128430000	0.817285000
6	0.025097000	-6.369862000	0.540920000
6	1.221143000	-6.620294000	-0.174656000
6	-0.743252000	-7.500651000	0.908053000
6	1.620019000	-7.910482000	-0.505514000
6	-0.332786000	-8.789260000	0.572291000
6	0.847065000	-9.013548000	-0.136959000
1	1.842494000	-5.770032000	-0.466309000

1	-1.671566000	-7.363935000	1.462600000
1	2.552060000	-8.055786000	-1.059620000
1	-0.955086000	-9.636673000	0.874630000
1	1.159680000	-10.027475000	-0.396110000

6.13.7 **INT4-Bi**

Energy(PBE0-D3(BJ)/def2-TZVPP) = -2497.1674359868

6	-1.724885000	-0.921977000	-2.181268000
7	-0.981806000	-0.735438000	-1.043623000
6	0.106989000	-1.518135000	-1.115207000
6	-1.084898000	-1.837185000	-2.951826000
1	-1.353469000	-2.248516000	-3.918776000
1	-2.654575000	-0.390475000	-2.354266000
7	0.052520000	-2.209787000	-2.276115000
6	0.982431000	-3.212290000	-2.873095000
83	1.607967000	-1.685403000	0.609902000
7	-1.071527000	-0.113799000	1.477927000
6	0.011929000	-0.740854000	1.953876000
6	-1.245830000	-0.163192000	3.679457000
7	-0.079509000	-0.786759000	3.303341000
1	-1.558277000	-0.060481000	4.711328000
1	-2.795165000	0.784187000	2.405141000
6	-1.858489000	0.253635000	2.540413000
6	0.856180000	-1.452464000	4.258241000
5	-1.402354000	0.377425000	0.005121000
6	2.295958000	-1.062150000	3.926477000
1	2.668991000	-1.545726000	3.011763000
1	2.398996000	0.025618000	3.818870000
1	2.955471000	-1.405256000	4.735472000
6	0.552204000	-0.965042000	5.673539000
1	0.643672000	0.128110000	5.757632000
1	-0.446718000	-1.272433000	6.013357000
1	1.281693000	-1.420920000	6.356474000
6	0.642268000	-2.962630000	4.174154000
1	-0.385136000	-3.217870000	4.475235000
1	0.797308000	-3.348838000	3.154347000
1	1.337745000	-3.470214000	4.858816000
6	0.176740000	-4.477749000	-3.169861000
1	-0.278443000	-4.871282000	-2.249646000
1	-0.616605000	-4.299678000	-3.909018000
1	0.848244000	-5.244213000	-3.582436000
6	2.118622000	-3.562202000	-1.925421000
1	2.774422000	-2.697498000	-1.745739000
1	1.748500000	-3.984921000	-0.978724000
1	2.740020000	-4.329036000	-2.407839000
6	1.555220000	-2.601417000	-4.152927000
1	0.765997000	-2.372663000	-4.883494000
1	2.102709000	-1.675584000	-3.925997000
1	2.250412000	-3.314207000	-4.619110000
6	-0.606150000	1.746697000	-0.300615000
6	-0.025325000	2.017536000	-1.546474000
6	-0.542151000	2.769210000	0.655711000
6	0.605033000	3.229769000	-1.816701000
6	0.088474000	3.983514000	0.395441000
6	0.682999000	4.235498000	-0.846217000
1	-0.046178000	1.255428000	-2.331601000

1	-0.982847000	2.614429000	1.644899000
1	1.062794000	3.394947000	-2.796837000
1	0.134430000	4.749074000	1.175482000
6	-3.013250000	0.557535000	-0.100743000
6	-3.886918000	-0.496085000	0.226678000
6	-3.606042000	1.741343000	-0.551817000
6	-5.266791000	-0.373346000	0.110760000
6	-4.992307000	1.872057000	-0.672992000
6	-5.849283000	0.819294000	-0.344528000
1	-3.478144000	-1.444659000	0.587185000
1	-2.968812000	2.588707000	-0.819053000
1	-5.909248000	-1.218328000	0.377420000
1	-5.416178000	2.815143000	-1.031221000
6	1.429219000	5.508230000	-1.113388000
1	1.246237000	5.877524000	-2.133665000
1	1.150083000	6.298309000	-0.401756000
1	2.515688000	5.341541000	-1.018473000
6	-7.339837000	0.949630000	-0.465028000
1	-7.829360000	0.844465000	0.516819000
1	-7.626229000	1.925490000	-0.881883000
1	-7.757816000	0.164956000	-1.115578000
6	2.422420000	0.531297000	0.043139000
6	2.505538000	1.633732000	0.873785000
6	3.075120000	0.663592000	-1.168817000
6	3.189371000	2.775973000	0.460610000
6	3.743199000	1.836356000	-1.502930000
7	3.797650000	2.865182000	-0.695137000
9	3.072810000	-0.350453000	-2.056414000
9	1.935075000	1.645125000	2.084413000
9	3.238638000	3.833404000	1.258816000
9	4.351624000	1.942488000	-2.676469000
8	0.604709000	-3.806587000	1.049567000
6	-0.455146000	-4.413417000	0.577661000
6	-1.686015000	-3.748904000	0.360373000
6	-0.406710000	-5.789942000	0.243248000
6	-2.779395000	-4.406246000	-0.195617000
6	-1.509454000	-6.442708000	-0.299257000
6	-2.704902000	-5.758535000	-0.537381000
1	-1.758525000	-2.695391000	0.634343000
1	0.530615000	-6.324808000	0.419093000
1	-3.707119000	-3.850062000	-0.362869000
1	-1.432295000	-7.505174000	-0.549505000
1	-3.564471000	-6.272390000	-0.973465000

6.13.8 **INT5-Bi**

Energy(PBE0-D3(BJ)/def2-TZVPP) = -2497.15512423264

6	-1.656375000	-0.879496000	-2.144081000
7	-0.852423000	-0.619248000	-1.063636000
6	0.212491000	-1.439523000	-1.124869000
6	-1.092164000	-1.886100000	-2.857746000
1	-1.436647000	-2.372610000	-3.761988000
1	-2.585730000	-0.345888000	-2.312405000
7	0.059893000	-2.248779000	-2.203541000
6	0.932775000	-3.353596000	-2.710474000
83	1.968297000	-1.312270000	0.368545000
7	-0.848915000	0.180641000	1.406083000
6	0.197188000	-0.542761000	1.828569000
6	-0.999464000	0.073693000	3.605313000
7	0.103632000	-0.634427000	3.180801000
1	-1.292031000	0.161433000	4.644805000
1	-2.485826000	1.185350000	2.389446000
6	-1.589233000	0.582302000	2.491881000
6	0.960261000	-1.477572000	4.059909000
5	-1.269434000	0.557449000	-0.062713000
6	2.406071000	-0.991831000	4.008743000
1	2.862980000	-1.123101000	3.018638000
1	2.471954000	0.071768000	4.274471000
1	3.010455000	-1.572168000	4.720402000
6	0.465639000	-1.375711000	5.500365000
1	0.530779000	-0.346401000	5.882137000
1	-0.568292000	-1.734514000	5.607220000
1	1.105492000	-2.006988000	6.131779000
6	0.842865000	-2.930580000	3.598692000
1	-0.206472000	-3.259667000	3.620018000
1	1.225710000	-3.070197000	2.577459000
1	1.428520000	-3.581200000	4.264025000
6	0.045888000	-4.392650000	-3.400270000
1	-0.750892000	-4.752904000	-2.732041000
1	-0.407950000	-4.013990000	-4.326281000
1	0.673950000	-5.249527000	-3.679358000
6	1.654232000	-4.038748000	-1.553771000
1	2.484663000	-3.423669000	-1.184804000
1	0.959083000	-4.299351000	-0.740947000
1	2.105018000	-4.969834000	-1.923987000
6	1.928000000	-2.758959000	-3.705300000
1	1.398034000	-2.251986000	-4.525711000
1	2.597901000	-2.053500000	-3.193868000
1	2.538712000	-3.566316000	-4.136797000
6	-0.601952000	1.947982000	-0.529212000
6	-0.579986000	2.322002000	-1.880003000
6	-0.101905000	2.882697000	0.386987000
6	-0.064593000	3.546587000	-2.298126000
6	0.414711000	4.109852000	-0.022085000
6	0.453939000	4.462515000	-1.376072000
1	-0.958407000	1.632557000	-2.639917000

1	-0.092915000	2.642525000	1.454566000
1	-0.045766000	3.791502000	-3.364041000
1	0.817522000	4.802772000	0.722975000
6	-2.896285000	0.620134000	-0.068479000
6	-3.652089000	-0.507035000	0.303740000
6	-3.614813000	1.769783000	-0.408385000
6	-5.041354000	-0.487382000	0.329162000
6	-5.012650000	1.798567000	-0.383408000
6	-5.752452000	0.672775000	-0.015901000
1	-3.134500000	-1.431493000	0.582513000
1	-3.071063000	2.672069000	-0.701557000
1	-5.592226000	-1.386630000	0.622537000
1	-5.538601000	2.718580000	-0.656126000
6	1.097251000	5.741249000	-1.820973000
1	0.764770000	6.034977000	-2.826947000
1	0.881990000	6.567242000	-1.126701000
1	2.193554000	5.620811000	-1.854013000
6	-7.253238000	0.691616000	0.016545000
1	-7.632544000	0.493543000	1.032202000
1	-7.650671000	1.663350000	-0.308792000
1	-7.676796000	-0.085762000	-0.639401000
6	2.461975000	0.864674000	-0.125651000
6	3.029263000	1.673522000	0.842235000
6	2.472353000	1.353505000	-1.422888000
6	3.572363000	2.905641000	0.491076000
6	3.035855000	2.603114000	-1.671906000
7	3.574926000	3.346484000	-0.738606000
9	1.958029000	0.668019000	-2.434861000
9	3.085714000	1.275860000	2.117709000
9	4.110829000	3.673575000	1.426202000
9	3.040576000	3.076310000	-2.907748000
8	3.624501000	-1.515085000	-1.347466000
6	4.764361000	-1.965059000	-0.882530000
6	5.396619000	-1.390251000	0.250592000
6	5.413632000	-3.075088000	-1.479974000
6	6.589499000	-1.904601000	0.755863000
6	6.607979000	-3.573375000	-0.972736000
6	7.207376000	-3.000448000	0.153825000
1	4.951659000	-0.502724000	0.710641000
1	4.949786000	-3.529201000	-2.359805000
1	7.047835000	-1.431145000	1.629291000
1	7.082119000	-4.428924000	-1.463305000
1	8.145367000	-3.397784000	0.548134000

6.13.9 **TS4-Bi**

Energy(PBE0-D3(BJ)/def2-SVP) = -2497.108711543322

6	1.755463000	0.356096000	2.477019000
7	1.363723000	-0.193697000	1.283580000
6	0.409599000	-1.106169000	1.531165000
6	1.044337000	-0.239903000	3.467496000
1	1.072441000	-0.078573000	4.538984000
1	2.525978000	1.117953000	2.533411000
7	0.222245000	-1.168482000	2.872766000
6	-0.739289000	-1.974485000	3.681034000
83	-0.779437000	-2.093234000	-0.152421000
7	1.998882000	-0.751124000	-1.168141000
6	1.034350000	-1.614269000	-1.512231000
6	2.648843000	-1.736842000	-3.031783000
7	1.424006000	-2.237322000	-2.654310000
1	3.177293000	-2.063665000	-3.919120000
1	3.881183000	-0.178017000	-2.041425000
6	2.999977000	-0.808384000	-2.102502000
6	0.689056000	-3.309860000	-3.381857000
5	1.929741000	0.392009000	-0.086434000
6	-0.733205000	-2.837220000	-3.666696000
1	-1.340202000	-2.708049000	-2.761856000
1	-0.727865000	-1.878079000	-4.204450000
1	-1.249798000	-3.582840000	-4.287335000
6	1.383014000	-3.586788000	-4.712693000
1	1.421914000	-2.688575000	-5.346189000
1	2.401713000	-3.976828000	-4.575803000
1	0.805666000	-4.352059000	-5.248957000
6	0.707786000	-4.581603000	-2.534314000
1	1.742337000	-4.898318000	-2.335510000
1	0.199368000	-4.438256000	-1.569677000
1	0.190799000	-5.391375000	-3.068844000
6	-1.211981000	-3.204577000	2.918064000
1	-1.920100000	-2.940550000	2.121106000
1	-0.370314000	-3.787062000	2.514923000
1	-1.764758000	-3.849481000	3.615237000
6	-1.929787000	-1.079318000	4.023742000
1	-1.605935000	-0.195795000	4.593366000
1	-2.428170000	-0.745419000	3.104089000
1	-2.650808000	-1.640653000	4.635845000
6	-0.023181000	-2.445544000	4.948802000
1	0.881992000	-3.020706000	4.702342000
1	0.255667000	-1.615721000	5.612195000
1	-0.703455000	-3.096854000	5.514622000
6	0.964017000	1.564547000	-0.633164000
6	0.706626000	2.694910000	0.157128000
6	0.410153000	1.566566000	-1.920200000
6	-0.055333000	3.761743000	-0.307946000
6	-0.364455000	2.626155000	-2.389450000
6	-0.614489000	3.745531000	-1.591183000
1	1.113903000	2.750413000	1.171110000

1	0.581785000	0.719889000	-2.591423000
1	-0.240266000	4.620212000	0.343741000
1	-0.792525000	2.579404000	-3.395266000
6	3.456225000	0.870616000	0.190447000
6	4.385956000	-0.019975000	0.757956000
6	3.938602000	2.133896000	-0.166090000
6	5.714370000	0.333626000	0.966458000
6	5.272274000	2.497226000	0.040330000
6	6.184445000	1.607490000	0.612757000
1	4.058862000	-1.025417000	1.044592000
1	3.256354000	2.856867000	-0.621191000
1	6.405955000	-0.389207000	1.410675000
1	5.610961000	3.495629000	-0.252741000
6	-1.509926000	4.849388000	-2.066781000
1	-1.349736000	5.776128000	-1.497192000
1	-1.357360000	5.064464000	-3.135137000
1	-2.563749000	4.550846000	-1.938518000
6	7.614224000	1.994724000	0.856606000
1	8.308115000	1.230236000	0.473520000
1	7.859646000	2.952285000	0.375936000
1	7.817809000	2.100085000	1.935120000
6	-2.400360000	-0.311985000	-0.226484000
6	-2.263679000	0.967745000	0.385082000
6	-3.054072000	-0.224709000	-1.484928000
6	-2.894859000	2.069323000	-0.140992000
6	-3.595826000	0.955665000	-1.940318000
7	-3.561224000	2.093477000	-1.281781000
9	-1.615263000	1.062989000	1.559715000
9	-3.152332000	-1.354136000	-2.225330000
9	-2.858443000	3.212738000	0.544210000
9	-4.199004000	0.965031000	-3.128785000
8	-3.099569000	-1.346242000	0.843522000
6	-4.407689000	-1.627117000	0.681770000
6	-5.371203000	-0.616825000	0.542021000
6	-4.813136000	-2.969385000	0.661855000
6	-6.713867000	-0.955918000	0.385974000
6	-6.159784000	-3.295529000	0.526826000
6	-7.118264000	-2.291015000	0.381671000
1	-5.065260000	0.430258000	0.569192000
1	-4.053021000	-3.749079000	0.757051000
1	-7.454700000	-0.159881000	0.274531000
1	-6.462468000	-4.346078000	0.524825000
1	-8.173744000	-2.547476000	0.265912000

6.14 Mechanism B (Redox-neutral S_NAr without ligand scrambling) (Bismuth) – xyz coordinates (in Å) and Energies (in Hartree)

6.14.1 **TS5-Bi**

Energy(PBE0-D3(BJ)/def2-TZVPP) = -3241.197566785109

6	-7.970867000	-2.830029000	4.287018000
7	-7.301940000	-2.505975000	5.438026000
6	-6.053610000	-2.989308000	5.355395000
6	-7.119667000	-3.530513000	3.494490000
1	-7.274620000	-3.953334000	2.507880000
1	-9.001557000	-2.536212000	4.120102000
7	-5.927697000	-3.635423000	4.171238000
6	-4.748608000	-4.288050000	3.526723000
83	-4.602566000	-2.873798000	7.128426000
7	-7.552680000	-1.943822000	7.964594000
6	-6.354311000	-2.262115000	8.472582000
6	-7.755836000	-1.985321000	10.160623000
7	-6.460009000	-2.296304000	9.821926000
1	-8.097471000	-1.936653000	11.189031000
1	-9.469510000	-1.502487000	8.837542000
6	-8.431033000	-1.768903000	9.002706000
6	-5.427243000	-2.487590000	10.882194000
5	-7.932208000	-1.500414000	6.487347000
6	-4.090674000	-2.889020000	10.281736000
1	-4.150586000	-3.849047000	9.750284000
1	-3.678390000	-2.093706000	9.640817000
1	-3.371052000	-3.029225000	11.099905000
6	-5.274096000	-1.160288000	11.628738000
1	-4.953285000	-0.361371000	10.947767000
1	-6.215865000	-0.855586000	12.107017000
1	-4.517459000	-1.275219000	12.417965000
6	-5.904490000	-3.583761000	11.835080000
1	-6.857648000	-3.324109000	12.315866000
1	-6.011964000	-4.543834000	11.315238000
1	-5.157397000	-3.714781000	12.630600000
6	-5.175737000	-5.662663000	3.010234000
1	-5.506091000	-6.309771000	3.832879000
1	-5.973135000	-5.597306000	2.257752000
1	-4.310897000	-6.142910000	2.531683000
6	-3.605546000	-4.469950000	4.512079000
1	-3.171553000	-3.508373000	4.819493000
1	-3.901460000	-5.074139000	5.379759000
1	-2.800801000	-5.019879000	4.005154000
6	-4.306231000	-3.391298000	2.368703000
1	-5.097018000	-3.294601000	1.610530000
1	-4.042987000	-2.388755000	2.731345000
1	-3.423162000	-3.830602000	1.882822000
6	-7.348531000	-0.017928000	6.224935000
6	-6.793995000	0.372020000	4.998635000
6	-7.455291000	0.975713000	7.208250000

6	-6.350124000	1.673192000	4.772104000
6	-7.014276000	2.278594000	6.990946000
6	-6.444520000	2.652626000	5.767972000
1	-6.691824000	-0.358911000	4.190244000
1	-7.886005000	0.728292000	8.183123000
1	-5.910993000	1.934734000	3.804359000
1	-7.101636000	3.021218000	7.789330000
6	-9.541241000	-1.583604000	6.343651000
6	-10.209378000	-2.798906000	6.577001000
6	-10.331638000	-0.490988000	5.975262000
6	-11.588561000	-2.914528000	6.452606000
6	-11.719322000	-0.600450000	5.847332000
6	-12.374289000	-1.811584000	6.084018000
1	-9.634943000	-3.683133000	6.865300000
1	-9.855354000	0.474093000	5.781459000
1	-12.068181000	-3.879586000	6.644681000
1	-12.305706000	0.276494000	5.556888000
6	-5.907885000	4.034781000	5.545627000
1	-6.124991000	4.393292000	4.528262000
1	-6.327744000	4.752264000	6.264871000
1	-4.811100000	4.044140000	5.665722000
6	-13.864311000	-1.939237000	5.954199000
1	-14.324075000	-2.226338000	6.913718000
1	-14.322915000	-0.994840000	5.628895000
1	-14.135848000	-2.718901000	5.224623000
6	-4.096023000	-0.608597000	6.673736000
6	-4.255588000	0.442555000	7.560065000
6	-3.430008000	-0.294523000	5.501244000
6	-3.778355000	1.711497000	7.235659000
6	-2.980134000	0.999390000	5.260309000
7	-3.150194000	1.972347000	6.118537000
9	-3.208155000	-1.234373000	4.570504000
9	-4.860468000	0.277352000	8.737707000
9	-3.960406000	2.708882000	8.085365000
9	-2.355196000	1.277196000	4.127639000
8	-5.733084000	-5.231733000	7.532258000
6	-7.054296000	-5.517339000	7.520606000
6	-7.809457000	-5.415902000	8.694711000
6	-7.666503000	-5.966991000	6.344740000
6	-9.158231000	-5.763826000	8.691513000
6	-9.015054000	-6.314953000	6.347100000
6	-9.763960000	-6.221401000	7.520879000
1	-7.318779000	-5.071174000	9.605361000
1	-7.063580000	-6.050799000	5.439842000
1	-9.738735000	-5.683242000	9.613714000
1	-9.483082000	-6.668491000	5.425223000
1	-10.820138000	-6.500254000	7.521995000
6	-4.787766000	-6.541158000	7.920046000
6	-5.052598000	-6.977534000	9.257505000
6	-5.687800000	-8.171483000	9.503078000
7	-5.983447000	-9.071456000	8.589653000
6	-5.631986000	-8.764561000	7.358907000
6	-4.995575000	-7.604882000	6.983715000

9	-6.009986000	-8.460762000	10.761999000
9	-4.794440000	-6.111000000	10.257860000
9	-3.612918000	-5.835808000	7.761219000
9	-4.685373000	-7.372016000	5.692883000
9	-5.893824000	-9.666076000	6.415402000

6.14.2 **INT6-Bi**

Energy(PBE0-D3(BJ)/def2-TZVPP) = -3241.198133614489

6	-7.979889000	-2.855438000	4.307778000
7	-7.306656000	-2.520524000	5.452689000
6	-6.054246000	-2.993065000	5.363953000
6	-7.127347000	-3.551959000	3.512798000
1	-7.284861000	-3.979641000	2.528773000
1	-9.014051000	-2.571188000	4.145810000
7	-5.929954000	-3.642704000	4.181271000
6	-4.743177000	-4.268946000	3.523295000
83	-4.595638000	-2.863606000	7.131932000
7	-7.549476000	-1.942884000	7.974386000
6	-6.351011000	-2.265715000	8.480005000
6	-7.746838000	-1.981605000	10.170953000
7	-6.453532000	-2.299178000	9.829880000
1	-8.085871000	-1.930982000	11.200092000
1	-9.461955000	-1.492370000	8.851808000
6	-8.424096000	-1.762782000	9.014456000
6	-5.417191000	-2.488420000	10.888313000
5	-7.931184000	-1.505203000	6.495787000
6	-4.094634000	-2.934425000	10.287929000
1	-4.183074000	-3.893965000	9.759649000
1	-3.657071000	-2.154373000	9.644380000
1	-3.378055000	-3.091989000	11.105634000
6	-5.230098000	-1.149201000	11.604986000
1	-4.889559000	-0.373088000	10.907949000
1	-6.164306000	-0.811232000	12.075948000
1	-4.476593000	-1.266438000	12.396910000
6	-5.913770000	-3.549420000	11.871125000
1	-6.845147000	-3.244579000	12.367636000
1	-6.068350000	-4.514194000	11.372790000
1	-5.154959000	-3.692075000	12.653361000
6	-5.159671000	-5.629518000	2.962886000
1	-5.516276000	-6.293913000	3.759955000
1	-5.933349000	-5.543498000	2.187966000
1	-4.282570000	-6.100795000	2.498048000
6	-3.602978000	-4.471215000	4.508408000
1	-3.172116000	-3.515918000	4.838919000
1	-3.895743000	-5.096832000	5.361765000
1	-2.793874000	-5.003491000	3.989690000
6	-4.303562000	-3.335172000	2.393809000
1	-5.094571000	-3.219570000	1.638444000
1	-4.044772000	-2.342983000	2.786618000
1	-3.417908000	-3.755777000	1.896226000
6	-7.341863000	-0.027547000	6.220055000
6	-6.773815000	0.347344000	4.995225000
6	-7.462589000	0.978675000	7.188810000
6	-6.330214000	1.646554000	4.756627000
6	-7.021635000	2.279416000	6.959604000
6	-6.438559000	2.638715000	5.738368000
1	-6.661704000	-0.393475000	4.197204000

1	-7.904609000	0.742699000	8.161453000
1	-5.881362000	1.896642000	3.790345000
1	-7.120152000	3.032336000	7.746919000
6	-9.540513000	-1.579944000	6.353376000
6	-10.215743000	-2.787762000	6.602839000
6	-10.324046000	-0.489903000	5.963055000
6	-11.594564000	-2.900013000	6.472208000
6	-11.711436000	-0.595526000	5.829125000
6	-12.373230000	-1.799910000	6.081066000
1	-9.646280000	-3.668928000	6.908597000
1	-9.842664000	0.469738000	5.755541000
1	-12.079278000	-3.859984000	6.676548000
1	-12.292223000	0.279097000	5.521053000
6	-5.903646000	4.019527000	5.503998000
1	-6.111327000	4.364430000	4.479982000
1	-6.333257000	4.744655000	6.209660000
1	-4.808208000	4.034399000	5.635358000
6	-13.862928000	-1.923357000	5.944114000
1	-14.330156000	-2.188059000	6.906467000
1	-14.314966000	-0.983936000	5.596087000
1	-14.133351000	-2.717005000	5.229395000
6	-4.085208000	-0.609182000	6.688955000
6	-4.271355000	0.445893000	7.566096000
6	-3.391804000	-0.300095000	5.530597000
6	-3.791853000	1.714935000	7.244495000
6	-2.944817000	0.995305000	5.291964000
7	-3.140310000	1.972003000	6.140212000
9	-3.141760000	-1.244881000	4.613482000
9	-4.902658000	0.285973000	8.728934000
9	-3.995528000	2.715167000	8.085005000
9	-2.295948000	1.269503000	4.172653000
8	-5.795887000	-5.292891000	7.551354000
6	-7.134420000	-5.569658000	7.539237000
6	-7.879571000	-5.448850000	8.712647000
6	-7.744462000	-5.991429000	6.357144000
6	-9.237044000	-5.762699000	8.703803000
6	-9.101730000	-6.305562000	6.355366000
6	-9.848907000	-6.200291000	7.528952000
1	-7.378575000	-5.121112000	9.623745000
1	-7.138579000	-6.084238000	5.455272000
1	-9.818482000	-5.670829000	9.624125000
1	-9.577111000	-6.640440000	5.430532000
1	-10.911676000	-6.452788000	7.526135000
6	-4.912818000	-6.478238000	7.896271000
6	-5.113344000	-6.970735000	9.239616000
6	-5.513959000	-8.256138000	9.499610000
7	-5.688534000	-9.199179000	8.596258000
6	-5.449355000	-8.825333000	7.355483000
6	-5.043056000	-7.574940000	6.963871000
9	-5.736808000	-8.594491000	10.770235000
9	-4.979988000	-6.071851000	10.243024000
9	-3.690535000	-5.769789000	7.753090000
9	-4.846323000	-7.292077000	5.655215000

9	-5.609796000	-9.752077000	6.410318000

6.14.3 **TS6-Bi**

Energy(PBE0-D3(BJ)/def2-TZVPP) = -3241.194292098415

6	-7.855689000	-3.126377000	4.410818000
7	-7.183727000	-2.659432000	5.508676000
6	-5.891199000	-3.000243000	5.391231000
6	-6.958611000	-3.764543000	3.613232000
1	-7.102793000	-4.257361000	2.658719000
1	-8.920296000	-2.965058000	4.277584000
7	-5.735329000	-3.685912000	4.232951000
6	-4.480467000	-4.104681000	3.537264000
83	-4.436435000	-2.748344000	7.152081000
7	-7.433650000	-1.937925000	7.983994000
6	-6.242990000	-2.291612000	8.486139000
6	-7.614111000	-1.935673000	10.182785000
7	-6.337971000	-2.313856000	9.835185000
1	-7.945615000	-1.866957000	11.213282000
1	-9.322647000	-1.410048000	8.869796000
6	-8.291349000	-1.706821000	9.028443000
6	-5.312761000	-2.591629000	10.883170000
5	-7.833665000	-1.595094000	6.486476000
6	-4.038772000	-3.155201000	10.275815000
1	-4.225376000	-4.064052000	9.684250000
1	-3.497585000	-2.398519000	9.684685000
1	-3.361108000	-3.436565000	11.093555000
6	-5.009126000	-1.275590000	11.600065000
1	-4.635439000	-0.523532000	10.892075000
1	-5.904408000	-0.871335000	12.094291000
1	-4.244976000	-1.446883000	12.371788000
6	-5.884898000	-3.621854000	11.858025000
1	-6.794312000	-3.260251000	12.356848000
1	-6.105190000	-4.566706000	11.344503000
1	-5.138275000	-3.824111000	12.638579000
6	-4.740310000	-5.385012000	2.743337000
1	-5.083383000	-6.197882000	3.394450000
1	-5.465801000	-5.236028000	1.931923000
1	-3.796024000	-5.693049000	2.273604000
6	-3.367723000	-4.368641000	4.541869000
1	-2.964268000	-3.434961000	4.960021000
1	-3.692835000	-5.045787000	5.344248000
1	-2.528261000	-4.845166000	4.017462000
6	-4.097742000	-2.968252000	2.587276000
1	-4.884763000	-2.804488000	1.836155000
1	-3.936843000	-2.033144000	3.139607000
1	-3.167391000	-3.223532000	2.059203000
6	-7.273986000	-0.126756000	6.108067000
6	-6.633340000	0.167117000	4.898071000
6	-7.502728000	0.953235000	6.973454000
6	-6.214903000	1.458713000	4.579880000
6	-7.089410000	2.245586000	6.664971000
6	-6.426579000	2.523729000	5.462013000
1	-6.442201000	-0.630603000	4.173138000

1	-8.010820000	0.782155000	7.926927000
1	-5.707297000	1.643996000	3.628304000
1	-7.274251000	3.058104000	7.373603000
6	-9.443244000	-1.694578000	6.344335000
6	-10.135360000	-2.847789000	6.748832000
6	-10.210460000	-0.672590000	5.770055000
6	-11.513216000	-2.972012000	6.595044000
6	-11.591668000	-0.790777000	5.611273000
6	-12.271484000	-1.941559000	6.024574000
1	-9.582724000	-3.678116000	7.193834000
1	-9.715889000	0.241914000	5.431688000
1	-12.008928000	-3.891027000	6.921386000
1	-12.153995000	0.029239000	5.154072000
6	-5.921350000	3.901215000	5.153649000
1	-5.957011000	4.113308000	4.074878000
1	-6.502416000	4.672071000	5.680107000
1	-4.869397000	4.004093000	5.471444000
6	-13.761665000	-2.056685000	5.884972000
1	-14.275215000	-1.569035000	6.730820000
1	-14.119476000	-1.568746000	4.966161000
1	-14.084676000	-3.107636000	5.868143000
6	-4.045858000	-0.455750000	6.811937000
6	-4.382862000	0.584783000	7.660973000
6	-3.220867000	-0.116349000	5.752574000
6	-3.929424000	1.875715000	7.389834000
6	-2.812914000	1.198646000	5.559916000
7	-3.162609000	2.164650000	6.370425000
9	-2.801383000	-1.056093000	4.892062000
9	-5.135364000	0.395660000	8.743936000
9	-4.283011000	2.866947000	8.190689000
9	-2.041615000	1.504595000	4.530063000
8	-6.436862000	-5.246305000	7.736319000
6	-7.761225000	-5.580574000	7.883602000
6	-8.386602000	-5.314283000	9.099710000
6	-8.480180000	-6.132848000	6.822494000
6	-9.740394000	-5.604230000	9.257911000
6	-9.830201000	-6.432175000	6.993792000
6	-10.463642000	-6.171764000	8.209281000
1	-7.801407000	-4.881842000	9.910701000
1	-7.977752000	-6.313587000	5.872349000
1	-10.230420000	-5.388840000	10.210300000
1	-10.393813000	-6.865060000	6.163998000
1	-11.522914000	-6.405998000	8.337179000
6	-5.529174000	-6.335108000	7.748403000
6	-5.433999000	-7.079712000	8.971998000
6	-5.219756000	-8.436712000	8.971053000
7	-5.171606000	-9.196550000	7.895684000
6	-5.351233000	-8.570350000	6.752827000
6	-5.569352000	-7.220770000	6.621992000
9	-5.074059000	-9.050438000	10.144583000
9	-5.477433000	-6.387125000	10.128848000
9	-4.254112000	-5.395338000	7.621869000
9	-5.772931000	-6.663630000	5.407237000

9	-5.343715000	-9.316031000	5.648117000

6.15 Mechanism C (Redox-neutral S_NAr after ligand scrambling) – xyz coordinates (in Å) and Energies (in Hartree)

6.15.1 **INT7-Bi**

Energy(PBE0-D3(BJ)/def2-TZVPP) = -2159.56751032937

6	-1.694516000	-0.933426000	-2.105709000
7	-0.998539000	-0.774171000	-0.932964000
6	0.101414000	-1.540289000	-0.987777000
6	-1.007604000	-1.806724000	-2.884692000
1	-1.227399000	-2.184199000	-3.877708000
1	-2.625527000	-0.410568000	-2.296922000
7	0.114428000	-2.176284000	-2.179289000
6	1.143065000	-3.055008000	-2.801532000
83	1.594110000	-1.665341000	0.744733000
7	-1.154663000	-0.142408000	1.580417000
6	-0.085499000	-0.772463000	2.085738000
6	-1.429693000	-0.245656000	3.767537000
7	-0.241468000	-0.849739000	3.432081000
1	-1.791454000	-0.169146000	4.785523000
1	-2.923141000	0.727330000	2.444802000
6	-1.992095000	0.195540000	2.610811000
6	0.634421000	-1.557295000	4.413399000
5	-1.416190000	0.358740000	0.098673000
6	2.087691000	-1.135149000	4.214596000
1	2.512786000	-1.475618000	3.260153000
1	2.196991000	-0.043105000	4.276536000
1	2.702752000	-1.590143000	5.003290000
6	0.222103000	-1.160200000	5.829686000
1	0.281512000	-0.072388000	5.982407000
1	-0.790307000	-1.507057000	6.079622000
1	0.914354000	-1.640159000	6.534491000
6	0.449751000	-3.062128000	4.228148000
1	-0.599419000	-3.340671000	4.409619000
1	0.715357000	-3.392578000	3.212085000
1	1.079052000	-3.601500000	4.951261000
6	0.473119000	-4.372867000	-3.187863000
1	0.053199000	-4.866774000	-2.299532000
1	-0.332037000	-4.224585000	-3.921349000
1	1.219327000	-5.040990000	-3.641348000
6	2.293262000	-3.331855000	-1.846920000
1	2.849113000	-2.409199000	-1.614896000
1	1.953841000	-3.836613000	-0.929283000
1	3.002025000	-4.006059000	-2.346960000
6	1.686841000	-2.318650000	-4.027700000
1	0.903885000	-2.134817000	-4.777085000
1	2.118910000	-1.351753000	-3.728527000
1	2.473437000	-2.923626000	-4.500940000
6	-0.558316000	1.698238000	-0.170719000
6	-0.077024000	2.029360000	-1.444341000
6	-0.339557000	2.639682000	0.842958000

6	0.596818000	3.222311000	-1.688358000
6	0.329583000	3.839554000	0.605730000
6	0.820738000	4.150623000	-0.664374000
1	-0.215540000	1.330344000	-2.274637000
1	-0.695393000	2.436285000	1.858150000
1	0.969951000	3.436367000	-2.694766000
1	0.491264000	4.540962000	1.429886000
6	-3.015650000	0.589089000	-0.056491000
6	-3.923527000	-0.464947000	0.156245000
6	-3.565177000	1.825486000	-0.409156000
6	-5.296767000	-0.293097000	0.025046000
6	-4.944629000	2.006405000	-0.543487000
6	-5.836408000	0.953047000	-0.329217000
1	-3.544025000	-1.453967000	0.432094000
1	-2.897626000	2.673332000	-0.586332000
1	-5.968400000	-1.139688000	0.199054000
1	-5.335691000	2.989662000	-0.822373000
6	1.611290000	5.401538000	-0.910482000
1	1.482412000	5.767602000	-1.940004000
1	1.324395000	6.206057000	-0.217250000
1	2.687095000	5.207441000	-0.761052000
6	-7.319913000	1.136468000	-0.468418000
1	-7.837845000	0.933313000	0.482931000
1	-7.571135000	2.160831000	-0.777801000
1	-7.739819000	0.443163000	-1.214735000
8	2.253745000	0.210492000	-0.333687000

6.15.2 **TS7-Bi**

Energy(PBE0-D3(BJ)/def2-TZVPP) = -2903.59029482337

6	-6.508652000	-4.826621000	3.054876000
7	-5.909321000	-4.425809000	4.221000000
6	-4.607521000	-4.742719000	4.149486000
6	-5.555217000	-5.385326000	2.261888000
1	-5.633726000	-5.804595000	1.265239000
1	-7.567499000	-4.671872000	2.874041000
7	-4.369237000	-5.325049000	2.953762000
6	-3.055819000	-5.589824000	2.293402000
83	-3.186654000	-4.490912000	5.913719000
7	-6.225857000	-3.608243000	6.671054000
6	-4.995331000	-3.787003000	7.166640000
6	-6.360152000	-3.408040000	8.866040000
7	-5.058415000	-3.670544000	8.513404000
1	-6.682661000	-3.266713000	9.890589000
1	-8.141348000	-3.189598000	7.555548000
6	-7.082593000	-3.370286000	7.711662000
6	-3.913701000	-3.783001000	9.465293000
5	-6.625764000	-3.358449000	5.160310000
6	-2.799524000	-2.838156000	9.018667000
1	-2.295664000	-3.168008000	8.098885000
1	-3.177537000	-1.820445000	8.847917000
1	-2.020840000	-2.801154000	9.793075000
6	-4.378634000	-3.361084000	10.855962000
1	-4.743777000	-2.323684000	10.865396000
1	-5.162123000	-4.025547000	11.247393000
1	-3.521409000	-3.424340000	11.539619000
6	-3.438827000	-5.232824000	9.514173000
1	-4.228939000	-5.893918000	9.894842000
1	-3.131031000	-5.628172000	8.536038000
1	-2.573221000	-5.311113000	10.187364000
6	-3.178605000	-6.822453000	1.399464000
1	-3.472241000	-7.705451000	1.981192000
1	-3.893602000	-6.674534000	0.578587000
1	-2.199392000	-7.017142000	0.940739000
6	-1.957998000	-5.825270000	3.322278000
1	-1.642003000	-4.880744000	3.793618000
1	-2.242478000	-6.571553000	4.076983000
1	-1.067568000	-6.204471000	2.802621000
6	-2.725596000	-4.344461000	1.467144000
1	-3.494517000	-4.161127000	0.702093000
1	-2.658468000	-3.461178000	2.120312000
1	-1.759244000	-4.480531000	0.960102000
6	-6.119378000	-1.877538000	4.748328000
6	-5.499288000	-1.584381000	3.528291000
6	-6.361236000	-0.789923000	5.601668000
6	-5.126234000	-0.286050000	3.182677000
6	-5.997019000	0.511121000	5.262811000
6	-5.361175000	0.788305000	4.046057000
1	-5.286805000	-2.387341000	2.816206000

1	-6.851381000	-0.957545000	6.565919000
1	-4.632276000	-0.104420000	2.223429000
1	-6.202269000	1.329605000	5.959745000
6	-8.223609000	-3.542123000	5.008883000
6	-8.843960000	-4.735741000	5.414564000
6	-9.051412000	-2.560201000	4.453958000
6	-10.212442000	-4.937465000	5.277084000
6	-10.427425000	-2.756208000	4.311377000
6	-11.034629000	-3.946607000	4.720316000
1	-8.237076000	-5.531824000	5.850613000
1	-8.612719000	-1.615210000	4.121711000
1	-10.652729000	-5.883464000	5.607359000
1	-11.043047000	-1.965249000	3.872350000
6	-4.904912000	2.177276000	3.707370000
1	-4.850855000	2.331452000	2.619854000
1	-5.575052000	2.938849000	4.132931000
1	-3.896550000	2.364918000	4.114489000
6	-12.512358000	-4.166941000	4.574236000
1	-12.986441000	-4.349053000	5.552158000
1	-13.005982000	-3.298711000	4.115323000
1	-12.723920000	-5.048595000	3.948144000
8	-2.707613000	-2.562745000	5.003550000
6	-2.550410000	-0.476161000	6.123946000
6	-0.644020000	-1.963299000	6.065807000
6	-0.484436000	0.158712000	7.222830000
1	-2.268115000	1.379837000	7.171583000
1	1.126079000	-1.274274000	7.080806000
6	-1.979333000	-1.694196000	5.713672000
6	-1.806139000	0.437247000	6.864411000
6	0.090042000	-1.045673000	6.816210000
1	-3.586200000	-0.273603000	5.844303000
1	-0.186358000	-2.900326000	5.734190000
1	0.093855000	0.877010000	7.808430000
8	-4.799823000	-6.878412000	6.480239000
6	-6.124817000	-7.213612000	6.661296000
6	-6.727078000	-6.903342000	7.877568000
6	-6.849391000	-7.838628000	5.645827000
6	-8.063640000	-7.236501000	8.088027000
6	-8.179802000	-8.185284000	5.872851000
6	-8.788887000	-7.891288000	7.092998000
1	-6.135714000	-6.414139000	8.651381000
1	-6.369918000	-8.043588000	4.688597000
1	-8.536772000	-6.990342000	9.041392000
1	-8.747558000	-8.680188000	5.081611000
1	-9.832614000	-8.163810000	7.264595000
6	-3.908645000	-7.989290000	6.440877000
6	-3.853162000	-8.805319000	7.617254000
6	-3.645116000	-10.161970000	7.530574000
7	-3.569728000	-10.848967000	6.409384000
6	-3.728342000	-10.152402000	5.305897000
6	-3.941172000	-8.796080000	5.259938000
9	-3.539165000	-10.852845000	8.663813000
9	-3.956783000	-8.199587000	8.813510000

9	-2.612326000	-7.044590000	6.380534000
9	-4.144271000	-8.164319000	4.082944000
9	-3.707441000	-10.825383000	4.156138000

6.15.3 **INT8-Bi**

Energy(PBE0-D3(BJ)/def2-TZVPP) = -1952.72639813289

6	-1.557178000	-0.758025000	-2.226999000
7	-0.866242000	-0.614899000	-1.048352000
6	0.193725000	-1.434832000	-1.080445000
6	-0.913617000	-1.685654000	-2.980842000
1	-1.154160000	-2.075733000	-3.962850000
1	-2.456433000	-0.189157000	-2.438819000
7	0.180869000	-2.101236000	-2.256208000
6	1.052047000	-3.223773000	-2.705111000
83	1.695056000	-1.576211000	0.632360000
7	-1.048498000	-0.070223000	1.480897000
6	-0.031975000	-0.794522000	1.965491000
6	-1.362396000	-0.270953000	3.658458000
7	-0.213090000	-0.935693000	3.302406000
1	-1.735815000	-0.228636000	4.674393000
1	-2.772971000	0.860869000	2.371743000
6	-1.876781000	0.267340000	2.520239000
6	0.608159000	-1.763416000	4.234418000
5	-1.304546000	0.482416000	0.013707000
6	2.070943000	-1.330260000	4.155723000
1	2.537854000	-1.551682000	3.185934000
1	2.175216000	-0.253405000	4.351578000
1	2.650446000	-1.879082000	4.911136000
6	0.126620000	-1.537107000	5.665405000
1	0.211790000	-0.482285000	5.966110000
1	-0.910290000	-1.872234000	5.809545000
1	0.760175000	-2.131887000	6.337345000
6	0.423763000	-3.234663000	3.862686000
1	-0.626744000	-3.528791000	4.010143000
1	0.686232000	-3.446398000	2.814388000
1	1.049209000	-3.859204000	4.517677000
6	0.365801000	-4.527824000	-2.296295000
1	0.270640000	-4.556167000	-1.200253000
1	-0.629915000	-4.609084000	-2.757580000
1	0.970379000	-5.386172000	-2.625157000
6	2.428136000	-3.140620000	-2.052664000
1	2.853903000	-2.126762000	-2.114502000
1	2.380385000	-3.498154000	-1.014336000
1	3.110658000	-3.821395000	-2.579670000
6	1.221707000	-3.131502000	-4.221397000
1	0.280281000	-3.294566000	-4.763497000
1	1.636871000	-2.155514000	-4.514280000
1	1.920162000	-3.915472000	-4.544783000
6	-0.493746000	1.856857000	-0.211803000
6	-0.062799000	2.259285000	-1.483195000
6	-0.272350000	2.759216000	0.835525000
6	0.568309000	3.481752000	-1.692897000
6	0.354155000	3.988317000	0.633298000
6	0.797369000	4.370013000	-0.634757000
1	-0.205272000	1.592997000	-2.339362000

1	-0.586928000	2.500112000	1.851312000
1	0.903339000	3.751706000	-2.699386000
1	0.522208000	4.655791000	1.483953000
6	-2.912307000	0.669799000	-0.135104000
6	-3.782013000	-0.421991000	0.043307000
6	-3.504513000	1.899971000	-0.435479000
6	-5.161246000	-0.290796000	-0.070344000
6	-4.890626000	2.040025000	-0.551163000
6	-5.744864000	0.950025000	-0.370284000
1	-3.364401000	-1.406921000	0.279411000
1	-2.866453000	2.775696000	-0.583741000
1	-5.803948000	-1.164762000	0.075803000
1	-5.316792000	3.019880000	-0.787458000
6	1.545544000	5.653175000	-0.845245000
1	1.403577000	6.044351000	-1.863687000
1	1.233012000	6.427702000	-0.129118000
1	2.627611000	5.491955000	-0.700778000
6	-7.235188000	1.089946000	-0.486837000
1	-7.734371000	0.842642000	0.464171000
1	-7.522523000	2.114515000	-0.762313000
1	-7.642757000	0.405940000	-1.248399000
8	2.232068000	0.418804000	-0.304187000
6	2.811782000	1.624938000	1.682340000
6	3.736130000	2.233629000	-0.460611000
6	4.343548000	3.495659000	1.521149000
1	3.426812000	2.824241000	3.358425000
1	5.077427000	3.911224000	-0.469901000
6	2.896327000	1.380576000	0.293453000
6	3.523958000	2.660958000	2.280831000
6	4.439219000	3.268409000	0.143915000
1	2.138793000	1.009785000	2.286107000
1	3.804703000	2.057954000	-1.536888000
1	4.899049000	4.310252000	1.991353000
9	0.915491000	-3.635371000	0.771968000

6.15.5 **TS8-Bi**

Energy(PBE0-D3(BJ)/def2-TZVPP) = -2668.476376394341

6	-2.147909000	-1.359032000	-2.193580000
7	-1.576107000	-1.135797000	-0.965326000
6	-0.442966000	-1.846708000	-0.894262000
6	-1.346419000	-2.212766000	-2.881009000
1	-1.448966000	-2.620005000	-3.881513000
1	-3.077177000	-0.881024000	-2.486366000
7	-0.279588000	-2.506496000	-2.062729000
6	0.872041000	-3.309842000	-2.560446000
83	0.759070000	-2.036941000	1.033025000
7	-1.799418000	-0.098316000	1.441831000
6	-0.713606000	-0.585309000	2.050619000
6	-2.036131000	0.270836000	3.607491000
7	-0.843664000	-0.376269000	3.381334000
1	-2.377980000	0.564062000	4.592754000
1	-3.575573000	0.901789000	2.140313000
6	-2.626555000	0.440763000	2.394643000
6	0.071025000	-0.862406000	4.452230000
5	-2.112205000	0.083188000	-0.099472000
6	1.511722000	-0.495497000	4.106475000
1	1.909643000	-1.065539000	3.255554000
1	1.611933000	0.572740000	3.868163000
1	2.160603000	-0.731367000	4.961400000
6	-0.294460000	-0.181096000	5.768674000
1	-0.236633000	0.913972000	5.684216000
1	-1.296776000	-0.465181000	6.119332000
1	0.424409000	-0.501225000	6.534737000
6	-0.107786000	-2.374250000	4.597833000
1	-1.156259000	-2.622226000	4.816953000
1	0.178025000	-2.921274000	3.687965000
1	0.521337000	-2.745094000	5.419720000
6	0.355366000	-4.690022000	-2.960946000
1	-0.108192000	-5.189654000	-2.099203000
1	-0.386463000	-4.630591000	-3.770041000
1	1.193298000	-5.306815000	-3.315396000
6	1.943650000	-3.454620000	-1.493295000
1	2.346631000	-2.475373000	-1.188056000
1	1.575756000	-4.030292000	-0.632173000
1	2.778691000	-4.031549000	-1.913215000
6	1.464808000	-2.562861000	-3.757015000
1	0.742665000	-2.462012000	-4.579530000
1	1.794297000	-1.557889000	-3.453356000
1	2.335811000	-3.115685000	-4.136713000
6	-1.363470000	1.432225000	-0.595367000
6	-0.461409000	1.471986000	-1.665762000
6	-1.573018000	2.643509000	0.084849000
6	0.213457000	2.639670000	-2.021134000
6	-0.905053000	3.814415000	-0.264436000
6	0.015047000	3.832406000	-1.320263000
1	-0.250350000	0.563657000	-2.236485000

1	-2.269763000	2.677863000	0.927763000
1	0.923211000	2.618828000	-2.853565000
1	-1.092632000	4.733227000	0.299691000
6	-3.723219000	0.082158000	-0.256245000
6	-4.453652000	-1.039481000	0.177853000
6	-4.452087000	1.127861000	-0.829081000
6	-5.836192000	-1.106598000	0.052193000
6	-5.842904000	1.066460000	-0.958515000
6	-6.560547000	-0.048582000	-0.519085000
1	-3.918430000	-1.883723000	0.626011000
1	-3.923538000	2.015380000	-1.189120000
1	-6.369450000	-1.996561000	0.400986000
1	-6.381117000	1.903475000	-1.413824000
6	0.791599000	5.072703000	-1.653266000
1	1.092892000	5.089757000	-2.710803000
1	0.210864000	5.982993000	-1.442882000
1	1.713632000	5.126293000	-1.049629000
6	-8.055258000	-0.119367000	-0.640795000
1	-8.535955000	-0.094425000	0.351101000
1	-8.452153000	0.721967000	-1.226358000
1	-8.373722000	-1.055312000	-1.126082000
8	1.981537000	-0.412141000	0.134515000
6	1.856066000	1.717190000	1.195086000
6	3.813972000	0.328663000	1.473434000
6	3.696496000	2.484785000	2.575085000
1	1.883593000	3.609257000	2.212263000
1	5.373976000	1.130911000	2.722062000
6	2.536364000	0.516161000	0.910988000
6	2.433124000	2.684699000	2.012297000
6	4.381261000	1.300814000	2.295342000
1	0.868972000	1.872033000	0.756439000
1	4.349431000	-0.598590000	1.251026000
1	4.144999000	3.245404000	3.218408000
14	-1.570382000	-4.946960000	1.719930000
9	-1.387933000	-3.154823000	1.531358000
6	-3.148484000	-4.488178000	2.724478000
1	-3.749182000	-3.706106000	2.233796000
1	-3.782746000	-5.377685000	2.873165000
1	-2.881443000	-4.111767000	3.727589000
6	-1.118277000	-6.476310000	2.779957000
1	-0.636990000	-6.157440000	3.721364000
1	-2.035539000	-7.020649000	3.053777000
1	-0.428158000	-7.177641000	2.288594000
6	-2.162174000	-5.469593000	0.008385000
1	-3.174628000	-5.899753000	0.063660000
1	-2.205892000	-4.597878000	-0.664150000
1	-1.498918000	-6.220790000	-0.445816000
8	0.276799000	-4.690060000	1.353609000
6	1.324457000	-5.510468000	1.421988000
6	2.312311000	-5.307725000	2.401725000
6	1.516271000	-6.541384000	0.484338000
6	3.453572000	-6.105826000	2.437718000
6	2.656629000	-7.338590000	0.530923000

6	3.634997000	-7.126820000	1.504276000
1	2.160949000	-4.515350000	3.139433000
1	0.754842000	-6.704641000	-0.281111000

6.15.6 **INT9-Bi**

Energy(PBE0-D3(BJ)/def2-TZVPP) = -2668.47872328111

6	-1.676767000	0.145894000	-1.951285000
7	-0.727011000	-0.367716000	-1.100415000
6	-0.360980000	-1.571072000	-1.550545000
6	-1.897472000	-0.770224000	-2.933748000
1	-2.569372000	-0.731782000	-3.782981000
1	-2.117305000	1.125424000	-1.793919000
7	-1.071344000	-1.840678000	-2.669987000
6	-0.979287000	-3.088302000	-3.480103000
83	1.002803000	-3.021368000	-0.416576000
7	0.435260000	-0.332261000	1.257688000
6	1.089922000	-1.503527000	1.302855000
6	1.203795000	-0.582874000	3.315980000
7	1.562453000	-1.676230000	2.561465000
1	1.477049000	-0.472157000	4.359665000
1	0.023556000	1.198721000	2.717206000
6	0.495841000	0.245327000	2.502610000
6	2.469130000	-2.730224000	3.106156000
5	-0.095297000	0.537204000	0.027798000
6	3.874030000	-2.132070000	3.172457000
1	4.208587000	-1.817497000	2.173371000
1	3.905815000	-1.260747000	3.843440000
1	4.578299000	-2.885597000	3.553491000
6	1.974699000	-3.129699000	4.496296000
1	2.022577000	-2.299360000	5.213912000
1	0.943195000	-3.505370000	4.453236000
1	2.618575000	-3.932098000	4.882122000
6	2.474268000	-3.964873000	2.219266000
1	1.459849000	-4.349684000	2.034843000
1	3.010774000	-3.785718000	1.274225000
1	3.035212000	-4.760041000	2.728501000
6	-1.476766000	-4.266982000	-2.643020000
1	-0.881563000	-4.461931000	-1.739150000
1	-2.511830000	-4.091693000	-2.317032000
1	-1.452472000	-5.184074000	-3.248539000
6	0.465833000	-3.265513000	-3.947078000
1	0.767212000	-2.421753000	-4.585045000
1	1.195992000	-3.317447000	-3.126790000
1	0.552044000	-4.194138000	-4.528740000
6	-1.872677000	-2.956629000	-4.710223000
1	-2.932644000	-2.853951000	-4.436500000
1	-1.576974000	-2.105837000	-5.341066000
1	-1.770952000	-3.871048000	-5.310263000
6	1.168205000	1.340895000	-0.593154000
6	1.513827000	1.293453000	-1.950211000
6	1.984658000	2.137294000	0.227123000
6	2.616000000	1.981721000	-2.457777000
6	3.086292000	2.828879000	-0.271274000
6	3.431101000	2.756660000	-1.627096000
1	0.921331000	0.689465000	-2.643013000

1	1.764745000	2.219293000	1.295592000
1	2.855534000	1.904380000	-3.522626000
1	3.697627000	3.432666000	0.406582000
6	-1.290903000	1.470376000	0.594133000
6	-2.414907000	0.864037000	1.185839000
6	-1.296773000	2.865549000	0.509144000
6	-3.479901000	1.612925000	1.672554000
6	-2.365702000	3.624475000	0.996238000
6	-3.474734000	3.014409000	1.587620000
1	-2.456088000	-0.227417000	1.264520000
1	-0.447206000	3.378802000	0.049335000
1	-4.335648000	1.103907000	2.127396000
1	-2.336232000	4.715282000	0.913375000
6	4.654434000	3.446022000	-2.156887000
1	4.546337000	3.699490000	-3.221640000
1	4.872104000	4.369905000	-1.600816000
1	5.539408000	2.793457000	-2.064993000
6	-4.628874000	3.819116000	2.111642000
1	-4.809938000	3.609984000	3.178102000
1	-4.450161000	4.898139000	2.002330000
1	-5.560515000	3.574338000	1.576148000
14	-2.114178000	-4.218793000	1.509339000
9	-1.359828000	-2.937680000	0.626412000
6	-3.567355000	-3.029055000	1.962781000
1	-4.079862000	-2.644922000	1.063939000
1	-4.325327000	-3.542272000	2.579716000
1	-3.213417000	-2.155043000	2.536148000
6	-1.470635000	-4.483927000	3.267184000
1	-1.200784000	-3.490889000	3.664240000
1	-2.289957000	-4.858546000	3.903035000
1	-0.607196000	-5.153904000	3.373415000
6	-3.134947000	-5.486826000	0.548302000
1	-3.962134000	-5.840945000	1.185554000
1	-3.602769000	-4.959080000	-0.299556000
1	-2.588330000	-6.356443000	0.160783000
8	2.706861000	-2.061967000	-1.446810000
6	0.019489000	-6.301586000	0.887863000
6	4.346300000	-0.682885000	-0.416345000
6	4.736948000	-3.055772000	-0.659790000
6	1.693689000	-7.989114000	0.294270000
6	-0.073206000	-8.563313000	1.810742000
6	6.426968000	-1.654332000	0.366696000
1	1.671403000	-5.979523000	-0.474031000
1	-1.496109000	-7.017673000	2.267429000
1	5.926302000	0.446228000	0.502614000
1	6.621685000	-3.787607000	0.081639000
1	1.479088000	-9.947553000	1.200958000
8	-0.423893000	-5.064660000	0.767999000
6	3.893476000	-1.943110000	-0.854884000
6	1.177722000	-6.702867000	0.179942000
6	-0.596913000	-7.275982000	1.707467000
6	5.598180000	-0.545183000	0.176966000
6	5.985101000	-2.908993000	-0.055806000

6	1.075070000	-8.936700000	1.112586000
1	3.696242000	0.180590000	-0.563848000
1	4.399352000	-4.036776000	-1.007826000

6.15.7 **TS10-Bi**

Energy(PBE0-D3(BJ)/def2-TZVPP) = -2696.750553054760

6	-6.257337000	-2.153698000	2.492202000
7	-5.930236000	-2.516588000	3.772632000
6	-5.382062000	-3.739976000	3.735031000
6	-5.909331000	-3.172971000	1.665223000
1	-6.009288000	-3.264845000	0.588835000
1	-6.718138000	-1.198779000	2.263079000
7	-5.374793000	-4.163958000	2.450616000
6	-4.986863000	-5.479370000	1.864514000
83	-4.323549000	-4.695235000	5.518224000
7	-5.721840000	-1.879254000	6.312034000
6	-5.065147000	-2.939228000	6.799184000
6	-5.289997000	-1.472207000	8.439751000
7	-4.792967000	-2.708319000	8.107063000
1	-5.200528000	-1.040865000	9.429203000
1	-6.364334000	-0.010266000	7.161953000
6	-5.864651000	-0.960571000	7.318672000
6	-4.267603000	-3.699987000	9.091654000
5	-6.577683000	-1.723814000	4.987344000
6	-2.932199000	-4.269372000	8.620986000
1	-3.028319000	-4.954768000	7.767024000
1	-2.230799000	-3.468263000	8.353027000
1	-2.491691000	-4.865281000	9.432164000
6	-4.036866000	-3.001267000	10.428701000
1	-3.310698000	-2.180078000	10.334879000
1	-4.970137000	-2.614230000	10.861063000
1	-3.628122000	-3.736489000	11.134736000
6	-5.323148000	-4.796062000	9.251226000
1	-6.259116000	-4.369457000	9.642123000
1	-5.554030000	-5.292048000	8.295850000
1	-4.964688000	-5.555456000	9.961262000
6	-6.214299000	-6.031428000	1.134498000
1	-7.059274000	-6.145516000	1.829857000
1	-6.530265000	-5.386315000	0.303028000
1	-5.971202000	-7.018248000	0.715910000
6	-4.585623000	-6.474866000	2.944101000
1	-3.613862000	-6.217832000	3.388643000
1	-5.366402000	-6.590256000	3.713620000
1	-4.454456000	-7.458369000	2.472377000
6	-3.820822000	-5.252758000	0.904059000
1	-4.097222000	-4.571653000	0.086287000
1	-2.964147000	-4.830490000	1.445754000
1	-3.522489000	-6.212605000	0.458337000
6	-6.619433000	-0.145857000	4.625128000
6	-5.542673000	0.495704000	3.992753000
6	-7.689221000	0.673474000	5.018104000
6	-5.528597000	1.872961000	3.774863000
6	-7.681047000	2.050799000	4.805504000
6	-6.597748000	2.679628000	4.179367000
1	-4.689731000	-0.090871000	3.639892000

1	-8.554064000	0.221156000	5.512086000
1	-4.667927000	2.330478000	3.277789000
1	-8.535606000	2.651915000	5.131374000
6	-8.030019000	-2.404574000	5.243884000
6	-8.309043000	-3.258230000	6.319837000
6	-9.070234000	-2.203301000	4.324011000
6	-9.549070000	-3.876555000	6.469185000
6	-10.312797000	-2.818138000	4.465565000
6	-10.577574000	-3.670450000	5.543246000
1	-7.539867000	-3.468945000	7.067744000
1	-8.913164000	-1.538510000	3.469236000
1	-9.719876000	-4.538247000	7.323991000
1	-11.096521000	-2.630566000	3.725210000
6	-6.597830000	4.161491000	3.939719000
1	-7.374714000	4.445409000	3.211018000
1	-6.810733000	4.716805000	4.866652000
1	-5.629999000	4.506133000	3.549154000
6	-11.907667000	-4.348949000	5.701329000
1	-12.333772000	-4.168283000	6.700744000
1	-12.631596000	-3.995749000	4.953308000
1	-11.812263000	-5.441214000	5.586939000
8	-2.336539000	-2.909078000	5.262087000
6	-2.697394000	-0.667067000	6.001816000
6	-0.831879000	-1.947064000	6.867705000
6	-1.253510000	0.291557000	7.685653000
1	-2.953576000	1.308082000	6.813133000
1	0.380912000	-0.959314000	8.347092000
6	-1.945206000	-1.836661000	6.033949000
6	-2.354654000	0.394501000	6.836380000
6	-0.490518000	-0.877226000	7.693092000
1	-3.547825000	-0.598917000	5.326569000
1	-0.247620000	-2.868153000	6.859005000
1	-0.982729000	1.125838000	8.336883000
6	-1.603630000	-3.089989000	4.013072000
6	-1.806208000	-2.043546000	3.049250000
6	-0.760541000	-1.509652000	2.337307000
7	0.503630000	-1.851119000	2.475193000
6	0.743967000	-2.777874000	3.379705000
6	-0.206473000	-3.393499000	4.156793000
9	-1.030745000	-0.559488000	1.442846000
9	-3.074451000	-1.622166000	2.855821000
9	-2.374933000	-4.336407000	3.628864000
9	0.129060000	-4.321516000	5.075866000
9	2.017426000	-3.129308000	3.552803000
9	-6.023606000	-5.820678000	6.045532000
6	-6.257337000	-2.153698000	2.492202000
7	-5.930236000	-2.516588000	3.772632000
6	-5.382062000	-3.739976000	3.735031000
6	-5.909331000	-3.172971000	1.665223000
1	-6.009288000	-3.264845000	0.588835000
1	-6.718138000	-1.198779000	2.263079000
7	-5.374793000	-4.163958000	2.450616000
6	-4.986863000	-5.479370000	1.864514000

83	-4.323549000	-4.695235000	5.518224000
7	-5.721840000	-1.879254000	6.312034000
6	-5.065147000	-2.939228000	6.799184000

6.16 Dimerization mechanism in C_6H_6 (Bismuth) – xyz coordinates (in Å) and Energies (in Hartree)

6.16.1 Bi(III)-F species 15-Bi

Energy(PBE0-D3(BJ)/def2-TZVPP) = -1745.88726355487

6	-1.932891000	-0.387333000	-2.382016000
7	-1.195404000	-0.367028000	-1.223360000
6	-0.253137000	-1.317297000	-1.305269000
6	-1.440088000	-1.373390000	-3.174618000
1	-1.760550000	-1.703163000	-4.156115000
1	-2.754459000	0.300334000	-2.553038000
7	-0.391417000	-1.949283000	-2.493153000
6	0.283157000	-3.185809000	-2.978796000
83	1.235504000	-1.713075000	0.391478000
7	-1.280186000	0.064447000	1.334277000
6	-0.383918000	-0.829792000	1.770681000
6	-1.603952000	-0.189965000	3.506453000
7	-0.571434000	-1.003096000	3.100308000
1	-1.956315000	-0.139853000	4.529625000
1	-2.837930000	1.200452000	2.296100000
6	-2.039581000	0.473134000	2.402307000
6	0.153563000	-1.964143000	3.979754000
5	-1.469464000	0.720939000	-0.100162000
6	1.657947000	-1.721667000	3.862684000
1	2.060337000	-2.006559000	2.880532000
1	1.903602000	-0.665148000	4.044056000
1	2.185449000	-2.338472000	4.603647000
6	-0.255591000	-1.725570000	5.430825000
1	-0.020345000	-0.702935000	5.761570000
1	-1.325001000	-1.921224000	5.593330000
1	0.306221000	-2.424450000	6.065373000
6	-0.229748000	-3.384276000	3.564413000
1	-1.305021000	-3.546376000	3.735556000
1	-0.024617000	-3.580286000	2.499667000
1	0.327949000	-4.108931000	4.176222000
6	-0.598902000	-4.370879000	-2.581750000
1	-0.680949000	-4.401572000	-1.484546000
1	-1.600548000	-4.283261000	-3.028971000
1	-0.143866000	-5.308564000	-2.934539000
6	1.660485000	-3.343881000	-2.344599000
1	2.258061000	-2.421637000	-2.415260000
1	1.560706000	-3.685283000	-1.304811000
1	2.205156000	-4.133335000	-2.881011000
6	0.443090000	-3.090426000	-4.495782000
1	-0.521067000	-3.093606000	-5.022387000
1	0.999970000	-2.185132000	-4.780820000
1	1.005008000	-3.967053000	-4.846216000
6	-0.490814000	1.988317000	-0.283225000
6	-0.080865000	2.413334000	-1.553925000
6	-0.093676000	2.782963000	0.798439000

6	0.685516000	3.562014000	-1.736146000
6	0.673473000	3.933931000	0.625776000
6	1.082918000	4.345880000	-0.646601000
1	-0.361414000	1.830153000	-2.436642000
1	-0.382071000	2.499069000	1.815320000
1	0.986524000	3.856698000	-2.746609000
1	0.965562000	4.525197000	1.499423000
6	-3.040004000	1.129826000	-0.213714000
6	-4.046408000	0.157680000	-0.063143000
6	-3.466155000	2.438950000	-0.456326000
6	-5.396646000	0.475713000	-0.150742000
6	-4.822324000	2.766979000	-0.545641000
6	-5.812671000	1.793945000	-0.394883000
1	-3.762379000	-0.882752000	0.128969000
1	-2.718772000	3.227825000	-0.578969000
1	-6.148648000	-0.310300000	-0.028271000
1	-5.115632000	3.803776000	-0.736991000
6	1.942763000	5.562237000	-0.837049000
1	1.679655000	6.103313000	-1.758694000
1	1.849841000	6.259074000	0.008972000
1	3.007862000	5.285706000	-0.917985000
6	-7.272157000	2.134234000	-0.485637000
1	-7.793565000	1.910121000	0.459132000
1	-7.423751000	3.199497000	-0.710292000
1	-7.771285000	1.546336000	-1.272610000
9	0.128249000	-3.648392000	0.450551000
9	1.897066000	0.293654000	0.081541000

6.16.2 **Bi(III) diaryl species 16-Bi**

Energy(PBE0-D3(BJ)/def2-TZVPP) = -2834.75612778051

6	1.880815000	0.293731000	2.708770000
7	1.236539000	-0.083769000	1.557750000
6	0.160869000	-0.808841000	1.892339000
6	1.191599000	-0.217726000	3.760005000
1	1.383925000	-0.145269000	4.825190000
1	2.783908000	0.894558000	2.690573000
7	0.119912000	-0.907981000	3.240365000
6	-0.817702000	-1.635653000	4.140007000
83	-1.233370000	-1.733084000	0.335249000
7	1.528808000	-0.719158000	-0.941577000
6	0.441919000	-1.459590000	-1.196515000
6	1.894761000	-1.797150000	-2.832100000
7	0.647126000	-2.127726000	-2.354086000
1	2.299177000	-2.201442000	-3.753959000
1	3.397130000	-0.414192000	-1.966510000
6	2.436843000	-0.918607000	-1.950487000
6	-0.230588000	-3.069284000	-3.102582000
5	1.740986000	0.420597000	0.141433000
6	-0.358253000	-2.543770000	-4.534766000
1	-0.782932000	-1.529009000	-4.531580000
1	0.607281000	-2.519003000	-5.057960000
1	-1.028428000	-3.202627000	-5.104577000
6	0.421126000	-4.451394000	-3.067047000
1	1.410552000	-4.443554000	-3.546291000
1	0.539522000	-4.794140000	-2.028483000
1	-0.211257000	-5.173752000	-3.602919000
6	-1.624337000	-3.140579000	-2.497032000
1	-1.636024000	-3.664669000	-1.530729000
1	-2.080888000	-2.142203000	-2.418872000
1	-2.259802000	-3.734134000	-3.168486000
6	-1.990219000	-2.233491000	3.378942000
1	-2.574075000	-1.459825000	2.859468000
1	-1.671296000	-3.029456000	2.689609000
1	-2.663614000	-2.707273000	4.106468000
6	-1.359662000	-0.634455000	5.162084000
1	-0.565604000	-0.216823000	5.796078000
1	-1.873598000	0.192552000	4.651077000
1	-2.079180000	-1.141464000	5.820317000
6	-0.031548000	-2.760830000	4.815077000
1	0.367490000	-3.454475000	4.059873000
1	0.807768000	-2.371503000	5.408606000
1	-0.693894000	-3.321930000	5.489692000
6	0.916246000	1.730500000	-0.312787000
6	0.340532000	2.609334000	0.614249000
6	0.817131000	2.091097000	-1.663229000
6	-0.321134000	3.768067000	0.216618000
6	0.156439000	3.247957000	-2.070510000
6	-0.435631000	4.106416000	-1.137466000
1	0.384942000	2.375003000	1.682503000

1	1.252641000	1.444674000	-2.431069000
1	-0.776661000	4.417206000	0.970770000
1	0.081759000	3.483806000	-3.136193000
6	3.335559000	0.702635000	0.261251000
6	4.232668000	-0.321001000	0.622160000
6	3.889808000	1.964190000	0.021128000
6	5.599958000	-0.093701000	0.732377000
6	5.263105000	2.199288000	0.131532000
6	6.144451000	1.176452000	0.488180000
1	3.849924000	-1.325836000	0.820968000
1	3.231958000	2.790873000	-0.260638000
1	6.263862000	-0.917022000	1.014172000
1	5.657061000	3.201262000	-0.064118000
6	-1.213342000	5.314393000	-1.566475000
1	-1.008499000	6.178677000	-0.916667000
1	-0.982952000	5.596893000	-2.603629000
1	-2.296430000	5.113415000	-1.506437000
6	7.621444000	1.416088000	0.609218000
1	8.188292000	0.769973000	-0.080320000
1	7.879452000	2.460536000	0.383993000
1	7.980205000	1.189482000	1.626213000
6	-2.092708000	0.533382000	-0.134598000
6	-2.703194000	1.250362000	0.876231000
6	-2.268824000	1.049143000	-1.403008000
6	-3.423494000	2.407726000	0.601390000
6	-2.991494000	2.224121000	-1.595876000
7	-3.562365000	2.876232000	-0.614137000
9	-2.604058000	0.845835000	2.159643000
9	-1.746205000	0.437996000	-2.479229000
9	-3.995957000	3.086353000	1.588587000
9	-3.115493000	2.729134000	-2.817098000
6	0.112781000	-3.886904000	0.819366000
6	-0.591088000	-5.073569000	0.748506000
6	1.466347000	-4.041227000	1.048515000
6	0.041519000	-6.306871000	0.889709000
6	2.034643000	-5.308305000	1.178318000
7	1.330647000	-6.410448000	1.097909000
9	2.281160000	-2.978491000	1.154819000
9	3.338699000	-5.431557000	1.392204000
9	-0.659883000	-7.431053000	0.817236000
9	-1.922770000	-5.063770000	0.526980000

6.16.3 **INT11-Bi**

Energy(PBE0-D3(BJ)/def2-TZVPP) = -2834.73970428393

6	1.714645000	0.285718000	2.532665000
7	1.069562000	-0.068959000	1.369513000
6	0.023887000	-0.869735000	1.657958000
6	1.070912000	-0.319908000	3.560638000
1	1.276673000	-0.298134000	4.626165000
1	2.586118000	0.932450000	2.539414000
7	0.034428000	-1.041548000	3.006549000
6	-0.837571000	-1.883690000	3.860918000
83	-1.600031000	-1.531861000	-0.149538000
7	1.308881000	-0.545073000	-1.170953000
6	0.405720000	-1.532110000	-1.293878000
6	1.930568000	-1.801446000	-2.879034000
7	0.804056000	-2.343448000	-2.307552000
1	2.444870000	-2.260666000	-3.716905000
1	3.074019000	-0.002027000	-2.281844000
6	2.239822000	-0.686588000	-2.170508000
6	0.376413000	-3.725477000	-2.676007000
5	1.541530000	0.533132000	-0.009089000
6	0.070170000	-3.775242000	-4.172553000
1	-0.709023000	-3.053231000	-4.442391000
1	0.962012000	-3.556196000	-4.776900000
1	-0.272196000	-4.785052000	-4.438671000
6	1.542246000	-4.660410000	-2.334950000
1	2.444667000	-4.421203000	-2.914462000
1	1.788541000	-4.595863000	-1.264448000
1	1.258179000	-5.697437000	-2.564638000
6	-0.839290000	-4.174004000	-1.881727000
1	-0.636334000	-4.182927000	-0.799969000
1	-1.724467000	-3.563055000	-2.103732000
1	-1.078183000	-5.206634000	-2.170549000
6	-1.870468000	-2.629696000	3.032429000
1	-2.564205000	-1.940181000	2.534486000
1	-1.395109000	-3.283202000	2.286645000
1	-2.463853000	-3.265512000	3.704203000
6	-1.547413000	-0.976431000	4.867906000
1	-0.830599000	-0.461930000	5.524011000
1	-2.146724000	-0.217884000	4.345855000
1	-2.214911000	-1.575837000	5.503887000
6	0.046949000	-2.906701000	4.579202000
1	0.581378000	-3.533733000	3.849692000
1	0.790028000	-2.423338000	5.229044000
1	-0.576397000	-3.558698000	5.208170000
6	0.809476000	1.934558000	-0.311613000
6	0.272800000	2.725301000	0.714911000
6	0.810805000	2.493149000	-1.596094000
6	-0.244100000	3.994724000	0.472352000
6	0.291365000	3.761760000	-1.848844000
6	-0.250302000	4.537559000	-0.818686000
1	0.247720000	2.338868000	1.738059000

1	1.214457000	1.918955000	-2.435310000
1	-0.664563000	4.575694000	1.298859000
1	0.294683000	4.155262000	-2.869601000
6	3.159568000	0.717332000	0.100296000
6	3.977899000	-0.385148000	0.409586000
6	3.806932000	1.941829000	-0.085829000
6	5.358351000	-0.272015000	0.519382000
6	5.195950000	2.064484000	0.022309000
6	5.998001000	0.962564000	0.325039000
1	3.516199000	-1.365613000	0.570110000
1	3.213818000	2.829426000	-0.322095000
1	5.958687000	-1.154878000	0.761069000
1	5.664814000	3.041197000	-0.131398000
6	-0.866113000	5.878744000	-1.085990000
1	-0.617764000	6.602712000	-0.295369000
1	-0.535519000	6.289899000	-2.050560000
1	-1.965198000	5.796626000	-1.120391000
6	7.489944000	1.081841000	0.442269000
1	8.001011000	0.433976000	-0.288161000
1	7.825453000	2.114452000	0.271051000
1	7.838306000	0.773368000	1.441187000
6	-2.207773000	0.690687000	-0.148360000
6	-2.809042000	1.150748000	1.012435000
6	-2.250494000	1.551914000	-1.235147000
6	-3.399157000	2.409974000	1.050372000
6	-2.887735000	2.786089000	-1.105048000
7	-3.441324000	3.196089000	0.006877000
9	-2.849391000	0.385072000	2.111118000
9	-1.707488000	1.244874000	-2.401570000
9	-3.953946000	2.838980000	2.173137000
9	-2.954374000	3.588885000	-2.154485000
6	-2.806398000	-1.460810000	-2.357902000
6	-4.143358000	-1.790588000	-2.228544000
6	-2.404924000	-1.188652000	-3.653429000
6	-4.987192000	-1.851739000	-3.335582000
6	-3.304253000	-1.290076000	-4.712954000
7	-4.563841000	-1.610933000	-4.550736000
9	-1.143240000	-0.843778000	-3.943192000
9	-2.884145000	-1.068774000	-5.952565000
9	-6.265564000	-2.170226000	-3.181448000
9	-4.657377000	-2.073680000	-1.016074000

6.16.4 **TS12-Bi**

Energy(PBE0-D3(BJ)/def2-TZVPP) = -2834.7007013768

6	1.927319000	0.195619000	2.715231000
7	1.427176000	-0.231096000	1.512599000
6	0.285507000	-0.900918000	1.732488000
6	1.084305000	-0.231575000	3.690332000
1	1.131501000	-0.089936000	4.764474000
1	2.842452000	0.774547000	2.784753000
7	0.070932000	-0.926617000	3.072846000
6	-1.062413000	-1.481347000	3.868350000
83	-0.914865000	-1.894495000	0.012055000
7	1.923528000	-0.753168000	-0.995989000
6	0.916145000	-1.570136000	-1.338043000
6	2.426123000	-1.634174000	-2.956134000
7	1.215572000	-2.131908000	-2.534371000
1	2.883138000	-1.920441000	-3.897373000
1	3.776157000	-0.196496000	-1.943198000
6	2.864100000	-0.781996000	-1.994751000
6	0.447640000	-3.072190000	-3.400982000
5	2.026321000	0.331661000	0.162351000
6	0.056744000	-2.325731000	-4.677756000
1	-0.593867000	-1.473375000	-4.439898000
1	0.939309000	-1.957497000	-5.219932000
1	-0.489780000	-3.008257000	-5.344302000
6	1.349140000	-4.269244000	-3.709139000
1	2.249034000	-3.980005000	-4.269492000
1	1.661707000	-4.769109000	-2.780126000
1	0.793908000	-4.992567000	-4.322657000
6	-0.810336000	-3.573875000	-2.716223000
1	-0.588187000	-4.129678000	-1.792999000
1	-1.529738000	-2.764340000	-2.515769000
1	-1.318756000	-4.269489000	-3.397882000
6	-1.902229000	-2.448368000	3.050430000
1	-2.470268000	-1.945965000	2.256630000
1	-1.297685000	-3.269957000	2.638528000
1	-2.653350000	-2.897101000	3.714862000
6	-1.924828000	-0.305539000	4.329605000
1	-1.344770000	0.398897000	4.943500000
1	-2.337089000	0.231892000	3.464285000
1	-2.762408000	-0.678680000	4.936584000
6	-0.480699000	-2.240507000	5.063069000
1	0.204005000	-3.033419000	4.727015000
1	0.057989000	-1.583187000	5.758976000
1	-1.302545000	-2.707026000	5.623682000
6	1.192762000	1.646435000	-0.263638000
6	0.305133000	2.300099000	0.602238000
6	1.386988000	2.237248000	-1.520710000
6	-0.354279000	3.470996000	0.235287000
6	0.735232000	3.409692000	-1.892626000
6	-0.153954000	4.049012000	-1.021908000
1	0.109846000	1.891477000	1.598230000

1	2.064859000	1.769592000	-2.241180000
1	-1.054915000	3.938227000	0.933638000
1	0.904097000	3.831069000	-2.887709000
6	3.604448000	0.602404000	0.419686000
6	4.457796000	-0.461116000	0.766820000
6	4.185918000	1.871182000	0.337944000
6	5.812593000	-0.268829000	1.011084000
6	5.547415000	2.072762000	0.582387000
6	6.386593000	1.008828000	0.921304000
1	4.047494000	-1.473761000	0.847434000
1	3.559724000	2.728528000	0.076125000
1	6.443324000	-1.122797000	1.277447000
1	5.965594000	3.081098000	0.507878000
6	-0.923098000	5.265385000	-1.440256000
1	-0.970097000	6.012692000	-0.633575000
1	-0.481804000	5.738946000	-2.328881000
1	-1.959998000	4.982500000	-1.686257000
6	7.851638000	1.211461000	1.178412000
1	8.463845000	0.661234000	0.445501000
1	8.128036000	2.273530000	1.118557000
1	8.137157000	0.840280000	2.175578000
6	-2.180596000	-0.154780000	-0.830915000
6	-2.936333000	0.591592000	0.113484000
6	-1.714394000	0.682441000	-1.881684000
6	-3.415220000	1.848282000	-0.180087000
6	-2.276497000	1.919610000	-2.095414000
7	-3.130203000	2.508483000	-1.280278000
9	-3.287112000	-0.001779000	1.277296000
9	-0.840617000	0.190366000	-2.775328000
9	-4.192282000	2.461109000	0.710952000
9	-1.934893000	2.594917000	-3.189353000
6	-3.461248000	-1.488980000	-1.599973000
6	-4.321153000	-2.182521000	-0.764728000
6	-3.974931000	-1.223614000	-2.860020000
6	-5.601773000	-2.534293000	-1.176546000
6	-5.272194000	-1.607353000	-3.194774000
7	-6.065397000	-2.240981000	-2.366238000
9	-3.237256000	-0.616780000	-3.799159000
9	-5.741615000	-1.346202000	-4.407481000
9	-6.399124000	-3.209712000	-0.358082000
9	-3.924291000	-2.574258000	0.466382000

6.17 Dimerization mechanism in CH_3CN (Bismuth) – xyz coordinates (in Å) and Energies (in Hartree)

6.17.1 **Bismuthinidene 3**

Energy(PBE0-D3(BJ)/def2-TZVPP) = -1546.2490304319

7	0.442998000	-1.286639000	-0.586769000
6	-0.864854000	-1.558559000	-0.420017000
6	0.145829000	-3.401845000	-1.158515000
1	0.257838000	-4.431159000	-1.478431000
1	2.142559000	-2.445554000	-1.241340000
7	-1.061381000	-2.858129000	-0.767855000
6	-2.358535000	-3.587903000	-0.787825000
83	-2.366906000	0.000111000	0.284042000
7	0.443145000	1.286974000	-0.586300000
6	-0.864697000	1.558924000	-0.419530000
6	0.146158000	3.402393000	-1.157347000
7	-1.061115000	2.858623000	-0.766951000
1	0.258259000	4.431804000	-1.476926000
1	2.142833000	2.446008000	-1.240339000
6	1.076106000	2.420049000	-1.043200000
6	-2.358225000	3.588475000	-0.786796000
5	1.210014000	0.000039000	-0.092802000
6	-3.048309000	3.429802000	0.566610000
1	-3.374407000	2.398192000	0.764297000
1	-2.382670000	3.746444000	1.382808000
1	-3.952324000	4.054771000	0.588363000
6	-2.102313000	5.075763000	-1.017852000
1	-1.457721000	5.504110000	-0.236101000
1	-1.653600000	5.270848000	-2.002197000
1	-3.068375000	5.597673000	-0.984981000
6	-3.214401000	3.039238000	-1.926775000
1	-2.711123000	3.196155000	-2.892513000
1	-3.400134000	1.962076000	-1.798493000
1	-4.182769000	3.559824000	-1.947626000
6	-3.214748000	-3.038285000	-1.927592000
1	-3.400388000	-1.961143000	-1.798982000
1	-2.711555000	-3.194961000	-2.893412000
1	-4.183158000	-3.558791000	-1.948522000
6	-3.048540000	-3.429552000	0.565661000
1	-2.382864000	-3.746412000	1.381745000
1	-3.374617000	-2.397987000	0.763620000
1	-3.952566000	-4.054509000	0.587304000
6	-2.102735000	-5.075153000	-1.019255000
1	-1.654134000	-5.270045000	-2.003688000
1	-1.458089000	-5.503708000	-0.237664000
1	-3.068825000	-5.597014000	-0.986410000
6	1.253751000	-0.000272000	1.527639000
6	1.315357000	-1.192210000	2.264294000
6	1.315849000	1.191299000	2.264824000
6	1.419033000	-1.197654000	3.655300000

6	1.419534000	1.196068000	3.655845000
6	1.467453000	-0.000961000	4.381451000
1	1.274921000	-2.152250000	1.738919000
1	1.275815000	2.151597000	1.739892000
1	1.461564000	-2.151057000	4.191747000
1	1.462459000	2.149207000	4.192727000
6	2.689881000	0.000056000	-0.760544000
6	2.849676000	0.000069000	-2.159233000
6	3.864786000	0.000032000	-0.001297000
6	4.104434000	0.000083000	-2.759904000
6	5.130448000	0.000051000	-0.595768000
6	5.275452000	0.000084000	-1.985488000
1	1.961718000	0.000074000	-2.800696000
1	3.793717000	0.000001000	1.090287000
1	4.184036000	0.000092000	-3.851713000
1	6.025013000	0.000036000	0.034907000
6	1.539568000	-0.001353000	5.881603000
1	2.060803000	-0.893209000	6.259851000
1	2.058823000	0.891411000	6.260409000
1	0.529871000	-0.002654000	6.326788000
6	6.628254000	0.000151000	-2.636890000
1	6.760473000	-0.884081000	-3.281126000
1	6.760725000	0.884891000	-3.280384000
1	7.434260000	-0.000261000	-1.889500000
7	0.442998000	-1.286639000	-0.586769000

6.17.2 **TS1-Bi**

Energy(PBE0-D3(BJ)/def2-TZVPP) = -2290.280486086226

6	2.237708000	-0.368269000	2.423699000
7	1.461145000	-0.485895000	1.297054000
6	0.352111000	-1.171863000	1.614539000
6	1.599622000	-1.003558000	3.441698000
1	1.897667000	-1.129073000	4.475910000
1	3.187968000	0.155497000	2.419408000
7	0.428119000	-1.509696000	2.926425000
6	-0.531603000	-2.359467000	3.691284000
83	-1.301729000	-1.643234000	0.114866000
7	1.445933000	-0.707332000	-1.280501000
6	0.283972000	-1.341923000	-1.499783000
6	1.513339000	-1.503076000	-3.340673000
7	0.312509000	-1.842492000	-2.760644000
1	1.783270000	-1.777819000	-4.353606000
1	3.194941000	-0.338038000	-2.485693000
6	2.213309000	-0.794763000	-2.415501000
6	-0.724246000	-2.692923000	-3.413418000
5	1.833114000	0.225828000	-0.067537000
6	-2.090762000	-2.032835000	-3.259931000
1	-2.465670000	-2.046627000	-2.227513000
1	-2.067331000	-0.989834000	-3.605582000
1	-2.827080000	-2.585643000	-3.859871000
6	-0.414284000	-2.811377000	-4.903230000
1	-0.386052000	-1.825116000	-5.389530000
1	0.534183000	-3.334124000	-5.090044000
1	-1.214305000	-3.399927000	-5.372233000
6	-0.689013000	-4.079885000	-2.773142000
1	0.300796000	-4.540727000	-2.907173000
1	-0.908161000	-4.033316000	-1.695654000
1	-1.443011000	-4.724374000	-3.247492000
6	-0.520030000	-3.766677000	3.096299000
1	-0.850424000	-3.771075000	2.047108000
1	0.491885000	-4.195567000	3.143627000
1	-1.201811000	-4.411862000	3.668538000
6	-1.919022000	-1.727958000	3.640794000
1	-1.897658000	-0.710014000	4.053041000
1	-2.335355000	-1.666255000	2.626899000
1	-2.614055000	-2.337261000	4.235548000
6	-0.089838000	-2.433839000	5.149990000
1	0.893896000	-2.912229000	5.259032000
1	-0.063547000	-1.439603000	5.619331000
1	-0.820801000	-3.045504000	5.695980000
6	1.040112000	1.626015000	-0.199439000
6	0.997360000	2.523235000	0.878068000
6	0.505034000	2.079287000	-1.414082000
6	0.476322000	3.808380000	0.745726000
6	-0.031564000	3.358904000	-1.549452000
6	-0.049568000	4.252707000	-0.472889000
1	1.398137000	2.218555000	1.850361000

1	0.511490000	1.421628000	-2.289113000
1	0.465293000	4.480757000	1.608470000
1	-0.447866000	3.671891000	-2.512184000
6	3.446157000	0.417760000	-0.099782000
6	4.298757000	-0.692640000	0.033664000
6	4.064083000	1.658822000	-0.292937000
6	5.684506000	-0.570184000	-0.016540000
6	5.453685000	1.791160000	-0.345447000
6	6.291612000	0.680182000	-0.204238000
1	3.865333000	-1.688398000	0.176873000
1	3.443465000	2.551600000	-0.411065000
1	6.312690000	-1.460392000	0.088107000
1	5.897010000	2.779526000	-0.502596000
6	-0.664366000	5.613666000	-0.609634000
1	-0.375835000	6.272886000	0.221647000
1	-0.373797000	6.097023000	-1.554953000
1	-1.763544000	5.530706000	-0.607766000
6	7.786025000	0.822120000	-0.227363000
1	8.100853000	1.663288000	-0.862284000
1	8.178884000	1.014600000	0.785537000
1	8.271747000	-0.093655000	-0.595074000
6	-2.972106000	0.455182000	0.421648000
6	-2.545947000	1.540856000	1.236119000
6	-3.526731000	0.873125000	-0.819057000
6	-2.709109000	2.839323000	0.807943000
6	-3.579943000	2.206655000	-1.151077000
7	-3.223329000	3.192471000	-0.352829000
9	-1.964124000	1.282600000	2.415012000
9	-3.937523000	-0.064803000	-1.685194000
9	-2.359057000	3.826659000	1.629580000
9	-4.053724000	2.548280000	-2.349132000
9	-3.709940000	-0.535013000	1.090210000

6.17.3 **INT1-Bi**

Energy(PBE0-D3(BJ)/def2-TZVPP) = -2290.33632741359

7	1.149715000	-0.096448000	1.490744000
6	0.081748000	-0.858791000	1.794931000
6	1.073871000	-0.213991000	3.691700000
1	1.244676000	-0.129797000	4.760459000
1	2.645952000	0.946951000	2.650293000
7	0.040748000	-0.947801000	3.150649000
6	-0.890357000	-1.691033000	4.040181000
83	-1.430299000	-1.736266000	0.067834000
7	1.428279000	-0.650256000	-1.038159000
6	0.457485000	-1.556218000	-1.248894000
6	1.913751000	-1.715170000	-2.912171000
7	0.762881000	-2.243032000	-2.379149000
1	2.364109000	-2.081762000	-3.827385000
1	3.204665000	-0.097446000	-2.128760000
6	2.325606000	-0.730778000	-2.073794000
6	0.048750000	-3.377754000	-3.043706000
5	1.648363000	0.441870000	0.100004000
6	-0.882409000	-2.791670000	-4.104094000
1	-1.654822000	-2.186374000	-3.609057000
1	-0.318209000	-2.179508000	-4.823893000
1	-1.369785000	-3.610218000	-4.654679000
6	1.091699000	-4.296152000	-3.684309000
1	1.616074000	-3.823782000	-4.526166000
1	1.833240000	-4.634539000	-2.945075000
1	0.572988000	-5.179445000	-4.081150000
6	-0.747129000	-4.188219000	-2.025820000
1	-0.141315000	-4.442678000	-1.142389000
1	-1.664574000	-3.651637000	-1.749481000
1	-1.059113000	-5.129418000	-2.499567000
6	-1.910845000	-2.488254000	3.245604000
1	-2.574576000	-1.836726000	2.660151000
1	-1.429884000	-3.229790000	2.591507000
1	-2.544858000	-3.037260000	3.955625000
6	-1.615706000	-0.675213000	4.925250000
1	-0.912680000	-0.113189000	5.556609000
1	-2.179765000	0.037868000	4.307673000
1	-2.318795000	-1.201251000	5.586999000
6	-0.063452000	-2.661291000	4.887660000
1	0.487615000	-3.362173000	4.242731000
1	0.656442000	-2.135339000	5.530389000
1	-0.735042000	-3.240259000	5.537554000
6	0.888342000	1.815560000	-0.284050000
6	0.232271000	2.600180000	0.674702000
6	0.937158000	2.333664000	-1.586205000
6	-0.356548000	3.821240000	0.351704000
6	0.349098000	3.552392000	-1.919523000
6	-0.317398000	4.318735000	-0.956288000
1	0.161486000	2.245044000	1.707261000
1	1.437056000	1.765860000	-2.376524000

1	-0.873270000	4.394753000	1.127641000
1	0.395952000	3.913446000	-2.951482000
6	3.259980000	0.648804000	0.231230000
6	4.085082000	-0.442278000	0.562653000
6	3.897304000	1.879398000	0.048571000
6	5.462541000	-0.311888000	0.697469000
6	5.282790000	2.020483000	0.182484000
6	6.091958000	0.929206000	0.507506000
1	3.632379000	-1.427422000	0.719875000
1	3.299204000	2.758890000	-0.206299000
1	6.068512000	-1.186296000	0.955442000
1	5.743765000	3.001569000	0.031390000
6	-1.020398000	5.592729000	-1.319747000
1	-0.946635000	6.340657000	-0.516146000
1	-0.615061000	6.030307000	-2.243635000
1	-2.094101000	5.401966000	-1.487995000
6	7.580306000	1.066129000	0.649516000
1	8.109695000	0.437954000	-0.085206000
1	7.904015000	2.106222000	0.502073000
1	7.917694000	0.741390000	1.646919000
6	-2.115958000	0.440981000	-0.210473000
6	-2.826013000	1.055902000	0.804712000
6	-2.081719000	1.108568000	-1.424649000
6	-3.451659000	2.278617000	0.582497000
6	-2.741030000	2.329039000	-1.549346000
7	-3.411682000	2.888462000	-0.573276000
9	-2.931980000	0.484479000	2.010757000
9	-1.428889000	0.629964000	-2.476379000
9	-4.124060000	2.862552000	1.564772000
9	-2.711570000	2.968745000	-2.710568000
9	-2.694387000	-1.859903000	-1.779274000
7	1.149715000	-0.096448000	1.490744000

6.17.4 **INT2-Bi**

Energy(PBE0-D3(BJ)/def2-TZVPP) = -2290.34166537108

6	-1.943888000	-0.354935000	-2.395515000
7	-1.244745000	-0.398507000	-1.212333000
6	-0.330588000	-1.377186000	-1.298026000
6	-1.459554000	-1.331277000	-3.203453000
1	-1.755878000	-1.621551000	-4.206527000
1	-2.735973000	0.365224000	-2.572021000
7	-0.460533000	-1.971185000	-2.505370000
6	0.206124000	-3.177584000	-3.076860000
83	1.072709000	-1.918071000	0.423253000
7	-1.313122000	0.040645000	1.353102000
6	-0.367471000	-0.796536000	1.797692000
6	-1.558031000	-0.140015000	3.543745000
7	-0.506487000	-0.926559000	3.136635000
1	-1.879613000	-0.062490000	4.575737000
1	-2.886207000	1.150952000	2.325754000
6	-2.057002000	0.458769000	2.429818000
6	0.273358000	-1.828002000	4.037030000
5	-1.529571000	0.676992000	-0.083541000
6	1.769029000	-1.637995000	3.784671000
1	2.107836000	-2.078558000	2.835599000
1	2.039973000	-0.573853000	3.795313000
1	2.331915000	-2.156295000	4.573101000
6	-0.009921000	-1.452571000	5.489139000
1	0.261950000	-0.407479000	5.699139000
1	-1.061941000	-1.615395000	5.761828000
1	0.600528000	-2.100374000	6.132849000
6	-0.175048000	-3.266703000	3.778770000
1	-1.217546000	-3.398666000	4.107094000
1	-0.126607000	-3.533644000	2.711292000
1	0.457238000	-3.957386000	4.355775000
6	-0.869743000	-4.254096000	-3.234958000
1	-1.312168000	-4.482852000	-2.253769000
1	-1.668141000	-3.937092000	-3.920791000
1	-0.415527000	-5.170292000	-3.638388000
6	1.293027000	-3.710702000	-2.158618000
1	2.101656000	-2.978654000	-2.011912000
1	0.864099000	-4.041544000	-1.200750000
1	1.744758000	-4.589870000	-2.638658000
6	0.818999000	-2.786717000	-4.420908000
1	0.057221000	-2.460243000	-5.142659000
1	1.550127000	-1.976032000	-4.287222000
1	1.335508000	-3.657708000	-4.848383000
6	-0.554851000	1.952494000	-0.266570000
6	0.062750000	2.257267000	-1.487521000
6	-0.344163000	2.858885000	0.782033000
6	0.863383000	3.386071000	-1.647457000
6	0.456110000	3.990160000	0.632163000
6	1.084918000	4.271907000	-0.585875000
1	-0.062318000	1.584687000	-2.341646000

1	-0.802375000	2.674541000	1.758240000
1	1.344044000	3.575343000	-2.612387000
1	0.609114000	4.662472000	1.481930000
6	-3.103478000	1.067188000	-0.192080000
6	-4.092529000	0.078220000	-0.036144000
6	-3.552547000	2.365621000	-0.450296000
6	-5.448357000	0.371284000	-0.130822000
6	-4.914252000	2.669352000	-0.547672000
6	-5.887800000	1.679774000	-0.389616000
1	-3.790912000	-0.955569000	0.165182000
1	-2.820895000	3.168319000	-0.580322000
1	-6.186722000	-0.426768000	-0.003404000
1	-5.225937000	3.698425000	-0.751896000
6	2.010072000	5.442475000	-0.738151000
1	1.883513000	5.935870000	-1.713731000
1	1.852829000	6.189118000	0.053576000
1	3.060493000	5.110418000	-0.678121000
6	-7.352684000	1.992978000	-0.488977000
1	-7.872167000	1.770060000	0.457106000
1	-7.521775000	3.052550000	-0.727550000
1	-7.838068000	1.384710000	-1.268966000
6	2.211613000	0.201818000	-0.058916000
6	2.498000000	1.215243000	0.836135000
6	2.835329000	0.319417000	-1.287216000
6	3.345480000	2.259644000	0.474494000
6	3.674519000	1.391435000	-1.568708000
7	3.925532000	2.337002000	-0.697552000
9	2.639718000	-0.605895000	-2.246224000
9	1.972395000	1.231712000	2.068299000
9	3.595519000	3.235151000	1.341411000
9	4.255449000	1.488777000	-2.760091000
9	-0.391845000	-3.623667000	0.592566000

6.17.5 **Bi(III)-F species 15-Bi**

Energy(PBE0-D3(BJ)/def2-TZVPP) = -1745.90593593033

6	-1.935088000	-0.385794000	-2.387470000
7	-1.209118000	-0.380665000	-1.220635000
6	-0.268632000	-1.334150000	-1.304750000
6	-1.436958000	-1.364273000	-3.186732000
1	-1.749363000	-1.682979000	-4.175043000
1	-2.752527000	0.305794000	-2.562565000
7	-0.397430000	-1.950433000	-2.501800000
6	0.286791000	-3.177138000	-3.004349000
83	1.197960000	-1.775058000	0.400194000
7	-1.292997000	0.052000000	1.338750000
6	-0.391886000	-0.837816000	1.777873000
6	-1.598712000	-0.182194000	3.516744000
7	-0.568981000	-0.996910000	3.110625000
1	-1.943442000	-0.122513000	4.542534000
1	-2.844103000	1.196297000	2.306684000
6	-2.044669000	0.469658000	2.409496000
6	0.167006000	-1.943507000	4.000398000
5	-1.482839000	0.703224000	-0.095666000
6	1.670125000	-1.715091000	3.851119000
1	2.057821000	-2.034704000	2.873360000
1	1.924551000	-0.655559000	3.999880000
1	2.202734000	-2.313686000	4.602996000
6	-0.216028000	-1.671212000	5.451997000
1	0.027514000	-0.641714000	5.753775000
1	-1.282649000	-1.860432000	5.637743000
1	0.357546000	-2.357447000	6.089782000
6	-0.234225000	-3.368927000	3.622757000
1	-1.306667000	-3.519915000	3.819941000
1	-0.050047000	-3.584943000	2.558989000
1	0.333037000	-4.083093000	4.237623000
6	-0.601113000	-4.369777000	-2.646851000
1	-0.705915000	-4.421804000	-1.552996000
1	-1.593658000	-4.274819000	-3.112189000
1	-0.136518000	-5.298947000	-3.008910000
6	1.655007000	-3.346621000	-2.355272000
1	2.255487000	-2.424953000	-2.403422000
1	1.551311000	-3.706694000	-1.322185000
1	2.204247000	-4.128219000	-2.898186000
6	0.467553000	-3.050663000	-4.516350000
1	-0.489669000	-3.041967000	-5.055081000
1	1.030112000	-2.140591000	-4.773336000
1	1.034130000	-3.921254000	-4.874038000
6	-0.499113000	1.970464000	-0.278221000
6	-0.068309000	2.385281000	-1.546533000
6	-0.117794000	2.775911000	0.802749000
6	0.703510000	3.531891000	-1.727185000
6	0.655452000	3.924656000	0.632117000
6	1.086461000	4.325213000	-0.637945000
1	-0.338195000	1.796501000	-2.429100000

1	-0.426200000	2.504299000	1.817381000
1	1.018619000	3.818557000	-2.735793000
1	0.932497000	4.524686000	1.504863000
6	-3.051048000	1.120439000	-0.211296000
6	-4.060144000	0.150949000	-0.059961000
6	-3.475225000	2.429721000	-0.457460000
6	-5.410009000	0.471608000	-0.149635000
6	-4.830852000	2.761571000	-0.549209000
6	-5.823810000	1.790573000	-0.397194000
1	-3.779356000	-0.889880000	0.134810000
1	-2.727844000	3.218619000	-0.581694000
1	-6.163814000	-0.312541000	-0.026208000
1	-5.122142000	3.798545000	-0.743396000
6	1.948563000	5.540522000	-0.825888000
1	1.701750000	6.070139000	-1.758486000
1	1.840446000	6.245162000	0.011693000
1	3.015223000	5.264445000	-0.885608000
6	-7.282391000	2.133667000	-0.490146000
1	-7.804333000	1.911604000	0.454789000
1	-7.431159000	3.198714000	-0.717687000
1	-7.781446000	1.543141000	-1.275151000
9	0.024559000	-3.673879000	0.464479000
9	1.946419000	0.219445000	0.071146000

6.17.6 **Bi(III) diaryl species 16-Bi**

Energy(PBE0-D3(BJ)/def2-TZVPP) = -2834.77043437336

6	1.866689000	0.305990000	2.702783000
7	1.228852000	-0.079623000	1.549657000
6	0.158340000	-0.814186000	1.884597000
6	1.180094000	-0.210538000	3.753622000
1	1.370085000	-0.135911000	4.819617000
1	2.762866000	0.917211000	2.689801000
7	0.116000000	-0.910395000	3.232413000
6	-0.816399000	-1.647827000	4.133199000
83	-1.235008000	-1.747231000	0.329907000
7	1.520212000	-0.708573000	-0.952689000
6	0.438954000	-1.460985000	-1.202099000
6	1.885538000	-1.784137000	-2.846511000
7	0.645058000	-2.127142000	-2.360330000
1	2.288807000	-2.185323000	-3.770847000
1	3.379225000	-0.386133000	-1.994916000
6	2.423937000	-0.899470000	-1.968314000
6	-0.227350000	-3.080956000	-3.103907000
5	1.731549000	0.425239000	0.134470000
6	-0.364802000	-2.562208000	-4.537171000
1	-0.800966000	-1.552230000	-4.536900000
1	0.599063000	-2.531607000	-5.063029000
1	-1.031347000	-3.231617000	-5.098721000
6	0.439802000	-4.455408000	-3.068281000
1	1.426659000	-4.436010000	-3.552589000
1	0.563801000	-4.796233000	-2.029559000
1	-0.188941000	-5.182306000	-3.602171000
6	-1.616910000	-3.165167000	-2.491798000
1	-1.618707000	-3.688194000	-1.524668000
1	-2.086652000	-2.172750000	-2.413288000
1	-2.247659000	-3.767405000	-3.159822000
6	-1.987960000	-2.248283000	3.373530000
1	-2.576879000	-1.478119000	2.854316000
1	-1.668989000	-3.045154000	2.685422000
1	-2.657778000	-2.723411000	4.103359000
6	-1.358659000	-0.653950000	5.161680000
1	-0.563162000	-0.234744000	5.792699000
1	-1.882281000	0.171173000	4.657218000
1	-2.070831000	-1.171041000	5.819905000
6	-0.020860000	-2.769798000	4.801822000
1	0.377796000	-3.459655000	4.042719000
1	0.817093000	-2.374782000	5.393599000
1	-0.679917000	-3.334526000	5.476509000

6	0.912298000	1.742105000	-0.316073000
6	0.328151000	2.614599000	0.612205000
6	0.827364000	2.114487000	-1.664697000
6	-0.326250000	3.779228000	0.217596000
6	0.174383000	3.277304000	-2.069451000
6	-0.424590000	4.129989000	-1.134958000
1	0.359609000	2.370175000	1.678763000
1	1.269226000	1.472903000	-2.433208000
1	-0.787808000	4.423092000	0.972905000
1	0.113146000	3.523370000	-3.134011000
6	3.328318000	0.707141000	0.255139000
6	4.223210000	-0.318436000	0.616111000
6	3.888189000	1.966922000	0.017854000
6	5.591408000	-0.095860000	0.729446000
6	5.262479000	2.199137000	0.130637000
6	6.140912000	1.173293000	0.487857000
1	3.837425000	-1.322429000	0.812893000
1	3.234419000	2.797014000	-0.264087000
1	6.252705000	-0.921354000	1.011416000
1	5.659904000	3.200349000	-0.062937000
6	-1.190587000	5.346587000	-1.562469000
1	-0.989413000	6.204381000	-0.902866000
1	-0.947394000	5.635993000	-2.594934000
1	-2.275575000	5.151904000	-1.516401000
6	7.618330000	1.408821000	0.612024000
1	8.184288000	0.760322000	-0.076045000
1	7.879041000	2.452835000	0.387687000
1	7.973908000	1.179578000	1.629585000
6	-2.093764000	0.537232000	-0.137223000
6	-2.709270000	1.249296000	0.874171000
6	-2.262759000	1.064317000	-1.402062000
6	-3.429561000	2.406854000	0.603045000
6	-2.984756000	2.239602000	-1.590667000
7	-3.564343000	2.886143000	-0.609428000
9	-2.616156000	0.843591000	2.156233000
9	-1.733001000	0.467482000	-2.481983000
9	-4.010332000	3.080224000	1.592181000
9	-3.103898000	2.754686000	-2.810915000
6	0.104957000	-3.898531000	0.818636000
6	-0.598736000	-5.086604000	0.783942000
6	1.460848000	-4.048254000	1.034410000
6	0.036590000	-6.314867000	0.946315000
6	2.030922000	-5.310520000	1.187316000
7	1.328447000	-6.416237000	1.142096000
9	2.279076000	-2.984754000	1.104999000
9	3.339698000	-5.430220000	1.388937000

9	-0.664732000	-7.443220000	0.908232000
9	-1.931852000	-5.085899000	0.580226000

6.17.7 **INT11-Bi**

Energy(PBE0-D3(BJ)/def2-TZVPP) = -2834.75487223741

6	1.699829000	0.299689000	2.530227000
7	1.057464000	-0.051704000	1.363890000
6	0.018919000	-0.862675000	1.650963000
6	1.061542000	-0.318438000	3.554724000
1	1.269126000	-0.305329000	4.620491000
1	2.566250000	0.953100000	2.544332000
7	0.030696000	-1.044105000	2.996797000
6	-0.826800000	-1.909568000	3.847107000
83	-1.588987000	-1.539606000	-0.130817000
7	1.302916000	-0.531459000	-1.175048000
6	0.404077000	-1.524564000	-1.288700000
6	1.926528000	-1.802930000	-2.873101000
7	0.802202000	-2.342238000	-2.295068000
1	2.443274000	-2.270878000	-3.705332000
1	3.065247000	0.005546000	-2.298061000
6	2.233043000	-0.679690000	-2.175772000
6	0.376551000	-3.731529000	-2.648026000
5	1.534056000	0.545747000	-0.014743000
6	0.068889000	-3.796465000	-4.143211000
1	-0.724176000	-3.090135000	-4.415923000
1	0.957069000	-3.570888000	-4.750558000
1	-0.259896000	-4.813892000	-4.396819000
6	1.545842000	-4.657725000	-2.298196000
1	2.444262000	-4.423495000	-2.886032000
1	1.792711000	-4.578164000	-1.228871000
1	1.260324000	-5.697307000	-2.513600000
6	-0.837335000	-4.172653000	-1.847615000
1	-0.633426000	-4.174793000	-0.765875000
1	-1.725068000	-3.567764000	-2.077098000
1	-1.073371000	-5.208285000	-2.127791000
6	-1.863115000	-2.650056000	3.018689000
1	-2.569486000	-1.959904000	2.538496000
1	-1.392382000	-3.294381000	2.261824000
1	-2.443955000	-3.297261000	3.690411000
6	-1.530359000	-1.024605000	4.877521000
1	-0.809586000	-0.517530000	5.535010000
1	-2.142406000	-0.262151000	4.375704000
1	-2.185436000	-1.642792000	5.508153000
6	0.076098000	-2.935949000	4.536046000
1	0.603547000	-3.546542000	3.787691000
1	0.822400000	-2.454833000	5.183953000
1	-0.535678000	-3.602027000	5.161460000
6	0.807877000	1.951457000	-0.322142000
6	0.264771000	2.743997000	0.700068000
6	0.817557000	2.509547000	-1.607165000
6	-0.249489000	4.014305000	0.453242000
6	0.300422000	3.778712000	-1.864968000
6	-0.247471000	4.556121000	-0.838691000
1	0.231548000	2.357553000	1.723060000

1	1.229311000	1.934864000	-2.442492000
1	-0.674038000	4.596355000	1.277167000
1	0.313086000	4.172904000	-2.885787000
6	3.153090000	0.727837000	0.099579000
6	3.965969000	-0.378590000	0.410932000
6	3.807505000	1.949110000	-0.083692000
6	5.346901000	-0.271528000	0.526021000
6	5.197268000	2.066735000	0.029572000
6	5.993574000	0.960685000	0.334930000
1	3.500139000	-1.357704000	0.567734000
1	3.220544000	2.840273000	-0.322952000
1	5.942315000	-1.157493000	0.768697000
1	5.671573000	3.041342000	-0.122339000
6	-0.858110000	5.899048000	-1.111397000
1	-0.614201000	6.623134000	-0.319380000
1	-0.520277000	6.308362000	-2.074411000
1	-1.957442000	5.820973000	-1.152967000
6	7.485712000	1.072944000	0.457169000
1	7.995180000	0.424782000	-0.274153000
1	7.825903000	2.104983000	0.291426000
1	7.829137000	0.756683000	1.455354000
6	-2.204700000	0.692659000	-0.144503000
6	-2.805030000	1.156456000	1.015463000
6	-2.252466000	1.553775000	-1.231008000
6	-3.399572000	2.413581000	1.050321000
6	-2.891669000	2.785857000	-1.104675000
7	-3.446905000	3.199194000	0.005782000
9	-2.840919000	0.399249000	2.117898000
9	-1.712386000	1.249000000	-2.401748000
9	-3.956532000	2.845584000	2.172413000
9	-2.962129000	3.588035000	-2.157480000
6	-2.814649000	-1.450279000	-2.363649000
6	-4.152592000	-1.774862000	-2.237314000
6	-2.414801000	-1.181665000	-3.659917000
6	-4.995912000	-1.833762000	-3.343824000
6	-3.312757000	-1.278203000	-4.719690000
7	-4.575056000	-1.594002000	-4.560938000
9	-1.152388000	-0.837520000	-3.953670000
9	-2.894392000	-1.052537000	-5.962000000
9	-6.278773000	-2.146806000	-3.191463000
9	-4.674964000	-2.054007000	-1.027152000

6.17.8 **TS12-Bi**

Energy(PBE0-D3(BJ)/def2-TZVPP) = -2834.72045835984

6	1.900810000	0.225598000	2.700941000
7	1.401370000	-0.202117000	1.497655000
6	0.271061000	-0.890753000	1.722185000
6	1.070539000	-0.221186000	3.678467000
1	1.122654000	-0.086731000	4.753876000
1	2.807701000	0.816943000	2.773225000
7	0.065632000	-0.928076000	3.062716000
6	-1.042730000	-1.522269000	3.867835000
83	-0.915176000	-1.912970000	0.019562000
7	1.895740000	-0.725503000	-1.009733000
6	0.903350000	-1.568629000	-1.332654000
6	2.397965000	-1.622907000	-2.964108000
7	1.202772000	-2.139420000	-2.523377000
1	2.854607000	-1.915661000	-3.904128000
1	3.729488000	-0.146961000	-1.985759000
6	2.826967000	-0.748477000	-2.018216000
6	0.450557000	-3.116191000	-3.365360000
5	2.003522000	0.355465000	0.148718000
6	0.033520000	-2.401844000	-4.651839000
1	-0.629772000	-1.556771000	-4.421364000
1	0.904078000	-2.029795000	-5.210559000
1	-0.508125000	-3.108146000	-5.297134000
6	1.378975000	-4.295811000	-3.657769000
1	2.268113000	-3.992336000	-4.227542000
1	1.704980000	-4.771045000	-2.720579000
1	0.835730000	-5.040734000	-4.255930000
6	-0.789734000	-3.635523000	-2.661861000
1	-0.547807000	-4.161278000	-1.726167000
1	-1.533247000	-2.841910000	-2.484234000
1	-1.276098000	-4.364707000	-3.324224000
6	-1.883663000	-2.484037000	3.045564000
1	-2.467160000	-1.973600000	2.267646000
1	-1.277168000	-3.294450000	2.614804000
1	-2.619582000	-2.949121000	3.715765000
6	-1.913130000	-0.371554000	4.374179000
1	-1.333064000	0.322999000	4.999159000
1	-2.343832000	0.183414000	3.528882000
1	-2.735134000	-0.775494000	4.982637000
6	-0.422632000	-2.299001000	5.030916000
1	0.262091000	-3.075367000	4.658411000
1	0.124571000	-1.648077000	5.725945000
1	-1.226177000	-2.787694000	5.599150000
6	1.184926000	1.683456000	-0.276042000
6	0.295683000	2.337935000	0.587452000
6	1.394483000	2.283412000	-1.527084000
6	-0.351843000	3.517766000	0.224220000
6	0.753749000	3.463317000	-1.896346000
6	-0.138506000	4.102749000	-1.027649000
1	0.090430000	1.922702000	1.578710000

1	2.078262000	1.817779000	-2.243460000
1	-1.050137000	3.987649000	0.923654000
1	0.938423000	3.893643000	-2.885335000
6	3.585118000	0.614208000	0.406242000
6	4.426101000	-0.461584000	0.746791000
6	4.182428000	1.876099000	0.332226000
6	5.783408000	-0.286815000	0.992022000
6	5.546804000	2.061101000	0.577542000
6	6.373460000	0.984720000	0.909879000
1	4.003868000	-1.469686000	0.821548000
1	3.568243000	2.743930000	0.075820000
1	6.403711000	-1.149976000	1.253399000
1	5.977388000	3.064887000	0.509384000
6	-0.891688000	5.330361000	-1.445090000
1	-1.015590000	6.034990000	-0.608972000
1	-0.390211000	5.853821000	-2.272077000
1	-1.901908000	5.051692000	-1.788874000
6	7.841186000	1.168000000	1.166652000
1	8.445075000	0.626470000	0.420331000
1	8.127151000	2.228598000	1.127806000
1	8.125681000	0.771238000	2.154146000
6	-2.162807000	-0.150806000	-0.817429000
6	-2.924999000	0.595302000	0.125281000
6	-1.696528000	0.696484000	-1.863079000
6	-3.423205000	1.840840000	-0.176631000
6	-2.274455000	1.923324000	-2.083350000
7	-3.146135000	2.504744000	-1.279048000
9	-3.277139000	0.006969000	1.290061000
9	-0.817762000	0.218687000	-2.759294000
9	-4.218069000	2.448640000	0.707063000
9	-1.937350000	2.603367000	-3.180447000
6	-3.475260000	-1.448403000	-1.614179000
6	-4.350881000	-2.136284000	-0.791031000
6	-3.978463000	-1.164421000	-2.873482000
6	-5.633169000	-2.465687000	-1.214634000
6	-5.278382000	-1.524153000	-3.220021000
7	-6.088037000	-2.154455000	-2.404333000
9	-3.230680000	-0.554592000	-3.805367000
9	-5.740454000	-1.239753000	-4.433302000
9	-6.449082000	-3.136329000	-0.407907000
9	-3.974236000	-2.537280000	0.441021000

7 NMR Characterization data

7.1 NMR characterization of defluoroalkoxylation products

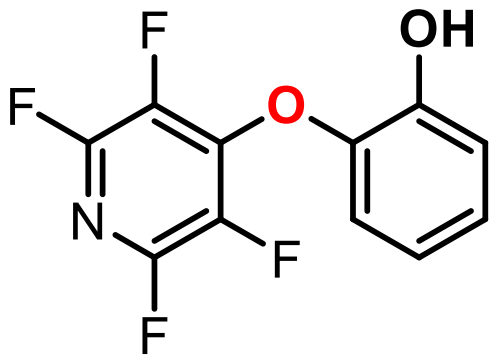

2f

^{1}H NMR, $CDCl_3$

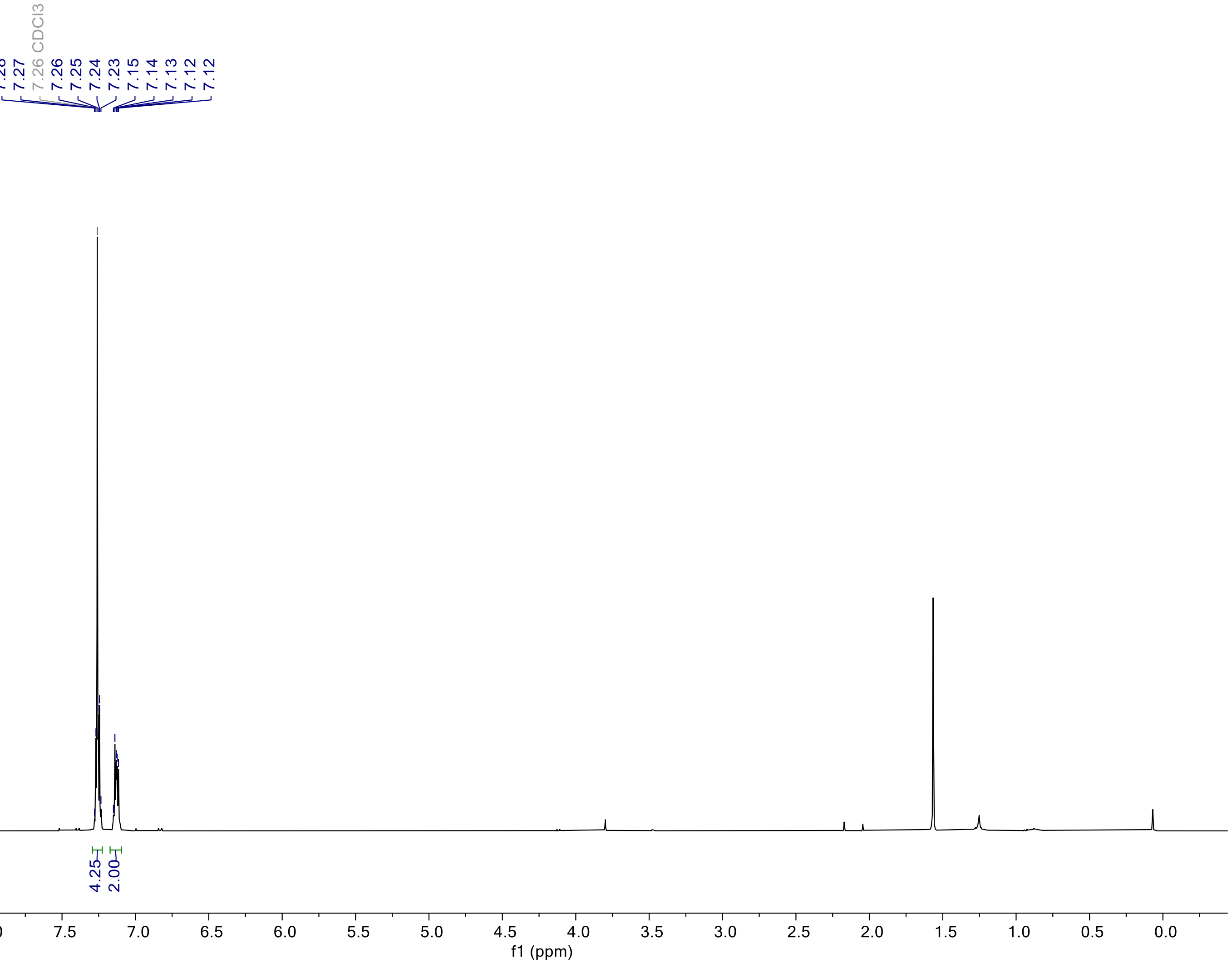

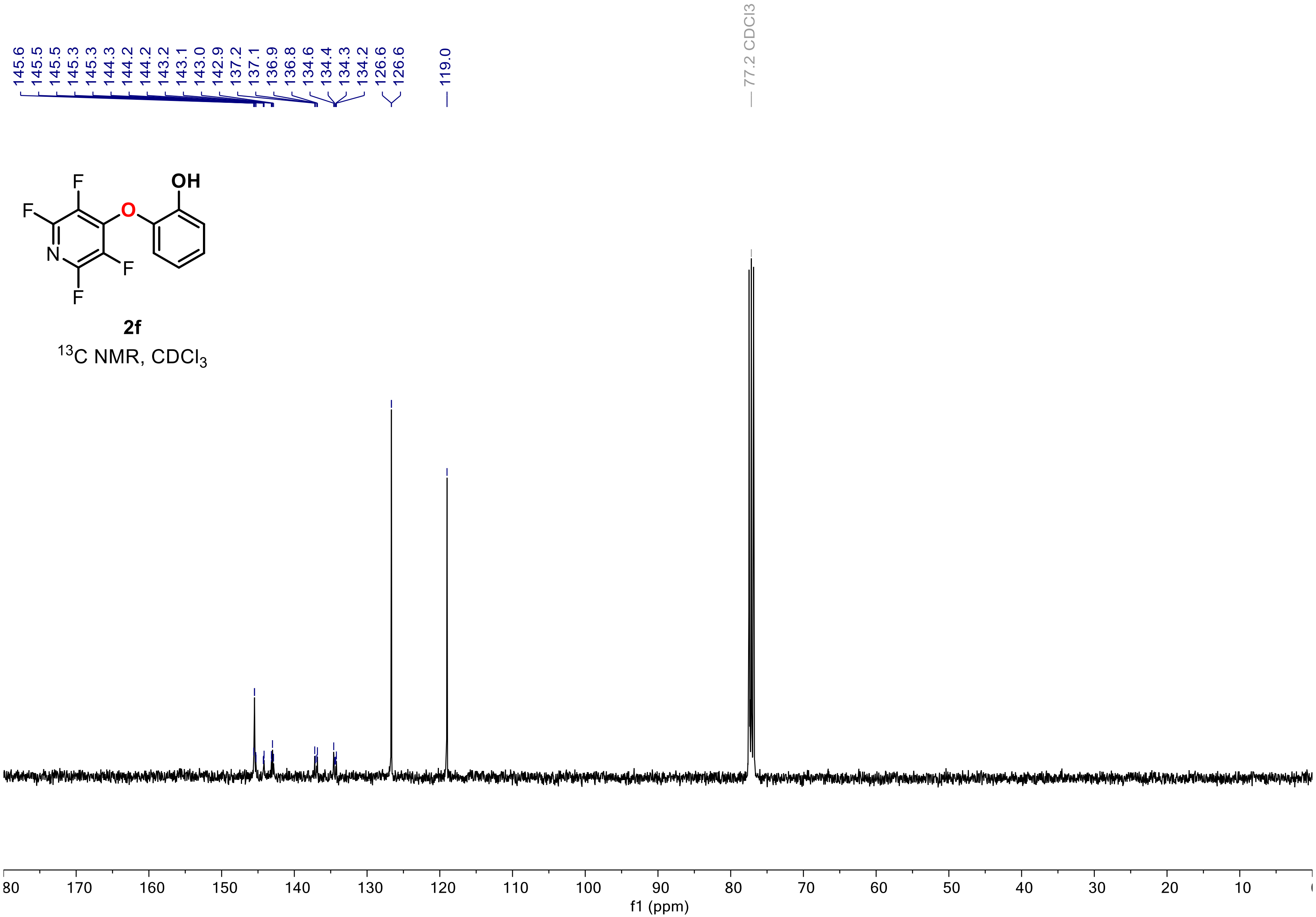
145.6
145.5
145.5
145.3
145.3
144.3
144.2
144.2
143.2
143.1
143.0
142.9
137.2
137.1
136.9
136.8
134.6
134.4
134.3
134.2
126.6
126.6
119.0
77.2 CDCl3
OH
F
F
O
N
F
F
2f
^{13}C NMR, $CDCl_3$
80
170
160
150
140
130
120
110
100
90
80
70
60
50
40
30
20
10
f1 (ppm)

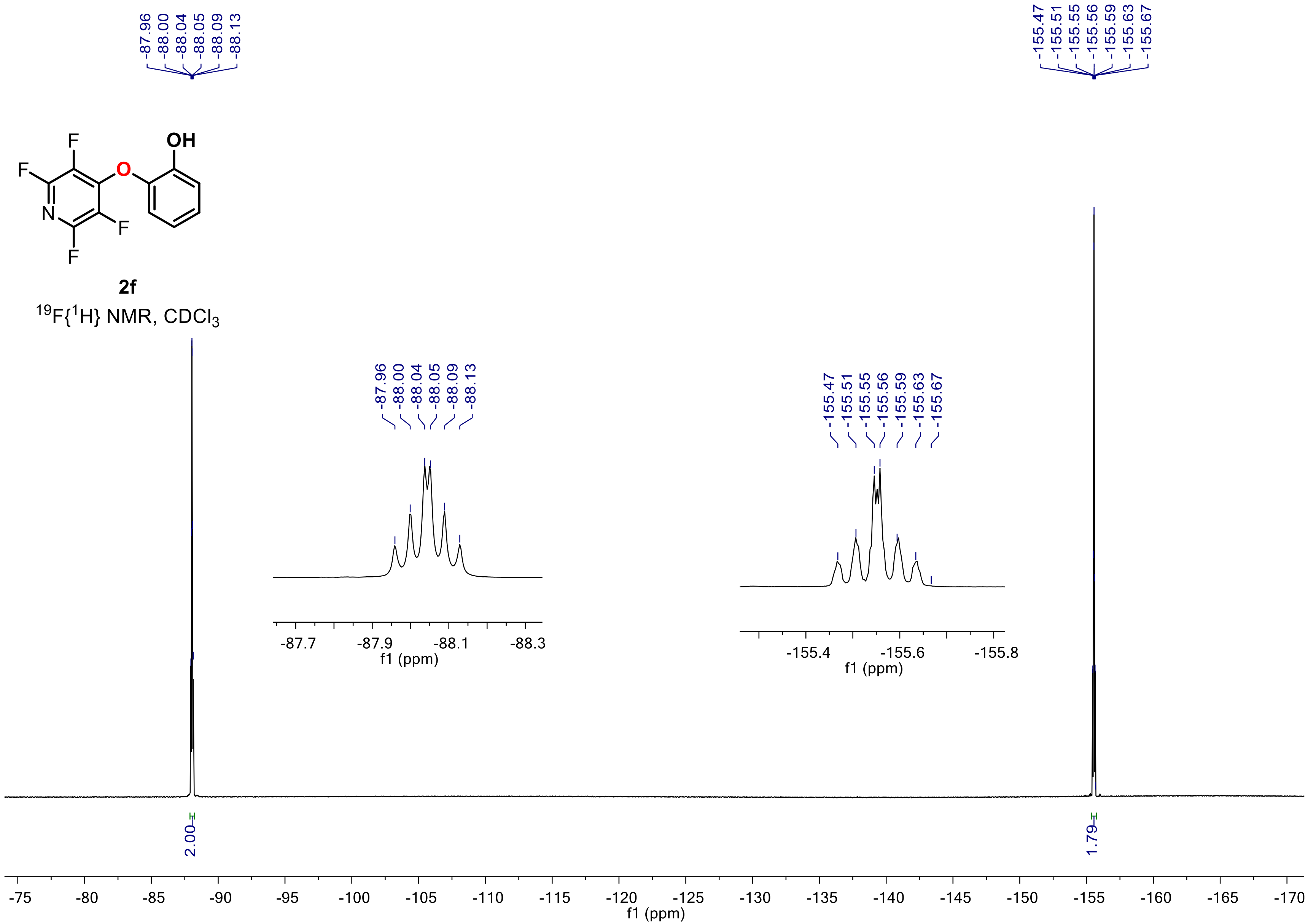

2f
$^{19}F\{^{1}H\}$ NMR, $CDCl_3$
-87.96
-88.00
-88.04
-88.05
-88.09
-88.13
-155.47
-155.51
-155.55
-155.56
-155.59
-155.63
-155.67
2.00
1.79
f1 (ppm)

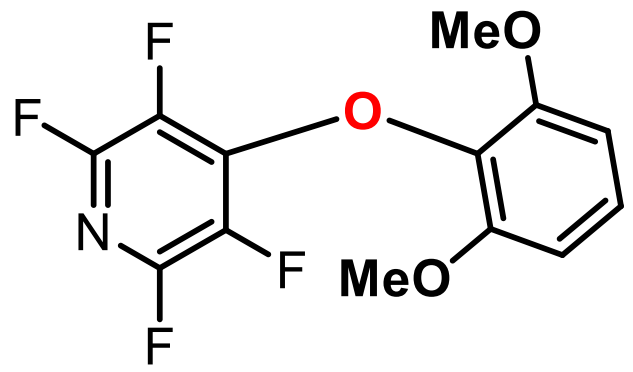

2g

^{1}H NMR, $CDCl_3$

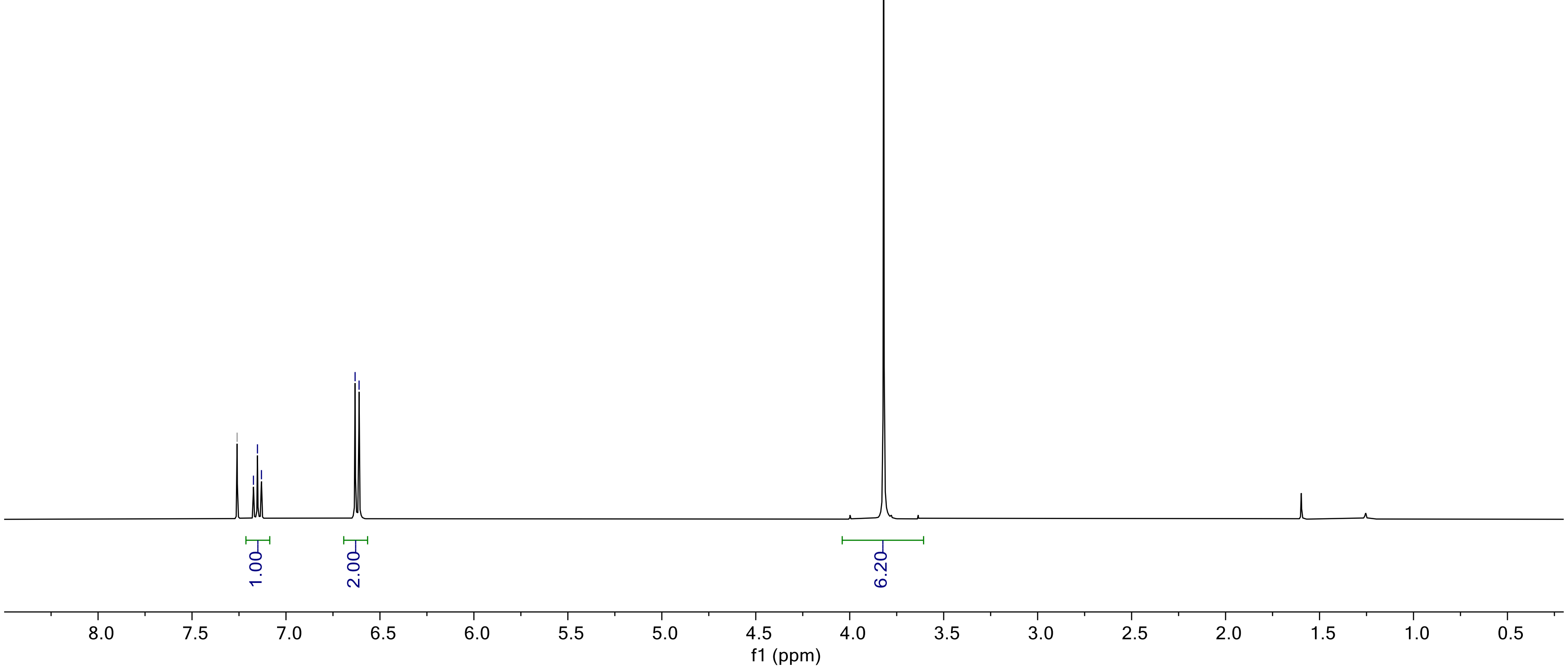

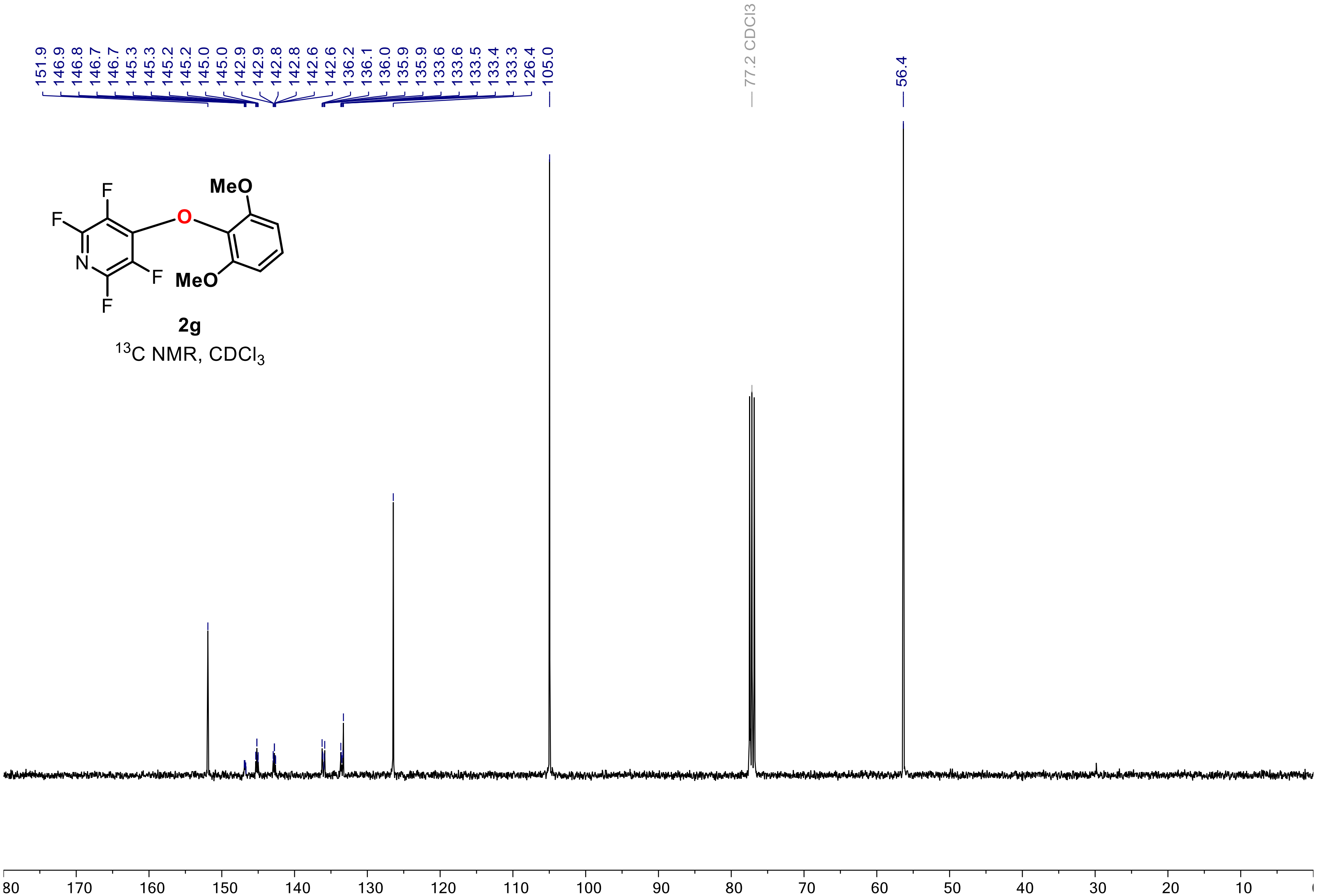

2g

^{13}C NMR, $CDCl_3$

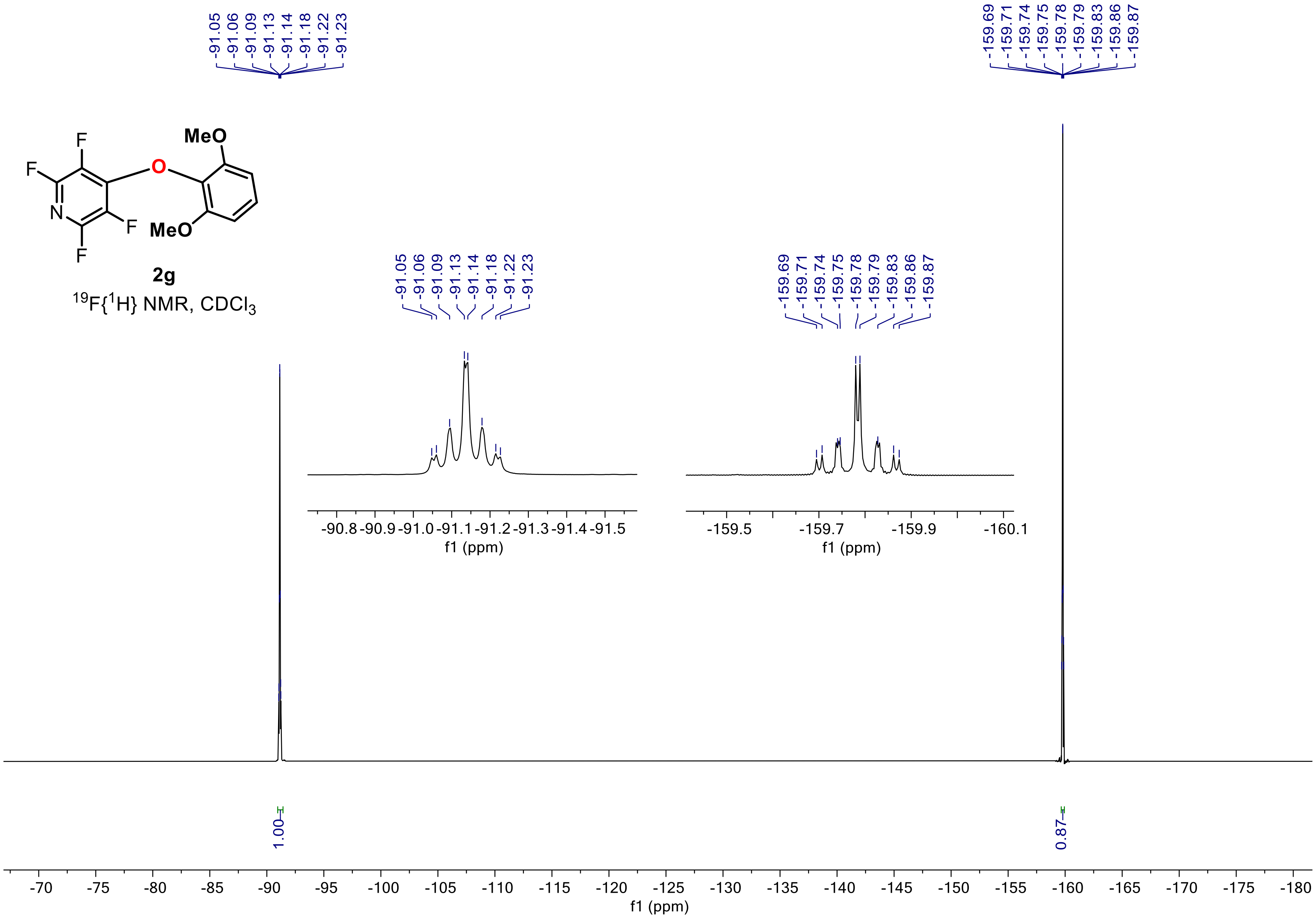

2g

$^{19}F\{^{1}H\}$ NMR, $CDCl_3$

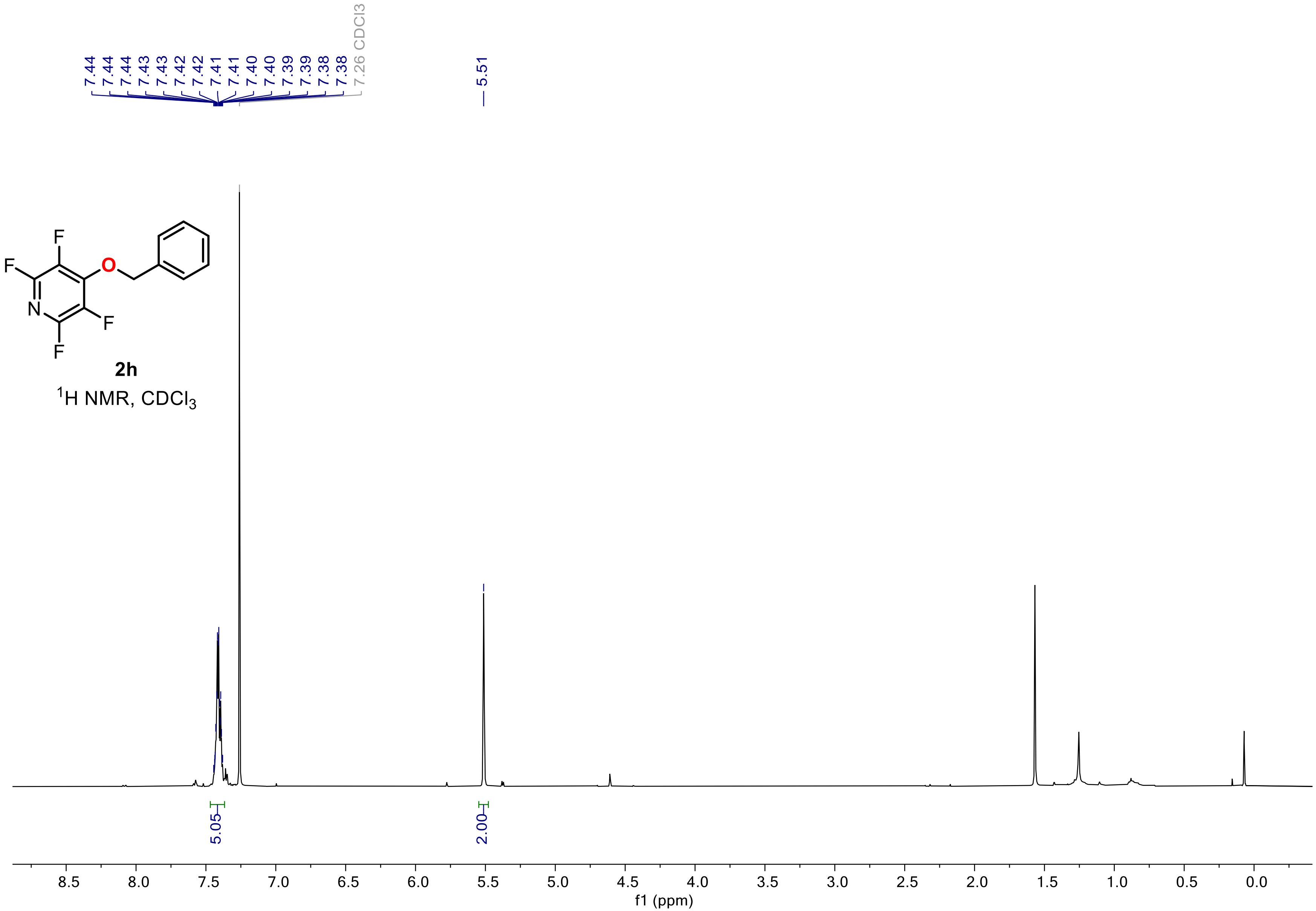

2h

^{1}H NMR, $CDCl_3$

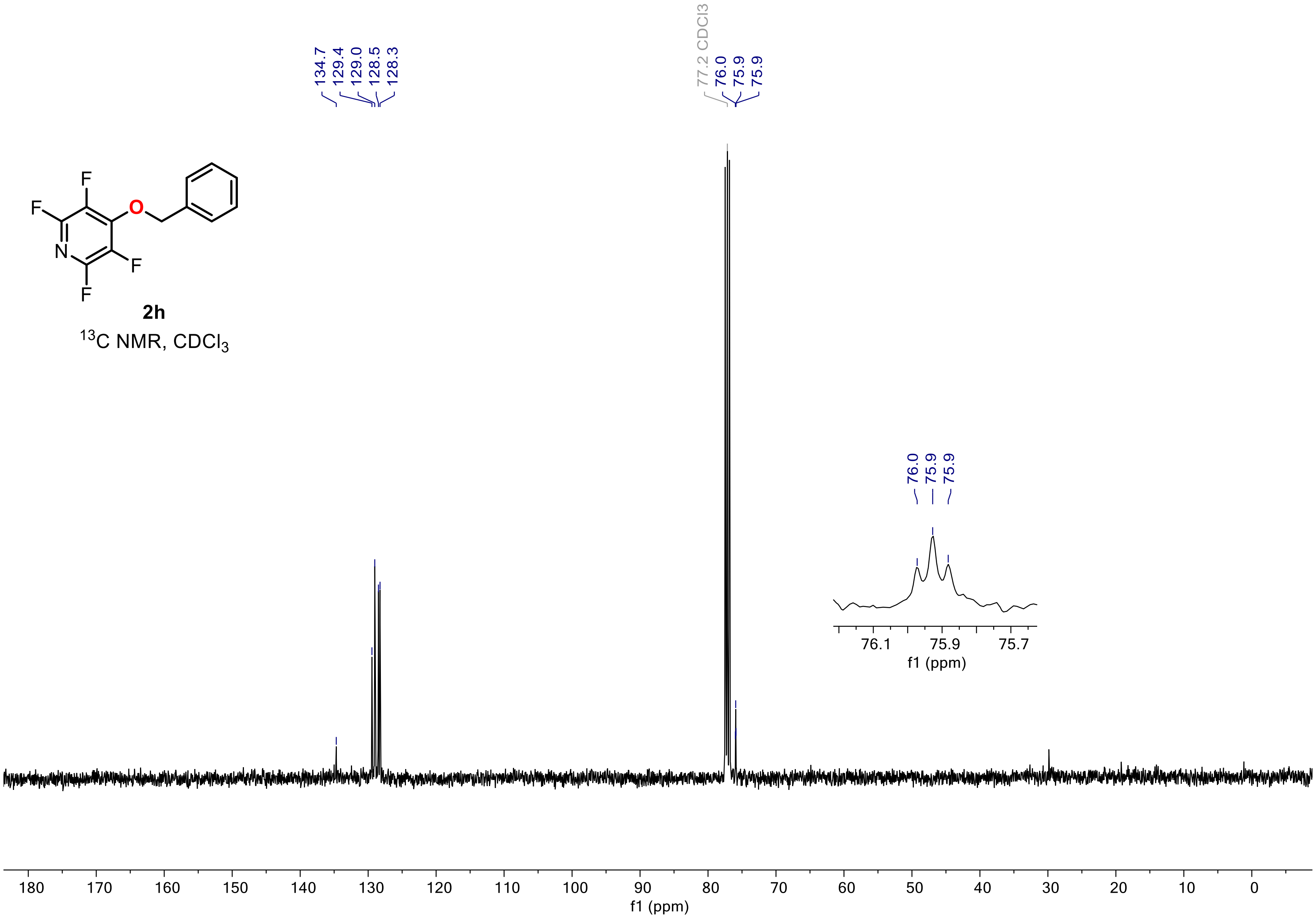

2h

^{13}C NMR, $CDCl_3$

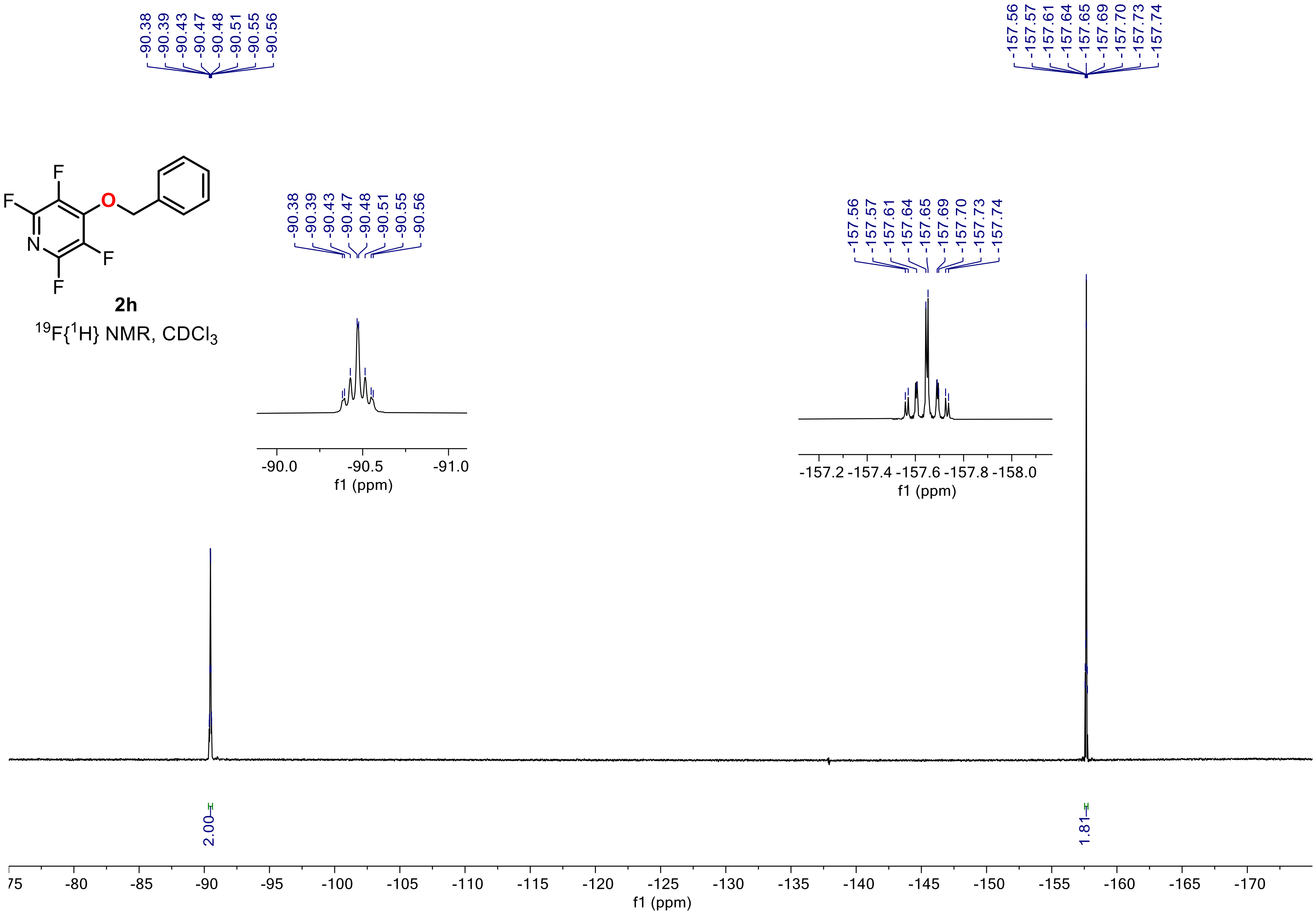
-90.38
-90.39
-90.43
-90.47
-90.48
-90.51
-90.55
-90.56
-157.56
-157.57
-157.61
-157.64
-157.65
-157.69
-157.70
-157.73
-157.74
2h
$^{19}F\{^1H\}$ NMR, $CDCl_3$
-90.0
-90.5
-91.0
f1 (ppm)
-157.2 -157.4 -157.6 -157.8 -158.0
f1 (ppm)
2.00
1.81
75 -80 -85 -90 -95 -100 -105 -110 -115 -120 -125 -130 -135 -140 -145 -150 -155 -160 -165 -170
f1 (ppm)

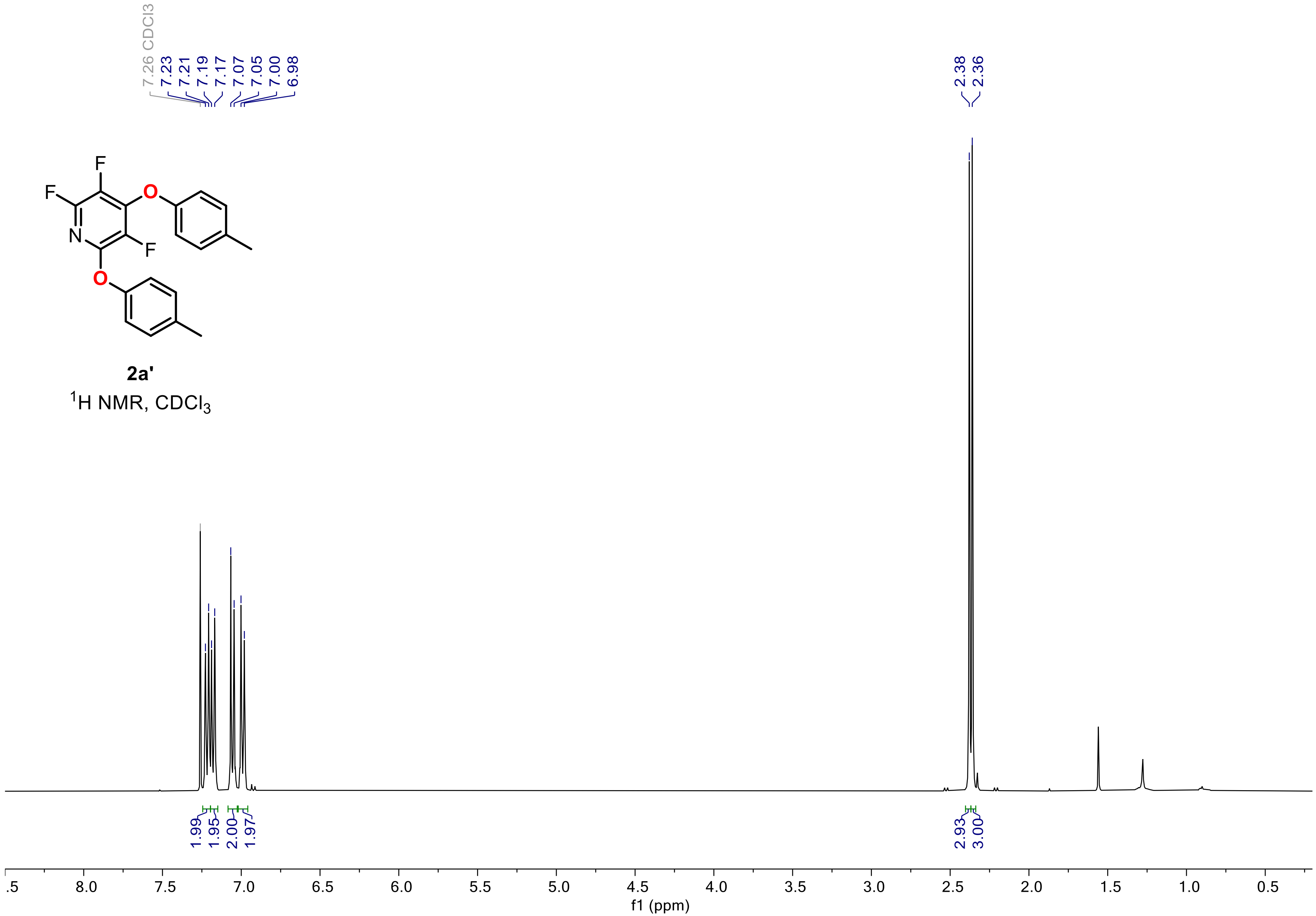

2a'

^{1}H NMR, $CDCl_3$

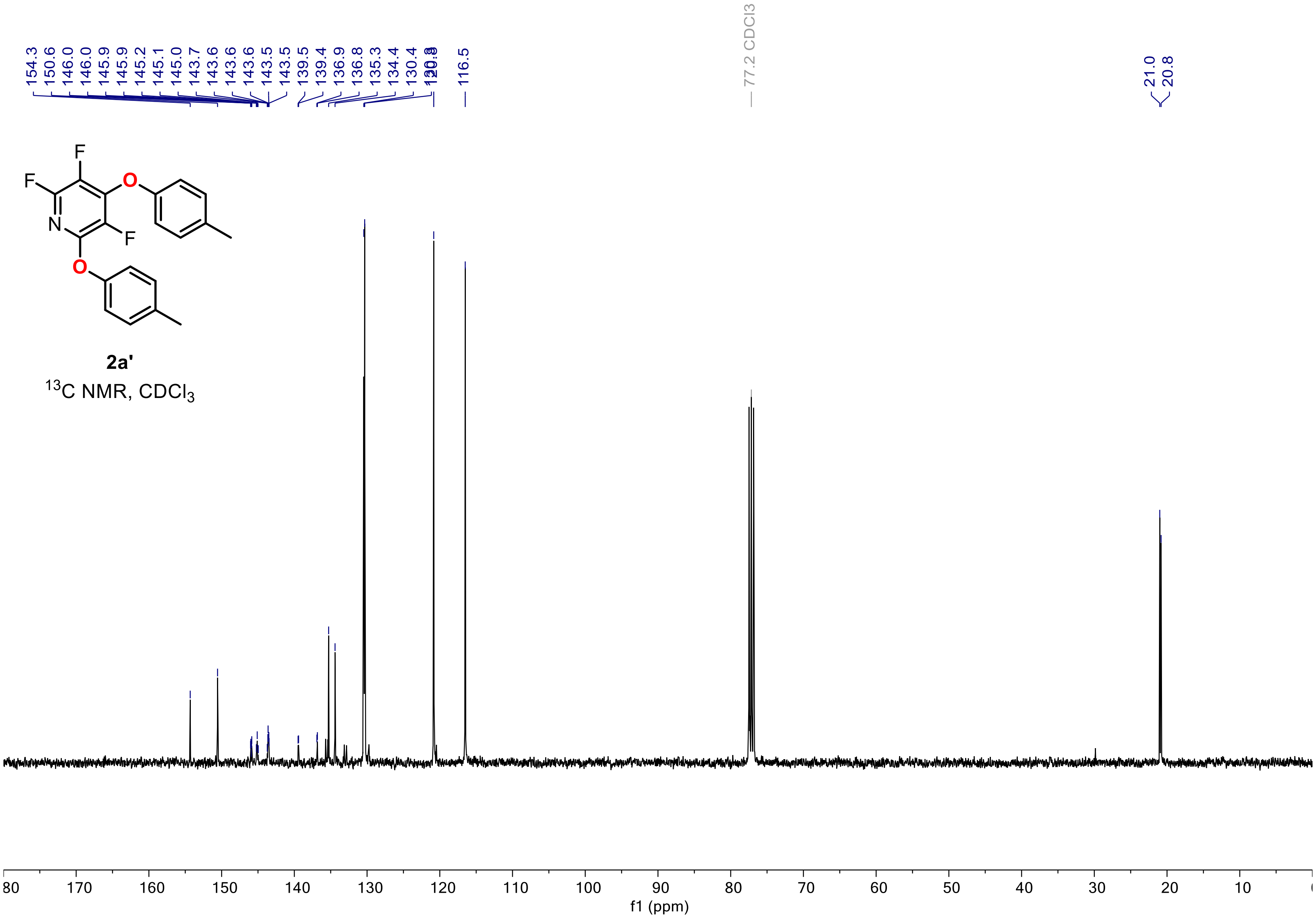

2a'

^{13}C NMR, $CDCl_3$

2a'

$^{19}F\{^{1}H\}$ NMR, $CDCl_3$

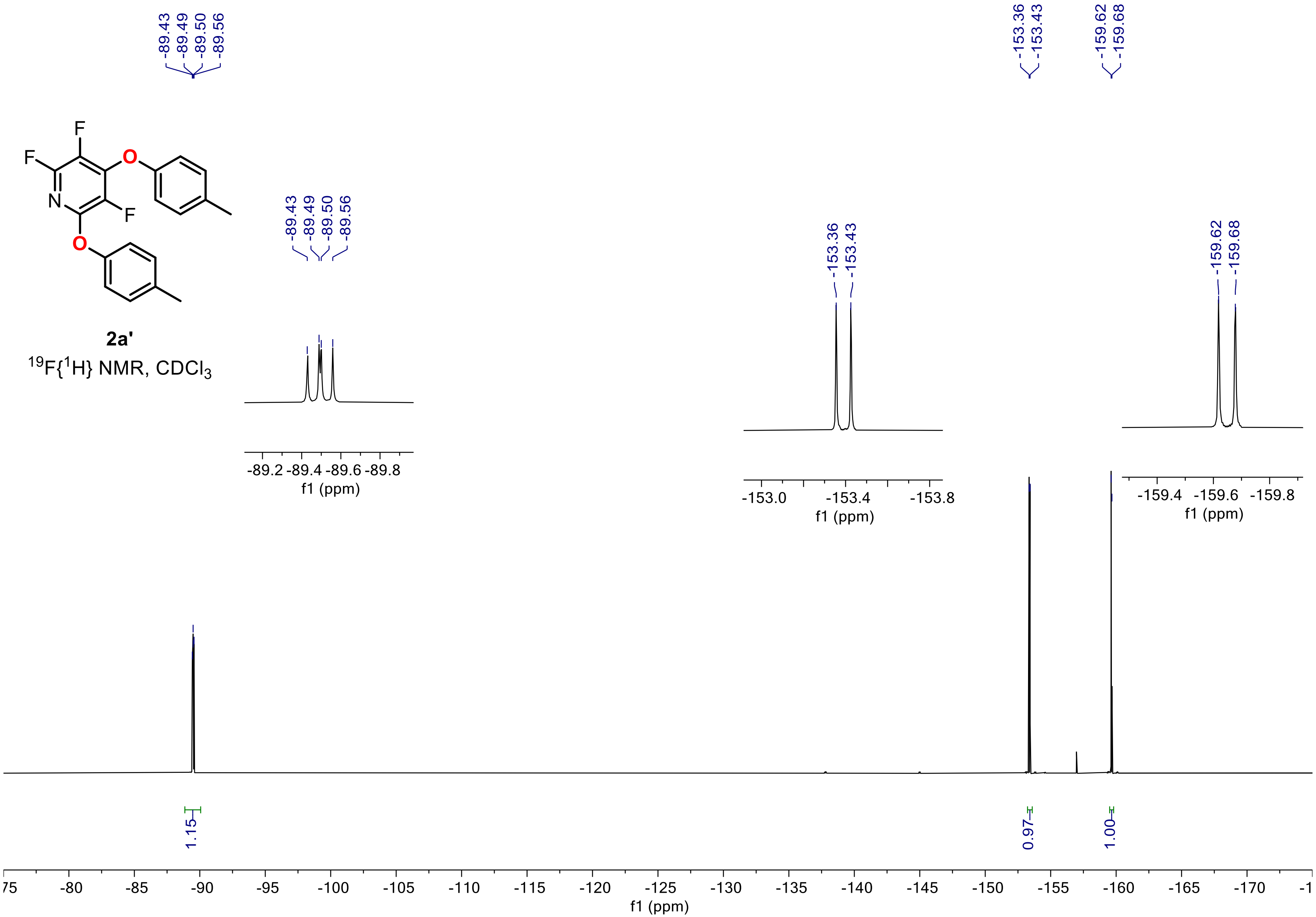

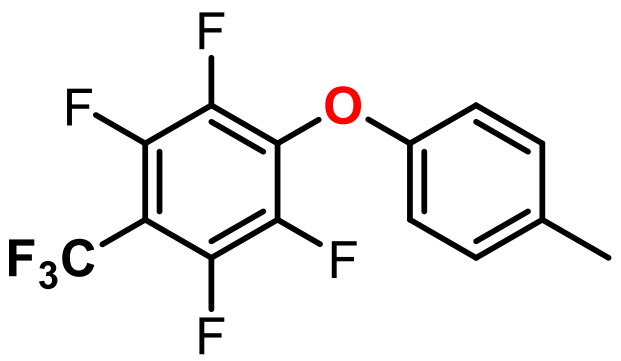

2i

^{1}H NMR, $CDCl_3$

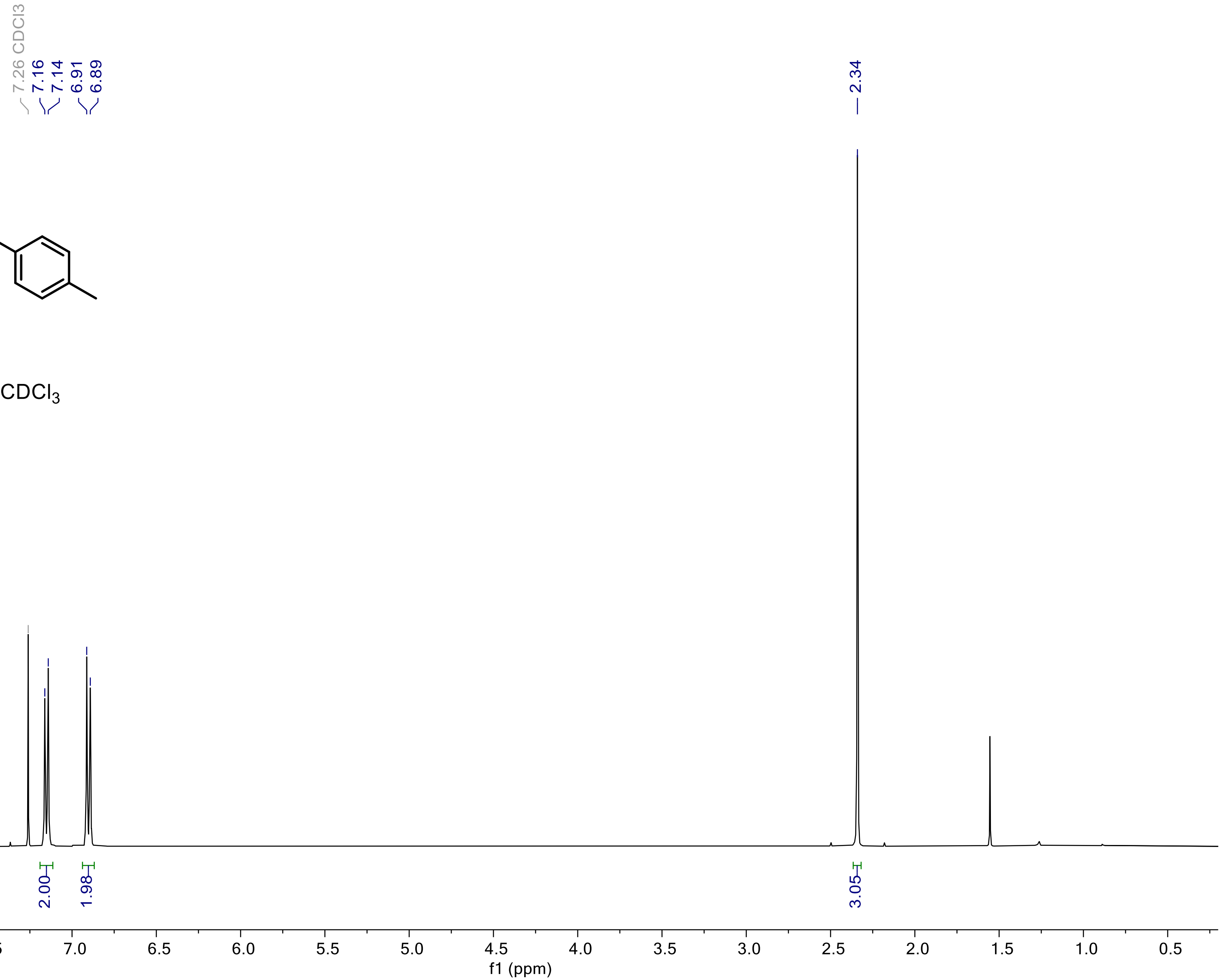

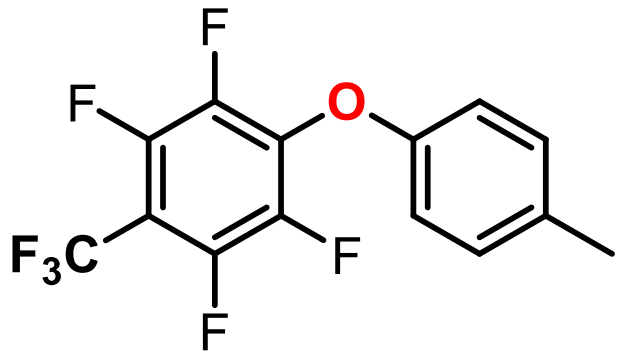

2i

^{13}C NMR, $CDCl_3$

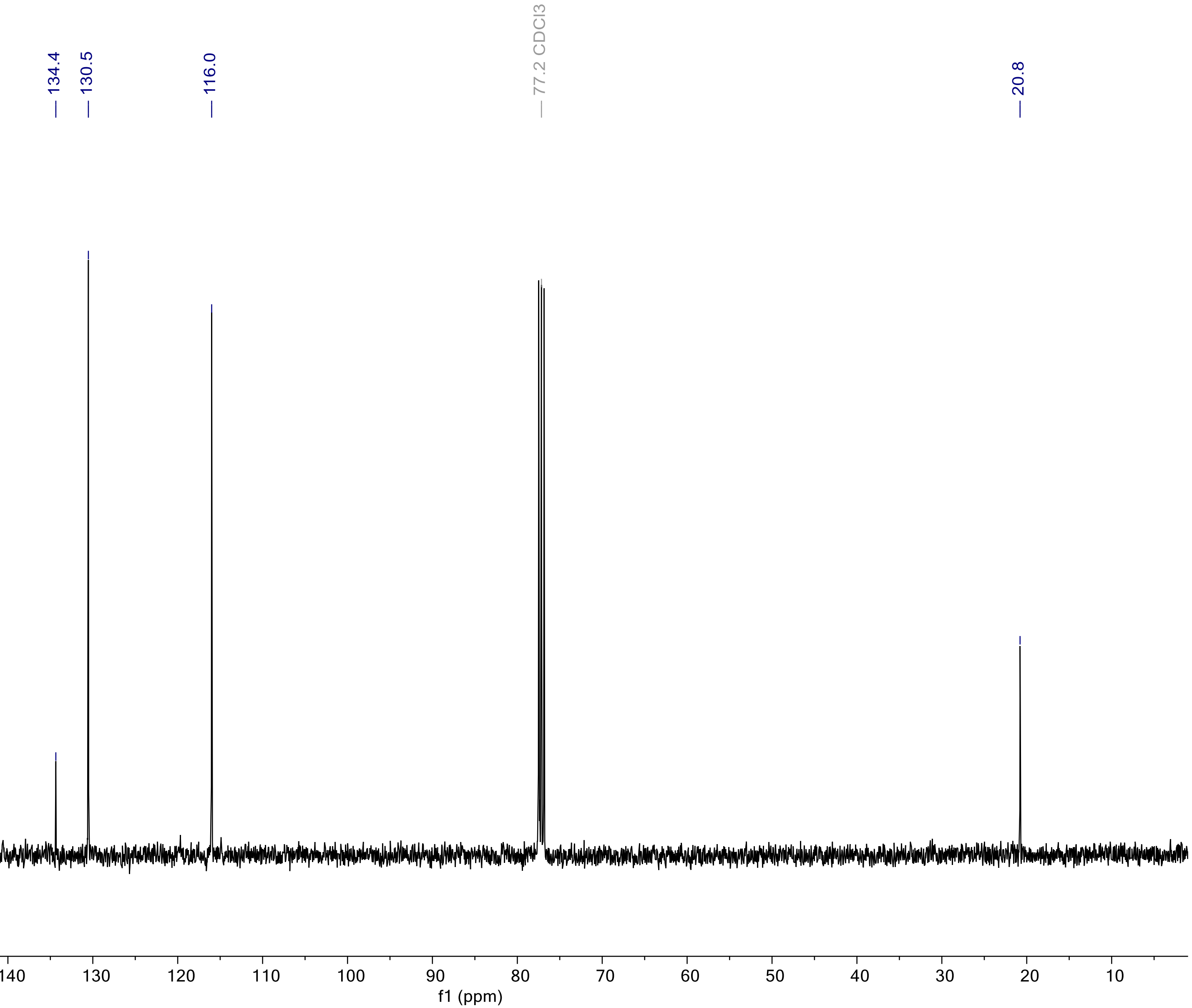

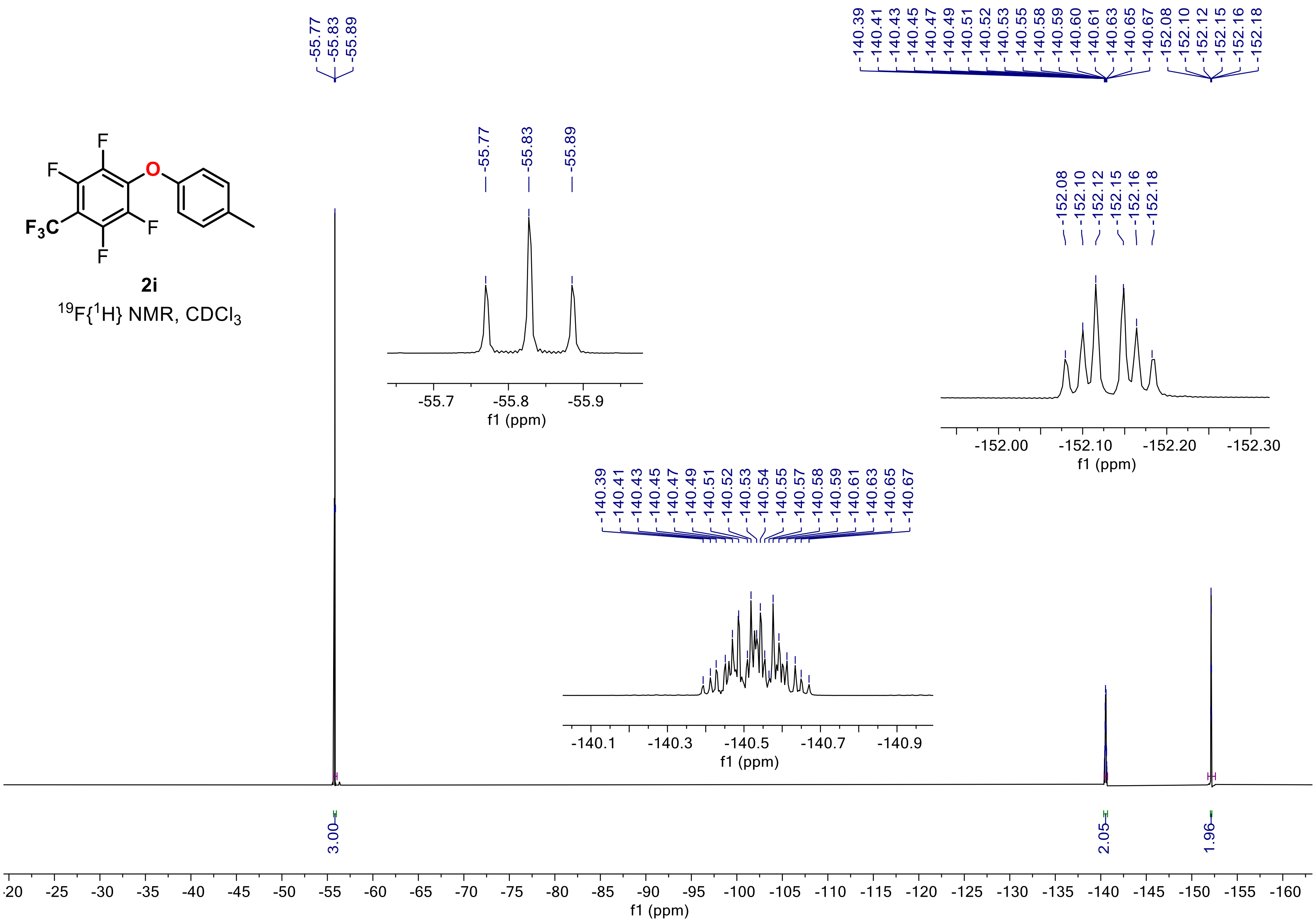
2i
$^{19}F\{^1H\}$ NMR, $CDCl_3$
-55.77
-55.83
-55.89
-140.39
-140.41
-140.43
-140.45
-140.47
-140.49
-140.51
-140.52
-140.53
-140.55
-140.58
-140.59
-140.60
-140.61
-140.63
-140.65
-140.67
-152.08
-152.10
-152.12
-152.15
-152.16
-152.18
3.00
2.05
1.96
f1 (ppm)

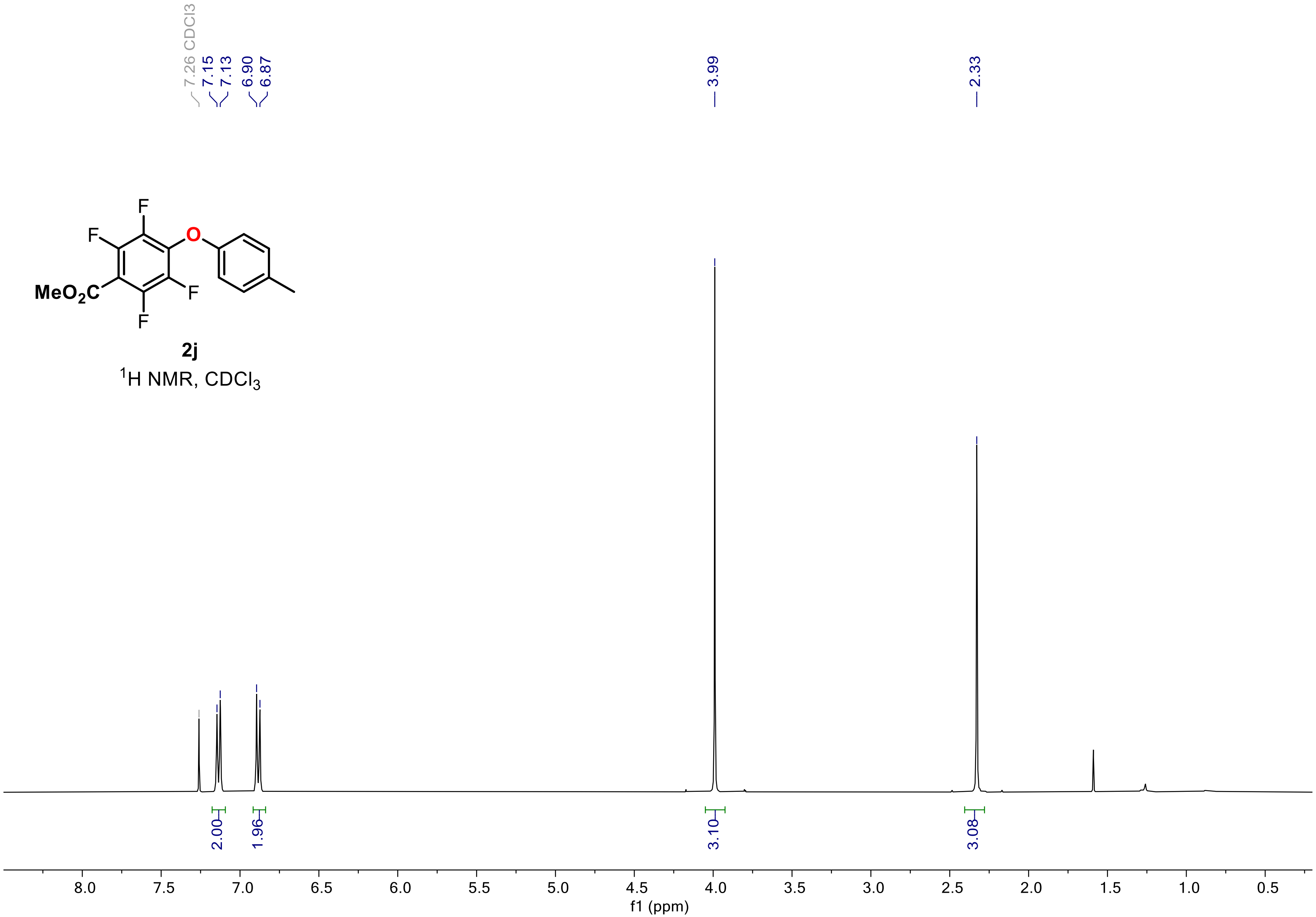

2j

^{1}H NMR, $CDCl_3$

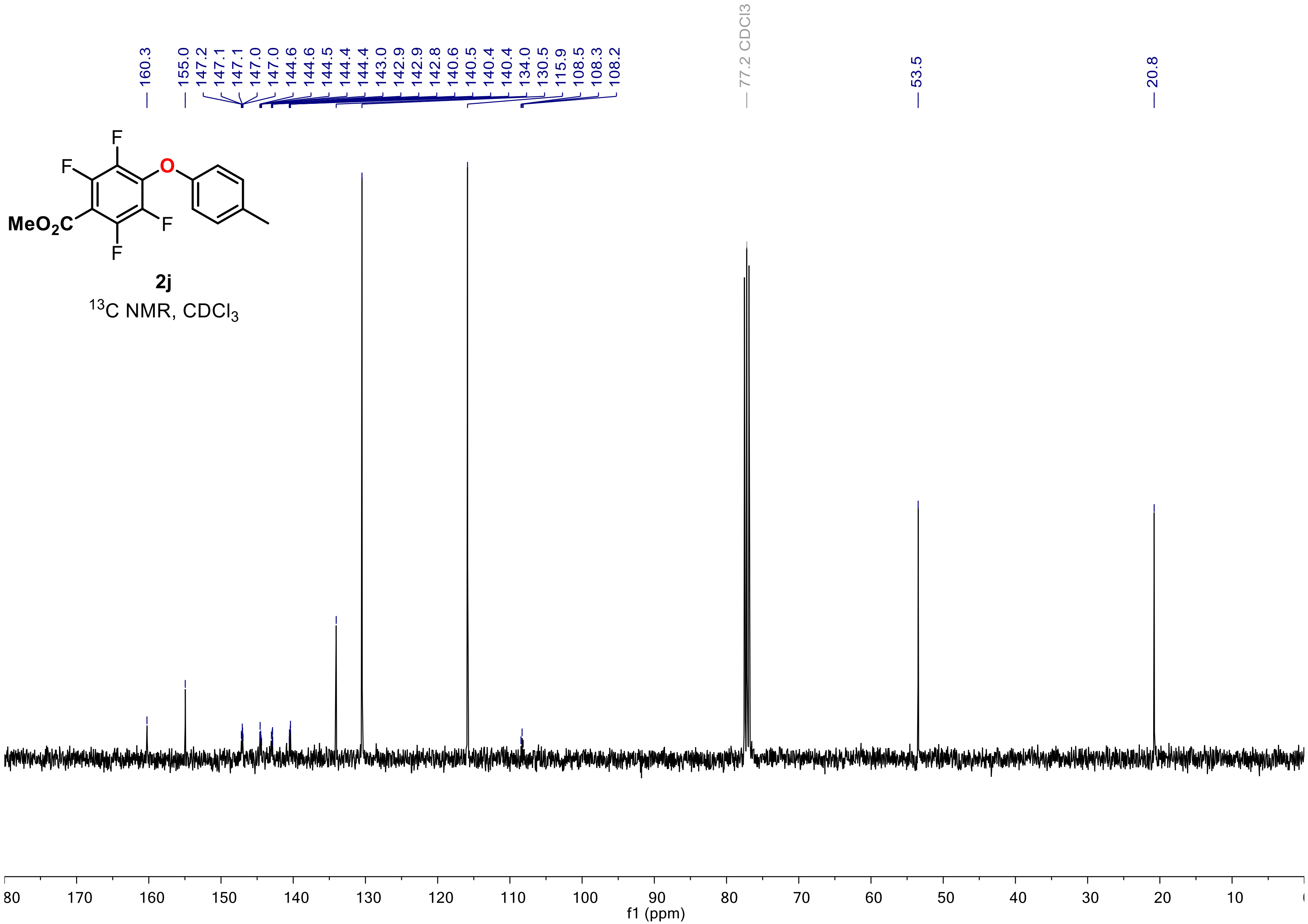

2j

^{13}C NMR, $CDCl_3$

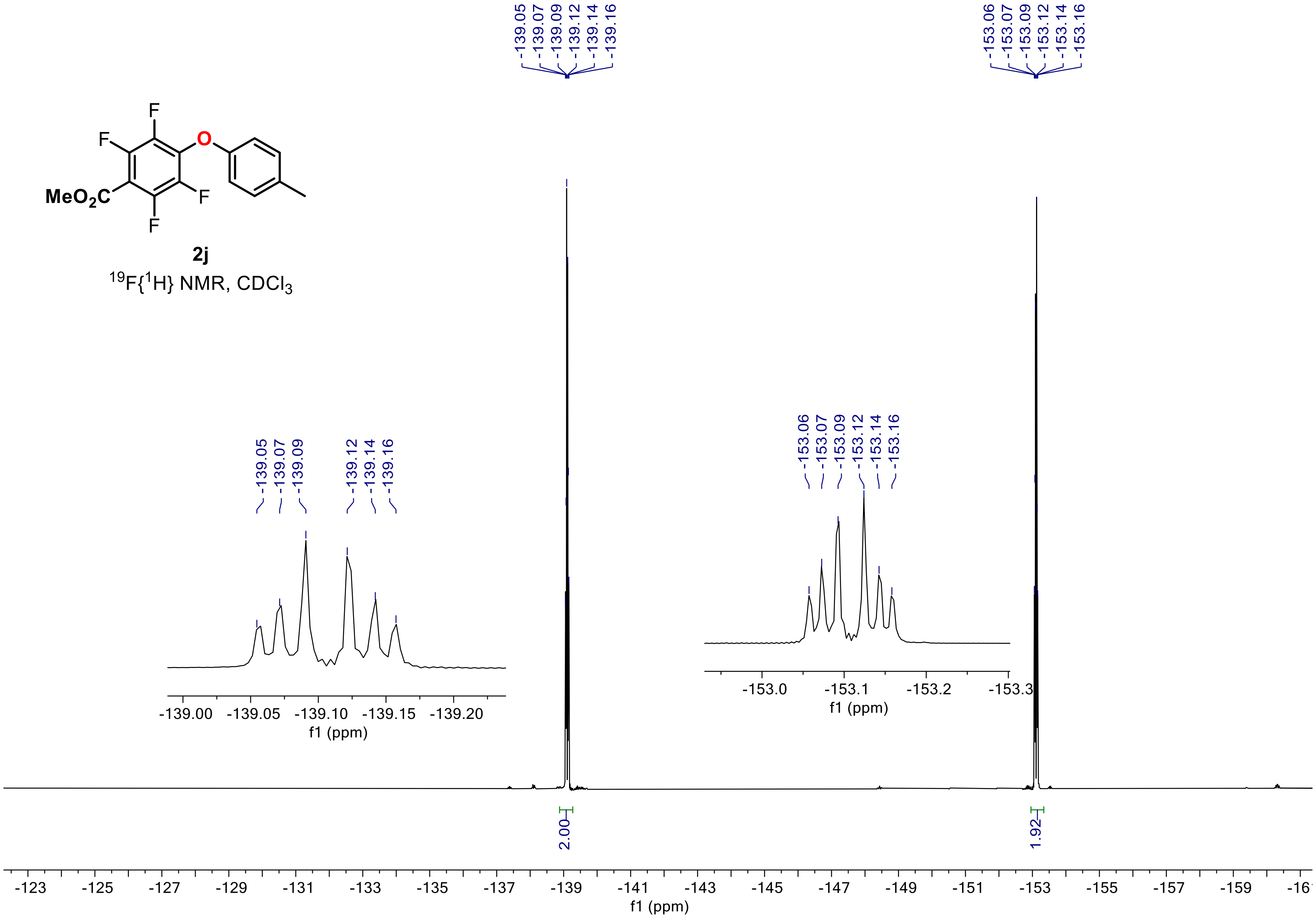

2j

$^{19}F\{^1H\}$ NMR, $CDCl_3$

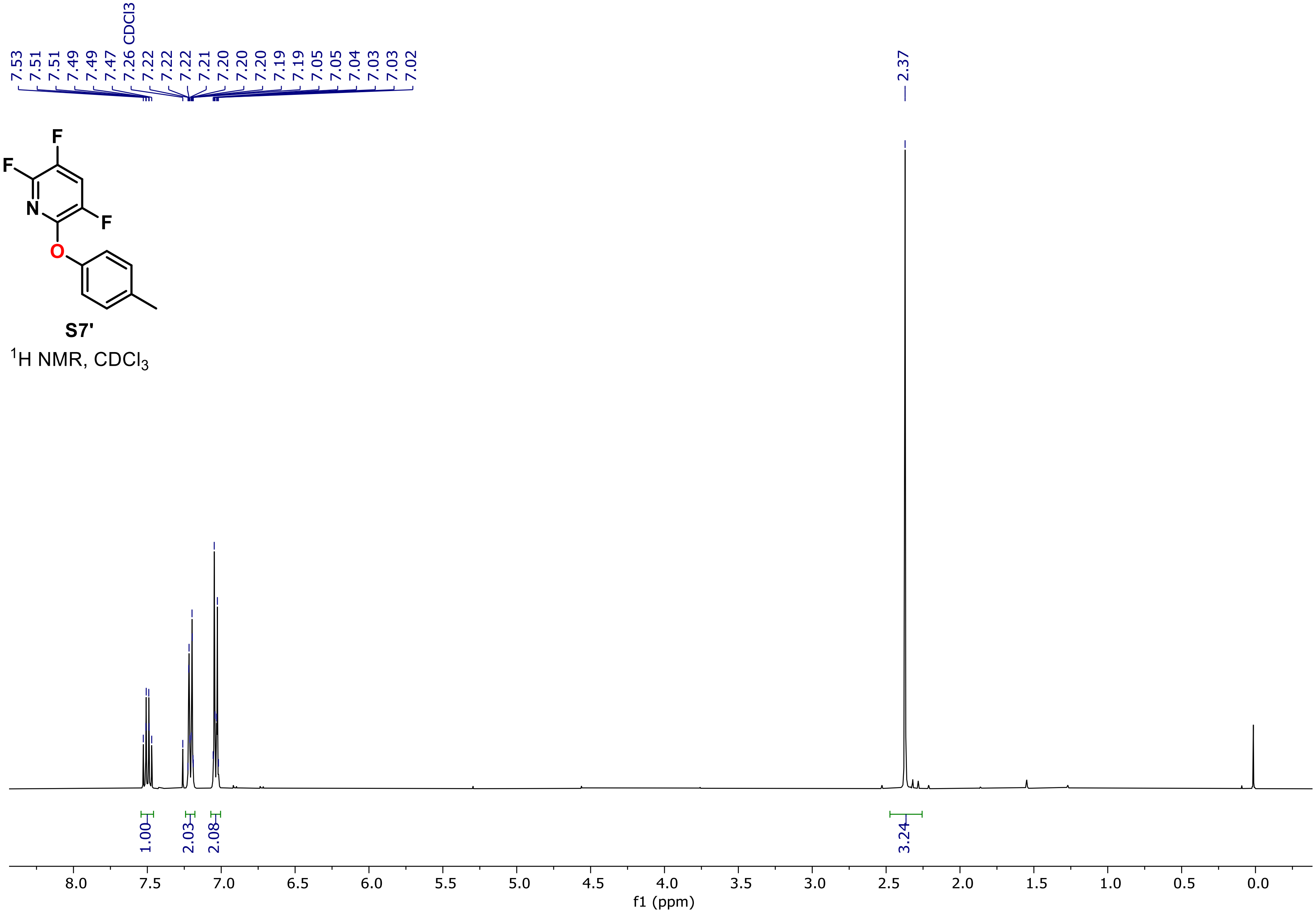
7.53
7.51
7.51
7.49
7.49
7.47
7.26 CDCl3
7.22
7.22
7.22
7.21
7.20
7.20
7.19
7.19
7.05
7.05
7.04
7.03
7.03
7.02
2.37
F
F
N
F
O
S7'
1H NMR, CDCl3
1.00
2.03
2.08
3.24
8.0
7.5
7.0
6.5
6.0
5.5
5.0
4.5
4.0
3.5
3.0
2.5
2.0
1.5
1.0
0.5
0.0
f1 (ppm)

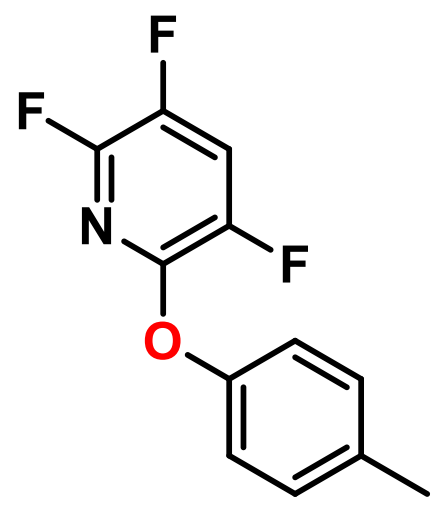

S7'

$^{13}C\{^{1}H\}$ NMR, $CDCl_3$

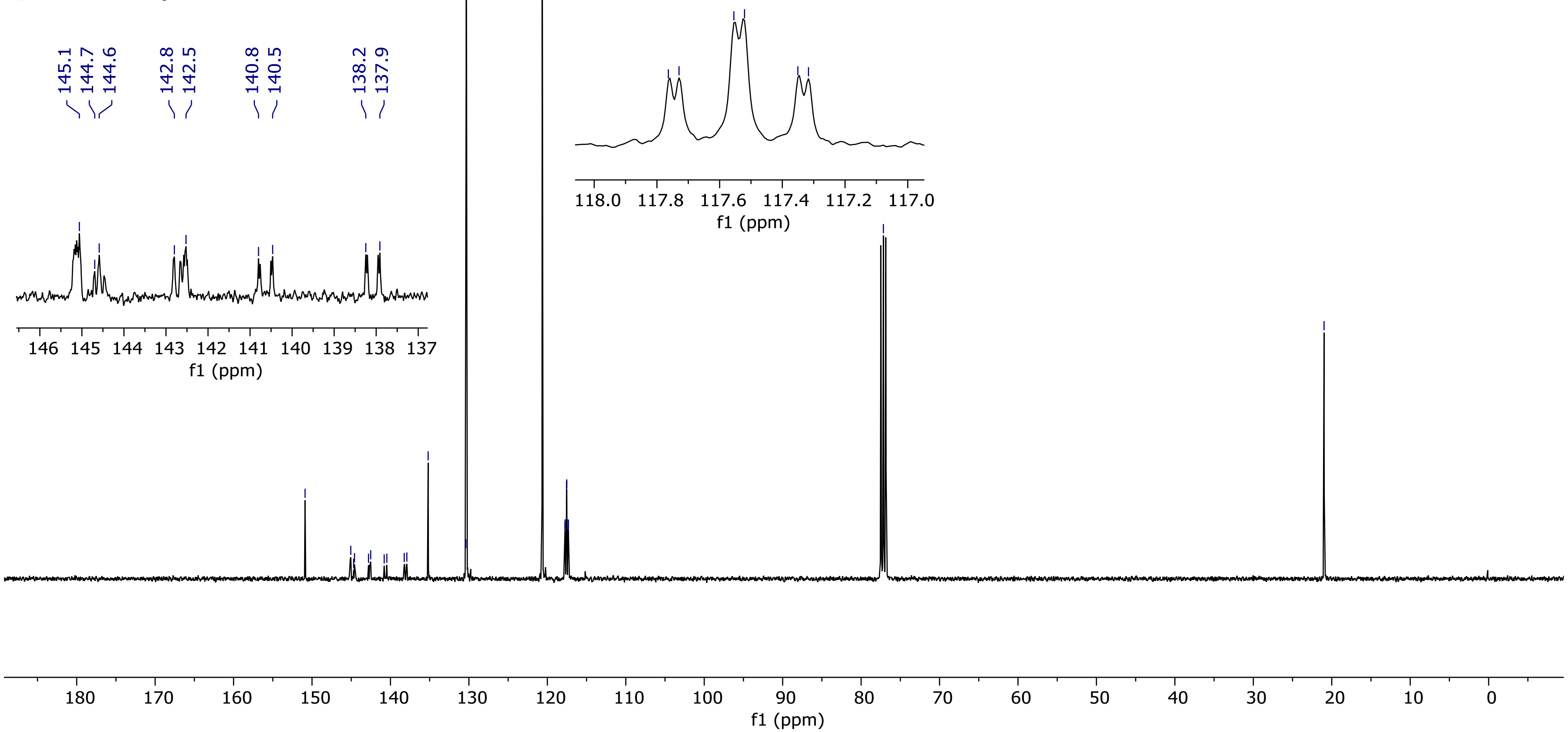

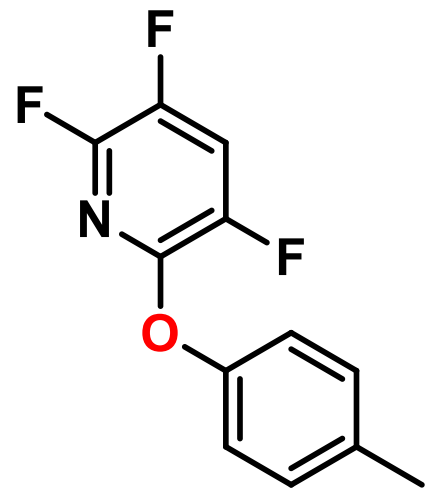

S7'

$^{19}F\{^1H\}$ NMR, $CDCl_3$

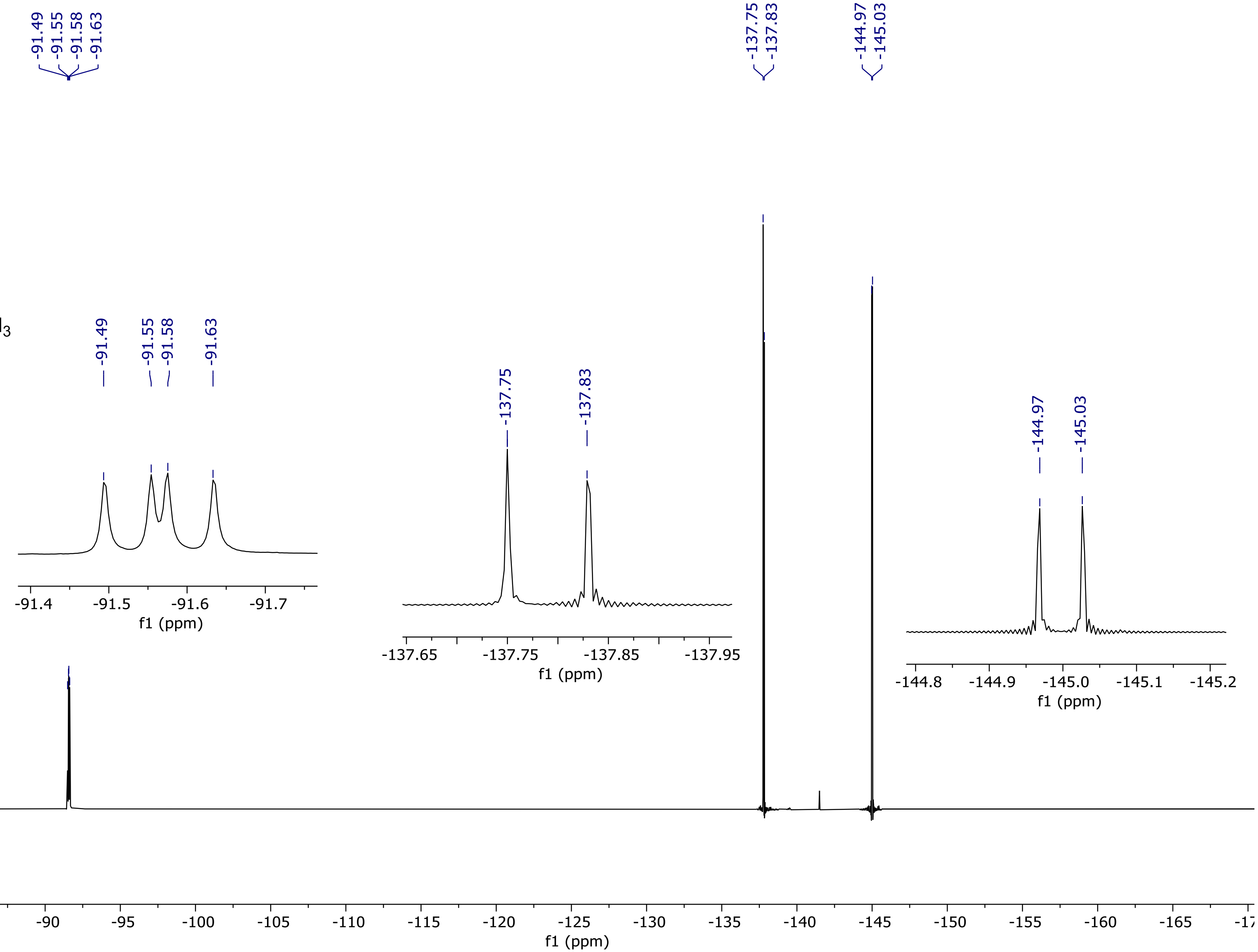

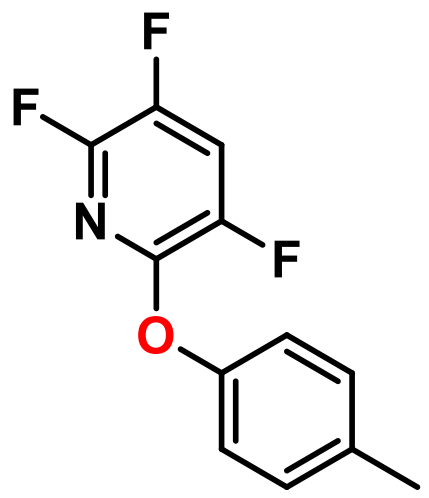

S7'

^{1}H NMR, C_6D_6

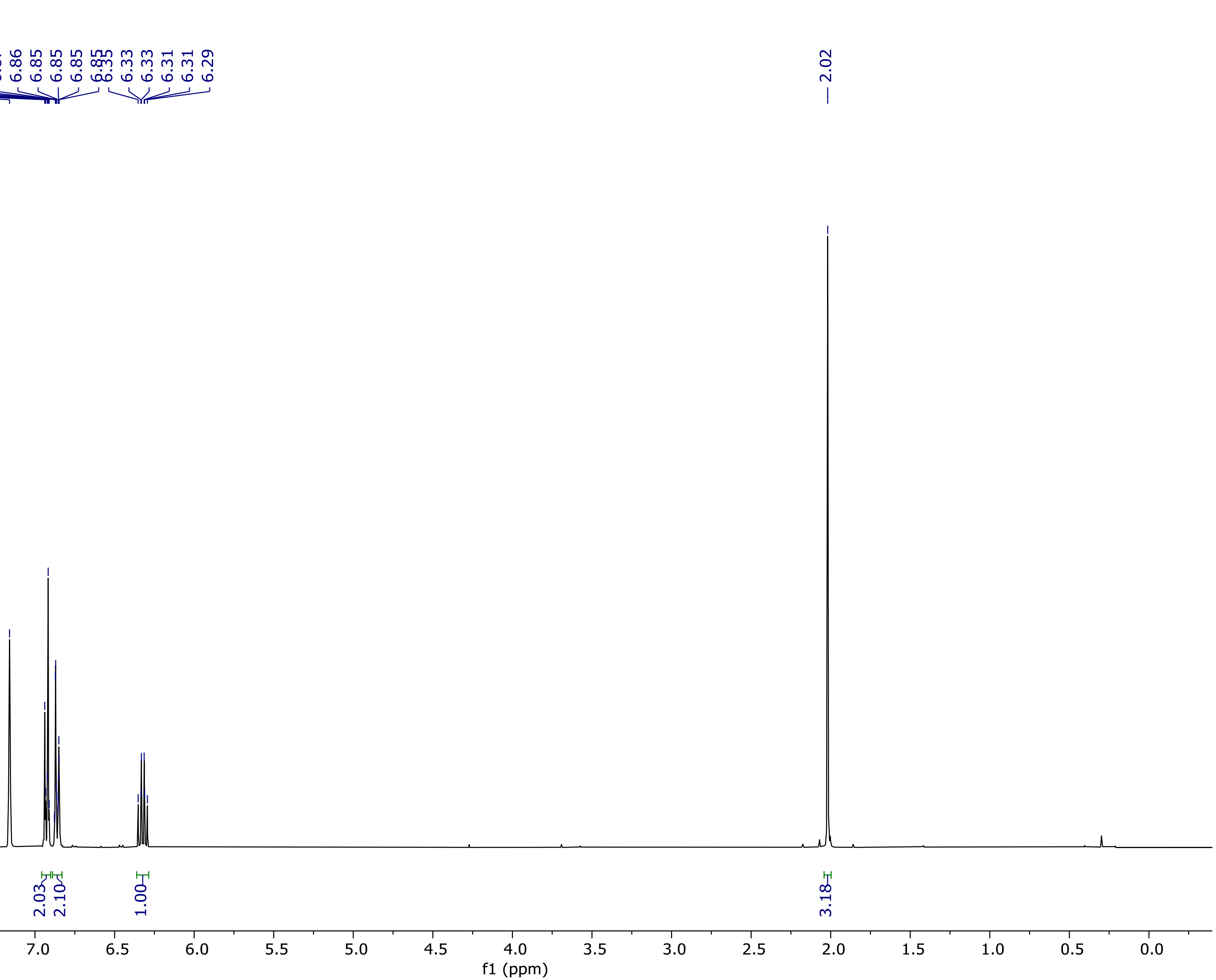

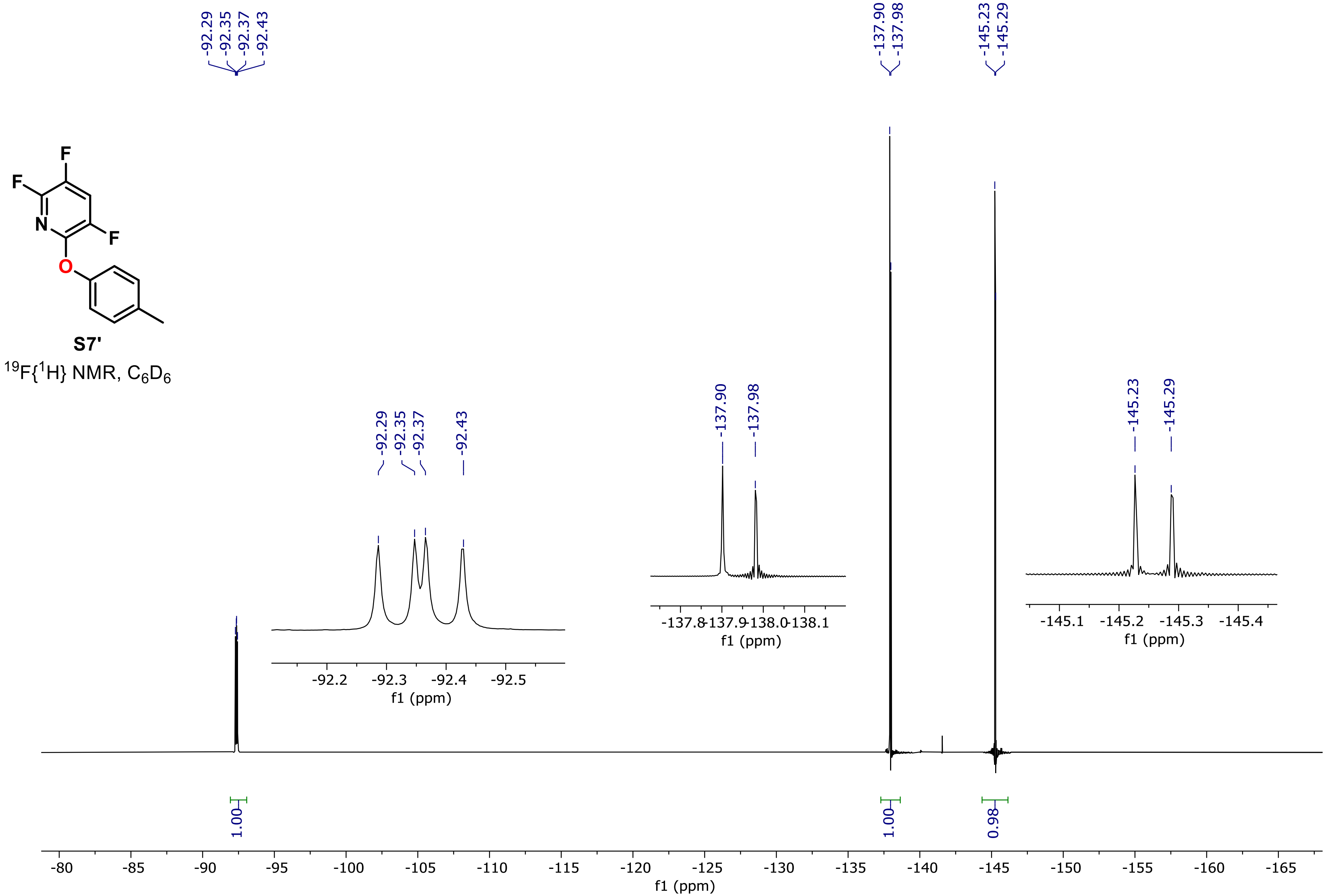

S7'
$^{19}F\{^{1}H\}$ NMR, C_6D_6
-92.29
-92.35
-92.37
-92.43
-137.90
-137.98
-145.23
-145.29
1.00
1.00
0.98
f1 (ppm)

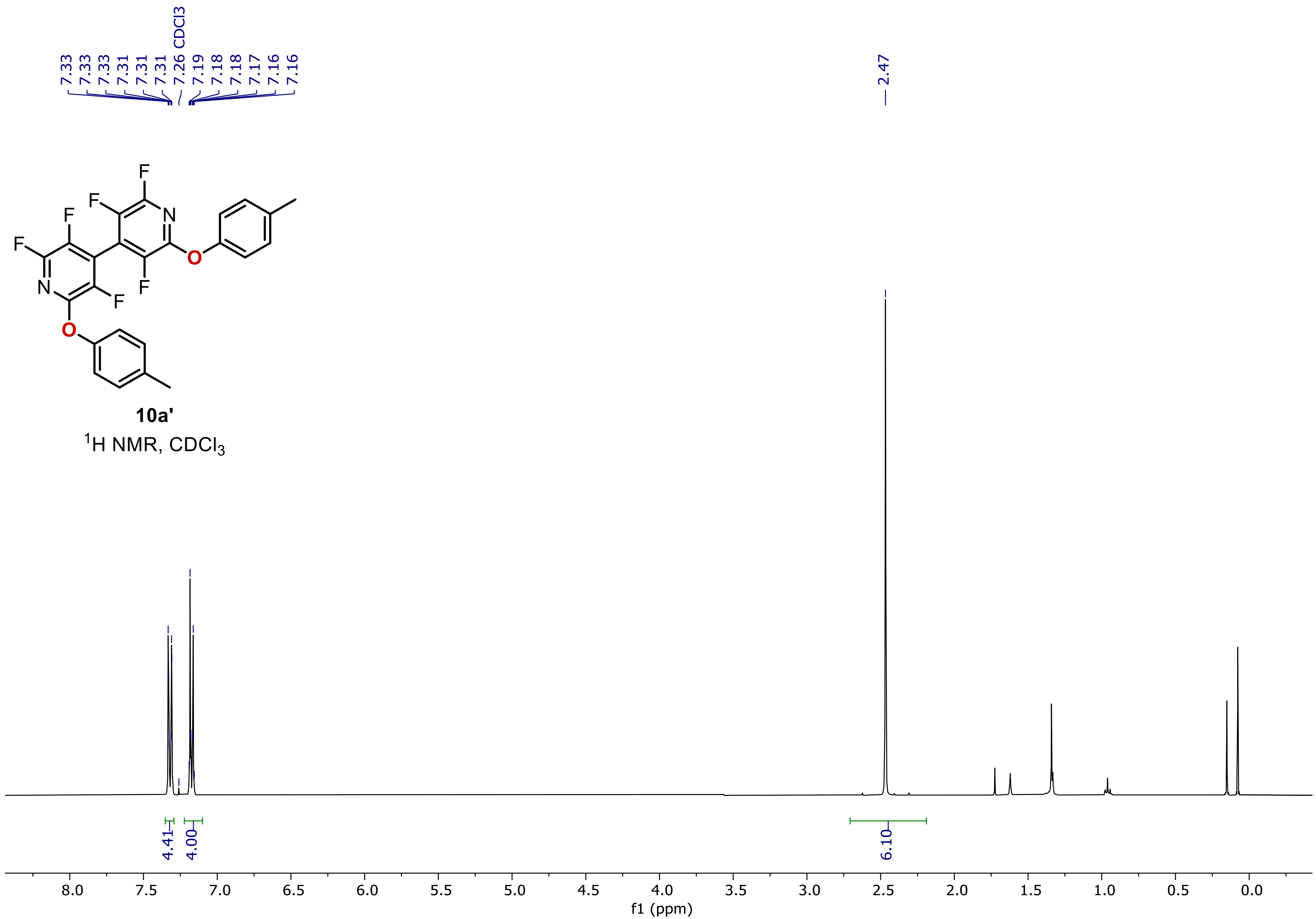

10a'

^{1}H NMR, $CDCl_3$

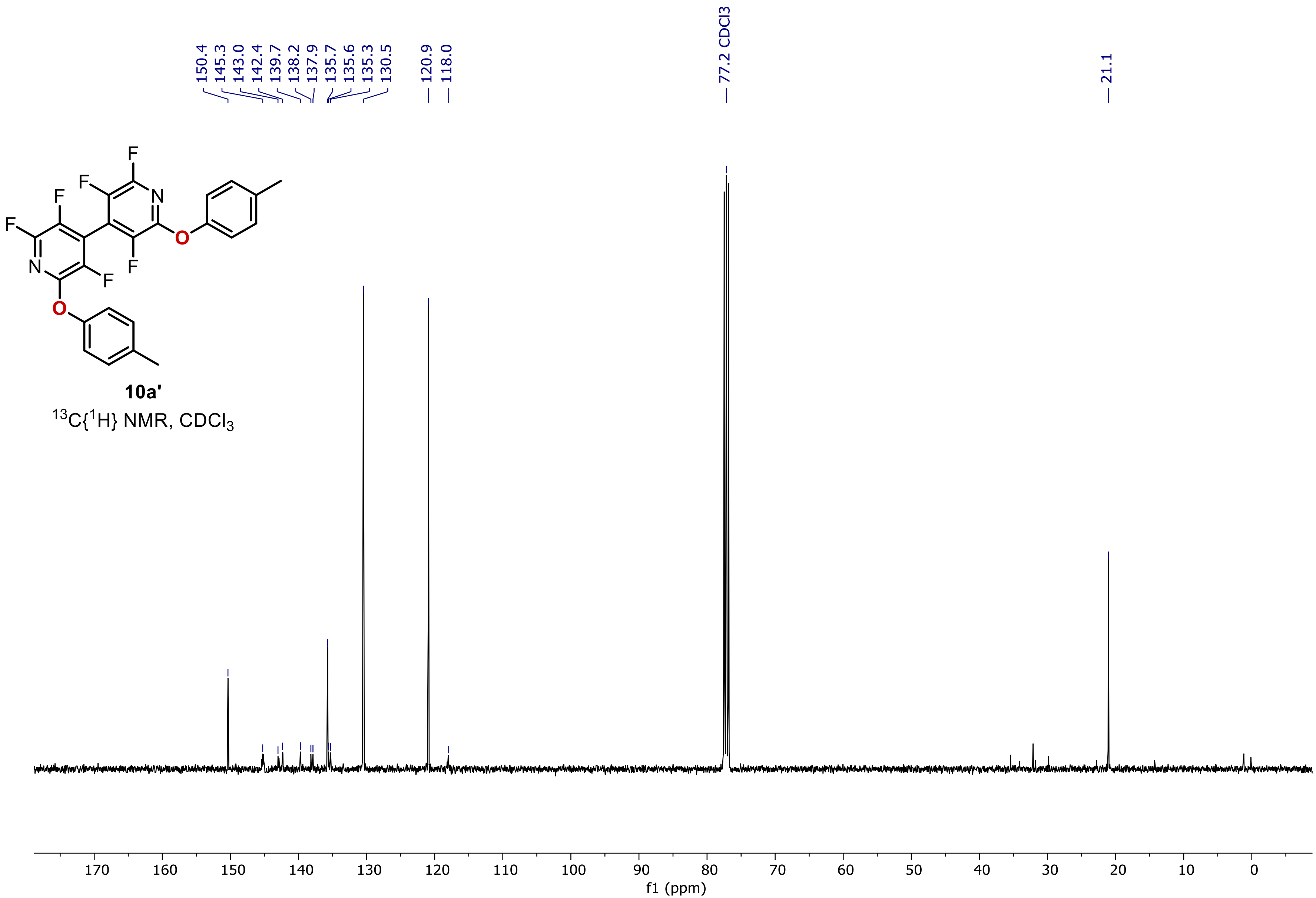

10a'

$^{13}C\{^{1}H\}$ NMR, $CDCl_3$

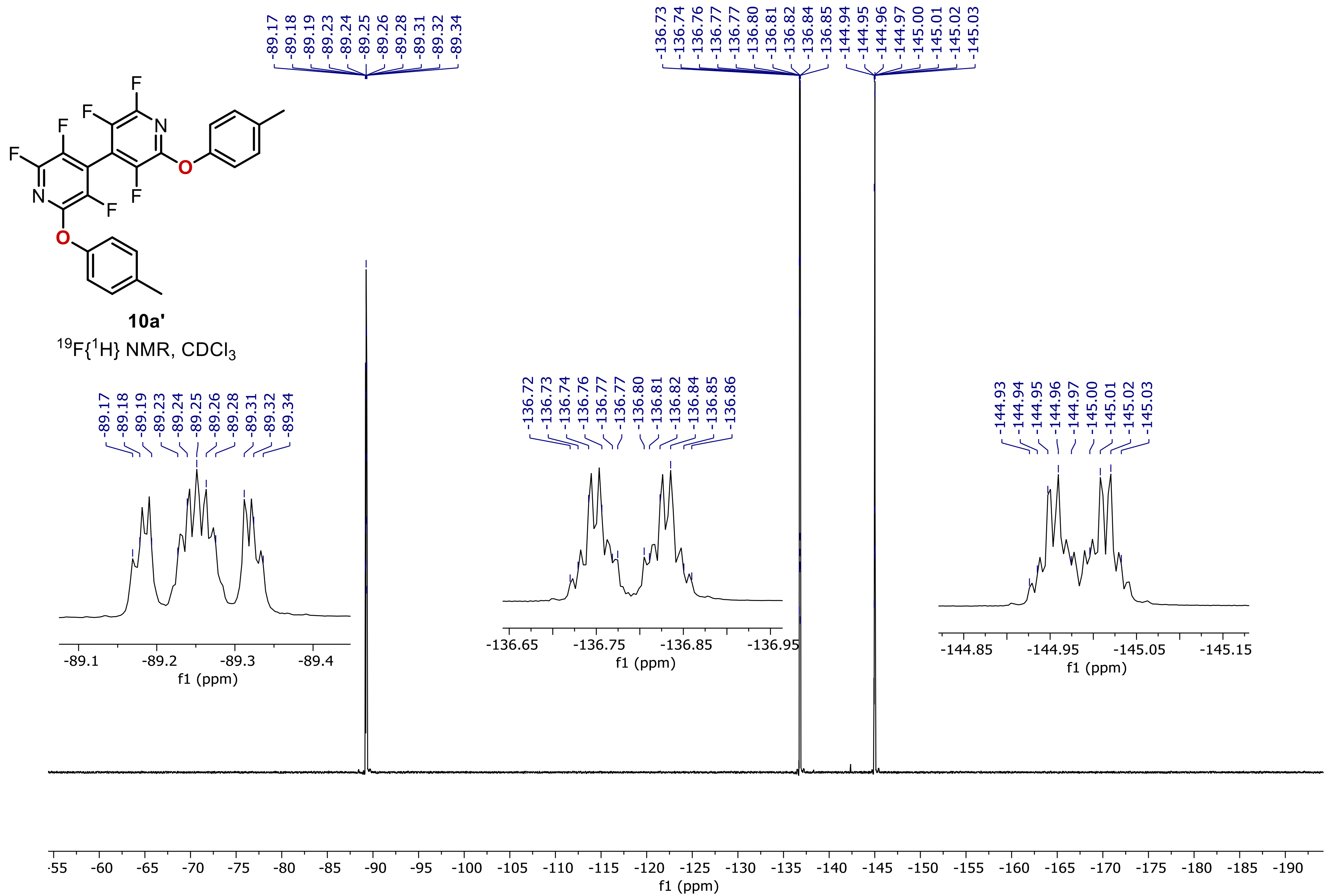
10a'
$^{19}F\{^1H\}$ NMR, $CDCl_3$
-89.17 -89.18 -89.19 -89.23 -89.24 -89.25 -89.26 -89.28 -89.31 -89.32 -89.34
-136.73 -136.74 -136.76 -136.77 -136.77 -136.80 -136.81 -136.82 -136.84 -136.85 -144.94 -144.95 -144.96 -144.97 -145.00 -145.01 -145.02 -145.03
-136.72 -136.73 -136.74 -136.76 -136.77 -136.77 -136.80 -136.81 -136.82 -136.84 -136.85 -136.86
-144.93 -144.94 -144.95 -144.96 -144.97 -145.00 -145.01 -145.02 -145.03
f1 (ppm)

7.2 NMR characterization of defluorothiolation products

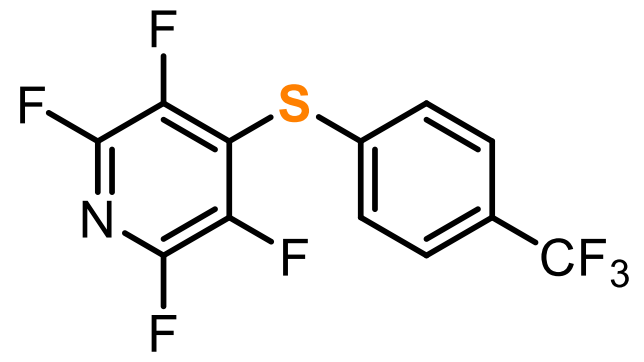

6c

^{1}H NMR, $CDCl_3$

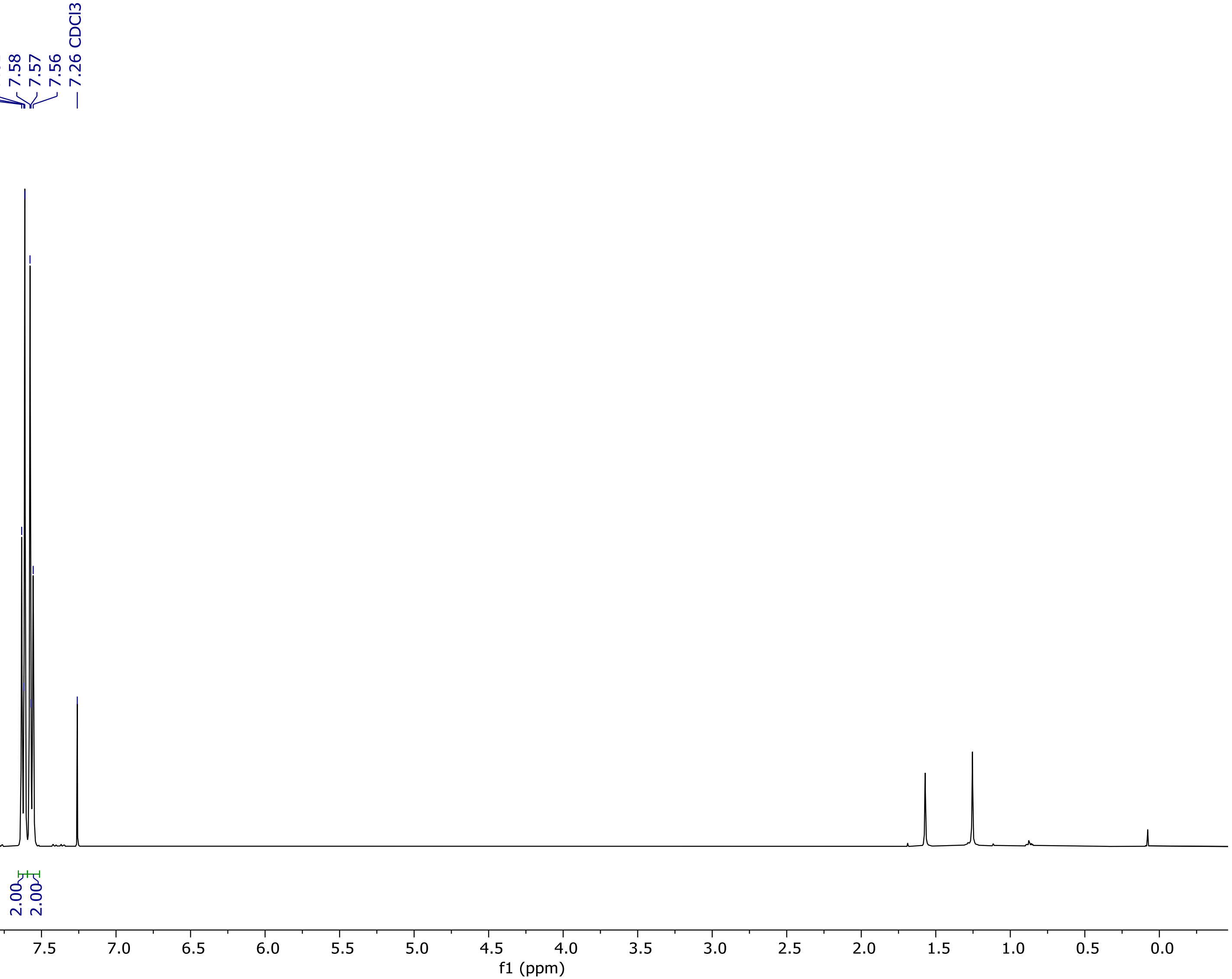

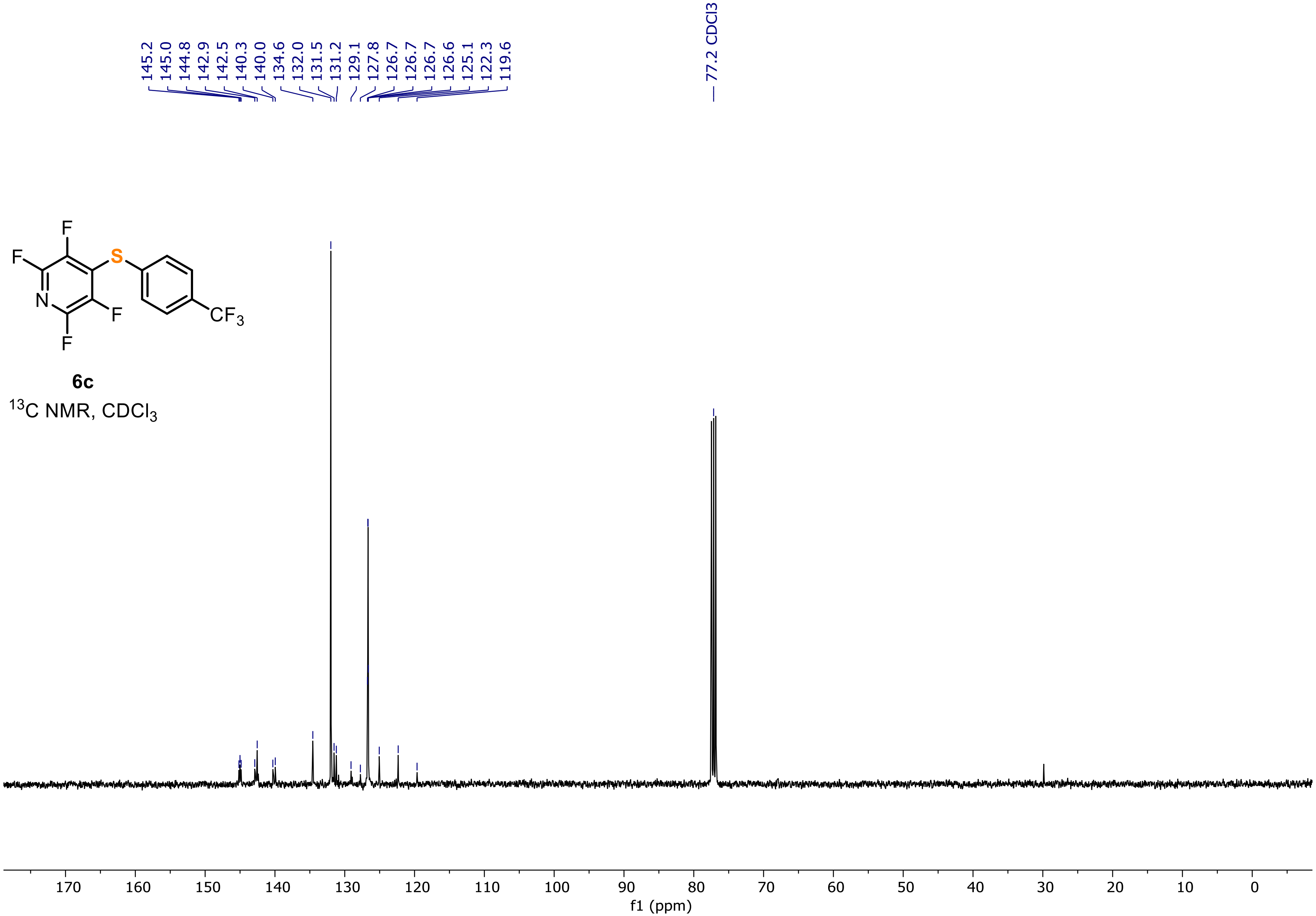
145.2
145.0
144.8
142.9
142.5
140.3
140.0
134.6
132.0
131.5
131.2
129.1
127.8
126.7
126.7
126.7
126.6
125.1
122.3
119.6
77.2 CDCl3
F
F
S
N
F
F
CF3
6c
13C NMR, CDCl3
170
160
150
140
130
120
110
100
90
80
70
60
50
40
30
20
10
0
f1 (ppm)

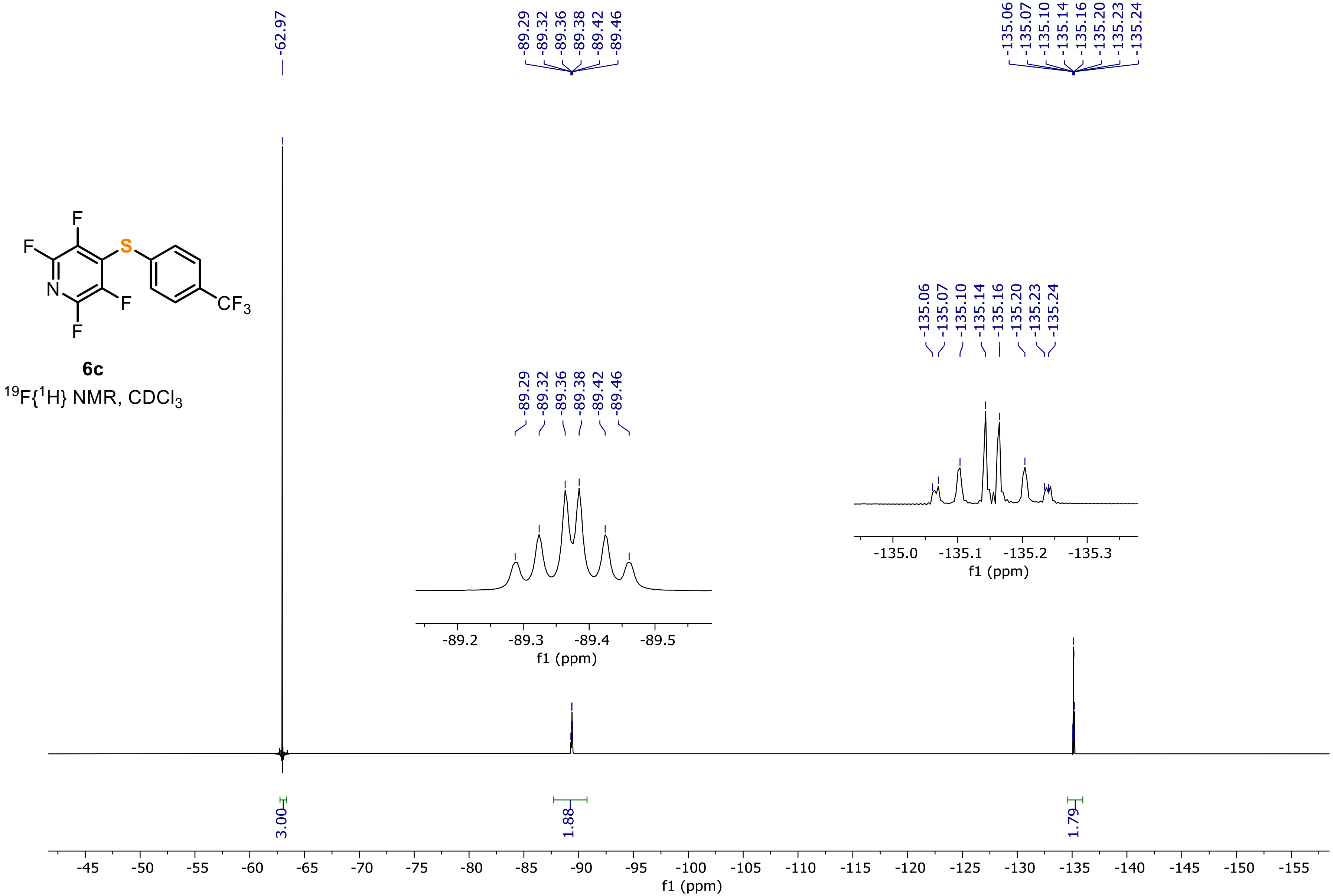

6c
$^{19}F\{^{1}H\}$ NMR, $CDCl_3$
-62.97
-89.29
-89.32
-89.36
-89.38
-89.42
-89.46
-135.06
-135.07
-135.10
-135.14
-135.16
-135.20
-135.23
-135.24
3.00
1.88
1.79
f1 (ppm)

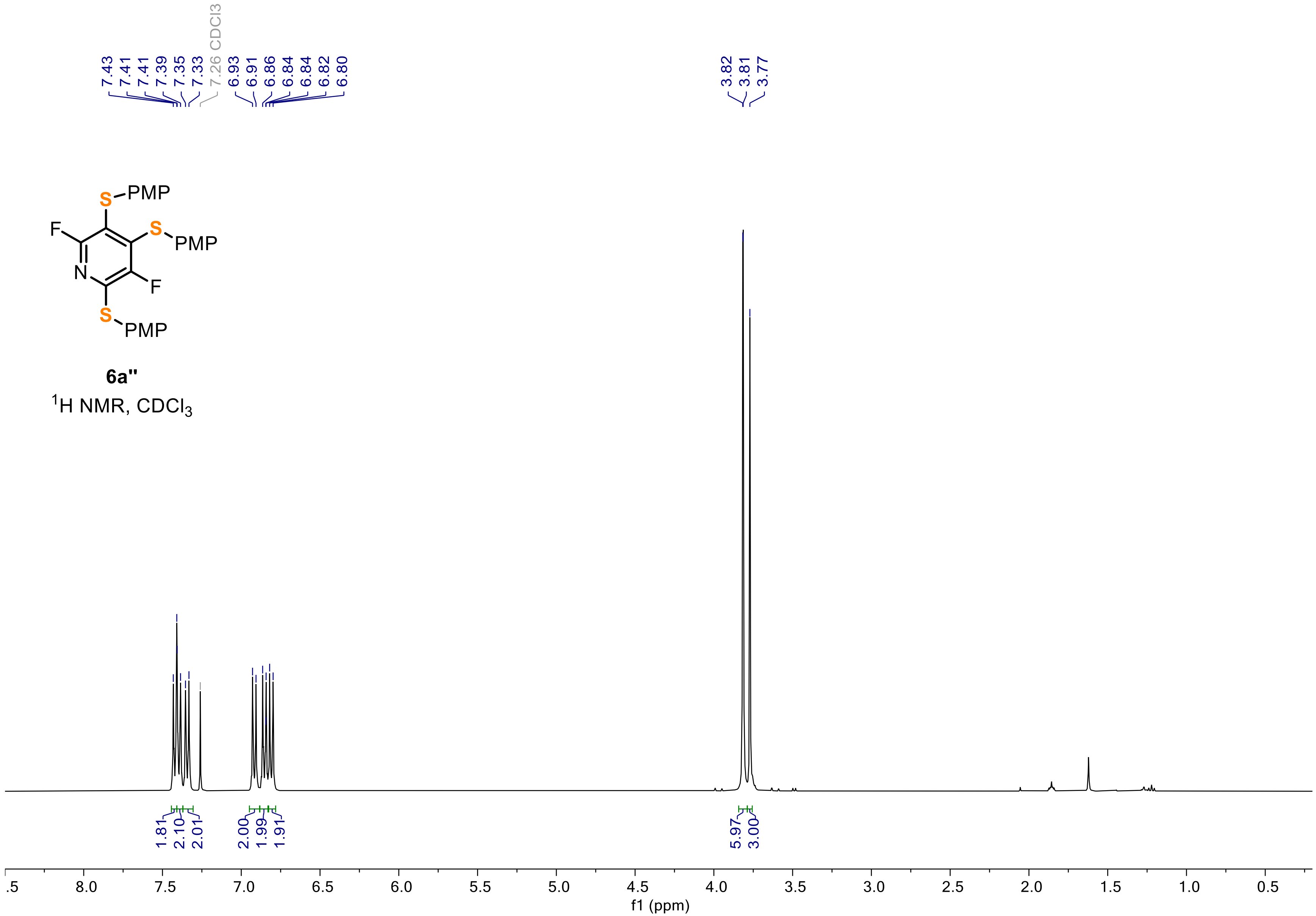

7.43
7.41
7.41
7.39
7.35
7.33
7.26 CDCl3
6.93
6.91
6.86
6.84
6.84
6.82
6.80
3.82
3.81
3.77
S
PMP
F
S
PMP
N
F
S
PMP
6a''
^{1}H NMR, CDCl$_3$
1.81
2.10
2.01
2.00
1.99
1.91
5.97
3.00
.5
8.0
7.5
7.0
6.5
6.0
5.5
5.0
4.5
4.0
3.5
3.0
2.5
2.0
1.5
1.0
0.5
f1 (ppm)

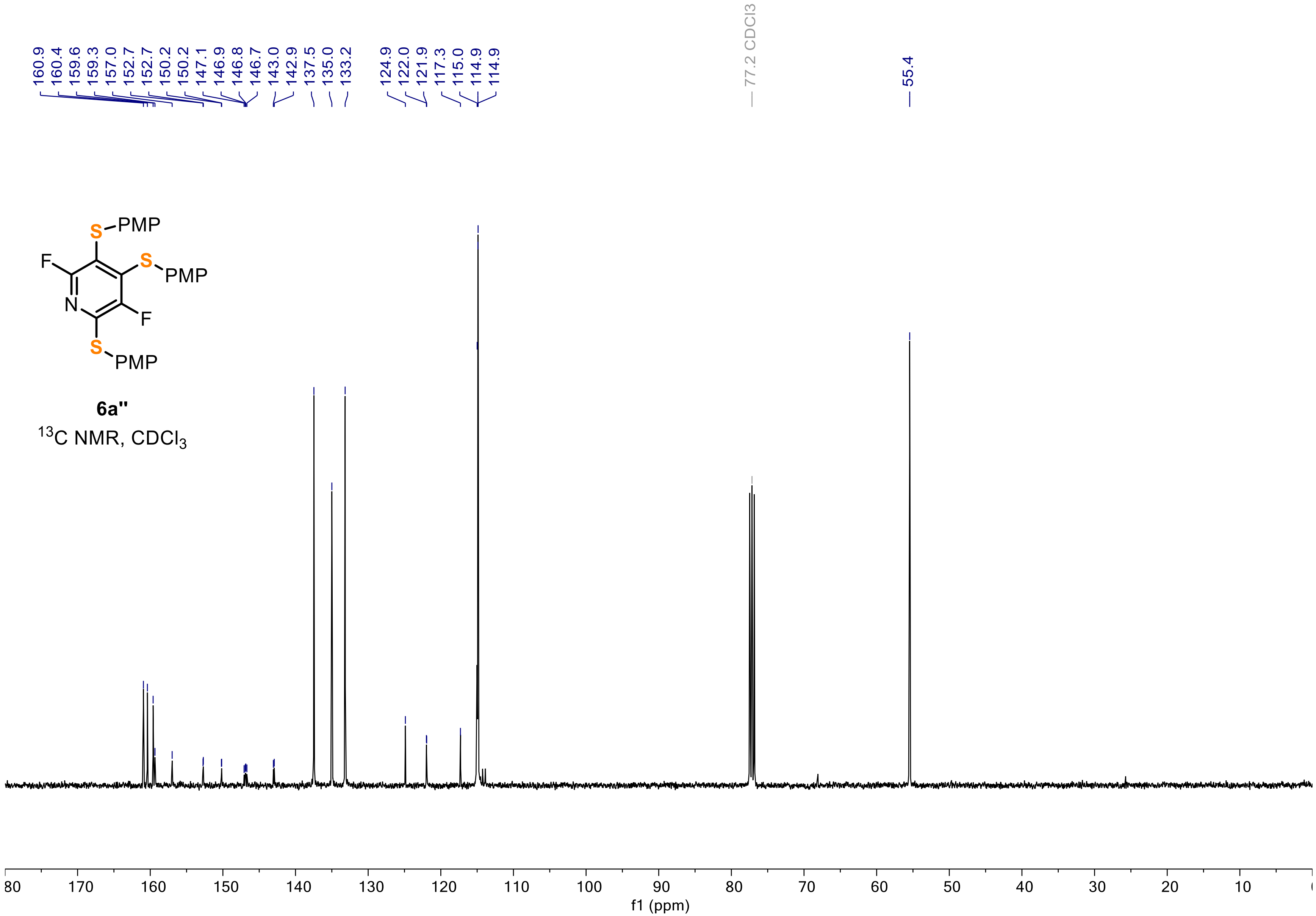
160.9
160.4
159.6
159.3
157.0
152.7
152.7
150.2
150.2
147.1
146.9
146.8
146.7
143.0
142.9
137.5
135.0
133.2
124.9
122.0
121.9
117.3
115.0
114.9
114.9
77.2 CDCl3
55.4
S
PMP
F
S
PMP
N
F
S
PMP
6a''
^{13}C NMR, $CDCl_3$
180
170
160
150
140
130
120
110
100
90
80
70
60
50
40
30
20
10
f1 (ppm)

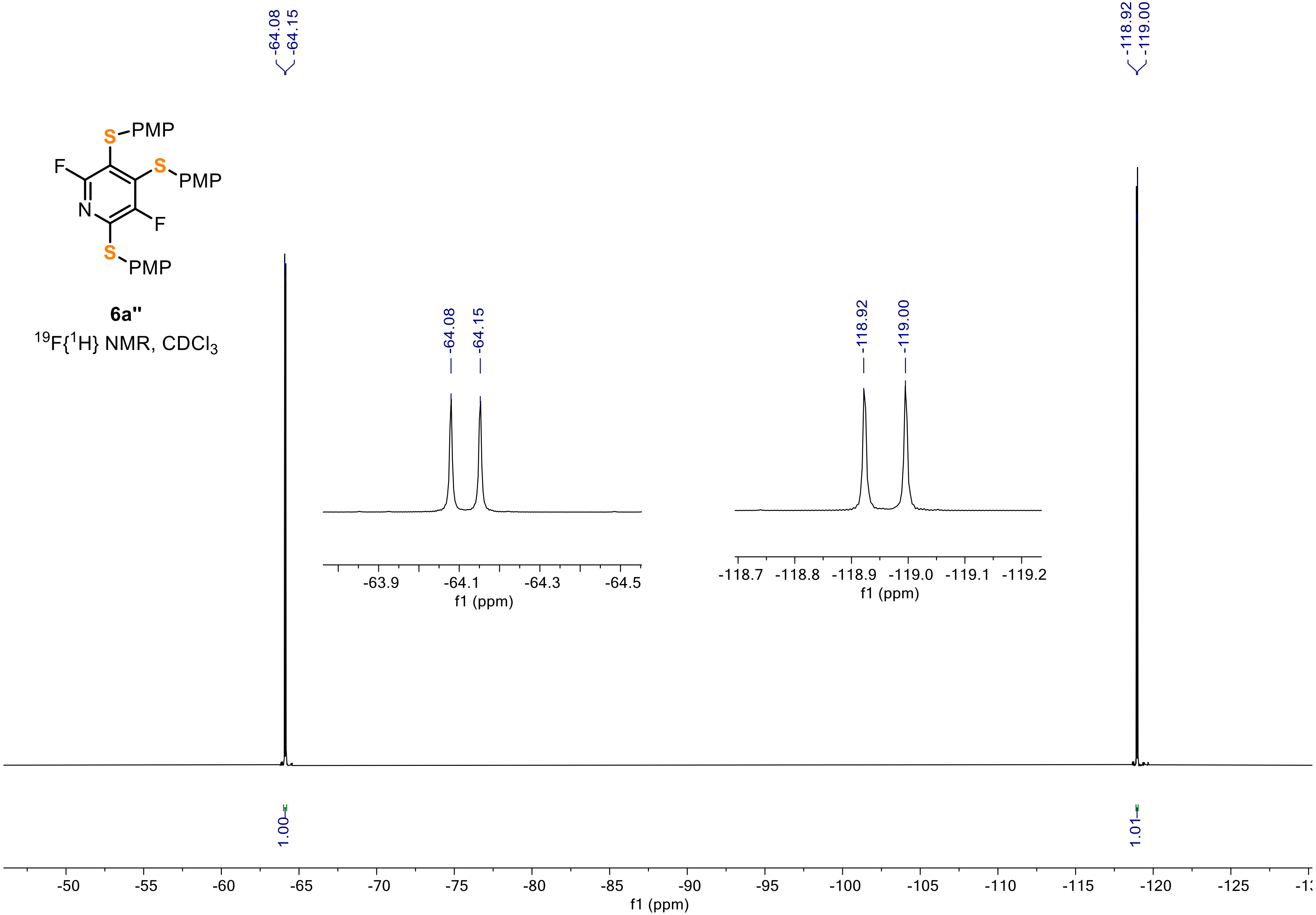

6a''

$^{19}F\{^{1}H\}$ NMR, $CDCl_3$

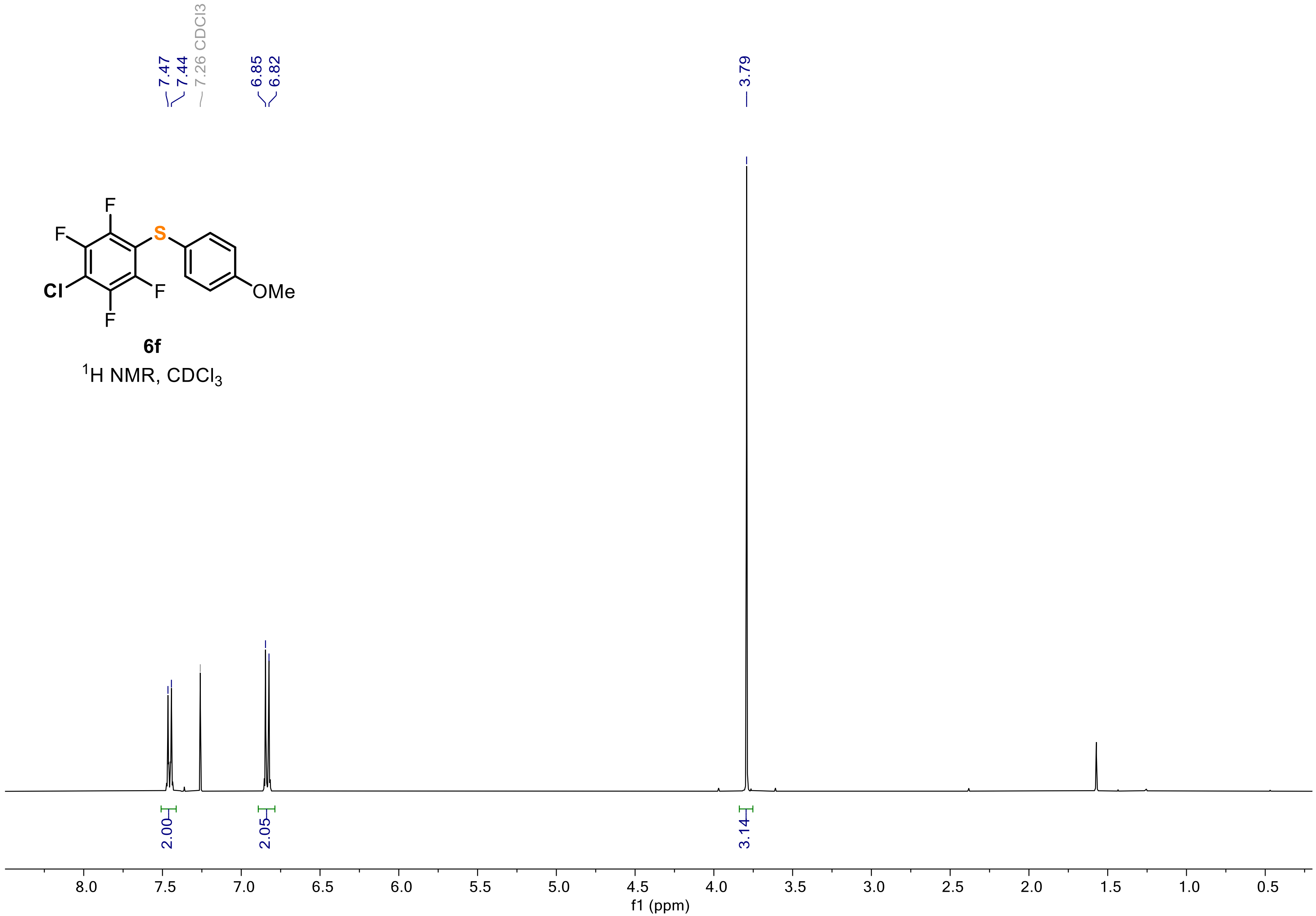
7.47
7.44
7.26 CDCl3
6.85
6.82
3.79
F
F
S
Cl
F
F
OMe
6f
^{1}H NMR, $CDCl_3$
2.00
2.05
3.14
8.0
7.5
7.0
6.5
6.0
5.5
5.0
4.5
4.0
3.5
3.0
2.5
2.0
1.5
1.0
0.5
f1 (ppm)

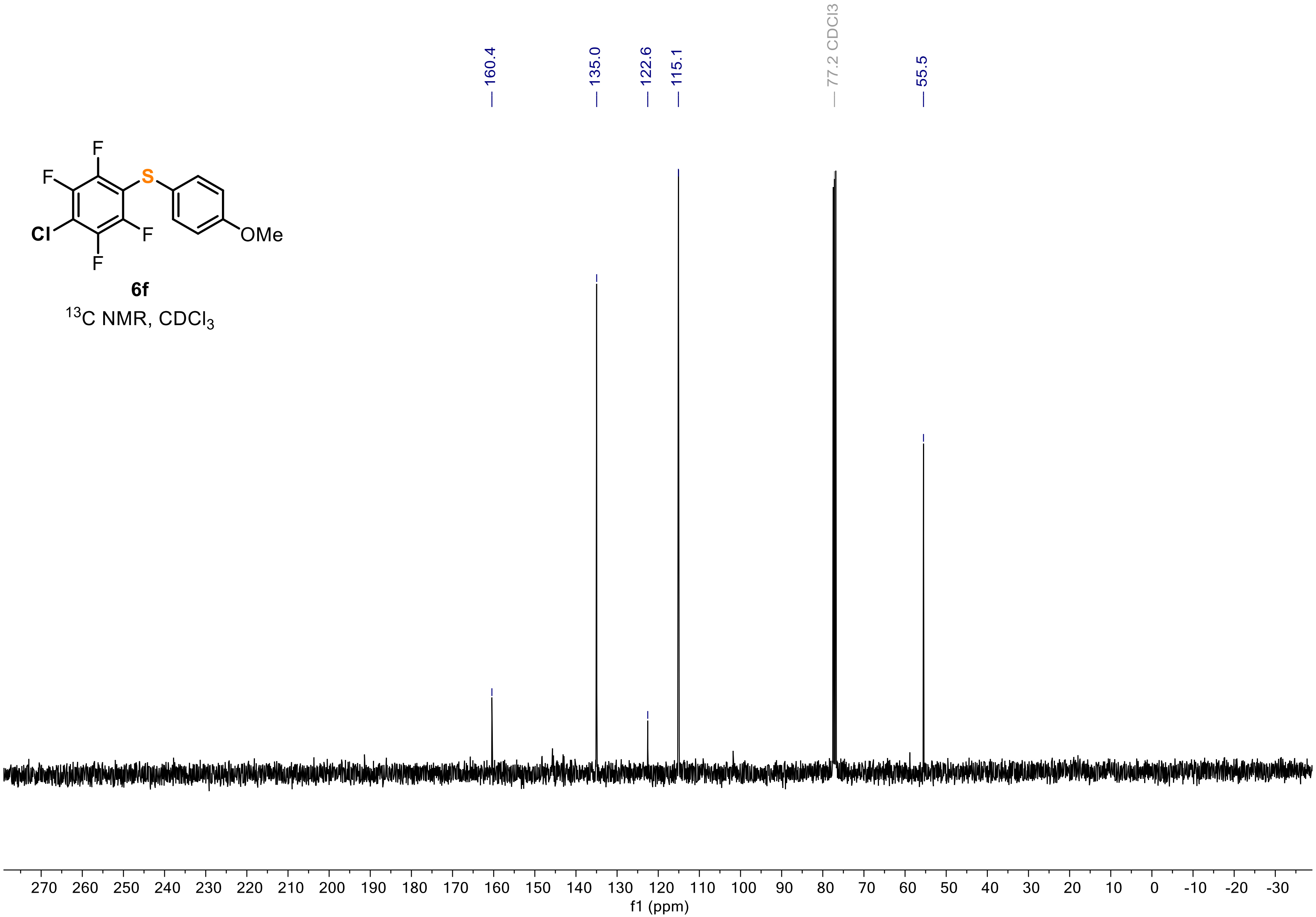

6f

^{13}C NMR, $CDCl_3$

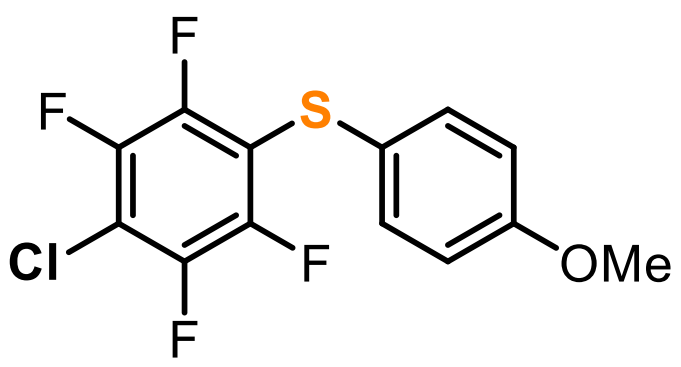

6f

$^{19}F\{^{1}H\}$ NMR, $CDCl_3$

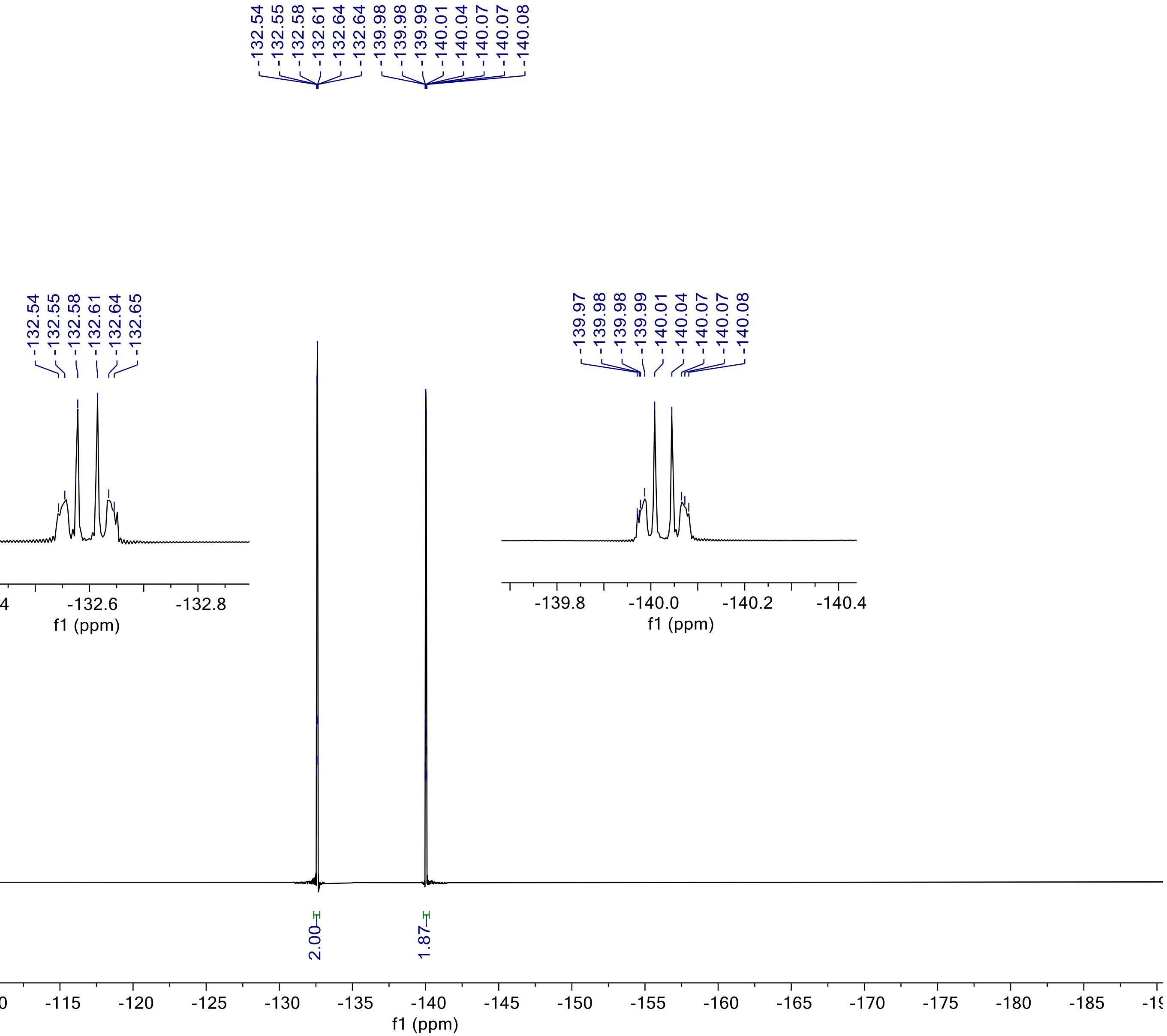

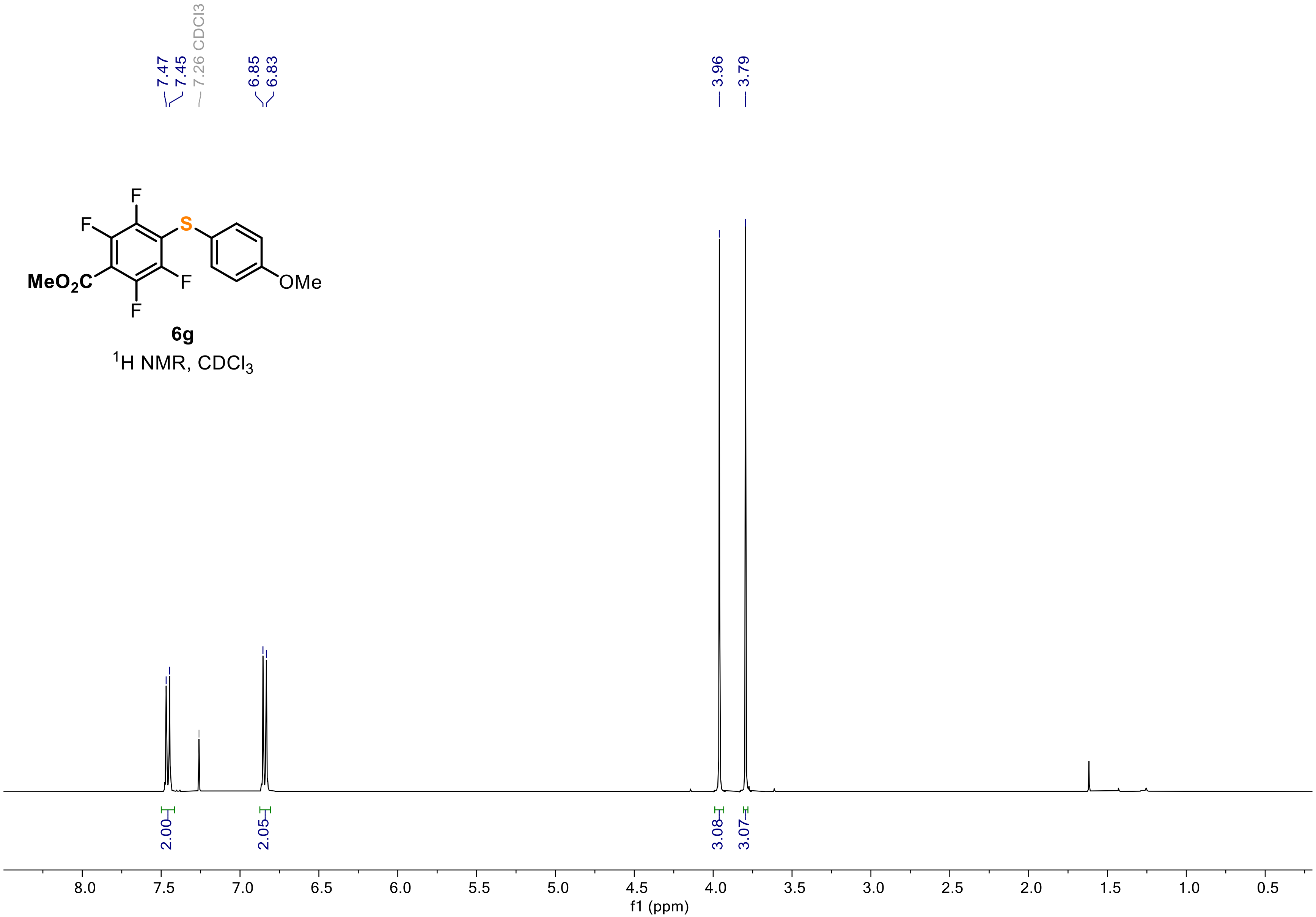

7.47
7.45
7.26 CDCl3
6.85
6.83
3.96
3.79
F
F
S
MeO2C
F
OMe
F
6g
1H NMR, CDCl3
2.00
2.05
3.08
3.07
8.0 7.5 7.0 6.5 6.0 5.5 5.0 4.5 4.0 3.5 3.0 2.5 2.0 1.5 1.0 0.5
f1 (ppm)

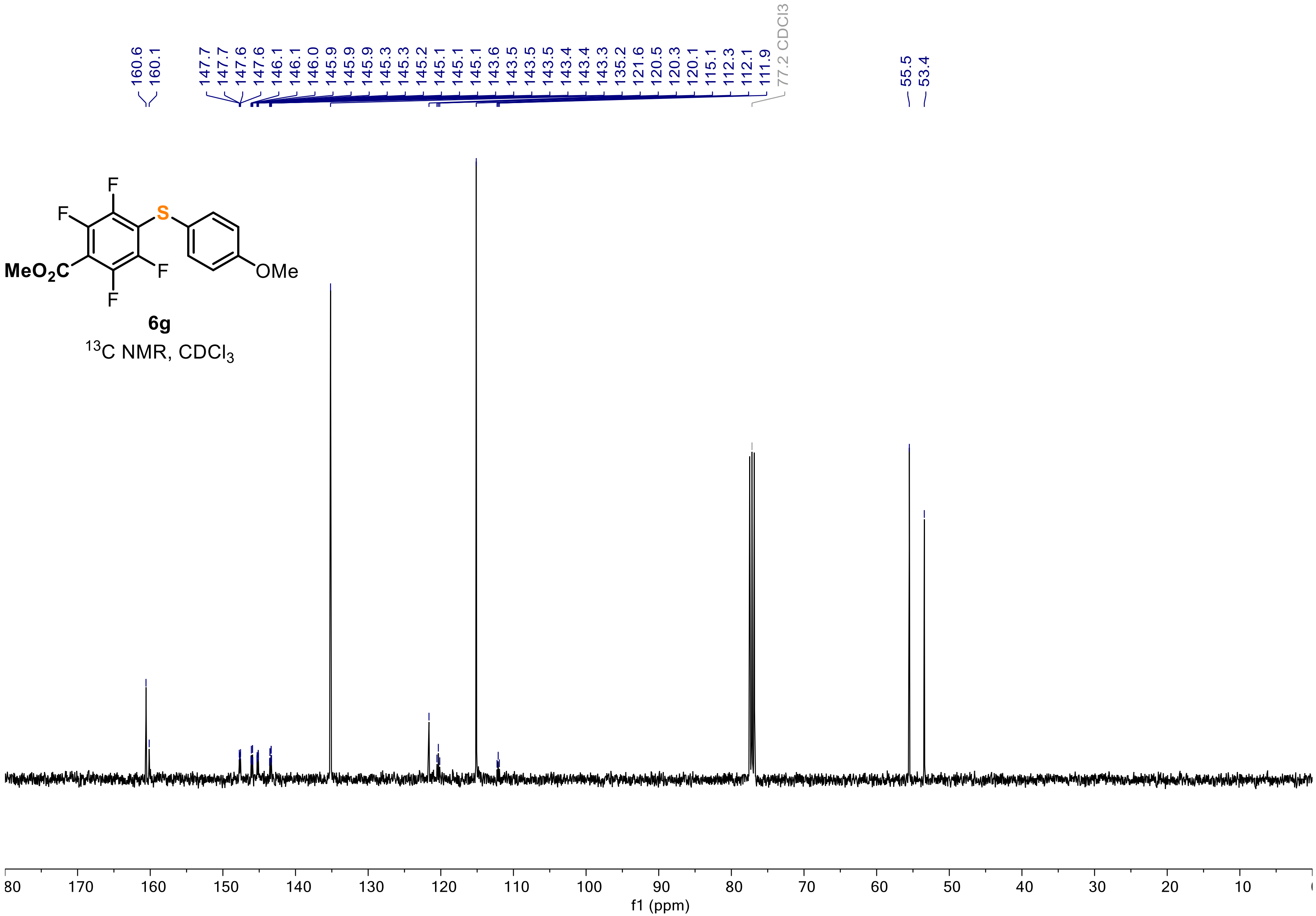
160.6
160.1
147.7
147.7
147.6
147.6
146.1
146.1
146.0
145.9
145.9
145.9
145.3
145.3
145.2
145.1
145.1
145.1
143.6
143.5
143.5
143.5
143.4
143.4
143.3
135.2
121.6
120.5
120.3
120.1
115.1
112.3
112.1
111.9
77.2 CDCl3
55.5
53.4
F
F
S
MeO2C
F
F
OMe
6g
13C NMR, CDCl3
180
170
160
150
140
130
120
110
100
90
80
70
60
50
40
30
20
10
f1 (ppm)

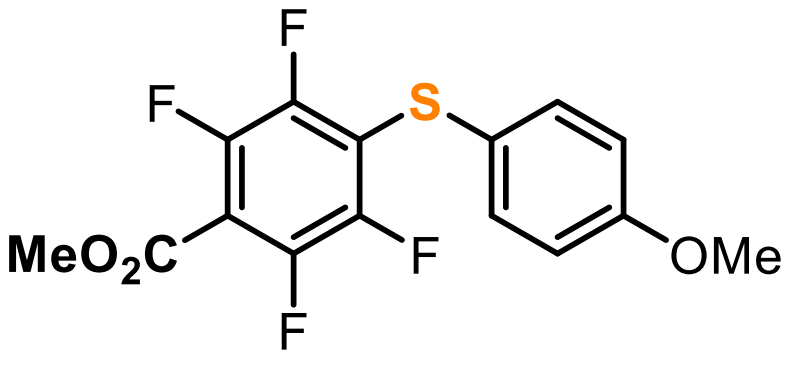

6g

$^{19}F\{^1H\}$ NMR, $CDCl_3$

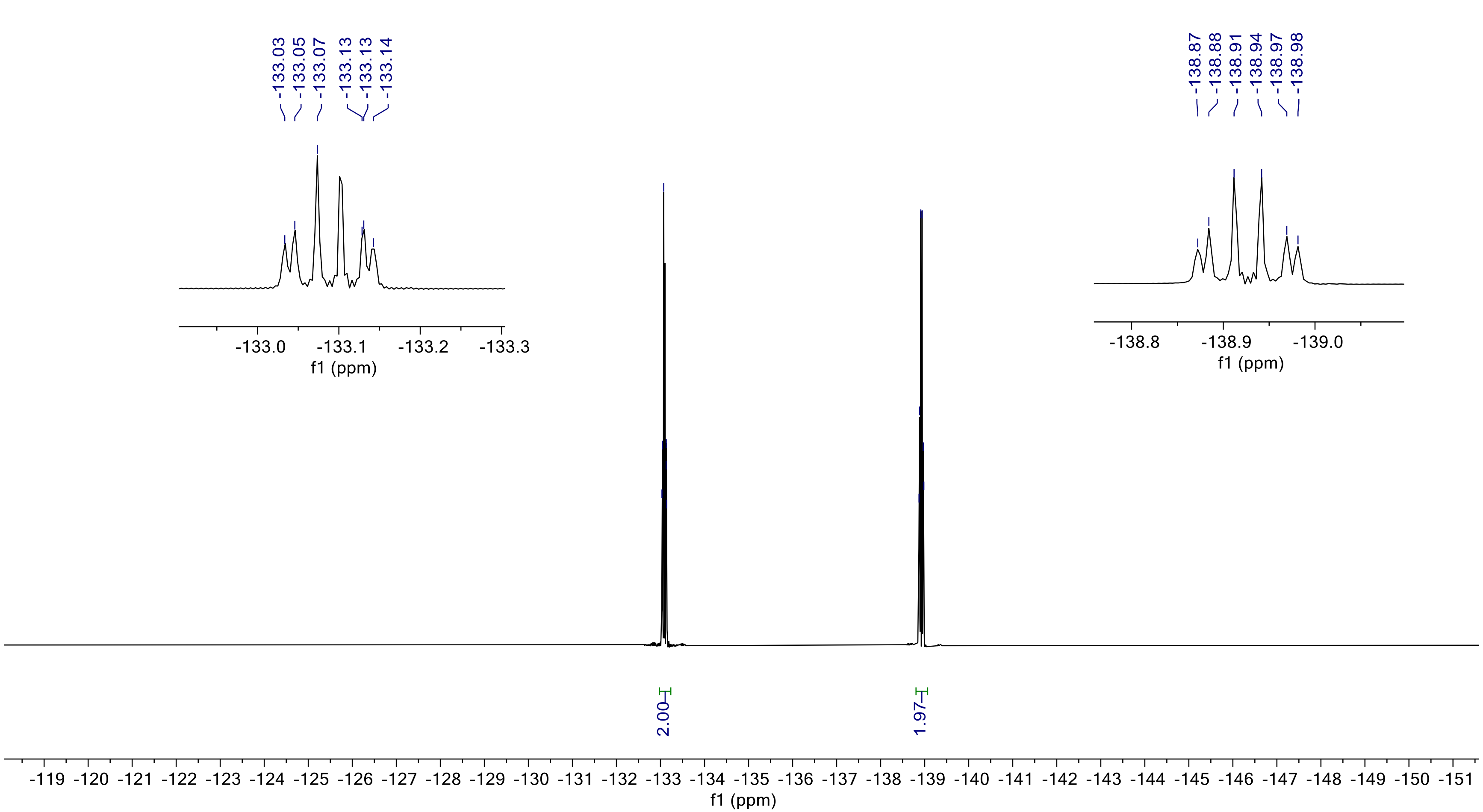

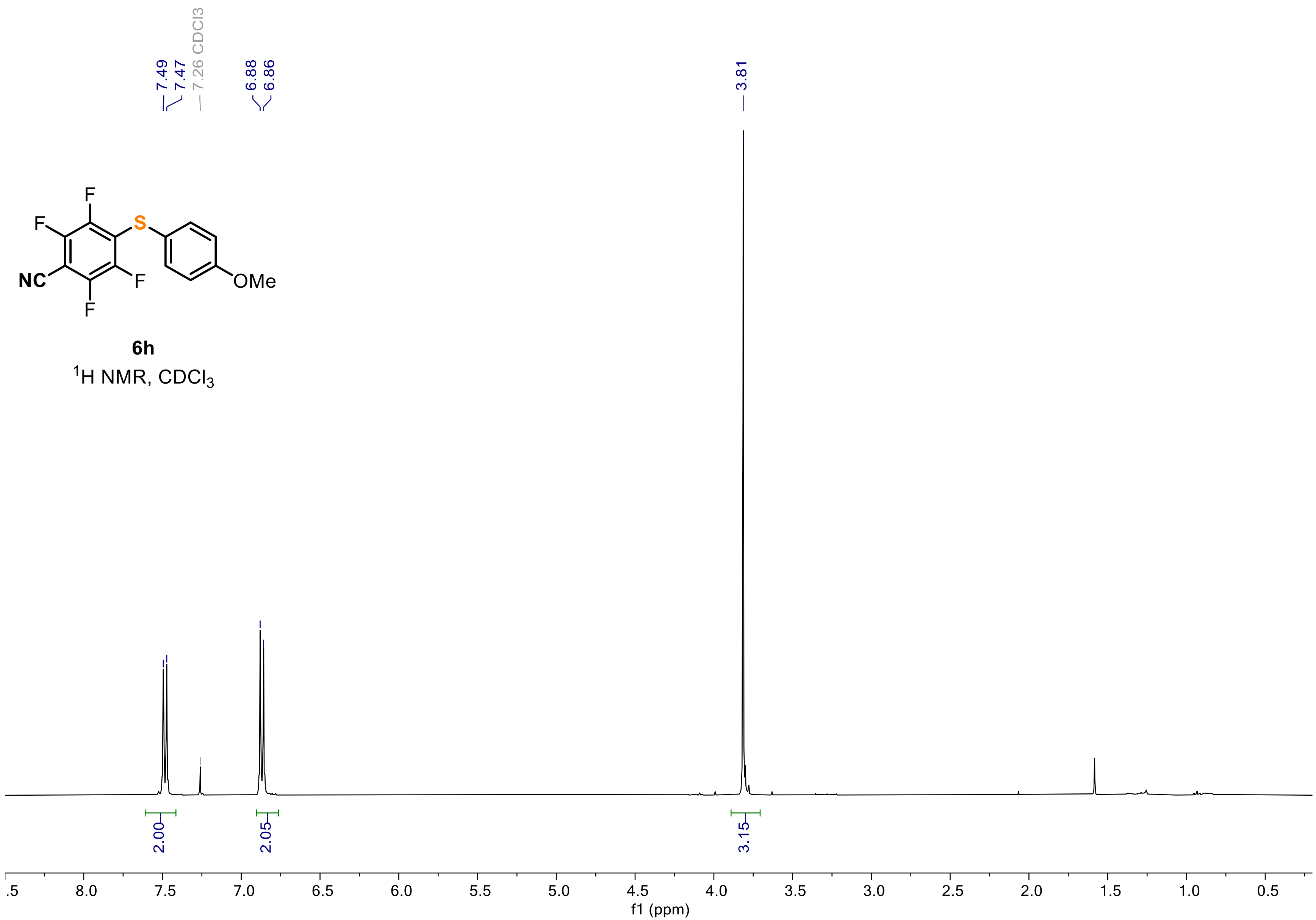

7.49
7.47
7.26 CDCl3
6.88
6.86
3.81
F
F
S
NC
F
OMe
F
6h
1H NMR, CDCl3
2.00
2.05
3.15
.5
8.0
7.5
7.0
6.5
6.0
5.5
5.0
4.5
4.0
3.5
3.0
2.5
2.0
1.5
1.0
0.5
f1 (ppm)

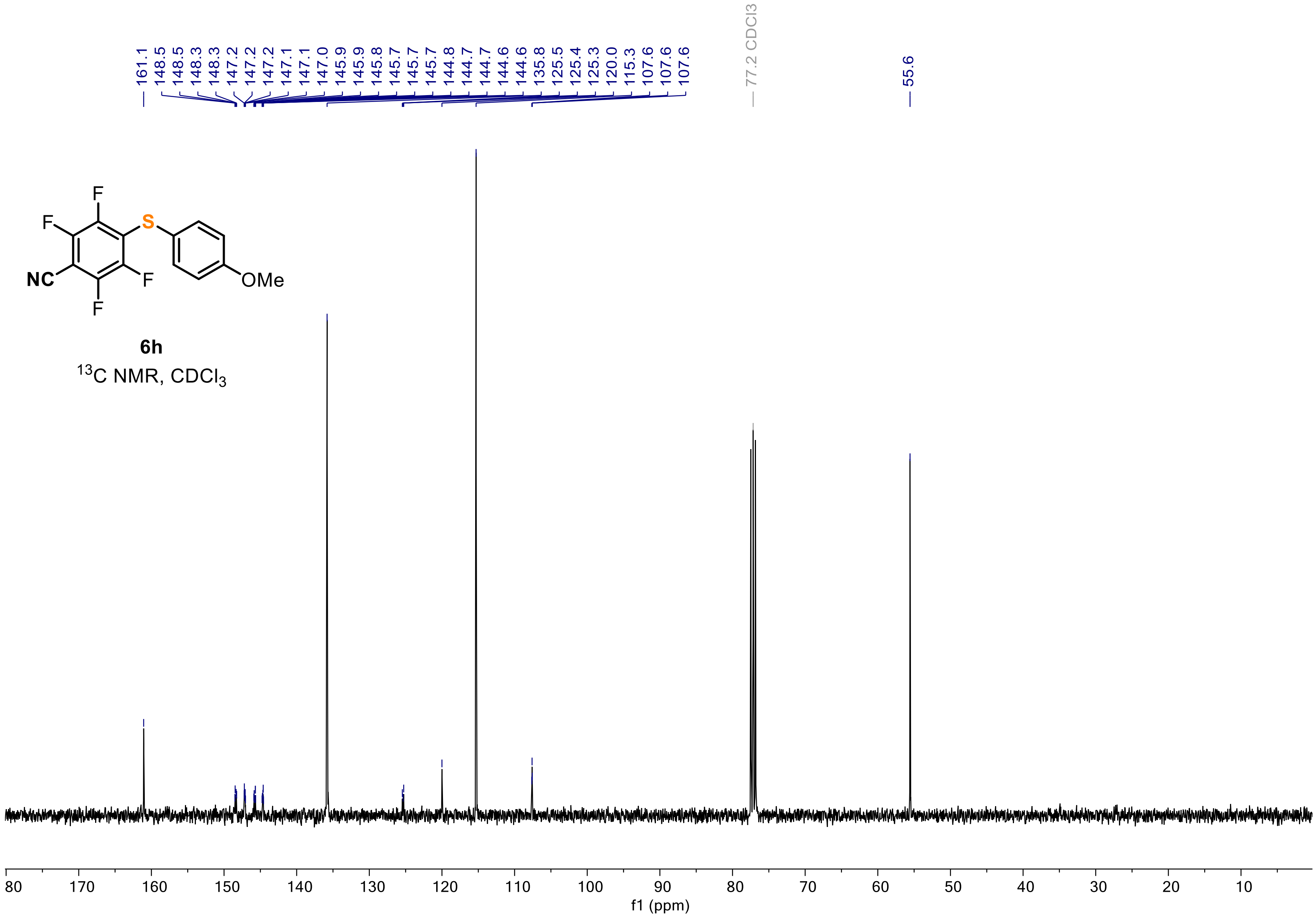
161.1
148.5
148.5
148.3
148.3
147.2
147.2
147.2
147.1
147.1
147.0
145.9
145.9
145.8
145.7
145.7
145.7
144.8
144.7
144.7
144.6
144.6
135.8
125.5
125.4
125.3
120.0
115.3
107.6
107.6
107.6
77.2 CDCl3
55.6
6h
13C NMR, CDCl3
f1 (ppm)

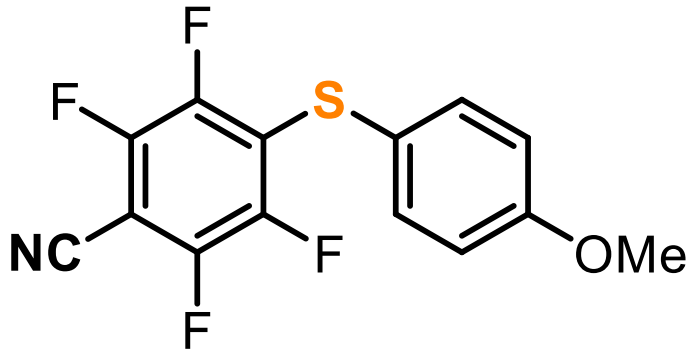

6h

$^{19}F\{^{1}H\}$ NMR, $CDCl_3$

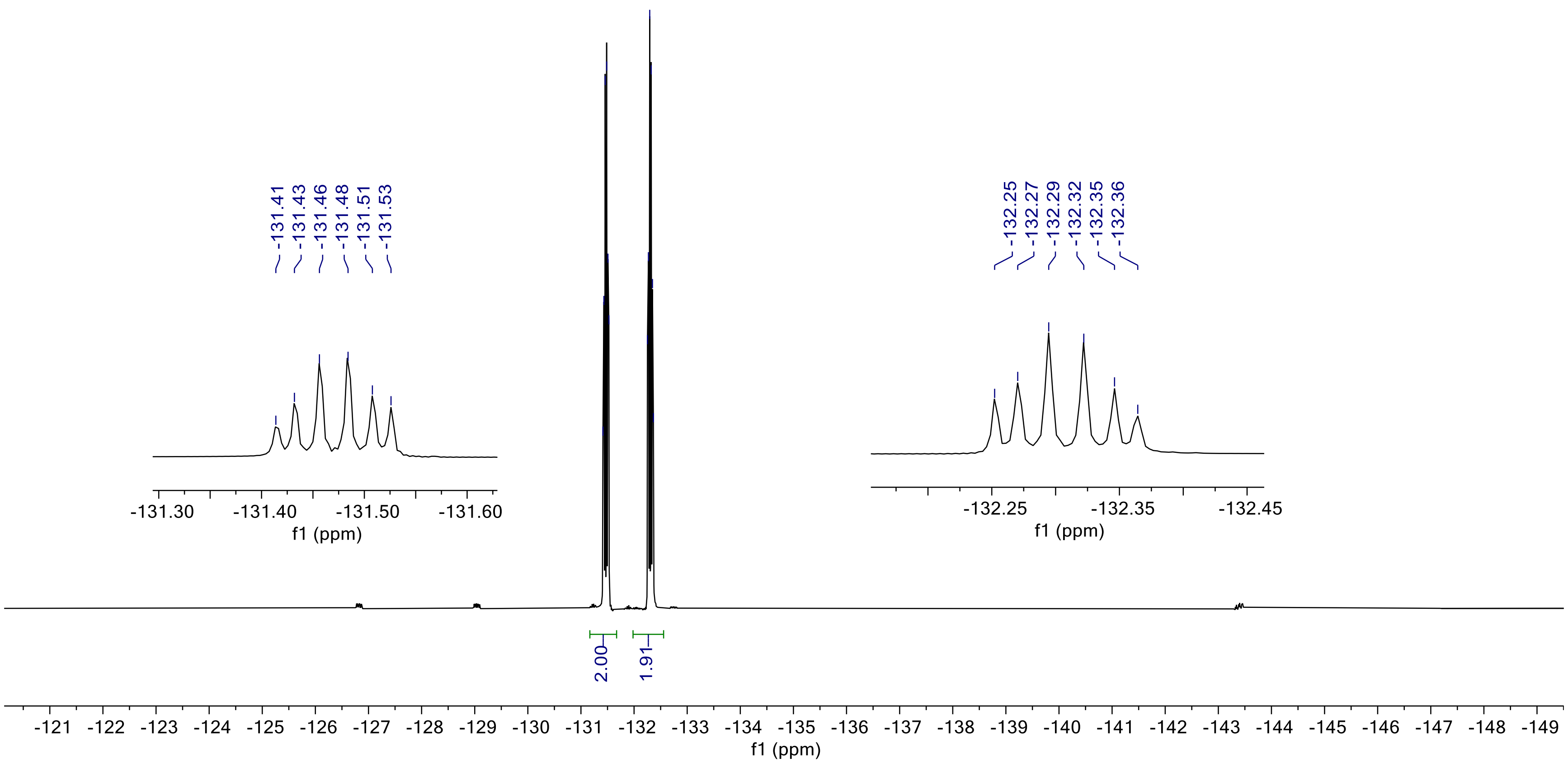

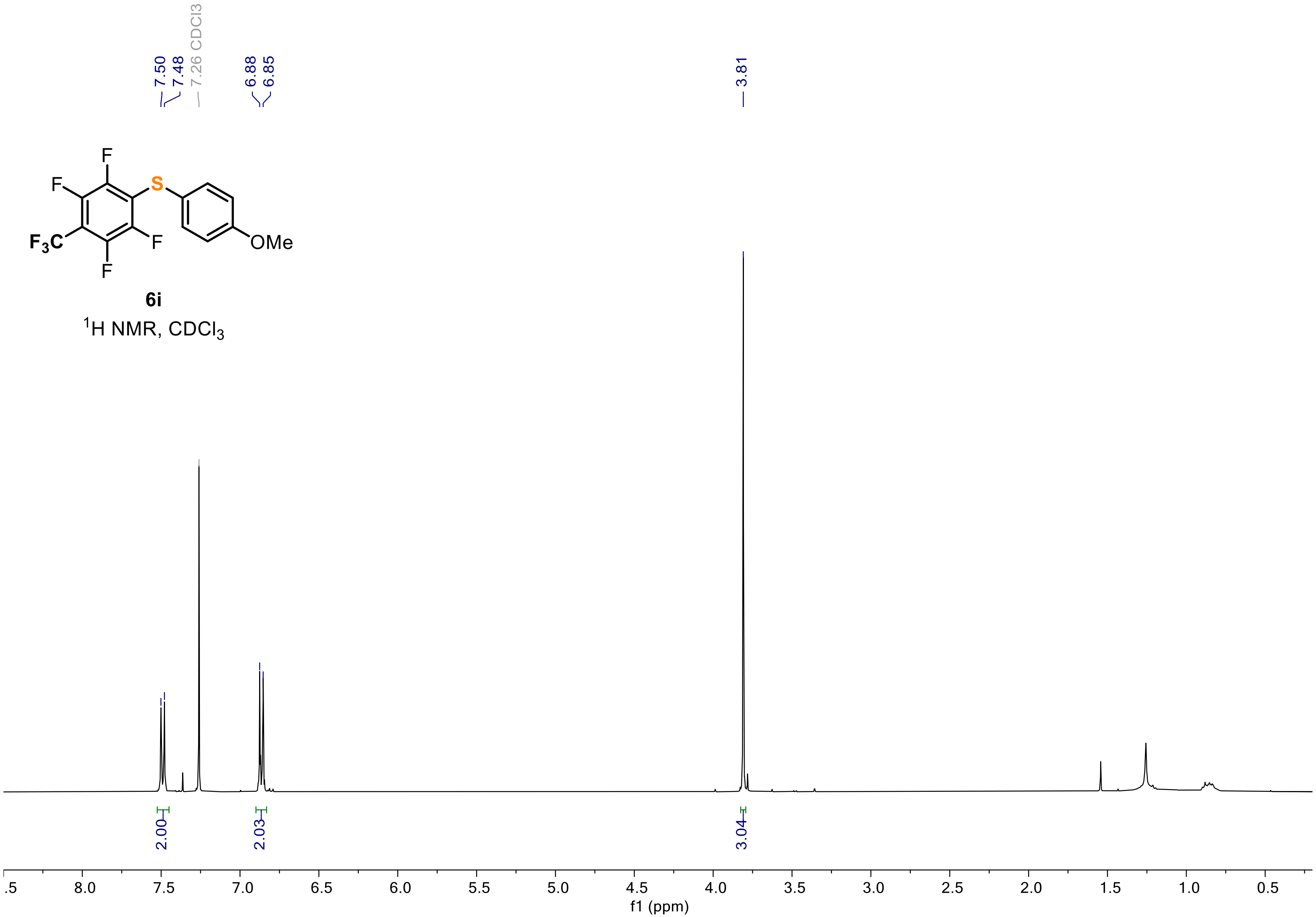

6i

^{1}H NMR, $CDCl_3$

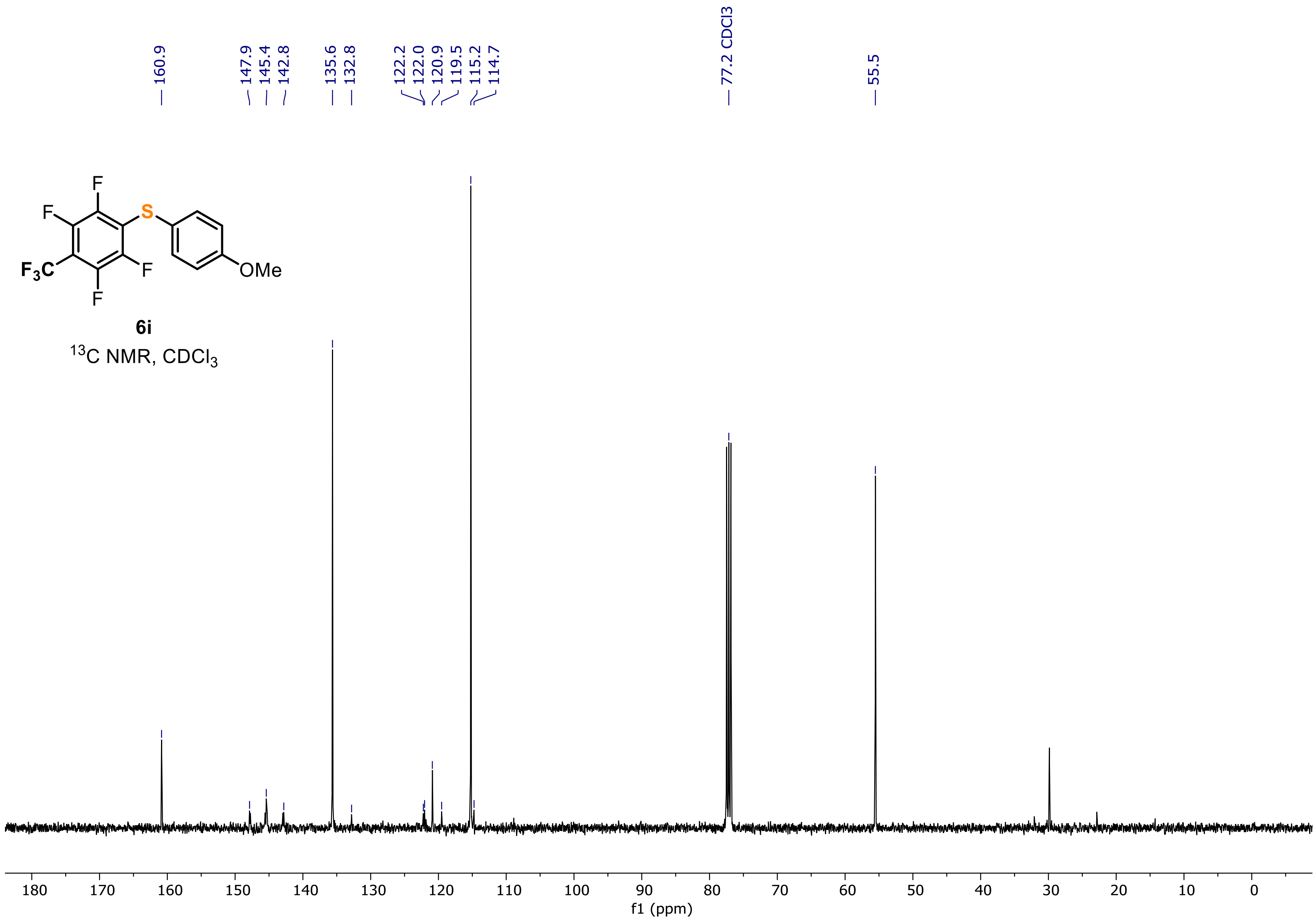
160.9
147.9
145.4
142.8
135.6
132.8
122.2
122.0
120.9
119.5
115.2
114.7
77.2 CDCl3
55.5
F
F
S
F3C
F
OMe
F
6i
13C NMR, CDCl3
180
170
160
150
140
130
120
110
100
90
80
70
60
50
40
30
20
10
0
f1 (ppm)

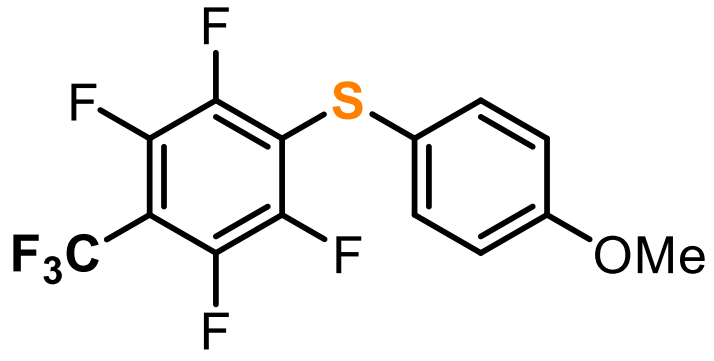

6i

$^{19}F\{^{1}H\}$ NMR, $CDCl_3$

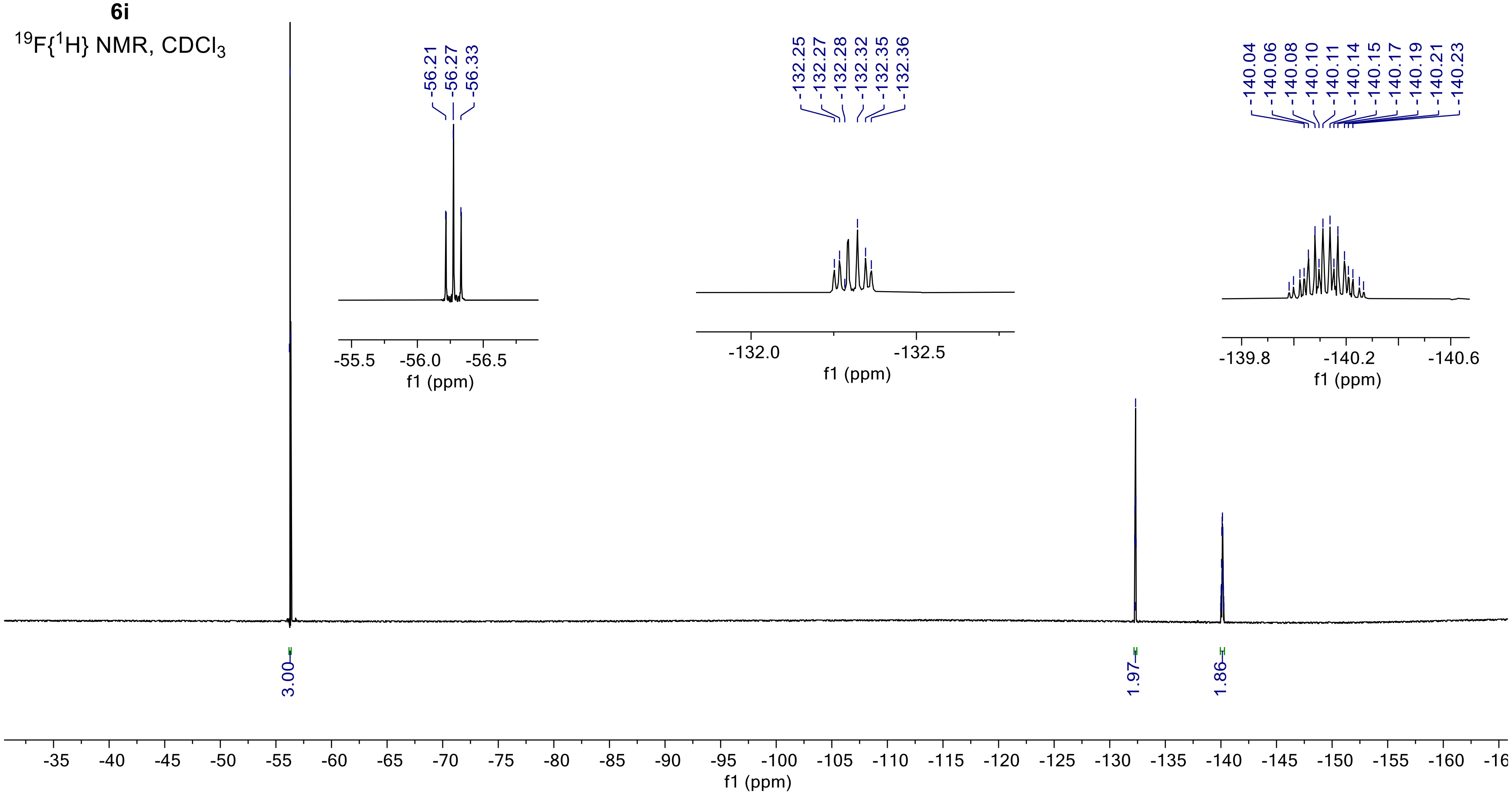

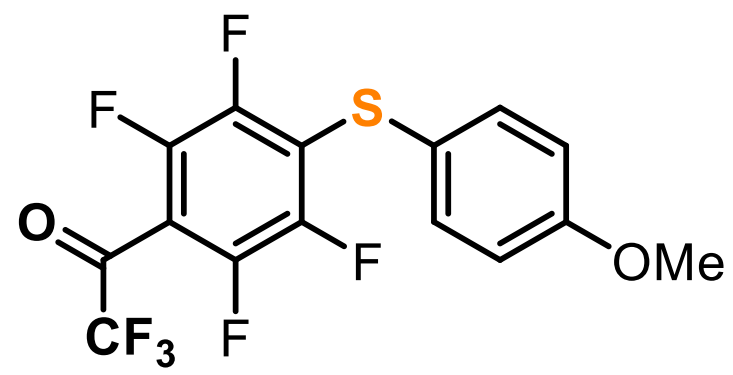

6j

^1H NMR, $CDCl_3$

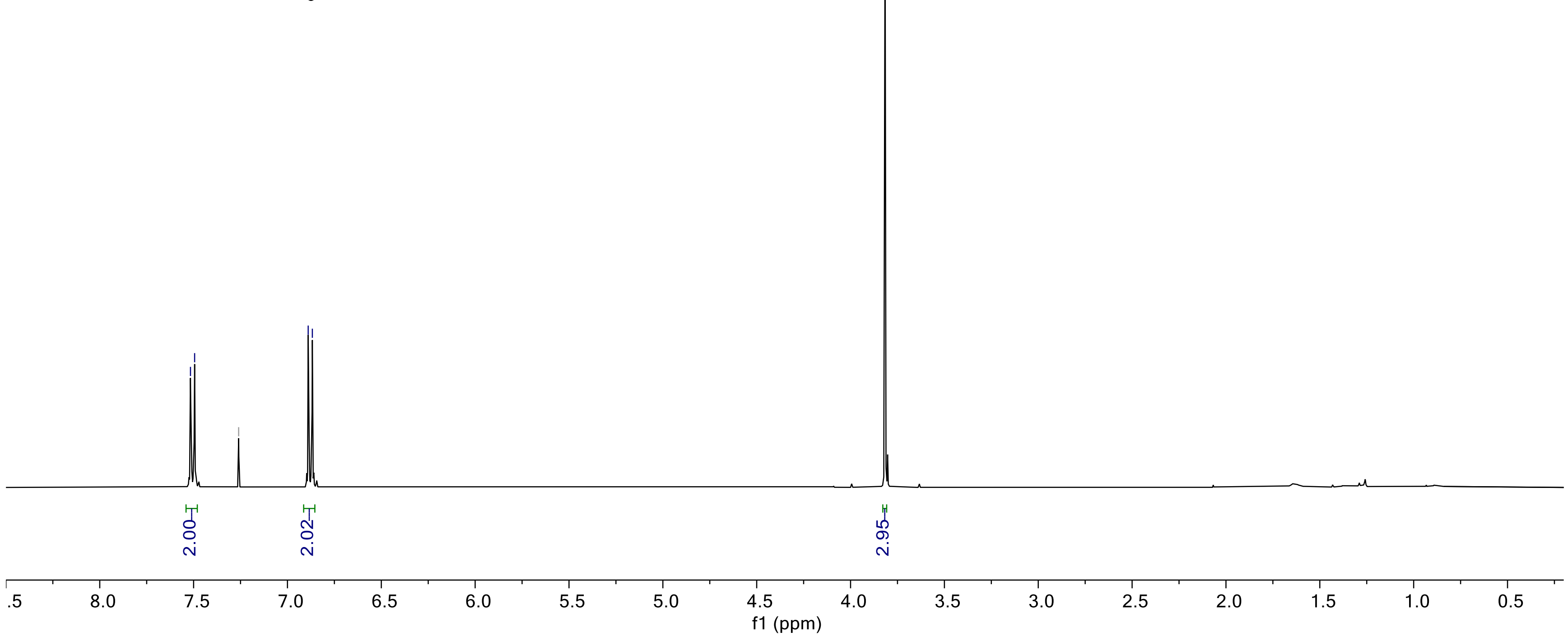

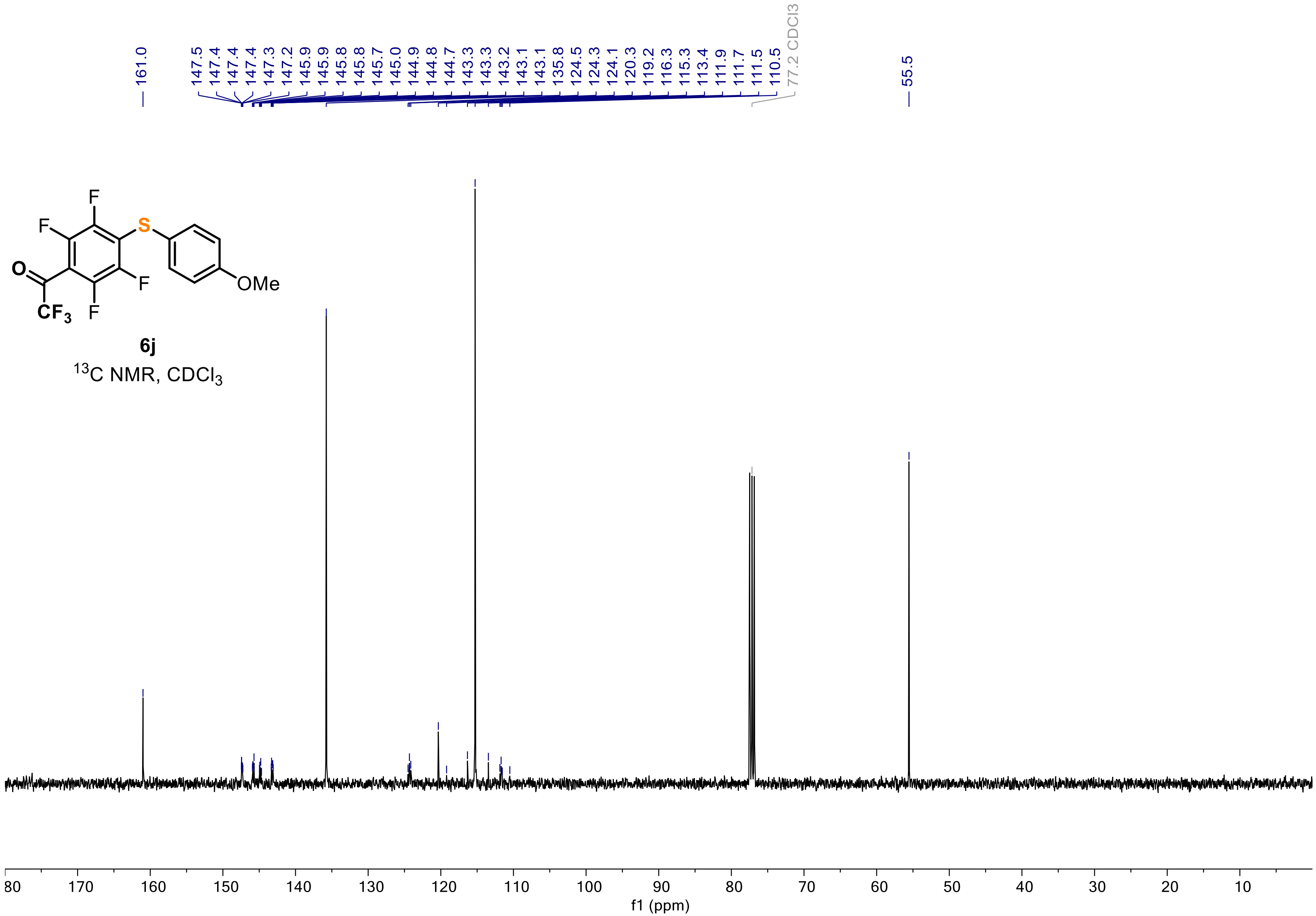

6j

^{13}C NMR, $CDCl_3$

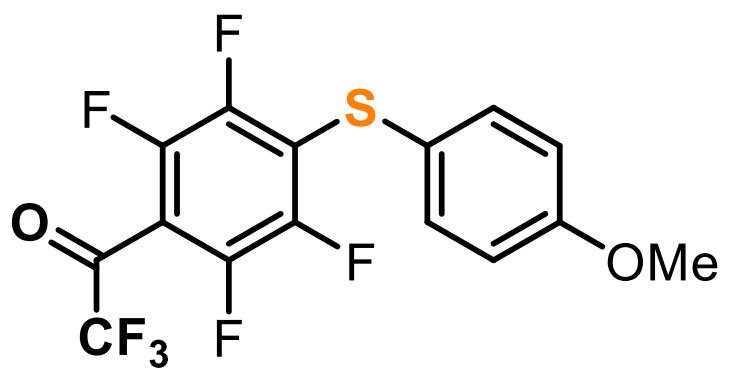

6j

$^{19}F\{^{1}H\}$ NMR, $CDCl_3$

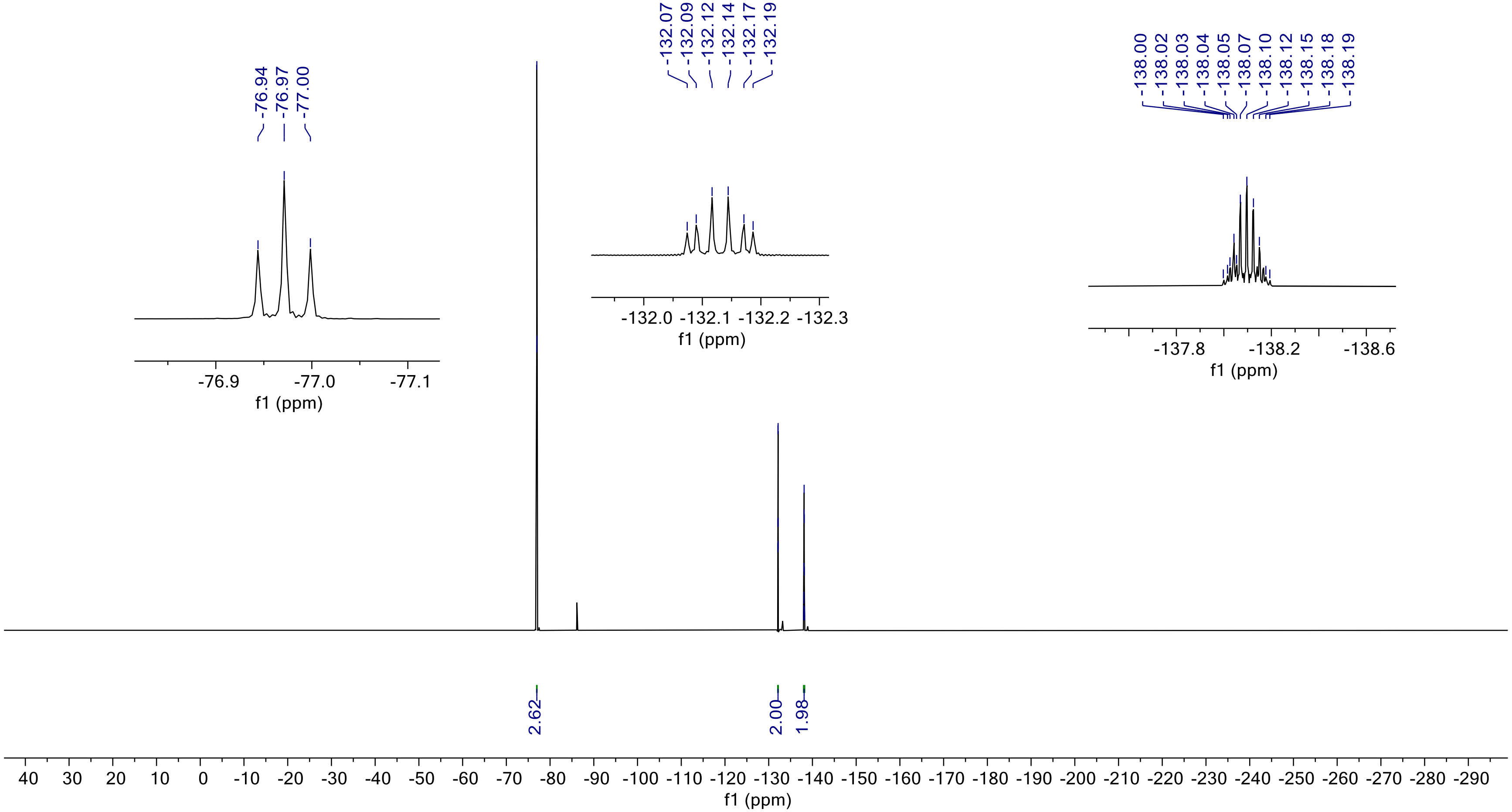

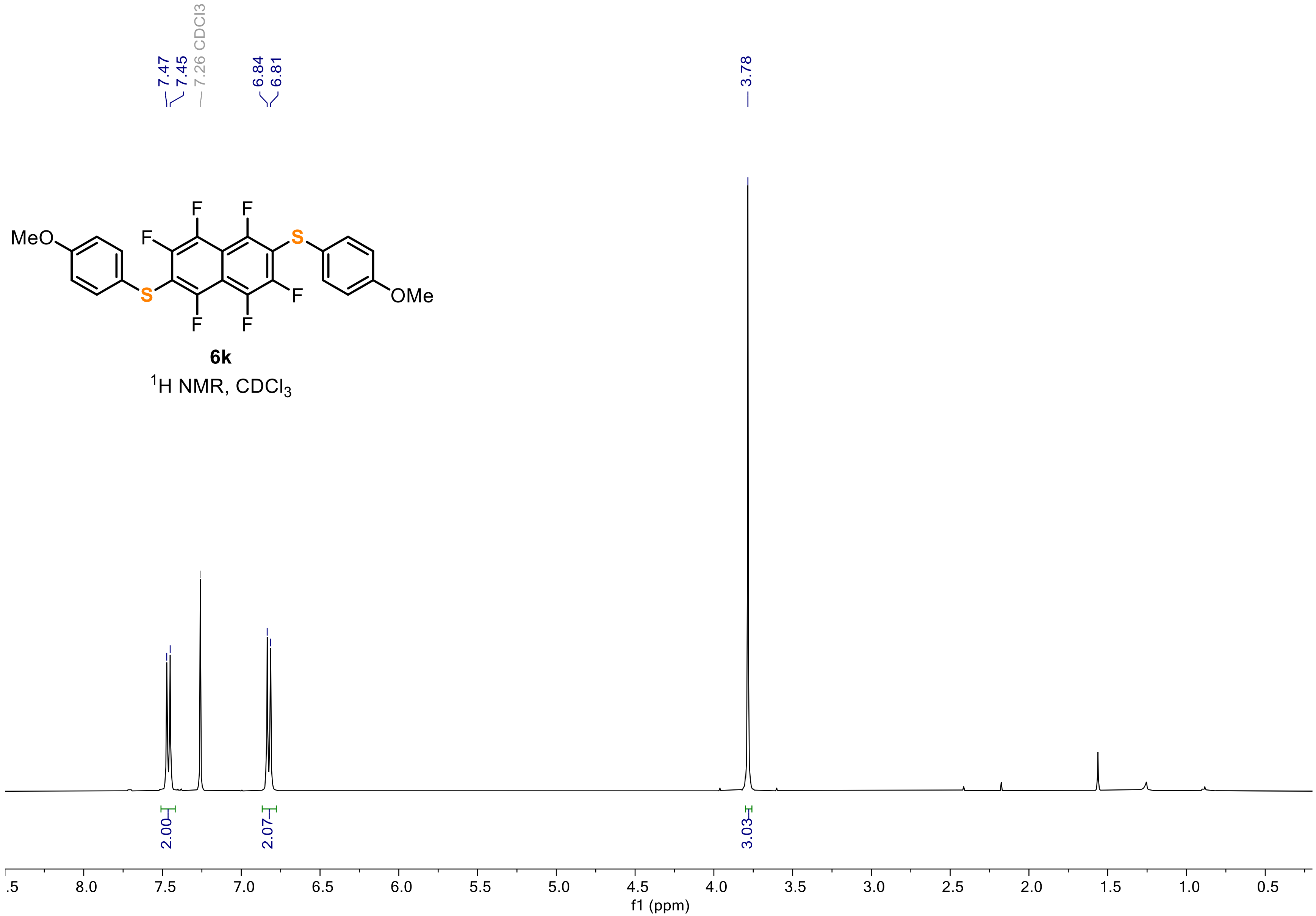
7.47
7.45
7.26 CDCl3
6.84
6.81
3.78
MeO
F
S
OMe
6k
^{1}H NMR, $CDCl_3$
2.00
2.07
3.03
.5
8.0
7.5
7.0
6.5
6.0
5.5
5.0
4.5
4.0
3.5
3.0
2.5
2.0
1.5
1.0
0.5
f1 (ppm)

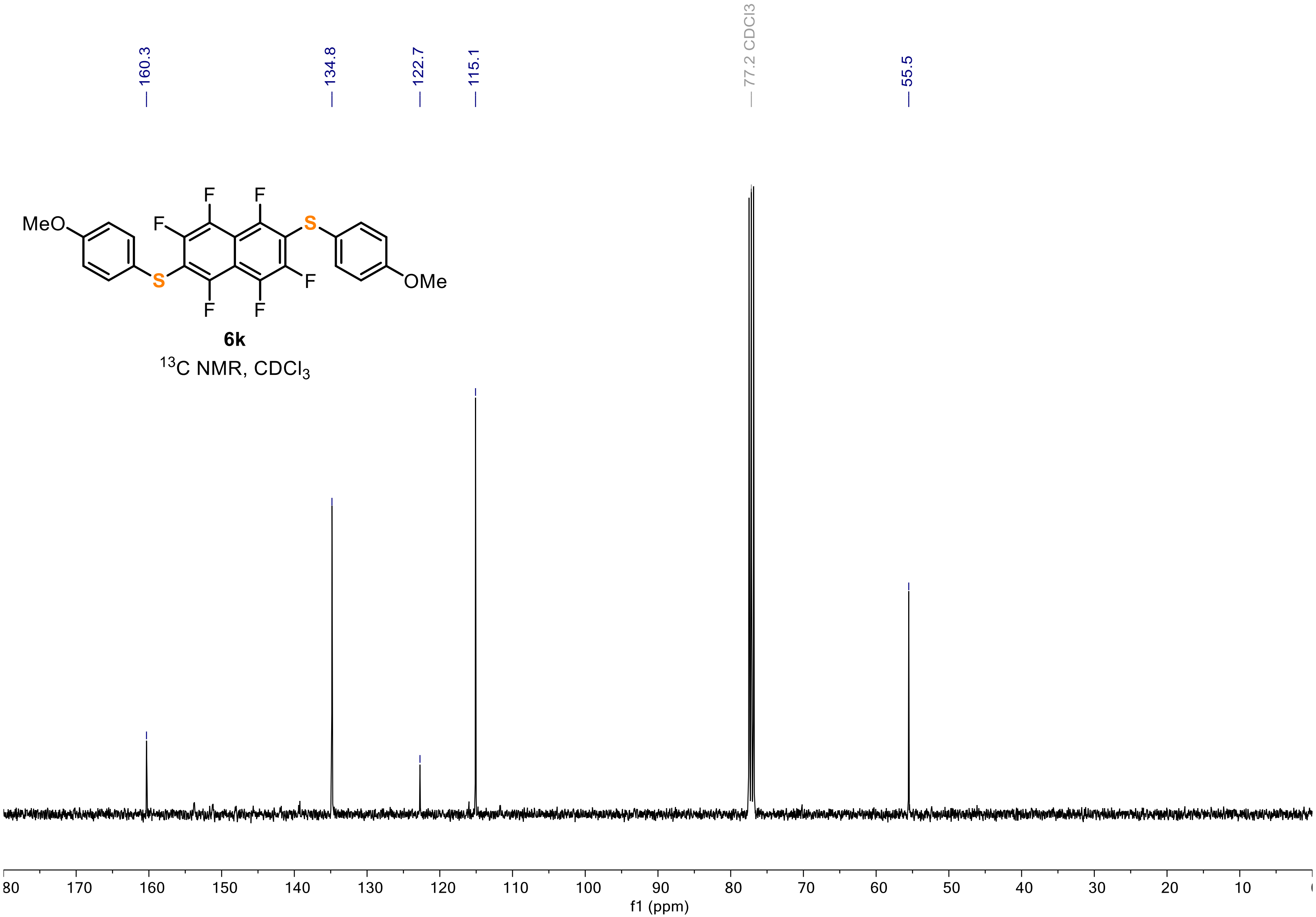

6k

^{13}C NMR, $CDCl_3$

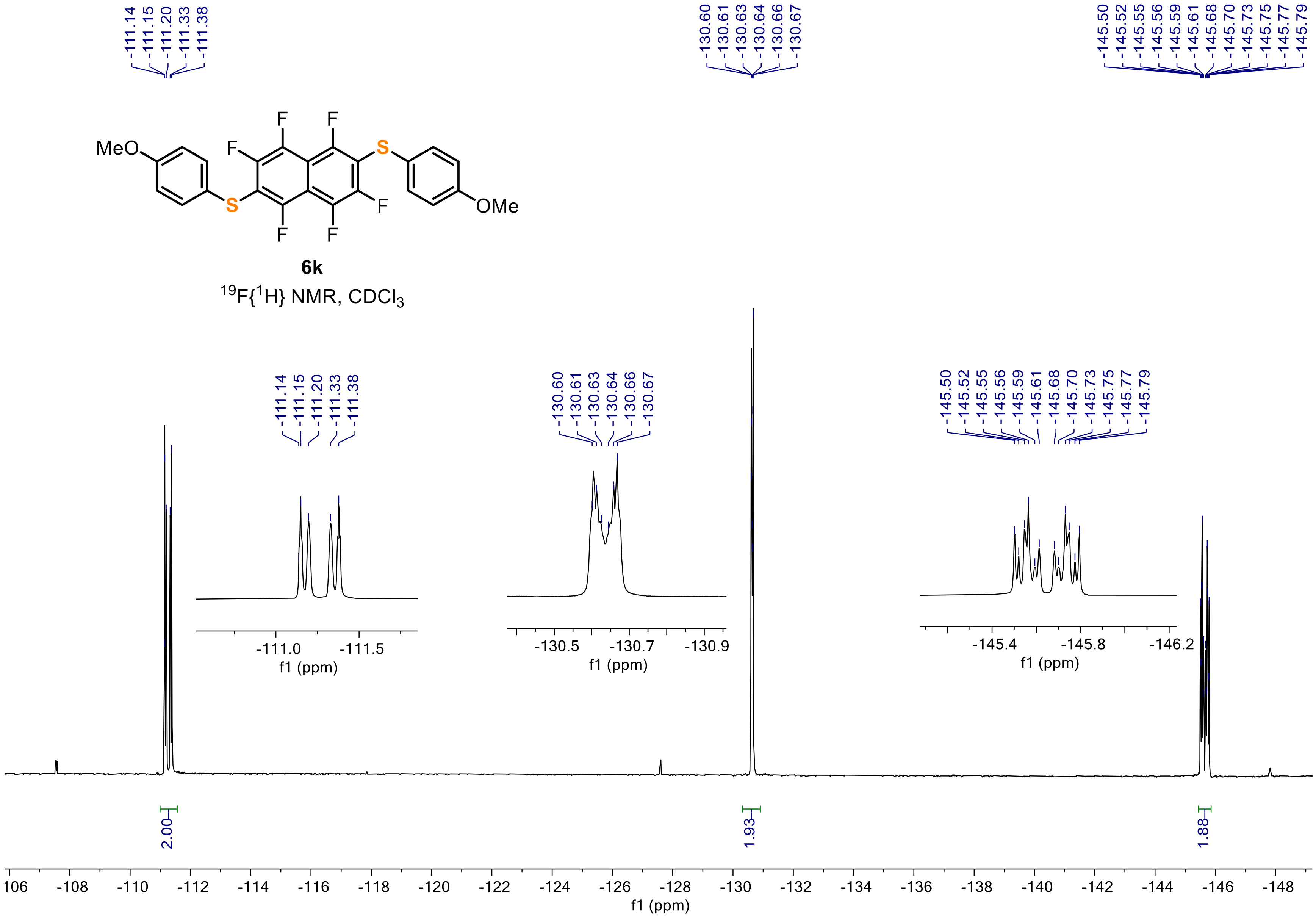

6k

$^{19}F\{^{1}H\}$ NMR, $CDCl_3$

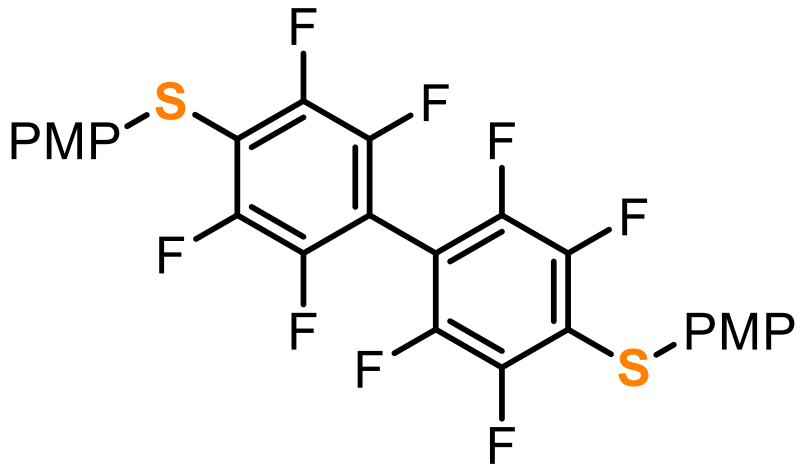

6l

^{1}H NMR, $CDCl_3$

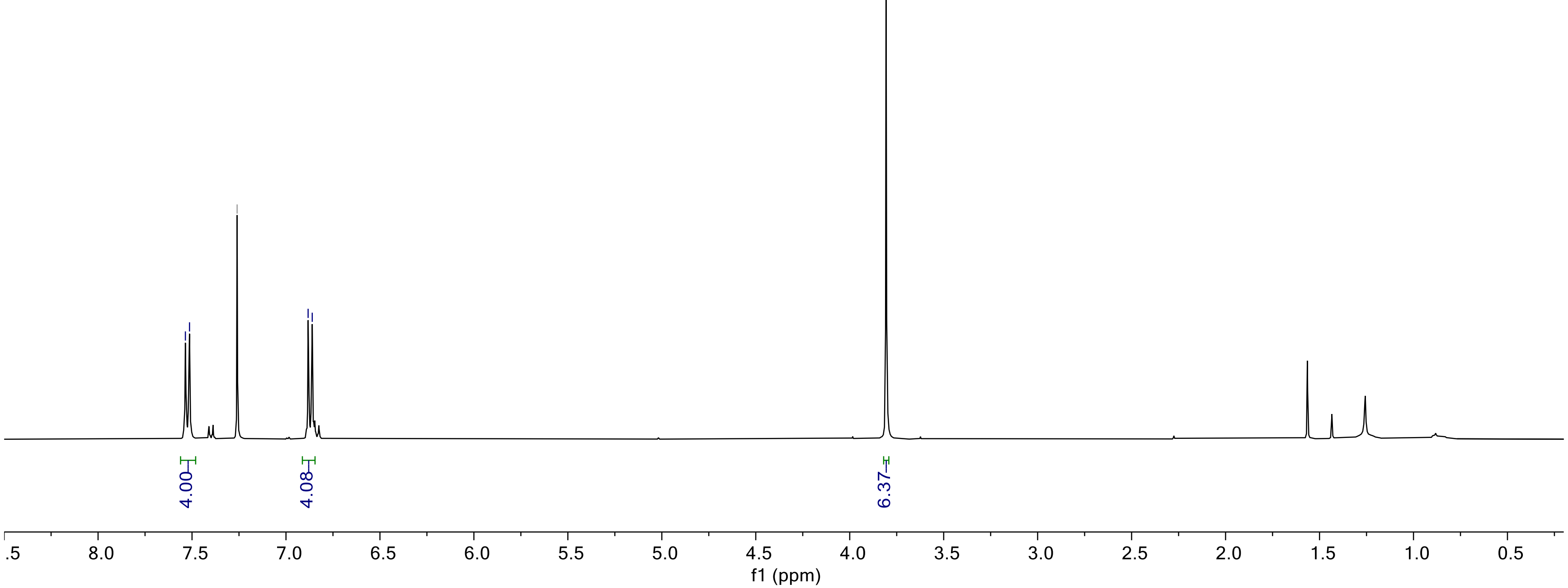

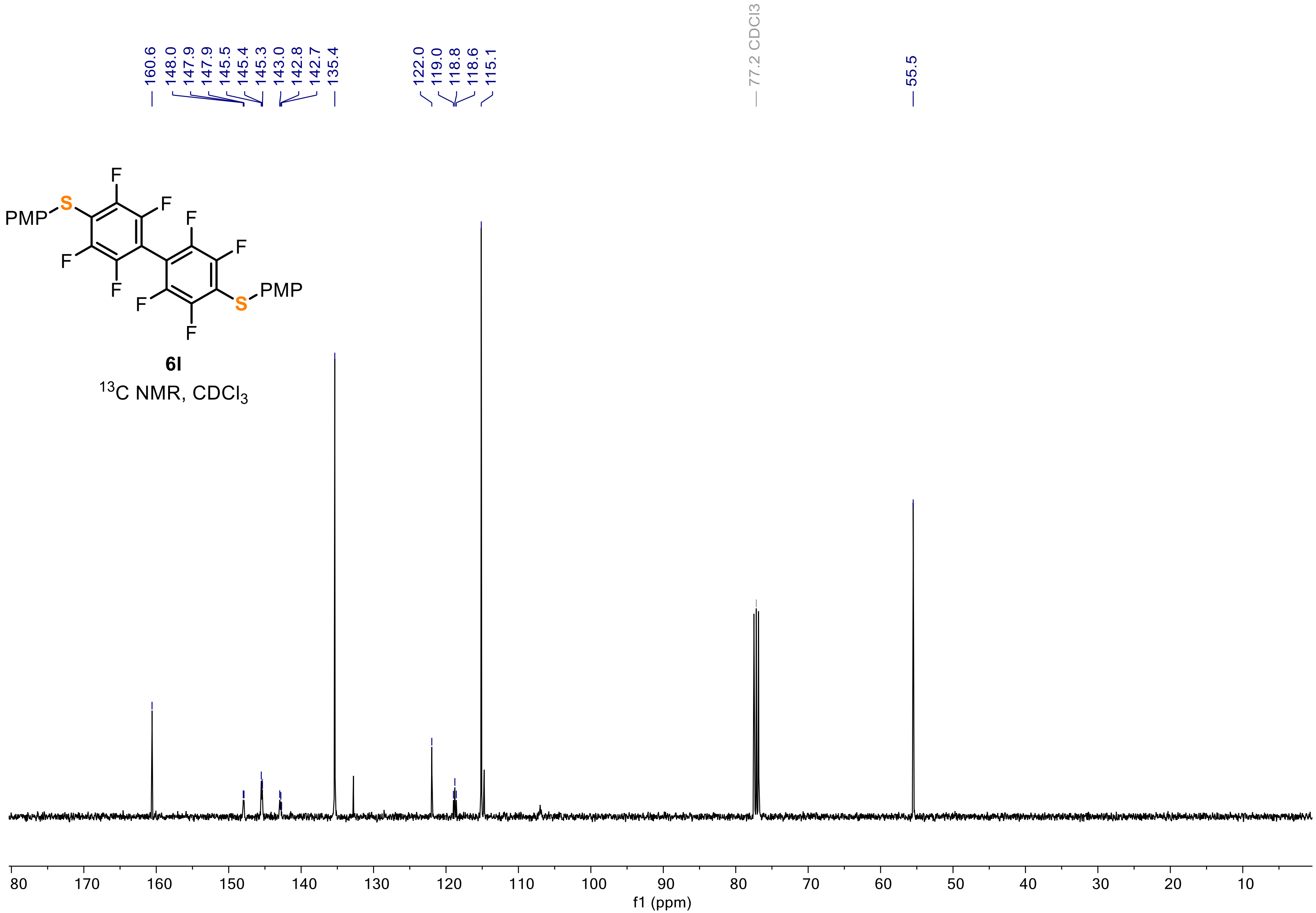
160.6
148.0
147.9
147.9
145.5
145.4
145.3
143.0
142.8
142.7
135.4
122.0
119.0
118.8
118.6
115.1
77.2 CDCl3
55.5
PMP
S
F
6l
^{13}C NMR, $CDCl_3$
180
170
160
150
140
130
120
110
100
90
80
70
60
50
40
30
20
10
f1 (ppm)

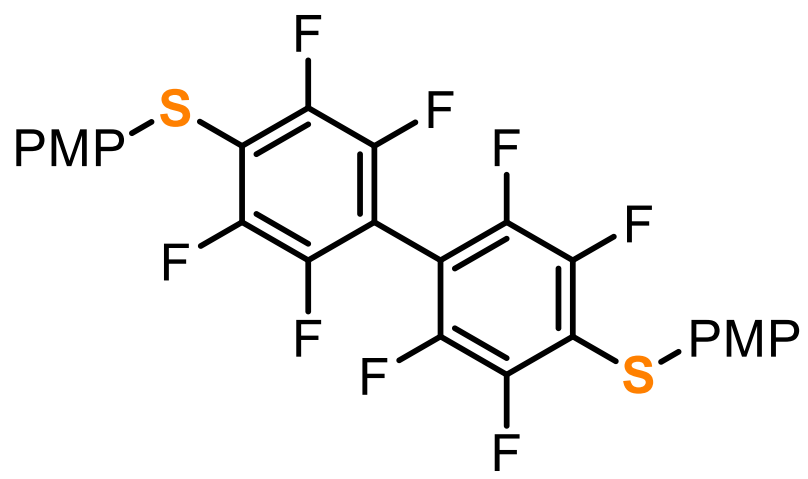

6l

$^{19}F\{^{1}H\}$ NMR, $CDCl_3$

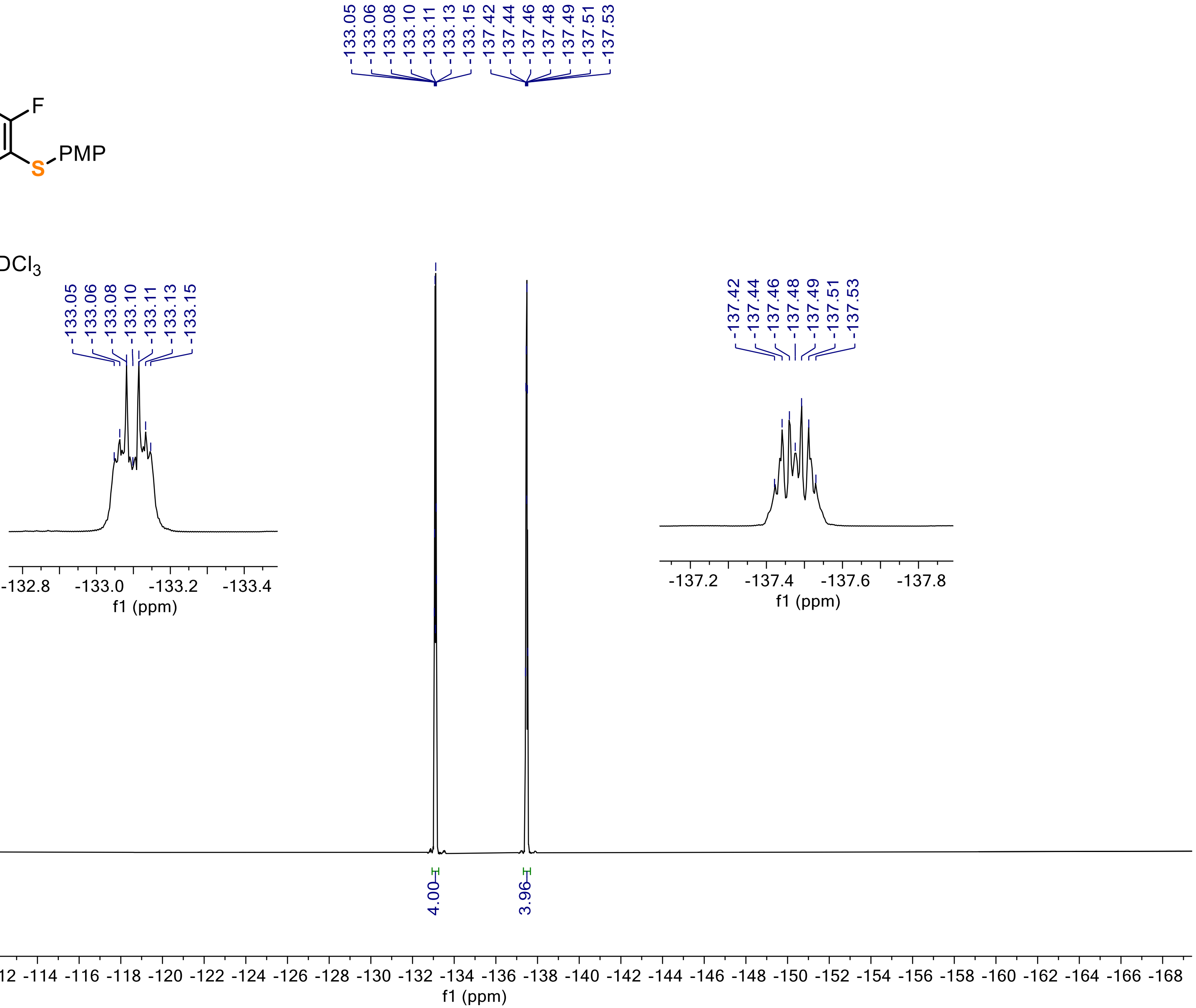

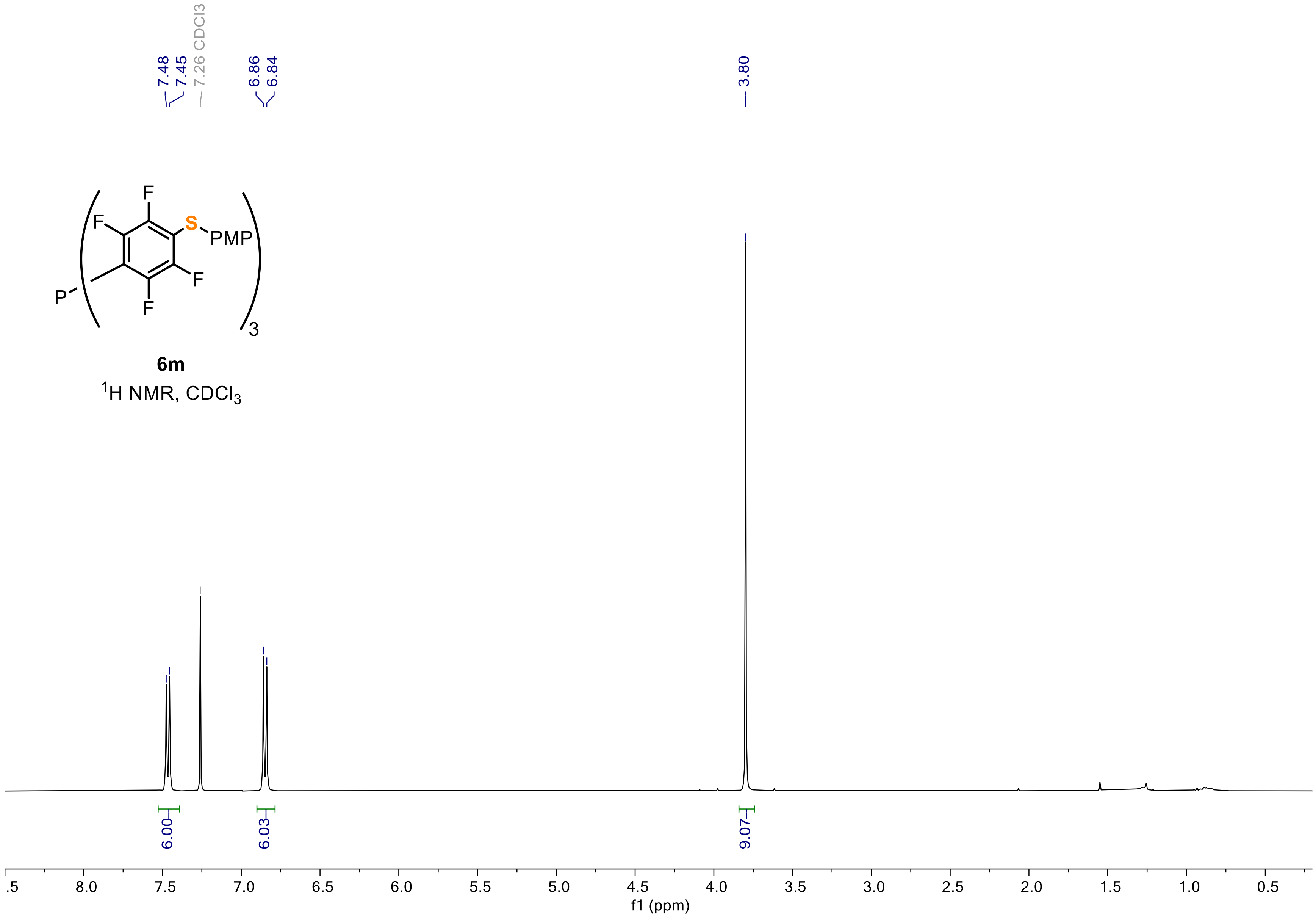

7.48
7.45
7.26 CDCl3
6.86
6.84
3.80
F
F
S
PMP
F
P
F
3
6m
1H NMR, CDCl3
6.00
6.03
9.07
.5
8.0
7.5
7.0
6.5
6.0
5.5
5.0
4.5
4.0
3.5
3.0
2.5
2.0
1.5
1.0
0.5
f1 (ppm)

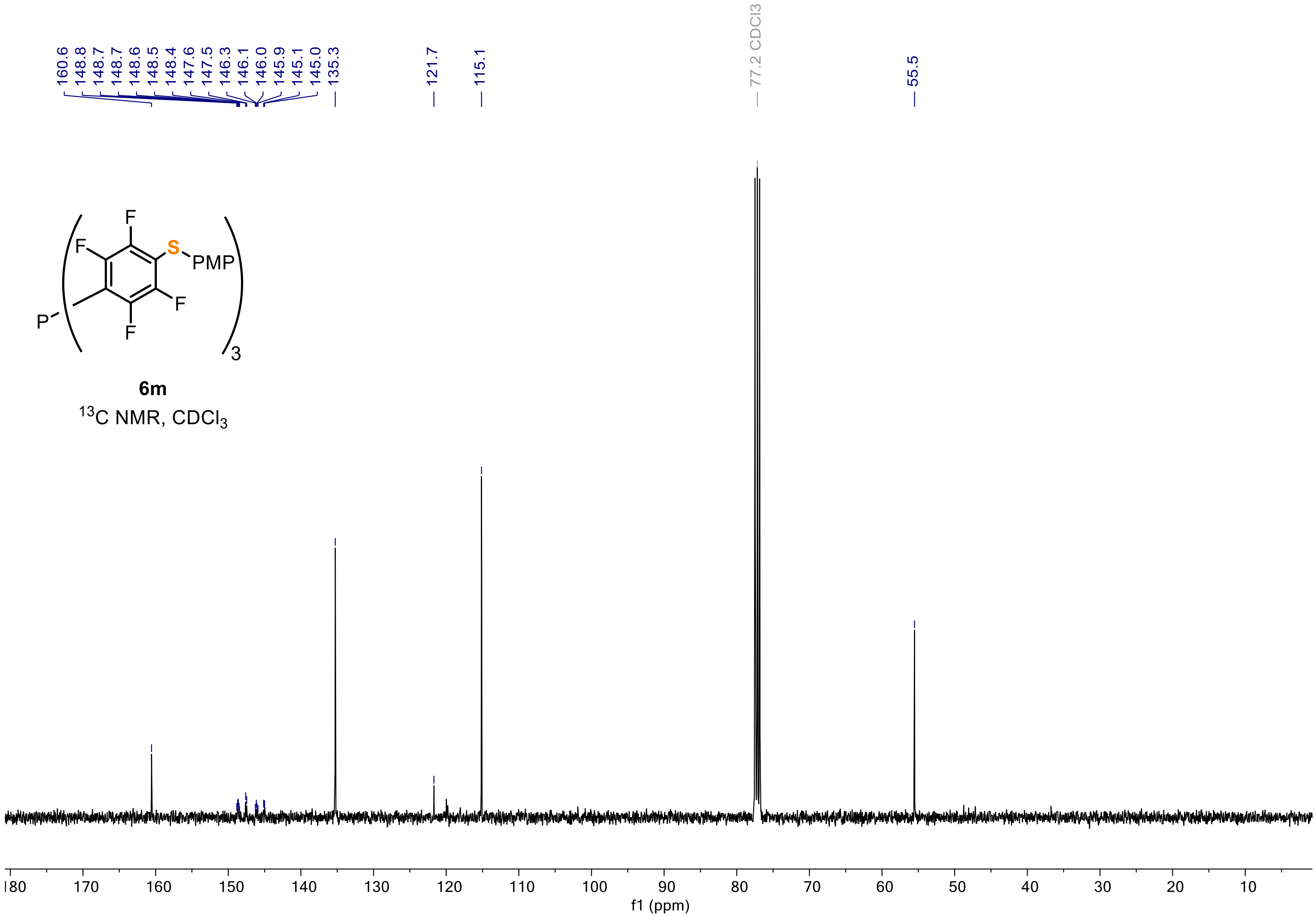

6m

^{13}C NMR, $CDCl_3$

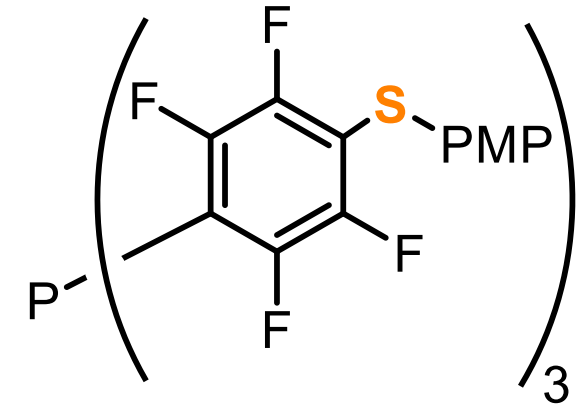

6m

$^{19}F\{^1H\}$ NMR, $CDCl_3$

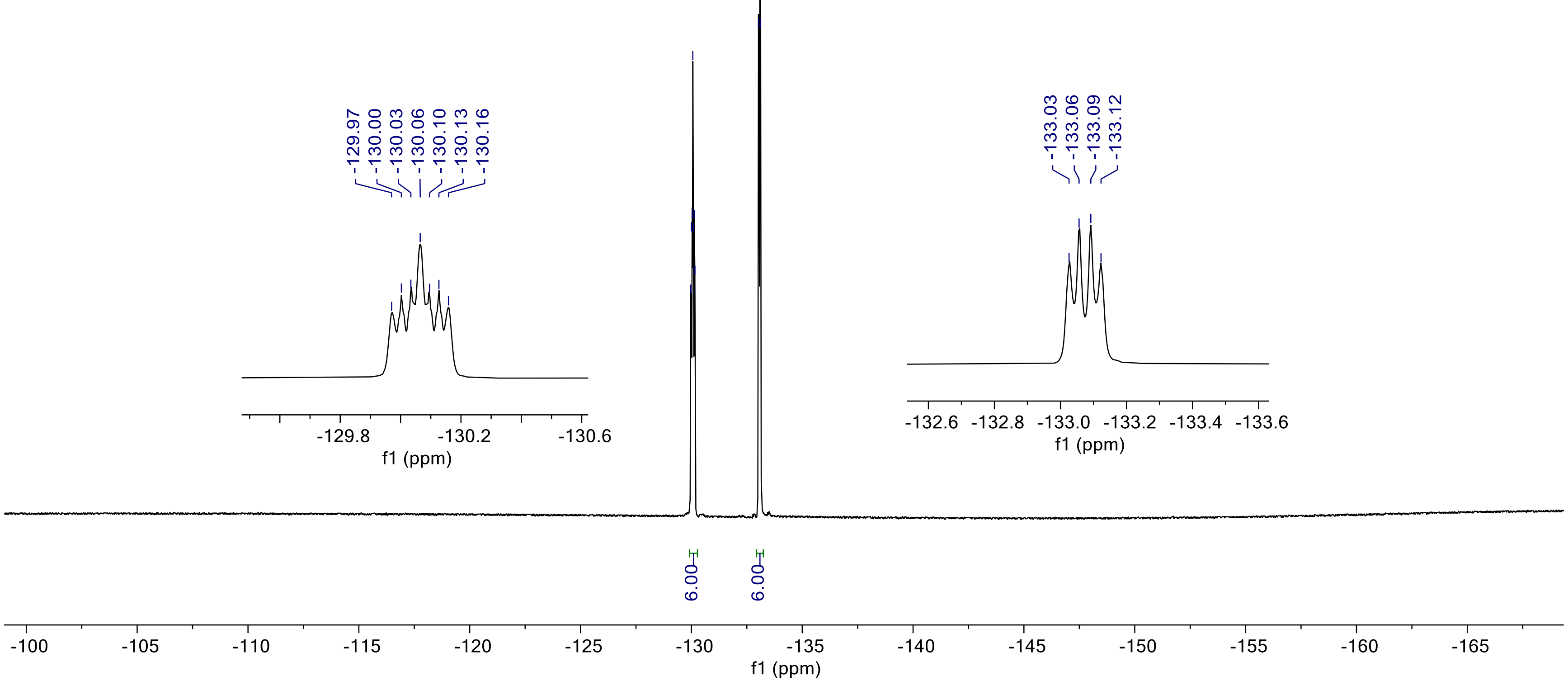

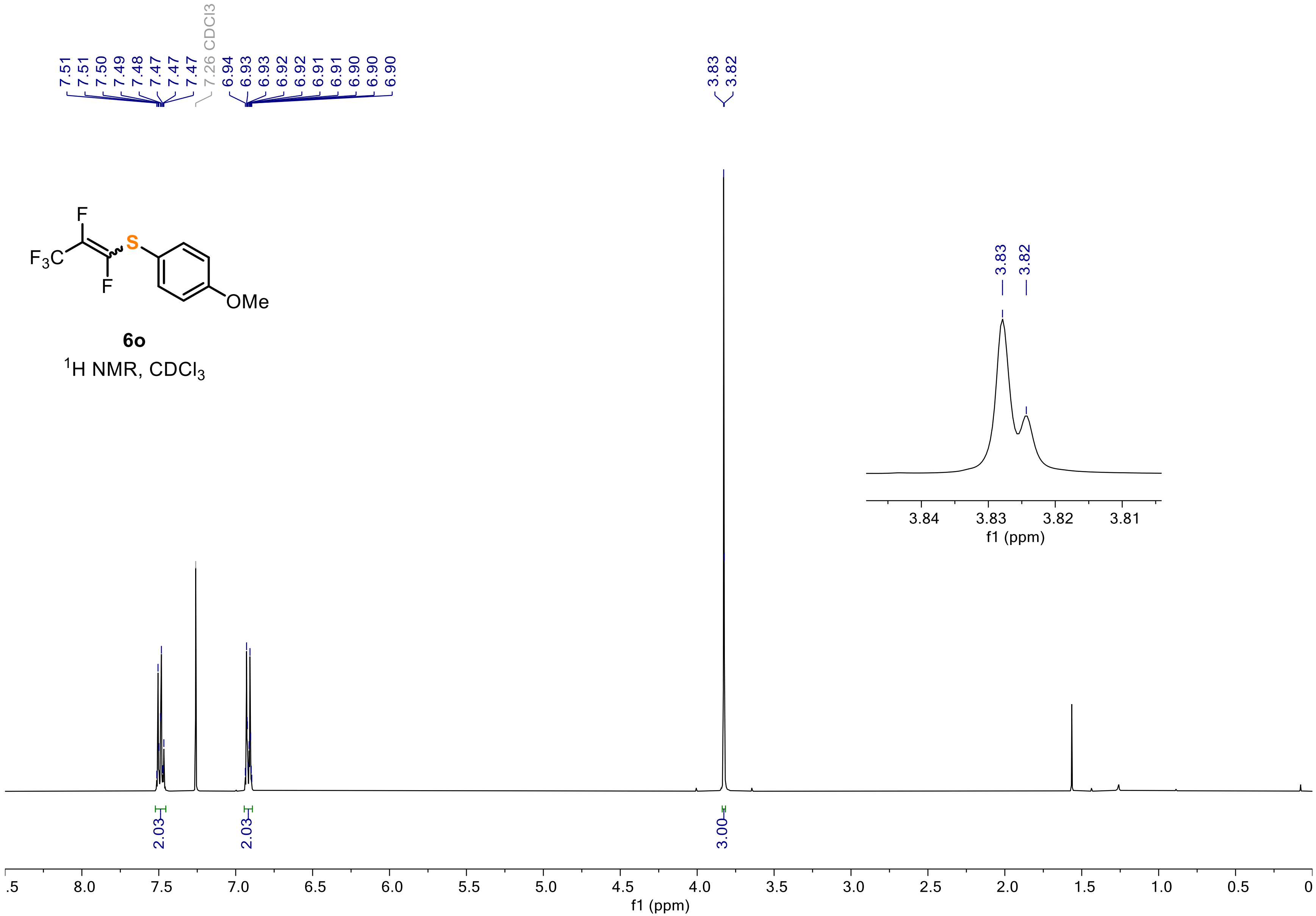

7.51
7.51
7.50
7.49
7.48
7.47
7.47
7.47
7.26 CDCl3
6.94
6.93
6.93
6.92
6.92
6.91
6.91
6.90
6.90
6.90
3.83
3.82
F
F3C
S
F
OMe
6o
1H NMR, CDCl3
3.83
3.82
3.84
3.83
3.82
3.81
f1 (ppm)
2.03
2.03
3.00
.5
8.0
7.5
7.0
6.5
6.0
5.5
5.0
4.5
4.0
3.5
3.0
2.5
2.0
1.5
1.0
0.5
0
f1 (ppm)

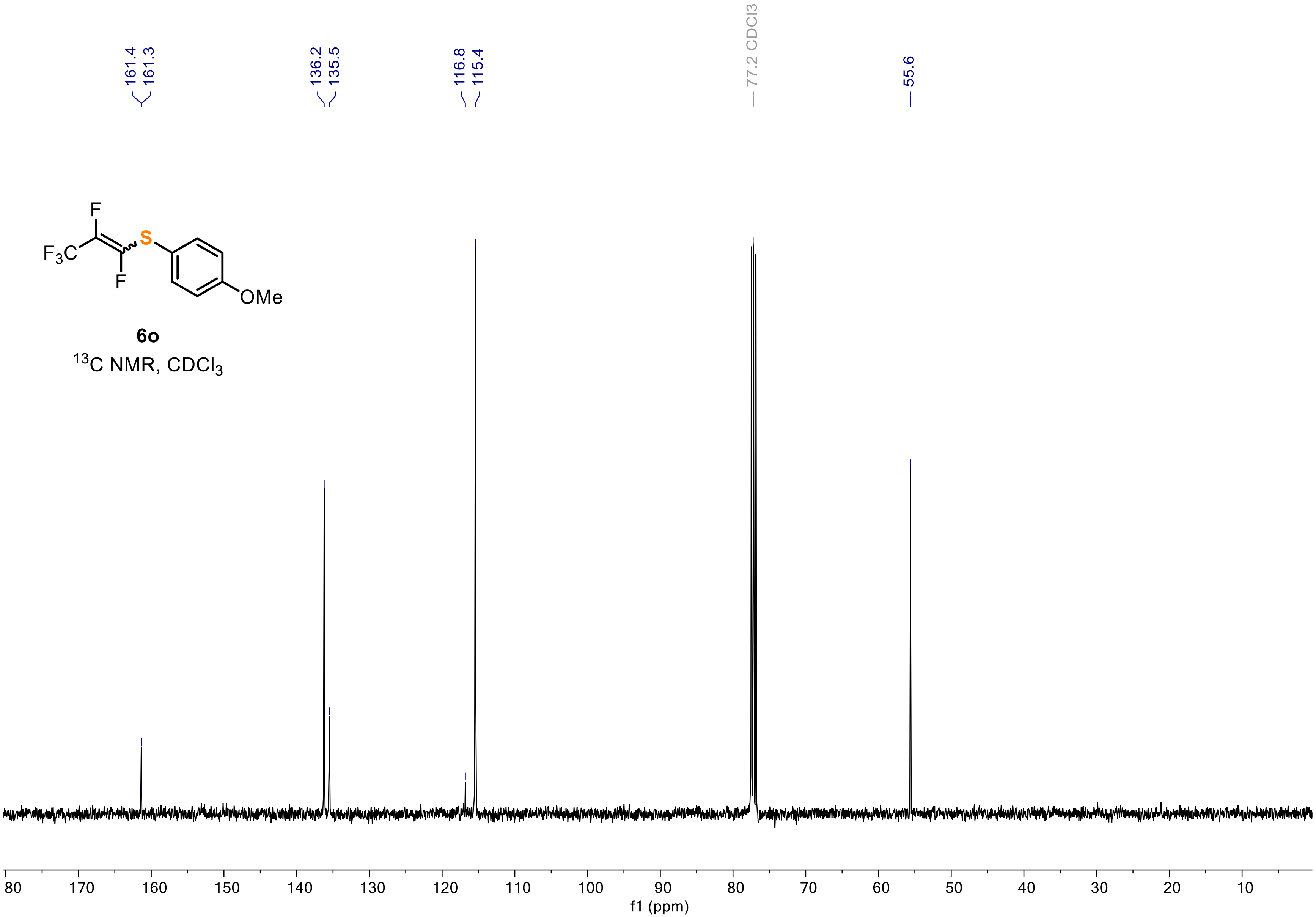

6o

^{13}C NMR, $CDCl_3$

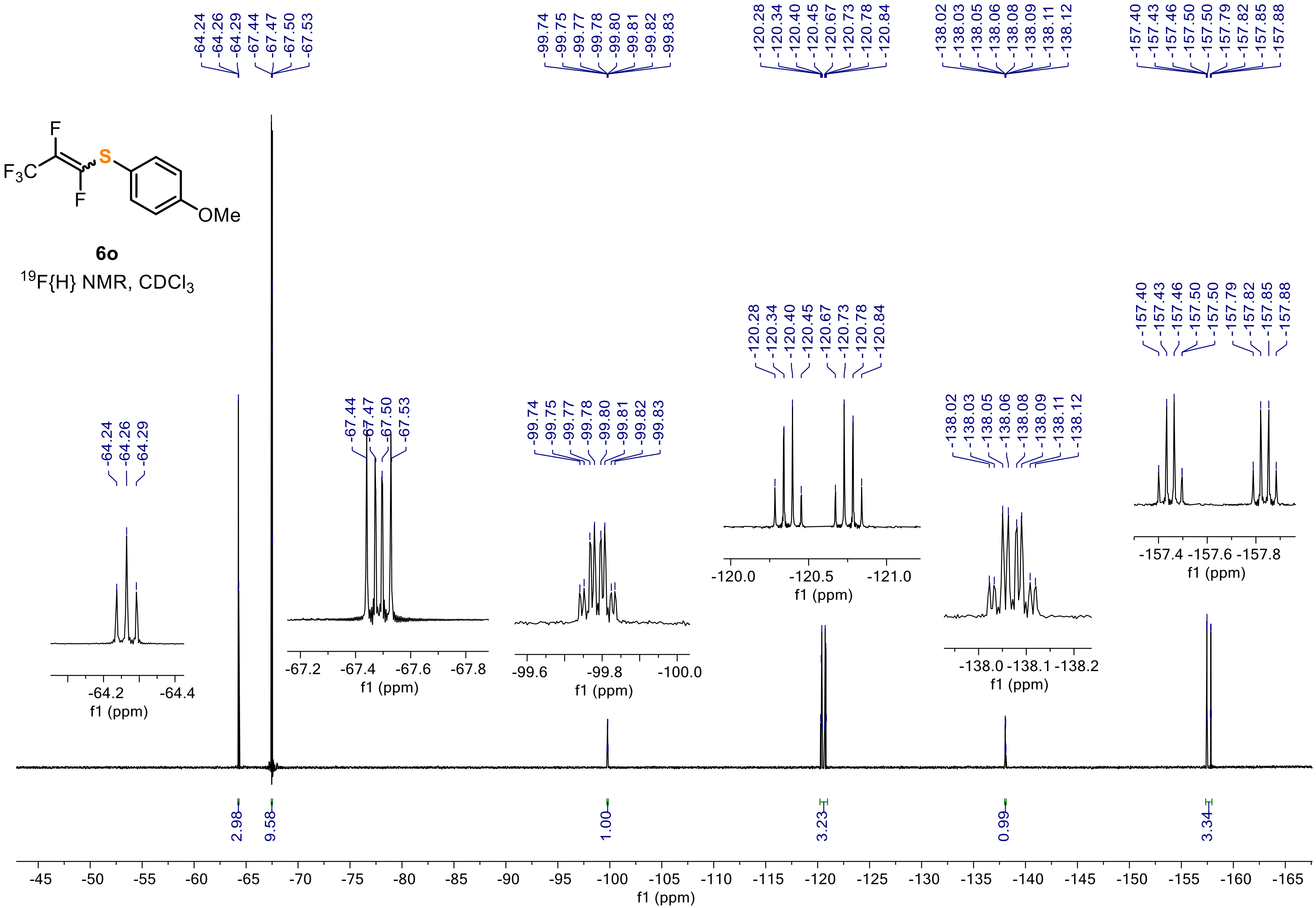
6o
19F{H} NMR, CDCl3
-64.24 -64.26 -64.29 -67.44 -67.47 -67.50 -67.53
-99.74 -99.75 -99.77 -99.78 -99.80 -99.81 -99.82 -99.83
-120.28 -120.34 -120.40 -120.45 -120.67 -120.73 -120.78 -120.84
-138.02 -138.03 -138.05 -138.06 -138.08 -138.09 -138.11 -138.12
-157.40 -157.43 -157.46 -157.50 -157.50 -157.79 -157.82 -157.85 -157.88
2.98 9.58 1.00 3.23 0.99 3.34
f1 (ppm)

7.3 NMR characterization of defluoroamination products

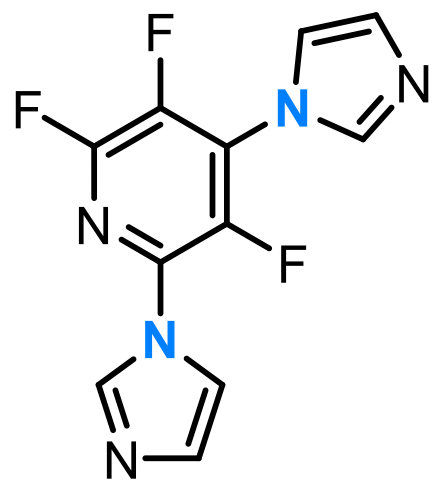

7a'

^{1}H NMR, $CDCl_3$

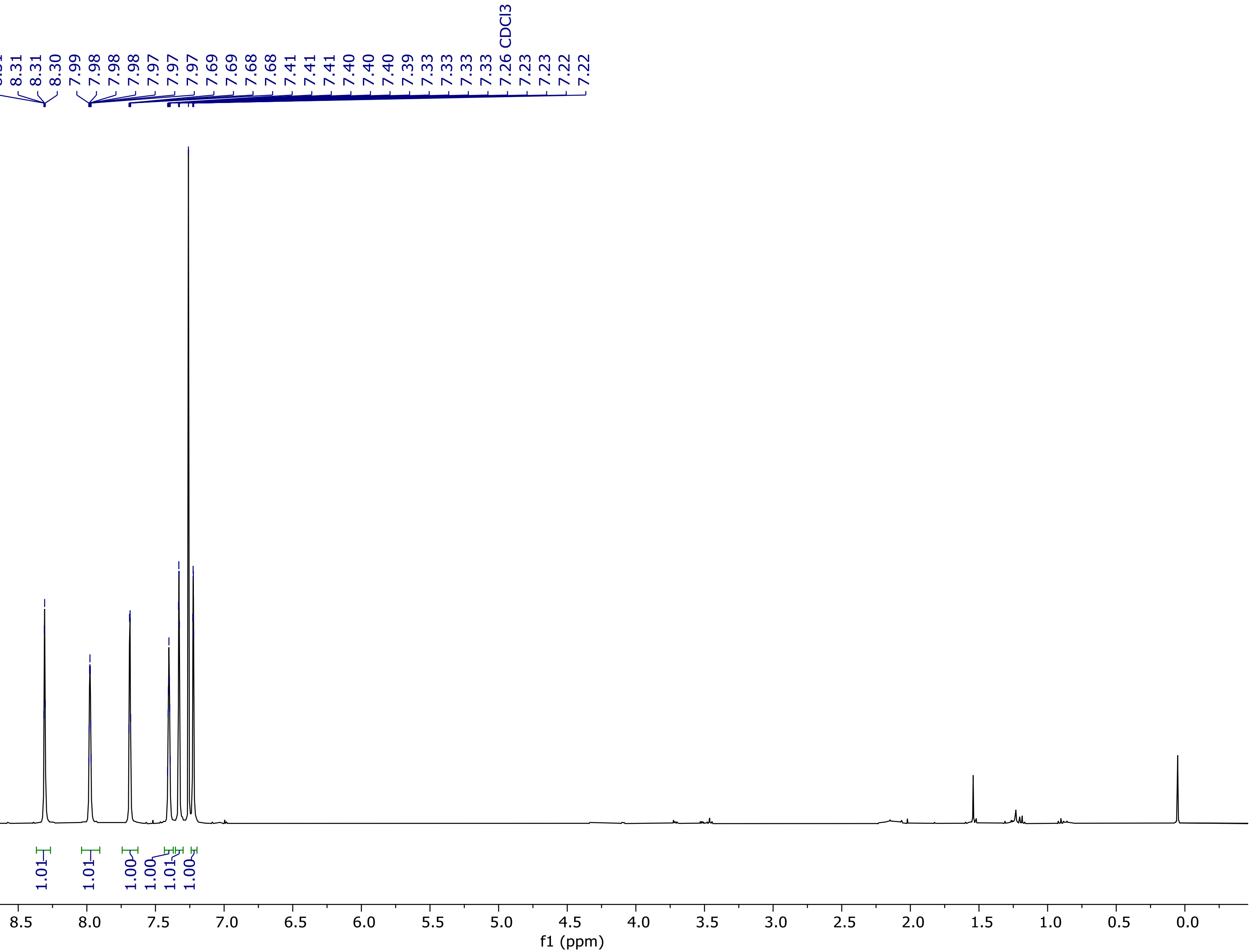

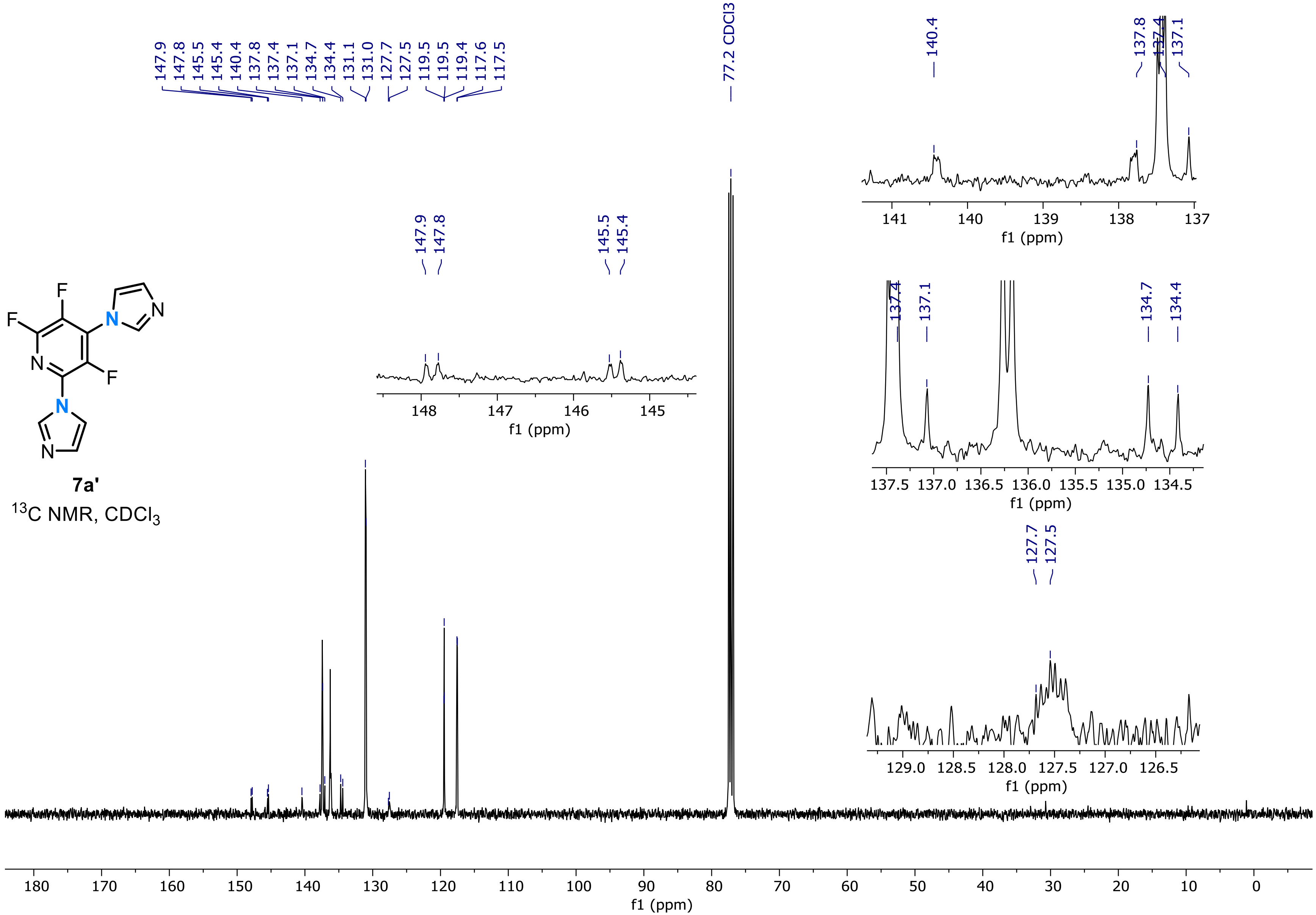

7a'

^{13}C NMR, $CDCl_3$

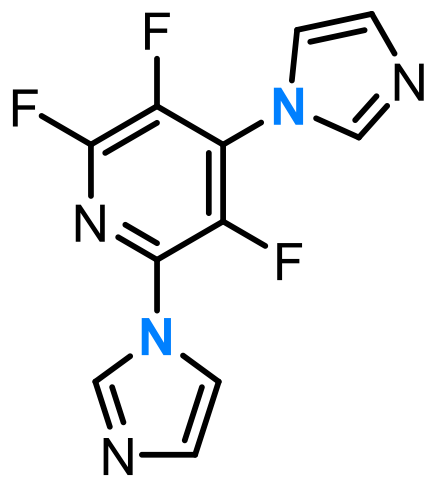

7a'

$^{19}F\{^{1}H\}$ NMR, $CDCl_3$

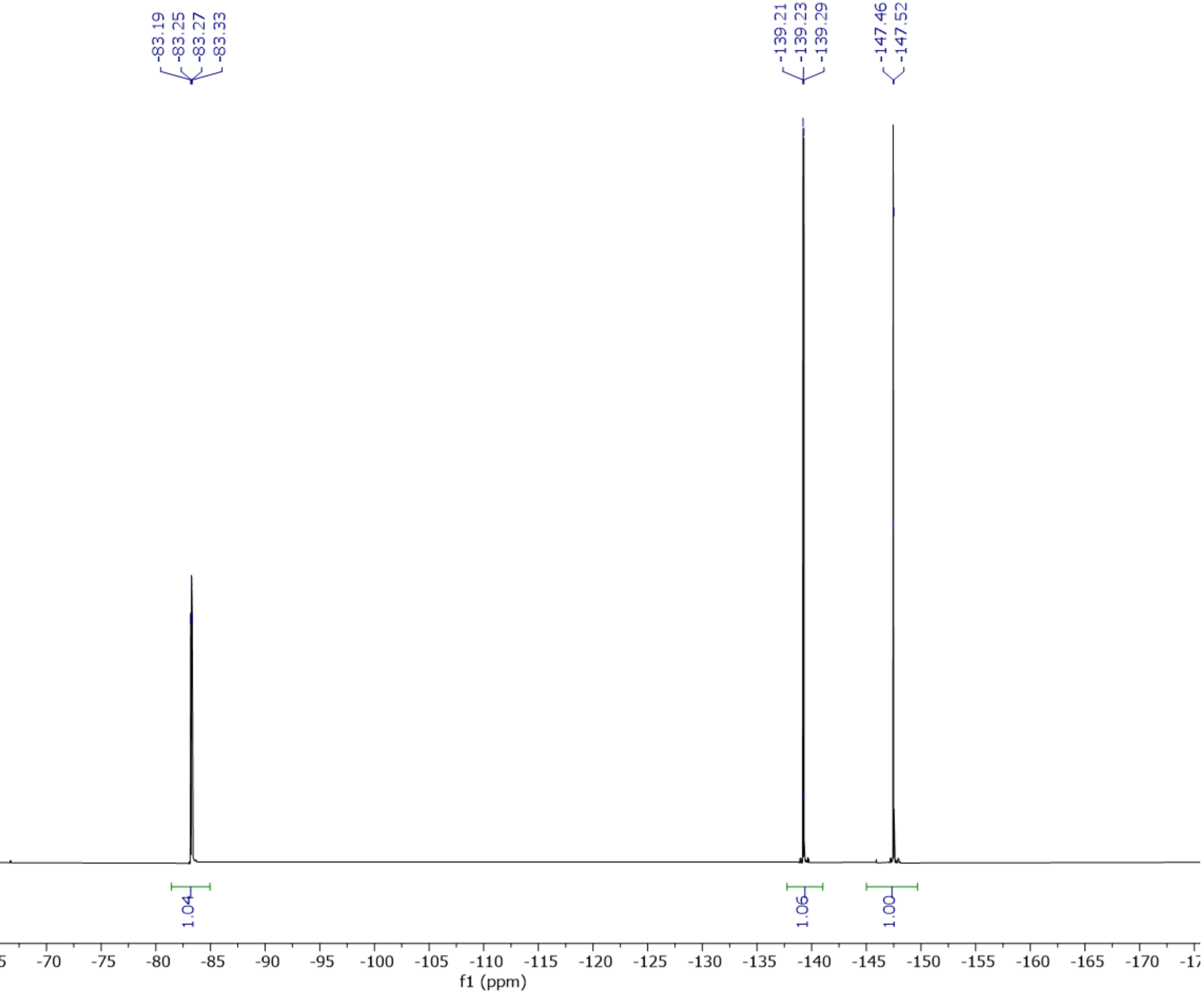

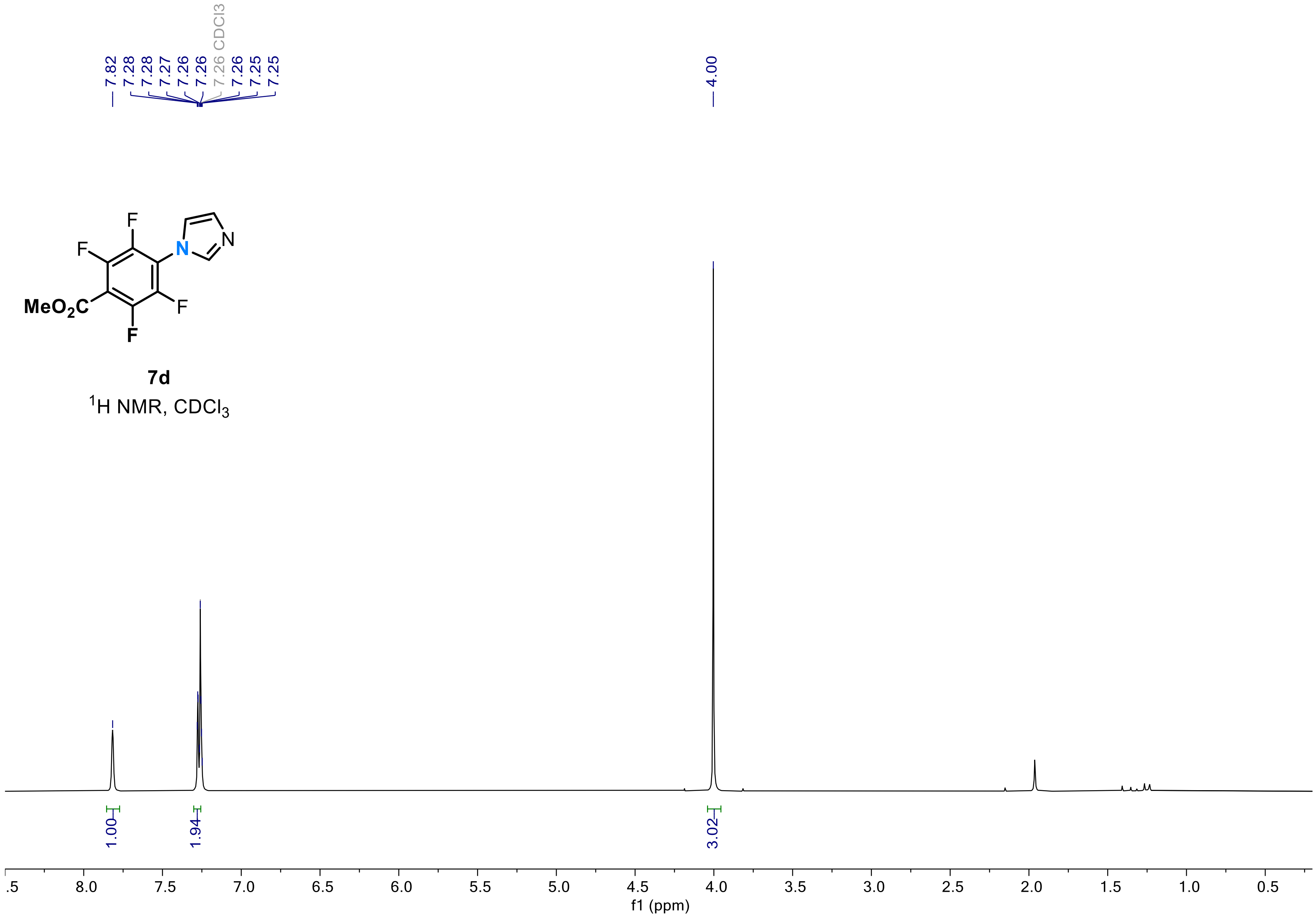
7.82
7.28
7.28
7.27
7.26
7.26
7.26 CDCl3
7.26
7.25
7.25
4.00
7d
1H NMR, CDCl3
1.00
1.94
3.02
.5 8.0 7.5 7.0 6.5 6.0 5.5 5.0 4.5 4.0 3.5 3.0 2.5 2.0 1.5 1.0 0.5
f1 (ppm)

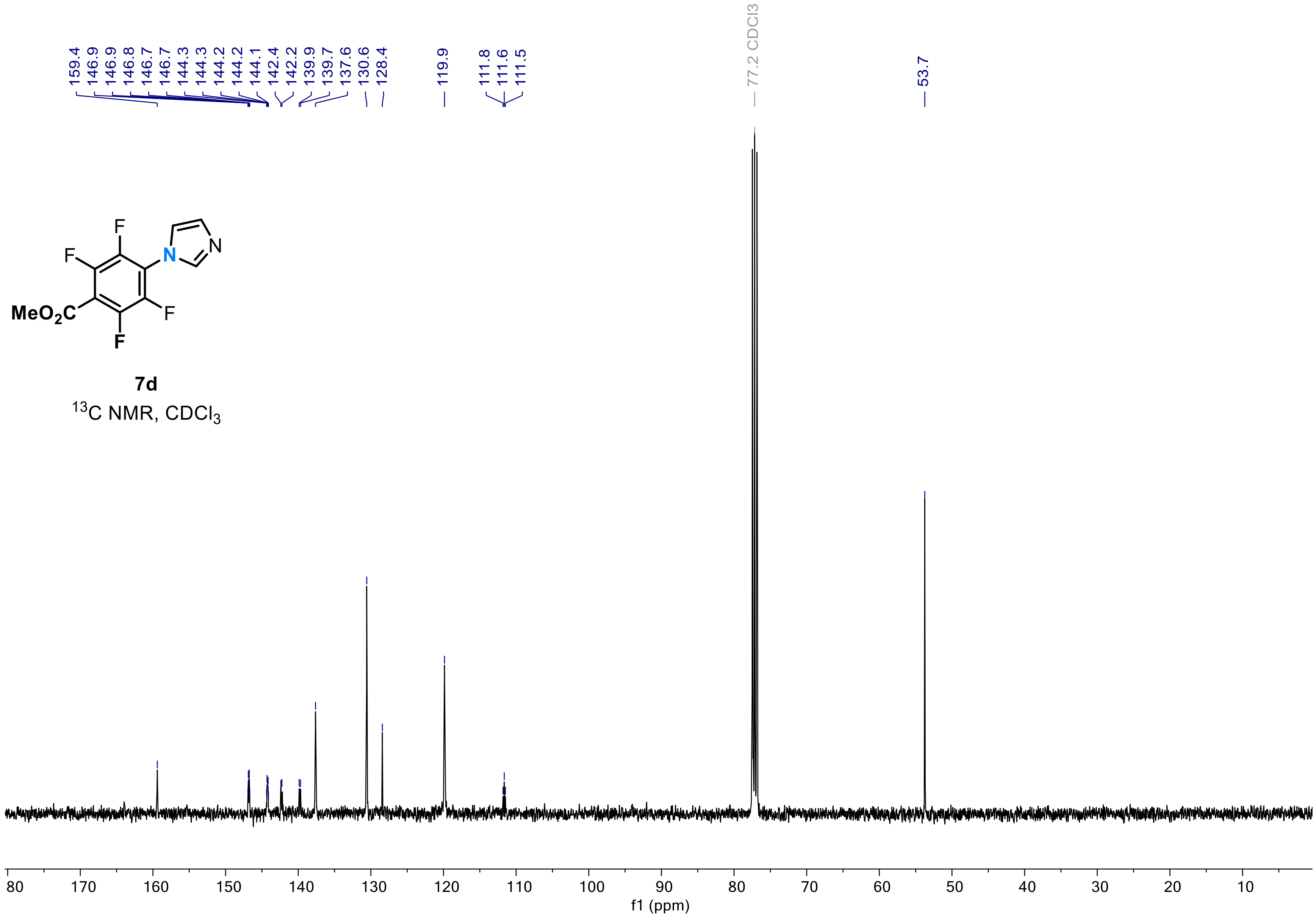
159.4
146.9
146.9
146.8
146.7
146.7
144.3
144.3
144.2
144.2
144.1
142.4
142.2
139.9
139.7
137.6
130.6
128.4
119.9
111.8
111.6
111.5
77.2 CDCl3
53.7
F
F
N
N
MeO2C
F
F
7d
13C NMR, CDCl3
80 170 160 150 140 130 120 110 100 90 80 70 60 50 40 30 20 10
f1 (ppm)

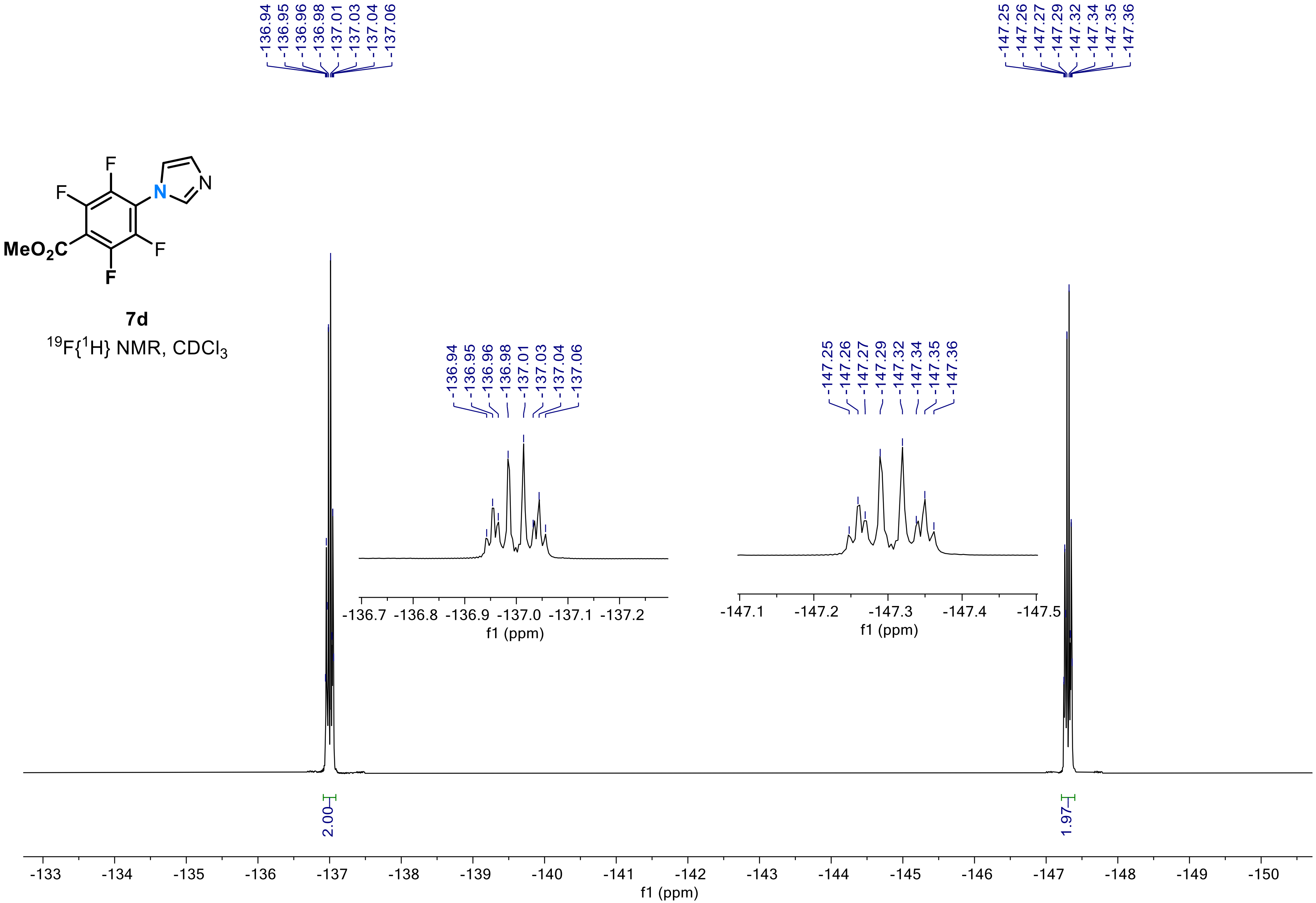

7d

$^{19}F\{^{1}H\}$ NMR, $CDCl_3$

7.4 NMR characterization of defluorophosphonylation products

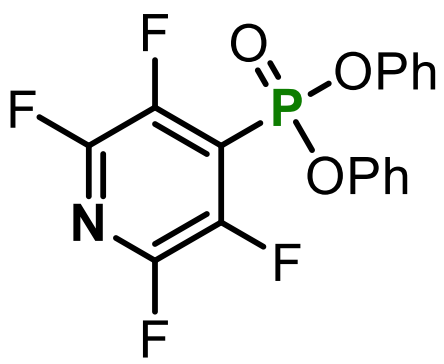

8a

^{1}H NMR, $CDCl_3$

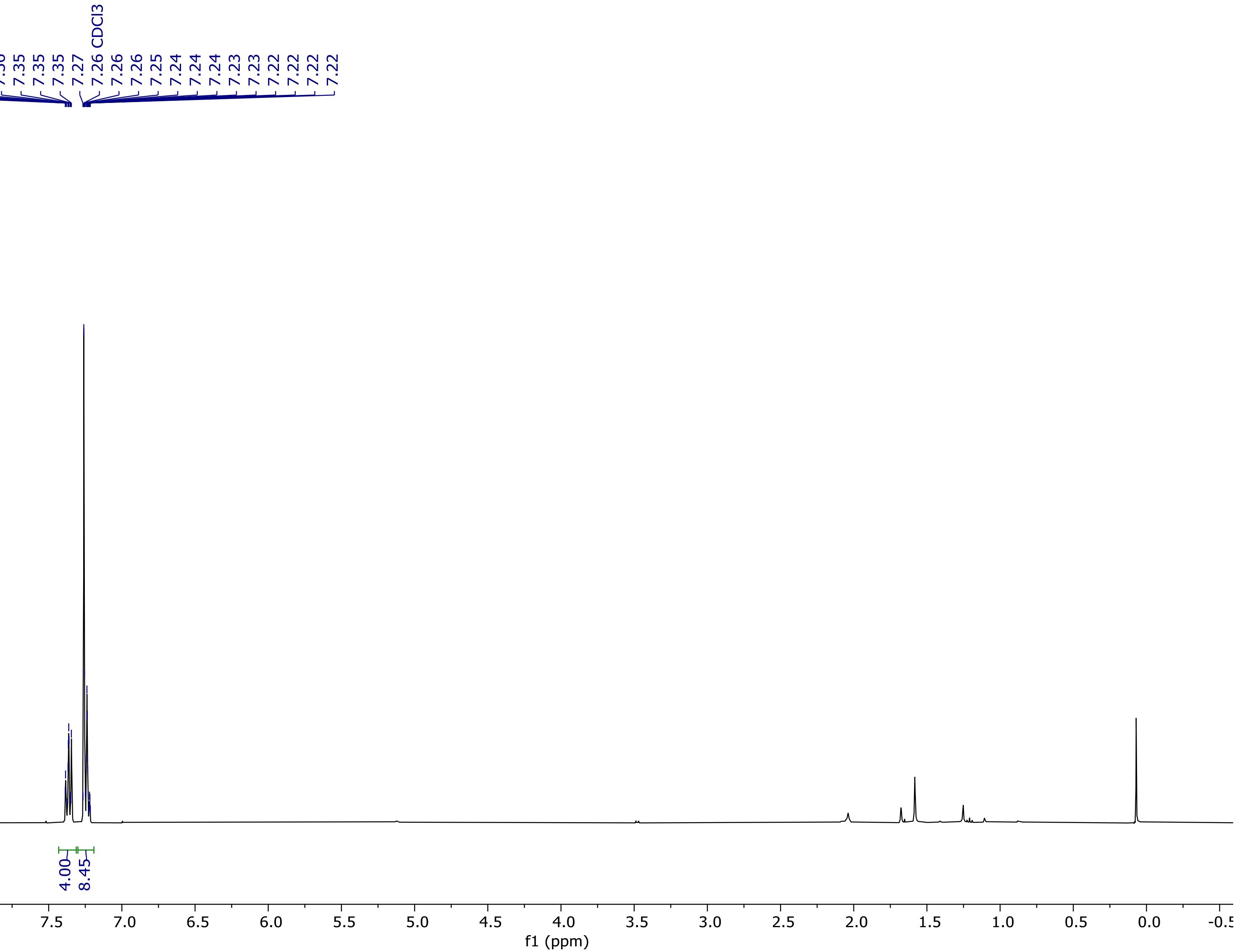

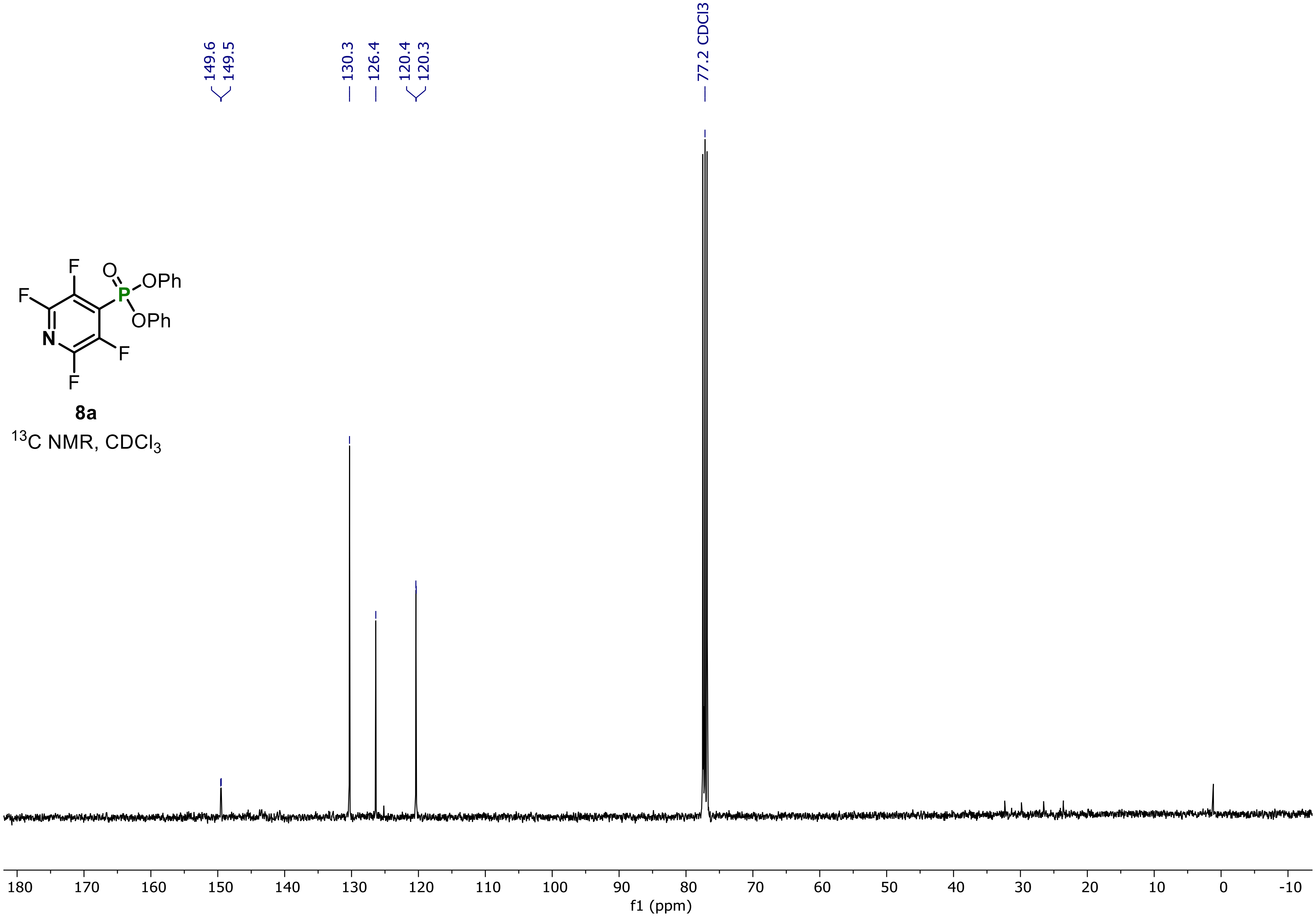

F
F
O
P
OPh
OPh
N
F
F
8a
13C NMR, CDCl3
149.6
149.5
130.3
126.4
120.4
120.3
77.2 CDCl3
180 170 160 150 140 130 120 110 100 90 80 70 60 50 40 30 20 10 0 -10
f1 (ppm)

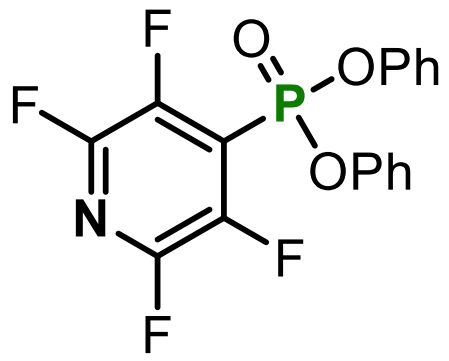

8a

$^{19}F\{^1H\}$ NMR, $CDCl_3$

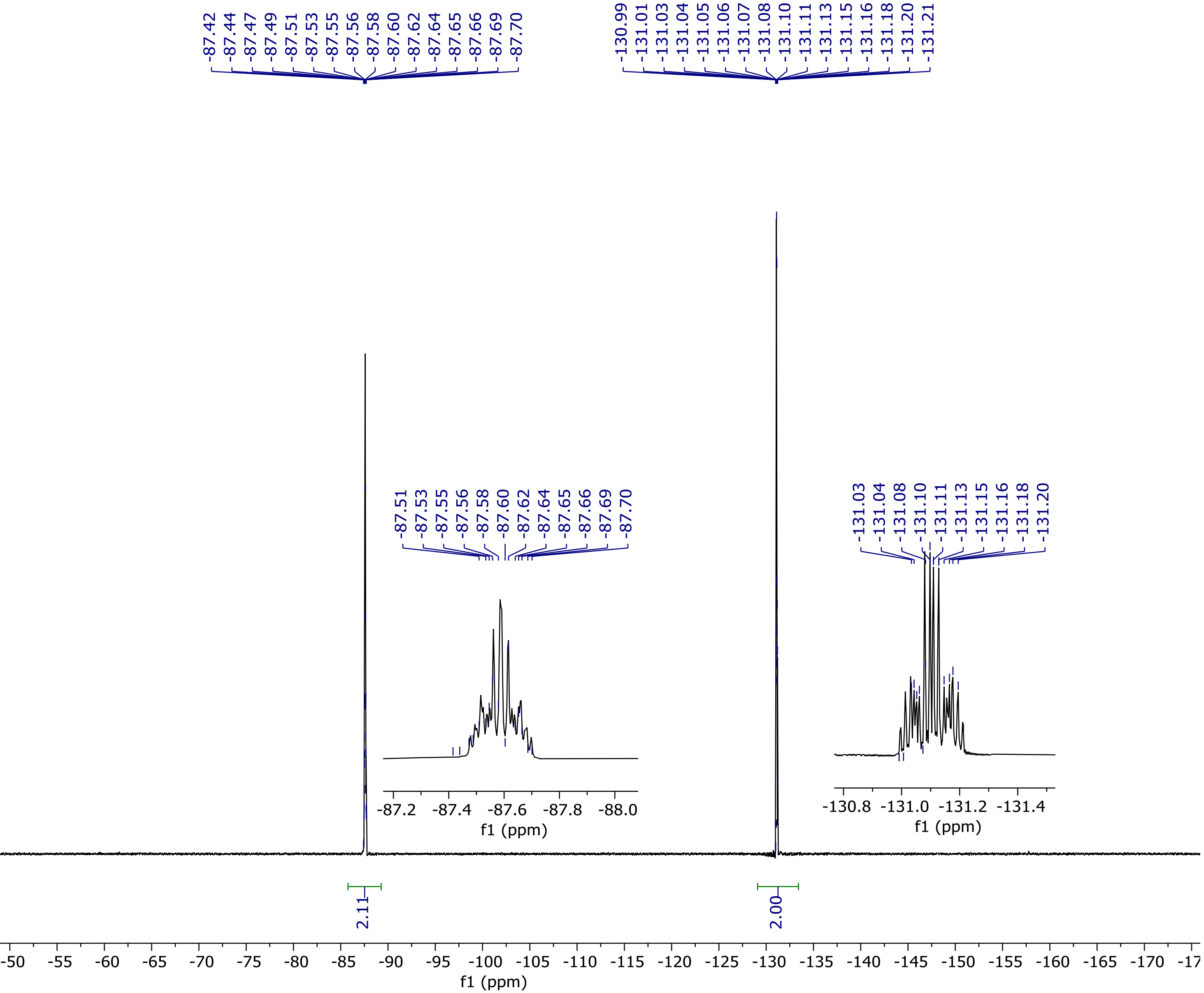

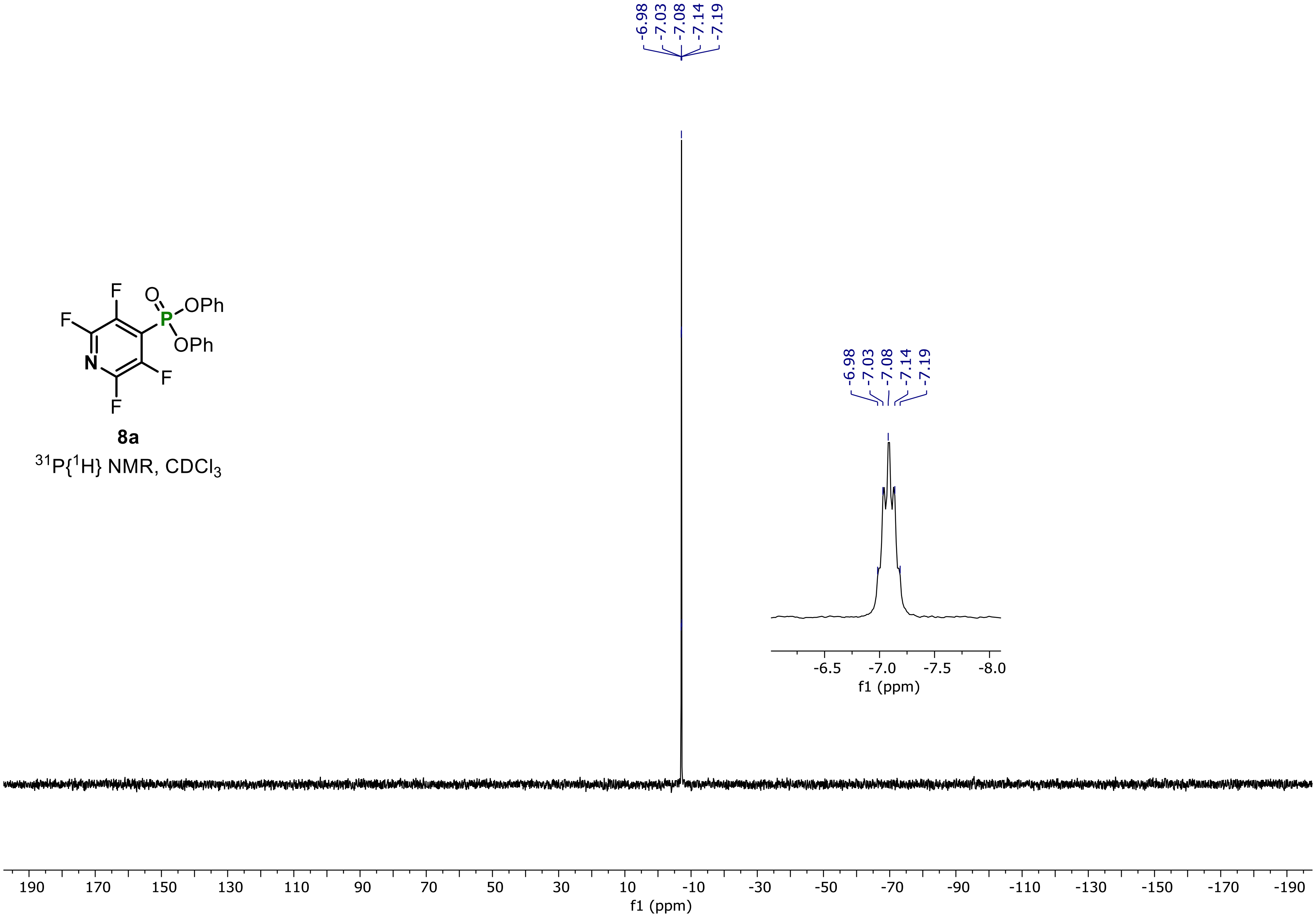

8a

$^{31}P\{^{1}H\}$ NMR, $CDCl_3$

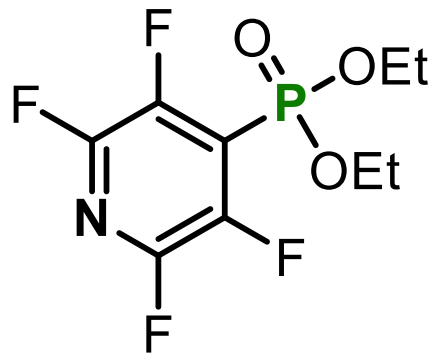

8b

^{1}H NMR, $CDCl_3$

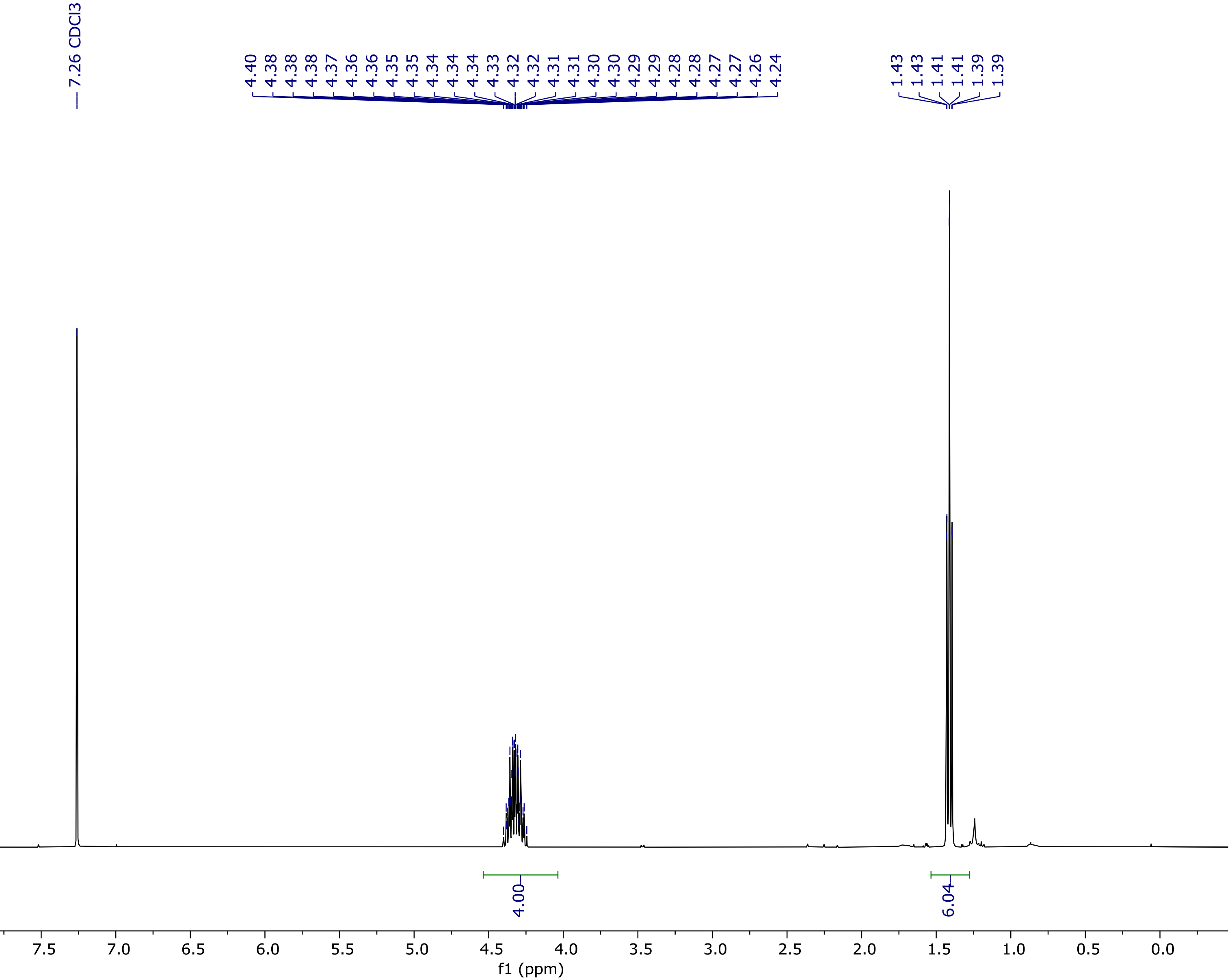

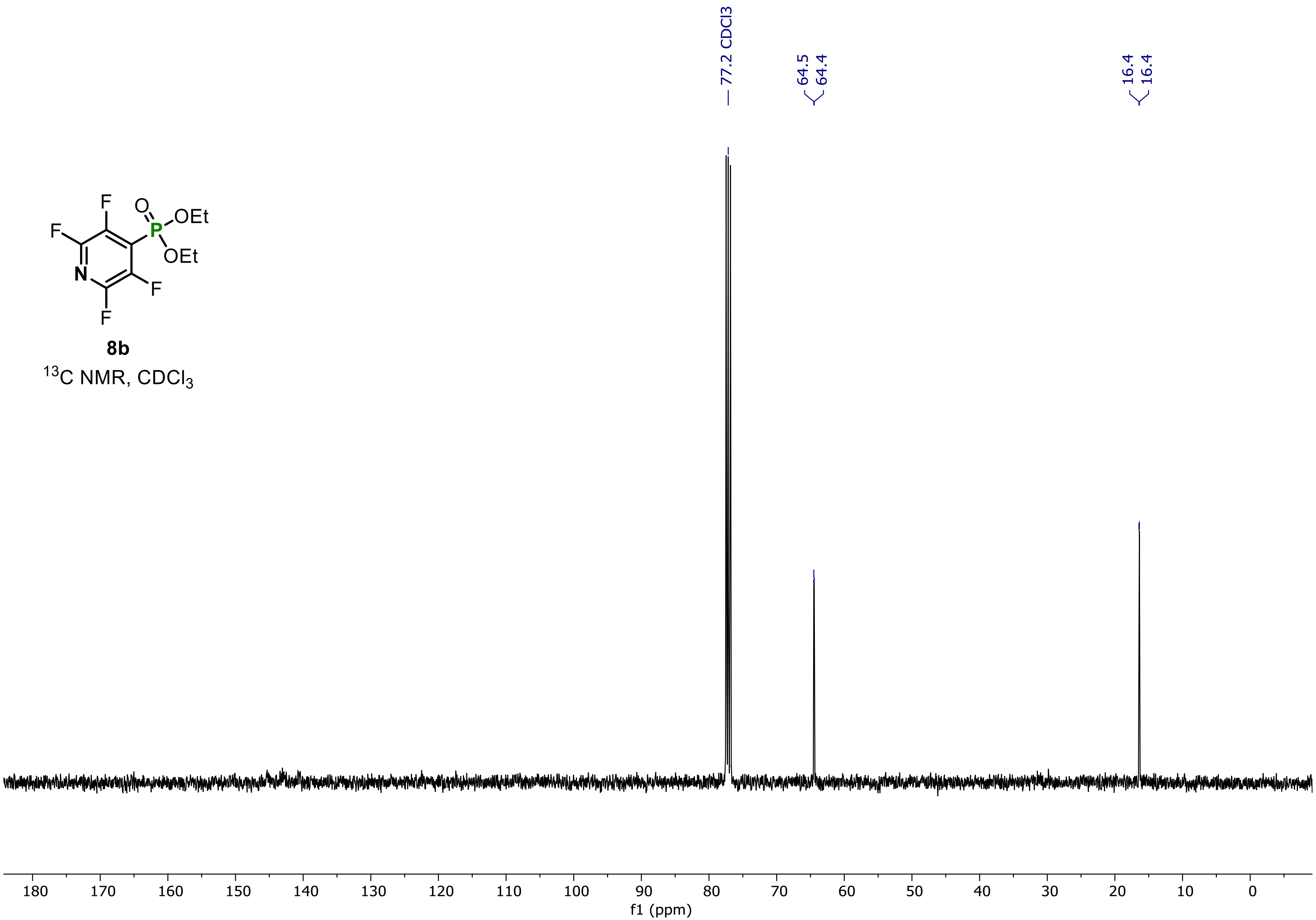

8b

^{13}C NMR, $CDCl_3$

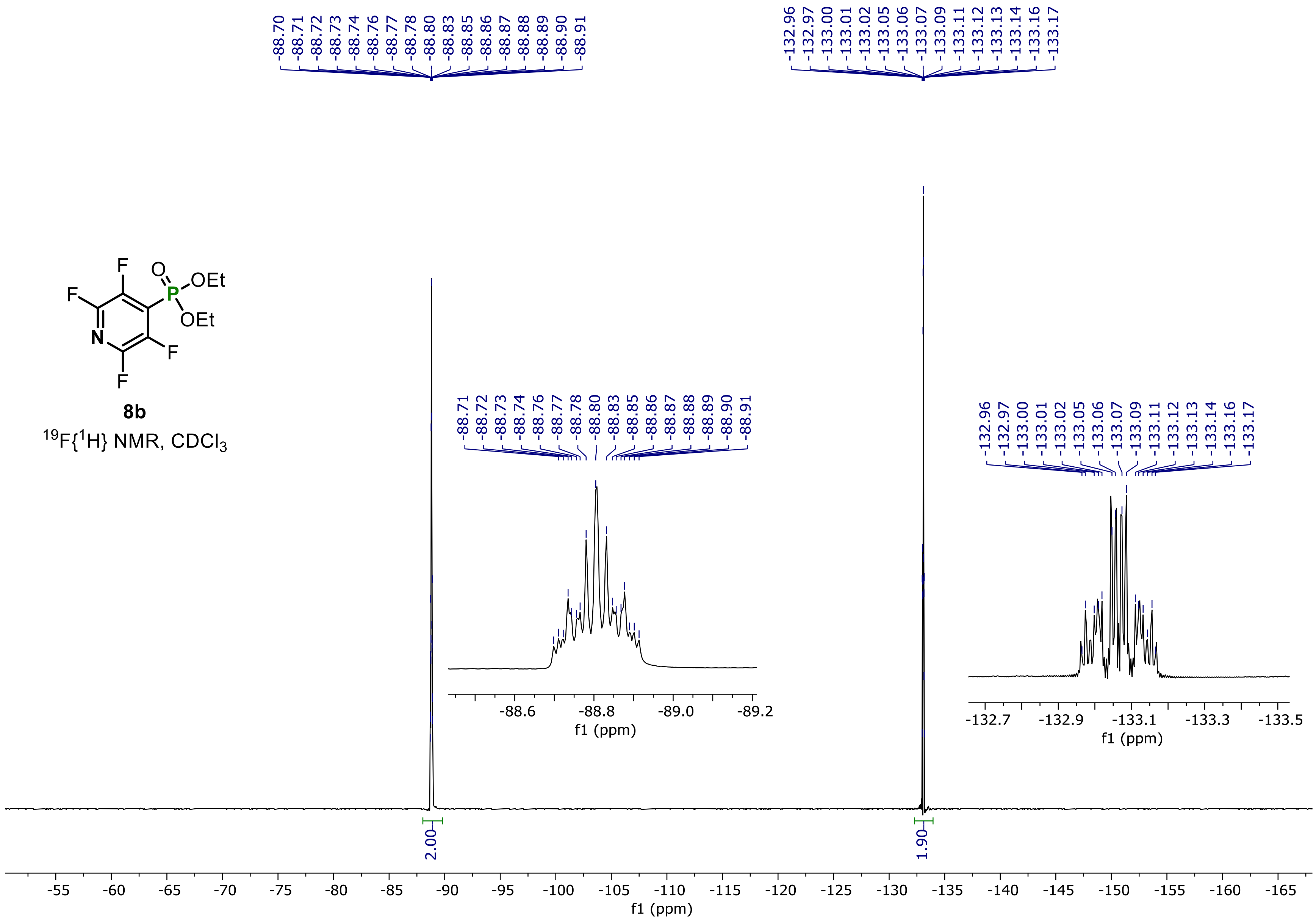

8b
$^{19}F\{^1H\}$ NMR, $CDCl_3$
f1 (ppm)
2.00
1.90

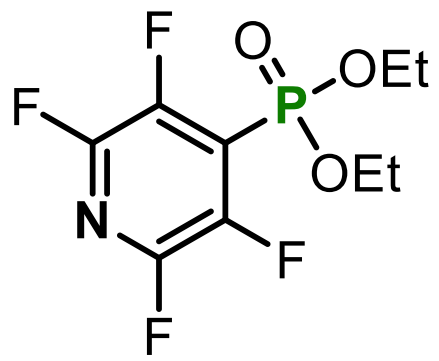

8b

$^{31}P\{^1H\}$ NMR, $CDCl_3$

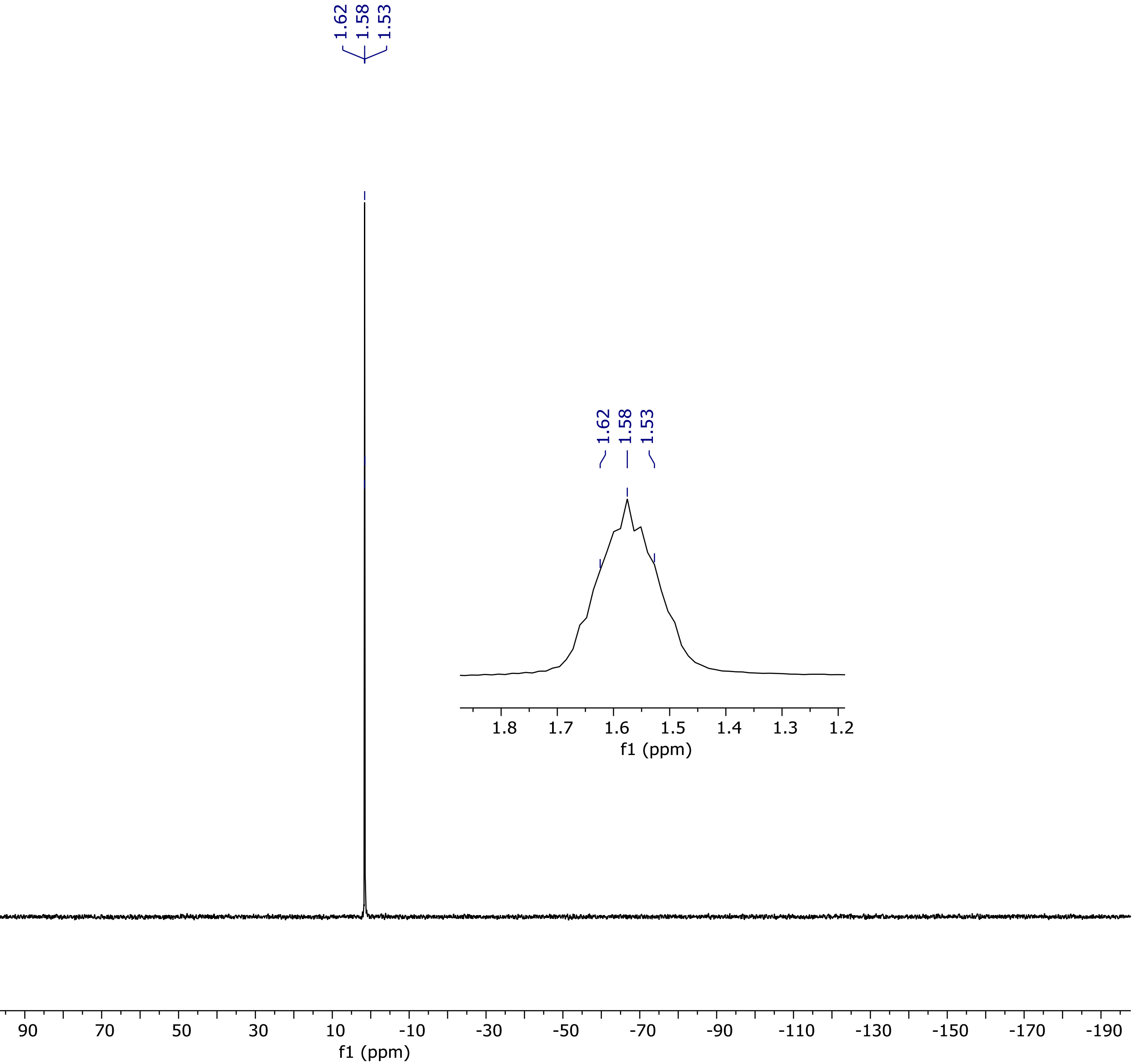

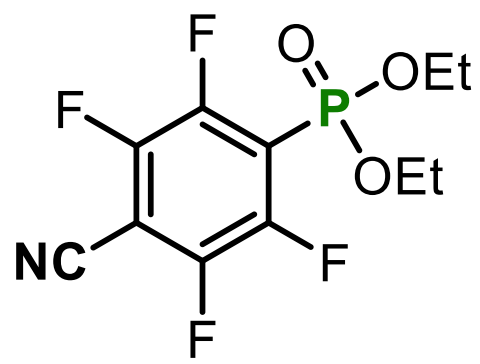

8c

^{1}H NMR, $CDCl_3$

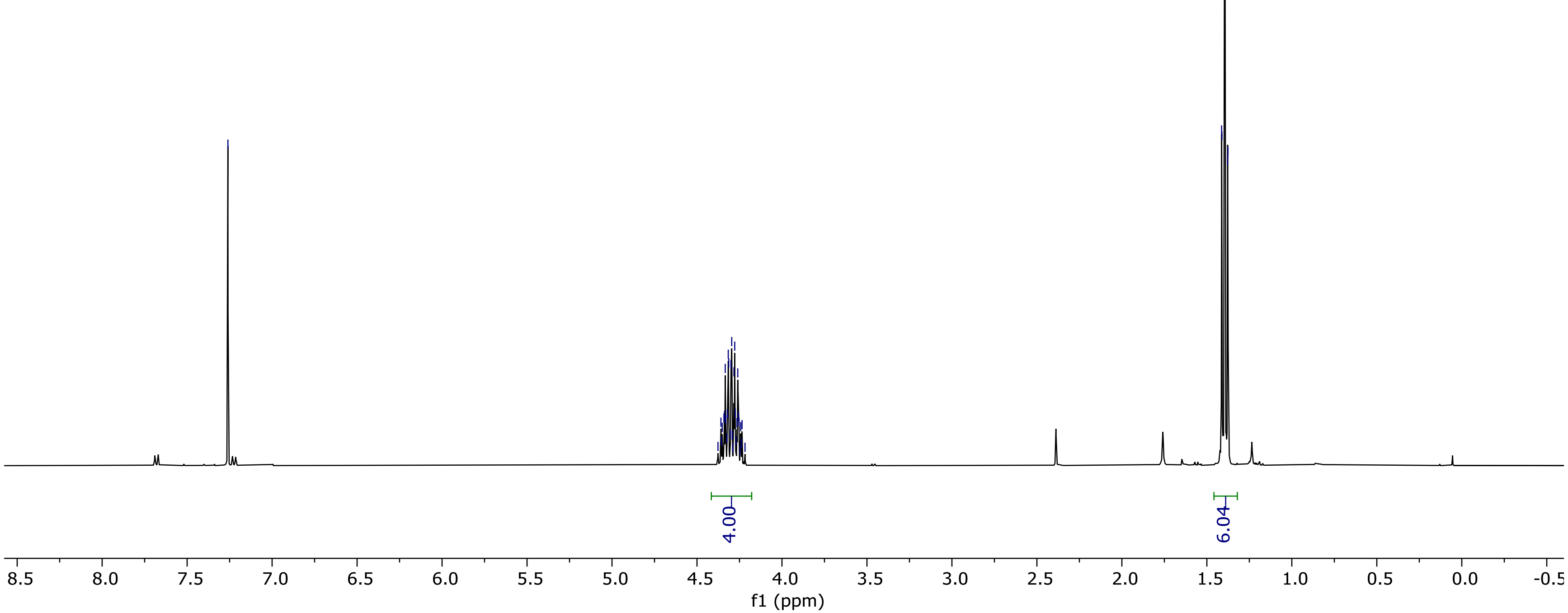

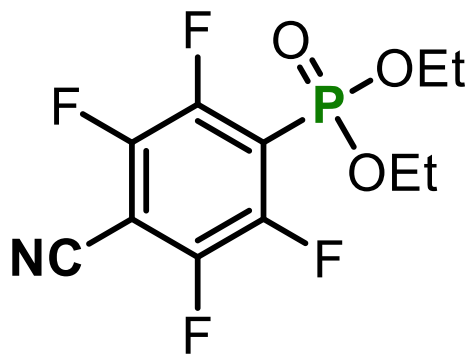

8c

^{13}C NMR, $CDCl_3$

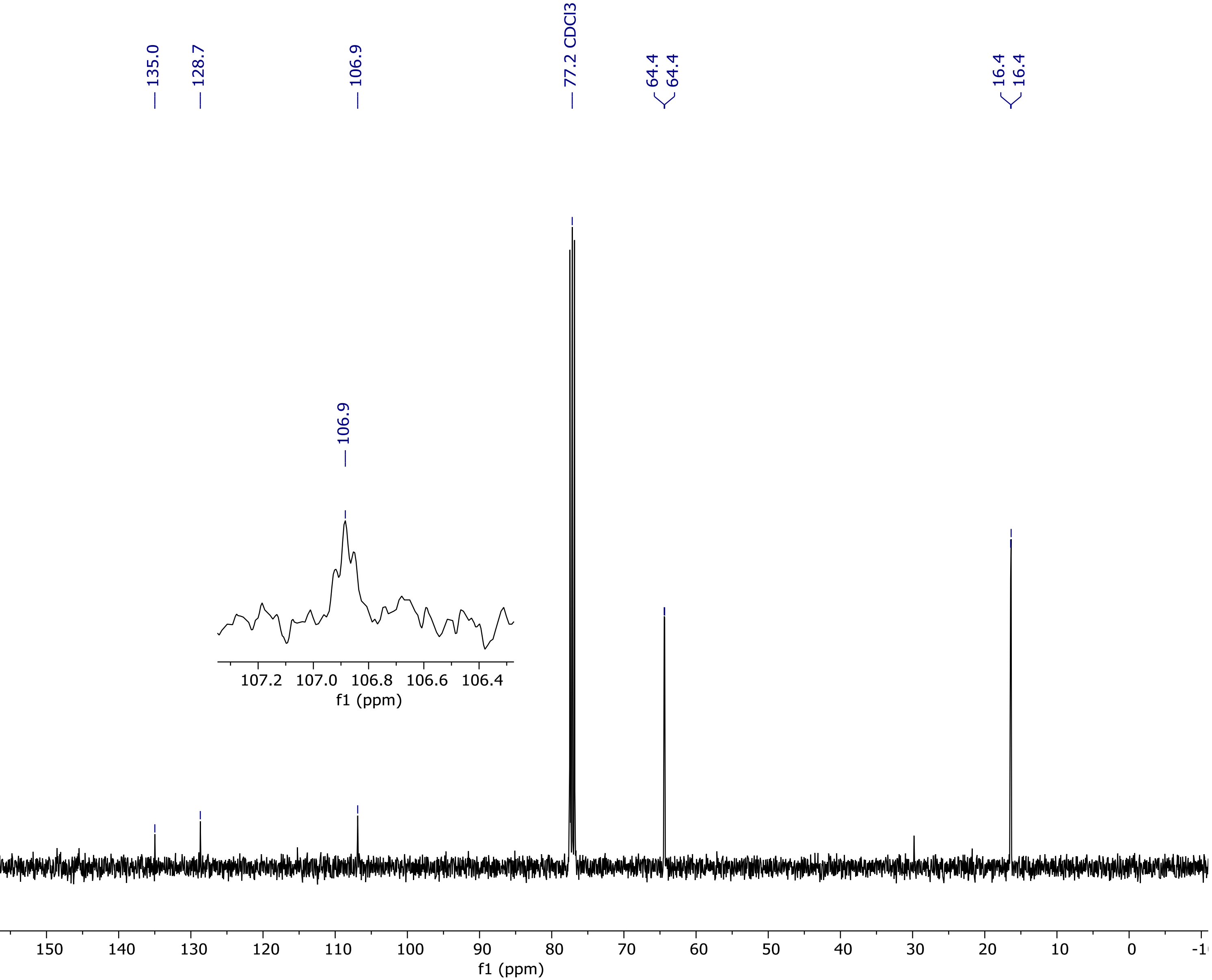

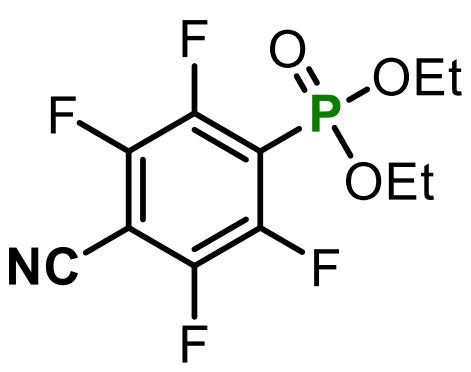

8c

$^{19}F\{^1H\}$ NMR, $CDCl_3$

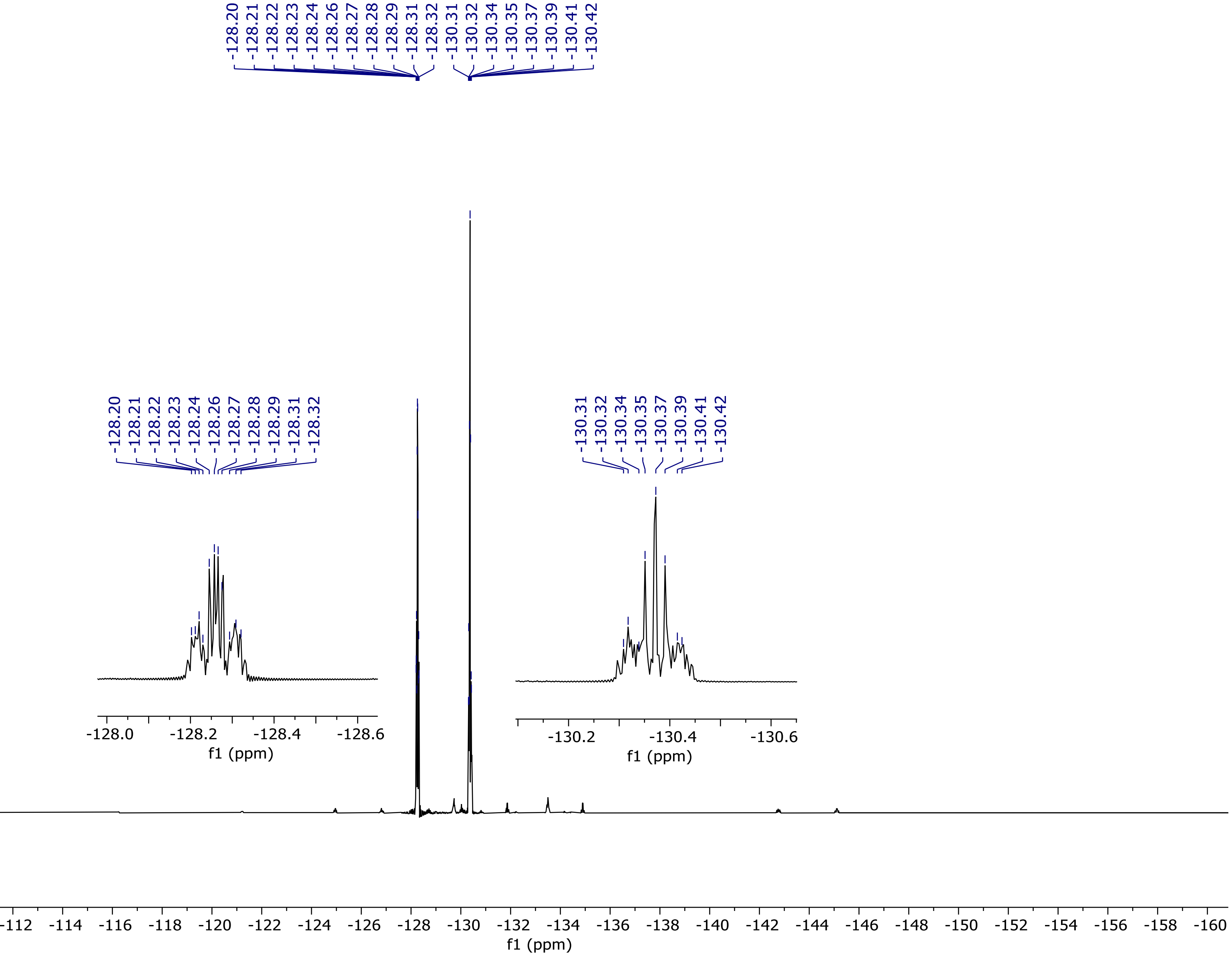

8c

$^{31}P\{^{1}H\}$ NMR, $CDCl_3$

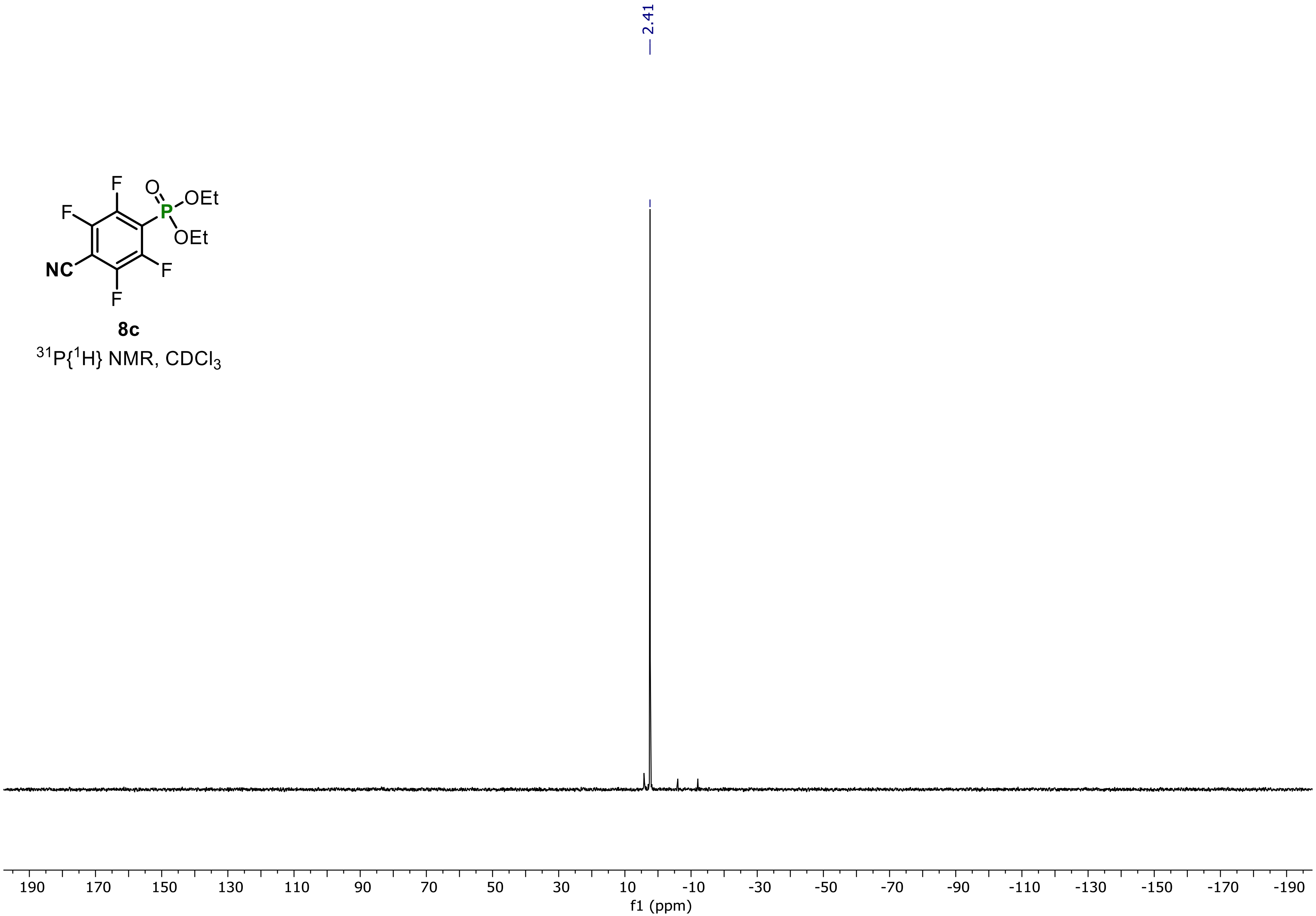

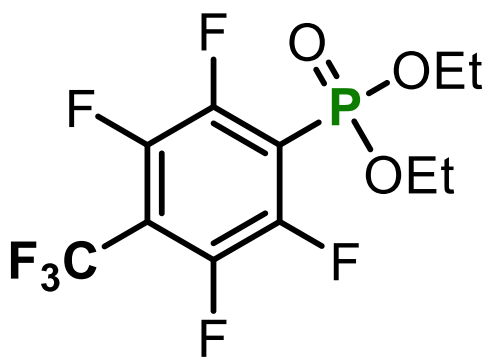

8d

^{1}H NMR, $CDCl_3$

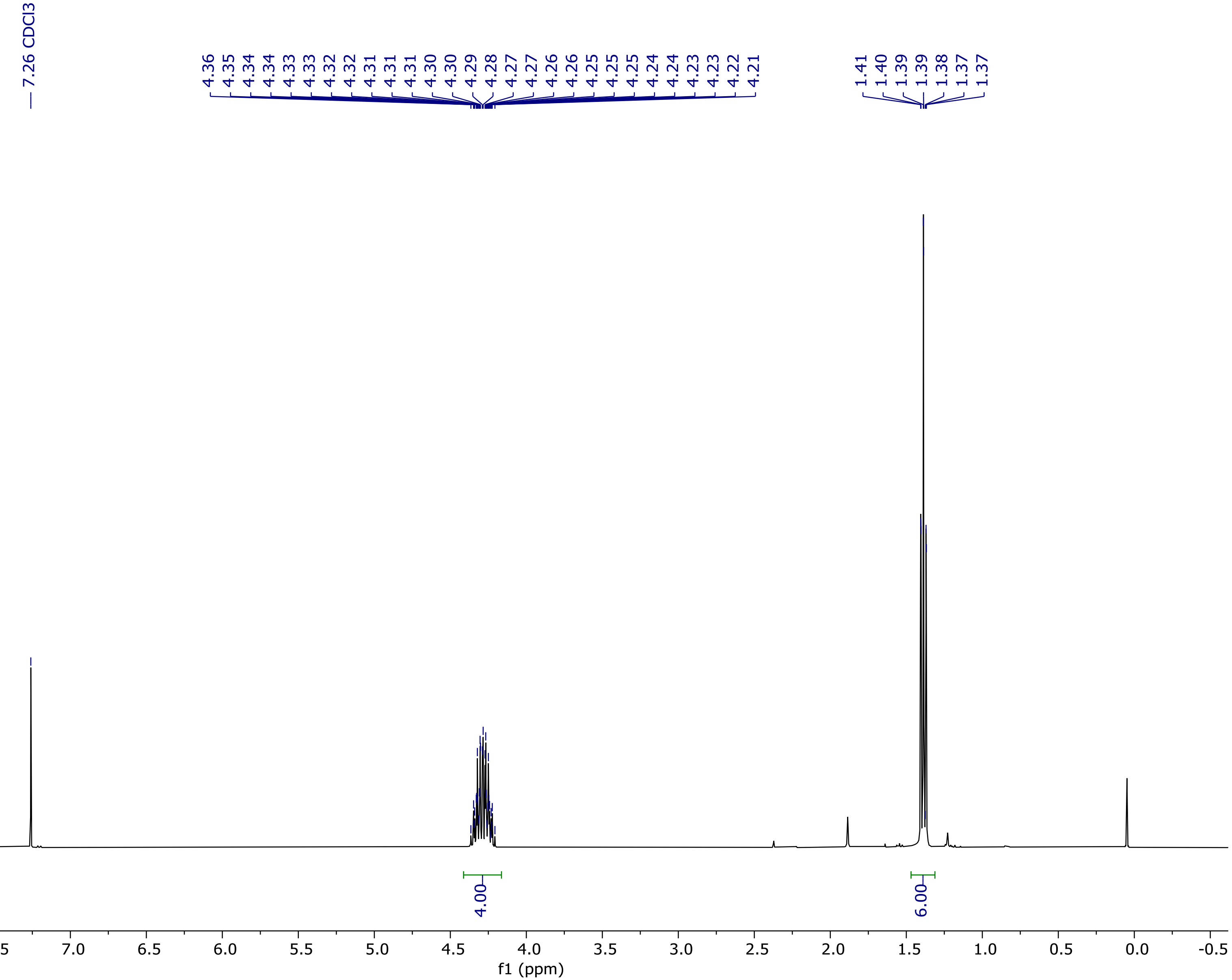

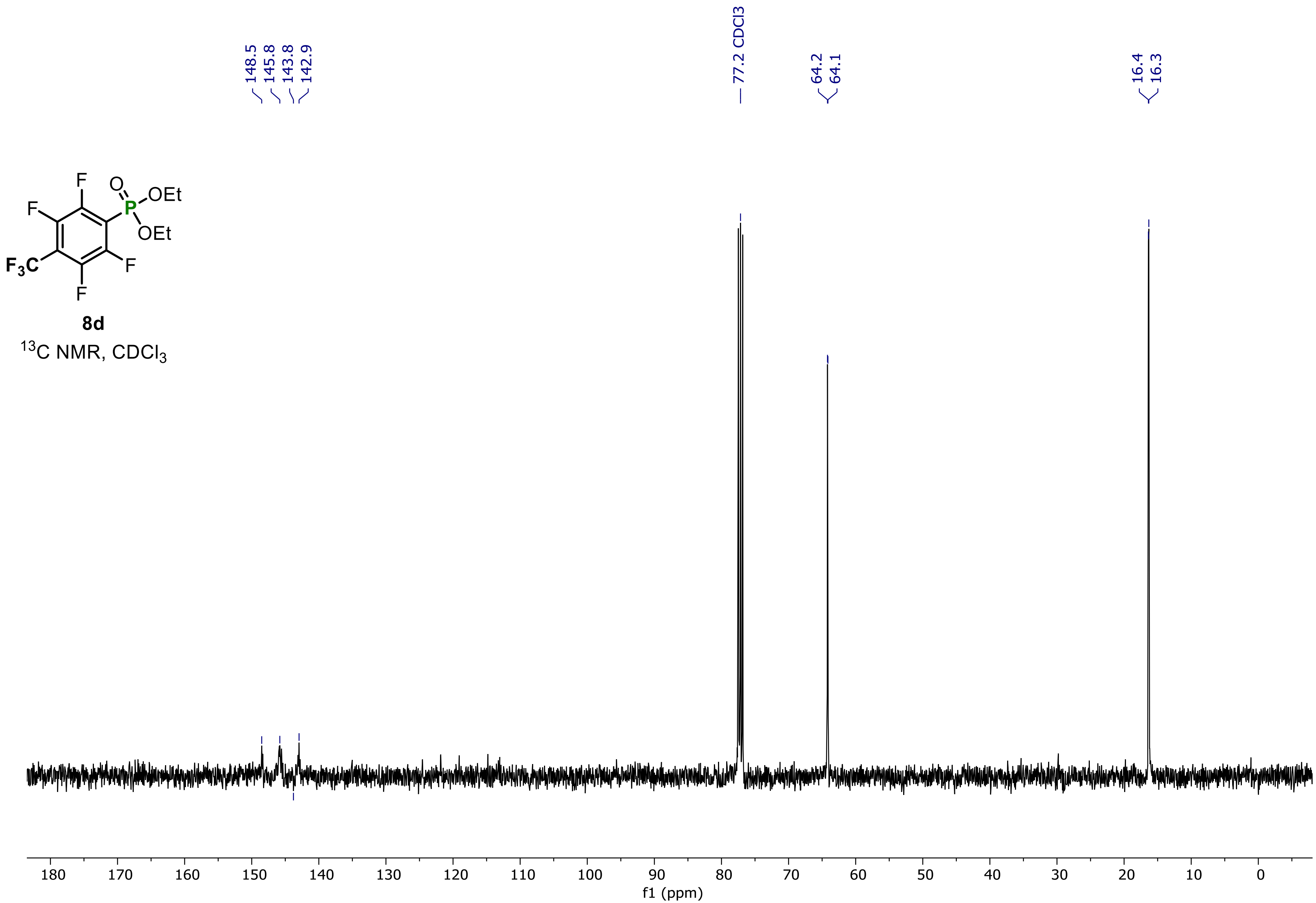

8d

^{13}C NMR, $CDCl_3$

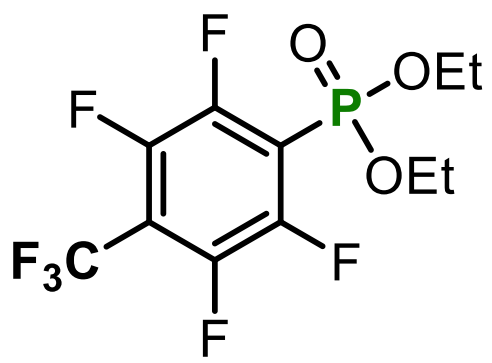

8d

$^{19}F\{^{1}H\}$ NMR, $CDCl_3$

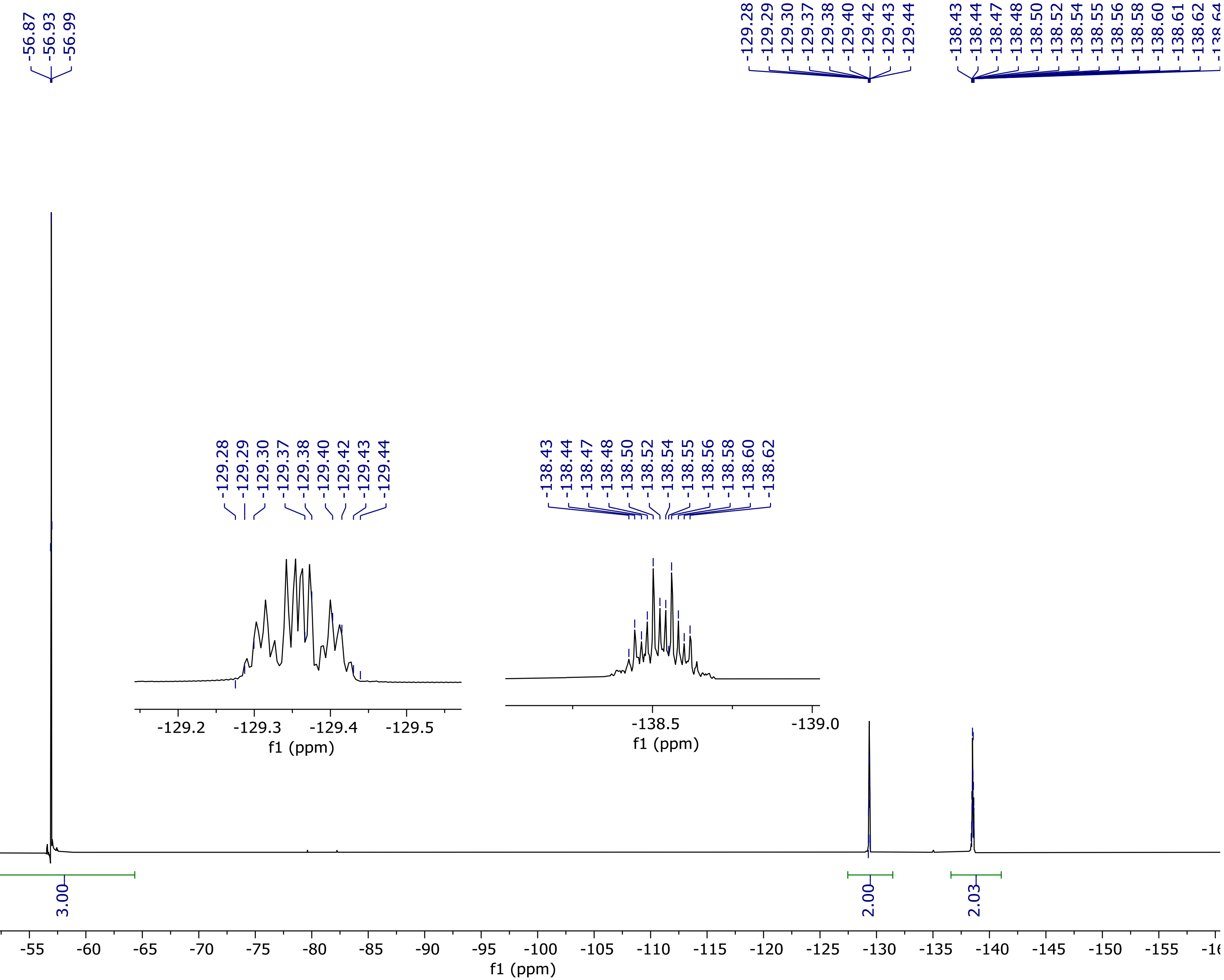

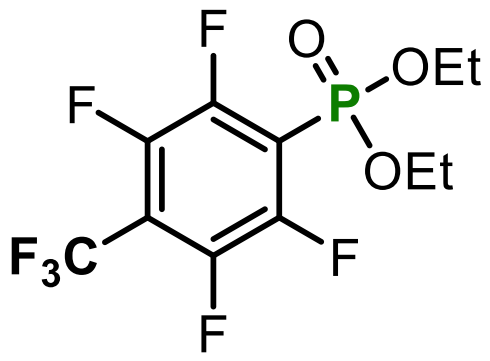

8d

$^{31}P\{^{1}H\}$ NMR, $CDCl_3$

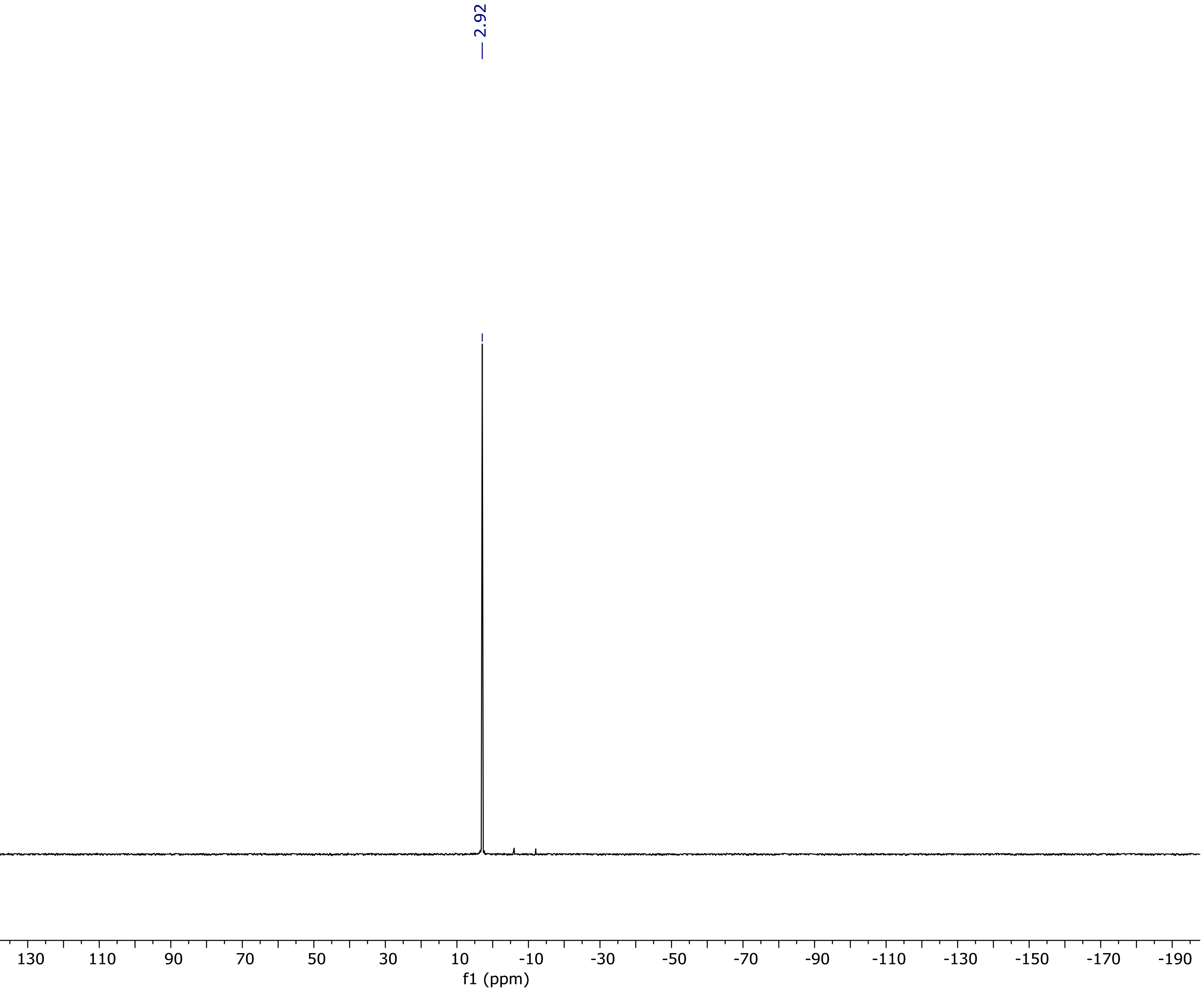

7.5 NMR characterization of defluoroselenation products

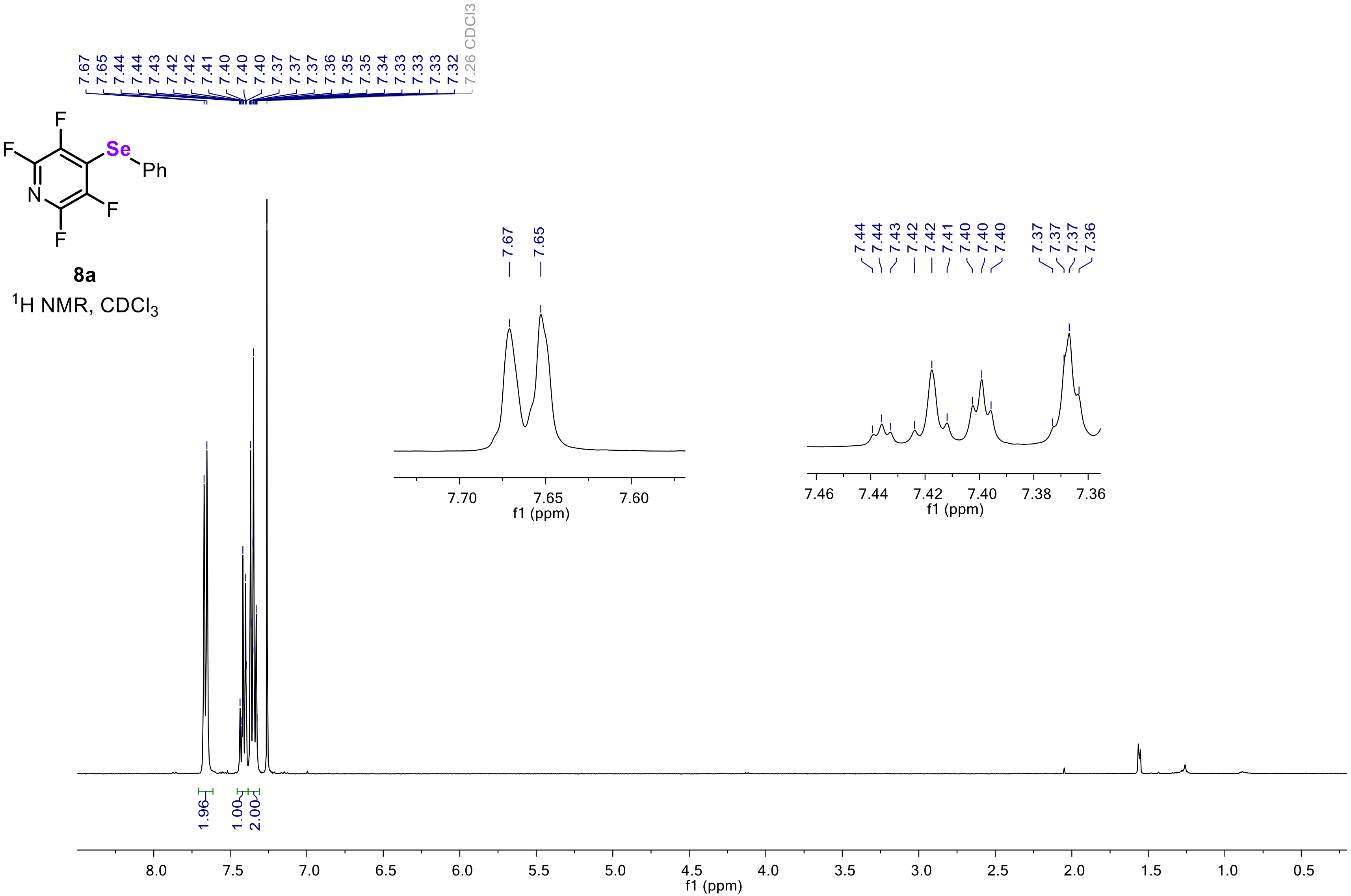

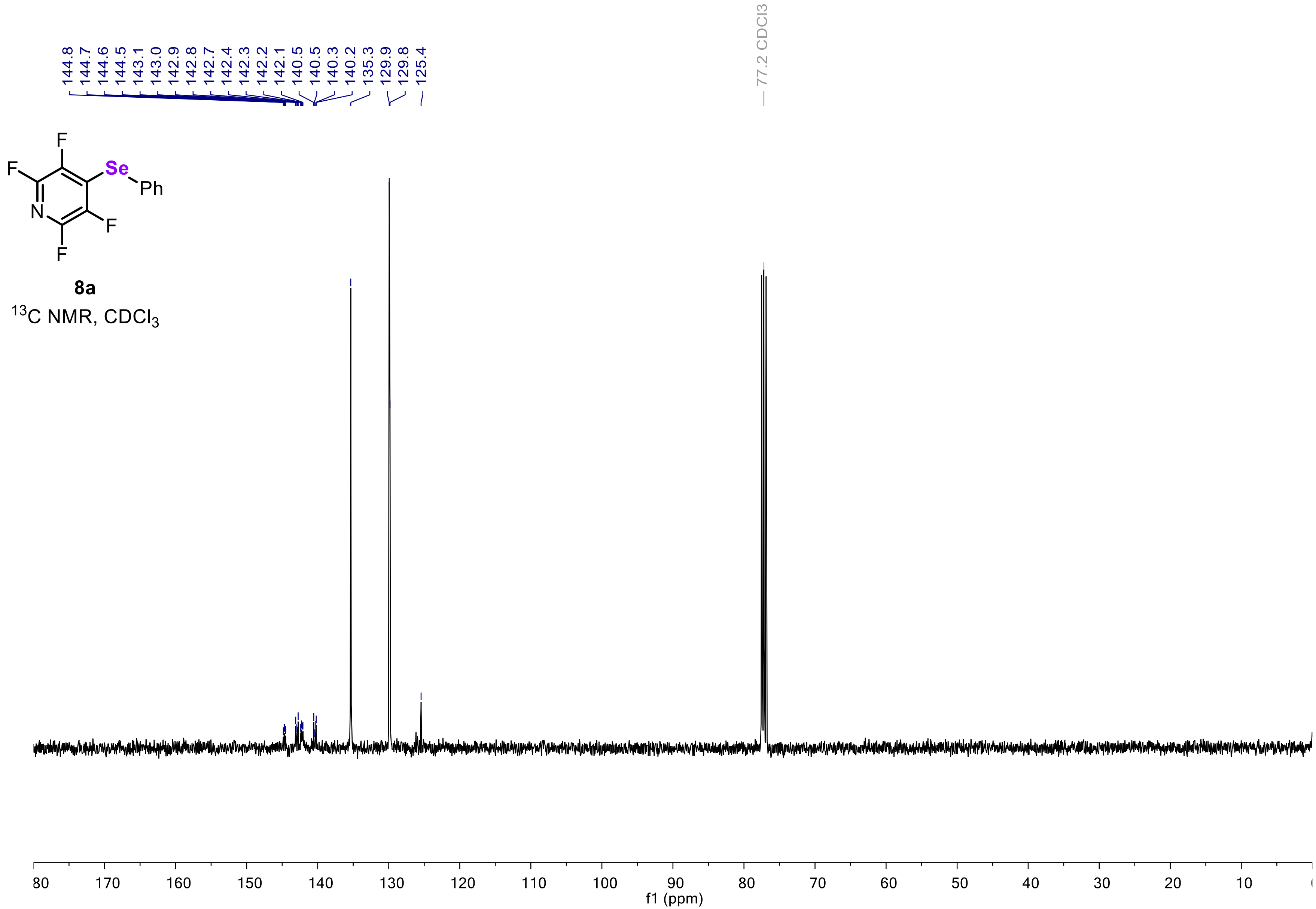

8a

^{13}C NMR, $CDCl_3$

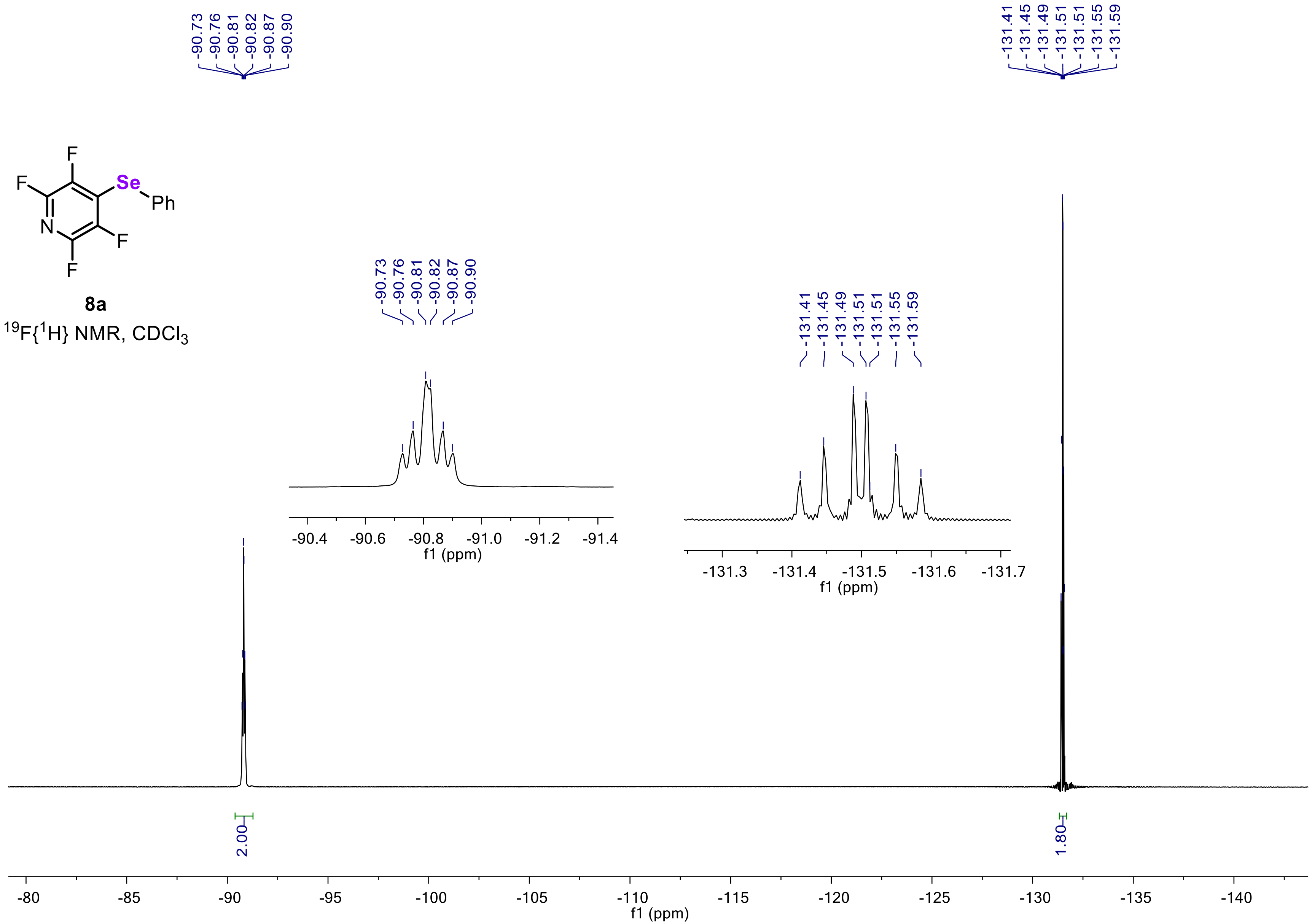

-90.73
-90.76
-90.81
-90.82
-90.87
-90.90
-131.41
-131.45
-131.49
-131.51
-131.51
-131.55
-131.59
F
F
Se
Ph
N
F
F
8a
19F{1H} NMR, CDCl3
f1 (ppm)
2.00
1.80

7.6 NMR characterization of defluoroarylation products

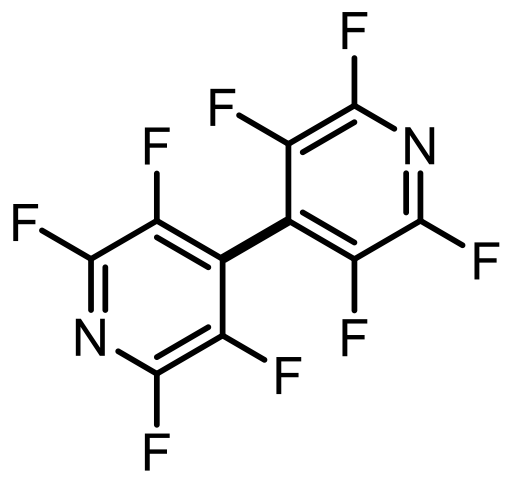

10a

$^{19}F\{^{1}H\}$ NMR, $CDCl_3$

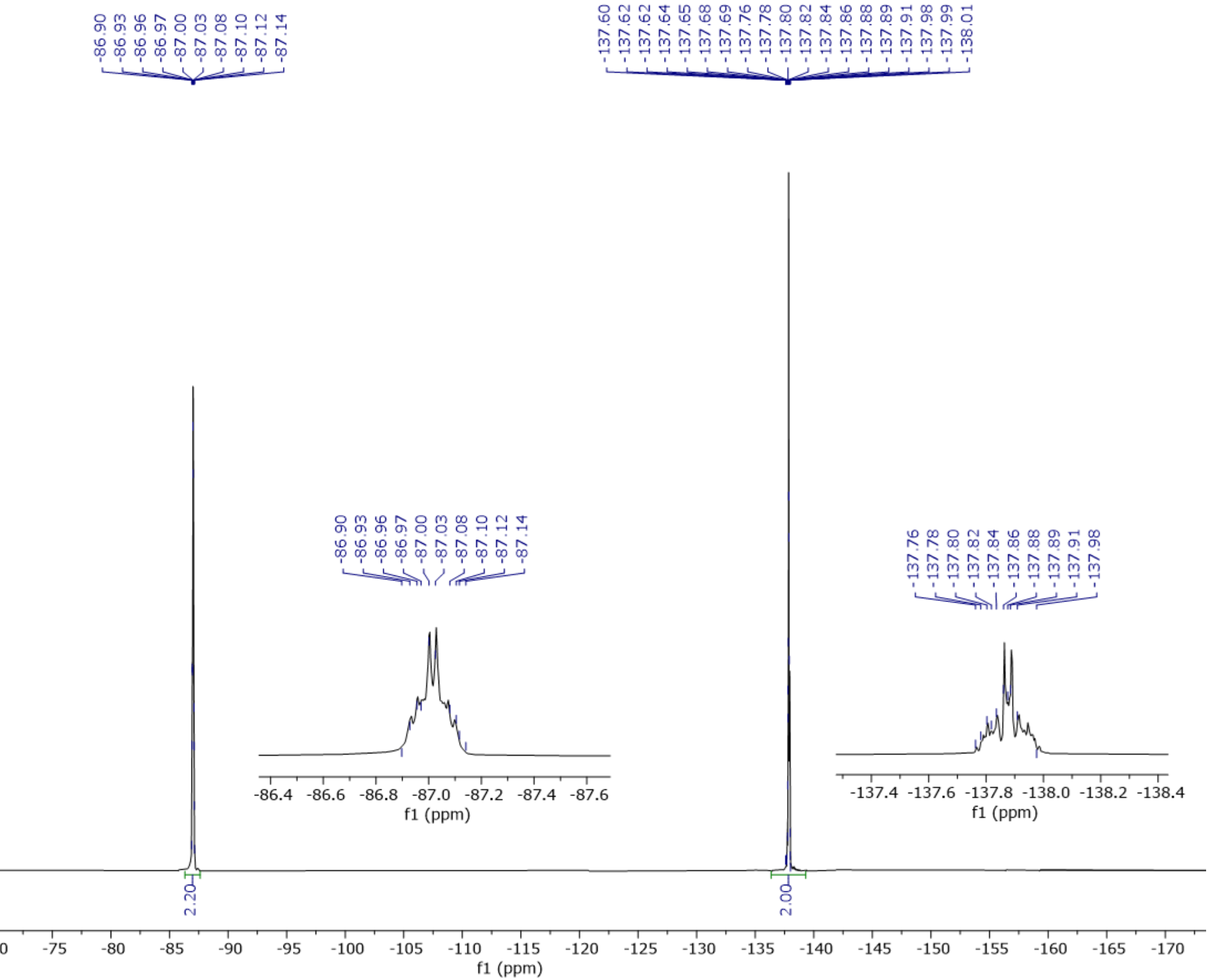

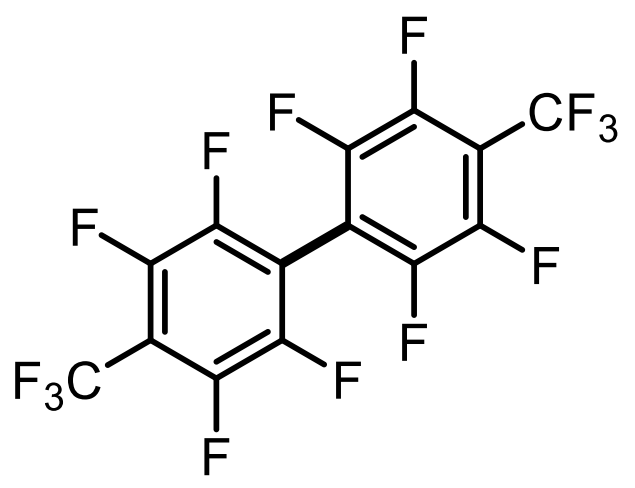

10b

$^{19}F\{1H\}$ NMR, $CDCl_3$

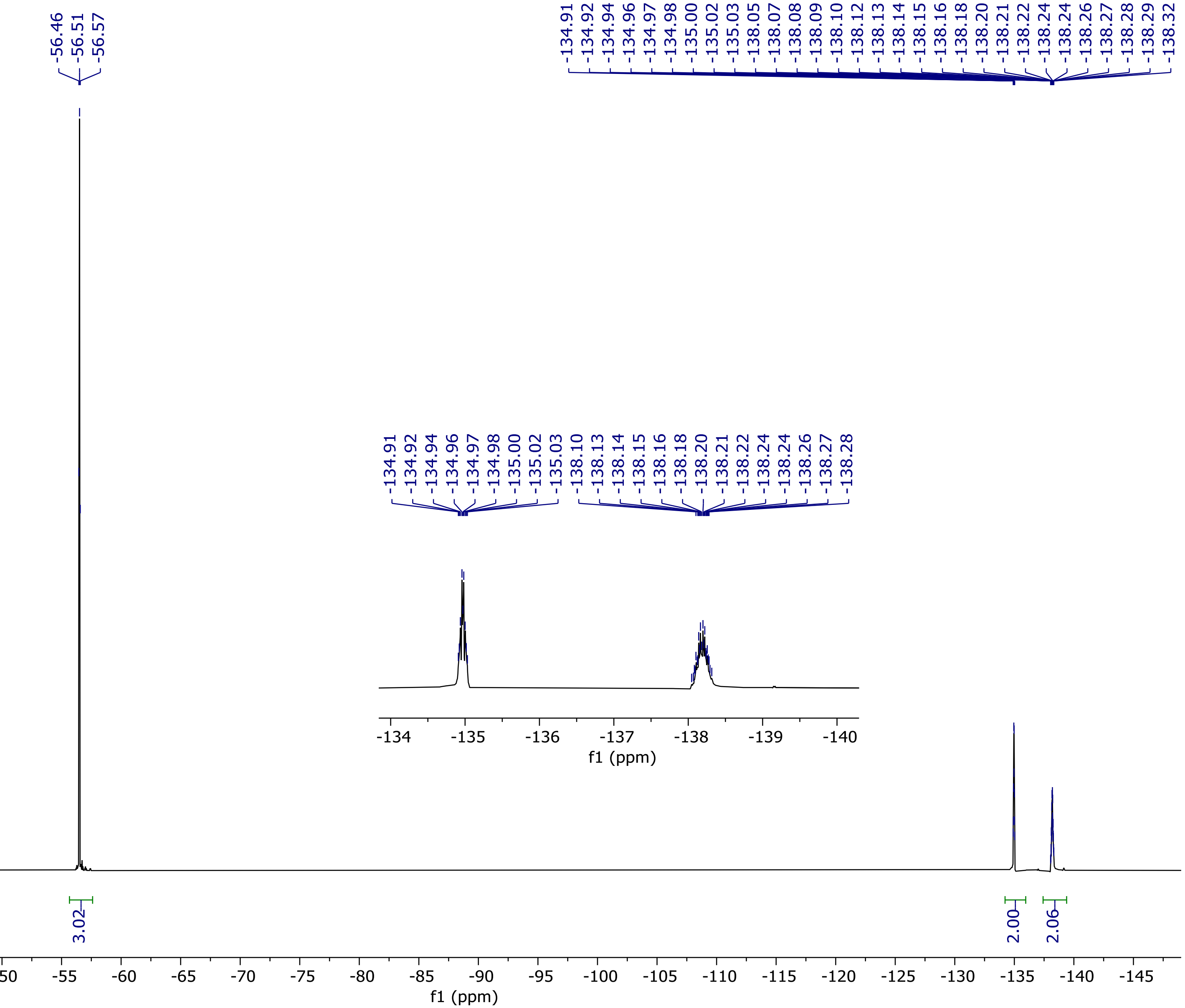

7.7 NMR characterization of Sb(III) relevant species 14 and 15

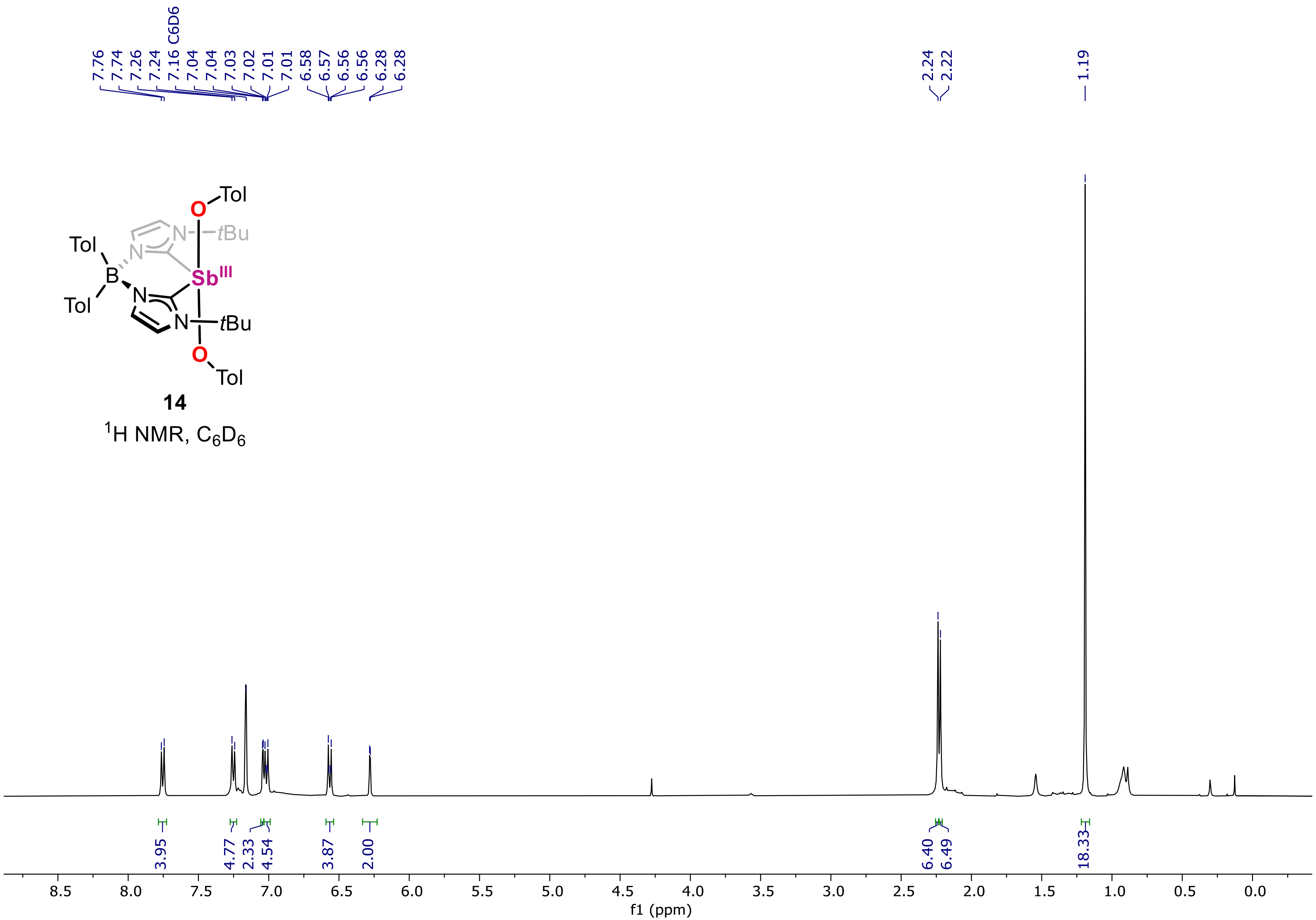

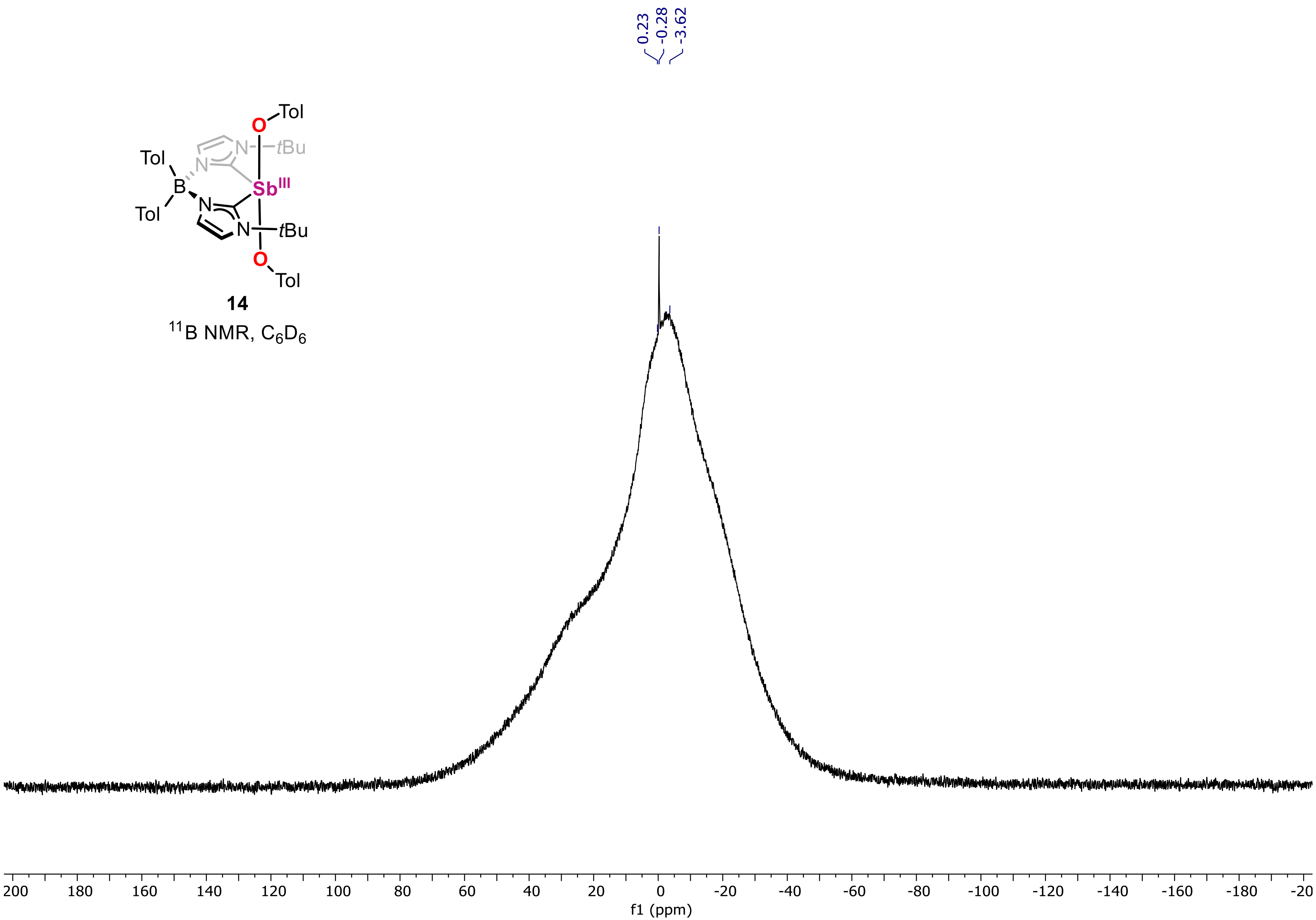

14

^{11}B NMR, C_6D_6

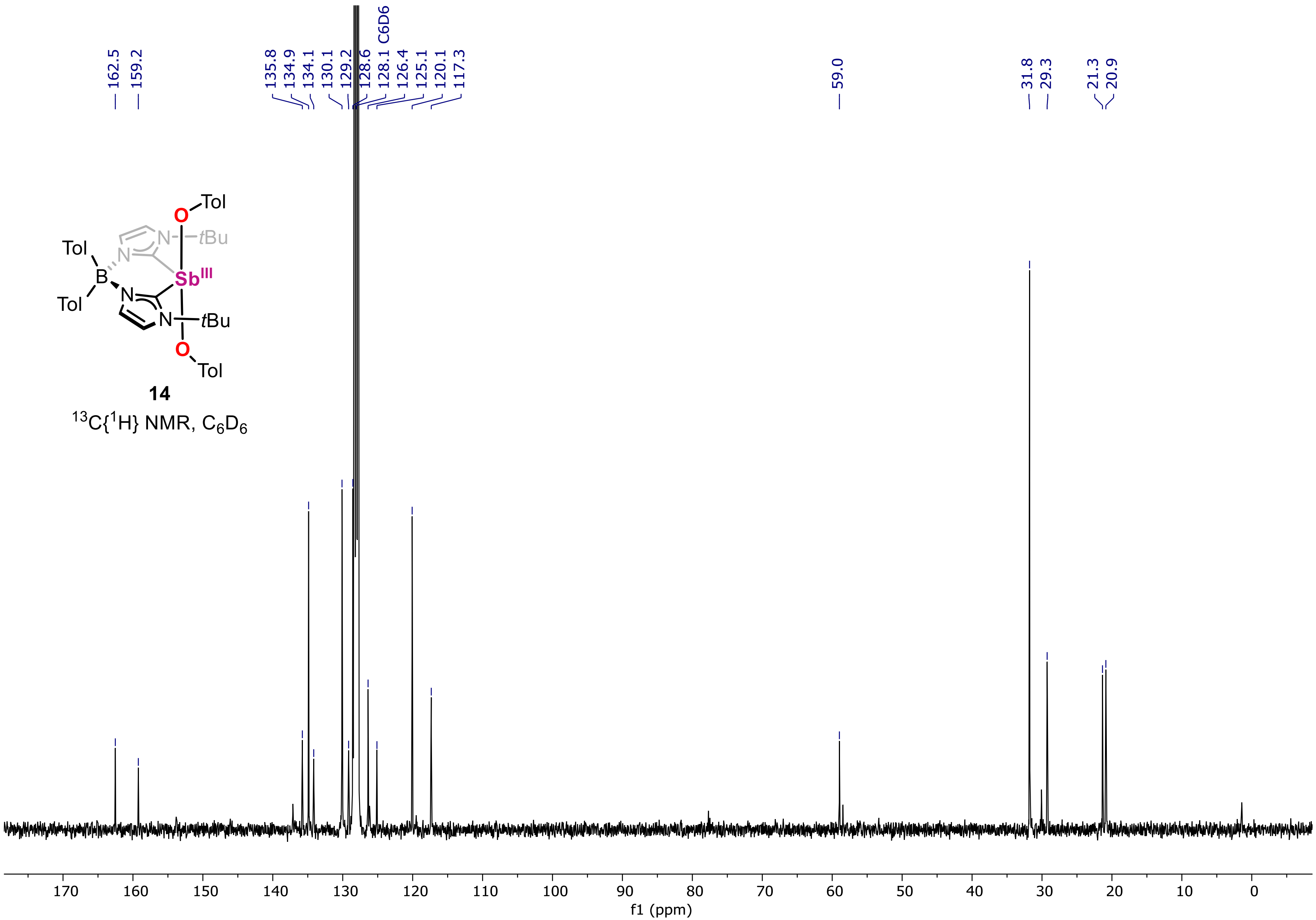
Tol
O
N
N
tBu
Tol
B
Sb^III
Tol
N
N
tBu
O
Tol
14
13C{1H} NMR, C6D6
162.5
159.2
135.8
134.9
134.1
130.1
129.2
128.6
128.1 C6D6
126.4
125.1
120.1
117.3
59.0
31.8
29.3
21.3
20.9
170
160
150
140
130
120
110
100
90
80
70
60
50
40
30
20
10
0
f1 (ppm)

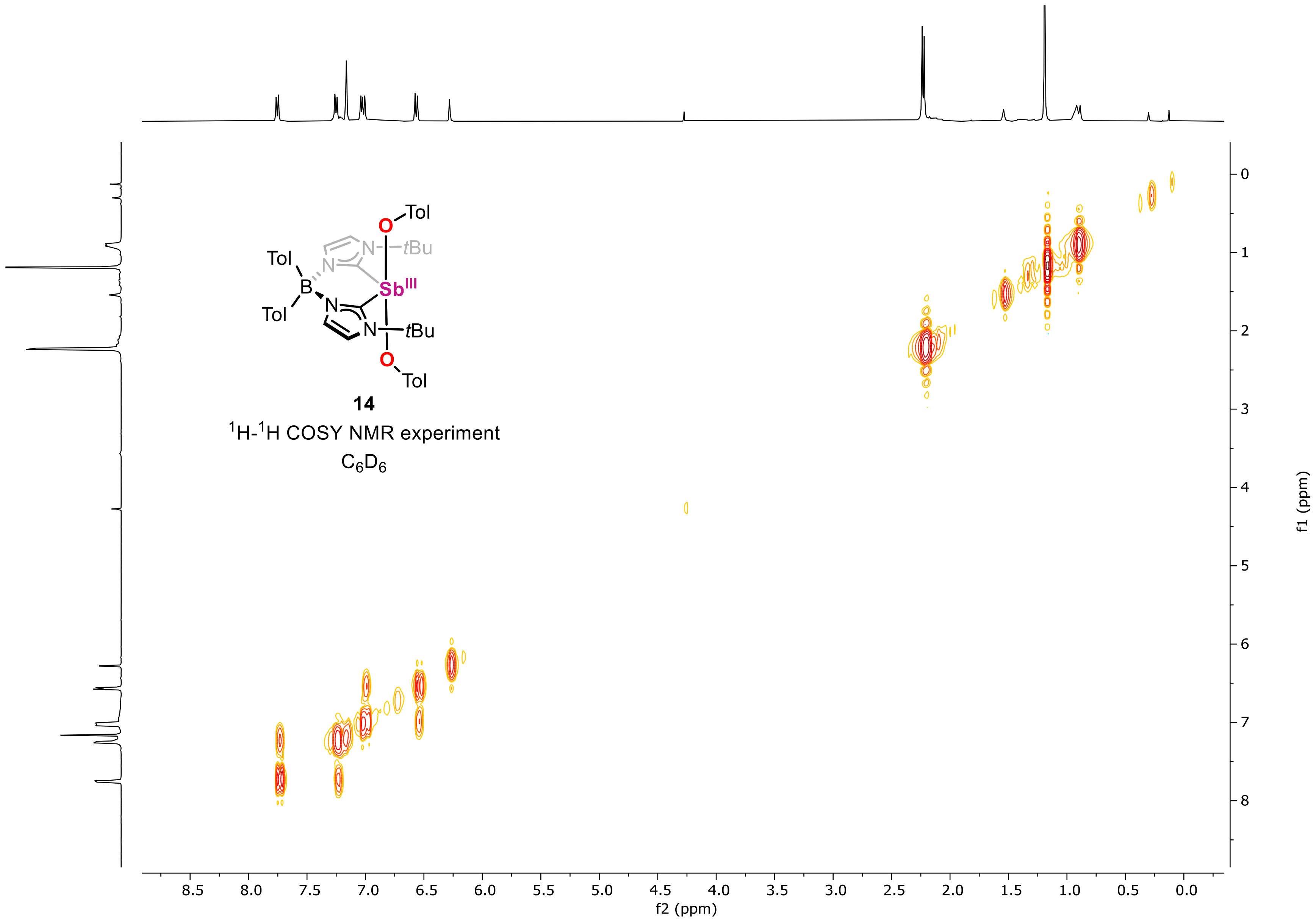

Tol
O
tBu
N
Tol
B
Sb^III
Tol
N
N
tBu
O
Tol
14
1H-1H COSY NMR experiment
C6D6
f2 (ppm)
f1 (ppm)
8.5
8.0
7.5
7.0
6.5
6.0
5.5
5.0
4.5
4.0
3.5
3.0
2.5
2.0
1.5
1.0
0.5
0.0

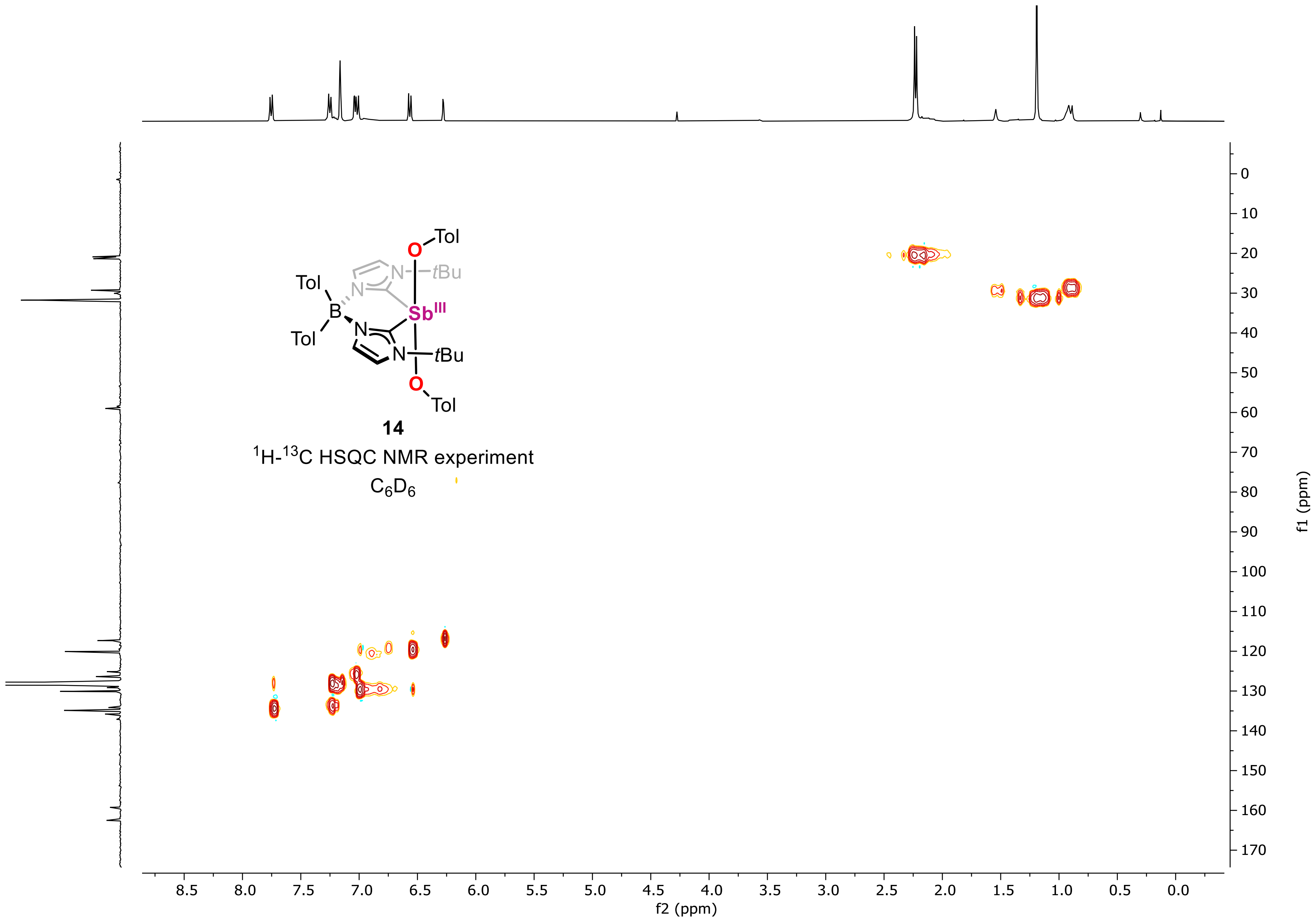
Tol
O
N
N
tBu
Tol
B
Sb^III
Tol
N
N
tBu
O
Tol
14
1H-13C HSQC NMR experiment
C6D6
f1 (ppm)
f2 (ppm)

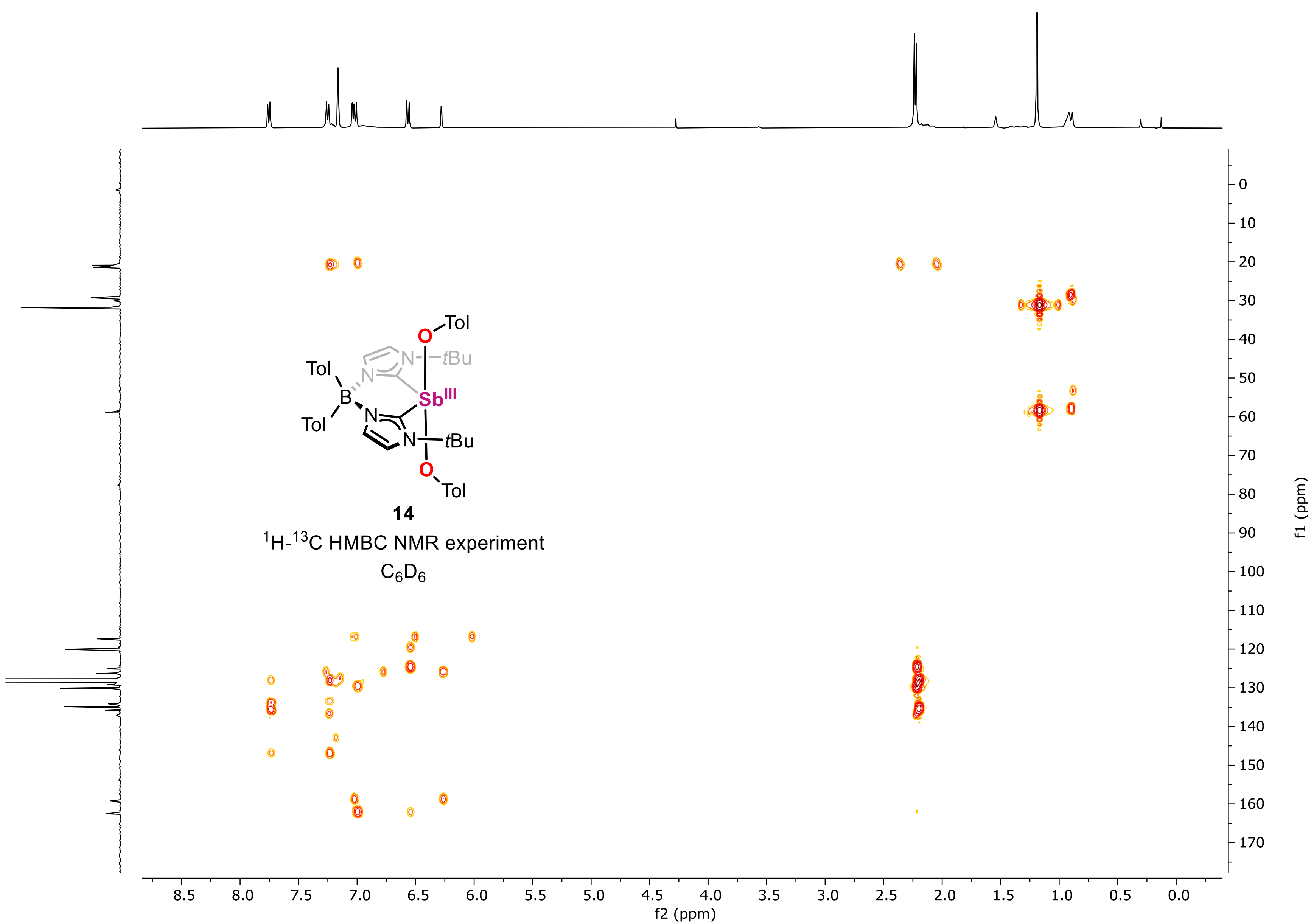
Tol
Tol
Tol
tBu
Sb^III
B
N
O
Tol
tBu
14
1H-13C HMBC NMR experiment
C6D6
f1 (ppm)
f2 (ppm)
0
10
20
30
40
50
60
70
80
90
100
110
120
130
140
150
160
170
8.5
8.0
7.5
7.0
6.5
6.0
5.5
5.0
4.5
4.0
3.5
3.0
2.5
2.0
1.5
1.0
0.5
0.0

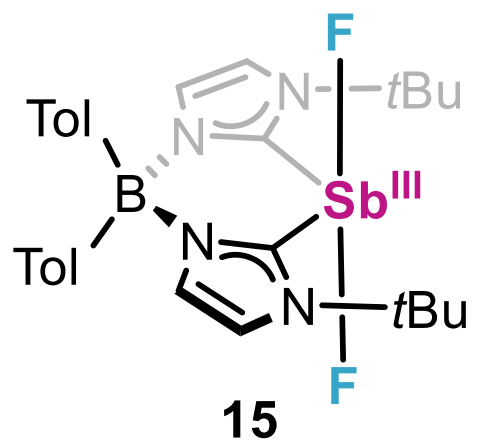

15

^{1}H NMR, C_6D_6

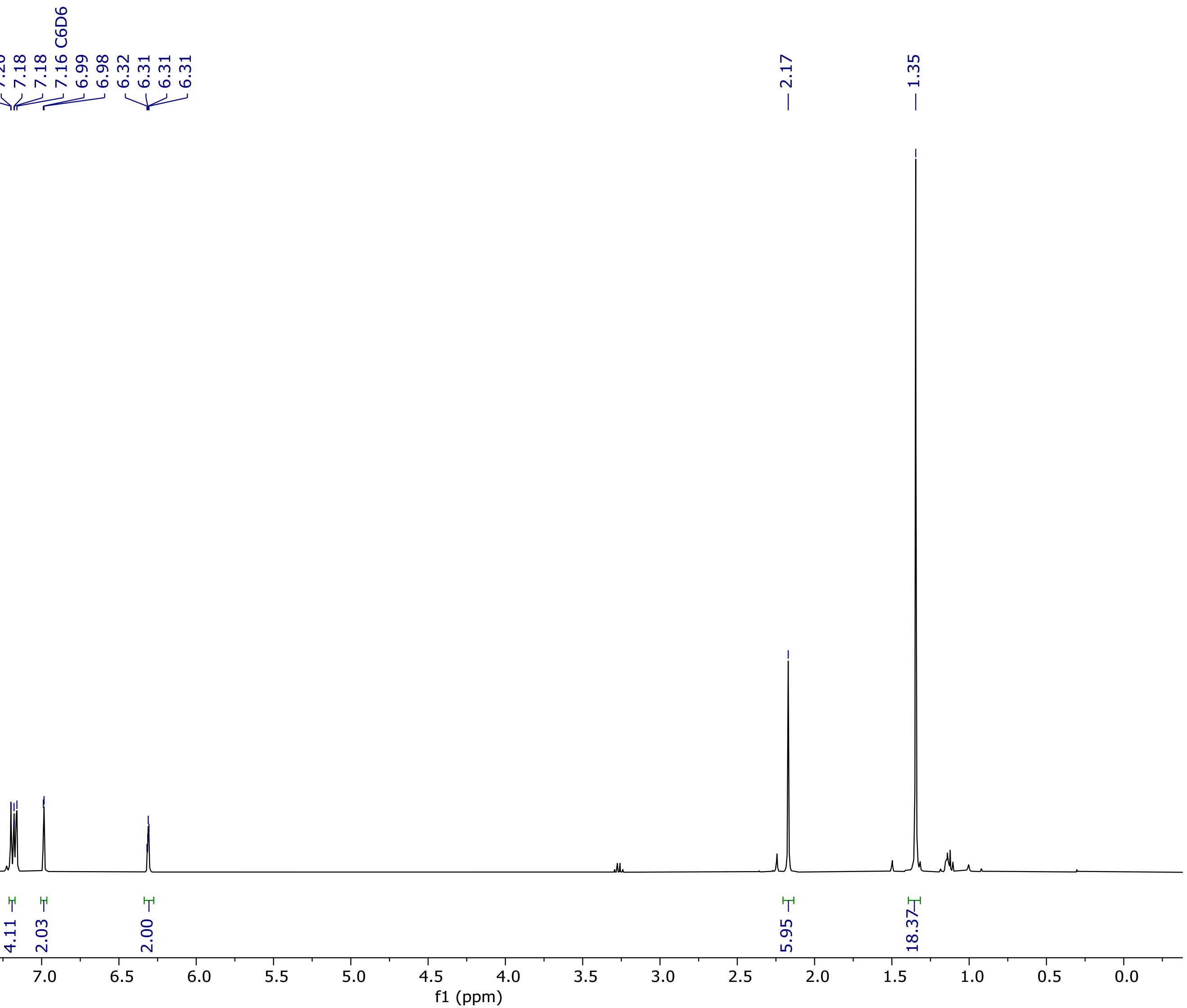

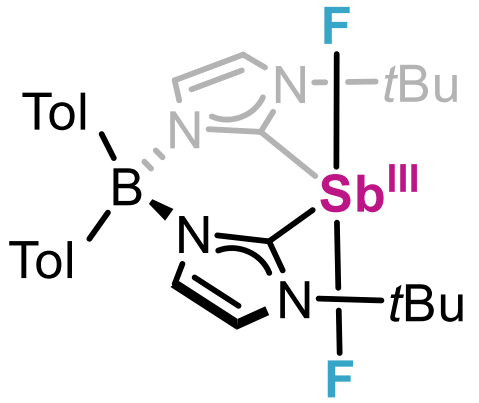

15

$^{19}F\{^{1}H\}$ NMR, C_6D_6

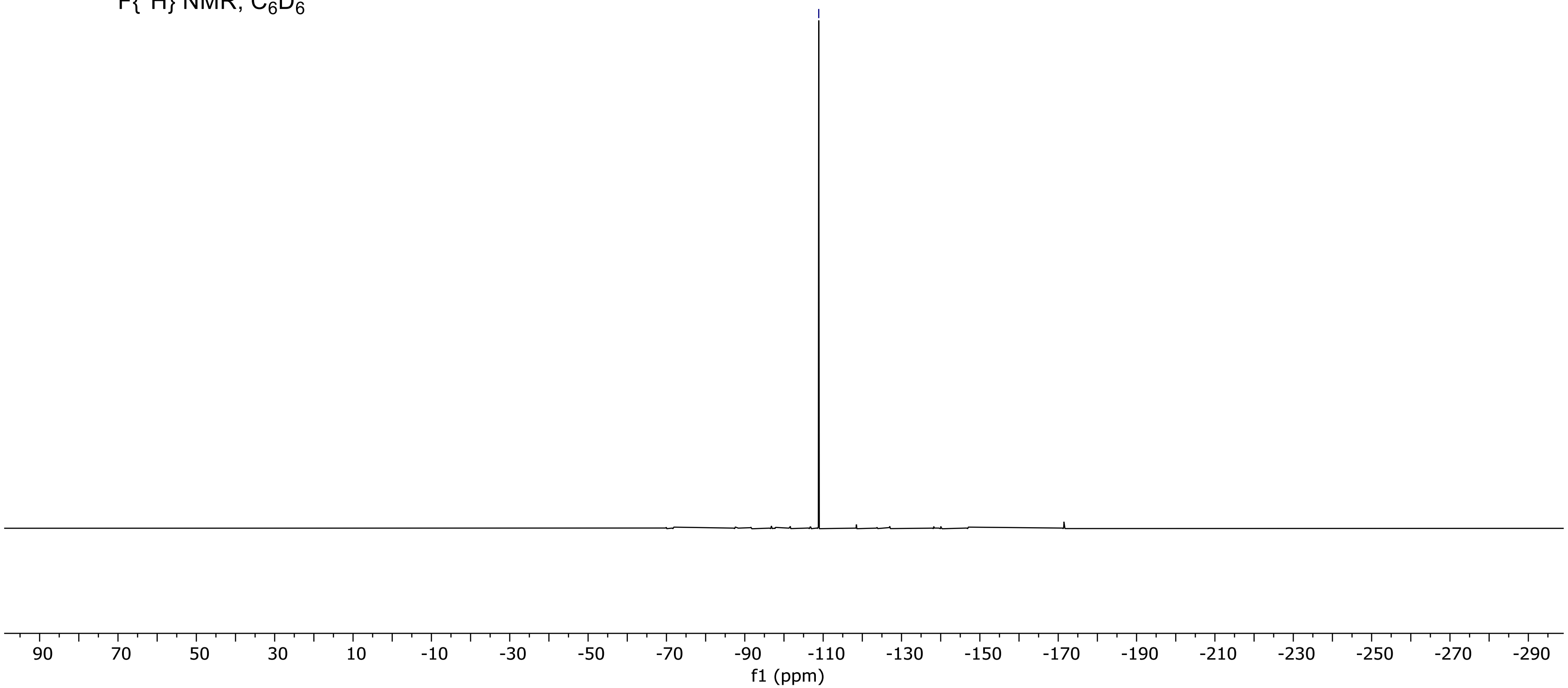

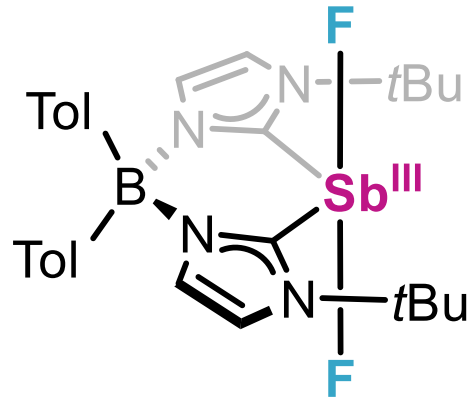

15

^{11}B NMR, C_6D_6

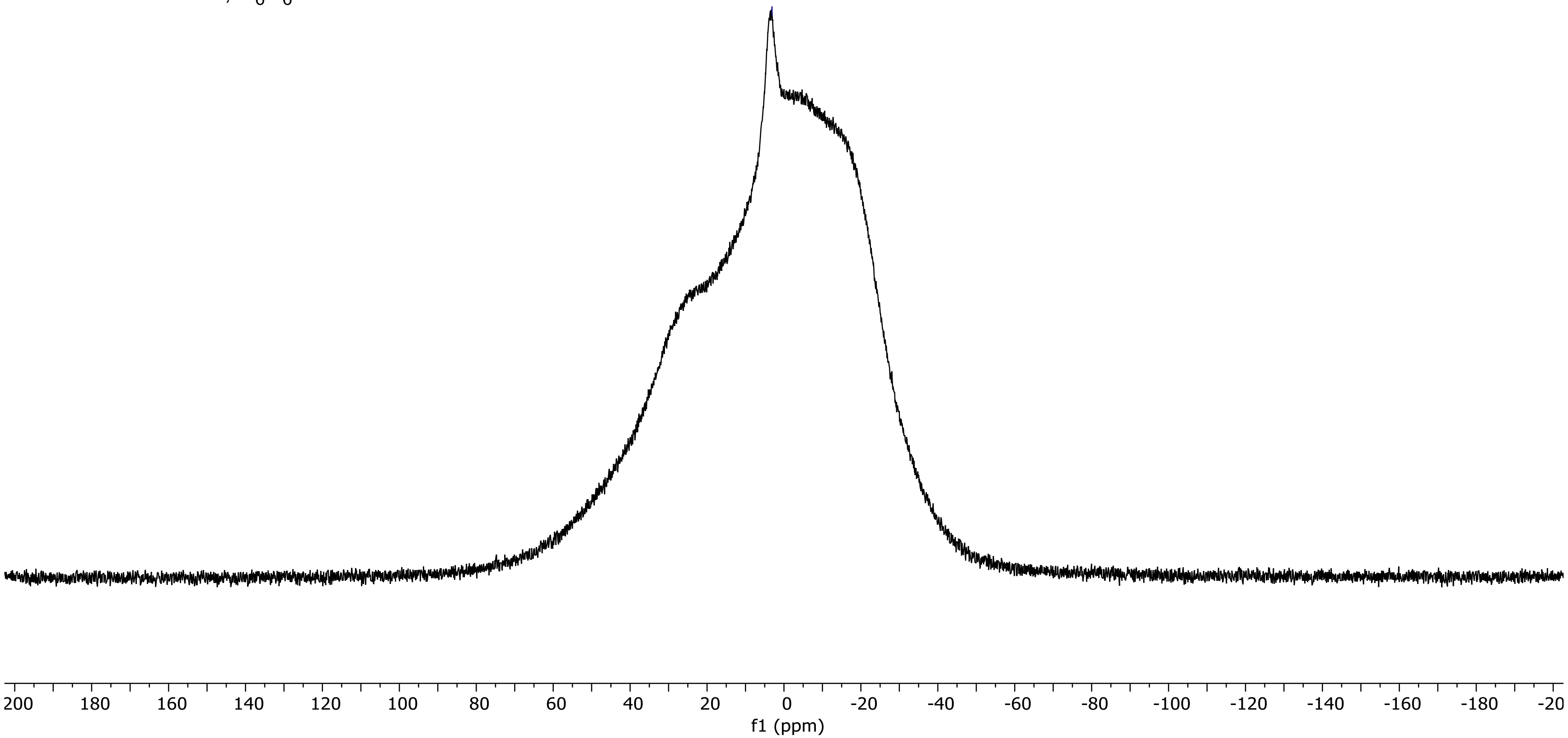

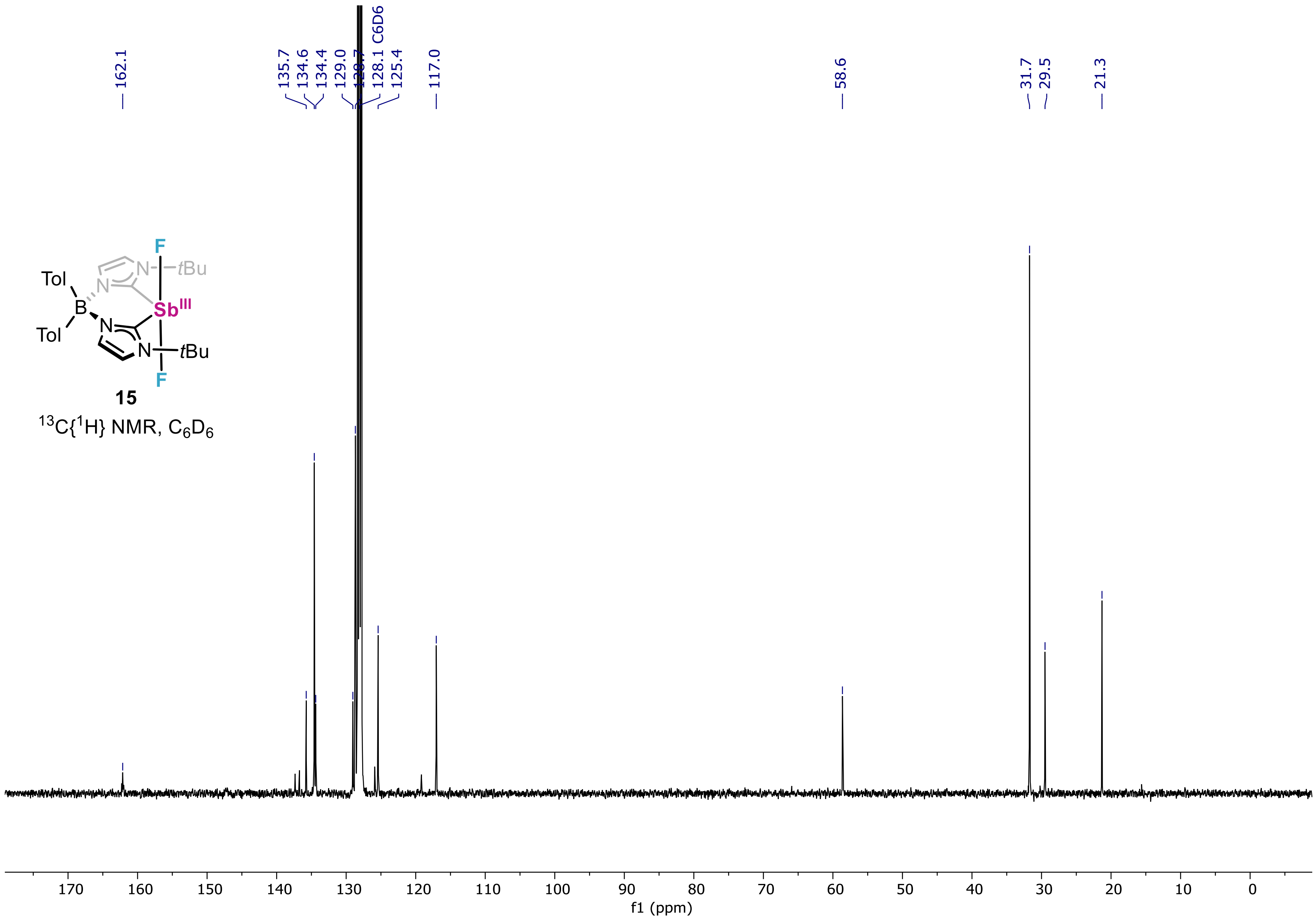

162.1
135.7
134.6
134.4
129.0
128.7
128.1 C6D6
125.4
117.0
58.6
31.7
29.5
21.3
F
N
N
tBu
Tol
B
Sb^III
Tol
N
N
tBu
F
15
13C{1H} NMR, C6D6
170
160
150
140
130
120
110
100
90
80
70
60
50
40
30
20
10
0
f1 (ppm)

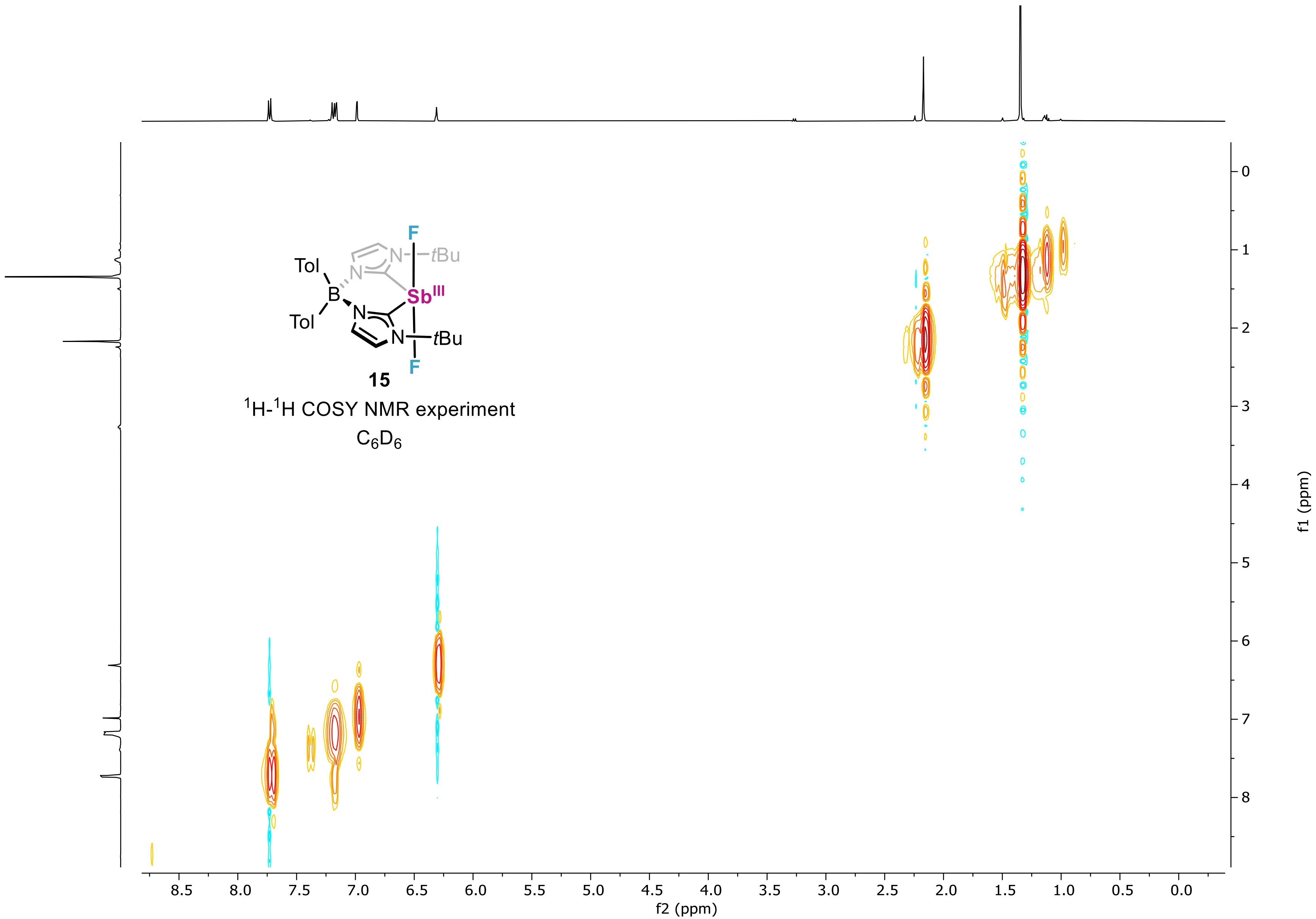
F
N
N
tBu
Tol
B
Tol
N
N
Sb^{III}
tBu
F
15
^{1}H-^{1}H COSY NMR experiment
C_6D_6
f2 (ppm)
f1 (ppm)

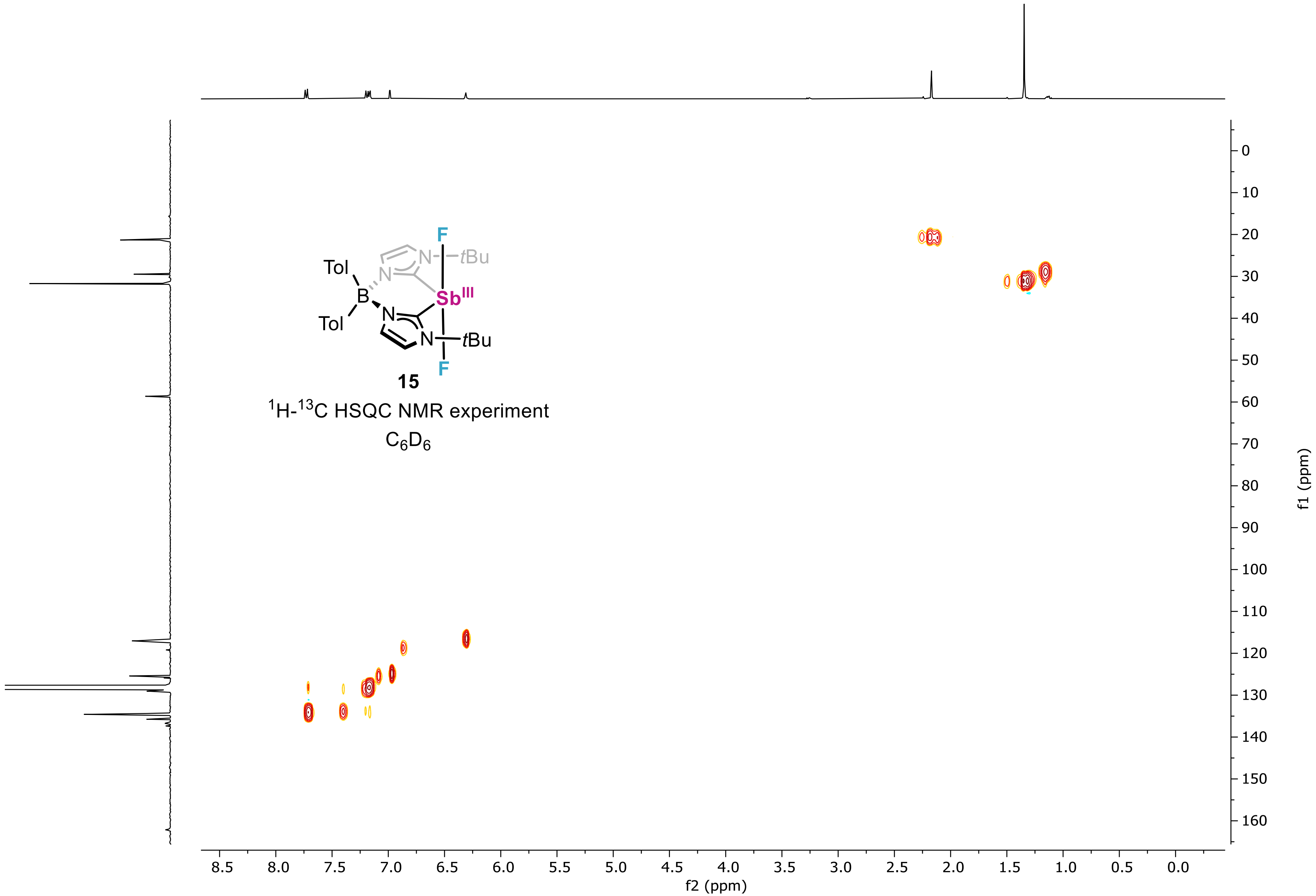

^{1}H-^{13}C HSQC NMR experiment

C_6D_6

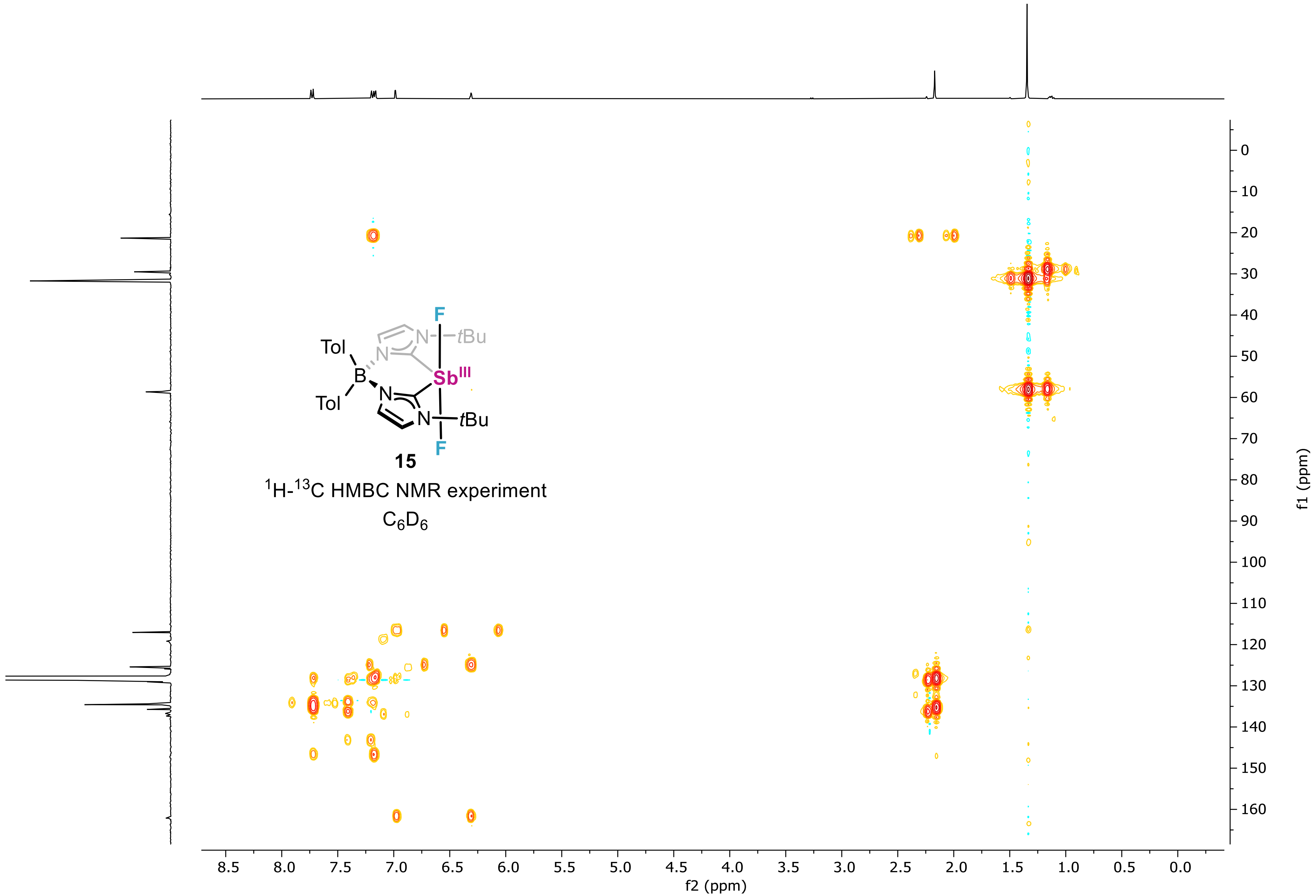

F
N
tBu
Tol
N
B
Sb^III
Tol
N
N
tBu
F
15
1H-13C HMBC NMR experiment
C6D6
f2 (ppm)
f1 (ppm)

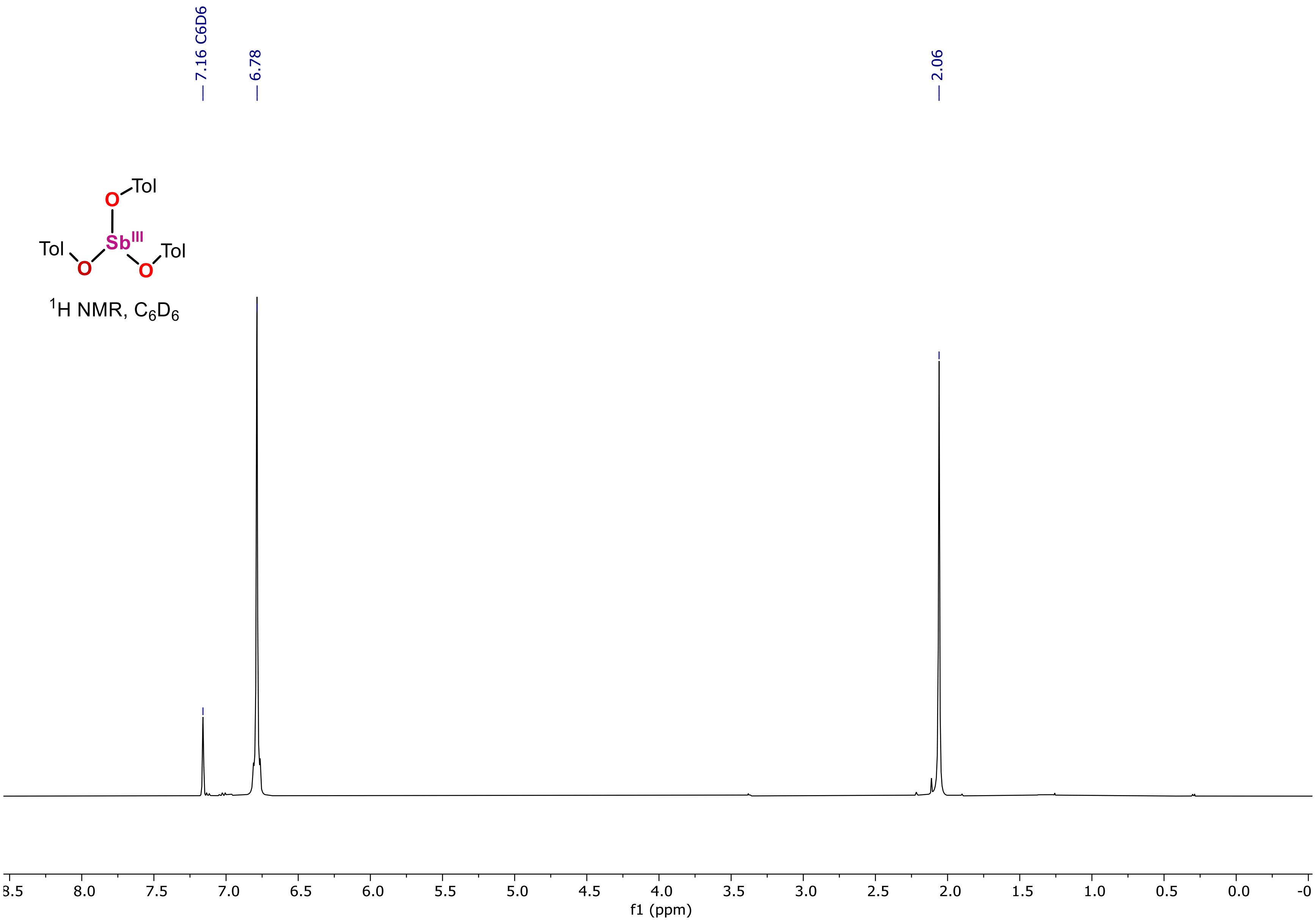

Tol
O
Sb^III
Tol
O
O
Tol
^{1}H NMR, C_6D_6
7.16 C6D6
6.78
2.06
8.5
8.0
7.5
7.0
6.5
6.0
5.5
5.0
4.5
4.0
3.5
3.0
2.5
2.0
1.5
1.0
0.5
0.0
-0
f1 (ppm)

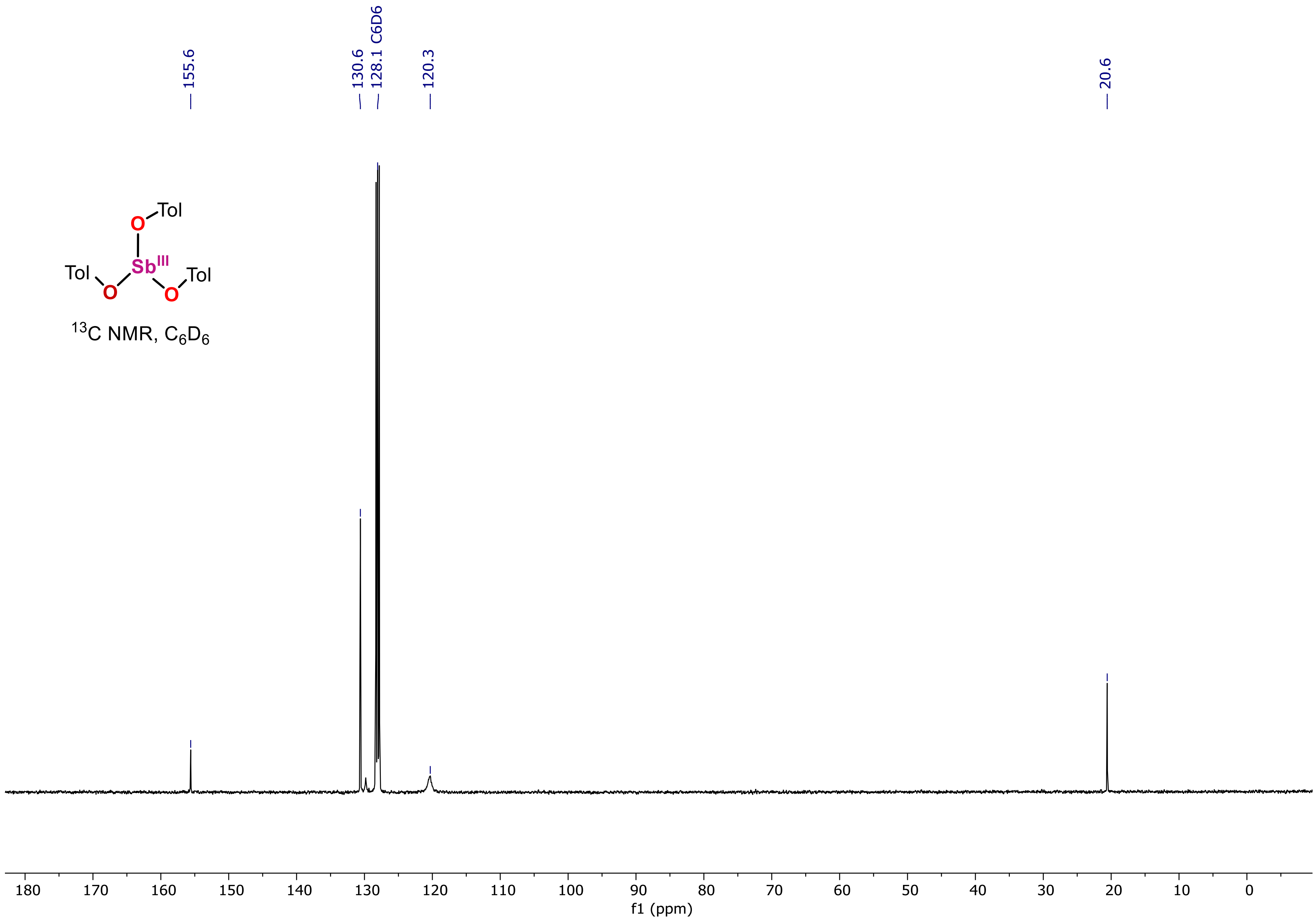
Tol
O
Sb^III
Tol
O
O
Tol
13C NMR, C6D6
155.6
130.6
128.1 C6D6
120.3
20.6
180 170 160 150 140 130 120 110 100 90 80 70 60 50 40 30 20 10 0
f1 (ppm)

8 References

(1) Fernando, S.; Chan, Y. C.; Fernandez, S.; Sabater, E.; Tizzard, G.; Coles, S. J.; Andrada, D. M.; Planas, O. Zwitterionic Heavier Pnictinidenes in Redox Catalysis. *Angew. Chem. Int. Ed.* **2025**, *64*, e202505697.

(2) Huang, M.; Li, K.; Zhang, Z.; Zhou, J. Antimony Redox Catalysis: Hydroboration of Disulfides through Unique Sb(I)/Sb(III) Redox Cycling. *J. Am. Chem. Soc.* **2024**, *146*, 20432–20438.

(3) Moores, L. C.; Kaur, D.; Smith, M. D.; Poole, J. S. Regioselectivity of Hydroxyl Radical Reactions with Arenes in Nonaqueous Solutions. *J. Org. Chem.* **2019**, *84*, 3260–3269.

(4) Shellnutt, Z. S.; Koide, K. Atmosphere Effects on Arene Reduction with Lithium and Ethylenediamine in THF. *J. Org. Chem.* **2025**, *90*, 3684–3697.

(5) Wu, J.; Wang, Z.; Chen, X.-Y.; Wu, Y.; Wang, D.; Peng, Q.; Wang, P. Para-Selective Borylation of Monosubstituted Benzenes Using a Transient Mediator. *Sci. China Chem.* **2020**, *63*, 336–340.

(6) Combs, J. R.; Lai, Y.-C.; Van Vranken, D. L. Tandem Insertion/[3,3]-Sigmatropic Rearrangement Involving the Formation of Silyl Ketene Acetals by Insertion of Rhodium Carbenes into S–Si Bonds. *Org. Lett.* **2021**, *23*, 2841–2845.

(7) Pahl, J.; Noone, E.; Uzelac, M.; Yuan, K.; Ingleson, M. J. Borylation Directed Borylation of Indoles Using Pyrazabole Electrophiles: A One-Pot Route to C7-Borylated-Indolines. *Angew. Chem. Int. Ed.* **2022**, *61*, e202206230.

(8) Sekine, M.; Okimoto, K.; Yamada, K.; Hata, T. Silyl Phosphites. 15. Reactions of Silyl Phosphites with.Alpha.-Halo Carbonyl Compounds. Elucidation of the Mechanism of the Perkow Reaction and Related Reactions with Confirmed Experiments. *J. Org. Chem.* **1981**, *46*, 2097–2107.

(9) Saito, T.; Nishiyama, H.; Tanahashi, H.; Kawakita, K.; Tsurugi, H.; Mashima, K. 1,4-Bis(Trimethylsilyl)-1,4-Diaza-2,5-Cyclohexadienes as Strong Salt-Free Reductants for Generating Low-Valent Early Transition Metals with Electron-Donating Ligands. *J. Am. Chem. Soc.* **2014**, *136*, 5161–5170.

(10) Zhang, J.; Wu, J.; Xiong, Y.; Cao, S. Synthesis of Unsymmetrical Biaryl Ethers through Nickel-Promoted Coupling of Polyfluoroarenes with Arylboronic Acids and Oxygen. *Chem. Commun.* **2012**, *48*, 8553–8555.

(11) Begg, C.; Dragomanova, V.; Yufit, D.; Blundell, T.; Cobb, S.; Fox, M.; Kitching, M.; Brittain, W. Enhanced Crystallinity of Tetrahalopyridyl (THP) Derivatized Compounds. ChemRxiv January 23, 2025. https://doi.org/10.26434/chemrxiv-2025-nn3vj.

(12) Mulryan, D.; Rodwell, J.; Phillips, N. A.; Crimmin, M. R. Au(I) Catalyzed HF Transfer: Tandem Alkyne Hydrofluorination and Perfluoroarene Functionalization. *ACS Catal.* **2022**, *12*, 3411–3419.

(13) Brittain, W. D. G.; Cobb, S. L. Tetrafluoropyridyl (TFP): A General Phenol Protecting Group Readily Cleaved under Mild Conditions. *Org. Biomol. Chem.* **2019**, *17*, 2110–2115.

(14) Mulryan, D.; White, A. J. P.; Crimmin, M. R. Organocatalyzed Fluoride Metathesis. *Org. Lett.* **2020**. *22*, 9351–9355

(15) Hsu, C.-M.; Lee, S.-C.; Tsai, H.-E.; Tsao, Y.-T.; Chan, C.-L.; Miñoza, S.; Tsai, Z.-N.; Li, L.-Y.; Liao, H.-H. Desulfurative Ni-Catalyzed Reductive Cross-Coupling of Benzyl Mercaptans/Mercaptoacetates with Aryl Halides. *J. Org. Chem.* **2022**, *87*, 3799–3803.

(16) Singh, A.; Kubik, J. J.; Weaver, J. D. Photocatalytic C–F Alkylation; Facile Access to Multifluorinated Arenes. *Chem. Sci.* **2015**, *6*, 7206–7212.

(17) Coe, P. L.; Rees, A. J.; Whittaker, J. Reactions of Polyfluoropyridines with Bidentate Nucleophiles: Attempts to Prepare Deazapurine Analogues. *J. Fluor. Chem.* **2001**, *107*, 13–22.

(18) Althagbi, H. I.; Edwards, A. J.; Nicholson, B. K.; Reason, D. A.; Saunders, G. C.; Sim, S. A.; van der Heijden, D. A. Arene-Perfluoroarene-Anion Stacking and Hydrogen Bonding Interactions in Imidazolium Salts for the Crystal Engineering of Polarity. *Cryst. Growth Des.* **2016**, *16*, 174–188.

(19) Moradi, T.; Ranjbar-Karimi, R.; Poorfreidoni, A.; Mehrabi, H. Perfluoropyridylation of Indoles via Reaction of Perfluorinated Pyridines with Indole Compounds. *J Iran Chem. Soc* **2020**, *17*, 1347–1357.

(20) Podolan, G.; Jungk, P.; Lentz, D.; Zimmer, R.; Reissig, H.-U. Studies on the Synthesis of Specifically Fluorinated 4-Amino- Pyridine Derivatives by Regioselective Nucleophilic Aromatic Substitution and Catalytic Hydrodefluorination. *Adv. Synth. Catal.* **2015**, *357*, 3215–3228.

(21) Schäfer, S.; Wirth, T. A Versatile and Highly Reactive Polyfluorinated Hypervalent Iodine(III) Compound. *Angew. Chem. Int. Ed.* **2010**, *49*, 2786–2789.

(22) Kundu, G.; Amrutha, P. R.; Tothadi, S.; Sen, S. S. Access to NHC–Boryl Mono- and Bis-Selenide and Utility as Mild Selenium Transfer Reagent Including to the C−F Bond. *Chem. Eur. J.* **2024**, *30*, e202302984.

(23) Bonfante, S.; Lorber, C.; Lynam, J. M.; Simonneau, A.; Slattery, J. M. Metallomimetic C–F Activation Catalysis by Simple Phosphines. *J. Am. Chem. Soc.* **2024**, *146*, 2005–2014.

(24) Vinogradov, A. S.; Platonov, V. E. Synthesis of Perfluorinated Biaryls by Reaction of Perfluoroarylzinc Compounds with Perfluoroarenes. *Russ J Org Chem* **2015**, *51*, 1388–1394.

(25) Neese, F. Software Update: The ORCA Program System—Version 6.0. *WIREs Comput. Mol. Sci.* **2025**, *15*, e70019.

(26) Adamo, C.; Barone, V. Toward Reliable Density Functional Methods without Adjustable Parameters: The PBE0 Model. *J. Chem. Phys.* **1999**, *110*, 6158.

(27) Ernzerhof, M.; Scuseria, G. E. Assessment of the Perdew–Burke–Ernzerhof Exchange-Correlation Functional. *J. Chem. Phys.* **1999**, *110*, 5029.

(28) Grimme, S.; Antony, J.; Ehrlich, S.; Krieg, H. A Consistent and Accurate Ab Initio Parametrization of Density Functional Dispersion Correction (DFT-D) for the 94 Elements H-Pu. *J. Chem. Phys.* **2010**, *132*, 154104.

(29) Grimme, S.; Ehrlich, S.; Goerigk, L. Effect of the Damping Function in Dispersion Corrected Density Functional Theory. *J. Comput. Chem.* **2011**, *32*, 1456.

(30) Weigend, F.; Ahlrichs, R. Balanced Basis Sets of Split Valence, Triple Zeta Valence and Quadruple Zeta Valence Quality for H to Rn: Design and Assessment of Accuracy. *Phys. Chem. Chem. Phys.* **2005**, *7*, 3297.

(31) McIver, J. W. Jr.; Komornicki, A. Structure of Transition States in Organic Reactions. General Theory and an Application to the Cyclobutene-Butadiene Isomerization Using a Semiempirical Molecular Orbital Method. *J. Am. Chem. Soc.* **1972**, *94*, 2625.

(32) Liu, X.; Moon, H. W., Moon; Spinnato, D.; Leutzsch, M.; Cornella, J. Catalytic 1,2-Migratory Insertion in a Bismuth Redox Platform: Reductive Arylation of Aldehydes. *Angew. Chem. Int. Ed.* **2025**, 64, e202510360.
